# Cuproptosis‐related miRNAs signature and immune infiltration characteristics in colorectal cancer

**DOI:** 10.1002/cam4.6270

**Published:** 2023-06-19

**Authors:** Zhonglin Zhu, Tianan Guo, Junyong Weng, Shanbao Li, Congcong Zhu, Qiuyan Zhao, Ye Xu

**Affiliations:** ^1^ Department of Colorectal Surgery Fudan University Shanghai Cancer Center Shanghai PR China; ^2^ Department of Oncology Shanghai Medical College, Fudan University Shanghai PR China; ^3^ Department of General Surgery Shanghai General Hospital, Shanghai Jiao Tong University School of Medicine Shanghai PR China; ^4^ Department of Gastroenterology Shanghai General Hospital, Shanghai Jiao Tong University School of Medicine Shanghai PR China; ^5^ Shanghai Key Laboratory of Pancreatic Diseases Shanghai General Hospital, Shanghai Jiao Tong University School of Medicine Shanghai PR China

**Keywords:** colorectal cancer, cuproptosis, immunotherapy, miRNAs, prognostic model

## Abstract

**Background:**

A novel form of cell death termed cuproptosis was proposed recently. miRNAs play important roles in colorectal cancer (CRC). However, their relationships have not been reported.

**Methods:**

miRNAs that negatively regulate 16 cuproptosis regulators were predicted using Targetscan database. The univariate Cox, LASSO, and multivariate Cox regression analyses were performed to select cuproptosis‐related miRNAs. GSEA and ssGSEA analysis was carried out for functional enrichment analysis. The immune cell proportion score (IPS) and the efficiencies of multiple chemotherapy drugs were compared between different risk groups. The CCK8, cell colony, edu, and flow cytometry assays were performed to validate the roles of miRNA. Luciferase reporter assay confirmed the regulatory mechanism of miRNA on cuproptosis.

**Results:**

Six cuproptosis‐related miRNAs (hsa‐miR‐653, hsa‐miR‐216a, hsa‐miR‐3684, hsa‐miR‐4437, hsa‐miR‐641, and hsa‐miR‐552) were screened out for model construction. The risk score could act as an independent prognostic indicator in CRC (*p* < 0.001, 95% HR = 1.243 (1.129–1.369)). The nomogram could efficiently predict the overall survival rate (AUC = 0.836). Then, the level of immunosuppressive pathways, immunosuppressive cells, stromal‐activated genes, and stromal score was higher in the high‐risk group. The IPS analysis showed a better response to immunotherapy in the low‐risk group. Also, the risk score was closely correlated with efficiencies of multiple chemotherapy drugs. Furthermore, miR‐653 was highly expressed in CRC tissues (*p* < 0.001), closely correlated with T stage (*p* < 0.001), metastasis (*p* < 0.001), and tumor stage (*p* < 0.001). High expression of miR‐653 predicted a shorter overall survival (*p* = 0.0282) and disease‐free survival (*p* = 0.0056). In addition, miR‐653 promoted cell proliferation, inhibited apoptosis, and negatively regulated the expression of DLD through directly binding to the 3’‐UTR of DLD mRNA.

**Conclusion:**

We constructed a cuproptosis‐related miRNA signature for the prediction of CRC patient survival and immunotherapy sensitivity. miR‐653 was highly expressed in CRC tissues, promoted cell proliferation, and inhibited apoptosis by negatively regulating the expression of DLD.

## Graphical abstract

In the manuscript, we systemically discussed a new format of cell death mechanism—cuproptosis, distinct from other known mechanisms including necrosis, apoptosis, autophagy, necroptosis, pyrotosis, and ferroptosis. We constructed a cuproptosis‐related prognosis model with 6 cuproptosis‐related miRNAs. The nomogram was constructed and could efficiently predict the overall survival rate of 1, 3, and 5 years. The cuproptosis‐related prognosis model was closely correlated with clinical‐pathological features and immune cell infiltration. In addition, the risk score contributes to predicting the efficiencies of multiple chemotherapy and immunotherapy drugs.

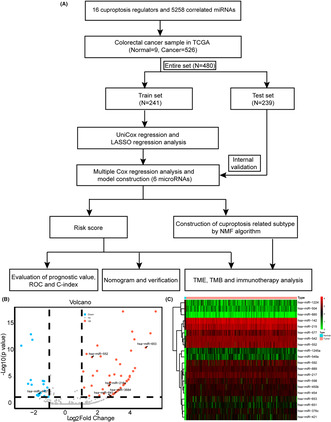

## 1 INTRODUCTION

Colorectal cancer (CRC) is the third leading cause of cancer‐related mortality worldwide.^1^ As one of the most common malignancies, CRC has the characteristics of easy metastasis and recurrence. The 5‐year survival rate of metastatic CRC is less than 20%.^2^ Thus, further exploration of the underlying mechanism of CRC progression and effective treatments for patients is urgent.^3^ In the past decade, immune checkpoint inhibitors (ICI) including antibodies of anti‐PD‐1/PD‐L1 and anti‐CTLA4 have benefited CRC patients with advanced stage.^4^, ^5^ High tumor mutation burden (TMB) has been posited as an immunotherapy biomarker for many cancer types.^6^ CRC tissues that have high TMB often possessed more cytotoxic T‐cell infiltration and largely responded to immunotherapy,^7^, ^8^ while low TMB and lack of immune cell infiltration contributed to immune resistance.^9^

Cell death is critical in the development of mammals and is essential for various biological processes. Cell death can be executed through several patterns, including apoptosis, necroptosis, pyroptosis, ferroptosis, alkaliptosis, oxeiptosis, and so on.^10^ Among them, ferroptosis, as an iron‐dependent cell death induced by excessive lipid hydroperoxides, was proved to be involved in a series of pathological features.^11^, ^12^ Similar to iron, copper is also one of the essential heavy metal ions in organisms. The researchers discovered that copper was essential for the activity of the autophagic kinases ULK1/2 to regulate autophagy and lung tumor growth.^13^ Copper is also a cofactor for enzymes that mediate mitochondrial respiration,^14^ kinases signaling,^15^ oxidative stress,^16^ and lipid metabolism.^17^ Excessive abundance of copper can trigger cell death. However, the exact mechanism remained unclear. Recent research revealed that too much copper accumulation in the mitochondria contributes to dihydrolipoamide S‐acetyltransferase (DLAT) aggregation, disturbs tricarboxylic acid (TCA) cycle, and leads to cell death. Researchers termed this novel form of cell death as cuproptosis.^18^ This novel mechanism will shed new light on copper‐related disease, including cancer.

miRNAs are short noncoding RNAs with 18–25 nucleotides.^19^ miRNAs exert biological effects by binding to 3′ untranslated region of the target mRNAs, causing degradation of the mRNAs or inhibiting their translation.^20^ Multiple studies have revealed miRNAs play crucial roles in CRC progression.^21^, ^22^, ^23^, ^24^ For example, miR‐146a inhibits colonic inflammation and inflammation‐associated CRC by modulating IL‐17 responses.^25^ MIR22HG facilitates immunotherapy in CRC via mediating TGFβ/SMAD signaling. In addition, the researchers discovered that decreased MIR22HG promoted CRC epithelial‐mesenchymal transition. These findings suggest MIR22HG may act as a potential therapy target in CRC.^26^ To date, the associations between cuproptosis and miRNAs in CRC have not been reported. Therefore, it will be of great significance to explore the cuproptosis‐related miRNAs in CRC.

Herein, we identified 6 cuproptosis‐related miRNAs, which are closely associated with the prognosis of CRC patients, and constructed a risk prediction model. Further analysis revealed that the cuproptosis‐related miRNAs signature could be used for prognosis evaluation and immunotherapy selection. What's more, we demonstrated that miR‐653 was highly expressed in colorectal cancer, closely correlated with clinicopathological features, and promoted cell proliferation by negatively regulating the expression of DLD.

## 2 MATERIALS AND METHODS

### 2.1 Data collection

The miRNA and mRNA expression data and relevant clinicopathological information on CRC were downloaded from The Cancer Genome Atlas (TCGA) database. The miRNA data includes 9 normal and 533 cancer tissues, and the mRNA data includes 51 normal and 638 cancer tissues. The intersect tissues include 9 normal and 526 cancer tissues. The work was conducted based on the 535 CRC tissues. In addition, 92 paired fresh frozen colorectal cancer tissues and adjacent normal tissues were collected from our hospital and stored in liquid nitrogen. All clinicopathological features were diagnosed by two pathologists. The present study was approved by the Ethics Committee of Fudan University Shanghai Cancer Center.

### 2.2 Obtain cuproptosis‐related miRNAs

A total of 16 cuproptosis regulators: FDX1, MTF1, DBT, CDKN2A, DLST, DLAT, LIPT1, LIAS, GLS, DLD, PDHA1, PDHB, GCSH, SLC31A1, ATP7A, and ATP7B, were collected from lipoylated TCA cycle pathway of copper‐induced cell death^18^ and copper transport protein.^27^, ^28^ The Targetscan database was used to predict the potential miRNAs of these cuproptosis regulators. Then, the expressions of these potential miRNAs were compared between normal and cancer tissues from TCGA database. The differentially expressed miRNAs were selected with the criteria of log2 fold change >1 or <−1 and FDR <0.05.

### 2.3 Construction of cuproptosis‐related miRNAs signature

The CRC patients of TCGA database were divided into train group and test group by geometric proportion delamination sample method with the function of createDataPartition (package “caret”). Then, prognostic cuproptosis‐related miRNAs were identified through univariate regression analysis, and these miRNAs were further analyzed to establish risk score by LASSO and multiple Cox regression model. The risk score of each sample was calculated as below:

$$\text{risk score}=\sum_{i=1}^{n}\text{coef}\;\left({\text{miRNA}}_{i}\right)\times \exp\;\left({\text{miRNA}}_{i}\right)$$

where coef (miRNA_*i*_) and exp (miRNA_*i*_), respectively, represent the risk coefficient and expression of each miRNA. Based on the median risk score, the samples were classified into high‐risk and low‐risk groups. The Kaplan–Meier curve was performed by R package “survminer.” To evaluate the accuracy of signature, the receiver operating characteristic (ROC) curve was conducted by R package “timeROC.” Via R package “survival,” “regplot,” and “rms,” a nomogram was established to predict overall survival rates of 1, 3, and 5 years.

### 2.4 Function enrichment analysis

Gene set enrichment analysis (GSEA) was conducted to assess related biological roles and potential functional pathways between the two groups using the hallmark gene sets (v7.5.1) and KEGG gene sets (v7.5.1).^29^ |NES| >1 and *p* < 0.05 were considered significantly different.

### 2.5 Immune infiltration analysis

Several computational methods including CIBERSORT, EPIC, MCPCOUNTER, QUANTISEQ, TIMER, and XCELL were employed to evaluate the immune infiltration. Single‐sample gene set enrichment analysis (ssGSEA) was used to analyze the difference in tumor‐infiltrating immune cells between the two risk groups. ESTIMATE algorithm was used to assess the immune score, stromal score, and estimate score of all the samples. The consensus molecular subtype (CMS) of CRC was evaluated with “CMScaller” package.

### 2.6 Somatic mutation analysis

The somatic mutation data were downloaded from the TCGA database. The significantly mutated genes and TMB were verified with R package “maftool.”

### 2.7 Involvements of the risk score in chemotherapy and immunotherapy

The immune cell proportion score (IPS) data of TCGA samples was assessed from The Cancer Immunome Altas (https://tcia.at/home). The IPS was compared between the high‐ and low‐risk groups. The “pRRophetic” package was used to assess the efficiencies of different anti‐tumor medicines. The half‐maximal inhibitory concentration (IC50) of 251 common chemotherapy medicines was compared between different risk groups.

### 2.8 RNA extraction and quantitative real‐time PCR (qPCR)

The total RNA of cells and tissues was extracted with TRIzol (TaKaRa, Japan). 500 ng total RNA was reversely transcribed for cDNA with primeScript™ RT reagent kit (TaKaRa). Relative RNA expression was detected by qPCR with SYBR Premix Ex Taq™ (TaKaRa). U6 was used as miRNA control. The specific primers were presented as follows: miR‐653, loop: GTCGTATCCAGTGCAGGGTCCGAGGTATTCGCACTGGATACGACGGTTCA, Forward: CGCGCGTTGAAACAATCTCTAC, Reverse: AGTGCAGGGTCCGAGGTATT; U6, loop: GTCGTATCCAGTGCAGGGTCCGAGGTATTCGCACTGGATACGACAAAATATGG, Forward: GCTCGCTTCGGCAGCACATATAC, Reverse: AGTGCAGGGTCCGAGGTATT; DLD, Forward: GAAAAATGAAACACTTGGTGGAACA, Reverse: CGCCATCAGCTTTCGTAGC; MTF1, Forward: GCCGCGGAGACAAGTCATTA, Reverse: GCCCTCTTCACCCCCTACTA; ACTB, Forward: CTTCGCGGGCGACGAT, Reverse: CCACATAGGAATCCTTCTGACC.

### 2.9 CCK8 assay

1000 cells/well were seeded in the 96‐well plate. After culturing for 24 h, 10 μl/well CCK8 (NCM Biotech) was added. Followed by continued incubation for 2 hours, the absorbance was measured at 450 nm. The absorbance was measured for 4 consecutive days. All experiments were executed in triplicate.

### 2.10 Cell colony assay

600 cells/well were seeded in the 6‐well plate at 37°C with 5% CO_2_. After culturing for 2 weeks, the culture medium was discarded and the plates were washed by Phosphate Buffered Saline (PBS). Then, the plates were fixed with 4% polymethanol for 20 min and stained with 0.1% crystal violet for 20 min. Finally, the plates were photographed and calculated. All experiments were executed in triplicate.

### 2.11 Edu assay

The EdU assay was performed by EdU Cell Proliferation Kit with Alexa Fluor 488 (Beyotime Biotechnology). 150,000 cells were seeded in 24‐well plates and cultured for 24 h. The next day, cells were incubated with 50 μM EdU for 2 h. Then, the cells were fixed, permeabilized, and stained by EdU staining. Finally, cell nuclei were stained by Hoechst 33342 for 30 min. Three random fields were selected to calculate cells and photographs. The percentage of EdU‐positive cells was computed by the formula: (EdU cells/Hoechst cells) × 100%. All experiments were performed in triplicate.

### 2.12 Flow cytometry

Cell apoptosis was detected by Annexin V‐PE/7‐AAD Apoptosis Detection Kit (Vazyme). Cells were dissociated by trypsin without EDTA. After washing twice with precooled PBS, the cells were diluted in 100 μL binding buffer. Then, 5 μL Annexin V‐PE and 5 μl 7‐AAD were added into the above binding buffer. After incubation for 10 min at room temperature, 400 μL binding buffer was added. Finally, the cell apoptosis state was analyzed by flow cytometry. All experiments were performed in triplicate.

### 2.13 Western blotting

Cells were lysed with RIPA lysis buffer supplemented with 1% phenylmethanesulfonyl fluoride. Then, the protein concentration was measured with BCA Protein Assay Kit (Beyotime). After adding SDS‐PAGE Sample Loading Buffer, the cell lysis was boiled at 100°C for 30 min. Protein lysates were separated by SDS‐PAGE and transferred onto PVDF membranes. The membranes were blocked with 5% fat‐free milk for 1.5 h at room temperature and incubated with primary antibodies (GAPDH, 1:1000, ABclonal; DLD, 1:1000, ABclonal) at 4°C overnight. Next day, the membranes were incubated with HRP‐conjugated secondary antibody for 2 h at room temperature. The specific protein was imaged with ECL reagent (Millipore).

### 2.14 Luciferase reporter assay

The luciferase reporter plasmids (Wild type, containing DLD 3′‐UTR sequence; Mutant type, containing mutant DLD 3′‐UTR sequence in binding site with miR‐653) were generated by HarO Life Co. The overexpression and knockdown vector of miR‐653 were constructed in RiBoBio. The luciferase reporter plasmids were co‐transfected into cells with mimics, inhibitor, or negative control using Lipofectamine™ 2000 reagent. After 48 h, the firefly luciferase and renilla luciferase activity were detected with luciferase reporter assay kit (Promega). Then, the ratio of firefly luciferase/Renilla luciferase activity was calculated.

### 2.15 Statistical analyses

The data were analyzed with R software (version 4.1.2) and Strawberry Perl (version 5.1.0). *p* value < 0.05 was deemed as being statistically significant.

## 3 RESULTS

### 3.1 Identification of cuproptosis‐related miRNAs

**FIGURE 1:**
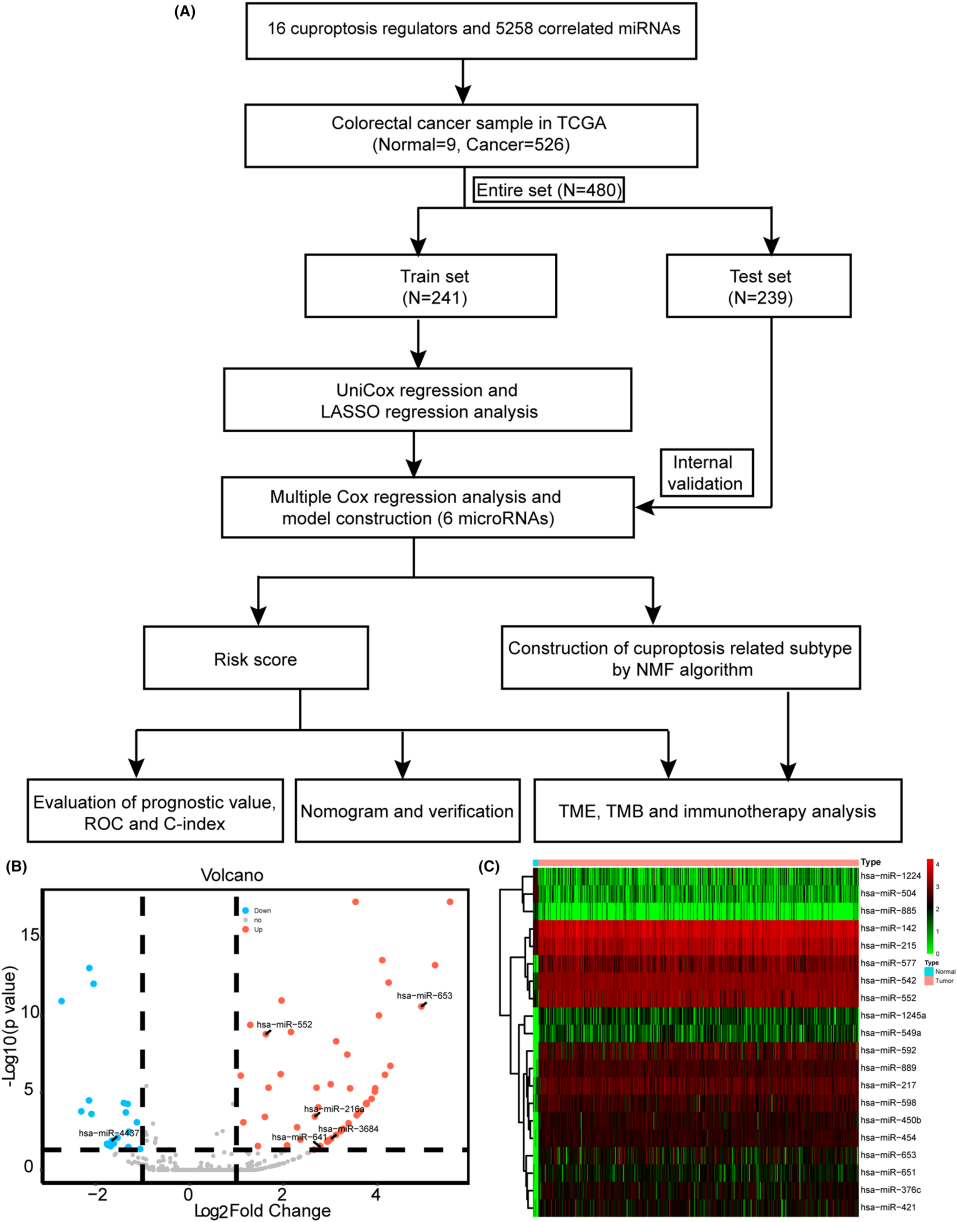
The process of this work and comparison of cuproptosis miRNAs between normal and cancer tissues in TCGA data. (A) The process of this work. (B) The volcano plot of differentially expressed cuproptosis miRNAs between normal and cancer tissues in TCGA data. (C) The heatmap of the top 20 differentially expressed cuproptosis miRNAs.

The process of our study was described in Figure 1A. Sixteen cuproptosis regulators were gathered from lipoylated TCA cycle pathway and transport of copper in recent publication. A total of 5258 miRNAs were predicted to negatively regulate the expression of these 16 cuproptosis regulators with Targetscan database (Table S1). The expression of the 5258 miRNAs in CRC of TCGA database was analyzed, and 72 differentially expressed miRNAs (|Log2 fold change| >1 and FDR <0.05) between normal and CRC samples were identified and visualized in Figure 1B (Table S2). The top 20 differentially expressed miRNAs were shown in Figure 1C.

### 3.2 Construction of prognosis model based on cuproptosis‐related miRNAs

**FIGURE 2:**
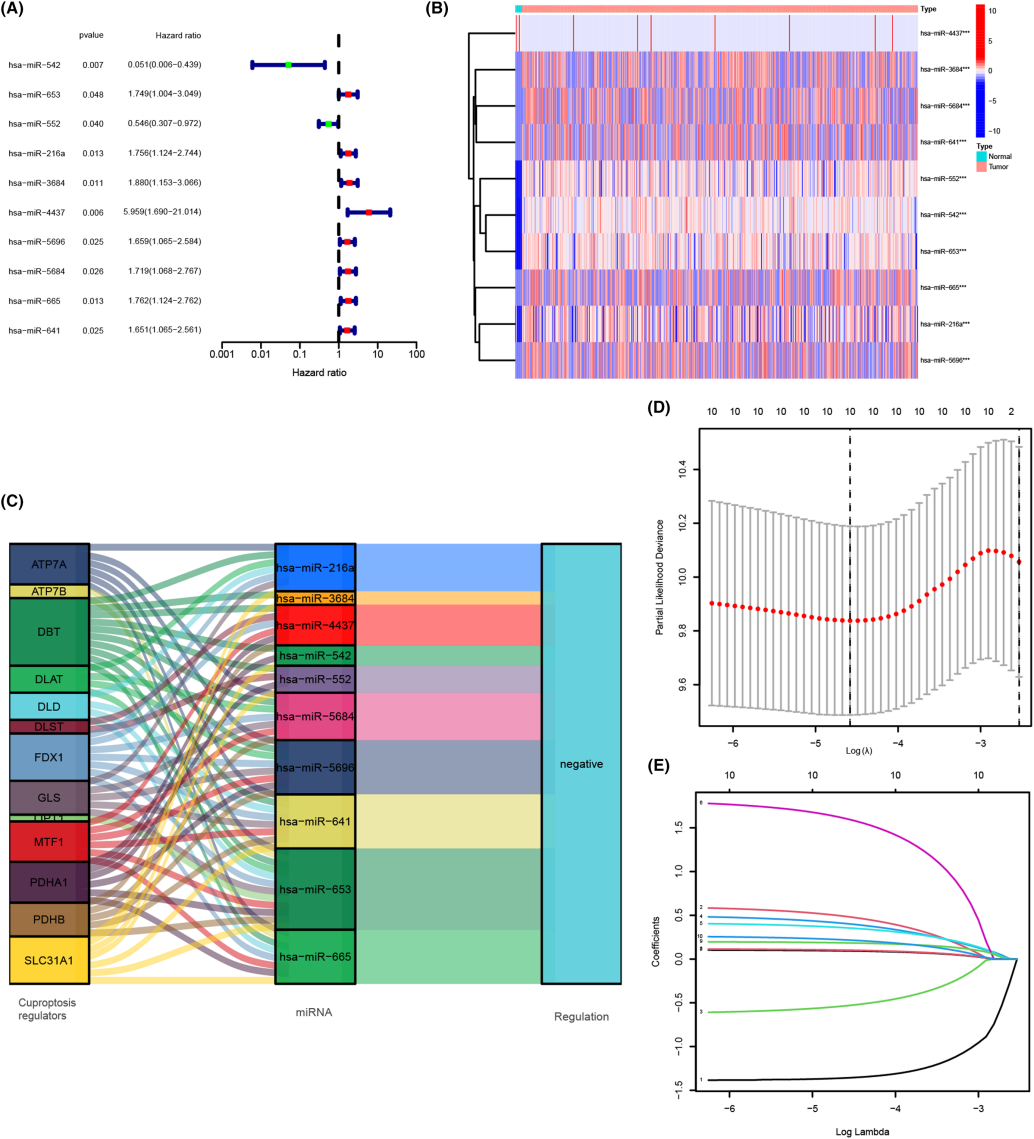
Identification of cuproptosis‐related miRNAs. (A) The results of univariate Cox regression analysis. (B) The heatmap of 10 prognosis‐related miRNAs expression in TCGA data. (C) Sankey diagram showed the correlations between cuproptosis regulators with prognosis‐related miRNAs. (D) The photo of cross‐validation in the LASSO model. (E) LASSO coefficient of prognosis‐related miRNAs.

Before the prognosis model was established, the CRC samples with complete pathological information were randomly divided into train set (*n* = 241) for model building and test set (*n* = 239) for validation. From the above 72 miRNAs, the univariate Cox regression analysis showed that 10 miRNAs were finally confirmed to be related to the prognosis of CRC patients in the train group (Figure 2A). The heatmap of differential expression analysis indicated that has‐miR‐3684, has‐miR‐5684, has‐miR‐641, has‐miR‐552, has‐miR‐542, has‐miR‐653, has‐miR‐665, has‐miR‐216a and has‐miR‐5696 were highly expressed in CRC tissues, while has‐miR‐4437 was lowly expressed (Figure 2B). Furthermore, the Sankey diagram showed the regulations between the 10 miRNAs and 16 cuproptosis regulators (Figure 2C). LASSO analysis further optimized the prognosis‐related miRNAs (Figure 2D,E). Finally, the multivariate Cox regression model analysis was adapted and the optimum prognostic model was constructed with 6 miRNAs (*p* < 0.05). The weighted coefficients of 6 miRNAs were shown in Table S3. The Kaplan–Meier curves indicated that patients with low expression of has‐miR‐216a, has‐miR‐552, has‐miR‐641, has‐miR‐653, or has‐miR‐3684 showed a longer overall survival (Figure S1A–E), respectively.

**FIGURE 3:**
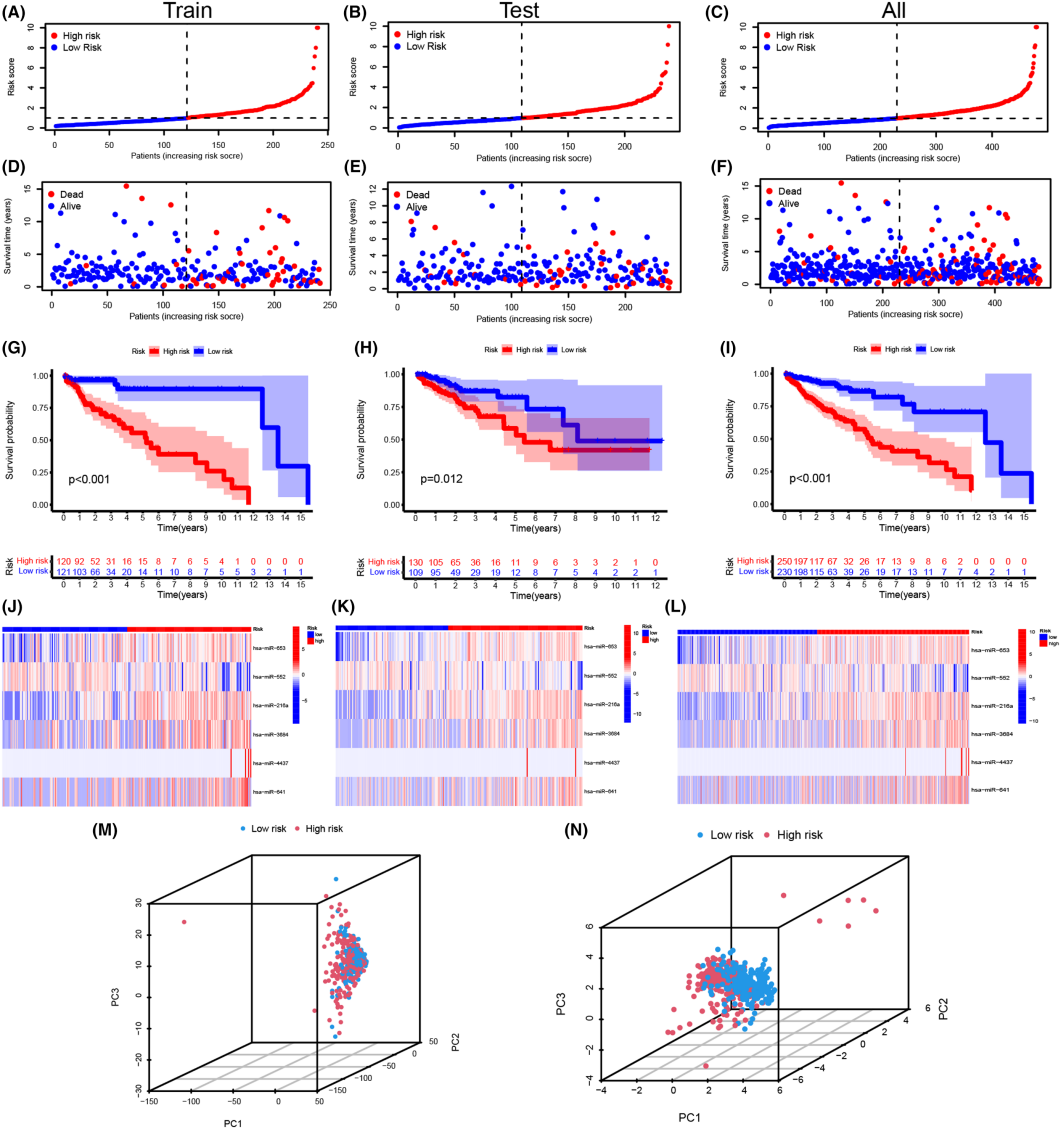
Construction of the cuproptosis‐related miRNA prognosis model. (A–C) The distribution of risk score in the train set, test set, and all samples, respectively. (D–F) The distribution of survival time and survival status in the above three sets. (G–I) The Kaplan–Meier survival curves of overall survival. (J–L) The heat map of 6 model miRNAs expression. (M, N) The PCA with all miRNAs and 6 model miRNAs for all the samples.

The risk score of all CRC samples was calculated, and the samples were divided into high‐ and low‐risk groups in the train set, test set, and all samples set, respectively (Table S4). The risk score of each patient was represented by scatterplot as shown in Figure 3A–C and the survival state of each CRC patient was shown in Figure 3D–F. Moreover, the death cases increased gradually with the increase in risk score. Kaplan–Meier survival curves demonstrated that the overall survival of the high‐risk group was shorter than that of the low‐risk group (Figure 3G–I). The heatmap exhibited that has‐miR‐653, has‐miR‐216a, has‐miR‐3684, has‐miR‐4437, and has‐miR‐641 were highly expressed in the high‐risk group, while has‐miR‐552 was highly expressed in the low‐risk group (Figure 3J–L). These results suggest that this new signature could effectively predict CRC patients' overall survival. In addition, the high‐risk group exhibited poorer clinicopathological features in stratified age, T, N, M, and stage (Figure S2). Furthermore, PCA analysis proved that CRC samples could not be distinguished by all cuproptosis‐related miRNAs, while were significantly distinguished by the 6 prognosis‐related miRNAs (Figure 3M,N).

### 3.3 Evaluation of cuproptosis‐related miRNA prognosis model

**FIGURE 4:**
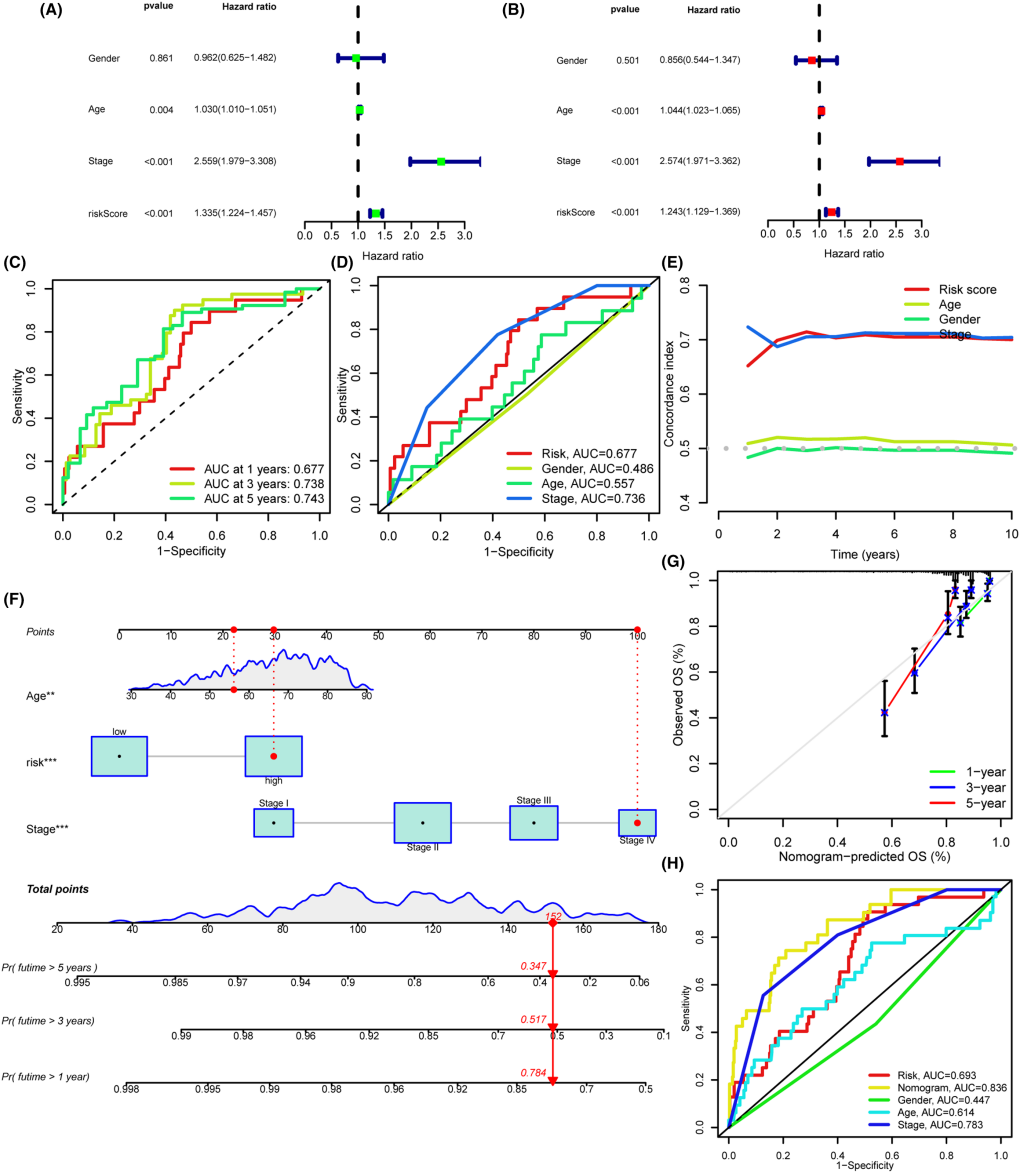
Appraisal of cuproptosis‐related miRNA prognosis model and construction of Nomogram. (A, B) The univariate and multivariate Cox regression analysis of overall survival in all samples. (C) The 1‐, 3‐, and 5‐year ROC of risk score. (D) The 1‐year ROC of risk score, gender, age, and stage. (E) The C‐index of risk score, age, gender, and stage. (F) The nomogram was generated. (G) The calibration curves for the probabilities of 1, 3, and 5 years. (H) The 1‐year ROC of nomogram, risk score, age, gender, and stage.

To verify whether the risk score could be an independent prognostic factor for CRC, the univariate and multivariate Cox regression analyses were conducted. As shown in Figure 4A,B, the HRs of risk score were 1.335 and 1.243 both with *p* < 0.05, indicating risk score as an independent prognostic poor indicator in CRC. Furthermore, the area under the curve of ROC of the train set is 0.677, 0.738, 0.743 at 1, 3, and 5 years (Figure 4C). Moreover, the value of risk score at 1 year was higher than that of gender and age, implying a better prediction efficiency for risk score, while the stage possessed the same prediction efficiency as risk score (Figure 4D). The C‐index confirmed the same results (Figure 4E). Meantime, the area under the curve of ROC in the all samples set (Figure S3A) and test set (Figure S3E) also verified the sensitivity and specificity of the risk score. In addition, the results of C‐index and area under the curve of ROC in the all samples set (Figure S3B,C) and test set (Figure S3E,F) showed a fine prediction efficiency of risk score for the overall survival. Collectively, this new cuproptosis‐related miRNA prognosis model exhibited an excellent predictive potential for the prognosis of CRC patients.

### 3.4 Construction of a predictive nomogram

Based on the cuproptosis‐related miRNA prognosis model (risk score) and clinicopathological factors (age, TNM stage), we constructed a nomogram to predict CRC patients' 1‐, 3‐, and 5‐year overall survival rate in the TCGA database (Figure 4F). The nomogram calibration plots at 1‐, 3‐, and 5‐year survival showed a good accordance with the actual mortality (Figure 4G). In addition, we generated the 1‐year ROC curve, and the area under the curve of prognostic nomogram was 0.836, which was obviously higher than these of gender, age, and stage (Figure 4H). The above results further support the predominant predictive ability of cuproptosis‐related miRNA prognosis model.

### 3.5 Clinical factors and immune cell infiltration analysis

**FIGURE 5:**
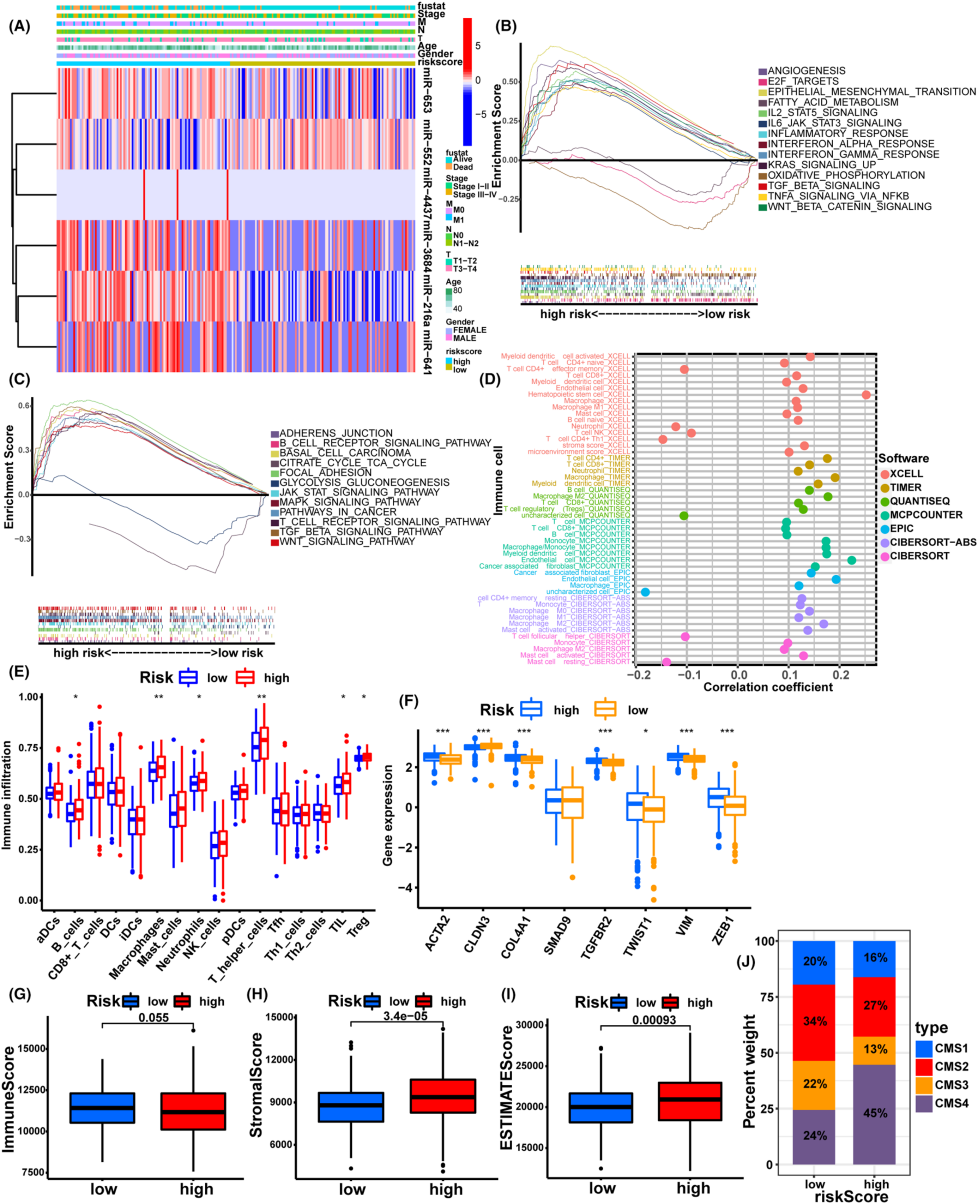
The associations between risk score with clinicopathological features and immune cell infiltration. (A) The heatmap of 6 model miRNAs expression and clinicopathological features in high‐ and low‐risk groups. (B) The heatmap of GSVA with hallmark sets. (C) The heatmap of GSVA with KEGG sets. (D) The immune cell bubble plot. (E) The enrichment of immune cells in high‐ and low‐risk groups by ssGSVA algorithm. (F) The expression of stromal‐activated genes between high‐ and low‐risk groups. (G–I) The immune score, stromal score, and ESTIMATE score in high‐ and low‐risk groups by ESTIMATE algorithm. (J) The distribution of CMS subtypes. *p < 0.05; **p < 0.01; ***p < 0.001.

Although immune checkpoint therapy has achieved significant improvement in clinical treatment, amounts of patients still did not respond to therapy.^30^, ^31^ It is known that the efficiency of immunotherapy was mainly correlated with the baseline immune response and pre‐existing immunity.^32^ The tumor microenvironment (TME) of CRC has been proven to influence the tumor progression and outcomes of immunotherapies.^33^, ^34^ The researchers found that immunoscore in CRC patients was a prognostic and predictive indicator, and the higher cytotoxic T‐cell (CD8+) density indicated a longer survival time.^35^ Then, the relation between clinical features and the two groups was analyzed. The findings demonstrated that the high‐risk group tended to present with a higher frequency of dead status, stage III‐IV, N1‐2, and T3‐4, implying more progressed clinicopathological features (Figure 5A). Further GSEA analysis of tumor hallmarks indicated that oncogenic pathways, including IL6/JAK/STAT3 signaling, KRAS signaling, and Wnt/β‐catenin signaling pathway were engaged in the high‐risk group (Figure 5B). GSEA analysis of KEGG pathway also demonstrated that some immune‐related signaling pathways were enriched in the high‐risk group, including B cell receptor, MAPK, JAK/STAT, T‐cell receptor, TGFβ, and Wnt signaling pathway (Figure 5C). The enrichment of these signaling pathways results in the expression of chemokines that eventually mediate immune escape from the TME.^36^ The immune cells enrichment analysis was performed by multiple algorithms. The bubble plot of immune cell enrichment showed that risk score was positively associated with the enrichment level of hematopoietic stem cell, CD4+ T cell, endothelial cell, M2 macrophage cell, and monocyte cell (Figure 5D, Figure S4). Besides, ssGSEA analysis also indicated high enrichment of B cells, macrophages, Tumor‐infiltrating lymphocytes (TILs), neutrophil, T helper cells, and Treg cells in the high‐risk group (Figure 5E), implicating the immunosuppressive phenotype of the high‐risk group. Besides, the level of stromal‐activated genes, including ACTA2, COL4A1, TGFBR2, TWIST1, VIMENTIN, and ZEB1, were increased significantly in the high‐risk group (Figure 5F). Similarly, the immune score was low in the high‐risk group, while the stromal score and ESTIMATE score in the high‐risk group were higher than those in the low‐risk group (Figure 5G–I, Table S5). To further verify the immune infiltration features of the high‐ and low‐risk groups, CMS of all the samples was evaluated by CMScaller package. The results indicated that the high‐risk group presented with a higher rate of CMS4 compared with the low‐risk group, implicating the immunosuppressive phenotype of the high‐risk group (Table S6). Taken together, these results revealed our cuproptosis‐related miRNA signature can not only serve as a prognostic marker but also reflect the status of immune cell infiltration.

### 3.6 Comparisons of the tumor somatic mutation and immunotherapy

**FIGURE 6:**
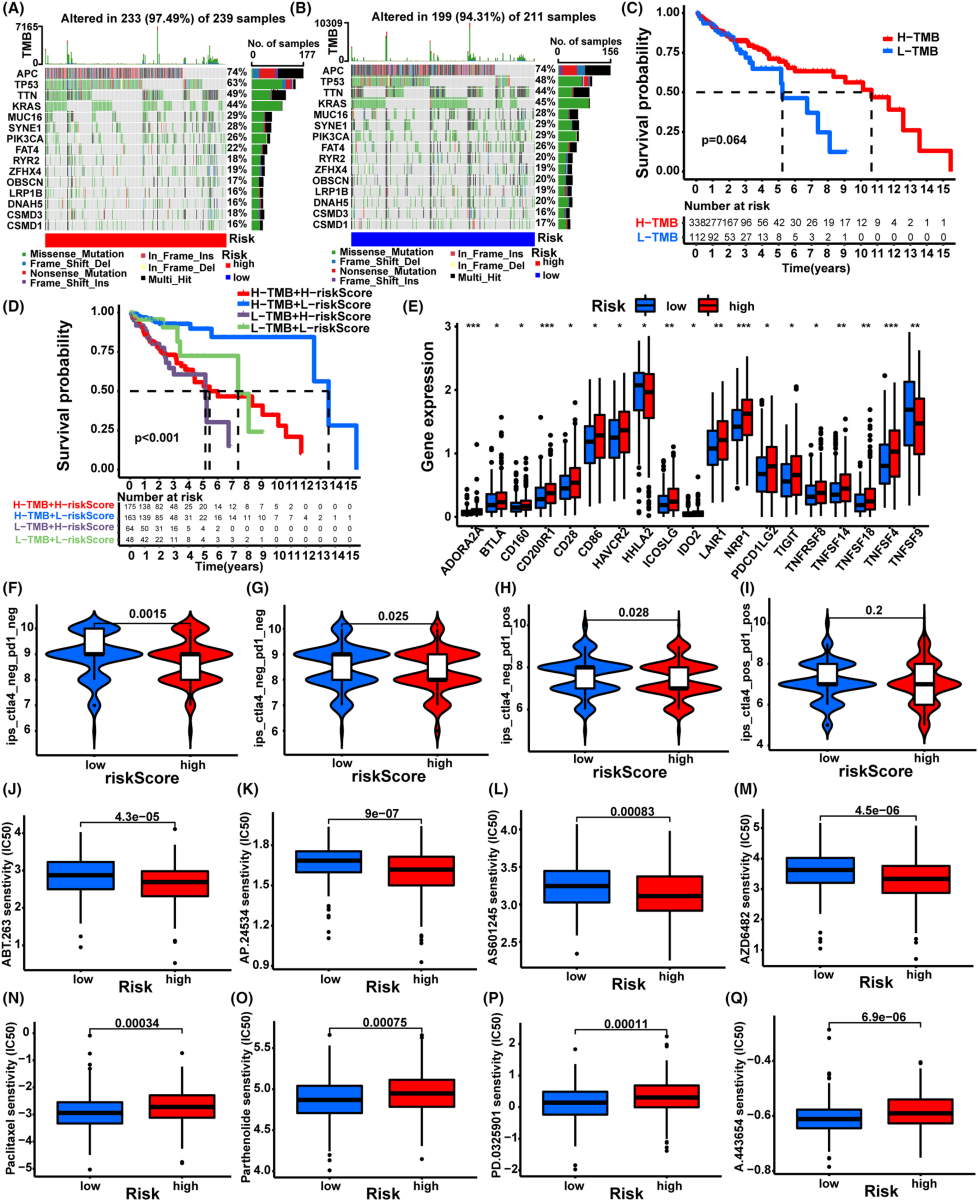
The relationship of the prognosis model with tumor somatic mutation and clinical treatment. (A, B) The waterfall plot of tumor somatic mutation in high‐ and low‐risk groups of TCGA data. (C) The Kaplan–Meier curves of overall survival in different TMB groups. (D) The Kaplan–Meier curves of overall survival in different groups of TMB combination with a risk score. (E) The expression of immune checkpoint genes. (F–I) The IPS in the high‐ and low‐risk groups. (J–Q) The IC50 of anti‐tumor drugs in the high‐ and low‐risk groups. *p < 0.05; **p < 0.01; ***p < 0.001.

Studies have shown that high TMB improved the survival of patients receiving immunotherapy across diverse cancers and TMB may act as a predictive biomarker.^37^ In our study, we found no significant difference in TMB between the high‐ and low‐risk groups (Figure 6A,B). Importantly, we found that CRC patients with higher TMB tended to live longer than patients with lower TBM (Figure 6C). When the TMB and risk score were combined, the patients with higher TMB in the low‐risk group showed longer survival time than those with lower TMB in the high‐risk group (Figure 6D). Next, we examined the levels of immune checkpoint‐related genes between the two risk groups. The histogram displayed that the expressions of multiple genes were higher in the high‐risk group (Figure 6E). Regretfully, there were no significant differences in the expression of PD‐1, PD‐L1, and CTLA4. However, the IPS analysis of TCGA samples (Table S7) showed that the IPS of anti‐PD‐1 and anti‐CTLA4 drugs in the low‐risk group was higher than that in the high‐risk group (Figure 6F–I), implicating more sensitive to anti‐PD‐1 and anti‐CTLA4 drugs of the low‐risk group. Last, we want to explore whether the risk score can predict the sensitivities of anti‐tumor chemotherapy drugs with “pRRophetic” package. The results showed that many anti‐tumor drugs presented lower IC50 in the high‐risk group, such as ABT.263, AP.24535, AS601245, and AZD6482 (Figure 6J–M), while multiple anti‐tumor drugs exhibited lower IC50 in the low‐risk group, such as paclitaxel, parthenolide, PD.0325901, and A.443654 (Figure 6N–Q), implying that risk score can provide reference for clinical therapy.

### 3.7 miR‐653 expression and its correlations with clinicopathological features of colorectal cancer

**FIGURE 7:**
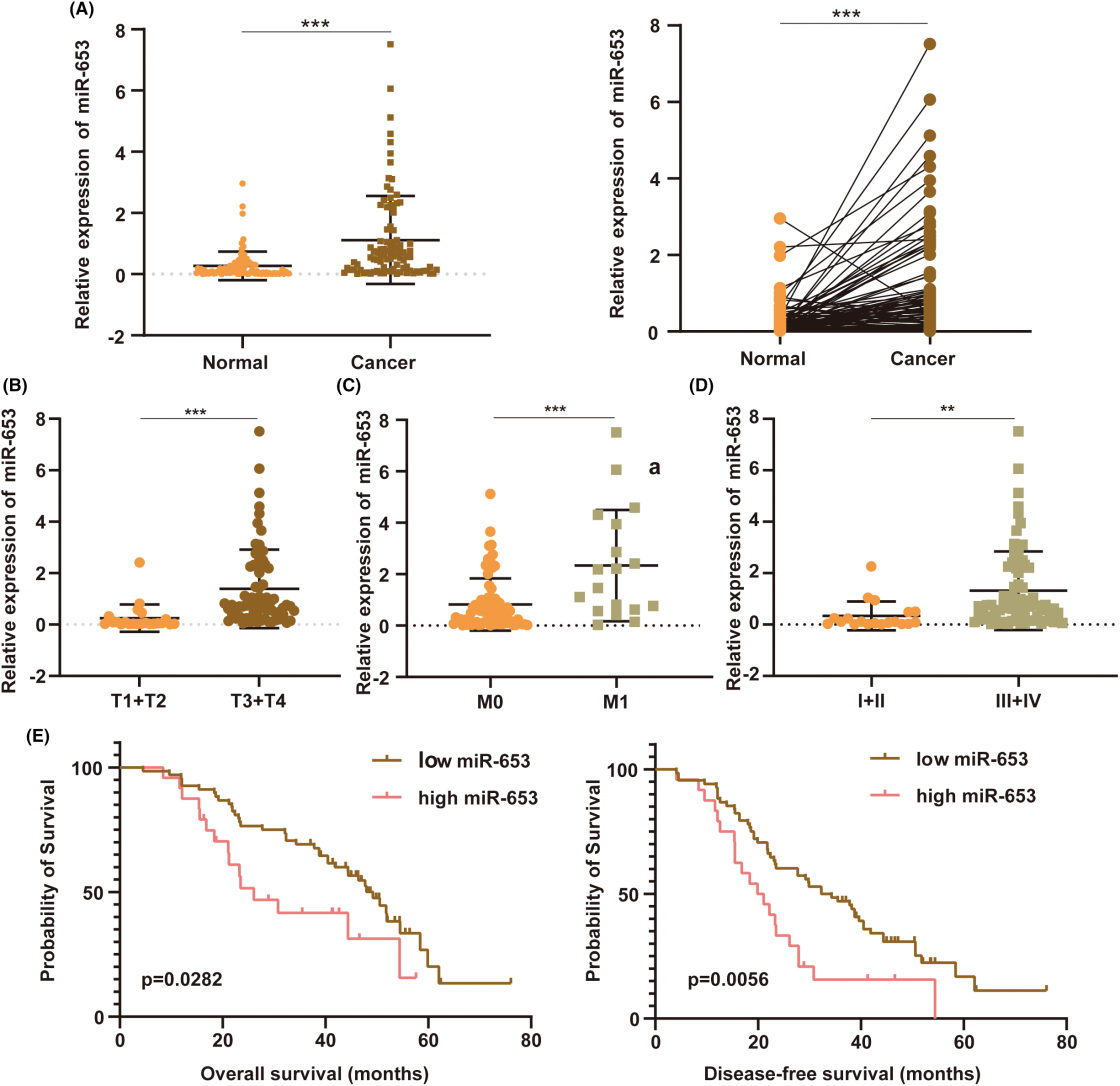
miR‐653 expression and its correlations with clinicopathological features of colorectal cancer. (A) The expression of miR‐653 in 92 paired colorectal cancer tissues and adjacent normal tissues. (B) The expression of miR‐653 was higher in the samples with T3 and T4 than in the samples with T2 and T1. (C) The expression of miR‐653 was higher in the samples with M1 than in the samples with M0. (D) The expression of miR‐653 was higher in the samples with Stage III and IV than in the samples with Stage I and III. (E) Kaplan–Meier survival analysis (log‐rank test) showed that patients with high miR‐653 expression had a lower overall survival and disease‐free survival than those with low miR‐653 expression. **p < 0.01; ***p < 0.001.

To further validate the model in clinical samples, we selected the differentially expressed miRNA (has‐miR‐653) with the highest expression from the 6 model miRNAs. First, we detected the expression of miR‐653 in 92 paired fresh frozen colorectal cancer tissues and adjacent normal tissues. The results showed that miR‐653 was significantly highly expressed in colorectal cancer tissues compared with adjacent normal tissues (Figure 7A). Then, we analyzed the correlations of the expression of miR‐653 with clinicopathological features of colorectal cancer. The expression of miR‐653 was significantly higher in the samples with T3 and T4 than in the samples with T2 and T1 (Figure 7B), in the samples with M1 than in the samples with M0 (Figure 7C), in the samples with Stage III and IV than in the samples with Stage I and II (Figure 7D). Next, we explored the correlations of the expression of miR‐653 with the survival of colorectal cancer. The results indicated that patients with high miR‐653 expression showed a shorter overall survival and disease‐free survival (Figure 7E). Taken together, miR‐653 is highly expressed in colorectal cancer tissues and closely correlated with the clinicopathological features of colorectal cancer.

### 3.8 miR‐653 promotes cell proliferation and inhibits apoptosis of colorectal cancer

**FIGURE 8:**
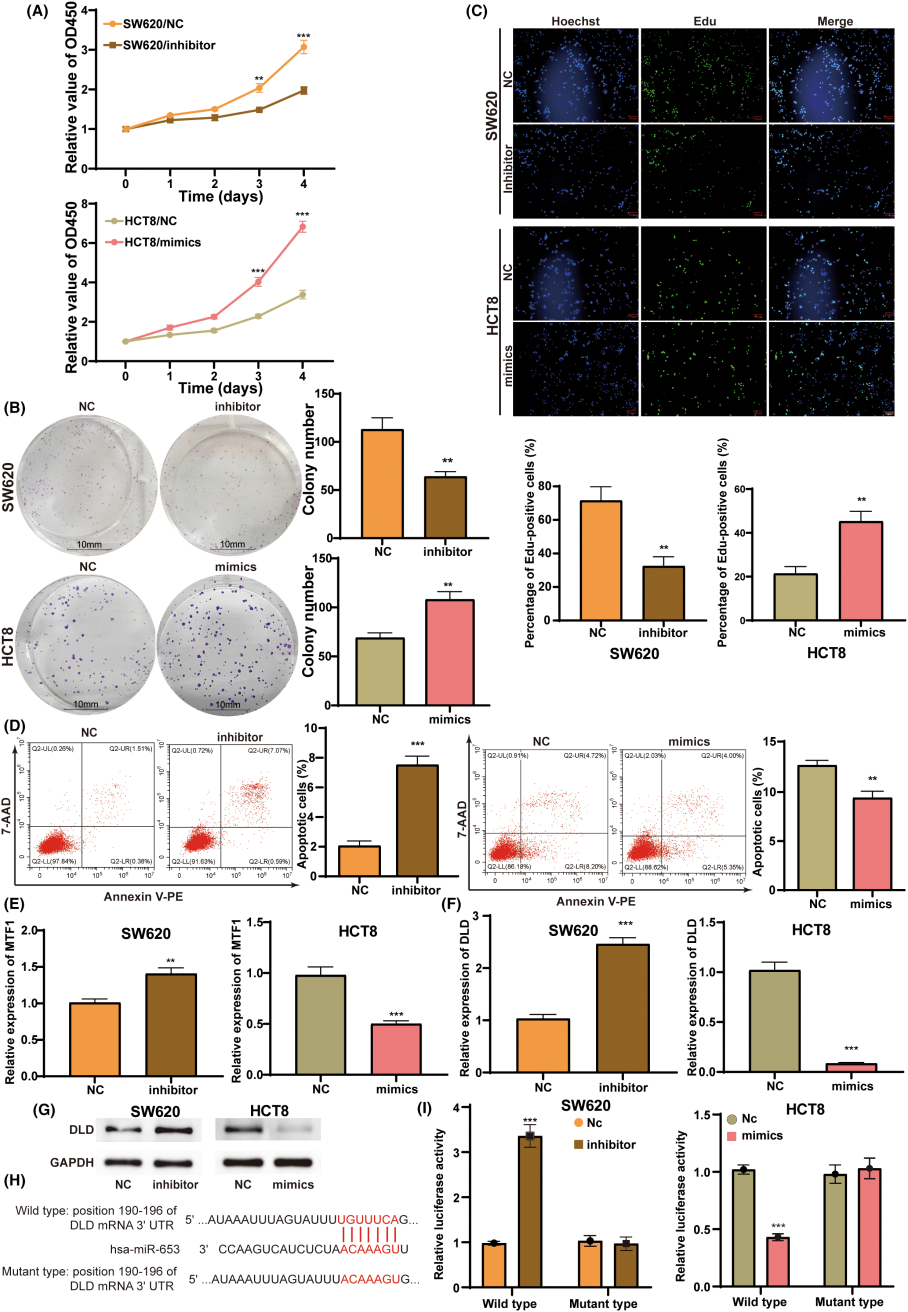
miR‐653 promotes cell proliferation and inhibits apoptosis of colorectal cancer by negatively regulating the expression of DLD. (A) The results of CCK8 assay after transfection of inhibitor or mimics. (B) The results of cell colony assay after transfection of inhibitor or mimics. (C) The results of edu assay after transfection of inhibitor or mimics. (D) The results of flow cytometry after transfection of inhibitor or mimics. (E) The qPCR results of MTF1 expression after transfection of inhibitor or mimics. (F) The qPCR results of DLD expression after transfection of inhibitor or mimics. (G) The western blotting results of DLD expression after transfection of inhibitor or mimics. (H) The wild‐type and mutant‐type binding site of miR‐653 on the 3′‐UTR of DLD mRNA. (I) The luciferase reporter assay of miR‐653 on the 3′‐UTR of DLD mRNA. **p < 0.01; ***p < 0.001.

To explore the effects of miR‐653 in colorectal cancer cells, the expression of miR‐653 in multiple colorectal cancer cells was detected. We found that the expression of miR‐653 was high in 8 colorectal cancer cells compared with normal colorectal mucosa cell (Figure S5A). SW620 cell showed the highest expression of miR‐653, and HCT8 cell showed the lowest expression of miR‐653. Thereby, we selected the two cell lines for further investigation. To alter the expression of miR‐653, inhibitors and mimics of miR‐653 were constructed. The qPCR results confirmed the efficiencies of knockdown and overexpression (Figure S3B,C). To test the effects of miR‐653 on cell proliferation, CCK8, cell colony, and edu assays were conducted. These assays all demonstrated that the knockdown of miR‐653 suppressed the cell proliferation, while the overexpression of miR‐653 promoted the cell proliferation (Figure 8A–C). In addition, the flow cytometry was performed to detect cell apoptosis. Knockdown of miR‐653 increased the percentage of apoptotic cells, while overexpression of miR‐653 decreased its percentage (Figure 8D).

According to above prediction from Targetscan database, MTF1 and DLD of key cuproptosis genes might be the target of miR‐653. To further explore the mechanism of miR‐653 on cuproptosis, we detected the expression of MTF1 and DLD under the alteration of miR‐653. qPCR results showed that miR‐653 inhibitor increased the expression of both MTF1 and DLD, while miR‐653 mimics decreased the expression of these two genes, of which DLD expression exhibited a bigger change (Figure 8E,F). Therefore, we investigated the regulation of miR‐653 on the expression of DLD in the following study. Western blotting showed the same results on the effects of miR‐653 on the expression of DLD protein (Figure 8G). Taking the consideration of the classical regulatory mechanism of miRNAs, we predicted that miR‐653 might bind the position 190–196 of DLD mRNA 3′‐UTR (Figure 8H). Next, we constructed the wild‐type luciferase reporter plasmid containing the wild DLD mRNA 3′‐UTR and mutant‐type luciferase reporter plasmid containing the mutant binding site on the DLD mRNA 3′‐UTR (Figure 8H). Luciferase reporter assay indicated that miR‐653 inhibitor enhanced the luciferase activity of wild‐type plasmid, while had no effect on the mutant‐type plasmid (Figure 8I). miR‐653 mimics exerted the similar effects (Figure 8I). Taken together, miR‐653 negatively regulates the expression of DLD, therefore promoting cell proliferation and inhibiting apoptosis of colorectal cancer.

## 4 DISCUSSION

A single miRNA can regulate several genes' expression and subsequent signaling pathways involved in tumorigenesis and tumor progression.^38^, ^39^ In particular, several miRNA‐related prognostic models have been demonstrated to play important roles in predicting the prognosis of patients with multiple tumor types.^40^, ^41^ Recently, the rise of cuproptosis further proved the important role of copper in cell function. Thus, exploring the impact of cuproptosis regulators on CRC patients' molecular patterns and prognostic values is imperative. In this study, a signature based on 6 cuproptosis‐related miRNAs was established. According to our prognostic model, the high‐risk group showed a poorer prognosis. Then, we verified that the risk score based on this prediction model was an independent prognostic factor for CRC patients. The prediction efficiency of this signature was evaluated by ROC and C‐index. This miRNA signature model was also confirmed in the test set and all samples set. In addition, combined with our newly developed prognosis model and CRC samples' clinicopathological factors, a predictive nomogram was constructed, which is expected to improve the accuracy of prognosis prediction.

With the deep research of multiple programmed cell death, such as autophagy, pyroptosis, and ferroptosis, we have got a better understanding of cancer development, prediction assessment, and clinical therapy. As a novel form of cell death, cuproptosis introduced a new insight into tumor cognition. Although cuproptosis has been rarely reported in cancer development and progression up to now, the dynamic balance of intracellular copper concentration has been demonstrated to influence multiple biological processes and human diseases, such as cell proliferation, autophagy, Wilson's disease, and Menke's disease.^13^, ^42^, ^43^, ^44^, ^45^ Therefore, regulating the transmembrane transport of copper ion is expected to be an ideal therapy for Wilson's disease and Menke's disease. In addition, some reports revealed that the levels of copper in the serum and tissues were higher in several cancer types than in normal people.^46^, ^47^ Nevertheless, the correlations of cuproptosis with tumor development and clinical therapy are urgent to be dissected. In this research, we identified 6 cuproptosis‐related miRNAs (hsa‐miR‐653, hsa‐miR‐216a, hsa‐miR‐3684, hsa‐miR‐4437, hsa‐miR‐641 and hsa‐miR‐552), and constructed a prognosis model for CRC. The risk score could act as an independent prognostic indicator (*p* < 0.001, 95% HR = 1.243 (1.129–1.369)) and the nomogram could efficiently predict the overall survival rate (AUC = 0.836). According to the previous studies, pyroptosis and ferroptosis signatures were significantly correlated with the type of tumor microenvironment and response to immunotherapy and chemotherapy.^48^, ^49^ Thereby, we next analyzed the immune infiltration type and treatment response in high‐ and low‐risk groups.

Previously, the tumors were divided into “hot” and “cold” tumors based on the immunoscore, which was estimated by the location, type, and density of immune cells.^50^, ^51^ Now, a more comprehensive classification was proposed as “hot,” “altered‐excluded,” “altered‐immunosuppressed,” and “cold” tumors, considering that apart from the T‐cell infiltration, other parameters such as the expression of CTLA4, PD‐1 or PD‐L1, TME status, and possible genomic mutations are both prognostic and predictive for patients.^32^, ^52^ The TME, composed of diverse chemokines, growth factors, extracellular matrix, and immune cells, significantly influences tumor progression and invasion.^53^, ^54^ Variable immune infiltrates and TME homeostasis led to different immunotherapy responses among different subtypes.^55^ In our prognostic signature, GSEA analysis showed that several immunosuppressive signaling pathways were enriched in the high‐risk group. The ssGSEA analysis also exhibited that immunosuppressive cells, such as macrophages, TILs, and Treg cells were highly infiltrated in the high‐risk group. These results demonstrated this cuproptosis‐related miRNA prognosis model will be useful as a guide for individual immunotherapy.

The ICI targets PD1 (nivolumab), PDL1 (atezolizumab), and CTLA4 (ipilimumab) were approved by FDA for CRC.^4^ Tumor mutational burden led to more peptide neoantigens encoded by the mutated genes, which were recognized as nonself by the immune system, in turn activating T cells for cytotoxic killing.^56^ Interestingly, several clinical trials demonstrated that patients with high TMB benefit more from the treatment of nivolumab plus ipilimumab regardless of the PD‐L1 expression level.^57^ Although no obvious differences were observed about TMB in different subgroups of our prognostic model, the significantly longer overall survival was illustrated in high TMB group. In addition, the immune cell proportion score analysis also indicated anti‐PD‐1 and anti‐CTLA4 were more sensitive in the low‐risk group. Therefore, this model can predict the sensitivity of ICI targets PD1 and CTLA4. In addition, IC50 of multiple anti‐tumor drugs presented with significant differences in the high‐ and low‐risk groups, further implying the important roles of risk score in predicting anti‐tumor efficiencies.

Many studies proved the effectiveness of miRNA‐based signatures in the prognosis and treatment of numerous tumors.^40^, ^58^ Our exploration also discovered that cuproptosis‐related miRNAs prognostic model was close to patients' survival and immune infiltration. Hsa‐miR‐216a, hsa‐miR‐3684, hsa‐miR‐4437, hsa‐miR‐641, and hsa‐miR‐552 have been reported to play suppressive or promotive roles in cancer. Hsa‐miR‐653 has been revealed to inhibit cancer progression in hepatocellular carcinoma, prostate cancer, breast cancer, papillary thyroid carcinoma, ovarian cancer, and gastric cancer,^59^, ^60^, ^61^, ^62^, ^63^, ^64^ while Liu et al. reported that miR‐653 enhances CRC progression by targeting circSETD3/KLF6 axis recently.^65^ Why does miR‐653 play an opposite role in CRC? We conducted a series of experiments to validate the roles of miR‐653 in CRC in this study. First, we found that miR‐653 was significantly highly expressed in 92 paired fresh frozen CRC tissues compared with adjacent normal tissues. Second, the expression of miR‐653 was closely associated with T stage, M stage, and tumor stage. Third, high miR‐653 expression predicted a shorter overall survival and disease‐free survival for CRC patients. Finally, in vitro experiments demonstrated that miR‐653 promotes cell proliferation and inhibits apoptosis of CRC. In terms of the regulatory mechanism, miR‐653 negatively regulated the expression of DLD by directly binding to the 3’‐UTR of DLD mRNA. Therefore, our experiments confirmed the high expression of miR‐653, its close correlations with clinical‐pathological features, and its key biological functions in CRC. Of course, abundant of clinical data on CRC should be collected to further evaluate the significance and role of this model by designing prospective studies.

## 5 CONCLUSIONS

In summary, a cuproptosis‐related miRNAs model was established to properly predict the prognosis. Targeting this signature, we can distinguish the immune types of CRC, contributing to individual therapy and improving the sensitivity of immunotherapy. In addition, miR‐653 was highly expressed in colorectal cancer tissues, closely correlated with the clinicopathological features, negatively regulated the expression of DLD, promoted cell proliferation, and inhibited apoptosis of colorectal cancer.

## AUTHOR CONTRIBUTIONS

**Zhonglin Zhu:** Conceptualization (equal); data curation (equal); methodology (equal); writing – original draft (equal). **Tianan Guo:** Formal analysis (equal); investigation (equal); visualization (equal). **Junyong Weng:** Funding acquisition (equal); methodology (equal); software (equal). **Shanbao Li:** Investigation (equal); methodology (equal); resources (equal). **Congcong Zhu:** Data curation (equal); investigation (equal). **Qiuyan Zhao:** Resources (equal); software (equal). **Ye Xu:** Conceptualization (equal); project administration (equal); supervision (equal); validation (equal); writing – review and editing (equal).

## FUNDING INFORMATION

This study was supported by the Science and Technology Commission of Shanghai Municipality (20DZ1100101), Shanghai Hospital Development Center (SKXZ2028), and National Natural Science Foundation of China (82003060).

## CONFLICT OF INTEREST STATEMENT

The authors have declared that no competing interests exist.

## ETHICS STATEMENT

Not applicable.

## Supporting information


Figure S1‐S5

Table S1

Table S2

Table S3

Table S4

Table S5

Table S6

Table S7

## Data Availability

The data used for the study are available online at: https://portal.gdc.cancer.gov/.
