# Supplementary material for: Cuproptosis‐related miRNAs signature and immune infiltration characteristics in colorectal cancer
Source: Cancer Med. 2023 Jun 19;12(15):16661–78. doi: 10.1002/cam4.6270 (PMC10469834; doi:10.1002/cam4.6270)
Supplement: Supplementary file 1 — Figure S1‐S5 [file CAM4-12-16661-s008.docx]

**Supplementary Materials:**

**Figure S1**

**
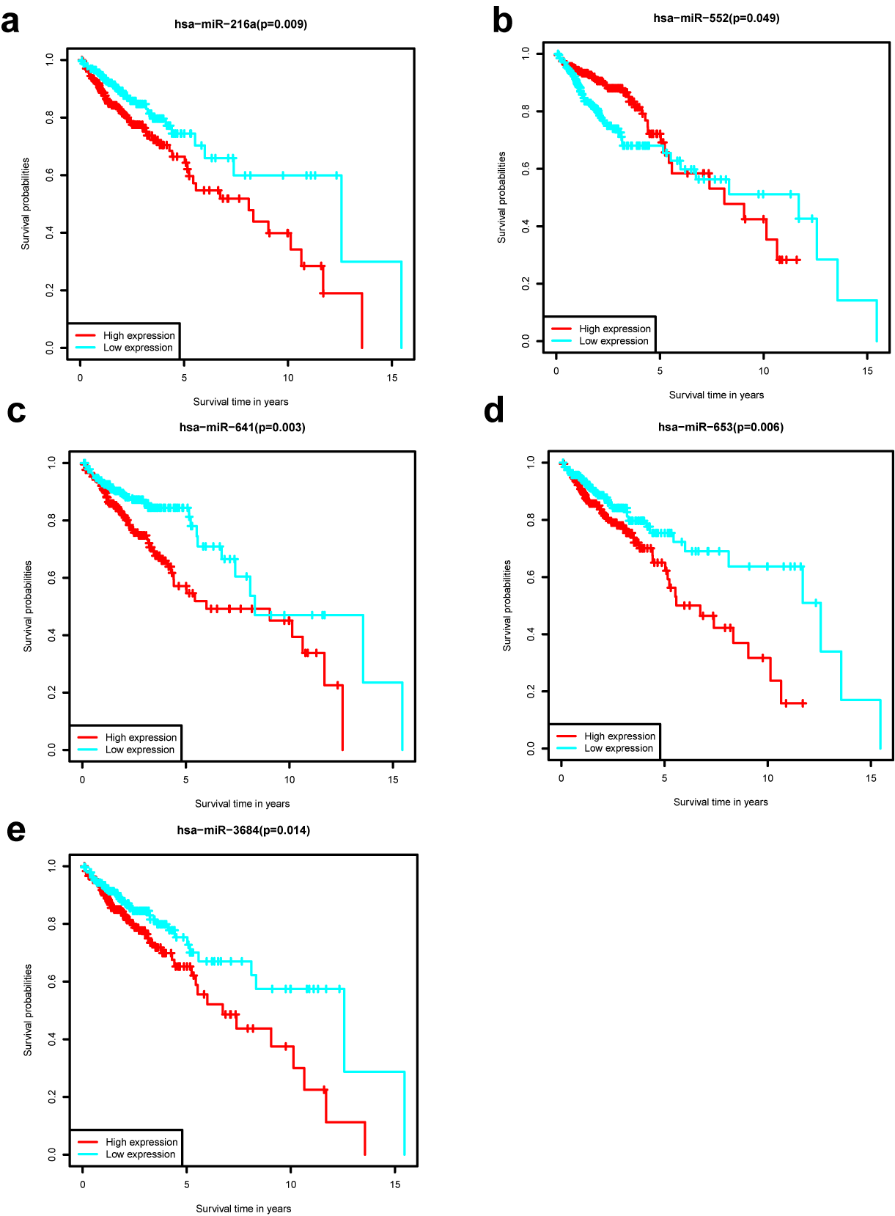
**

FIGURE S1 The Kaplan-Meier curves of model miRNAs in TCGA data. The Kaplan-Meier curves of hsa-miR-216a (A), hsa-miR-552 (B), hsa-miR-641 (C), hsa-miR-653 (D) and hsa-miR-3684 (E) in TCGA samples.

**Figure S2**


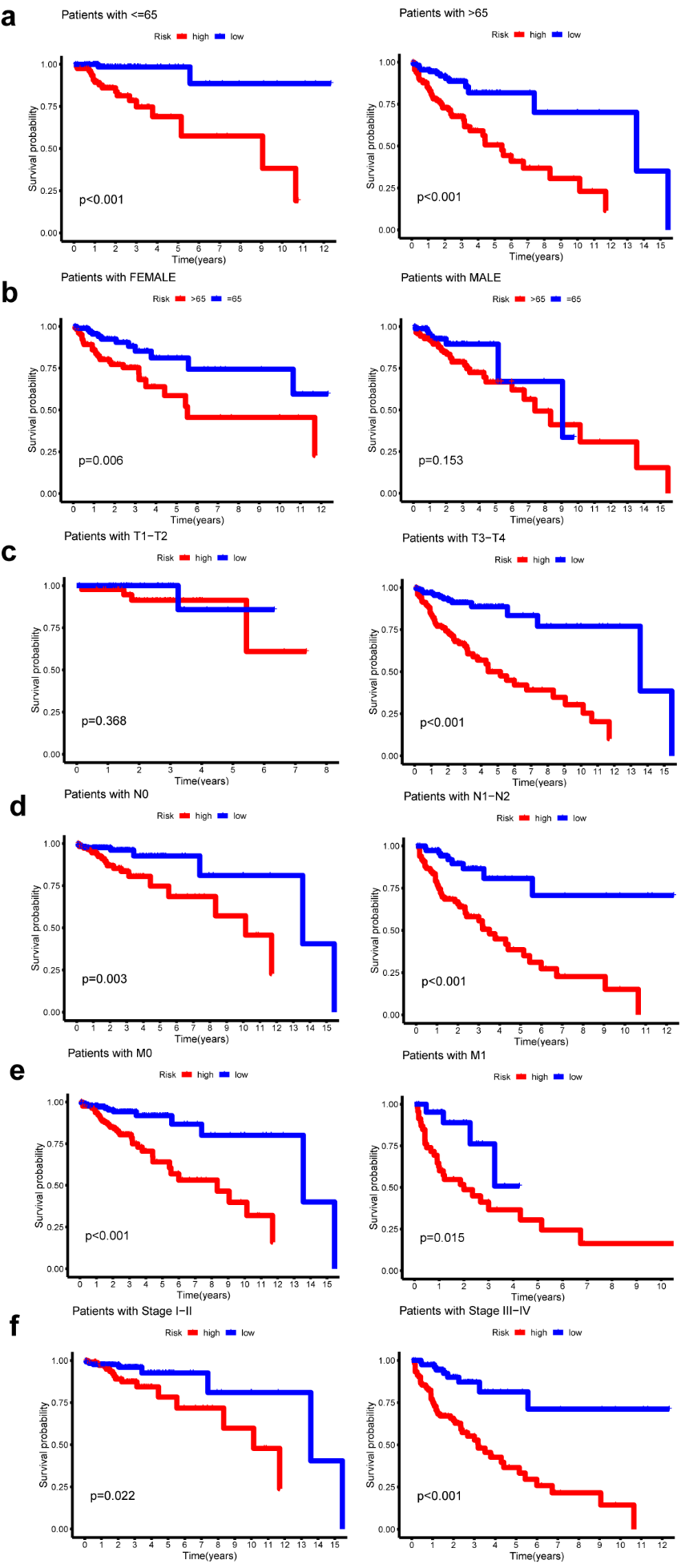


FIGURE S2 The Kaplan-Meier curves of overall survival in the high and low risk group with different clinicopathological features. The Kaplan-Meier curves of high and low risk groups in TCGA samples with <=65 and >65 years old (A), female and male (B), T1-T2 stages and T3-T4 stages (C), N0 stages and N1-N2 stages (D), M0 stages and M1 stages (E), TNM I-II stages and TNM III-IV stages (F), respectively.

**Figure S3**

**
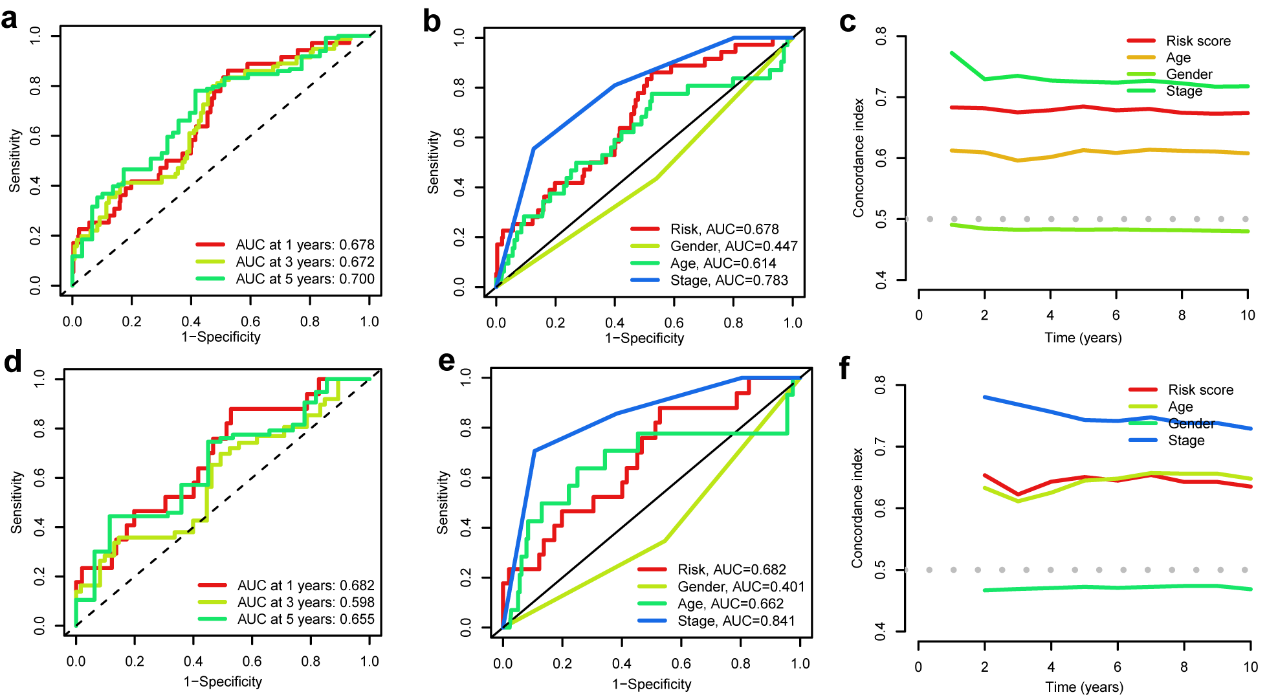
**

FIGURE S3 Verification of cuproptosis-related prognosis model with the test sets and all samples. (A) The 1-, 3-, and 5-year ROC of risk score in the test sets. (B) The 1-year ROC of risk score, gender, age and stage in the test sets. (C) The C-index of risk score, age, gender and stage in the test sets. (D) The 1-, 3-, and 5-year ROC of risk score in all samples. (E) The 1-year ROC of risk score, gender, age and stage in all samples. (F) The C-index of risk score, age, gender and stage in all samples.

**Figure S4**

**
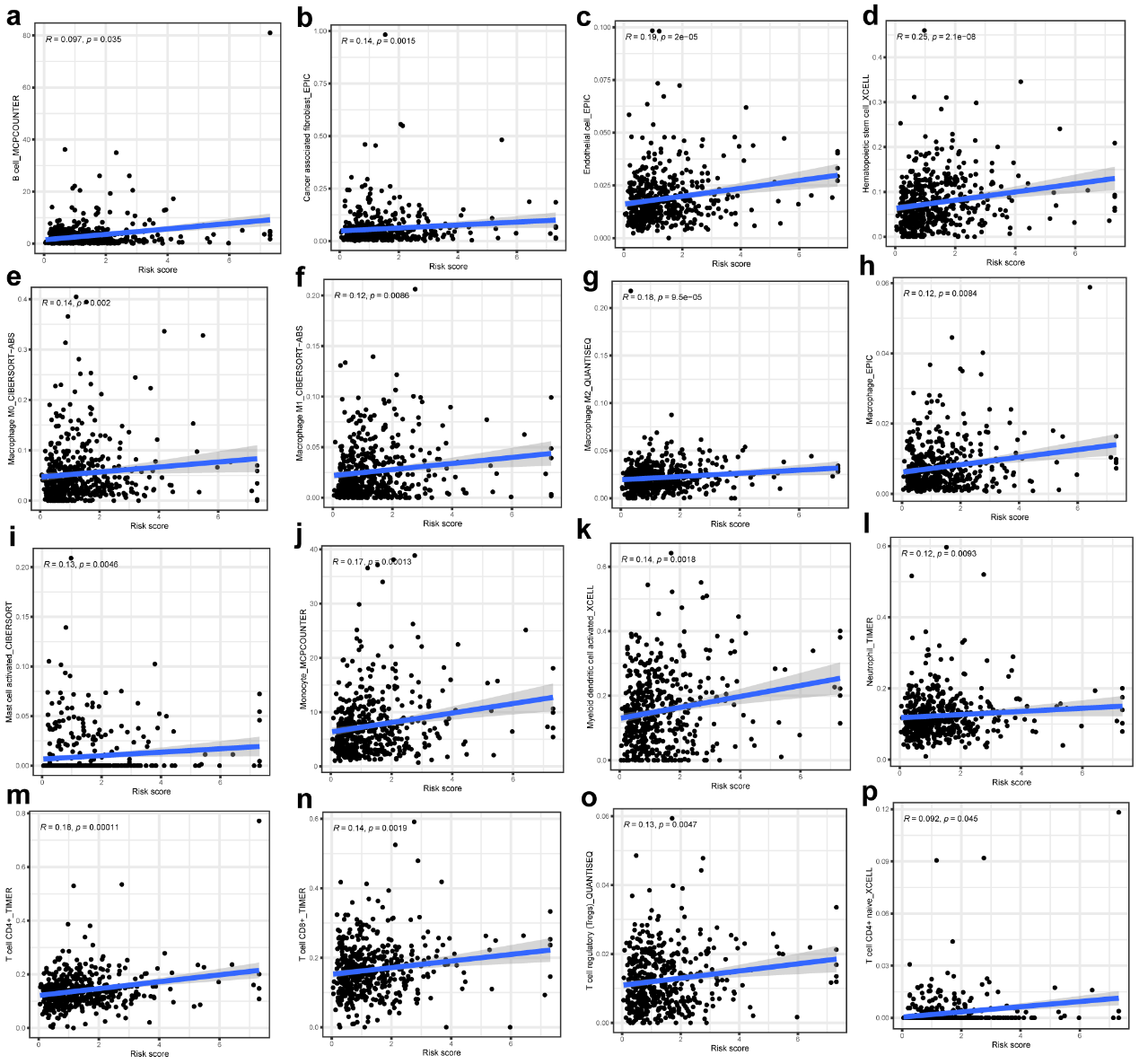
**

FIGURE S4 The correlations between risk score and the enrichment level of immune cells. Pearson correlation analysis between risk score with the levels of immune cells by multiple algorithms (A-P).

**Figure S5**


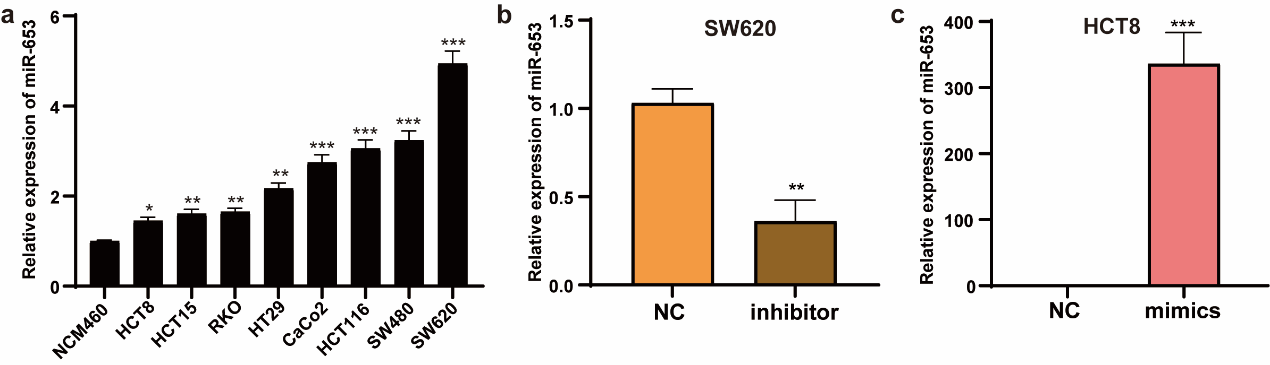


FIGURE S5 MiR-653 expression in different colorectal cancer cells. (A) The expression of miR-653 in colorectal cancer cells. (B-C) The relative expression of miR-653 after transfection of inhibitor or mimics. *P < 0.05;**P < 0.01; ***P < 0.001.
