# Supplementary material for: Cuproptosis‐related miRNAs signature and immune infiltration characteristics in colorectal cancer
Source: Cancer Med. 2023 Jun 19;12(15):16661–78. doi: 10.1002/cam4.6270 (PMC10469834; doi:10.1002/cam4.6270)
Supplement: Supplementary file 2 — Table S1 [file CAM4-12-16661-s004.docx]

TABLE S1. The miRNAs were predicted to negatively regulate the expression of these 16 cuproptosis regulators with Targetscan database.

| gene | miRNA |
| --- | --- |
| ATP7A | hsa-miR-6934-5p |
| ATP7A | hsa-miR-1283a |
| ATP7A | hsa-miR-2388-5p |
| ATP7A | hsa-miR-487-5p |
| ATP7A | hsa-miR-3085-3p |
| ATP7A | hsa-miR-6971-3p |
| ATP7A | hsa-miR-548ag |
| ATP7A | hsa-miR-511-3p |
| ATP7A | hsa-miR-26a-3p |
| ATP7A | hsa-miR-486-5p |
| ATP7A | hsa-miR-4510 |
| ATP7A | hsa-miR-188-5p |
| ATP7A | hsa-miR-7239-5p |
| ATP7A | hsa-miR-3909 |
| ATP7A | hsa-miR-3101-3p |
| ATP7A | hsa-miR-2285u |
| ATP7A | hsa-miR-8892 |
| ATP7A | hsa-miR-1912 |
| ATP7A | hsa-miR-543-5p |
| ATP7A | hsa-miR-7667-5p |
| ATP7A | hsa-miR-374b-5p |
| ATP7A | hsa-miR-627-5p |
| ATP7A | hsa-miR-1638 |
| ATP7A | hsa-miR-6862-3p |
| ATP7A | hsa-miR-7207-5p |
| ATP7A | hsa-miR-376c |
| ATP7A | hsa-miR-5116 |
| ATP7A | hsa-miR-3133 |
| ATP7A | hsa-miR-302c-3p |
| ATP7A | hsa-miR-2136 |
| ATP7A | hsa-miR-879-3p |
| ATP7A | hsa-miR-344f-3p |
| ATP7A | hsa-miR-6915-3p |
| ATP7A | hsa-miR-4640-3p |
| ATP7A | hsa-miR-552-5p |
| ATP7A | hsa-miR-676-3p |
| ATP7A | hsa-miR-4697-3p |
| ATP7A | hsa-miR-411-5p |
| ATP7A | hsa-miR-7260-3p |
| ATP7A | hsa-miR-548ao-3p |
| ATP7A | hsa-miR-939-3p |
| ATP7A | hsa-miR-292-5p |
| ATP7A | hsa-miR-350 |
| ATP7A | hsa-miR-4709-5p |
| ATP7A | hsa-miR-6086 |
| ATP7A | hsa-miR-2284n |
| ATP7A | hsa-miR-2488 |
| ATP7A | hsa-miR-4665-5p |
| ATP7A | hsa-miR-601 |
| ATP7A | hsa-miR-6984-5p |
| ATP7A | hsa-miR-302a-5p |
| ATP7A | hsa-miR-7321-3p |
| ATP7A | hsa-miR-298-3p |
| ATP7A | hsa-miR-5110 |
| ATP7A | hsa-miR-6881-3p |
| ATP7A | hsa-miR-2298 |
| ATP7A | hsa-miR-7398r-5p |
| ATP7A | hsa-miR-7398d-3p |
| ATP7A | hsa-miR-3619-5p |
| ATP7A | hsa-miR-548j-5p |
| ATP7A | hsa-miR-5189-3p |
| ATP7A | hsa-miR-7163-5p |
| ATP7A | hsa-miR-6755-3p |
| ATP7A | hsa-miR-877-5p |
| ATP7A | hsa-miR-3689-3p |
| ATP7A | hsa-miR-7974 |
| ATP7A | hsa-miR-3576 |
| ATP7A | hsa-miR-7086-5p |
| ATP7A | hsa-miR-429 |
| ATP7A | hsa-miR-652-5p |
| ATP7A | hsa-miR-6883-5p |
| ATP7A | hsa-miR-193-5p |
| ATP7A | hsa-miR-2432 |
| ATP7A | hsa-miR-101-1-5p |
| ATP7A | hsa-miR-890-5p |
| ATP7A | hsa-miR-548u |
| ATP7A | hsa-miR-3123 |
| ATP7A | hsa-miR-885 |
| ATP7A | hsa-miR-377-5p |
| ATP7A | hsa-miR-96-5p |
| ATP7A | hsa-miR-379-5p |
| ATP7A | hsa-miR-3595 |
| ATP7A | hsa-miR-496.2 |
| ATP7A | hsa-miR-7850-5p |
| ATP7A | hsa-miR-425-5p |
| ATP7A | hsa-miR-2411-3p |
| ATP7A | hsa-miR-302b-5p |
| ATP7A | hsa-miR-499b-5p |
| ATP7A | hsa-miR-3606-5p |
| ATP7A | hsa-miR-320-5p |
| ATP7A | hsa-miR-935 |
| ATP7A | hsa-miR-6990-3p |
| ATP7A | hsa-miR-5096 |
| ATP7A | hsa-miR-3120-5p |
| ATP7A | hsa-miR-5692a |
| ATP7A | hsa-miR-3126-3p |
| ATP7A | hsa-miR-1273h-5p |
| ATP7A | hsa-miR-6319 |
| ATP7A | hsa-miR-6753-5p |
| ATP7A | hsa-miR-624-5p |
| ATP7A | hsa-miR-8062 |
| ATP7A | hsa-miR-6817-3p |
| ATP7A | hsa-miR-3087-3p |
| ATP7A | hsa-miR-3067-5p |
| ATP7A | hsa-miR-134-3p |
| ATP7A | hsa-miR-7351-5p |
| ATP7A | hsa-miR-7664-5p |
| ATP7A | hsa-miR-2472 |
| ATP7A | hsa-miR-517 |
| ATP7A | hsa-miR-548bb-3p |
| ATP7A | hsa-miR-551-5p |
| ATP7A | hsa-miR-765 |
| ATP7A | hsa-miR-6481 |
| ATP7A | hsa-miR-3571 |
| ATP7A | hsa-miR-214-5p |
| ATP7A | hsa-miR-1273g-3p |
| ATP7A | hsa-miR-183-5p |
| ATP7A | hsa-miR-411-3p |
| ATP7A | hsa-miR-15 |
| ATP7A | hsa-miR-2891 |
| ATP7A | hsa-miR-466bco-5p |
| ATP7A | hsa-miR-539-3p |
| ATP7A | hsa-miR-7110-3p |
| ATP7A | hsa-miR-460b-5p |
| ATP7A | hsa-miR-3928-5p |
| ATP7A | hsa-miR-7-3p |
| ATP7A | hsa-miR-8107 |
| ATP7A | hsa-miR-2337 |
| ATP7A | hsa-miR-30e-3p |
| ATP7A | hsa-miR-7386g-3p |
| ATP7A | hsa-miR-1587 |
| ATP7A | hsa-miR-7331-2-3p |
| ATP7A | hsa-miR-7231-5p |
| ATP7A | hsa-miR-1249-5p |
| ATP7A | hsa-miR-409 |
| ATP7A | hsa-miR-2441 |
| ATP7A | hsa-miR-4311 |
| ATP7A | hsa-miR-6766-5p |
| ATP7A | hsa-miR-6133 |
| ATP7A | hsa-miR-351-5p |
| ATP7A | hsa-miR-7856-5p |
| ATP7A | hsa-miR-7291-3p |
| ATP7A | hsa-miR-4698 |
| ATP7A | hsa-miR-883a-5p |
| ATP7A | hsa-miR-338-5p |
| ATP7A | hsa-miR-1251-3p |
| ATP7A | hsa-miR-6513-5p |
| ATP7A | hsa-miR-7977 |
| ATP7A | hsa-miR-216b |
| ATP7A | hsa-miR-7032-3p |
| ATP7A | hsa-miR-374-3p |
| ATP7A | hsa-miR-3976 |
| ATP7A | hsa-miR-133a-5p |
| ATP7A | hsa-miR-548w |
| ATP7A | hsa-miR-7159-3p |
| ATP7A | hsa-miR-8084 |
| ATP7A | hsa-miR-6639-5p |
| ATP7A | hsa-miR-1958 |
| ATP7A | hsa-miR-22-5p |
| ATP7A | hsa-miR-4474-3p |
| ATP7A | hsa-miR-199-5p |
| ATP7A | hsa-miR-656-3p |
| ATP7A | hsa-miR-6555-5p |
| ATP7A | hsa-miR-126-3p |
| ATP7A | hsa-let-7a-3p |
| ATP7A | hsa-miR-548e-5p |
| ATP7A | hsa-miR-7219-5p |
| ATP7A | hsa-miR-6499-3p |
| ATP7A | hsa-miR-7680-5p |
| ATP7A | hsa-miR-1199-5p |
| ATP7A | hsa-miR-3154 |
| ATP7A | hsa-miR-548g-5p |
| ATP7A | hsa-miR-548t-5p |
| ATP7A | hsa-miR-3544-3p |
| ATP7A | hsa-miR-7340-5p |
| ATP7A | hsa-miR-761 |
| ATP7A | hsa-miR-6925-5p |
| ATP7A | hsa-miR-2370-3p |
| ATP7A | hsa-miR-293-5p |
| ATP7A | hsa-miR-4422 |
| ATP7A | hsa-miR-1224 |
| ATP7A | hsa-miR-184 |
| ATP7A | hsa-miR-8895 |
| ATP7A | hsa-miR-6501-3p |
| ATP7A | hsa-miR-589-5p |
| ATP7A | hsa-miR-548i-5p |
| ATP7A | hsa-miR-561-3p |
| ATP7A | hsa-miR-7650-5p |
| ATP7A | hsa-miR-6779-5p |
| ATP7A | hsa-miR-2284aa |
| ATP7A | hsa-miR-7037-5p |
| ATP7A | hsa-miR-367-5p |
| ATP7A | hsa-miR-1976 |
| ATP7A | hsa-miR-194-2-3p |
| ATP7A | hsa-miR-6981-5p |
| ATP7A | hsa-miR-21-5p |
| ATP7A | hsa-miR-548ap-5p |
| ATP7A | hsa-miR-107-5p |
| ATP7A | hsa-miR-449c-5p |
| ATP7A | hsa-miR-126b-5p |
| ATP7A | hsa-miR-3147 |
| ATP7A | hsa-miR-6860 |
| ATP7A | hsa-miR-2284a |
| ATP7A | hsa-miR-597-5p |
| ATP7A | hsa-miR-500b-3p |
| ATP7A | hsa-miR-5579-5p |
| ATP7A | hsa-miR-99a-3p |
| ATP7A | hsa-miR-548az-5p |
| ATP7A | hsa-miR-7385f-3p |
| ATP7A | hsa-miR-619 |
| ATP7A | hsa-miR-6916-3p |
| ATP7A | hsa-miR-219-1-3p |
| ATP7A | hsa-miR-548ab |
| ATP7A | hsa-miR-7373c-3p |
| ATP7A | hsa-miR-7299-5p |
| ATP7A | hsa-miR-6127 |
| ATP7A | hsa-miR-1180-3p |
| ATP7A | hsa-miR-106-3p |
| ATP7A | hsa-miR-7328-5p |
| ATP7A | hsa-miR-136-5p |
| ATP7A | hsa-miR-201-3p |
| ATP7A | hsa-miR-6720-5p |
| ATP7A | hsa-miR-548n |
| ATP7A | hsa-miR-1236-5p |
| ATP7A | hsa-miR-8902 |
| ATP7A | hsa-miR-6077 |
| ATP7A | hsa-miR-1297 |
| ATP7A | hsa-miR-706 |
| ATP7A | hsa-miR-3613b |
| ATP7A | hsa-miR-195-3p |
| ATP7A | hsa-miR-4765 |
| ATP7A | hsa-miR-570-3p |
| ATP7A | hsa-miR-2888 |
| ATP7A | hsa-miR-452-5p |
| ATP7A | hsa-miR-200 |
| ATP7A | hsa-miR-7171-3p |
| ATP7A | hsa-miR-7063-3p |
| ATP7A | hsa-miR-6868-5p |
| ATP7A | hsa-miR-6519 |
| ATP7A | hsa-miR-6334 |
| ATP7A | hsa-miR-880-5p |
| ATP7A | hsa-miR-7315-3p |
| ATP7A | hsa-miR-934 |
| ATP7A | hsa-miR-548aa |
| ATP7A | hsa-miR-141-3p |
| ATP7A | hsa-miR-6890-3p |
| ATP7A | hsa-miR-7246-3p |
| ATP7A | hsa-miR-101a-5p |
| ATP7A | hsa-miR-18 |
| ATP7A | hsa-miR-6864-5p |
| ATP7A | hsa-miR-3617-3p |
| ATP7A | hsa-miR-221-3p |
| ATP7A | hsa-miR-7225-3p |
| ATP7A | hsa-miR-702-5p |
| ATP7A | hsa-miR-96-5p |
| ATP7A | hsa-miR-877-3p |
| ATP7A | hsa-miR-2284u |
| ATP7A | hsa-miR-128 |
| ATP7A | hsa-miR-4446 |
| ATP7A | hsa-miR-548k |
| ATP7A | hsa-miR-8847 |
| ATP7A | hsa-miR-5004-3p |
| ATP7A | hsa-miR-2286 |
| ATP7A | hsa-miR-518d-5p |
| ATP7A | hsa-miR-101a-3p.2 |
| ATP7A | hsa-miR-579 |
| ATP7A | hsa-miR-7055-5p |
| ATP7A | hsa-miR-8094 |
| ATP7A | hsa-miR-216a-3p |
| ATP7A | hsa-miR-2484 |
| ATP7A | hsa-miR-24-5p |
| ATP7A | hsa-miR-154-5p |
| ATP7A | hsa-miR-487a-5p |
| ATP7A | hsa-miR-7330-3p |
| ATP7A | hsa-miR-8861 |
| ATP7A | hsa-miR-6980-3p |
| ATP7A | hsa-miR-4738-3p |
| ATP7A | hsa-miR-1545a-3p |
| ATP7A | hsa-miR-7372-5p |
| ATP7A | hsa-miR-6942-5p |
| ATP7A | hsa-miR-299-5p |
| ATP7A | hsa-miR-7162-3p |
| ATP7A | hsa-miR-190a-3p |
| ATP7A | hsa-miR-126-5p |
| ATP7A | hsa-miR-759 |
| ATP7A | hsa-miR-7106-3p |
| ATP7A | hsa-miR-743ab-3p |
| ATP7A | hsa-miR-6808-5p |
| ATP7A | hsa-miR-1258-5p |
| ATP7A | hsa-miR-1243 |
| ATP7A | hsa-miR-9-3p |
| ATP7A | hsa-miR-200a-5p |
| ATP7A | hsa-miR-4791 |
| ATP7A | hsa-miR-7395-3p |
| ATP7A | hsa-miR-877-5p |
| ATP7A | hsa-miR-7039-3p |
| ATP7A | hsa-miR-3958 |
| ATP7A | hsa-miR-7242-5p |
| ATP7A | hsa-miR-7398a-3p |
| ATP7A | hsa-miR-4640-5p |
| ATP7A | hsa-miR-451-3p |
| ATP7A | hsa-miR-2113 |
| ATP7A | hsa-miR-7685-3p |
| ATP7A | hsa-miR-1295b-5p |
| ATP7A | hsa-miR-8065 |
| ATP7A | hsa-miR-340-3p |
| ATP7A | hsa-miR-7073-5p |
| ATP7A | hsa-miR-629-5p |
| ATP7A | hsa-miR-7053-5p |
| ATP7A | hsa-miR-6760-3p |
| ATP7A | hsa-miR-7111-5p |
| ATP7A | hsa-miR-30ad-3p |
| ATP7A | hsa-miR-1416-5p |
| ATP7A | hsa-miR-8806 |
| ATP7A | hsa-miR-548bb-5p |
| ATP7A | hsa-miR-449-5p |
| ATP7A | hsa-miR-545-3p |
| ATP7A | hsa-miR-9500 |
| ATP7A | hsa-miR-8904b |
| ATP7A | hsa-miR-148-5p |
| ATP7A | hsa-miR-7196-5p |
| ATP7A | hsa-miR-3919 |
| ATP7A | hsa-miR-6992-3p |
| ATP7A | hsa-miR-4501 |
| ATP7A | hsa-miR-4661-5p |
| ATP7A | hsa-miR-4777-5p |
| ATP7A | hsa-let-7-3p |
| ATP7A | hsa-miR-298-5p |
| ATP7A | hsa-miR-186-5p |
| ATP7A | hsa-miR-1451-3p |
| ATP7A | hsa-miR-6959-5p |
| ATP7A | hsa-miR-888-5p |
| ATP7A | hsa-miR-5003-3p |
| ATP7A | hsa-miR-885-3p |
| ATP7A | hsa-miR-5093 |
| ATP7A | hsa-miR-7277-5p |
| ATP7A | hsa-miR-124 |
| ATP7A | hsa-miR-742-3p |
| ATP7A | hsa-miR-2285x |
| ATP7A | hsa-miR-4459 |
| ATP7A | hsa-miR-6828-3p |
| ATP7A | hsa-miR-6965-3p |
| ATP7A | hsa-miR-6715b-5p |
| ATP7A | hsa-miR-1-3p |
| ATP7A | hsa-miR-7578 |
| ATP7A | hsa-miR-2461-3p |
| ATP7A | hsa-miR-7168-3p |
| ATP7A | hsa-miR-133-5p |
| ATP7A | hsa-miR-7650-3p |
| ATP7A | hsa-miR-292a-3p |
| ATP7A | hsa-miR-3106-5p |
| ATP7A | hsa-miR-2426 |
| ATP7A | hsa-miR-606 |
| ATP7A | hsa-miR-7297-5p |
| ATP7A | hsa-miR-3613-3p |
| ATP7A | hsa-miR-3138 |
| ATP7A | hsa-miR-340-5p |
| ATP7A | hsa-miR-3584-5p |
| ATP7A | hsa-miR-3658 |
| ATP7A | hsa-miR-7652-3p |
| ATP7A | hsa-miR-3561-3p |
| ATP7A | hsa-miR-6878-5p |
| ATP7A | hsa-miR-154-5p |
| ATP7A | hsa-miR-3577-3p |
| ATP7A | hsa-miR-3714 |
| ATP7A | hsa-miR-32-3p |
| ATP7A | hsa-miR-1731-5p |
| ATP7A | hsa-miR-381-3p |
| ATP7A | hsa-miR-3612 |
| ATP7A | hsa-miR-485-5p |
| ATP7A | hsa-miR-454-5p |
| ATP7A | hsa-miR-23-3p |
| ATP7A | hsa-miR-3966 |
| ATP7A | hsa-miR-6875-3p |
| ATP7A | hsa-miR-216c-3p |
| ATP7A | hsa-miR-548d-5p |
| ATP7A | hsa-miR-8817 |
| ATP7A | hsa-miR-6798-3p |
| ATP7A | hsa-miR-2285z |
| ATP7A | hsa-miR-7352-5p |
| ATP7A | hsa-miR-7205-3p |
| ATP7A | hsa-miR-2480 |
| ATP7A | hsa-miR-6331 |
| ATP7A | hsa-miR-7703 |
| ATP7A | hsa-miR-7014-5p |
| ATP7A | hsa-miR-200ab-5p |
| ATP7A | hsa-miR-6987-3p |
| ATP7A | hsa-miR-30-3p |
| ATP7A | hsa-miR-28-5p |
| ATP7A | hsa-miR-3059-3p |
| ATP7A | hsa-miR-195-5p |
| ATP7A | hsa-miR-450a |
| ATP7A | hsa-miR-376c-5p |
| ATP7A | hsa-miR-1910-3p |
| ATP7A | hsa-miR-7161-5p |
| ATP7A | hsa-miR-6926-5p |
| ATP7A | hsa-miR-3927-5p |
| ATP7A | hsa-miR-194-1-3p |
| ATP7A | hsa-miR-5009-3p |
| ATP7A | hsa-miR-654-3p |
| ATP7A | hsa-miR-2385-5p |
| ATP7A | hsa-miR-509-3-5p |
| ATP7A | hsa-miR-6901-3p |
| ATP7A | hsa-miR-3613-5p |
| ATP7A | hsa-miR-7390-5p |
| ATP7A | hsa-miR-7478-5p |
| ATP7A | hsa-miR-6509-3p |
| ATP7A | hsa-miR-207 |
| ATP7A | hsa-miR-6774-5p |
| ATP7A | hsa-miR-2362 |
| ATP7A | hsa-miR-5190 |
| ATP7A | hsa-let-7f-2-3p |
| ATP7A | hsa-miR-5582-3p |
| ATP7A | hsa-miR-193b-5p |
| ATP7A | hsa-miR-4511 |
| ATP7A | hsa-miR-34b-3p |
| ATP7A | hsa-miR-2460 |
| ATP7A | hsa-miR-2296 |
| ATP7A | hsa-miR-517-5p |
| ATP7A | hsa-miR-421-5p |
| ATP7A | hsa-miR-8908c |
| ATP7A | hsa-miR-8890 |
| ATP7A | hsa-miR-4284 |
| ATP7A | hsa-miR-4503 |
| ATP7A | hsa-miR-612 |
| ATP7A | hsa-miR-34a-3p |
| ATP7A | hsa-miR-204-5p |
| ATP7A | hsa-miR-7174-5p |
| ATP7A | hsa-miR-1298 |
| ATP7A | hsa-miR-577 |
| ATP7A | hsa-miR-5703 |
| ATP7A | hsa-miR-1193-5p |
| ATP7A | hsa-miR-1288-3p |
| ATP7A | hsa-miR-6360 |
| ATP7A | hsa-miR-556-5p |
| ATP7A | hsa-miR-3921 |
| ATP7A | hsa-miR-3668 |
| ATP7A | hsa-miR-8550 |
| ATP7A | hsa-miR-3103-5p |
| ATP7A | hsa-miR-2421 |
| ATP7A | hsa-miR-4999-5p |
| ATP7A | hsa-miR-2486-3p |
| ATP7A | hsa-miR-148a-3p |
| ATP7A | hsa-miR-4660 |
| ATP7A | hsa-miR-7236-5p |
| ATP7A | hsa-miR-4312 |
| ATP7A | hsa-miR-219-5p |
| ATP7A | hsa-miR-7186-3p |
| ATP7A | hsa-miR-708-3p |
| ATP7A | hsa-miR-4490 |
| ATP7A | hsa-miR-6380 |
| ATP7A | hsa-miR-206-3p |
| ATP7A | hsa-miR-6520 |
| ATP7A | hsa-miR-146-5p |
| ATP7A | hsa-miR-291b-3p |
| ATP7A | hsa-miR-1183 |
| ATP7A | hsa-miR-1260a |
| ATP7A | hsa-miR-4441 |
| ATP7A | hsa-miR-762 |
| ATP7A | hsa-miR-205-5p |
| ATP7A | hsa-miR-579-3p |
| ATP7A | hsa-miR-7119-5p |
| ATP7A | hsa-miR-4668-3p |
| ATP7A | hsa-miR-4799-5p |
| ATP7A | hsa-miR-106a-3p |
| ATP7A | hsa-miR-7384-3p |
| ATP7A | hsa-miR-6857-3p |
| ATP7A | hsa-miR-344f-5p |
| ATP7A | hsa-miR-6827-3p |
| ATP7A | hsa-miR-4729 |
| ATP7A | hsa-miR-16c-3p |
| ATP7A | hsa-miR-6372 |
| ATP7A | hsa-miR-578 |
| ATP7A | hsa-miR-1258 |
| ATP7A | hsa-miR-5586-3p |
| ATP7A | hsa-miR-203-3p.2 |
| ATP7A | hsa-miR-298 |
| ATP7A | hsa-miR-3562 |
| ATP7A | hsa-miR-7360-5p |
| ATP7A | hsa-miR-511-5p |
| ATP7A | hsa-miR-2292 |
| ATP7A | hsa-miR-7385e-3p |
| ATP7A | hsa-miR-9-5p |
| ATP7A | hsa-miR-616-5p |
| ATP7A | hsa-miR-4483 |
| ATP7A | hsa-miR-2284y |
| ATP7A | hsa-miR-31-5p |
| ATP7A | hsa-miR-181b-2-3p |
| ATP7A | hsa-miR-7385a-3p |
| ATP7A | hsa-miR-6414 |
| ATP7A | hsa-miR-3551-5p |
| ATP7A | hsa-miR-205-5p |
| ATP7A | hsa-miR-30 |
| ATP7A | hsa-miR-494 |
| ATP7A | hsa-miR-3173-3p |
| ATP7A | hsa-miR-4703-3p |
| ATP7A | hsa-miR-1547-5p |
| ATP7A | hsa-miR-6843-3p |
| ATP7A | hsa-miR-650 |
| ATP7A | hsa-miR-741-3p |
| ATP7A | hsa-miR-6772-3p |
| ATP7A | hsa-miR-4684-5p |
| ATP7A | hsa-miR-504-3p |
| ATP7A | hsa-miR-3569 |
| ATP7A | hsa-miR-146 |
| ATP7A | hsa-miR-8075 |
| ATP7A | hsa-miR-2443 |
| ATP7A | hsa-miR-3620-5p |
| ATP7A | hsa-miR-709 |
| ATP7A | hsa-miR-875 |
| ATP7A | hsa-miR-8118 |
| ATP7A | hsa-miR-1749-3p |
| ATP7A | hsa-miR-764 |
| ATP7A | hsa-miR-503-3p |
| ATP7A | hsa-miR-204-5p |
| ATP7A | hsa-miR-3596a |
| ATP7A | hsa-miR-584-5p |
| ATP7A | hsa-miR-4482-3p |
| ATP7A | hsa-miR-8905 |
| ATP7A | hsa-miR-2370-5p |
| ATP7A | hsa-miR-7199-5p |
| ATP7A | hsa-miR-1247-3p |
| ATP7A | hsa-miR-211-5p |
| ATP7A | hsa-miR-527 |
| ATP7A | hsa-miR-6708-5p |
| ATP7A | hsa-miR-320-3p |
| ATP7A | hsa-miR-7160-5p |
| ATP7A | hsa-miR-320a |
| ATP7A | hsa-miR-7-5p |
| ATP7A | hsa-miR-7670-5p |
| ATP7A | hsa-miR-463-5p |
| ATP7A | hsa-miR-1298-3p |
| ATP7A | hsa-miR-4272 |
| ATP7A | hsa-miR-367-3p |
| ATP7A | hsa-miR-200c-3p |
| ATP7A | hsa-miR-7331-5p |
| ATP7A | hsa-miR-362-5p |
| ATP7A | hsa-miR-3470a |
| ATP7A | hsa-miR-2411-5p |
| ATP7A | hsa-miR-7844-5p |
| ATP7A | hsa-miR-548p |
| ATP7A | hsa-miR-3672 |
| ATP7A | hsa-miR-6724-5p |
| ATP7A | hsa-miR-4675 |
| ATP7A | hsa-miR-3580-3p |
| ATP7A | hsa-miR-4279 |
| ATP7A | hsa-miR-596 |
| ATP7A | hsa-miR-4768-3p |
| ATP7A | hsa-miR-548am-3p |
| ATP7A | hsa-miR-325-5p |
| ATP7A | hsa-miR-3158-3p |
| ATP7A | hsa-miR-629-3p |
| ATP7A | hsa-miR-128-3p |
| ATP7A | hsa-miR-4793-3p |
| ATP7A | hsa-miR-7302-3p |
| ATP7A | hsa-miR-7366-5p |
| ATP7A | hsa-miR-4789-3p |
| ATP7A | hsa-miR-382-3p |
| ATP7A | hsa-miR-4771 |
| ATP7A | hsa-miR-375 |
| ATP7A | hsa-miR-144-3p |
| ATP7A | hsa-miR-18-3p |
| ATP7A | hsa-miR-2681-5p |
| ATP7A | hsa-miR-6403 |
| ATP7A | hsa-miR-8843 |
| ATP7A | hsa-miR-506-3p |
| ATP7A | hsa-miR-505-3p |
| ATP7A | hsa-miR-3913-5p |
| ATP7A | hsa-miR-7015-3p |
| ATP7A | hsa-miR-539-5p |
| ATP7A | hsa-miR-302-3p |
| ATP7A | hsa-miR-3073a-3p |
| ATP7A | hsa-miR-6830-3p |
| ATP7A | hsa-miR-450a-1-3p |
| ATP7A | hsa-miR-7260-5p |
| ATP7A | hsa-miR-7309-3p |
| ATP7A | hsa-miR-8796 |
| ATP7A | hsa-miR-15a-5p |
| ATP7A | hsa-miR-1243-5p |
| ATP7A | hsa-miR-7185-3p |
| ATP7A | hsa-miR-6804-3p |
| ATP7A | hsa-miR-7285-3p |
| ATP7A | hsa-miR-15-5p |
| ATP7A | hsa-miR-3589 |
| ATP7A | hsa-miR-299a-5p |
| ATP7A | hsa-miR-6871-3p |
| ATP7A | hsa-miR-6776-3p |
| ATP7A | hsa-miR-613 |
| ATP7A | hsa-miR-7688-3p |
| ATP7A | hsa-miR-5622-3p |
| ATP7A | hsa-miR-6759-3p |
| ATP7A | hsa-miR-1741 |
| ATP7A | hsa-miR-4789-5p |
| ATP7A | hsa-miR-1224-3p |
| ATP7A | hsa-miR-5010-3p |
| ATP7A | hsa-miR-6919-3p |
| ATP7A | hsa-miR-500 |
| ATP7A | hsa-miR-29a-3p |
| ATP7A | hsa-miR-7009-3p |
| ATP7A | hsa-miR-7394b-5p |
| ATP7A | hsa-miR-197-3p |
| ATP7A | hsa-miR-5098 |
| ATP7A | hsa-miR-3473g |
| ATP7A | hsa-miR-122-5p |
| ATP7A | hsa-miR-1555-5p |
| ATP7A | hsa-miR-592 |
| ATP7A | hsa-miR-137 |
| ATP7A | hsa-miR-3145-3p |
| ATP7A | hsa-miR-7299-3p |
| ATP7A | hsa-miR-4507 |
| ATP7A | hsa-miR-2340 |
| ATP7A | hsa-miR-7314-5p |
| ATP7A | hsa-miR-190-3p |
| ATP7A | hsa-miR-548az-3p |
| ATP7A | hsa-miR-376b-5p |
| ATP7A | hsa-miR-135-5p |
| ATP7A | hsa-miR-7078-3p |
| ATP7A | hsa-miR-8818 |
| ATP7A | hsa-miR-760-3p |
| ATP7A | hsa-miR-4266 |
| ATP7A | hsa-miR-29a-3p |
| ATP7A | hsa-miR-3671 |
| ATP7A | hsa-miR-5582-5p |
| ATP7A | hsa-miR-141-3p |
| ATP7A | hsa-miR-7248-3p |
| ATP7A | hsa-miR-1301-5p |
| ATP7A | hsa-miR-7273-5p |
| ATP7A | hsa-miR-15b-3p |
| ATP7A | hsa-miR-548o-5p |
| ATP7A | hsa-miR-369-3p |
| ATP7A | hsa-miR-518a-5p |
| ATP7A | hsa-miR-766-5p |
| ATP7A | hsa-miR-3945 |
| ATP7A | hsa-miR-599 |
| ATP7A | hsa-miR-1582 |
| ATP7A | hsa-miR-6739-5p |
| ATP7A | hsa-miR-6849-3p |
| ATP7A | hsa-miR-5131 |
| ATP7A | hsa-miR-3058-3p |
| ATP7A | hsa-miR-513a-3p |
| ATP7A | hsa-miR-6827-5p |
| ATP7A | hsa-miR-8881 |
| ATP7A | hsa-miR-1283 |
| ATP7A | hsa-miR-7293-5p |
| ATP7A | hsa-miR-7117-5p |
| ATP7A | hsa-miR-7270-5p |
| ATP7A | hsa-miR-4269 |
| ATP7A | hsa-miR-20b-3p |
| ATP7A | hsa-miR-3079-5p |
| ATP7A | hsa-miR-1434-5p |
| ATP7A | hsa-miR-4524-5p |
| ATP7A | hsa-miR-8875 |
| ATP7A | hsa-miR-6552-3p |
| ATP7A | hsa-miR-214-3p |
| ATP7A | hsa-miR-5088-3p |
| ATP7A | hsa-miR-654-5p |
| ATP7A | hsa-miR-7363-5p |
| ATP7A | hsa-miR-3667-5p |
| ATP7A | hsa-miR-30d-3p |
| ATP7A | hsa-miR-874 |
| ATP7A | hsa-miR-8823 |
| ATP7A | hsa-miR-1231-3p |
| ATP7A | hsa-miR-6129 |
| ATP7A | hsa-miR-3092-5p |
| ATP7A | hsa-miR-484 |
| ATP7A | hsa-miR-1293 |
| ATP7A | hsa-miR-203-3p |
| ATP7A | hsa-miR-7214-5p |
| ATP7A | hsa-miR-7334-3p |
| ATP7A | hsa-miR-6932-3p |
| ATP7A | hsa-miR-380-3p |
| ATP7A | hsa-miR-105b |
| ATP7A | hsa-miR-1292-5p |
| ATP7A | hsa-miR-1301-3p |
| ATP7A | hsa-miR-4328 |
| ATP7A | hsa-miR-431-5p |
| ATP7A | hsa-miR-6871-5p |
| ATP7A | hsa-miR-4534 |
| ATP7A | hsa-miR-5681a |
| ATP7A | hsa-miR-3535 |
| ATP7A | hsa-miR-712-5p |
| ATP7A | hsa-miR-548c-3p |
| ATP7A | hsa-miR-548ah-3p |
| ATP7A | hsa-miR-471-3p |
| ATP7A | hsa-miR-26-3p |
| ATP7A | hsa-miR-584-3p |
| ATP7A | hsa-miR-7202-3p |
| ATP7A | hsa-miR-3622b-5p |
| ATP7A | hsa-miR-624-3p |
| ATP7A | hsa-miR-103a-2-5p |
| ATP7A | hsa-miR-506-3p |
| ATP7A | hsa-miR-4803 |
| ATP7A | hsa-miR-1948-5p |
| ATP7A | hsa-miR-302c-5p |
| ATP7A | hsa-miR-6960-5p |
| ATP7A | hsa-miR-8103 |
| ATP7A | hsa-miR-292a-5p |
| ATP7A | hsa-miR-466b-4-3p |
| ATP7A | hsa-miR-548aj-5p |
| ATP7A | hsa-miR-7397-3p |
| ATP7A | hsa-miR-7374c-3p |
| ATP7A | hsa-miR-4419a |
| ATP7A | hsa-miR-139-5p |
| ATP7A | hsa-miR-4637 |
| ATP7A | hsa-miR-34b-3p |
| ATP7A | hsa-miR-524-3p |
| ATP7A | hsa-miR-7226-3p |
| ATP7A | hsa-miR-2285y |
| ATP7A | hsa-miR-4774-3p |
| ATP7A | hsa-miR-294-3p |
| ATP7A | hsa-miR-871-5p |
| ATP7A | hsa-miR-6536 |
| ATP7A | hsa-miR-3160-3p |
| ATP7A | hsa-miR-6733-5p |
| ATP7A | hsa-miR-456-3p |
| ATP7A | hsa-miR-379-5p |
| ATP7A | hsa-miR-8802 |
| ATP7A | hsa-miR-548an |
| ATP7A | hsa-miR-466b-2-3p |
| ATP7A | hsa-miR-505-5p |
| ATP7A | hsa-miR-525-5p |
| ATP7A | hsa-miR-4662-5p |
| ATP7A | hsa-miR-8055 |
| ATP7A | hsa-miR-211-5p |
| ATP7A | hsa-miR-6746-5p |
| ATP7A | hsa-miR-6357 |
| ATP7A | hsa-miR-2459 |
| ATP7A | hsa-miR-150-5p |
| ATP7A | hsa-miR-6629-5p |
| ATP7A | hsa-miR-548e-3p |
| ATP7A | hsa-miR-8104 |
| ATP7A | hsa-miR-7308-3p |
| ATP7A | hsa-miR-6982-3p |
| ATP7A | hsa-miR-467cd-5p |
| ATP7A | hsa-miR-4753-3p |
| ATP7A | hsa-miR-6722-3p |
| ATP7A | hsa-miR-3974 |
| ATP7A | hsa-miR-680 |
| ATP7A | hsa-miR-6731-3p |
| ATP7A | hsa-miR-8794 |
| ATP7A | hsa-miR-4735-5p |
| ATP7A | hsa-miR-200a-3p |
| ATP7A | hsa-miR-570-3p |
| ATP7A | hsa-miR-548aq-5p |
| ATP7A | hsa-miR-548aj-3p |
| ATP7A | hsa-miR-592-5p |
| ATP7A | hsa-miR-6922-3p |
| ATP7A | hsa-miR-7151-3p |
| ATP7A | hsa-miR-1306-3p |
| ATP7A | hsa-miR-8067 |
| ATP7A | hsa-miR-4524a-5p |
| ATP7A | hsa-miR-4451 |
| ATP7A | hsa-miR-7169-3p |
| ATP7A | hsa-miR-2358 |
| ATP7A | hsa-miR-8052 |
| ATP7A | hsa-miR-4755-5p |
| ATP7A | hsa-miR-3664-5p |
| ATP7A | hsa-miR-32-5p |
| ATP7A | hsa-miR-342-3p |
| ATP7A | hsa-miR-496 |
| ATP7A | hsa-miR-3653-5p |
| ATP7A | hsa-miR-135b-3p |
| ATP7A | hsa-miR-3663-3p |
| ATP7A | hsa-miR-742-5p |
| ATP7A | hsa-miR-2357 |
| ATP7A | hsa-miR-7398l-5p |
| ATP7A | hsa-miR-7328-3p |
| ATP7A | hsa-miR-509-3p |
| ATP7A | hsa-miR-635 |
| ATP7A | hsa-miR-2417 |
| ATP7A | hsa-miR-514a-3p |
| ATP7A | hsa-miR-2446 |
| ATP7A | hsa-miR-345-3p |
| ATP7A | hsa-miR-513b-5p |
| ATP7A | hsa-miR-6778-5p |
| ATP7A | hsa-miR-502-5p |
| ATP7A | hsa-miR-6882-5p |
| ATP7A | hsa-miR-1227-3p |
| ATP7A | hsa-miR-7341-5p |
| ATP7A | hsa-miR-6900-5p |
| ATP7A | hsa-miR-7234-5p |
| ATP7A | hsa-miR-6944-3p |
| ATP7A | hsa-miR-466b-5p |
| ATP7A | hsa-miR-4672 |
| ATP7A | hsa-miR-4659b-3p |
| ATP7A | hsa-miR-623 |
| ATP7A | hsa-miR-2424 |
| ATP7A | hsa-miR-2318 |
| ATP7A | hsa-miR-1662 |
| ATP7A | hsa-miR-4778-3p |
| ATP7A | hsa-miR-7049-5p |
| ATP7A | hsa-miR-1279 |
| ATP7A | hsa-miR-6385 |
| ATP7A | hsa-miR-7683-3p |
| ATP7A | hsa-miR-7300-5p |
| ATP7A | hsa-miR-8888 |
| ATP7A | hsa-miR-548a-3p |
| ATP7A | hsa-miR-7258-5p |
| ATP7A | hsa-miR-4680 |
| ATP7A | hsa-miR-3068-3p |
| ATP7A | hsa-miR-520f-3p |
| ATP7A | hsa-miR-466 |
| ATP7A | hsa-miR-544-3p |
| ATP7A | hsa-miR-6835-3p |
| ATP7A | hsa-miR-548l |
| ATP7A | hsa-miR-7649-3p |
| ATP7A | hsa-miR-6797-3p |
| ATP7A | hsa-miR-7343-3p |
| ATP7A | hsa-miR-5046 |
| ATP7A | hsa-miR-504 |
| ATP7A | hsa-miR-532-5p |
| ATP7A | hsa-miR-196c-3p |
| ATP7A | hsa-miR-203b-3p |
| ATP7A | hsa-miR-667-3p |
| ATP7A | hsa-miR-1912-3p |
| ATP7A | hsa-miR-28-3p |
| ATP7A | hsa-miR-7287-3p |
| ATP7A | hsa-miR-330-3p.2 |
| ATP7A | hsa-miR-208-3p |
| ATP7A | hsa-miR-7387-5p |
| ATP7A | hsa-miR-875-3p |
| ATP7A | hsa-miR-424-5p |
| ATP7A | hsa-miR-21-3p |
| ATP7A | hsa-miR-409-5p |
| ATP7A | hsa-miR-942-3p |
| ATP7A | hsa-miR-548j-3p |
| ATP7A | hsa-miR-562 |
| ATP7A | hsa-miR-152-5p |
| ATP7A | hsa-miR-6868-3p |
| ATP7A | hsa-miR-7026-5p |
| ATP7A | hsa-miR-4425 |
| ATP7A | hsa-miR-6966-3p |
| ATP7A | hsa-miR-6128 |
| ATP7A | hsa-miR-146b-3p |
| ATP7A | hsa-miR-3604 |
| ATP7A | hsa-miR-1277-5p |
| ATP7A | hsa-miR-4695-5p |
| ATP7A | hsa-miR-218-5p |
| ATP7A | hsa-miR-4761-3p |
| ATP7A | hsa-miR-1841 |
| ATP7A | hsa-miR-292b-5p |
| ATP7A | hsa-miR-6937-5p |
| ATP7A | hsa-miR-7649-5p |
| ATP7A | hsa-miR-4434 |
| ATP7A | hsa-miR-342-3p |
| ATP7A | hsa-miR-6926-3p |
| ATP7A | hsa-miR-584-5p |
| ATP7A | hsa-miR-942-5p |
| ATP7A | hsa-miR-6736-3p |
| ATP7A | hsa-miR-5695 |
| ATP7A | hsa-miR-5571-5p |
| ATP7A | hsa-miR-4524a-3p |
| ATP7A | hsa-miR-698-5p |
| ATP7A | hsa-miR-7210-3p |
| ATP7A | hsa-miR-7078-5p |
| ATP7A | hsa-miR-454 |
| ATP7A | hsa-miR-19-5p |
| ATP7A | hsa-miR-891 |
| ATP7A | hsa-miR-3066-3p |
| ATP7A | hsa-miR-2399-5p |
| ATP7A | hsa-miR-3660 |
| ATP7A | hsa-miR-224-3p |
| ATP7A | hsa-miR-8899 |
| ATP7A | hsa-miR-152 |
| ATP7A | hsa-miR-190-5p |
| ATP7A | hsa-miR-6748-5p |
| ATP7A | hsa-miR-2285 |
| ATP7A | hsa-miR-6885-3p |
| ATP7A | hsa-miR-513 |
| ATP7A | hsa-miR-2284g |
| ATP7A | hsa-miR-4778-5p |
| ATP7A | hsa-miR-548x-3p |
| ATP7A | hsa-miR-5587-5p |
| ATP7A | hsa-miR-216b-5p |
| ATP7A | hsa-miR-138-1-3p |
| ATP7A | hsa-miR-1747-5p |
| ATP7A | hsa-miR-1545b-3p |
| ATP7A | hsa-miR-142 |
| ATP7A | hsa-miR-487b-5p |
| ATP7A | hsa-miR-7287-5p |
| ATP7A | hsa-miR-6508-5p |
| ATP7A | hsa-miR-4656 |
| ATP7A | hsa-miR-455-3p.2 |
| ATP7A | hsa-miR-684 |
| ATP7A | hsa-miR-204-3p |
| ATP7A | hsa-miR-432-3p |
| ATP7A | hsa-miR-1949 |
| ATP7A | hsa-miR-4301 |
| ATP7A | hsa-miR-154-3p |
| ATP7A | hsa-miR-495-5p |
| ATP7A | hsa-miR-369-3p |
| ATP7A | hsa-miR-3108-5p |
| ATP7A | hsa-miR-934-5p |
| ATP7A | hsa-miR-455-5p |
| ATP7A | hsa-miR-7027-5p |
| ATP7A | hsa-miR-466q |
| ATP7A | hsa-miR-331-3p |
| ATP7A | hsa-miR-4492 |
| ATP7A | hsa-miR-7035-5p |
| ATP7A | hsa-miR-25-3p |
| ATP7A | hsa-miR-616-3p |
| ATP7A | hsa-miR-106-5p |
| ATP7A | hsa-miR-6946-3p |
| ATP7A | hsa-miR-7301-5p |
| ATP7A | hsa-miR-6929-3p |
| ATP7A | hsa-miR-548as-5p |
| ATP7A | hsa-miR-8060 |
| ATP7A | hsa-miR-7194-5p |
| ATP7A | hsa-miR-409-3p |
| ATP7A | hsa-miR-4653-5p |
| ATP7A | hsa-miR-2422 |
| ATP7A | hsa-miR-3094-3p |
| ATP7A | hsa-miR-6972-3p |
| ATP7A | hsa-miR-6893-5p |
| ATP7A | hsa-miR-1188-5p |
| ATP7A | hsa-miR-3084bc-5p |
| ATP7A | hsa-miR-2288 |
| ATP7A | hsa-miR-101-3p |
| ATP7A | hsa-miR-3962 |
| ATP7A | hsa-miR-1911-3p |
| ATP7A | hsa-miR-556-3p |
| ATP7A | hsa-miR-508-5p |
| ATP7A | hsa-miR-1291 |
| ATP7A | hsa-miR-511 |
| ATP7A | hsa-miR-363-3p |
| ATP7A | hsa-miR-616-3p |
| ATP7A | hsa-miR-7093-5p |
| ATP7A | hsa-miR-4762-5p |
| ATP7A | hsa-miR-7026-3p |
| ATP7A | hsa-miR-4496 |
| ATP7A | hsa-miR-493-3p |
| ATP7A | hsa-miR-6728-3p |
| ATP7A | hsa-miR-1288 |
| ATP7A | hsa-miR-7386e-5p |
| ATP7A | hsa-miR-2896 |
| ATP7A | hsa-miR-30c-2-3p |
| ATP7A | hsa-miR-758-5p |
| ATP7A | hsa-miR-6918-5p |
| ATP7A | hsa-miR-4774-5p |
| ATP7A | hsa-miR-99b-3p |
| ATP7A | hsa-miR-486-5p |
| ATP7A | hsa-miR-6800-5p |
| ATP7A | hsa-miR-4522 |
| ATP7A | hsa-miR-195-5p |
| ATP7A | hsa-miR-8105 |
| ATP7A | hsa-miR-6707-5p |
| ATP7A | hsa-miR-7092-5p |
| ATP7A | hsa-miR-4642 |
| ATP7A | hsa-miR-548as-3p |
| ATP7A | hsa-miR-7317-3p |
| ATP7A | hsa-miR-4257 |
| ATP7A | hsa-miR-6911-3p |
| ATP7A | hsa-miR-7198-3p |
| ATP7A | hsa-miR-466l-3p |
| ATP7A | hsa-miR-411a |
| ATP7A | hsa-miR-384-5p |
| ATP7A | hsa-miR-33a-3p |
| ATP7A | hsa-miR-6891-5p |
| ATP7A | hsa-miR-936 |
| ATP7A | hsa-miR-7366-3p |
| ATP7A | hsa-miR-646 |
| ATP7A | hsa-miR-5088-5p |
| ATP7A | hsa-miR-4659a-3p |
| ATP7A | hsa-miR-3662 |
| ATP7A | hsa-miR-653 |
| ATP7A | hsa-miR-216c-5p |
| ATP7A | hsa-miR-7240-5p |
| ATP7A | hsa-miR-6704-5p |
| ATP7A | hsa-miR-3175 |
| ATP7A | hsa-miR-1903 |
| ATP7A | hsa-miR-3588 |
| ATP7A | hsa-miR-101b-3p.2 |
| ATP7A | hsa-miR-3572-5p |
| ATP7A | hsa-miR-494-3p |
| ATP7A | hsa-miR-3606-3p |
| ATP7A | hsa-miR-5189-5p |
| ATP7A | hsa-miR-6873-3p |
| ATP7A | hsa-miR-4539 |
| ATP7A | hsa-miR-490-3p |
| ATP7A | hsa-miR-548a-5p |
| ATP7A | hsa-let-7abf-3p |
| ATP7A | hsa-miR-139-3p |
| ATP7A | hsa-miR-7010-5p |
| ATP7A | hsa-miR-6130 |
| ATP7A | hsa-miR-2284d |
| ATP7A | hsa-miR-32-5p |
| ATP7A | hsa-miR-516-3p |
| ATP7A | hsa-miR-760 |
| ATP7A | hsa-miR-1837 |
| ATP7A | hsa-miR-3940-5p |
| ATP7A | hsa-miR-4291 |
| ATP7A | hsa-miR-519 |
| ATP7A | hsa-miR-6994-5p |
| ATP7A | hsa-miR-3109-5p |
| ATP7A | hsa-miR-300 |
| ATP7A | hsa-miR-5003-5p |
| ATP7A | hsa-miR-2400 |
| ATP7A | hsa-miR-605 |
| ATP7A | hsa-miR-7065-3p |
| ATP7A | hsa-miR-6542-5p |
| ATP7A | hsa-miR-7371e-3p |
| ATP7A | hsa-miR-674-3p |
| ATP7A | hsa-miR-3153 |
| ATP7A | hsa-miR-7398u-3p |
| ATP7A | hsa-miR-455-3p |
| ATP7A | hsa-miR-7277-3p |
| ATP7A | hsa-miR-661 |
| ATP7A | hsa-miR-412-3p |
| ATP7A | hsa-miR-7154-3p |
| ATP7A | hsa-miR-664 |
| ATP7A | hsa-miR-1545b-3-3p |
| ATP7A | hsa-miR-143-5p |
| ATP7A | hsa-miR-3089-3p |
| ATP7A | hsa-miR-382-5p |
| ATP7A | hsa-miR-3073a-5p |
| ATP7A | hsa-miR-129-5p |
| ATP7A | hsa-miR-2284j |
| ATP7A | hsa-miR-181b-1-3p |
| ATP7A | hsa-miR-5123 |
| ATP7A | hsa-miR-139-5p |
| ATP7A | hsa-miR-2284x |
| ATP7A | hsa-miR-4763-3p |
| ATP7A | hsa-miR-135 |
| ATP7A | hsa-miR-7678-3p |
| ATP7A | hsa-miR-6771-3p |
| ATP7A | hsa-miR-6587-3p |
| ATP7A | hsa-miR-105-5p |
| ATP7A | hsa-miR-2482 |
| ATP7A | hsa-miR-428 |
| ATP7A | hsa-miR-7257-3p |
| ATP7A | hsa-miR-4736 |
| ATP7A | hsa-miR-545-3p |
| ATP7A | hsa-miR-2301 |
| ATP7A | hsa-miR-548bd-3p |
| ATP7A | hsa-miR-6721-5p |
| ATP7A | hsa-miR-518-3p |
| ATP7A | hsa-miR-7339-5p |
| ATP7A | hsa-miR-374c-5p |
| ATP7A | hsa-miR-3910 |
| ATP7A | hsa-miR-2284z |
| ATP7A | hsa-miR-4798-5p |
| ATP7A | hsa-miR-6362 |
| ATP7A | hsa-miR-1273e |
| ATP7A | hsa-miR-3151 |
| ATP7A | hsa-miR-4776-3p |
| ATP7A | hsa-miR-2334 |
| ATP7A | hsa-miR-452-5p |
| ATP7A | hsa-miR-297 |
| ATP7A | hsa-miR-7090-3p |
| ATP7A | hsa-miR-1950 |
| ATP7A | hsa-miR-6951-3p |
| ATP7A | hsa-miR-892-3p |
| ATP7A | hsa-miR-548av-5p |
| ATP7A | hsa-miR-4649-3p |
| ATP7A | hsa-miR-4432 |
| ATP7A | hsa-miR-548au-5p |
| ATP7A | hsa-miR-2325 |
| ATP7A | hsa-miR-1470 |
| ATP7A | hsa-miR-548x-5p |
| ATP7A | hsa-miR-186-3p |
| ATP7A | hsa-miR-103-2-5p |
| ATP7A | hsa-miR-2466-5p |
| ATP7A | hsa-miR-365-5p |
| ATP7A | hsa-miR-378b |
| ATP7A | hsa-miR-219c-3p |
| ATP7A | hsa-miR-3548 |
| ATP7A | hsa-miR-7081-5p |
| ATP7A | hsa-miR-411-5p |
| ATP7A | hsa-miR-7173-3p |
| ATP7A | hsa-miR-16-2-3p |
| ATP7A | hsa-miR-6976-3p |
| ATP7A | hsa-miR-92b-5p |
| ATP7A | hsa-miR-7170-5p |
| ATP7A | hsa-miR-6338 |
| ATP7A | hsa-miR-2284i |
| ATP7A | hsa-miR-4512 |
| ATP7A | hsa-miR-7243-3p |
| ATP7A | hsa-miR-216a |
| ATP7A | hsa-miR-4647 |
| ATP7A | hsa-miR-7282-3p |
| ATP7A | hsa-miR-326-5p |
| ATP7A | hsa-miR-3151-5p |
| ATP7A | hsa-miR-769 |
| ATP7A | hsa-miR-1970 |
| ATP7A | hsa-miR-6942-3p |
| ATP7A | hsa-miR-216b-3p |
| ATP7A | hsa-miR-302d-5p |
| ATP7A | hsa-miR-2285ad |
| ATP7A | hsa-miR-4263 |
| ATP7A | hsa-miR-15b-5p |
| ATP7A | hsa-miR-2284-3p |
| ATP7A | hsa-miR-4256 |
| ATP7A | hsa-miR-31-5p |
| ATP7A | hsa-miR-7657-5p |
| ATP7A | hsa-miR-29b-1-5p |
| ATP7A | hsa-miR-7064-5p |
| ATP7A | hsa-miR-3938 |
| ATP7A | hsa-miR-548ay-5p |
| ATP7A | hsa-miR-294 |
| ATP7A | hsa-miR-7337-3p |
| ATP7A | hsa-miR-660-5p |
| ATP7A | hsa-miR-3093-3p |
| ATP7A | hsa-miR-486-3p |
| ATP7A | hsa-miR-5132-3p |
| ATP7A | hsa-miR-449b-3p |
| ATP7A | hsa-miR-1194 |
| ATP7A | hsa-let-7c-1-3p |
| ATP7A | hsa-miR-669b-5p |
| ATP7A | hsa-miR-4536-5p |
| ATP7A | hsa-miR-125-5p |
| ATP7A | hsa-miR-6792-5p |
| ATP7A | hsa-miR-3473c |
| ATP7A | hsa-miR-25-3p |
| ATP7A | hsa-miR-6345 |
| ATP7A | hsa-miR-7363-3p |
| ATP7A | hsa-miR-7681-5p |
| ATP7A | hsa-miR-7151-5p |
| ATP7A | hsa-miR-3543 |
| ATP7A | hsa-miR-1243-3p |
| ATP7A | hsa-miR-223-3p |
| ATP7A | hsa-miR-4703-5p |
| ATP7A | hsa-miR-135-3p |
| ATP7A | hsa-miR-6996-5p |
| ATP7A | hsa-miR-7386d-3p |
| ATP7A | hsa-miR-502a |
| ATP7A | hsa-miR-7085-5p |
| ATP7A | hsa-miR-548ar-3p |
| ATP7A | hsa-miR-664-3p |
| ATP7A | hsa-miR-2285b |
| ATP7A | hsa-miR-3472 |
| ATP7A | hsa-miR-655-5p |
| ATP7A | hsa-miR-2346 |
| ATP7A | hsa-miR-548f-3p |
| ATP7A | hsa-miR-5197-3p |
| ATP7A | hsa-miR-3102 |
| ATP7A | hsa-miR-20a-3p |
| ATP7A | hsa-miR-16-5p |
| ATP7A | hsa-miR-5107-3p |
| ATP7A | hsa-miR-9a-3p |
| ATP7A | hsa-miR-8874 |
| ATP7A | hsa-miR-4802-5p |
| ATP7A | hsa-miR-5696 |
| ATP7A | hsa-miR-4457 |
| ATP7A | hsa-miR-7342-3p |
| ATP7A | hsa-miR-1233-5p |
| ATP7A | hsa-miR-3929 |
| ATP7A | hsa-miR-344h-3p |
| ATP7A | hsa-miR-6928-3p |
| ATP7A | hsa-miR-122-3p |
| ATP7A | hsa-miR-449a |
| ATP7A | hsa-miR-378a-5p |
| ATP7A | hsa-miR-8054 |
| ATP7A | hsa-miR-3187-5p |
| ATP7A | hsa-miR-2365 |
| ATP7A | hsa-miR-7259-5p |
| ATP7A | hsa-miR-7371d-5p |
| ATP7A | hsa-miR-7207-3p |
| ATP7A | hsa-miR-301 |
| ATP7A | hsa-miR-7193-3p |
| ATP7A | hsa-miR-4643 |
| ATP7A | hsa-miR-7025-3p |
| ATP7A | hsa-miR-1284 |
| ATP7A | hsa-miR-6083 |
| ATP7A | hsa-miR-367-3p |
| ATP7A | hsa-miR-7327-5p |
| ATP7A | hsa-miR-105-5p |
| ATP7A | hsa-miR-421 |
| ATP7A | hsa-miR-290 |
| ATP7A | hsa-miR-434-5p |
| ATP7A | hsa-miR-376bc-5p |
| ATP7A | hsa-miR-625-5p |
| ATP7A | hsa-miR-669n |
| ATP7A | hsa-miR-290b-5p |
| ATP7A | hsa-miR-410 |
| ATP7A | hsa-miR-203-3p.1 |
| ATP7A | hsa-miR-2311 |
| ATP7A | hsa-miR-4270 |
| ATP7A | hsa-miR-124-5p |
| ATP7A | hsa-miR-1388-3p |
| ATP7A | hsa-miR-34-5p |
| ATP7A | hsa-miR-881-3p |
| ATP7A | hsa-miR-130 |
| ATP7A | hsa-miR-6531 |
| ATP7A | hsa-miR-568 |
| ATP7A | hsa-miR-29a-1-5p |
| ATP7A | hsa-miR-223-3p |
| ATP7A | hsa-miR-891b |
| ATP7A | hsa-miR-7235-5p |
| ATP7A | hsa-miR-7360-3p |
| ATP7A | hsa-miR-4741 |
| ATP7A | hsa-miR-6126 |
| ATP7A | hsa-miR-599-5p |
| ATP7A | hsa-miR-548ar-5p |
| ATP7A | hsa-miR-3078-3p |
| ATP7A | hsa-miR-3122 |
| ATP7A | hsa-miR-3978 |
| ATP7A | hsa-miR-7274-3p |
| ATP7A | hsa-miR-4252 |
| ATP7A | hsa-miR-9b-3p |
| ATP7A | hsa-miR-3060-5p |
| ATP7A | hsa-miR-2284v |
| ATP7A | hsa-miR-7251-5p |
| ATP7A | hsa-miR-652-3p |
| ATP7A | hsa-miR-7389-5p |
| ATP7A | hsa-miR-520 |
| ATP7A | hsa-miR-520gh |
| ATP7A | hsa-miR-6986-3p |
| ATP7A | hsa-miR-182-5p |
| ATP7A | hsa-miR-4708-3p |
| ATP7A | hsa-miR-3938-5p |
| ATP7A | hsa-miR-2861 |
| ATP7A | hsa-miR-6315 |
| ATP7A | hsa-miR-7321-5p |
| ATP7A | hsa-miR-2425-5p |
| ATP7A | hsa-miR-6874-5p |
| ATP7A | hsa-miR-1603 |
| ATP7A | hsa-miR-544a |
| ATP7A | hsa-miR-183-3p |
| ATP7A | hsa-miR-7223-3p |
| ATP7A | hsa-miR-6574-5p |
| ATP7A | hsa-miR-3201 |
| ATP7A | hsa-miR-4277 |
| ATP7A | hsa-miR-758-3p |
| ATP7A | hsa-miR-20-3p |
| ATP7A | hsa-miR-30-5p |
| ATP7A | hsa-miR-3096-5p |
| ATP7A | hsa-miR-7386e-3p |
| ATP7A | hsa-miR-3607-3p |
| ATP7A | hsa-miR-4645-5p |
| ATP7A | hsa-miR-7189-5p |
| ATP7A | hsa-miR-191-5p |
| ATP7A | hsa-miR-3596b |
| ATP7A | hsa-miR-320e |
| ATP7A | hsa-miR-7672-5p |
| ATP7A | hsa-miR-675-3p |
| ATP7A | hsa-miR-6859-5p |
| ATP7A | hsa-miR-3126-5p |
| ATP7A | hsa-miR-212-3p |
| ATP7A | hsa-miR-1228-3p |
| ATP7A | hsa-miR-5627-3p |
| ATP7A | hsa-miR-3622a-3p |
| ATP7A | hsa-miR-5000-5p |
| ATP7A | hsa-miR-222-3p |
| ATP7A | hsa-miR-124-3p.1 |
| ATP7A | hsa-miR-6769b-5p |
| ATP7A | hsa-miR-642a-5p |
| ATP7A | hsa-miR-743-5p |
| ATP7A | hsa-miR-1275 |
| ATP7A | hsa-miR-17-5p |
| ATP7A | hsa-miR-7371-5p |
| ATP7A | hsa-miR-872 |
| ATP7A | hsa-miR-763 |
| ATP7A | hsa-miR-676-5p |
| ATP7A | hsa-miR-2406 |
| ATP7A | hsa-miR-1744-5p |
| ATP7A | hsa-miR-7154-5p |
| ATP7A | hsa-miR-3686 |
| ATP7A | hsa-miR-548t-3p |
| ATP7A | hsa-miR-7390-3p |
| ATP7A | hsa-miR-452-3p |
| ATP7A | hsa-miR-6806-5p |
| ATP7A | hsa-miR-302-5p |
| ATP7A | hsa-miR-7183-3p |
| ATP7A | hsa-miR-7386i-3p |
| ATP7A | hsa-miR-1983 |
| ATP7A | hsa-miR-7371b-3p |
| ATP7A | hsa-miR-2320-5p |
| ATP7A | hsa-miR-145-3p |
| ATP7A | hsa-miR-569 |
| ATP7A | hsa-miR-7386f-3p |
| ATP7A | hsa-miR-607 |
| ATP7A | hsa-miR-7329-3p |
| ATP7A | hsa-miR-548c-3p |
| ATP7A | hsa-miR-124-3p.2 |
| ATP7A | hsa-miR-371b-5p |
| ATP7A | hsa-miR-8838 |
| ATP7A | hsa-miR-4446-3p |
| ATP7A | hsa-miR-640 |
| ATP7A | hsa-miR-944 |
| ATP7A | hsa-miR-3145-5p |
| ATP7A | hsa-miR-4635 |
| ATP7A | hsa-miR-291-3p |
| ATP7A | hsa-miR-7307-3p |
| ATP7A | hsa-miR-6546-5p |
| ATP7A | hsa-miR-2312 |
| ATP7A | hsa-miR-3189-3p |
| ATP7A | hsa-miR-2364 |
| ATP7A | hsa-miR-3622b-3p |
| ATP7A | hsa-miR-7169-5p |
| ATP7A | hsa-miR-3173-3p |
| ATP7A | hsa-miR-4719 |
| ATP7A | hsa-miR-1814a |
| ATP7A | hsa-miR-10a-3p |
| ATP7A | hsa-miR-4419b |
| ATP7A | hsa-miR-6967-3p |
| ATP7A | hsa-miR-374b-3p |
| ATP7A | hsa-miR-3609 |
| ATP7A | hsa-miR-6344 |
| ATP7A | hsa-miR-142-3p.2 |
| ATP7A | hsa-miR-548ae-3p |
| ATP7A | hsa-miR-3099 |
| ATP7A | hsa-miR-548ak |
| ATP7A | hsa-miR-548ap-3p |
| ATP7A | hsa-miR-449a-3p |
| ATP7A | hsa-miR-7312-5p |
| ATP7A | hsa-miR-1285-3p |
| ATP7A | hsa-miR-548am-5p |
| ATP7A | hsa-miR-3085 |
| ATP7A | hsa-miR-30c-1-3p |
| ATP7A | hsa-miR-146a-3p |
| ATP7A | hsa-miR-4687-3p |
| ATP7A | hsa-miR-509-5p |
| ATP7A | hsa-miR-6553-3p |
| ATP7A | hsa-miR-376a-5p |
| ATP7A | hsa-miR-3969 |
| ATP7A | hsa-miR-548h-5p |
| ATP7A | hsa-miR-874-3p |
| ATP7A | hsa-miR-2284k |
| ATP7A | hsa-miR-6998-5p |
| ATP7A | hsa-miR-7208-5p |
| ATP7A | hsa-miR-382-5p |
| ATP7A | hsa-miR-4433a-3p |
| ATP7A | hsa-miR-8854 |
| ATP7A | hsa-miR-4418 |
| ATP7A | hsa-miR-124-3p |
| ATP7A | hsa-miR-466b-3p |
| ATP7A | hsa-miR-7370-5p |
| ATP7A | hsa-miR-140-3p |
| ATP7A | hsa-miR-3165 |
| ATP7A | hsa-miR-520g-3p |
| ATP7A | hsa-miR-1-5p |
| ATP7A | hsa-miR-6744-5p |
| ATP7A | hsa-miR-4722-5p |
| ATP7A | hsa-miR-2485 |
| ATP7A | hsa-miR-7854-3p |
| ATP7A | hsa-miR-7453-3p |
| ATP7A | hsa-miR-7006-3p |
| ATP7A | hsa-miR-1678 |
| ATP7A | hsa-miR-101b-3p.1 |
| ATP7A | hsa-miR-7347-3p |
| ATP7A | hsa-miR-30a-3p |
| ATP7A | hsa-miR-3062-5p |
| ATP7A | hsa-miR-5580-3p |
| ATP7A | hsa-miR-7161-3p |
| ATP7A | hsa-miR-488-3p |
| ATP7A | hsa-miR-425 |
| ATP7A | hsa-miR-888-3p |
| ATP7A | hsa-miR-19b-5p |
| ATP7A | hsa-miR-6780a-5p |
| ATP7A | hsa-miR-543-3p |
| ATP7A | hsa-let-7ag-3p |
| ATP7A | hsa-miR-3564 |
| ATP7A | hsa-miR-6790-3p |
| ATP7A | hsa-miR-381-3p |
| ATP7A | hsa-miR-377-3p |
| ATP7A | hsa-miR-7338-3p |
| ATP7A | hsa-miR-2339 |
| ATP7A | hsa-miR-6505-5p |
| ATP7A | hsa-miR-4677-5p |
| ATP7A | hsa-miR-8877 |
| ATP7A | hsa-miR-6947-3p |
| ATP7A | hsa-miR-4471 |
| ATP7A | hsa-miR-510-3p |
| ATP7A | hsa-miR-514a-3p |
| ATP7A | hsa-miR-9-5p |
| ATP7A | hsa-miR-5622-5p |
| ATP7A | hsa-miR-6956-5p |
| ATP7A | hsa-miR-449c-3p |
| ATP7A | hsa-miR-340-5p |
| ATP7A | hsa-miR-7393-3p |
| ATP7A | hsa-miR-677-5p |
| ATP7A | hsa-miR-5623-3p |
| ATP7A | hsa-miR-665 |
| ATP7A | hsa-miR-8880 |
| ATP7A | hsa-miR-514-3p |
| ATP7A | hsa-miR-1264 |
| ATP7A | hsa-miR-3675-3p |
| ATP7A | hsa-miR-214-3p |
| ATP7A | hsa-miR-138-5p |
| ATP7A | hsa-miR-2284 |
| ATP7A | hsa-miR-3149 |
| ATP7A | hsa-miR-3968 |
| ATP7A | hsa-miR-4662b |
| ATP7A | hsa-miR-183-5p |
| ATP7A | hsa-miR-6975-3p |
| ATP7A | hsa-miR-501-5p |
| ATP7A | hsa-miR-3162-3p |
| ATP7A | hsa-miR-6962-3p |
| ATP7A | hsa-miR-200c-3p |
| ATP7A | hsa-miR-10b-5p |
| ATP7A | hsa-miR-6839-3p |
| ATP7A | hsa-miR-7278-3p |
| ATP7A | hsa-miR-501-3p |
| ATP7A | hsa-miR-3073b-3p |
| ATP7A | hsa-miR-133 |
| ATP7A | hsa-miR-8856 |
| ATP7A | hsa-miR-7043-3p |
| ATP7A | hsa-miR-3148 |
| ATP7A | hsa-miR-2284m |
| ATP7A | hsa-miR-1237-3p |
| ATP7A | hsa-miR-631 |
| ATP7A | hsa-miR-548ba |
| ATP7A | hsa-miR-92a-3p |
| ATP7A | hsa-miR-3578 |
| ATP7A | hsa-miR-677 |
| ATP7A | hsa-miR-424-3p |
| ATP7A | hsa-miR-3544-5p |
| ATP7A | hsa-miR-4275 |
| ATP7A | hsa-miR-5583-5p |
| ATP7A | hsa-miR-767-3p |
| ATP7A | hsa-miR-7173-5p |
| ATP7A | hsa-miR-130b-5p |
| ATP7A | hsa-miR-3143 |
| ATP7A | hsa-miR-7005-5p |
| ATP7A | hsa-miR-6864-3p |
| ATP7A | hsa-miR-1685-3p |
| ATP7A | hsa-miR-660-5p |
| ATP7A | hsa-miR-3121-3p |
| ATP7A | hsa-miR-6796-3p |
| ATP7A | hsa-miR-1256 |
| ATP7A | hsa-miR-6917-3p |
| ATP7A | hsa-miR-3086-3p |
| ATP7A | hsa-miR-212-5p |
| ATP7A | hsa-miR-468-5p |
| ATP7A | hsa-miR-3121-3p |
| ATP7A | hsa-miR-4446-5p |
| ATP7A | hsa-miR-3654 |
| ATP7A | hsa-miR-1b-5p |
| ATP7A | hsa-miR-3071-3p |
| ATP7A | hsa-miR-7386d-5p |
| ATP7A | hsa-miR-3664-3p |
| ATP7A | hsa-miR-22-3p |
| ATP7A | hsa-miR-701-3p |
| ATP7A | hsa-miR-3568 |
| ATP7A | hsa-miR-374-5p |
| ATP7A | hsa-miR-4652-3p |
| ATP7A | hsa-miR-8108 |
| ATP7A | hsa-miR-485-3p |
| ATP7A | hsa-miR-743b-5p |
| ATP7A | hsa-miR-324-3p |
| ATP7A | hsa-miR-2386 |
| ATP7A | hsa-miR-576-3p |
| ATP7A | hsa-miR-3200-5p |
| ATP7A | hsa-miR-152-3p |
| ATP7A | hsa-miR-7661-5p |
| ATP7A | hsa-miR-3680-3p |
| ATP7A | hsa-miR-363-5p |
| ATP7A | hsa-miR-523-3p |
| ATP7A | hsa-miR-6756-5p |
| ATP7A | hsa-miR-8098 |
| ATP7A | hsa-miR-942 |
| ATP7A | hsa-miR-548ah-5p |
| ATP7A | hsa-miR-6941-5p |
| ATP7A | hsa-miR-1305 |
| ATP7A | hsa-miR-548d-3p |
| ATP7A | hsa-miR-627-3p |
| ATP7A | hsa-miR-203a-3p.1 |
| ATP7A | hsa-miR-1943-3p |
| ATP7A | hsa-miR-1898 |
| ATP7A | hsa-miR-5619-3p |
| ATP7A | hsa-miR-7253-5p |
| ATP7A | hsa-miR-409a |
| ATP7A | hsa-miR-4742-3p |
| ATP7A | hsa-miR-2398 |
| ATP7A | hsa-miR-1250-3p |
| ATP7A | hsa-miR-2285t |
| ATP7A | hsa-miR-361-5p |
| ATP7A | hsa-miR-6729-3p |
| ATP7A | hsa-miR-7655-5p |
| ATP7A | hsa-miR-142b |
| ATP7A | hsa-miR-6965-5p |
| ATP7A | hsa-miR-4433b-3p |
| ATP7A | hsa-miR-6134 |
| ATP7A | hsa-miR-130-5p |
| ATP7A | hsa-miR-1251-5p |
| ATP7A | hsa-miR-6515-5p |
| ATP7A | hsa-miR-7386h-5p |
| ATP7A | hsa-miR-6809-3p |
| ATP7A | hsa-miR-544b |
| ATP7A | hsa-miR-4493 |
| ATP7A | hsa-miR-4505 |
| ATP7A | hsa-miR-130a-5p |
| ATP7A | hsa-miR-424-5p |
| ATP7A | hsa-miR-467b-5p |
| ATP7A | hsa-miR-7665-3p |
| ATP7A | hsa-miR-7674-3p |
| ATP7A | hsa-miR-874-5p |
| ATP7A | hsa-miR-3529-3p |
| ATP7A | hsa-miR-7398-5p |
| ATP7A | hsa-miR-29-3p |
| ATP7A | hsa-miR-567 |
| ATP7A | hsa-miR-6973b-3p |
| ATP7A | hsa-miR-500-3p |
| ATP7A | hsa-miR-101-3p.2 |
| ATP7A | hsa-miR-7047-5p |
| ATP7A | hsa-miR-489-3p |
| ATP7A | hsa-miR-148 |
| ATP7A | hsa-miR-541-3p |
| ATP7A | hsa-miR-488-3p |
| ATP7A | hsa-miR-2284c |
| ATP7A | hsa-miR-7648-3p |
| ATP7A | hsa-miR-7398b-3p |
| ATP7A | hsa-miR-548i-3p |
| ATP7A | hsa-miR-548av-3p |
| ATP7A | hsa-miR-496-3p |
| ATP7A | hsa-miR-922 |
| ATP7A | hsa-miR-4666a-5p |
| ATP7A | hsa-miR-26-5p |
| ATP7A | hsa-miR-2392 |
| ATP7A | hsa-miR-7116-3p |
| ATP7A | hsa-miR-7203-5p |
| ATP7A | hsa-miR-939-5p |
| ATP7A | hsa-miR-7276-3p |
| ATP7A | hsa-miR-1899 |
| ATP7A | hsa-miR-4426 |
| ATP7A | hsa-miR-503-5p |
| ATP7A | hsa-miR-138-2-3p |
| ATP7A | hsa-miR-6983-3p |
| ATP7A | hsa-miR-18-5p |
| ATP7A | hsa-miR-1271-5p |
| ATP7A | hsa-miR-8081 |
| ATP7A | hsa-miR-196a-1-3p |
| ATP7A | hsa-miR-766-3p |
| ATP7A | hsa-miR-202-5p |
| ATP7A | hsa-miR-1838 |
| ATP7A | hsa-miR-4750-3p |
| ATP7A | hsa-miR-6381 |
| ATP7A | hsa-miR-21-5p |
| ATP7A | hsa-miR-323 |
| ATP7A | hsa-miR-4666b |
| ATP7A | hsa-miR-194-3p |
| ATP7A | hsa-miR-363-3p |
| ATP7A | hsa-miR-7212-5p |
| ATP7A | hsa-miR-7352-3p |
| ATP7A | hsa-miR-7848-3p |
| ATP7A | hsa-miR-5682 |
| ATP7A | hsa-miR-500-5p |
| ATP7A | hsa-miR-593-3p |
| ATP7A | hsa-miR-3583-5p |
| ATP7A | hsa-miR-503-5p |
| ATP7A | hsa-miR-570-5p |
| ATP7A | hsa-miR-145-5p |
| ATP7A | hsa-miR-336-3p |
| ATP7A | hsa-miR-126a-5p |
| ATP7A | hsa-miR-3084-5p |
| ATP7A | hsa-miR-5010-5p |
| ATP7A | hsa-miR-4694-3p |
| ATP7A | hsa-miR-7211-3p |
| ATP7A | hsa-miR-507b |
| ATP7A | hsa-miR-335-5p |
| ATP7A | hsa-miR-576-5p |
| ATP7A | hsa-miR-26 |
| ATP7A | hsa-miR-2894 |
| ATP7A | hsa-miR-502b |
| ATP7A | hsa-miR-98-3p |
| ATP7A | hsa-miR-3200-3p |
| ATP7A | hsa-miR-489 |
| ATP7A | hsa-miR-3561-5p |
| ATP7A | hsa-miR-6770-5p |
| ATP7A | hsa-miR-374 |
| ATP7A | hsa-miR-7118-5p |
| ATP7A | hsa-miR-3591-5p |
| ATP7A | hsa-miR-4724-5p |
| ATP7A | hsa-miR-6900-3p |
| ATP7A | hsa-miR-887-5p |
| ATP7A | hsa-miR-6797-5p |
| ATP7A | hsa-miR-3977 |
| ATP7A | hsa-miR-127-5p |
| ATP7A | hsa-miR-1-3p |
| ATP7A | hsa-miR-1262-5p |
| ATP7A | hsa-miR-4743-3p |
| ATP7A | hsa-miR-3656 |
| ATP7A | hsa-miR-6970-5p |
| ATP7A | hsa-miR-8077 |
| ATP7A | hsa-miR-3180-5p |
| ATP7A | hsa-miR-7175-3p |
| ATP7A | hsa-miR-7013-5p |
| ATP7A | hsa-miR-7313-5p |
| ATP7A | hsa-miR-151-3p |
| ATP7A | hsa-miR-8080 |
| ATP7A | hsa-miR-362-5p |
| ATP7A | hsa-miR-6788-5p |
| ATP7A | hsa-miR-330-3p |
| ATP7A | hsa-miR-101-3p.1 |
| ATP7A | hsa-miR-7371a-5p |
| ATP7A | hsa-miR-206-5p |
| ATP7A | hsa-miR-5687 |
| ATP7A | hsa-miR-7388c-5p |
| ATP7A | hsa-miR-676-3p |
| ATP7A | hsa-miR-4516 |
| ATP7A | hsa-miR-6806-3p |
| ATP7A | hsa-miR-497-5p |
| ATP7A | hsa-miR-2463 |
| ATP7A | hsa-miR-2352 |
| ATP7A | hsa-miR-1253 |
| ATP7A | hsa-miR-921 |
| ATP7A | hsa-miR-940 |
| ATP7A | hsa-miR-5132-5p |
| ATP7A | hsa-miR-7386c-5p |
| ATP7A | hsa-miR-6975-5p |
| ATP7A | hsa-miR-1257 |
| ATP7A | hsa-miR-27-3p |
| ATP7A | hsa-miR-1267 |
| ATP7A | hsa-miR-295-5p |
| ATP7A | hsa-miR-181a-2-3p |
| ATP7A | hsa-miR-7651-3p |
| ATP7A | hsa-miR-4523 |
| ATP7A | hsa-miR-6510-3p |
| ATP7A | hsa-miR-7371b-5p |
| ATP7A | hsa-miR-1955-3p |
| ATP7A | hsa-miR-7092-3p |
| ATP7A | hsa-miR-500a-5p |
| ATP7A | hsa-miR-3084-3p |
| ATP7A | hsa-miR-600 |
| ATP7A | hsa-miR-4766-5p |
| ATP7A | hsa-miR-1285-3p |
| ATP7A | hsa-miR-8082 |
| ATP7A | hsa-miR-888-5p |
| ATP7A | hsa-miR-4438 |
| ATP7A | hsa-miR-513c-3p |
| ATP7A | hsa-miR-6862-5p |
| ATP7A | hsa-miR-491-5p |
| ATP7A | hsa-miR-7024-5p |
| ATP7A | hsa-miR-7271-3p |
| ATP7A | hsa-miR-6829-5p |
| ATP7A | hsa-miR-3105-5p |
| ATP7A | hsa-miR-3185 |
| ATP7A | hsa-miR-7159-5p |
| ATP7A | hsa-miR-1260a |
| ATP7A | hsa-miR-291-5p |
| ATP7A | hsa-miR-149-5p |
| ATP7A | hsa-miR-4787-3p |
| ATP7A | hsa-miR-2116-5p |
| ATP7A | hsa-miR-3605-5p |
| ATP7A | hsa-miR-16-1-3p |
| ATP7A | hsa-miR-3081-5p |
| ATP7A | hsa-miR-641 |
| ATP7A | hsa-miR-6833-3p |
| ATP7A | hsa-miR-766-3p |
| ATP7A | hsa-miR-181 |
| ATP7A | hsa-miR-343 |
| ATP7A | hsa-miR-7344-3p |
| ATP7A | hsa-miR-6320 |
| ATP7A | hsa-miR-642 |
| ATP7A | hsa-miR-499a-3p |
| ATP7A | hsa-miR-7394-3p |
| ATP7A | hsa-miR-526 |
| ATP7A | hsa-miR-7398g-3p |
| ATP7A | hsa-miR-4768-5p |
| ATP7A | hsa-miR-6896-3p |
| ATP7A | hsa-miR-344-5p |
| ATP7A | hsa-miR-6682-3p |
| ATP7A | hsa-miR-371a-5p |
| ATP7A | hsa-miR-3592 |
| ATP7A | hsa-miR-294-5p |
| ATP7A | hsa-miR-5011-5p |
| ATP7A | hsa-miR-7-5p |
| ATP7A | hsa-miR-7a-1-3p |
| ATP7A | hsa-miR-4667-3p |
| ATP7A | hsa-miR-1185-3p |
| ATP7A | hsa-miR-1296-3p |
| ATP7A | hsa-miR-500b-5p |
| ATP7A | hsa-miR-550a-3p |
| ATP7A | hsa-miR-1230 |
| ATP7A | hsa-miR-1434-3p |
| ATP7A | hsa-miR-544a |
| ATP7A | hsa-miR-7164-5p |
| ATP7A | hsa-miR-7216-5p |
| ATP7A | hsa-miR-7245-5p |
| ATP7A | hsa-miR-1909-3p |
| ATP7A | hsa-miR-7061-5p |
| ATP7A | hsa-miR-8083 |
| ATP7A | hsa-miR-2284b |
| ATP7A | hsa-miR-2284-5p |
| ATP7A | hsa-miR-3618-5p |
| ATP7A | hsa-miR-889 |
| ATP7A | hsa-miR-6238 |
| ATP7A | hsa-miR-934-3p |
| ATP7A | hsa-miR-4443 |
| ATP7A | hsa-miR-200bc |
| ATP7A | hsa-miR-499b-5p |
| ATP7A | hsa-miR-200bc-3p |
| ATP7A | hsa-miR-96-3p |
| ATP7A | hsa-miR-548aef |
| ATP7A | hsa-miR-5684 |
| ATP7A | hsa-miR-200a-3p |
| ATP7A | hsa-miR-4433a-5p |
| ATP7A | hsa-miR-633 |
| ATP7A | hsa-miR-7362-3p |
| ATP7A | hsa-miR-92-3p |
| ATP7A | hsa-miR-147-5p |
| ATP7A | hsa-miR-605-3p |
| ATP7A | hsa-miR-200-3p |
| ATP7A | hsa-miR-362-3p |
| ATP7A | hsa-miR-6516-5p |
| ATP7A | hsa-miR-320a |
| ATP7A | hsa-miR-548ac |
| ATP7A | hsa-miR-1252-5p |
| ATP7A | hsa-miR-6957-5p |
| ATP7A | hsa-miR-2276-3p |
| ATP7A | hsa-miR-548g-3p |
| ATP7A | hsa-miR-497-3p |
| ATP7A | hsa-miR-2355-5p |
| ATP7A | hsa-miR-4329 |
| ATP7A | hsa-miR-206 |
| ATP7A | hsa-miR-6132 |
| ATP7A | hsa-miR-669bf-3p |
| ATP7A | hsa-miR-3557-5p |
| ATP7A | hsa-miR-3064-3p |
| ATP7A | hsa-miR-6940-3p |
| ATP7A | hsa-miR-1468-3p |
| ATP7A | hsa-miR-4796-5p |
| ATP7A | hsa-miR-211-3p |
| ATP7A | hsa-miR-7398r-3p |
| ATP7A | hsa-miR-1a-5p |
| ATP7A | hsa-miR-7322-5p |
| ATP7A | hsa-miR-1286 |
| ATP7A | hsa-miR-7674-5p |
| ATP7A | hsa-miR-3102-5p |
| ATP7A | hsa-miR-378g |
| ATP7A | hsa-miR-6122-3p |
| ATP7A | hsa-miR-1292-5p |
| ATP7A | hsa-miR-4728-5p |
| ATP7A | hsa-miR-133b-5p |
| ATP7A | hsa-miR-192-5p |
| ATP7A | hsa-miR-1252 |
| ATP7A | hsa-miR-5583-3p |
| ATP7A | hsa-miR-1207 |
| ATP7A | hsa-miR-1304-3p |
| ATP7A | hsa-miR-561-3p |
| ATP7A | hsa-miR-4494 |
| ATP7A | hsa-miR-190b-3p |
| ATP7A | hsa-miR-1571 |
| ATP7A | hsa-miR-7282-5p |
| ATP7A | hsa-miR-92b-3p |
| ATP7A | hsa-miR-7374b-3p |
| ATP7A | hsa-miR-4764-3p |
| ATP7A | hsa-miR-3199 |
| ATP7A | hsa-miR-580 |
| ATP7A | hsa-miR-6119-3p |
| ATP7A | hsa-miR-133-3p |
| ATP7A | hsa-miR-4261 |
| ATP7A | hsa-miR-371 |
| ATP7A | hsa-miR-6514-5p |
| ATP7A | hsa-miR-7684-5p |
| ATP7A | hsa-miR-3156-3p |
| ATP7A | hsa-miR-301a-5p |
| ATP7A | hsa-miR-6074 |
| ATP7A | hsa-miR-467a-5p |
| ATP7A | hsa-miR-135ab-3p |
| ATP7A | hsa-miR-144-3p |
| ATP7A | hsa-miR-656-5p |
| ATP7A | hsa-miR-6784-3p |
| ATP7A | hsa-miR-512-5p |
| ATP7A | hsa-miR-892b |
| ATP7A | hsa-miR-3646 |
| ATP7A | hsa-miR-6750-5p |
| ATP7A | hsa-miR-499-5p |
| ATP7A | hsa-miR-7843-3p |
| ATP7A | hsa-miR-7386l-3p |
| ATP7A | hsa-miR-153-5p |
| ATP7A | hsa-let-7b-3p |
| ATP7A | hsa-miR-200c-5p |
| ATP7A | hsa-miR-3079-3p |
| ATP7A | hsa-miR-133b |
| ATP7A | hsa-miR-5007-5p |
| ATP7A | hsa-miR-7664-3p |
| ATP7A | hsa-miR-196-5p |
| ATP7A | hsa-miR-497-5p |
| ATP7A | hsa-miR-3129-3p |
| ATP7A | hsa-miR-223-5p |
| ATP7A | hsa-miR-30b-3p |
| ATP7A | hsa-miR-576-5p |
| ATP7A | hsa-miR-5618-3p |
| ATP7A | hsa-miR-5697-3p |
| ATP7A | hsa-miR-7249-3p |
| ATP7A | hsa-miR-548z |
| ATP7A | hsa-miR-6773-5p |
| ATP7A | hsa-miR-218-5p |
| ATP7A | hsa-miR-7323-5p |
| ATP7A | hsa-miR-7462-3p |
| ATP7A | hsa-miR-1248 |
| ATP7A | hsa-miR-628-5p |
| ATP7A | hsa-miR-3556a |
| ATP7A | hsa-let-7f-1-3p |
| ATP7A | hsa-miR-4766-3p |
| ATP7A | hsa-miR-2467-5p |
| ATP7A | hsa-miR-5095 |
| ATP7A | hsa-miR-218a-1-3p |
| ATP7A | hsa-miR-4506 |
| ATP7A | hsa-miR-6848-3p |
| ATP7A | hsa-miR-7350-3p |
| ATP7A | hsa-miR-2322-5p |
| ATP7A | hsa-miR-3083-5p |
| ATP7A | hsa-miR-495-3p |
| ATP7A | hsa-miR-125b-2-3p |
| ATP7A | hsa-miR-29a-5p |
| ATP7A | hsa-miR-148b-3p |
| ATP7A | hsa-miR-6769a-5p |
| ATP7A | hsa-miR-221-3p |
| ATP7A | hsa-miR-1237-3p |
| ATP7A | hsa-miR-7244-5p |
| ATP7A | hsa-miR-7386g-5p |
| ATP7A | hsa-miR-3152-5p |
| ATP7A | hsa-miR-10-5p |
| ATP7A | hsa-miR-7015-5p |
| ATP7A | hsa-miR-2486-5p |
| ATP7A | hsa-miR-7062-5p |
| ATP7A | hsa-miR-2054 |
| ATP7A | hsa-miR-495-3p |
| ATP7A | hsa-miR-6913-3p |
| ATP7A | hsa-miR-1897-5p |
| ATP7A | hsa-miR-93-5p |
| ATP7A | hsa-miR-3575 |
| ATP7A | hsa-miR-3617-5p |
| ATP7A | hsa-miR-149-3p |
| ATP7A | hsa-miR-7398q-3p |
| ATP7A | hsa-miR-295-3p |
| ATP7A | hsa-miR-7398m-3p |
| ATP7A | hsa-miR-7469-5p |
| ATP7A | hsa-miR-3158-5p |
| ATP7A | hsa-miR-1260b |
| ATP7A | hsa-miR-3475-5p |
| ATP7A | hsa-miR-138-3p |
| ATP7A | hsa-miR-30c-3p |
| ATP7A | hsa-miR-8836 |
| ATP7A | hsa-miR-7361-3p |
| ATP7A | hsa-miR-548i |
| ATP7A | hsa-miR-6534 |
| ATP7A | hsa-miR-5698 |
| ATP7A | hsa-miR-373-3p |
| ATP7A | hsa-miR-7021-3p |
| ATP7A | hsa-miR-6935-3p |
| ATP7A | hsa-miR-802-3p |
| ATP7A | hsa-miR-383-5p.2 |
| ATP7A | hsa-miR-4693-3p |
| ATP7A | hsa-miR-7289-5p |
| ATP7A | hsa-miR-6785-5p |
| ATP7A | hsa-miR-1960 |
| ATP7A | hsa-miR-2367-3p |
| ATP7A | hsa-miR-2410 |
| ATP7A | hsa-miR-365-3p |
| ATP7A | hsa-miR-6165 |
| ATP7A | hsa-miR-182-5p |
| ATP7A | hsa-miR-224-5p |
| ATP7A | hsa-miR-7022-3p |
| ATP7A | hsa-miR-6515-3p |
| ATP7A | hsa-miR-185-5p |
| ATP7A | hsa-miR-4477b |
| ATP7A | hsa-miR-7261-3p |
| ATP7A | hsa-miR-144-5p |
| ATP7A | hsa-miR-142-5p |
| ATP7A | hsa-miR-5787 |
| ATP7A | hsa-miR-881-5p |
| ATP7A | hsa-miR-6940-5p |
| ATP7A | hsa-miR-4517 |
| ATP7A | hsa-miR-2371 |
| ATP7A | hsa-miR-6745 |
| ATP7A | hsa-miR-1902 |
| ATP7A | hsa-miR-6507-3p |
| ATP7A | hsa-miR-1227-3p |
| ATP7A | hsa-miR-4468 |
| ATP7A | hsa-miR-3125 |
| ATP7A | hsa-miR-4282 |
| ATP7A | hsa-miR-329-3p |
| ATP7A | hsa-miR-597 |
| ATP7A | hsa-miR-2468 |
| ATP7A | hsa-miR-7279-5p |
| ATP7A | hsa-miR-301-5p |
| ATP7A | hsa-miR-208a-3p |
| ATP7A | hsa-miR-8814 |
| ATP7A | hsa-miR-7355-5p |
| ATP7A | hsa-miR-20-5p |
| ATP7A | hsa-miR-212-3p |
| ATP7A | hsa-miR-4307 |
| ATP7A | hsa-miR-6807-5p |
| ATP7A | hsa-miR-7291-5p |
| ATP7A | hsa-miR-187-3p |
| ATP7A | hsa-miR-5692bc |
| ATP7A | hsa-miR-4498 |
| ATP7A | hsa-miR-6081 |
| ATP7A | hsa-miR-5006-3p |
| ATP7A | hsa-miR-1273d |
| ATP7A | hsa-miR-5125-5p |
| ATP7A | hsa-miR-6768-5p |
| ATP7A | hsa-miR-6912-5p |
| ATP7A | hsa-miR-4499 |
| ATP7A | hsa-miR-378 |
| ATP7A | hsa-miR-548aq-3p |
| ATP7A | hsa-miR-6508-3p |
| ATP7A | hsa-miR-1912-5p |
| ATP7A | hsa-miR-7371f-5p |
| ATP7A | hsa-miR-2467-3p |
| ATP7A | hsa-miR-219a-1-3p |
| ATP7A | hsa-miR-19 |
| ATP7A | hsa-miR-880-3p |
| ATP7A | hsa-miR-1207-5p |
| ATP7A | hsa-miR-7354-3p |
| ATP7A | hsa-miR-291 |
| ATP7A | hsa-miR-148-3p |
| ATP7A | hsa-miR-7045-3p |
| ATP7A | hsa-miR-6870-5p |
| ATP7A | hsa-miR-548ae-5p |
| ATP7A | hsa-miR-302 |
| ATP7A | hsa-miR-380 |
| ATP7A | hsa-miR-7119-3p |
| ATP7A | hsa-miR-7374a-3p |
| ATP7A | hsa-miR-4433b-5p |
| ATP7A | hsa-miR-669j |
| ATP7A | hsa-miR-6730-5p |
| ATP7A | hsa-miR-3102-3p |
| ATP7A | hsa-miR-16-3p |
| ATP7A | hsa-miR-187-5p |
| ATP7A | hsa-miR-1306-5p |
| ATP7A | hsa-miR-1636 |
| ATP7A | hsa-miR-3936 |
| ATP7A | hsa-miR-2399-3p |
| ATP7A | hsa-miR-4673 |
| ATP7A | hsa-miR-24-3p |
| ATP7A | hsa-miR-6511a-5p |
| ATP7A | hsa-miR-3064-5p |
| ATP7A | hsa-miR-3094-5p |
| ATP7A | hsa-miR-6799-5p |
| ATP7A | hsa-miR-448 |
| ATP7A | hsa-miR-7043-5p |
| ATP7A | hsa-miR-590-3p |
| ATP7A | hsa-miR-6822-5p |
| ATP7A | hsa-miR-5584-5p |
| ATP7A | hsa-miR-7150 |
| ATP7A | hsa-miR-6939-3p |
| ATP7A | hsa-miR-3655 |
| ATP7A | hsa-miR-3134 |
| ATP7A | hsa-miR-7016-3p |
| ATP7A | hsa-miR-373-5p |
| ATP7A | hsa-miR-7373d-3p |
| ATP7A | hsa-miR-222-5p |
| ATP7A | hsa-miR-141-5p |
| ATP7A | hsa-miR-329b |
| ATP7A | hsa-miR-4781-3p |
| ATP7A | hsa-miR-3661 |
| ATP7A | hsa-miR-6754-5p |
| ATP7A | hsa-miR-1197 |
| ATP7A | hsa-miR-4317 |
| ATP7A | hsa-miR-4455 |
| ATP7A | hsa-miR-132-3p |
| ATP7A | hsa-miR-155-5p |
| ATP7A | hsa-miR-6502-5p |
| ATP7A | hsa-miR-873-3p |
| ATP7A | hsa-miR-7365-3p |
| ATP7A | hsa-miR-4775 |
| ATP7A | hsa-miR-605-5p |
| ATP7A | hsa-miR-217-5p |
| ATP7A | hsa-miR-301b-5p |
| ATP7A | hsa-miR-1814 |
| ATP7A | hsa-miR-222-3p |
| ATP7A | hsa-miR-2284l |
| ATP7A | hsa-miR-4773 |
| ATP7A | hsa-miR-335-3p |
| ATP7A | hsa-miR-1185-5p |
| ATP7A | hsa-miR-1179 |
| ATP7A | hsa-miR-5001-5p |
| ATP7A | hsa-miR-2293 |
| ATP7A | hsa-miR-322-5p |
| ATP7A | hsa-miR-6894-5p |
| ATP7A | hsa-miR-208-5p |
| ATP7A | hsa-miR-4280 |
| ATP7A | hsa-miR-526b-5p |
| ATP7A | hsa-miR-7106-5p |
| ATP7A | hsa-miR-6948-5p |
| ATP7A | hsa-miR-490-5p |
| ATP7A | hsa-miR-599-3p |
| ATP7A | hsa-miR-375-3p |
| ATP7A | hsa-miR-95-5p |
| ATP7A | hsa-miR-7351-3p |
| ATP7A | hsa-miR-7371e-5p |
| ATP7A | hsa-miR-6507-5p |
| ATP7A | hsa-miR-29 |
| ATP7A | hsa-miR-4478 |
| ATP7A | hsa-miR-7168-5p |
| ATP7A | hsa-miR-548b-5p |
| ATP7A | hsa-miR-329 |
| ATP7A | hsa-let-7a-2-3p |
| ATP7A | hsa-miR-1225-3p |
| ATP7A | hsa-miR-3065-5p |
| ATP7A | hsa-miR-3063-3p |
| ATP7A | hsa-miR-3590-3p |
| ATP7A | hsa-miR-610 |
| ATP7A | hsa-miR-510-5p |
| ATP7A | hsa-miR-6954-5p |
| ATP7A | hsa-miR-7007-5p |
| ATP7A | hsa-miR-520-3p |
| ATP7A | hsa-miR-1966-3p |
| ATP7A | hsa-miR-6998-3p |
| ATP7A | hsa-miR-3925-5p |
| ATP7A | hsa-miR-8059 |
| ATP7A | hsa-miR-34b-5p |
| ATP7A | hsa-miR-7385-5p |
| ATP7A | hsa-miR-7036a-3p |
| ATP7A | hsa-miR-7023-5p |
| ATP7A | hsa-miR-653-5p |
| ATP7A | hsa-miR-3916 |
| ATP7A | hsa-miR-502-5p |
| ATP7A | hsa-miR-6740-3p |
| ATP7A | hsa-miR-6920-3p |
| ATP7A | hsa-miR-7g-3p |
| ATP7A | hsa-miR-7667-3p |
| ATP7A | hsa-miR-7338-5p |
| ATP7A | hsa-miR-6760-5p |
| ATP7A | hsa-miR-7275-5p |
| ATP7A | hsa-miR-7386i-5p |
| ATP7A | hsa-miR-519-5p |
| ATP7A | hsa-miR-518-5p |
| ATP7A | hsa-miR-3100-5p |
| ATP7A | hsa-miR-577-5p |
| ATP7A | hsa-miR-215 |
| ATP7A | hsa-miR-6821-3p |
| ATP7A | hsa-miR-7344-5p |
| ATP7A | hsa-miR-4723-5p |
| ATP7A | hsa-miR-580-5p |
| ATP7A | hsa-miR-1271-5p |
| ATP7A | hsa-miR-4663 |
| ATP7A | hsa-miR-548 |
| ATP7A | hsa-miR-7386l-5p |
| ATP7A | hsa-miR-203 |
| ATP7A | hsa-miR-6867-5p |
| ATP7A | hsa-miR-5011-3p |
| ATP7A | hsa-miR-7062-3p |
| ATP7A | hsa-miR-4650-3p |
| ATP7A | hsa-miR-2333 |
| ATP7A | hsa-miR-101-3p |
| ATP7A | hsa-miR-3938-3p |
| ATP7A | hsa-miR-7082-5p |
| ATP7A | hsa-miR-7247-5p |
| ATP7A | hsa-miR-7681-3p |
| ATP7A | hsa-miR-2351 |
| ATP7A | hsa-miR-4276 |
| ATP7A | hsa-miR-669i |
| ATP7A | hsa-miR-590-5p |
| ATP7A | hsa-miR-7340-3p |
| ATP7A | hsa-miR-6916-5p |
| ATP7A | hsa-miR-924 |
| ATP7A | hsa-miR-802 |
| ATP7A | hsa-miR-548c-5p |
| ATP7A | hsa-miR-16-5p |
| ATP7A | hsa-miR-548f-5p |
| ATP7A | hsa-miR-302c-3p.2 |
| ATP7A | hsa-miR-7398l-3p |
| ATP7A | hsa-miR-6076 |
| ATP7A | hsa-miR-344g-3p |
| ATP7A | hsa-miR-150-3p |
| ATP7A | hsa-miR-5129-5p |
| ATP7A | hsa-miR-7353-5p |
| ATP7A | hsa-miR-6713-3p |
| ATP7A | hsa-miR-6888-3p |
| ATP7A | hsa-miR-628-3p |
| ATP7A | hsa-miR-6961-5p |
| ATP7A | hsa-miR-7355-3p |
| ATP7A | hsa-miR-2393 |
| ATP7A | hsa-miR-559 |
| ATP7A | hsa-miR-423-5p |
| ATP7A | hsa-miR-6216 |
| ATP7A | hsa-miR-2458 |
| ATP7A | hsa-miR-548ai |
| ATP7A | hsa-miR-7304-3p |
| ATP7A | hsa-miR-3585-5p |
| ATP7A | hsa-miR-7069-3p |
| ATP7A | hsa-miR-2117 |
| ATP7A | hsa-miR-548aw |
| ATP7A | hsa-miR-548h-3p |
| ATP7A | hsa-miR-6758-3p |
| ATP7A | hsa-miR-1827 |
| ATP7A | hsa-miR-2310 |
| ATP7A | hsa-miR-196b-3p |
| ATP7A | hsa-miR-1544-3p |
| ATP7A | hsa-miR-582-3p |
| ATP7A | hsa-miR-3618 |
| ATP7A | hsa-miR-1198-3p |
| ATP7A | hsa-miR-1264-3p |
| ATP7A | hsa-miR-7370-3p |
| ATP7A | hsa-miR-2284ab |
| ATP7A | hsa-miR-192-3p |
| ATP7A | hsa-miR-1894-5p |
| ATP7A | hsa-miR-520f-5p |
| ATP7A | hsa-miR-2303 |
| ATP7A | hsa-miR-6836-5p |
| ATP7A | hsa-miR-493-5p |
| ATP7A | hsa-miR-505-3p.2 |
| ATP7A | hsa-miR-1643a-5p |
| ATP7A | hsa-miR-6951-5p |
| ATP7A | hsa-miR-6765-3p |
| ATP7A | hsa-miR-322-3p |
| ATP7A | hsa-miR-8087 |
| ATP7A | hsa-miR-4699-3p |
| ATP7A | hsa-miR-4693-5p |
| ATP7A | hsa-miR-642-3p |
| ATP7A | hsa-miR-3560 |
| ATP7A | hsa-miR-873-5p |
| ATP7A | hsa-miR-7398-3p |
| ATP7A | hsa-miR-29-5p |
| ATP7A | hsa-miR-547-3p |
| ATP7A | hsa-miR-199-3p |
| ATP7A | hsa-miR-3924 |
| ATP7A | hsa-miR-548at-5p |
| ATP7A | hsa-miR-372-5p |
| ATP7A | hsa-miR-4536-5p |
| ATP7A | hsa-miR-8901 |
| ATP7A | hsa-miR-548y |
| ATP7A | hsa-miR-694 |
| ATP7A | hsa-miR-26-2-3p |
| ATP7A | hsa-miR-186-5p |
| ATP7A | hsa-miR-582-5p |
| ATP7A | hsa-miR-6354 |
| ATP7A | hsa-miR-1262-3p |
| ATP7A | hsa-miR-3098-5p |
| ATP7A | hsa-miR-4427 |
| ATP7A | hsa-miR-383-3p |
| ATP7A | hsa-miR-582-5p |
| ATP7A | hsa-miR-8100 |
| ATP7A | hsa-miR-92 |
| ATP7A | hsa-miR-8805 |
| ATP7A | hsa-miR-1261 |
| ATP7A | hsa-miR-216 |
| ATP7A | hsa-miR-548ad-5p |
| ATP7A | hsa-miR-6356 |
| ATP7A | hsa-miR-3163 |
| ATP7A | hsa-miR-6991-5p |
| ATP7A | hsa-miR-1252-3p |
| ATP7A | hsa-miR-4253 |
| ATP7A | hsa-miR-6971-5p |
| ATP7A | hsa-miR-300-5p |
| ATP7A | hsa-miR-6525 |
| ATP7A | hsa-miR-26b-3p |
| ATP7A | hsa-miR-3664-5p |
| ATP7A | hsa-miR-188-3p |
| ATP7A | hsa-miR-7332-3p |
| ATP7A | hsa-miR-543 |
| ATP7A | hsa-miR-129-5p |
| ATP7A | hsa-miR-290a-5p |
| ATP7A | hsa-miR-8793 |
| ATP7A | hsa-miR-7056-5p |
| ATP7A | hsa-miR-205-3p |
| ATP7A | hsa-miR-3084a-5p |
| ATP7A | hsa-miR-3559-5p |
| ATP7A | hsa-miR-133a-3p.2 |
| ATP7B | hsa-miR-29b-5p |
| ATP7B | hsa-miR-7667-5p |
| ATP7B | hsa-miR-4759 |
| ATP7B | hsa-miR-7192-5p |
| ATP7B | hsa-miR-1299 |
| ATP7B | hsa-miR-4762-3p |
| ATP7B | hsa-miR-3566 |
| ATP7B | hsa-miR-6902-3p |
| ATP7B | hsa-miR-6715b-5p |
| ATP7B | hsa-miR-7028-5p |
| ATP7B | hsa-miR-4273 |
| ATP7B | hsa-miR-6862-3p |
| ATP7B | hsa-miR-7049-5p |
| ATP7B | hsa-miR-7192-3p |
| ATP7B | hsa-miR-500b-3p |
| ATP7B | hsa-miR-15b-3p |
| ATP7B | hsa-miR-3909 |
| ATP7B | hsa-miR-7031-3p |
| ATP7B | hsa-miR-4667-5p |
| ATP7B | hsa-miR-3096-5p |
| ATP7B | hsa-miR-1266 |
| ATP7B | hsa-miR-7163-5p |
| ATP7B | hsa-miR-199b-3-5p |
| ATP7B | hsa-miR-383 |
| ATP7B | hsa-miR-4503 |
| ATP7B | hsa-miR-5004-5p |
| ATP7B | hsa-miR-519 |
| ATP7B | hsa-miR-1295b-5p |
| ATP7B | hsa-miR-350 |
| ATP7B | hsa-miR-6805-5p |
| ATP7B | hsa-miR-329-5p |
| ATP7B | hsa-miR-7008-3p |
| ATP7B | hsa-miR-5194 |
| ATP7B | hsa-miR-3100-3p |
| ATP7B | hsa-miR-4481 |
| ATP7B | hsa-miR-434-3p |
| ATP7B | hsa-miR-939-3p |
| ATP7B | hsa-miR-6719-3p |
| ATP7B | hsa-miR-2323 |
| ATP7B | hsa-miR-4511 |
| ATP7B | hsa-miR-6854-5p |
| ATP7B | hsa-miR-128 |
| ATP7B | hsa-miR-612 |
| ATP7B | hsa-miR-483-5p |
| ATP7B | hsa-miR-7194-5p |
| ATP7B | hsa-miR-2113 |
| ATP7B | hsa-miR-34a-3p |
| ATP7B | hsa-miR-33-5p |
| ATP7B | hsa-miR-4279 |
| ATP7B | hsa-miR-7277-5p |
| ATP7B | hsa-miR-409 |
| ATP7B | hsa-miR-7039-3p |
| ATP7B | hsa-miR-761 |
| ATP7B | hsa-miR-351-5p |
| ATP7B | hsa-miR-1982-3p |
| ATP7B | hsa-miR-346-3p |
| ATP7B | hsa-miR-3159 |
| ATP7B | hsa-miR-106-3p |
| ATP7B | hsa-miR-3688-5p |
| ATP7B | hsa-miR-130-3p |
| ATP7B | hsa-miR-383-5p.1 |
| ATP7B | hsa-miR-4492 |
| ATP7B | hsa-miR-4425 |
| ATP7B | hsa-miR-219-1-3p |
| ATP7B | hsa-miR-1546-5p |
| ATP7B | hsa-miR-411-5p |
| ATP7B | hsa-miR-2441 |
| ATP7B | hsa-miR-433-5p |
| ATP7B | hsa-miR-5004-3p |
| ATP7B | hsa-miR-6772-3p |
| ATP7B | hsa-miR-7296-5p |
| ATP7B | hsa-miR-2284-5p |
| ATP7B | hsa-miR-873-5p.1 |
| ATP7B | hsa-miR-6764-5p |
| ATP7B | hsa-miR-1243-5p |
| ATP7B | hsa-miR-1188-3p |
| ATP7B | hsa-miR-599 |
| ATP7B | hsa-miR-3576 |
| ATP7B | hsa-miR-7386k-5p |
| ATP7B | hsa-miR-7107-3p |
| ATP7B | hsa-miR-2883 |
| ATP7B | hsa-miR-1191b-5p |
| ATP7B | hsa-miR-181b-2-3p |
| ATP7B | hsa-miR-7285-5p |
| ATP7B | hsa-miR-6120-3p |
| ATP7B | hsa-miR-141-3p |
| ATP7B | hsa-miR-4311 |
| ATP7B | hsa-miR-6866-5p |
| ATP7B | hsa-miR-7664-5p |
| ATP7B | hsa-miR-4726-3p |
| ATP7B | hsa-miR-1262-5p |
| ATP7B | hsa-miR-1-3p |
| ATP7B | hsa-miR-1915-3p |
| ATP7B | hsa-miR-1231-3p |
| ATP7B | hsa-miR-7347-5p |
| ATP7B | hsa-miR-4632-5p |
| ATP7B | hsa-miR-34b-3p |
| ATP7B | hsa-miR-7242-5p |
| ATP7B | hsa-miR-7843-5p |
| ATP7B | hsa-miR-3155a |
| ATP7B | hsa-miR-329a |
| ATP7B | hsa-miR-101a-5p |
| ATP7B | hsa-miR-326-3p |
| ATP7B | hsa-miR-337-3p |
| ATP7B | hsa-miR-4648 |
| ATP7B | hsa-miR-2893 |
| ATP7B | hsa-miR-6758-5p |
| ATP7B | hsa-miR-7044-5p |
| ATP7B | hsa-miR-1273h-5p |
| ATP7B | hsa-miR-1205 |
| ATP7B | hsa-miR-1258-5p |
| ATP7B | hsa-miR-7090-5p |
| ATP7B | hsa-miR-136-5p |
| ATP7B | hsa-miR-3473c |
| ATP7B | hsa-miR-7094b-2-5p |
| ATP7B | hsa-miR-4434 |
| ATP7B | hsa-miR-6501-3p |
| ATP7B | hsa-miR-584-5p |
| ATP7B | hsa-miR-5096 |
| ATP7B | hsa-miR-3571 |
| ATP7B | hsa-miR-3106-3p |
| ATP7B | hsa-miR-150-5p |
| ATP7B | hsa-miR-6720-5p |
| ATP7B | hsa-miR-1260a |
| ATP7B | hsa-miR-191-3p |
| ATP7B | hsa-miR-7342-3p |
| ATP7B | hsa-miR-7386j-3p |
| ATP7B | hsa-miR-3106-5p |
| ATP7B | hsa-miR-92a-1-5p |
| ATP7B | hsa-miR-22-5p |
| ATP7B | hsa-miR-7007-3p |
| ATP7B | hsa-miR-7398a-3p |
| ATP7B | hsa-miR-877-5p |
| ATP7B | hsa-miR-1264-5p |
| ATP7B | hsa-miR-2466-3p |
| ATP7B | hsa-miR-6335 |
| ATP7B | hsa-miR-4709-5p |
| ATP7B | hsa-miR-551-5p |
| ATP7B | hsa-let-7-3p |
| ATP7B | hsa-miR-651-3p |
| ATP7B | hsa-miR-7333-5p |
| ATP7B | hsa-miR-181b-3p |
| ATP7B | hsa-miR-2464-5p |
| ATP7B | hsa-miR-7024-3p |
| ATP7B | hsa-miR-7578 |
| ATP7B | hsa-miR-6128 |
| ATP7B | hsa-miR-204-5p |
| ATP7B | hsa-miR-2277-3p |
| ATP7B | hsa-miR-760 |
| ATP7B | hsa-miR-7159-3p |
| ATP7B | hsa-miR-4685-5p |
| ATP7B | hsa-miR-6819-3p |
| ATP7B | hsa-miR-7164-5p |
| ATP7B | hsa-miR-136-5p |
| ATP7B | hsa-miR-4738-3p |
| ATP7B | hsa-miR-4518 |
| ATP7B | hsa-miR-7075-5p |
| ATP7B | hsa-miR-340-5p |
| ATP7B | hsa-miR-7042-3p |
| ATP7B | hsa-miR-4739 |
| ATP7B | hsa-miR-7281-3p |
| ATP7B | hsa-miR-6945-5p |
| ATP7B | hsa-miR-24-5p |
| ATP7B | hsa-miR-7680-5p |
| ATP7B | hsa-miR-29b-1-5p |
| ATP7B | hsa-miR-7173-3p |
| ATP7B | hsa-miR-7187-3p |
| ATP7B | hsa-miR-7651-5p |
| ATP7B | hsa-miR-6919-3p |
| ATP7B | hsa-miR-6908-3p |
| ATP7B | hsa-miR-190-3p |
| ATP7B | hsa-miR-8080 |
| ATP7B | hsa-miR-1197-5p |
| ATP7B | hsa-let-7c-1-3p |
| ATP7B | hsa-miR-4791 |
| ATP7B | hsa-miR-2373-3p |
| ATP7B | hsa-miR-7003-3p |
| ATP7B | hsa-miR-429 |
| ATP7B | hsa-miR-4650 |
| ATP7B | hsa-miR-7340-5p |
| ATP7B | hsa-miR-562 |
| ATP7B | hsa-miR-6982-3p |
| ATP7B | hsa-miR-2329-5p |
| ATP7B | hsa-miR-4725-5p |
| ATP7B | hsa-miR-320e |
| ATP7B | hsa-miR-298-3p |
| ATP7B | hsa-miR-340-3p |
| ATP7B | hsa-miR-518-3p |
| ATP7B | hsa-miR-4441 |
| ATP7B | hsa-miR-6840-3p |
| ATP7B | hsa-miR-216a-3p |
| ATP7B | hsa-miR-124 |
| ATP7B | hsa-miR-4461 |
| ATP7B | hsa-miR-3612 |
| ATP7B | hsa-miR-7386e-3p |
| ATP7B | hsa-miR-6879-5p |
| ATP7B | hsa-miR-3652 |
| ATP7B | hsa-miR-6938-5p |
| ATP7B | hsa-miR-669hk-3p |
| ATP7B | hsa-miR-504-5p |
| ATP7B | hsa-miR-490-3p |
| ATP7B | hsa-miR-100-3p |
| ATP7B | hsa-miR-15 |
| ATP7B | hsa-miR-698-5p |
| ATP7B | hsa-miR-7847-3p |
| ATP7B | hsa-miR-10 |
| ATP7B | hsa-miR-6837-5p |
| ATP7B | hsa-miR-3059-3p |
| ATP7B | hsa-miR-6830-5p |
| ATP7B | hsa-miR-4457 |
| ATP7B | hsa-miR-6778-5p |
| ATP7B | hsa-miR-5685 |
| ATP7B | hsa-miR-760-3p |
| ATP7B | hsa-miR-885-3p |
| ATP7B | hsa-miR-1239 |
| ATP7B | hsa-miR-7012-5p |
| ATP7B | hsa-miR-5585-3p |
| ATP7B | hsa-miR-6831-5p |
| ATP7B | hsa-miR-1260b |
| ATP7B | hsa-miR-186-5p |
| ATP7B | hsa-miR-320-3p |
| ATP7B | hsa-miR-4792 |
| ATP7B | hsa-miR-3689-3p |
| ATP7B | hsa-miR-7224-3p |
| ATP7B | hsa-miR-8895 |
| ATP7B | hsa-miR-6368 |
| ATP7B | hsa-miR-194-2-3p |
| ATP7B | hsa-miR-3573-5p |
| ATP7B | hsa-miR-1253 |
| ATP7B | hsa-miR-6090 |
| ATP7B | hsa-miR-377-5p |
| ATP7B | hsa-miR-548ad-3p |
| ATP7B | hsa-miR-10b-5p |
| ATP7B | hsa-miR-326 |
| ATP7B | hsa-miR-6404 |
| ATP7B | hsa-miR-8098 |
| ATP7B | hsa-miR-7855-5p |
| ATP7B | hsa-miR-7302-3p |
| ATP7B | hsa-miR-7246-3p |
| ATP7B | hsa-miR-219a-2-3p |
| ATP7B | hsa-miR-4788 |
| ATP7B | hsa-miR-487-5p |
| ATP7B | hsa-miR-6346 |
| ATP7B | hsa-miR-7662-5p |
| ATP7B | hsa-miR-4760-5p |
| ATP7B | hsa-miR-7658-3p |
| ATP7B | hsa-miR-379-5p |
| ATP7B | hsa-miR-574-5p |
| ATP7B | hsa-miR-6877-3p |
| ATP7B | hsa-miR-4282 |
| ATP7B | hsa-miR-2368-3p |
| ATP7B | hsa-miR-6824-3p |
| ATP7B | hsa-miR-146-5p |
| ATP7B | hsa-miR-8064 |
| ATP7B | hsa-miR-5626-5p |
| ATP7B | hsa-miR-4657 |
| ATP7B | hsa-miR-7276-3p |
| ATP7B | hsa-miR-466b-5p |
| ATP7B | hsa-miR-5193 |
| ATP7B | hsa-miR-6939-5p |
| ATP7B | hsa-miR-7386k-3p |
| ATP7B | hsa-miR-4687-3p |
| ATP7B | hsa-miR-4682 |
| ATP7B | hsa-miR-548at-3p |
| ATP7B | hsa-miR-548as-3p |
| ATP7B | hsa-miR-4772-5p |
| ATP7B | hsa-miR-7274-3p |
| ATP7B | hsa-miR-3154 |
| ATP7B | hsa-miR-1273g-3p |
| ATP7B | hsa-miR-93-5p |
| ATP7B | hsa-miR-154-5p |
| ATP7B | hsa-miR-3934-3p |
| ATP7B | hsa-miR-7231-5p |
| ATP7B | hsa-miR-153-5p |
| ATP7B | hsa-miR-1839-5p |
| ATP7B | hsa-miR-1840 |
| ATP7B | hsa-miR-1243-3p |
| ATP7B | hsa-miR-545-3p |
| ATP7B | hsa-miR-548ah-5p |
| ATP7B | hsa-miR-540-3p |
| ATP7B | hsa-miR-577 |
| ATP7B | hsa-miR-3572-5p |
| ATP7B | hsa-miR-548az-5p |
| ATP7B | hsa-miR-583 |
| ATP7B | hsa-miR-548az-3p |
| ATP7B | hsa-miR-4318 |
| ATP7B | hsa-miR-6860 |
| ATP7B | hsa-miR-7197-3p |
| ATP7B | hsa-miR-3145-3p |
| ATP7B | hsa-miR-7179-3p |
| ATP7B | hsa-miR-7032-5p |
| ATP7B | hsa-miR-6076 |
| ATP7B | hsa-miR-7232-3p |
| ATP7B | hsa-miR-494 |
| ATP7B | hsa-miR-6791-5p |
| ATP7B | hsa-miR-3092-5p |
| ATP7B | hsa-miR-3097-5p |
| ATP7B | hsa-miR-568 |
| ATP7B | hsa-miR-2296 |
| ATP7B | hsa-miR-6792-5p |
| ATP7B | hsa-miR-5582-5p |
| ATP7B | hsa-miR-5617-5p |
| ATP7B | hsa-miR-516-3p |
| ATP7B | hsa-miR-32-5p |
| ATP7B | hsa-miR-193 |
| ATP7B | hsa-miR-138-2-3p |
| ATP7B | hsa-miR-2377 |
| ATP7B | hsa-miR-216 |
| ATP7B | hsa-miR-146 |
| ATP7B | hsa-miR-664-5p |
| ATP7B | hsa-miR-7262a-5p |
| ATP7B | hsa-miR-3067-5p |
| ATP7B | hsa-miR-7053-5p |
| ATP7B | hsa-miR-5134-5p |
| ATP7B | hsa-miR-138-5p |
| ATP7B | hsa-miR-7009-3p |
| ATP7B | hsa-miR-3135b |
| ATP7B | hsa-miR-3613-3p |
| ATP7B | hsa-miR-6986-3p |
| ATP7B | hsa-miR-5626-3p |
| ATP7B | hsa-miR-6773-3p |
| ATP7B | hsa-miR-5134-3p |
| ATP7B | hsa-miR-134-3p |
| ATP7B | hsa-miR-6407 |
| ATP7B | hsa-miR-7293-5p |
| ATP7B | hsa-miR-2446 |
| ATP7B | hsa-miR-544-5p |
| ATP7B | hsa-miR-138-5p |
| ATP7B | hsa-miR-181b-1-3p |
| ATP7B | hsa-miR-4495 |
| ATP7B | hsa-miR-587 |
| ATP7B | hsa-miR-6904-5p |
| ATP7B | hsa-miR-8800 |
| ATP7B | hsa-miR-7670-5p |
| ATP7B | hsa-miR-1227-3p |
| ATP7B | hsa-miR-6856-5p |
| ATP7B | hsa-miR-520-5p |
| ATP7B | hsa-miR-344-3p |
| ATP7B | hsa-miR-1233-5p |
| ATP7B | hsa-miR-1286 |
| ATP7B | hsa-miR-23b-5p |
| ATP7B | hsa-miR-6804-5p |
| ATP7B | hsa-miR-1251-5p |
| ATP7B | hsa-miR-1839-3p |
| ATP7B | hsa-miR-6818-5p |
| ATP7B | hsa-miR-2347 |
| ATP7B | hsa-miR-4801 |
| ATP7B | hsa-miR-4768-3p |
| ATP7B | hsa-miR-6893-5p |
| ATP7B | hsa-miR-26a-3p |
| ATP7B | hsa-miR-6513-3p |
| ATP7B | hsa-miR-7650-5p |
| ATP7B | hsa-miR-103-3p |
| ATP7B | hsa-miR-6902-5p |
| ATP7B | hsa-miR-219-3p |
| ATP7B | hsa-miR-29-3p |
| ATP7B | hsa-miR-5586-3p |
| ATP7B | hsa-miR-1956-5p |
| ATP7B | hsa-miR-4292 |
| ATP7B | hsa-miR-328-5p |
| ATP7B | hsa-miR-185-5p |
| ATP7B | hsa-miR-3562 |
| ATP7B | hsa-miR-4753-5p |
| ATP7B | hsa-miR-4531 |
| ATP7B | hsa-miR-199-1-5p |
| ATP7B | hsa-miR-6785-5p |
| ATP7B | hsa-miR-7166-3p |
| ATP7B | hsa-miR-1843 |
| ATP7B | hsa-miR-6990-5p |
| ATP7B | hsa-miR-670-5p |
| ATP7B | hsa-miR-6887-5p |
| ATP7B | hsa-miR-589-5p |
| ATP7B | hsa-miR-410 |
| ATP7B | hsa-miR-6086 |
| ATP7B | hsa-miR-25-3p |
| ATP7B | hsa-miR-4761-5p |
| ATP7B | hsa-miR-449b-3p |
| ATP7B | hsa-miR-7234-5p |
| ATP7B | hsa-miR-424-5p |
| ATP7B | hsa-miR-667-5p |
| ATP7B | hsa-miR-7843-3p |
| ATP7B | hsa-miR-34b-3p |
| ATP7B | hsa-miR-2985 |
| ATP7B | hsa-miR-4443 |
| ATP7B | hsa-miR-210-5p |
| ATP7B | hsa-miR-152-5p |
| ATP7B | hsa-miR-4793-5p |
| ATP7B | hsa-miR-373-3p |
| ATP7B | hsa-miR-548t-5p |
| ATP7B | hsa-miR-7239-5p |
| ATP7B | hsa-miR-497-5p |
| ATP7B | hsa-miR-5703 |
| ATP7B | hsa-miR-3613b |
| ATP7B | hsa-miR-154-5p |
| ATP7B | hsa-miR-671-5p |
| ATP7B | hsa-miR-376ab |
| ATP7B | hsa-miR-367-3p |
| ATP7B | hsa-miR-525-5p |
| ATP7B | hsa-miR-18-3p |
| ATP7B | hsa-miR-519e-3p |
| ATP7B | hsa-miR-128-3p |
| ATP7B | hsa-miR-195-5p |
| ATP7B | hsa-miR-7036b-3p |
| ATP7B | hsa-miR-548g-5p |
| ATP7B | hsa-miR-2415-3p |
| ATP7B | hsa-miR-548e-3p |
| ATP7B | hsa-miR-1958 |
| ATP7B | hsa-miR-7657-5p |
| ATP7B | hsa-miR-6350 |
| ATP7B | hsa-miR-8106 |
| ATP7B | hsa-miR-147a |
| ATP7B | hsa-miR-19a-5p |
| ATP7B | hsa-miR-544a |
| ATP7B | hsa-miR-5006-5p |
| ATP7B | hsa-miR-7010-5p |
| ATP7B | hsa-miR-5582-3p |
| ATP7B | hsa-miR-502-5p |
| ATP7B | hsa-miR-2308 |
| ATP7B | hsa-miR-4786-3p |
| ATP7B | hsa-miR-3618-3p |
| ATP7B | hsa-miR-376a-2-5p |
| ATP7B | hsa-miR-6885-5p |
| ATP7B | hsa-miR-6715-5p |
| ATP7B | hsa-miR-98-3p |
| ATP7B | hsa-miR-525-5p |
| ATP7B | hsa-miR-3660 |
| ATP7B | hsa-miR-377-3p |
| ATP7B | hsa-miR-7681-5p |
| ATP7B | hsa-miR-2286 |
| ATP7B | hsa-miR-6987-3p |
| ATP7B | hsa-miR-7214-5p |
| ATP7B | hsa-miR-6324 |
| ATP7B | hsa-miR-379-5p |
| ATP7B | hsa-miR-150-5p |
| ATP7B | hsa-miR-6753-3p |
| ATP7B | hsa-miR-7050-3p |
| ATP7B | hsa-miR-330-3p.1 |
| ATP7B | hsa-miR-3191-5p |
| ATP7B | hsa-miR-548f-5p |
| ATP7B | hsa-miR-4755-3p |
| ATP7B | hsa-miR-455-3p |
| ATP7B | hsa-miR-140-3p |
| ATP7B | hsa-miR-3138 |
| ATP7B | hsa-miR-3925-3p |
| ATP7B | hsa-miR-122-5p |
| ATP7B | hsa-miR-7226-5p |
| ATP7B | hsa-miR-7015-3p |
| ATP7B | hsa-miR-107-3p |
| ATP7B | hsa-miR-655-5p |
| ATP7B | hsa-miR-7027-5p |
| ATP7B | hsa-miR-879-5p |
| ATP7B | hsa-miR-6754-5p |
| ATP7B | hsa-miR-5107-3p |
| ATP7B | hsa-miR-548t-3p |
| ATP7B | hsa-let-7-5p |
| ATP7B | hsa-miR-543-3p |
| ATP7B | hsa-miR-490-3p |
| ATP7B | hsa-miR-6134 |
| ATP7B | hsa-miR-467f |
| ATP7B | hsa-miR-7116-3p |
| ATP7B | hsa-miR-202-3p |
| ATP7B | hsa-miR-7037-3p |
| ATP7B | hsa-miR-501-3p |
| ATP7B | hsa-miR-2682-3p |
| ATP7B | hsa-miR-7066-3p |
| ATP7B | hsa-miR-3187-5p |
| ATP7B | hsa-miR-666-5p |
| ATP7B | hsa-miR-1297 |
| ATP7B | hsa-miR-1303 |
| ATP7B | hsa-miR-1912 |
| ATP7B | hsa-miR-7321-3p |
| ATP7B | hsa-miR-30-3p |
| ATP7B | hsa-miR-7018-5p |
| ATP7B | hsa-miR-6792-3p |
| ATP7B | hsa-miR-1224-3p |
| ATP7B | hsa-miR-3550 |
| ATP7B | hsa-miR-3653-5p |
| ATP7B | hsa-miR-3962 |
| ATP7B | hsa-miR-4450 |
| ATP7B | hsa-miR-636 |
| ATP7B | hsa-miR-3103-5p |
| ATP7B | hsa-miR-627-3p |
| ATP7B | hsa-miR-8550 |
| ATP7B | hsa-miR-2431-3p |
| ATP7B | hsa-miR-190a-3p |
| ATP7B | hsa-miR-539-5p |
| ATP7B | hsa-miR-653 |
| ATP7B | hsa-miR-4769-5p |
| ATP7B | hsa-miR-3975 |
| ATP7B | hsa-miR-2330-5p |
| ATP7B | hsa-miR-2453 |
| ATP7B | hsa-miR-1938 |
| ATP7B | hsa-miR-5110 |
| ATP7B | hsa-miR-2359 |
| ATP7B | hsa-miR-548ax |
| ATP7B | hsa-let-7f-2-3p |
| ATP7B | hsa-miR-2285r |
| ATP7B | hsa-miR-7272-3p |
| ATP7B | hsa-miR-570-3p |
| ATP7B | hsa-miR-548h-5p |
| ATP7B | hsa-miR-195-5p |
| ATP7B | hsa-miR-875-3p |
| ATP7B | hsa-miR-2444 |
| ATP7B | hsa-miR-877-5p |
| ATP7B | hsa-miR-376a-5p |
| ATP7B | hsa-miR-3078-3p |
| ATP7B | hsa-miR-6828-3p |
| ATP7B | hsa-miR-3620-3p |
| ATP7B | hsa-miR-6338 |
| ATP7B | hsa-miR-6954-3p |
| ATP7B | hsa-miR-7371d-5p |
| ATP7B | hsa-miR-6759-3p |
| ATP7B | hsa-miR-6322 |
| ATP7B | hsa-miR-7683-3p |
| ATP7B | hsa-miR-374a-3p |
| ATP7B | hsa-miR-6517 |
| ATP7B | hsa-miR-3097-3p |
| ATP7B | hsa-miR-887-5p |
| ATP7B | hsa-miR-5001-5p |
| ATP7B | hsa-miR-6328 |
| ATP7B | hsa-miR-6983-5p |
| ATP7B | hsa-miR-4743-3p |
| ATP7B | hsa-miR-7052-5p |
| ATP7B | hsa-miR-6926-3p |
| ATP7B | hsa-miR-19-3p |
| ATP7B | hsa-miR-1948-3p |
| ATP7B | hsa-miR-382-5p |
| ATP7B | hsa-miR-7386h-3p |
| ATP7B | hsa-miR-494-3p |
| ATP7B | hsa-miR-7059-3p |
| ATP7B | hsa-miR-2483-3p |
| ATP7B | hsa-miR-7191-3p |
| ATP7B | hsa-miR-5189-5p |
| ATP7B | hsa-miR-548aa |
| ATP7B | hsa-miR-1913 |
| ATP7B | hsa-miR-7312-3p |
| ATP7B | hsa-miR-888-3p |
| ATP7B | hsa-miR-7686-3p |
| ATP7B | hsa-miR-6841-3p |
| ATP7B | hsa-miR-758-3p |
| ATP7B | hsa-miR-570-3p |
| ATP7B | hsa-miR-4430 |
| ATP7B | hsa-miR-669b-5p |
| ATP7B | hsa-miR-650 |
| ATP7B | hsa-miR-224-5p |
| ATP7B | hsa-miR-3607-3p |
| ATP7B | hsa-miR-548aj-3p |
| ATP7B | hsa-miR-2352 |
| ATP7B | hsa-miR-296-3p |
| ATP7B | hsa-miR-6863 |
| ATP7B | hsa-miR-6511-5p |
| ATP7B | hsa-miR-3084bc-5p |
| ATP7B | hsa-miR-4758-5p |
| ATP7B | hsa-miR-6761-5p |
| ATP7B | hsa-miR-194-1-3p |
| ATP7B | hsa-miR-4679 |
| ATP7B | hsa-miR-7664-3p |
| ATP7B | hsa-miR-32-5p |
| ATP7B | hsa-miR-6845-3p |
| ATP7B | hsa-miR-541-3p |
| ATP7B | hsa-miR-3084-3p |
| ATP7B | hsa-miR-3099-3p |
| ATP7B | hsa-miR-7175-5p |
| ATP7B | hsa-miR-1247-3p |
| ATP7B | hsa-miR-7076-5p |
| ATP7B | hsa-miR-7-5p |
| ATP7B | hsa-miR-6764-3p |
| ATP7B | hsa-miR-210-3p |
| ATP7B | hsa-miR-451b |
| ATP7B | hsa-miR-3593-5p |
| ATP7B | hsa-miR-5787 |
| ATP7B | hsa-miR-7390-3p |
| ATP7B | hsa-miR-214-3p |
| ATP7B | hsa-miR-6849-3p |
| ATP7B | hsa-miR-6935-5p |
| ATP7B | hsa-miR-367-3p |
| ATP7B | hsa-miR-139-5p |
| ATP7B | hsa-miR-181-3p |
| ATP7B | hsa-miR-214-5p |
| ATP7B | hsa-miR-106-5p |
| ATP7B | hsa-miR-5585-5p |
| ATP7B | hsa-miR-1932 |
| ATP7B | hsa-miR-515-5p |
| ATP7B | hsa-miR-7646-3p |
| ATP7B | hsa-miR-3912-5p |
| ATP7B | hsa-miR-6540-3p |
| ATP7B | hsa-miR-548c-3p |
| ATP7B | hsa-miR-4685-3p |
| ATP7B | hsa-miR-6911-5p |
| ATP7B | hsa-miR-2365 |
| ATP7B | hsa-miR-5112 |
| ATP7B | hsa-miR-380-3p |
| ATP7B | hsa-miR-7116-5p |
| ATP7B | hsa-miR-548a-3p |
| ATP7B | hsa-miR-7108-5p |
| ATP7B | hsa-miR-741-5p |
| ATP7B | hsa-miR-124-3p.1 |
| ATP7B | hsa-miR-6381 |
| ATP7B | hsa-miR-7087-5p |
| ATP7B | hsa-miR-216-5p |
| ATP7B | hsa-miR-6511-3p |
| ATP7B | hsa-miR-6932-5p |
| ATP7B | hsa-miR-1290 |
| ATP7B | hsa-miR-376ab-3p |
| ATP7B | hsa-miR-2299-5p |
| ATP7B | hsa-miR-31-5p |
| ATP7B | hsa-miR-650abc |
| ATP7B | hsa-miR-339-3p |
| ATP7B | hsa-miR-8858 |
| ATP7B | hsa-miR-455-3p.1 |
| ATP7B | hsa-miR-504 |
| ATP7B | hsa-miR-145-5p |
| ATP7B | hsa-miR-3061-3p |
| ATP7B | hsa-miR-6769b-3p |
| ATP7B | hsa-miR-3087-5p |
| ATP7B | hsa-miR-3125 |
| ATP7B | hsa-miR-30c-2-3p |
| ATP7B | hsa-miR-7081-5p |
| ATP7B | hsa-miR-548ap-3p |
| ATP7B | hsa-miR-4745-5p |
| ATP7B | hsa-miR-186-3p |
| ATP7B | hsa-miR-762 |
| ATP7B | hsa-miR-7113-5p |
| ATP7B | hsa-miR-7304-3p |
| ATP7B | hsa-miR-2887 |
| ATP7B | hsa-miR-1193 |
| ATP7B | hsa-miR-133 |
| ATP7B | hsa-miR-16-2-3p |
| ATP7B | hsa-miR-4316 |
| ATP7B | hsa-miR-4257 |
| ATP7B | hsa-miR-2284aa |
| ATP7B | hsa-miR-6972-3p |
| ATP7B | hsa-miR-4252 |
| ATP7B | hsa-miR-6319 |
| ATP7B | hsa-miR-671-5p |
| ATP7B | hsa-miR-4707-5p |
| ATP7B | hsa-miR-677 |
| ATP7B | hsa-miR-6744-5p |
| ATP7B | hsa-miR-1207-3p |
| ATP7B | hsa-miR-3911 |
| ATP7B | hsa-miR-4716-5p |
| ATP7B | hsa-miR-6795-5p |
| ATP7B | hsa-miR-4691-5p |
| ATP7B | hsa-miR-4491 |
| ATP7B | hsa-miR-505-5p |
| ATP7B | hsa-miR-532-5p |
| ATP7B | hsa-miR-635 |
| ATP7B | hsa-miR-7240-5p |
| ATP7B | hsa-miR-98-5p |
| ATP7B | hsa-miR-4269 |
| ATP7B | hsa-miR-660-3p |
| ATP7B | hsa-miR-465d-3p |
| ATP7B | hsa-miR-7318-5p |
| ATP7B | hsa-miR-7315-3p |
| ATP7B | hsa-miR-6132 |
| ATP7B | hsa-miR-573 |
| ATP7B | hsa-miR-7385a-3p |
| ATP7B | hsa-miR-376a-3p |
| ATP7B | hsa-miR-3667-5p |
| ATP7B | hsa-miR-3688-3p |
| ATP7B | hsa-miR-7314-5p |
| ATP7B | hsa-miR-381-3p |
| ATP7B | hsa-miR-3927-3p |
| ATP7B | hsa-miR-741-3p |
| ATP7B | hsa-miR-7193-5p |
| ATP7B | hsa-miR-669c-3p |
| ATP7B | hsa-miR-3060-3p |
| ATP7B | hsa-miR-2327 |
| ATP7B | hsa-miR-6975-5p |
| ATP7B | hsa-miR-8108 |
| ATP7B | hsa-miR-432-5p |
| ATP7B | hsa-miR-6932-3p |
| ATP7B | hsa-miR-2136 |
| ATP7B | hsa-miR-4635 |
| ATP7B | hsa-miR-466l-3p |
| ATP7B | hsa-miR-7398p-3p |
| ATP7B | hsa-miR-2307 |
| ATP7B | hsa-miR-483-3p.2 |
| ATP7B | hsa-miR-181a-2-3p |
| ATP7B | hsa-miR-584-5p |
| ATP7B | hsa-miR-7386m-3p |
| ATP7B | hsa-miR-4482-3p |
| ATP7B | hsa-miR-1270 |
| ATP7B | hsa-miR-4656 |
| ATP7B | hsa-miR-763 |
| ATP7B | hsa-miR-4287 |
| ATP7B | hsa-miR-6917-5p |
| ATP7B | hsa-let-7a-2-3p |
| ATP7B | hsa-miR-7162-3p |
| ATP7B | hsa-miR-449-5p |
| ATP7B | hsa-miR-4472 |
| ATP7B | hsa-miR-519-3p |
| ATP7B | hsa-miR-330-5p |
| ATP7B | hsa-miR-6909-5p |
| ATP7B | hsa-miR-6516 |
| ATP7B | hsa-miR-129-5p |
| ATP7B | hsa-miR-548av-3p |
| ATP7B | hsa-miR-331-3p |
| ATP7B | hsa-miR-361-3p |
| ATP7B | hsa-miR-324-5p |
| ATP7B | hsa-miR-140-5p |
| ATP7B | hsa-miR-7374b-3p |
| ATP7B | hsa-miR-9-3p |
| ATP7B | hsa-miR-4763-3p |
| ATP7B | hsa-miR-7220-3p |
| ATP7B | hsa-miR-663b |
| ATP7B | hsa-miR-128-5p |
| ATP7B | hsa-miR-7227-5p |
| ATP7B | hsa-miR-27b-5p |
| ATP7B | hsa-miR-380 |
| ATP7B | hsa-miR-342-5p |
| ATP7B | hsa-miR-6715-3p |
| ATP7B | hsa-miR-3616-5p |
| ATP7B | hsa-miR-2309 |
| ATP7B | hsa-miR-6385 |
| ATP7B | hsa-miR-684 |
| ATP7B | hsa-miR-874-5p |
| ATP7B | hsa-miR-7258-5p |
| ATP7B | hsa-miR-6089 |
| ATP7B | hsa-miR-7055-5p |
| ATP7B | hsa-miR-4667 |
| ATP7B | hsa-miR-1343-5p |
| ATP7B | hsa-miR-4728-5p |
| ATP7B | hsa-miR-508-5p |
| ATP7B | hsa-miR-5130 |
| ATP7B | hsa-miR-26-3p |
| ATP7B | hsa-miR-1668 |
| ATP7B | hsa-miR-4438 |
| ATP7B | hsa-miR-3927-3p |
| ATP7B | hsa-miR-4324 |
| ATP7B | hsa-miR-493-3p |
| ATP7B | hsa-miR-298-5p |
| ATP7B | hsa-miR-1950 |
| ATP7B | hsa-miR-2311 |
| ATP7B | hsa-miR-708-5p |
| ATP7B | hsa-miR-218-2-3p |
| ATP7B | hsa-miR-3084-5p |
| ATP7B | hsa-miR-201-3p |
| ATP7B | hsa-miR-6959-5p |
| ATP7B | hsa-miR-8103 |
| ATP7B | hsa-miR-7198-5p |
| ATP7B | hsa-miR-125 |
| ATP7B | hsa-miR-6807-5p |
| ATP7B | hsa-miR-5124a |
| ATP7B | hsa-miR-3090-3p |
| ATP7B | hsa-miR-34c-5p |
| ATP7B | hsa-miR-503-5p |
| ATP7B | hsa-miR-4710 |
| ATP7B | hsa-miR-7190-5p |
| ATP7B | hsa-miR-15c-3p |
| ATP7B | hsa-miR-2331-3p |
| ATP7B | hsa-miR-200bc-3p |
| ATP7B | hsa-miR-548n |
| ATP7B | hsa-miR-7241-3p |
| ATP7B | hsa-miR-484 |
| ATP7B | hsa-miR-6721-5p |
| ATP7B | hsa-miR-6724-5p |
| ATP7B | hsa-miR-7362-3p |
| ATP7B | hsa-miR-133b-5p |
| ATP7B | hsa-miR-1915-5p |
| ATP7B | hsa-miR-2278 |
| ATP7B | hsa-miR-7106-5p |
| ATP7B | hsa-miR-20 |
| ATP7B | hsa-miR-7156-5p |
| ATP7B | hsa-miR-706 |
| ATP7B | hsa-miR-5691 |
| ATP7B | hsa-miR-3474 |
| ATP7B | hsa-miR-6119-5p |
| ATP7B | hsa-miR-7398t-3p |
| ATP7B | hsa-miR-502-5p |
| ATP7B | hsa-miR-669bf-3p |
| ATP7B | hsa-miR-519e-5p |
| ATP7B | hsa-miR-410-3p |
| ATP7B | hsa-miR-3058-5p |
| ATP7B | hsa-miR-7182-3p |
| ATP7B | hsa-miR-873a-5p.2 |
| ATP7B | hsa-miR-6779-5p |
| ATP7B | hsa-miR-6993-5p |
| ATP7B | hsa-miR-6816-3p |
| ATP7B | hsa-miR-450b-3p |
| ATP7B | hsa-miR-3431 |
| ATP7B | hsa-miR-193b-5p |
| ATP7B | hsa-miR-1468-3p |
| ATP7B | hsa-miR-30-5p |
| ATP7B | hsa-miR-615-5p |
| ATP7B | hsa-miR-7188-3p |
| ATP7B | hsa-miR-4684-5p |
| ATP7B | hsa-miR-4516 |
| ATP7B | hsa-miR-1972 |
| ATP7B | hsa-miR-766-3p |
| ATP7B | hsa-miR-15a-3p |
| ATP7B | hsa-miR-5683 |
| ATP7B | hsa-miR-6859-5p |
| ATP7B | hsa-miR-3186-5p |
| ATP7B | hsa-miR-4645-5p |
| ATP7B | hsa-miR-653-5p |
| ATP7B | hsa-miR-346 |
| ATP7B | hsa-miR-7033-5p |
| ATP7B | hsa-miR-3622a-3p |
| ATP7B | hsa-miR-891a-3p |
| ATP7B | hsa-miR-340-5p |
| ATP7B | hsa-miR-2904 |
| ATP7B | hsa-miR-1544-3p |
| ATP7B | hsa-miR-344eh-5p |
| ATP7B | hsa-miR-4664-5p |
| ATP7B | hsa-miR-7174-3p |
| ATP7B | hsa-miR-204-3p |
| ATP7B | hsa-miR-4436b-3p |
| ATP7B | hsa-miR-126-5p |
| ATP7B | hsa-miR-6922-5p |
| ATP7B | hsa-miR-1229-3p |
| ATP7B | hsa-miR-644a |
| ATP7B | hsa-miR-324 |
| ATP7B | hsa-miR-1285-3p |
| ATP7B | hsa-miR-7684-5p |
| ATP7B | hsa-let-7 |
| ATP7B | hsa-miR-335-5p |
| ATP7B | hsa-miR-548f-3p |
| ATP7B | hsa-miR-1976 |
| ATP7B | hsa-miR-331-3p |
| ATP7B | hsa-miR-6865-3p |
| ATP7B | hsa-miR-6808-5p |
| ATP7B | hsa-miR-6973a-5p |
| ATP7B | hsa-miR-4746-5p |
| ATP7B | hsa-miR-2116-5p |
| ATP7B | hsa-miR-1260a |
| ATP7B | hsa-miR-149-5p |
| ATP7B | hsa-miR-375-3p |
| ATP7B | hsa-miR-548j-3p |
| ATP7B | hsa-miR-4459 |
| ATP7B | hsa-miR-181 |
| ATP7B | hsa-miR-365-5p |
| ATP7B | hsa-miR-4653-5p |
| ATP7B | hsa-miR-2361 |
| ATP7B | hsa-miR-6417 |
| ATP7B | hsa-miR-3558-3p |
| ATP7B | hsa-miR-4677-3p |
| ATP7B | hsa-miR-548aq-3p |
| ATP7B | hsa-miR-6514-3p |
| ATP7B | hsa-miR-935 |
| ATP7B | hsa-miR-3069-5p |
| ATP7B | hsa-miR-4275 |
| ATP7B | hsa-miR-767-3p |
| ATP7B | hsa-miR-3622b-3p |
| ATP7B | hsa-miR-504-5p.1 |
| ATP7B | hsa-miR-6731-5p |
| ATP7B | hsa-miR-7649-3p |
| ATP7B | hsa-miR-7648-5p |
| ATP7B | hsa-miR-4526 |
| ATP7B | hsa-miR-548cej-3p |
| ATP7B | hsa-miR-876-5p |
| ATP7B | hsa-miR-1238-3p |
| ATP7B | hsa-miR-7056-3p |
| ATP7B | hsa-miR-5187-3p |
| ATP7B | hsa-miR-3609 |
| ATP7B | hsa-miR-302-3p |
| ATP7B | hsa-miR-3158-5p |
| ATP7B | hsa-miR-6957-5p |
| ATP7B | hsa-miR-3575 |
| ATP7B | hsa-miR-6992-3p |
| ATP7B | hsa-miR-2300-5p |
| ATP7B | hsa-miR-6809-5p |
| ATP7B | hsa-miR-4307 |
| ATP7B | hsa-miR-98-5p |
| ATP7B | hsa-miR-6405 |
| ATP7B | hsa-miR-6977-5p |
| ATP7B | hsa-miR-548aj-5p |
| ATP7B | hsa-miR-3664-3p |
| ATP7B | hsa-miR-8114 |
| ATP7B | hsa-miR-200a-3p |
| ATP7B | hsa-miR-7372-3p |
| ATP7B | hsa-miR-125-5p |
| ATP7B | hsa-miR-6388 |
| ATP7B | hsa-miR-3577 |
| ATP7B | hsa-miR-7092-5p |
| ATP7B | hsa-miR-6858-5p |
| ATP7B | hsa-miR-4447 |
| ATP7B | hsa-miR-152-3p |
| ATP7B | hsa-miR-4470 |
| ATP7B | hsa-miR-551-3p |
| ATP7B | hsa-miR-670-3p |
| ATP7B | hsa-miR-7058-5p |
| ATP7B | hsa-miR-548ay-3p |
| ATP7B | hsa-miR-127-5p |
| ATP7B | hsa-miR-197-3p |
| ATP7B | hsa-miR-1964-5p |
| ATP7B | hsa-miR-2382-5p |
| ATP7B | hsa-miR-591 |
| ATP7B | hsa-miR-4722-5p |
| ATP7B | hsa-miR-548g-3p |
| ATP7B | hsa-miR-323 |
| ATP7B | hsa-miR-6507-3p |
| ATP7B | hsa-miR-6801-5p |
| ATP7B | hsa-miR-497-3p |
| ATP7B | hsa-miR-4737 |
| ATP7B | hsa-miR-183-3p |
| ATP7B | hsa-miR-6877-5p |
| ATP7B | hsa-miR-644a |
| ATP7B | hsa-miR-7078-5p |
| ATP7B | hsa-miR-330-3p |
| ATP7B | hsa-miR-140-3p.2 |
| ATP7B | hsa-miR-6732-3p |
| ATP7B | hsa-miR-23-3p |
| ATP7B | hsa-miR-7008-5p |
| ATP7B | hsa-miR-6071 |
| ATP7B | hsa-miR-4251 |
| ATP7B | hsa-miR-4514 |
| ATP7B | hsa-miR-1896 |
| ATP7B | hsa-miR-30d-3p |
| ATP7B | hsa-miR-1245b-3p |
| ATP7B | hsa-miR-7163-3p |
| ATP7B | hsa-miR-6965-5p |
| ATP7B | hsa-miR-544b |
| ATP7B | hsa-miR-3079-3p |
| ATP7B | hsa-miR-890-3p |
| ATP7B | hsa-miR-4677-5p |
| ATP7B | hsa-miR-93-5p |
| ATP7B | hsa-miR-1842 |
| ATP7B | hsa-miR-106 |
| ATP7B | hsa-miR-7292-5p |
| ATP7B | hsa-miR-5003-5p |
| ATP7B | hsa-miR-4645-3p |
| ATP7B | hsa-miR-8119 |
| ATP7B | hsa-miR-142-5p |
| ATP7B | hsa-miR-580-5p |
| ATP7B | hsa-miR-567 |
| ATP7B | hsa-miR-7109-5p |
| ATP7B | hsa-miR-3113-5p |
| ATP7B | hsa-miR-2425-5p |
| ATP7B | hsa-miR-2328-5p |
| ATP7B | hsa-miR-29 |
| ATP7B | hsa-miR-380-5p |
| ATP7B | hsa-miR-6888-3p |
| ATP7B | hsa-miR-8089 |
| ATP7B | hsa-miR-302 |
| ATP7B | hsa-miR-7854-3p |
| ATP7B | hsa-miR-548aw |
| ATP7B | hsa-miR-199 |
| ATP7B | hsa-miR-3922-5p |
| ATP7B | hsa-miR-381-5p |
| ATP7B | hsa-miR-4767 |
| ATP7B | hsa-miR-3185 |
| ATP7B | hsa-miR-7106-3p |
| ATP7B | hsa-miR-6774-5p |
| ATP7B | hsa-miR-6929-5p |
| ATP7B | hsa-miR-6784-3p |
| ATP7B | hsa-miR-383-5p.2 |
| ATP7B | hsa-miR-6975-3p |
| ATP7B | hsa-miR-3113-3p |
| ATP7B | hsa-miR-205-5p |
| ATP7B | hsa-miR-7666-3p |
| ATP7B | hsa-miR-7235-5p |
| ATP7B | hsa-miR-2285j |
| ATP7B | hsa-miR-707 |
| ATP7B | hsa-miR-2313-5p |
| ATP7B | hsa-miR-669g |
| ATP7B | hsa-miR-146b-3p |
| ATP7B | hsa-miR-8073 |
| ATP7B | hsa-miR-5002-5p |
| ATP7B | hsa-miR-7074-5p |
| ATP7B | hsa-miR-1195 |
| ATP7B | hsa-miR-7198-3p |
| ATP7B | hsa-miR-455-5p |
| ATP7B | hsa-miR-28-5p |
| ATP7B | hsa-miR-3614-3p |
| ATP7B | hsa-miR-4789-3p |
| ATP7B | hsa-miR-1291 |
| ATP7B | hsa-miR-6951-3p |
| ATP7B | hsa-miR-7057-5p |
| ATP7B | hsa-miR-124-3p |
| ATP7B | hsa-miR-677-5p |
| ATP7B | hsa-miR-5009-3p |
| ATP7B | hsa-miR-7005-5p |
| ATP7B | hsa-miR-8076 |
| ATP7B | hsa-miR-4766-3p |
| ATP7B | hsa-miR-4649-3p |
| ATP7B | hsa-miR-7041-5p |
| ATP7B | hsa-miR-7356-5p |
| ATP7B | hsa-miR-4270 |
| ATP7B | hsa-miR-7249-3p |
| ATP7B | hsa-miR-7354-5p |
| ATP7B | hsa-miR-378d |
| ATP7B | hsa-miR-6894-3p |
| ATP7B | hsa-miR-409b |
| ATP7B | hsa-miR-1225-3p |
| ATP7B | hsa-miR-8061 |
| ATP7B | hsa-miR-6986-5p |
| ATP7B | hsa-miR-3103-3p |
| ATP7B | hsa-miR-6731-3p |
| ATP7B | hsa-miR-504-3p |
| ATP7B | hsa-miR-7238-5p |
| ATP7B | hsa-miR-650d |
| ATP7B | hsa-miR-7094-1-5p |
| ATP7B | hsa-miR-2360 |
| ATP7B | hsa-miR-6528 |
| ATP7B | hsa-miR-7225-5p |
| ATP7B | hsa-miR-8113 |
| ATP7B | hsa-miR-129-5p |
| ATP7B | hsa-miR-7027-3p |
| ATP7B | hsa-miR-203 |
| ATP7B | hsa-miR-3098-3p |
| ATP7B | hsa-miR-145a-3p |
| ATP7B | hsa-miR-3133 |
| ATP7B | hsa-miR-326-5p |
| ATP7B | hsa-miR-6768-3p |
| ATP7B | hsa-miR-2465 |
| ATP7B | hsa-miR-33a-3p |
| ATP7B | hsa-miR-6934-3p |
| ATP7B | hsa-miR-3662 |
| ATP7B | hsa-miR-211-5p |
| ATP7B | hsa-miR-6802-3p |
| ATP7B | hsa-miR-295-3p |
| ATP7B | hsa-miR-3057-3p |
| ATP7B | hsa-miR-143-5p |
| ATP7B | hsa-miR-6874-5p |
| ATP7B | hsa-miR-7252-5p |
| ATP7B | hsa-miR-873-5p |
| ATP7B | hsa-miR-6727-5p |
| ATP7B | hsa-miR-5571-5p |
| ATP7B | hsa-miR-485-5p |
| ATP7B | hsa-miR-329b |
| ATP7B | hsa-miR-711 |
| ATP7B | hsa-miR-653-3p |
| ATP7B | hsa-miR-7350-5p |
| ATP7B | hsa-miR-3974 |
| ATP7B | hsa-miR-3923 |
| ATP7B | hsa-miR-6780a-5p |
| ATP7B | hsa-miR-940 |
| ATP7B | hsa-miR-3470b |
| ATP7B | hsa-miR-545-3p |
| ATP7B | hsa-miR-6790-3p |
| ATP7B | hsa-miR-3202 |
| ATP7B | hsa-miR-7188-5p |
| ATP7B | hsa-miR-876 |
| ATP7B | hsa-miR-2306 |
| ATP7B | hsa-miR-2285z |
| ATP7B | hsa-miR-6852-5p |
| ATP7B | hsa-miR-151a-5p |
| ATP7B | hsa-miR-5093 |
| ATP7B | hsa-miR-7181-3p |
| ATP7B | hsa-miR-873-5p.2 |
| ATP7B | hsa-miR-3057-5p |
| ATP7B | hsa-miR-4654 |
| ATP7B | hsa-miR-221-5p |
| ATP7B | hsa-miR-320a |
| ATP7B | hsa-miR-1324 |
| ATP7B | hsa-miR-7286-3p |
| ATP7B | hsa-miR-424-5p |
| ATP7B | hsa-miR-498 |
| ATP7B | hsa-miR-7173-5p |
| ATP7B | hsa-miR-6354 |
| ATP7B | hsa-miR-580 |
| ATP7B | hsa-miR-665 |
| ATP7B | hsa-miR-6373 |
| ATP7B | hsa-miR-7029-3p |
| ATP7B | hsa-miR-4700-5p |
| ATP7B | hsa-miR-4527 |
| ATP7B | hsa-miR-7047-5p |
| ATP7B | hsa-miR-599-3p |
| ATP7B | hsa-miR-1343-3p |
| ATP7B | hsa-miR-2467-5p |
| ATP7B | hsa-miR-6857-5p |
| ATP7B | hsa-miR-203a-3p.1 |
| ATP7B | hsa-miR-7648-3p |
| ATP7B | hsa-miR-20-5p |
| ATP7B | hsa-miR-6883-5p |
| ATP7B | hsa-miR-5088-3p |
| ATP7B | hsa-miR-920 |
| ATP7B | hsa-miR-7061-5p |
| ATP7B | hsa-miR-3140-5p |
| ATP7B | hsa-miR-4723-3p |
| ATP7B | hsa-miR-133a-5p |
| ATP7B | hsa-miR-539-3p |
| ATP7B | hsa-miR-548x-5p |
| ATP7B | hsa-miR-939-5p |
| ATP7B | hsa-miR-7215-3p |
| ATP7B | hsa-miR-4281 |
| ATP7B | hsa-miR-512-3p |
| ATP7B | hsa-miR-2115-3p |
| ATP7B | hsa-miR-154b |
| ATP7B | hsa-miR-3200-5p |
| ATP7B | hsa-miR-8093 |
| ATP7B | hsa-miR-4520-3p |
| ATP7B | hsa-miR-626 |
| ATP7B | hsa-miR-1906 |
| ATP7B | hsa-miR-7386g-3p |
| ATP7B | hsa-miR-553 |
| ATP7B | hsa-miR-363-3p |
| ATP7B | hsa-miR-194-3p |
| ATP7B | hsa-miR-4695-5p |
| ATP7B | hsa-miR-7063-5p |
| ATP7B | hsa-miR-30b-3p |
| ATP7B | hsa-miR-548ah-3p |
| ATP7B | hsa-miR-6720-3p |
| ATP7B | hsa-miR-7074-3p |
| ATP7B | hsa-miR-4452 |
| ATP7B | hsa-miR-4692 |
| ATP7B | hsa-miR-124-5p |
| ATP7B | hsa-miR-3574 |
| ATP7B | hsa-miR-628-5p |
| ATP7B | hsa-miR-7351-3p |
| ATP7B | hsa-miR-7236-5p |
| ATP7B | hsa-miR-6940-5p |
| ATP7B | hsa-miR-872-3p |
| ATP7B | hsa-miR-6739-3p |
| ATP7B | hsa-miR-2447 |
| ATP7B | hsa-miR-6842-3p |
| ATP7B | hsa-miR-92 |
| ATP7B | hsa-miR-7291-3p |
| ATP7B | hsa-miR-1953 |
| ATP7B | hsa-miR-3150b-3p |
| ATP7B | hsa-miR-548p |
| ATP7B | hsa-miR-7189-5p |
| ATP7B | hsa-miR-7398r-3p |
| ATP7B | hsa-miR-15-5p |
| ATP7B | hsa-miR-1954 |
| ATP7B | hsa-miR-7118-5p |
| ATP7B | hsa-miR-324-3p |
| ATP7B | hsa-miR-618 |
| ATP7B | hsa-miR-7208-5p |
| ATP7B | hsa-miR-4505 |
| ATP7B | hsa-miR-6950-5p |
| ATP7B | hsa-miR-1934-5p |
| ATP7B | hsa-miR-804 |
| ATP7B | hsa-miR-4308 |
| ATP7B | hsa-miR-187-3p |
| ATP7B | hsa-miR-4680-5p |
| ATP7B | hsa-miR-5008-5p |
| ATP7B | hsa-miR-1271-3p |
| ATP7B | hsa-miR-4533 |
| ATP7B | hsa-miR-2364 |
| ATP7B | hsa-miR-6773-5p |
| ATP7B | hsa-miR-4274 |
| ATP7B | hsa-miR-513a |
| ATP7B | hsa-miR-5010-3p |
| ATP7B | hsa-miR-5197-3p |
| ATP7B | hsa-miR-211-5p |
| ATP7B | hsa-miR-6890-3p |
| ATP7B | hsa-miR-942-5p |
| ATP7B | hsa-miR-4329 |
| ATP7B | hsa-miR-206 |
| ATP7B | hsa-miR-6742-3p |
| ATP7B | hsa-miR-20-3p |
| ATP7B | hsa-miR-4267 |
| ATP7B | hsa-miR-211-3p |
| ATP7B | hsa-miR-6990-3p |
| ATP7B | hsa-miR-7113-3p |
| ATP7B | hsa-miR-4734 |
| ATP7B | hsa-miR-124-3p.2 |
| ATP7B | hsa-miR-6900-5p |
| ATP7B | hsa-miR-6735-5p |
| ATP7B | hsa-miR-4330 |
| ATP7B | hsa-miR-7082-3p |
| ATP7B | hsa-miR-497-5p |
| ATP7B | hsa-miR-4709-3p |
| ATP7B | hsa-miR-1281 |
| ATP7B | hsa-miR-2339 |
| ATP7B | hsa-miR-4638-3p |
| ATP7B | hsa-miR-200ab-5p |
| ATP7B | hsa-miR-5580-5p |
| ATP7B | hsa-miR-766-3p |
| ATP7B | hsa-miR-1249-3p |
| ATP7B | hsa-miR-5121 |
| ATP7B | hsa-miR-6898-5p |
| ATP7B | hsa-miR-7398n-3p |
| ATP7B | hsa-miR-449c-3p |
| ATP7B | hsa-miR-3128 |
| ATP7B | hsa-miR-467-3p |
| ATP7B | hsa-miR-27-3p |
| ATP7B | hsa-miR-4451 |
| ATP7B | hsa-miR-196a-3p |
| ATP7B | hsa-miR-3921 |
| ATP7B | hsa-miR-1952 |
| ATP7B | hsa-miR-6821-3p |
| ATP7B | hsa-miR-5586-5p |
| ATP7B | hsa-miR-6894-5p |
| ATP7B | hsa-miR-7665-3p |
| ATP7B | hsa-miR-3058-3p |
| ATP7B | hsa-miR-7007-5p |
| ATP7B | hsa-miR-7398b-3p |
| ATP7B | hsa-miR-3675-5p |
| ATP7B | hsa-miR-208-5p |
| ATP7B | hsa-miR-7035-5p |
| ATP7B | hsa-miR-759 |
| ATP7B | hsa-miR-9a-3p |
| ATP7B | hsa-miR-6131 |
| ATP7B | hsa-miR-6787-3p |
| ATP7B | hsa-miR-4764-5p |
| ATP7B | hsa-miR-4749-3p |
| ATP7B | hsa-miR-6846-5p |
| ATP7B | hsa-miR-4272 |
| ATP7B | hsa-miR-4277 |
| ATP7B | hsa-miR-361-5p |
| ATP7B | hsa-miR-6503-5p |
| ATP7B | hsa-miR-7016-3p |
| ATP7B | hsa-miR-7111-3p |
| ATP7B | hsa-miR-700-5p |
| ATP7B | hsa-miR-6971-3p |
| ATP7B | hsa-miR-6875-3p |
| ATP7B | hsa-miR-6321 |
| ATP7B | hsa-miR-4724-5p |
| ATP7B | hsa-miR-101-3p |
| ATP7B | hsa-miR-6828-5p |
| ATP7B | hsa-miR-4673 |
| ATP7B | hsa-miR-7218-5p |
| ATP7B | hsa-miR-3596 |
| ATP7B | hsa-miR-1934-3p |
| ATP7B | hsa-miR-132-3p |
| ATP7B | hsa-miR-580-3p |
| ATP7B | hsa-miR-362-3p |
| ATP7B | hsa-miR-6962-5p |
| ATP7B | hsa-miR-423-5p |
| ATP7B | hsa-miR-99-3p |
| ATP7B | hsa-miR-218-5p |
| ATP7B | hsa-miR-122 |
| ATP7B | hsa-miR-548h-3p |
| ATP7B | hsa-miR-6848-5p |
| ATP7B | hsa-miR-181-5p |
| ATP7B | hsa-miR-669n |
| ATP7B | hsa-miR-301-5p |
| ATP7B | hsa-miR-6797-3p |
| ATP7B | hsa-miR-6944-5p |
| ATP7B | hsa-miR-541-5p |
| ATP7B | hsa-miR-1296-3p |
| ATP7B | hsa-miR-1285-3p |
| ATP7B | hsa-miR-7216-5p |
| ATP7B | hsa-miR-4524b-3p |
| ATP7B | hsa-miR-29a-1-5p |
| ATP7B | hsa-miR-7085-3p |
| ATP7B | hsa-miR-873a-5p.1 |
| ATP7B | hsa-miR-8827 |
| ATP7B | hsa-miR-6377 |
| ATP7B | hsa-miR-5581-3p |
| ATP7B | hsa-miR-2902 |
| ATP7B | hsa-miR-3654 |
| ATP7B | hsa-miR-1306-5p |
| ATP7B | hsa-miR-6730-3p |
| ATP7B | hsa-miR-4732-3p |
| ATP7B | hsa-miR-6366 |
| ATP7B | hsa-miR-4731-5p |
| ATP7B | hsa-miR-548am-3p |
| ATP7B | hsa-miR-518 |
| ATP7B | hsa-miR-6769b-5p |
| ATP7B | hsa-miR-133a-3p.1 |
| ATP7B | hsa-miR-1207 |
| ATP7B | hsa-miR-7974 |
| ATP7B | hsa-miR-3070-3p |
| ATP7B | hsa-miR-7398-3p |
| ATP7B | hsa-miR-2454-3p |
| ATP7B | hsa-miR-6947-3p |
| ATP7B | hsa-miR-1904 |
| ATP7B | hsa-miR-3145-5p |
| ATP7B | hsa-miR-434-5p |
| ATP7B | hsa-miR-6964-3p |
| ATP7B | hsa-miR-1197 |
| ATP7B | hsa-miR-7026-3p |
| ATP7B | hsa-miR-17-5p |
| ATP7B | hsa-miR-7256-5p |
| ATP7B | hsa-miR-5619-3p |
| ATP7B | hsa-miR-362-5p |
| ATP7B | hsa-miR-6878-5p |
| ATP7B | hsa-miR-7660-3p |
| ATP7B | hsa-miR-30c-3p |
| ATP7B | hsa-miR-6722-3p |
| ATP7B | hsa-miR-7388b-5p |
| ATP7B | hsa-miR-1238-5p |
| ATP7B | hsa-miR-3594-5p |
| ATP7B | hsa-miR-491-3p |
| ATP7B | hsa-miR-2449 |
| ATP7B | hsa-miR-519-5p |
| ATP7B | hsa-miR-7184-5p |
| ATP7B | hsa-miR-1909-3p |
| ATP7B | hsa-miR-892b |
| ATP7B | hsa-miR-690 |
| ATP7B | hsa-miR-3200-3p |
| ATP7B | hsa-miR-1912-3p |
| ATP7B | hsa-miR-466f-3p |
| ATP7B | hsa-miR-130-5p |
| ATP7B | hsa-miR-4800-5p |
| ATP7B | hsa-miR-705 |
| ATP7B | hsa-miR-18-5p |
| ATP7B | hsa-miR-548aef |
| ATP7B | hsa-miR-7218-3p |
| ATP7B | hsa-miR-370-5p |
| ATP7B | hsa-miR-2326 |
| ATP7B | hsa-miR-1193-3p |
| ATP7B | hsa-miR-6781-3p |
| ATP7B | hsa-miR-34 |
| ATP7B | hsa-miR-6512-5p |
| ATP7B | hsa-miR-548i-5p |
| ATP7B | hsa-miR-4499 |
| ATP7B | hsa-miR-7256-3p |
| ATP7B | hsa-miR-770-3p |
| ATP7B | hsa-miR-6945-3p |
| ATP7B | hsa-miR-490-5p |
| ATP7B | hsa-miR-3194-3p |
| ATP7B | hsa-miR-7045-3p |
| ATP7B | hsa-miR-2285af |
| ATP7B | hsa-miR-6503-3p |
| ATP7B | hsa-miR-217-5p |
| ATP7B | hsa-miR-6523a |
| ATP7B | hsa-miR-329-3p |
| ATP7B | hsa-miR-376-3p |
| ATP7B | hsa-miR-2114-5p |
| ATP7B | hsa-miR-4728-3p |
| ATP7B | hsa-miR-659-5p |
| ATP7B | hsa-miR-7354-3p |
| ATP7B | hsa-miR-1960 |
| ATP7B | hsa-miR-7316-3p |
| ATP7B | hsa-miR-577-5p |
| ATP7B | hsa-miR-7170-3p |
| ATP7B | hsa-miR-3065-5p |
| ATP7B | hsa-miR-219a-1-3p |
| ATP7B | hsa-miR-571 |
| ATP7B | hsa-miR-4779 |
| ATP7B | hsa-miR-758-5p |
| ATP7B | hsa-miR-3978 |
| ATP7B | hsa-miR-760-5p |
| ATP7B | hsa-miR-3130-3p |
| ATP7B | hsa-miR-7301-3p |
| ATP7B | hsa-miR-6843-3p |
| ATP7B | hsa-miR-8086 |
| ATP7B | hsa-miR-133b |
| ATP7B | hsa-miR-1207-5p |
| ATP7B | hsa-miR-1228-3p |
| ATP7B | hsa-miR-5007-5p |
| ATP7B | hsa-miR-2451 |
| ATP7B | hsa-miR-1284 |
| ATP7B | hsa-miR-3189-3p |
| ATP7B | hsa-miR-548ae-3p |
| ATP7B | hsa-miR-582-3p |
| ATP7B | hsa-miR-6765-3p |
| ATP7B | hsa-miR-3077-5p |
| ATP7B | hsa-miR-576-5p |
| ATP7B | hsa-miR-4666b |
| ATP7B | hsa-miR-4502 |
| ATP7B | hsa-miR-7398m-3p |
| ATP7B | hsa-miR-10b-3p |
| ATP7B | hsa-miR-2467-3p |
| ATP7B | hsa-miR-6752-3p |
| ATP7B | hsa-miR-548ao-5p |
| ATP7B | hsa-miR-144-3p |
| ATP7B | hsa-miR-7247-5p |
| ATP7B | hsa-miR-133-3p |
| ATP7B | hsa-miR-7183-5p |
| ATP7B | hsa-miR-1322 |
| ATP7B | hsa-miR-548 |
| ATP7B | hsa-miR-486-3p |
| ATP7B | hsa-miR-373-3p |
| ATP7B | hsa-miR-1294 |
| ATP7B | hsa-miR-5617-3p |
| ATP7B | hsa-miR-687 |
| ATP7B | hsa-miR-7671-5p |
| ATP7B | hsa-miR-183-5p |
| ATP7B | hsa-miR-6781-5p |
| ATP7B | hsa-miR-6788-5p |
| ATP7B | hsa-miR-3552 |
| ATP7B | hsa-miR-3547 |
| ATP7B | hsa-miR-532-3p |
| ATP7B | hsa-miR-379-3p |
| ATP7B | hsa-miR-6770-5p |
| ATP7B | hsa-miR-7091-3p |
| ATP7B | hsa-miR-588 |
| ATP7B | hsa-miR-4705 |
| ATP7B | hsa-miR-657 |
| ATP7B | hsa-miR-2438 |
| ATP7B | hsa-miR-2315 |
| ATP7B | hsa-miR-2397-3p |
| ATP7B | hsa-miR-17-3p |
| ATP7B | hsa-miR-6927-3p |
| ATP7B | hsa-miR-885 |
| ATP7B | hsa-miR-5114 |
| ATP7B | hsa-miR-16-5p |
| ATP7B | hsa-miR-125a-3p |
| ATP7B | hsa-miR-3099 |
| ATP7B | hsa-miR-322-3p |
| ATP7B | hsa-miR-140-3p.1 |
| ATP7B | hsa-miR-548c-3p |
| ATP7B | hsa-miR-16-5p |
| ATP7B | hsa-miR-1777 |
| ATP7B | hsa-miR-30a-3p |
| ATP7B | hsa-miR-350-3p |
| ATP7B | hsa-miR-7030-5p |
| ATP7B | hsa-miR-27 |
| ATP7B | hsa-miR-215-3p |
| ATP7B | hsa-miR-3059-5p |
| ATP7B | hsa-miR-93-3p |
| ATP7B | hsa-miR-604 |
| ATP7B | hsa-miR-5011-3p |
| ATP7B | hsa-miR-1266-5p |
| ATP7B | hsa-miR-2472 |
| ATP7B | hsa-miR-199b-2-5p |
| ATP7B | hsa-miR-23a-5p |
| ATP7B | hsa-miR-6800-5p |
| ATP7B | hsa-miR-3916 |
| ATP7B | hsa-miR-186-5p |
| ATP7B | hsa-miR-6979-5p |
| ATP7B | hsa-miR-7706 |
| ATP7B | hsa-miR-674-5p |
| ATP7B | hsa-miR-493-5p |
| ATP7B | hsa-miR-196-5p |
| ATP7B | hsa-miR-6768-5p |
| ATP7B | hsa-miR-7166-5p |
| ATP7B | hsa-miR-3620-5p |
| ATP7B | hsa-miR-3098-5p |
| ATP7B | hsa-miR-378 |
| ATP7B | hsa-miR-29-5p |
| ATP7B | hsa-miR-5196-3p |
| ATP7B | hsa-miR-542-3p |
| ATP7B | hsa-miR-6769a-3p |
| ATP7B | hsa-miR-298 |
| ATP7B | hsa-miR-6325 |
| ATP7B | hsa-miR-376b-3p |
| ATP7B | hsa-miR-30e-3p |
| ATP7B | hsa-miR-3201 |
| ATP7B | hsa-miR-154a |
| ATP7B | hsa-miR-301-3p |
| ATP7B | hsa-miR-344g-3p |
| ATP7B | hsa-miR-204-5p |
| ATP7B | hsa-miR-1251 |
| ATP7B | hsa-miR-202-3p |
| ATP7B | hsa-miR-141-5p |
| ATP7B | hsa-miR-520 |
| ATP7B | hsa-miR-7365-3p |
| ATP7B | hsa-miR-561-5p |
| ATP7B | hsa-miR-4433a-5p |
| ATP7B | hsa-miR-4303 |
| ATP7B | hsa-miR-466g |
| ATP7B | hsa-miR-697 |
| ATP7B | hsa-miR-6940-3p |
| ATP7B | hsa-miR-6848-3p |
| ATP7B | hsa-miR-2477 |
| ATP7B | hsa-miR-16-3p |
| ATP7B | hsa-miR-7313-5p |
| ATP7B | hsa-miR-6793-3p |
| ATP7B | hsa-miR-2485 |
| ATP7B | hsa-miR-7214-3p |
| ATP7B | hsa-miR-2355-5p |
| ATP7B | hsa-miR-5695 |
| ATP7B | hsa-miR-344b-3p |
| ATP7B | hsa-miR-370-3p |
| ATP7B | hsa-miR-344-5p |
| ATP7B | hsa-miR-6534 |
| ATP7B | hsa-miR-874-3p |
| ATP7B | hsa-miR-770-5p |
| ATP7B | hsa-miR-2486-5p |
| ATP7B | hsa-miR-518c-5p |
| ATP7B | hsa-miR-4641 |
| ATP7B | hsa-miR-7031-5p |
| ATP7B | hsa-miR-1540 |
| ATP7B | hsa-miR-335-3p |
| ATP7B | hsa-miR-372 |
| ATP7B | hsa-miR-26b-3p |
| ATP7B | hsa-miR-375 |
| ATP7B | hsa-miR-376a-1-5p |
| ATP7B | hsa-miR-3076-5p |
| ATP7B | hsa-miR-296-5p |
| ATP7B | hsa-miR-4690-3p |
| ATP7B | hsa-miR-5621-5p |
| ATP7B | hsa-miR-6836-5p |
| ATP7B | hsa-miR-6811-3p |
| ATP7B | hsa-miR-1973 |
| ATP7B | hsa-miR-5129-5p |
| ATP7B | hsa-miR-7048-5p |
| ATP7B | hsa-miR-3473f |
| ATP7B | hsa-miR-322-5p |
| ATP7B | hsa-miR-92-3p |
| ATP7B | hsa-miR-6320 |
| ATP7B | hsa-miR-4689 |
| ATP7B | hsa-miR-513a-5p |
| ATP7B | hsa-miR-9-3-3p |
| ATP7B | hsa-miR-372-3p |
| ATP7B | hsa-miR-34c-3p |
| ATP7B | hsa-miR-3183 |
| ATP7B | hsa-miR-2376 |
| ATP7B | hsa-miR-24-3p |
| ATP7B | hsa-miR-3547-5p |
| ATP7B | hsa-miR-422 |
| ATP7B | hsa-miR-3089-5p |
| ATP7B | hsa-miR-6926-5p |
| ATP7B | hsa-miR-24-3p |
| ATP7B | hsa-miR-188-3p |
| ATP7B | hsa-miR-3179 |
| ATP7B | hsa-miR-551b-5p |
| ATP7B | hsa-miR-624-5p |
| ATP7B | hsa-miR-135a-1-3p |
| ATP7B | hsa-miR-212-3p |
| ATP7B | hsa-miR-8832 |
| ATP7B | hsa-miR-544-3p |
| ATP7B | hsa-miR-6749-3p |
| ATP7B | hsa-miR-492 |
| ATP7B | hsa-miR-6340 |
| ATP7B | hsa-miR-208a-3p |
| ATP7B | hsa-miR-7398d-3p |
| ATP7B | hsa-miR-1827 |
| ATP7B | hsa-miR-4731-3p |
| ATP7B | hsa-miR-7079-5p |
| ATP7B | hsa-miR-500-3p |
| ATP7B | hsa-miR-7-5p |
| ATP7B | hsa-miR-548ar-3p |
| ATP7B | hsa-miR-3692-5p |
| ATP7B | hsa-miR-2396 |
| ATP7B | hsa-miR-6529 |
| ATP7B | hsa-miR-6971-5p |
| ATP7B | hsa-miR-7352-3p |
| ATP7B | hsa-miR-1-3p |
| ATP7B | hsa-miR-3918 |
| ATP7B | hsa-miR-3070-2-3p |
| ATP7B | hsa-miR-6799-5p |
| ATP7B | hsa-miR-4717-3p |
| ATP7B | hsa-miR-421 |
| ATP7B | hsa-miR-6914-5p |
| ATP7B | hsa-miR-6823-5p |
| ATP7B | hsa-miR-7327-5p |
| ATP7B | hsa-miR-491-5p |
| ATP7B | hsa-miR-222-5p |
| ATP7B | hsa-miR-7682-3p |
| ATP7B | hsa-miR-92a-2-5p |
| ATP7B | hsa-miR-6974-3p |
| ATP7B | hsa-miR-3064-5p |
| ATP7B | hsa-miR-2290 |
| ATP7B | hsa-miR-7349-3p |
| ATP7B | hsa-miR-432-5p |
| ATP7B | hsa-miR-2313-3p |
| ATP7B | hsa-miR-149-3p |
| ATP7B | hsa-miR-133a-3p.2 |
| ATP7B | hsa-miR-411-3p |
| ATP7B | hsa-miR-6332 |
| ATP7B | hsa-miR-370 |
| ATP7B | hsa-miR-5694 |
| ATP7B | hsa-miR-7656-3p |
| ATP7B | hsa-miR-512 |
| ATP7B | hsa-miR-510-3p |
| ATP7B | hsa-miR-7286-5p |
| ATP7B | hsa-miR-2433 |
| ATP7B | hsa-miR-7030-3p |
| ATP7B | hsa-miR-10-5p |
| ATP7B | hsa-miR-548x-3p |
| ATP7B | hsa-miR-34-5p |
| ATP7B | hsa-miR-5680 |
| ATP7B | hsa-miR-561-3p |
| ATP7B | hsa-miR-3173-3p |
| ATP7B | hsa-miR-7241-5p |
| ATP7B | hsa-miR-6995-3p |
| ATP7B | hsa-miR-520-3p |
| ATP7B | hsa-miR-1184 |
| ATP7B | hsa-miR-1982-5p |
| ATP7B | hsa-miR-873-5p |
| ATP7B | hsa-miR-6903-5p |
| ATP7B | hsa-miR-2117 |
| ATP7B | hsa-miR-185-3p |
| ATP7B | hsa-miR-185-5p |
| ATP7B | hsa-miR-4650-3p |
| ATP7B | hsa-miR-6952-5p |
| ATP7B | hsa-miR-8845 |
| ATP7B | hsa-miR-2284z |
| ATP7B | hsa-miR-6525 |
| ATP7B | hsa-miR-543 |
| ATP7B | hsa-miR-518g-3p |
| ATP7B | hsa-miR-2305 |
| ATP7B | hsa-miR-4286 |
| ATP7B | hsa-miR-2450a |
| ATP7B | hsa-miR-4498 |
| ATP7B | hsa-miR-8085 |
| ATP7B | hsa-miR-148-3p |
| ATP7B | hsa-miR-192-3p |
| ATP7B | hsa-miR-25-3p |
| ATP7B | hsa-miR-1276 |
| ATP7B | hsa-miR-6078 |
| ATP7B | hsa-miR-7398l-3p |
| ATP7B | hsa-miR-6918-3p |
| ATP7B | hsa-miR-589-5p |
| ATP7B | hsa-miR-3163 |
| ATP7B | hsa-miR-3544-5p |
| ATP7B | hsa-miR-6978-5p |
| ATP7B | hsa-miR-300-5p |
| ATP7B | hsa-miR-3120-3p |
| ATP7B | hsa-miR-5107-5p |
| CDKN2A | hsa-miR-485 |
| CDKN2A | hsa-miR-4719 |
| CDKN2A | hsa-miR-4727-5p |
| CDKN2A | hsa-miR-6756-5p |
| CDKN2A | hsa-miR-660-5p |
| CDKN2A | hsa-miR-4799-5p |
| CDKN2A | hsa-miR-5001-3p |
| CDKN2A | hsa-miR-4765 |
| CDKN2A | hsa-miR-380 |
| CDKN2A | hsa-miR-4302 |
| CDKN2A | hsa-miR-449c-3p |
| CDKN2A | hsa-miR-500a-3p |
| CDKN2A | hsa-miR-566 |
| CDKN2A | hsa-miR-3194-5p |
| CDKN2A | hsa-miR-6769a-5p |
| CDKN2A | hsa-miR-373-3p |
| CDKN2A | hsa-miR-138-2-3p |
| CDKN2A | hsa-miR-548e-5p |
| CDKN2A | hsa-miR-654-5p |
| CDKN2A | hsa-miR-34 |
| CDKN2A | hsa-miR-944 |
| CDKN2A | hsa-miR-22-5p |
| CDKN2A | hsa-miR-3141 |
| CDKN2A | hsa-miR-4446-5p |
| CDKN2A | hsa-miR-507 |
| CDKN2A | hsa-miR-4268 |
| CDKN2A | hsa-miR-541-3p |
| CDKN2A | hsa-miR-764 |
| CDKN2A | hsa-miR-6877-3p |
| CDKN2A | hsa-miR-663b |
| CDKN2A | hsa-miR-4698 |
| CDKN2A | hsa-miR-125 |
| CDKN2A | hsa-miR-6796-3p |
| CDKN2A | hsa-miR-7168-5p |
| CDKN2A | hsa-miR-8836 |
| CDKN2A | hsa-miR-6858-3p |
| CDKN2A | hsa-miR-4447 |
| CDKN2A | hsa-miR-6766-5p |
| CDKN2A | hsa-miR-4699-5p |
| CDKN2A | hsa-miR-6068 |
| CDKN2A | hsa-miR-380-3p |
| CDKN2A | hsa-miR-6889-5p |
| CDKN2A | hsa-miR-5010-5p |
| CDKN2A | hsa-miR-2899 |
| CDKN2A | hsa-miR-2429 |
| CDKN2A | hsa-miR-7108-5p |
| CDKN2A | hsa-miR-4513 |
| CDKN2A | hsa-miR-5088-3p |
| CDKN2A | hsa-miR-1203 |
| CDKN2A | hsa-miR-1236-5p |
| CDKN2A | hsa-miR-7865 |
| CDKN2A | hsa-miR-519-3p |
| CDKN2A | hsa-miR-7154-5p |
| CDKN2A | hsa-miR-2385-3p |
| CDKN2A | hsa-miR-575 |
| CDKN2A | hsa-miR-802 |
| CDKN2A | hsa-miR-1842 |
| CDKN2A | hsa-miR-1268a |
| CDKN2A | hsa-miR-4439 |
| CDKN2A | hsa-miR-2114-3p |
| CDKN2A | hsa-miR-152-5p |
| CDKN2A | hsa-miR-576-3p |
| CDKN2A | hsa-miR-4727-3p |
| CDKN2A | hsa-miR-6808-5p |
| CDKN2A | hsa-miR-149-3p |
| CDKN2A | hsa-miR-8884 |
| CDKN2A | hsa-miR-486-3p |
| CDKN2A | hsa-miR-548q |
| CDKN2A | hsa-miR-518-3p |
| CDKN2A | hsa-miR-623 |
| CDKN2A | hsa-miR-508-5p |
| CDKN2A | hsa-miR-933 |
| CDKN2A | hsa-miR-218-1-3p |
| CDKN2A | hsa-miR-5196-5p |
| CDKN2A | hsa-miR-302-3p |
| CDKN2A | hsa-miR-211-5p |
| CDKN2A | hsa-miR-1178-3p |
| CDKN2A | hsa-miR-3940-5p |
| CDKN2A | hsa-miR-6769b-5p |
| CDKN2A | hsa-miR-1307-3p |
| CDKN2A | hsa-miR-4740-3p |
| CDKN2A | hsa-miR-7208-3p |
| CDKN2A | hsa-miR-659-3p |
| CDKN2A | hsa-miR-8055 |
| CDKN2A | hsa-miR-3578 |
| CDKN2A | hsa-miR-2306 |
| CDKN2A | hsa-miR-3145-5p |
| CDKN2A | hsa-miR-196b-3p |
| CDKN2A | hsa-miR-604 |
| CDKN2A | hsa-miR-1291 |
| CDKN2A | hsa-miR-372-3p |
| CDKN2A | hsa-miR-362-5p |
| CDKN2A | hsa-miR-2902 |
| CDKN2A | hsa-miR-2367-3p |
| CDKN2A | hsa-miR-8842 |
| CDKN2A | hsa-miR-4274 |
| CDKN2A | hsa-miR-5697-3p |
| CDKN2A | hsa-miR-520-3p |
| CDKN2A | hsa-miR-338-5p |
| CDKN2A | hsa-miR-658 |
| CDKN2A | hsa-miR-1909-5p |
| CDKN2A | hsa-miR-149-5p |
| CDKN2A | hsa-miR-5006-5p |
| CDKN2A | hsa-miR-4263 |
| CDKN2A | hsa-miR-6769b-3p |
| CDKN2A | hsa-miR-5011-5p |
| CDKN2A | hsa-miR-4694-3p |
| CDKN2A | hsa-miR-2285y |
| CDKN2A | hsa-miR-362-5p |
| CDKN2A | hsa-miR-507b |
| CDKN2A | hsa-miR-6501-3p |
| CDKN2A | hsa-miR-125-5p |
| CDKN2A | hsa-miR-2284x |
| CDKN2A | hsa-miR-7186-5p |
| CDKN2A | hsa-miR-548an |
| CDKN2A | hsa-miR-4271 |
| CDKN2A | hsa-miR-6804-3p |
| CDKN2A | hsa-miR-2114-5p |
| CDKN2A | hsa-miR-1908-5p |
| CDKN2A | hsa-miR-96-5p |
| CDKN2A | hsa-miR-4755-5p |
| CDKN2A | hsa-miR-3682-3p |
| CDKN2A | hsa-miR-450b-5p |
| CDKN2A | hsa-miR-27b-5p |
| CDKN2A | hsa-miR-410-3p |
| CDKN2A | hsa-miR-134 |
| CDKN2A | hsa-miR-4723-5p |
| CDKN2A | hsa-miR-4673 |
| CDKN2A | hsa-miR-6734-3p |
| CDKN2A | hsa-miR-597-3p |
| CDKN2A | hsa-miR-5698 |
| CDKN2A | hsa-miR-4737 |
| CDKN2A | hsa-miR-1199-5p |
| CDKN2A | hsa-miR-1296 |
| CDKN2A | hsa-miR-218-2-3p |
| CDKN2A | hsa-miR-6793-5p |
| CDKN2A | hsa-miR-8888 |
| CDKN2A | hsa-miR-4634 |
| CDKN2A | hsa-miR-1388-3p |
| CDKN2A | hsa-miR-510-3p |
| CDKN2A | hsa-miR-2346 |
| CDKN2A | hsa-miR-2285l |
| CDKN2A | hsa-miR-488-3p |
| CDKN2A | hsa-miR-2471-3p |
| CDKN2A | hsa-miR-335-5p |
| CDKN2A | hsa-miR-153-5p |
| CDKN2A | hsa-miR-2909 |
| CDKN2A | hsa-miR-6817-3p |
| CDKN2A | hsa-miR-3191-5p |
| CDKN2A | hsa-miR-6777-5p |
| CDKN2A | hsa-miR-7192-3p |
| CDKN2A | hsa-miR-4725-3p |
| CDKN2A | hsa-miR-7111-5p |
| CDKN2A | hsa-miR-204-5p |
| CDKN2A | hsa-miR-1910-3p |
| CDKN2A | hsa-miR-514a-3p |
| CDKN2A | hsa-miR-4502 |
| CDKN2A | hsa-miR-6511a-5p |
| CDKN2A | hsa-miR-3136-5p |
| CDKN2A | hsa-miR-4645-5p |
| CDKN2A | hsa-miR-300 |
| CDKN2A | hsa-miR-194-3p |
| CDKN2A | hsa-miR-29b-1-5p |
| CDKN2A | hsa-miR-1275 |
| CDKN2A | hsa-miR-1277-5p |
| CDKN2A | hsa-miR-4519 |
| CDKN2A | hsa-miR-3662 |
| CDKN2A | hsa-miR-4536-3p |
| CDKN2A | hsa-miR-4747-5p |
| CDKN2A | hsa-miR-2370-5p |
| CDKN2A | hsa-miR-134-5p |
| CDKN2A | hsa-miR-4466 |
| CDKN2A | hsa-miR-6817-5p |
| CDKN2A | hsa-miR-3183 |
| CDKN2A | hsa-miR-30e |
| CDKN2A | hsa-miR-4769-3p |
| CDKN2A | hsa-miR-18 |
| CDKN2A | hsa-miR-4786-3p |
| CDKN2A | hsa-miR-4472 |
| CDKN2A | hsa-miR-3680-3p |
| CDKN2A | hsa-miR-4723-3p |
| CDKN2A | hsa-miR-625-5p |
| CDKN2A | hsa-miR-2421 |
| CDKN2A | hsa-miR-1911-5p |
| CDKN2A | hsa-miR-657 |
| CDKN2A | hsa-miR-6765-3p |
| CDKN2A | hsa-miR-141-3p |
| CDKN2A | hsa-miR-2402 |
| CDKN2A | hsa-miR-8060 |
| CDKN2A | hsa-miR-518c-5p |
| CDKN2A | hsa-miR-204-5p |
| CDKN2A | hsa-miR-6857-3p |
| CDKN2A | hsa-miR-1271-5p |
| CDKN2A | hsa-miR-1307-3p |
| CDKN2A | hsa-miR-5680 |
| CDKN2A | hsa-miR-6507-5p |
| CDKN2A | hsa-miR-6855-3p |
| CDKN2A | hsa-miR-485-5p |
| CDKN2A | hsa-miR-219a-5p |
| CDKN2A | hsa-miR-6893-5p |
| CDKN2A | hsa-miR-182-3p |
| CDKN2A | hsa-miR-6819-3p |
| CDKN2A | hsa-miR-548au-3p |
| CDKN2A | hsa-miR-4651 |
| CDKN2A | hsa-miR-6780b-5p |
| CDKN2A | hsa-miR-374 |
| CDKN2A | hsa-miR-9-5p |
| CDKN2A | hsa-miR-6729-3p |
| CDKN2A | hsa-miR-670-3p |
| CDKN2A | hsa-miR-500-3p |
| CDKN2A | hsa-miR-518 |
| CDKN2A | hsa-miR-335-5p |
| CDKN2A | hsa-miR-663a |
| CDKN2A | hsa-miR-542-5p |
| CDKN2A | hsa-miR-576-5p |
| CDKN2A | hsa-miR-576-5p |
| CDKN2A | hsa-miR-3136-5p |
| CDKN2A | hsa-miR-8808 |
| CDKN2A | hsa-miR-6775-3p |
| CDKN2A | hsa-miR-1180-3p |
| CDKN2A | hsa-miR-3910 |
| CDKN2A | hsa-miR-6870-5p |
| CDKN2A | hsa-miR-513b-5p |
| CDKN2A | hsa-miR-3646 |
| CDKN2A | hsa-miR-3155a |
| CDKN2A | hsa-miR-449a |
| CDKN2A | hsa-miR-513 |
| CDKN2A | hsa-miR-3163 |
| CDKN2A | hsa-miR-146b-3p |
| CDKN2A | hsa-miR-4755-3p |
| CDKN2A | hsa-miR-365-3p |
| CDKN2A | hsa-miR-452-3p |
| CDKN2A | hsa-miR-598-5p |
| CDKN2A | hsa-miR-326 |
| CDKN2A | hsa-miR-4507 |
| CDKN2A | hsa-miR-1296-5p |
| CDKN2A | hsa-miR-1228-5p |
| CDKN2A | hsa-miR-522-3p |
| CDKN2A | hsa-miR-2681-5p |
| CDKN2A | hsa-miR-4330 |
| CDKN2A | hsa-miR-2285ad |
| CDKN2A | hsa-miR-1252-3p |
| CDKN2A | hsa-miR-5006-3p |
| CDKN2A | hsa-miR-522-3p |
| CDKN2A | hsa-miR-8834a |
| CDKN2A | hsa-miR-381-5p |
| CDKN2A | hsa-miR-2285 |
| CDKN2A | hsa-miR-5000-5p |
| CDKN2A | hsa-miR-211-5p |
| CDKN2A | hsa-miR-4781-5p |
| CDKN2A | hsa-miR-6738-3p |
| CDKN2A | hsa-miR-675-5p |
| CDKN2A | hsa-miR-4676-5p |
| CDKN2A | hsa-miR-5000-3p |
| CDKN2A | hsa-miR-24-5p |
| CDKN2A | hsa-miR-6764-3p |
| CDKN2A | hsa-miR-6824-3p |
| CDKN2A | hsa-miR-874 |
| CDKN2A | hsa-miR-6499-3p |
| CDKN2A | hsa-miR-2425-5p |
| CDKN2A | hsa-miR-1243-5p |
| CDKN2A | hsa-miR-940 |
| CDKN2A | hsa-miR-3714 |
| CDKN2A | hsa-miR-425-5p |
| CDKN2A | hsa-miR-605-3p |
| CDKN2A | hsa-miR-763 |
| CDKN2A | hsa-miR-381-3p |
| CDKN2A | hsa-miR-561-3p |
| CDKN2A | hsa-miR-128 |
| CDKN2A | hsa-miR-483-3p.2 |
| CDKN2A | hsa-miR-3164 |
| CDKN2A | hsa-miR-760 |
| CDKN2A | hsa-miR-128-3p |
| CDKN2A | hsa-miR-621 |
| CDKN2A | hsa-miR-1434-3p |
| CDKN2A | hsa-miR-6883-3p |
| CDKN2A | hsa-miR-557 |
| CDKN2A | hsa-miR-92a-5p |
| CDKN2A | hsa-miR-2355-5p |
| CDKN2A | hsa-miR-1178-3p |
| CDKN2A | hsa-miR-2467-5p |
| CDKN2A | hsa-miR-1286 |
| CDKN2A | hsa-miR-4474-3p |
| CDKN2A | hsa-miR-369-3p |
| CDKN2A | hsa-miR-1296-3p |
| CDKN2A | hsa-miR-24-3p |
| CDKN2A | hsa-miR-7862 |
| CDKN2A | hsa-miR-324-5p |
| CDKN2A | hsa-miR-6851-3p |
| CDKN2A | hsa-miR-8065 |
| CDKN2A | hsa-miR-212-3p |
| CDKN2A | hsa-miR-186-3p |
| CDKN2A | hsa-miR-938 |
| CDKN2A | hsa-miR-7859 |
| CDKN2A | hsa-miR-570-3p |
| CDKN2A | hsa-miR-1911-3p |
| CDKN2A | hsa-miR-7207-5p |
| CDKN2A | hsa-miR-484 |
| CDKN2A | hsa-miR-7183-3p |
| CDKN2A | hsa-miR-6132 |
| CDKN2A | hsa-miR-1257 |
| CDKN2A | hsa-miR-9-5p |
| CDKN2A | hsa-miR-608 |
| CDKN2A | hsa-miR-298 |
| CDKN2A | hsa-miR-659-5p |
| CDKN2A | hsa-miR-6807-5p |
| CDKN2A | hsa-miR-365a-3p |
| CDKN2A | hsa-miR-410 |
| CDKN2A | hsa-miR-668-5p |
| CDKN2A | hsa-miR-17-3p |
| CDKN2A | hsa-miR-570-3p |
| CDKN2A | hsa-miR-2417 |
| CDKN2A | hsa-miR-4665-5p |
| CDKN2A | hsa-miR-670 |
| CDKN2A | hsa-miR-7202-3p |
| CDKN2A | hsa-miR-129-2-3p |
| CDKN2A | hsa-miR-328-3p |
| CDKN2A | hsa-miR-1268b |
| CDKN2A | hsa-miR-500 |
| DBT | hsa-miR-8908c |
| DBT | hsa-miR-7313-3p |
| DBT | hsa-miR-5584-5p |
| DBT | hsa-miR-125a-3p |
| DBT | hsa-miR-1299 |
| DBT | hsa-miR-367-5p |
| DBT | hsa-miR-5580-3p |
| DBT | hsa-miR-130-3p |
| DBT | hsa-miR-2284v |
| DBT | hsa-miR-5703 |
| DBT | hsa-miR-6907-3p |
| DBT | hsa-miR-6772-5p |
| DBT | hsa-miR-92a-3p |
| DBT | hsa-miR-548ay-5p |
| DBT | hsa-miR-331-3p |
| DBT | hsa-miR-664a-3p |
| DBT | hsa-miR-522-3p |
| DBT | hsa-miR-2457 |
| DBT | hsa-miR-4436b-3p |
| DBT | hsa-miR-193 |
| DBT | hsa-miR-548aq-5p |
| DBT | hsa-miR-1199-5p |
| DBT | hsa-miR-7191-5p |
| DBT | hsa-miR-509-5p |
| DBT | hsa-miR-15-5p |
| DBT | hsa-miR-1897-5p |
| DBT | hsa-miR-500 |
| DBT | hsa-miR-499b-5p |
| DBT | hsa-miR-6791-3p |
| DBT | hsa-miR-621 |
| DBT | hsa-miR-139-3p |
| DBT | hsa-miR-1294 |
| DBT | hsa-miR-5702 |
| DBT | hsa-miR-519e-5p |
| DBT | hsa-miR-5694 |
| DBT | hsa-miR-1228-3p |
| DBT | hsa-miR-544-3p |
| DBT | hsa-miR-324-3p |
| DBT | hsa-miR-5006-3p |
| DBT | hsa-miR-23 |
| DBT | hsa-miR-518-5p |
| DBT | hsa-miR-4657 |
| DBT | hsa-miR-130b-5p |
| DBT | hsa-miR-302c-3p |
| DBT | hsa-miR-8062 |
| DBT | hsa-miR-7159-3p |
| DBT | hsa-miR-524-3p |
| DBT | hsa-miR-5582-3p |
| DBT | hsa-miR-4420 |
| DBT | hsa-miR-139-5p |
| DBT | hsa-miR-4699-3p |
| DBT | hsa-miR-2339 |
| DBT | hsa-miR-4272 |
| DBT | hsa-miR-543-3p |
| DBT | hsa-miR-1239 |
| DBT | hsa-miR-6867-5p |
| DBT | hsa-miR-556-3p |
| DBT | hsa-miR-1301-3p |
| DBT | hsa-miR-452-3p |
| DBT | hsa-miR-449-5p |
| DBT | hsa-miR-450b-5p |
| DBT | hsa-miR-411-5p.1 |
| DBT | hsa-miR-3083-5p |
| DBT | hsa-miR-563 |
| DBT | hsa-miR-7386a-3-3p |
| DBT | hsa-miR-2364 |
| DBT | hsa-miR-3187-3p |
| DBT | hsa-miR-8793 |
| DBT | hsa-miR-5681b |
| DBT | hsa-miR-5002-5p |
| DBT | hsa-miR-7352-5p |
| DBT | hsa-miR-3677-5p |
| DBT | hsa-miR-7188-5p |
| DBT | hsa-miR-877-5p |
| DBT | hsa-miR-2285e |
| DBT | hsa-miR-1667-3p |
| DBT | hsa-miR-7344-3p |
| DBT | hsa-miR-10a-3p |
| DBT | hsa-miR-291-3p |
| DBT | hsa-miR-2284z |
| DBT | hsa-miR-2399-5p |
| DBT | hsa-miR-2421 |
| DBT | hsa-miR-8862 |
| DBT | hsa-miR-3166 |
| DBT | hsa-miR-4795-5p |
| DBT | hsa-miR-6608-3p |
| DBT | hsa-miR-1295b-5p |
| DBT | hsa-miR-627-3p |
| DBT | hsa-miR-409-5p |
| DBT | hsa-miR-208-3p |
| DBT | hsa-miR-4726-3p |
| DBT | hsa-miR-6997-5p |
| DBT | hsa-miR-8055 |
| DBT | hsa-miR-4668-3p |
| DBT | hsa-miR-4732-5p |
| DBT | hsa-miR-1184 |
| DBT | hsa-miR-7183-5p |
| DBT | hsa-miR-580 |
| DBT | hsa-miR-7386h-5p |
| DBT | hsa-miR-181b-1-3p |
| DBT | hsa-miR-6849-3p |
| DBT | hsa-miR-141-5p |
| DBT | hsa-miR-576-3p |
| DBT | hsa-miR-518-3p |
| DBT | hsa-miR-206-5p |
| DBT | hsa-miR-8808 |
| DBT | hsa-miR-519 |
| DBT | hsa-miR-6501-3p |
| DBT | hsa-miR-3976 |
| DBT | hsa-miR-140-5p |
| DBT | hsa-miR-1302 |
| DBT | hsa-miR-4663 |
| DBT | hsa-miR-4659a-5p |
| DBT | hsa-miR-4298 |
| DBT | hsa-miR-635 |
| DBT | hsa-miR-668 |
| DBT | hsa-miR-5000-3p |
| DBT | hsa-miR-302b-5p |
| DBT | hsa-miR-6743-3p |
| DBT | hsa-miR-3066-5p |
| DBT | hsa-miR-32-5p |
| DBT | hsa-miR-6870-3p |
| DBT | hsa-miR-4329 |
| DBT | hsa-miR-1277-5p |
| DBT | hsa-miR-4717-3p |
| DBT | hsa-miR-513c-5p |
| DBT | hsa-miR-520-3p |
| DBT | hsa-miR-628-3p |
| DBT | hsa-miR-2404 |
| DBT | hsa-miR-8857 |
| DBT | hsa-miR-4269 |
| DBT | hsa-miR-4796-3p |
| DBT | hsa-miR-28-5p |
| DBT | hsa-miR-4646-3p |
| DBT | hsa-miR-492 |
| DBT | hsa-miR-7399-5p |
| DBT | hsa-miR-1298-3p |
| DBT | hsa-miR-4501 |
| DBT | hsa-miR-4776-3p |
| DBT | hsa-miR-1227-3p |
| DBT | hsa-miR-200c-3p |
| DBT | hsa-miR-126-3p |
| DBT | hsa-miR-670 |
| DBT | hsa-miR-8881 |
| DBT | hsa-miR-2397-3p |
| DBT | hsa-miR-4700-5p |
| DBT | hsa-miR-7862 |
| DBT | hsa-miR-320e |
| DBT | hsa-miR-625-5p |
| DBT | hsa-miR-769 |
| DBT | hsa-miR-548am-3p |
| DBT | hsa-miR-6864-5p |
| DBT | hsa-miR-545-3p |
| DBT | hsa-miR-521 |
| DBT | hsa-miR-487b-5p |
| DBT | hsa-miR-764 |
| DBT | hsa-let-7b-3p |
| DBT | hsa-miR-146b-3p |
| DBT | hsa-miR-3619-3p |
| DBT | hsa-miR-4516 |
| DBT | hsa-miR-542 |
| DBT | hsa-miR-6528 |
| DBT | hsa-miR-3620-5p |
| DBT | hsa-miR-1226-3p |
| DBT | hsa-miR-6087 |
| DBT | hsa-miR-4433b-3p |
| DBT | hsa-miR-3190-3p |
| DBT | hsa-miR-487-3p |
| DBT | hsa-miR-3119 |
| DBT | hsa-miR-7199-5p |
| DBT | hsa-miR-3585-5p |
| DBT | hsa-miR-6779-5p |
| DBT | hsa-miR-3154 |
| DBT | hsa-miR-571 |
| DBT | hsa-miR-188-5p |
| DBT | hsa-miR-7371f-5p |
| DBT | hsa-miR-524-5p |
| DBT | hsa-miR-6891-5p |
| DBT | hsa-miR-7183-3p |
| DBT | hsa-miR-3618 |
| DBT | hsa-miR-4756-3p |
| DBT | hsa-miR-4708-5p |
| DBT | hsa-let-7f-1-3p |
| DBT | hsa-miR-4735-5p |
| DBT | hsa-miR-6358 |
| DBT | hsa-miR-4640-5p |
| DBT | hsa-miR-302f |
| DBT | hsa-miR-1290 |
| DBT | hsa-miR-7398p-3p |
| DBT | hsa-miR-581 |
| DBT | hsa-miR-514a-3p |
| DBT | hsa-miR-101-5p |
| DBT | hsa-miR-3655 |
| DBT | hsa-miR-1193-3p |
| DBT | hsa-miR-8849 |
| DBT | hsa-miR-378g |
| DBT | hsa-miR-4303 |
| DBT | hsa-miR-2473 |
| DBT | hsa-miR-3144-3p |
| DBT | hsa-miR-4667-5p |
| DBT | hsa-miR-516b |
| DBT | hsa-miR-29b-2-5p |
| DBT | hsa-miR-130c-5p |
| DBT | hsa-miR-5697 |
| DBT | hsa-miR-4654 |
| DBT | hsa-miR-5189-5p |
| DBT | hsa-miR-2318 |
| DBT | hsa-miR-187-5p |
| DBT | hsa-miR-1434-3p |
| DBT | hsa-miR-615-5p |
| DBT | hsa-miR-211-5p |
| DBT | hsa-miR-6506-5p |
| DBT | hsa-miR-1178-3p |
| DBT | hsa-miR-105-5p |
| DBT | hsa-miR-7092-5p |
| DBT | hsa-miR-706 |
| DBT | hsa-miR-877-5p |
| DBT | hsa-miR-208a-5p |
| DBT | hsa-miR-6756-5p |
| DBT | hsa-miR-487a-3p |
| DBT | hsa-miR-3184-3p |
| DBT | hsa-miR-374b-3p |
| DBT | hsa-miR-4472 |
| DBT | hsa-miR-3182 |
| DBT | hsa-miR-548t-3p |
| DBT | hsa-miR-6840-3p |
| DBT | hsa-miR-7192-3p |
| DBT | hsa-miR-6822-5p |
| DBT | hsa-miR-669e-3p |
| DBT | hsa-miR-30d-3p |
| DBT | hsa-miR-7234-5p |
| DBT | hsa-miR-3945 |
| DBT | hsa-miR-548c-3p |
| DBT | hsa-miR-4313 |
| DBT | hsa-miR-149-3p |
| DBT | hsa-miR-548e-3p |
| DBT | hsa-miR-6778-3p |
| DBT | hsa-miR-410-5p |
| DBT | hsa-miR-548t-5p |
| DBT | hsa-miR-467f |
| DBT | hsa-miR-8887 |
| DBT | hsa-miR-6076 |
| DBT | hsa-miR-503-3p |
| DBT | hsa-miR-1275 |
| DBT | hsa-miR-3074-3p |
| DBT | hsa-miR-8903 |
| DBT | hsa-miR-3564 |
| DBT | hsa-miR-548bb-5p |
| DBT | hsa-miR-223-3p |
| DBT | hsa-miR-21-3p |
| DBT | hsa-miR-3150a-5p |
| DBT | hsa-miR-7195-3p |
| DBT | hsa-miR-3191-5p |
| DBT | hsa-miR-300 |
| DBT | hsa-miR-8073 |
| DBT | hsa-miR-6745 |
| DBT | hsa-miR-4633-5p |
| DBT | hsa-miR-507b |
| DBT | hsa-miR-6715b-3p |
| DBT | hsa-miR-15a-3p |
| DBT | hsa-miR-628-3p |
| DBT | hsa-miR-3132 |
| DBT | hsa-miR-330-3p |
| DBT | hsa-miR-6847-3p |
| DBT | hsa-miR-3168 |
| DBT | hsa-miR-381-3p |
| DBT | hsa-miR-1603 |
| DBT | hsa-let-7abf-3p |
| DBT | hsa-miR-664a-5p |
| DBT | hsa-miR-7286-3p |
| DBT | hsa-miR-660-5p |
| DBT | hsa-miR-6237 |
| DBT | hsa-miR-548 |
| DBT | hsa-miR-342-3p |
| DBT | hsa-miR-7859 |
| DBT | hsa-miR-1388-5p |
| DBT | hsa-miR-431-5p |
| DBT | hsa-miR-466c-5p |
| DBT | hsa-miR-26c |
| DBT | hsa-miR-4517 |
| DBT | hsa-miR-449b-3p |
| DBT | hsa-miR-5586-3p |
| DBT | hsa-miR-301-5p |
| DBT | hsa-miR-1247-3p |
| DBT | hsa-miR-4263 |
| DBT | hsa-miR-548ay-3p |
| DBT | hsa-miR-7660-3p |
| DBT | hsa-miR-221-5p |
| DBT | hsa-miR-9b-3p |
| DBT | hsa-miR-4540 |
| DBT | hsa-miR-4311 |
| DBT | hsa-miR-6699-5p |
| DBT | hsa-miR-548c-5p |
| DBT | hsa-miR-2414 |
| DBT | hsa-miR-6750-5p |
| DBT | hsa-miR-4529-3p |
| DBT | hsa-miR-26b-3p |
| DBT | hsa-miR-7174-5p |
| DBT | hsa-miR-3162-3p |
| DBT | hsa-miR-432-3p |
| DBT | hsa-miR-8066 |
| DBT | hsa-miR-129-5p |
| DBT | hsa-miR-1273f |
| DBT | hsa-miR-6499-3p |
| DBT | hsa-miR-9-5p |
| DBT | hsa-miR-2322-5p |
| DBT | hsa-miR-4658 |
| DBT | hsa-miR-513c-3p |
| DBT | hsa-miR-454 |
| DBT | hsa-miR-3925-3p |
| DBT | hsa-miR-449a-3p |
| DBT | hsa-miR-371b-5p |
| DBT | hsa-miR-4727-3p |
| DBT | hsa-miR-2465 |
| DBT | hsa-miR-1323 |
| DBT | hsa-miR-3179 |
| DBT | hsa-miR-650abc |
| DBT | hsa-miR-6766-5p |
| DBT | hsa-miR-5087 |
| DBT | hsa-miR-7272-5p |
| DBT | hsa-miR-1584-3p |
| DBT | hsa-miR-588 |
| DBT | hsa-miR-223-3p |
| DBT | hsa-miR-6818-3p |
| DBT | hsa-miR-3682-3p |
| DBT | hsa-miR-144-5p |
| DBT | hsa-miR-3653-5p |
| DBT | hsa-miR-644a |
| DBT | hsa-miR-21-5p |
| DBT | hsa-miR-26-3p |
| DBT | hsa-miR-3678-3p |
| DBT | hsa-miR-1293 |
| DBT | hsa-miR-216a-5p |
| DBT | hsa-miR-1233-5p |
| DBT | hsa-miR-617 |
| DBT | hsa-miR-4672 |
| DBT | hsa-miR-34bc-3p |
| DBT | hsa-miR-1225-3p |
| DBT | hsa-miR-6870-5p |
| DBT | hsa-miR-376c-3p |
| DBT | hsa-miR-4495 |
| DBT | hsa-miR-2114-5p |
| DBT | hsa-miR-6888-3p |
| DBT | hsa-miR-7283-3p |
| DBT | hsa-miR-516-5p |
| DBT | hsa-miR-7a-2-3p |
| DBT | hsa-miR-6859-5p |
| DBT | hsa-miR-1254 |
| DBT | hsa-miR-3942-5p |
| DBT | hsa-miR-1343-5p |
| DBT | hsa-miR-3613b |
| DBT | hsa-miR-509-3p |
| DBT | hsa-miR-5011-5p |
| DBT | hsa-miR-8836 |
| DBT | hsa-miR-3125 |
| DBT | hsa-miR-197-3p |
| DBT | hsa-miR-5699-3p |
| DBT | hsa-miR-513a |
| DBT | hsa-miR-1825 |
| DBT | hsa-miR-6516 |
| DBT | hsa-miR-7226-3p |
| DBT | hsa-miR-504-5p.1 |
| DBT | hsa-miR-363-3p |
| DBT | hsa-miR-2681-5p |
| DBT | hsa-miR-6942-3p |
| DBT | hsa-miR-6790-5p |
| DBT | hsa-miR-425-5p |
| DBT | hsa-miR-6765-3p |
| DBT | hsa-miR-373-3p |
| DBT | hsa-miR-134-5p |
| DBT | hsa-miR-6776-5p |
| DBT | hsa-miR-2424 |
| DBT | hsa-miR-6083 |
| DBT | hsa-miR-7154-3p |
| DBT | hsa-miR-1183 |
| DBT | hsa-miR-7110-5p |
| DBT | hsa-miR-3149 |
| DBT | hsa-miR-2334 |
| DBT | hsa-miR-379-3p |
| DBT | hsa-miR-362-3p |
| DBT | hsa-miR-496 |
| DBT | hsa-miR-4781-3p |
| DBT | hsa-miR-510 |
| DBT | hsa-miR-505-3p.2 |
| DBT | hsa-miR-6917-5p |
| DBT | hsa-miR-6885-3p |
| DBT | hsa-miR-6127 |
| DBT | hsa-miR-190-3p |
| DBT | hsa-miR-4755-5p |
| DBT | hsa-miR-3187-5p |
| DBT | hsa-miR-370-3p |
| DBT | hsa-miR-4276 |
| DBT | hsa-miR-2392 |
| DBT | hsa-miR-8851 |
| DBT | hsa-miR-7091-3p |
| DBT | hsa-miR-344-3p |
| DBT | hsa-miR-8904a |
| DBT | hsa-miR-6516-5p |
| DBT | hsa-miR-374c-5p |
| DBT | hsa-miR-98-3p |
| DBT | hsa-miR-4762-5p |
| DBT | hsa-miR-5007-5p |
| DBT | hsa-miR-7061-5p |
| DBT | hsa-miR-325 |
| DBT | hsa-miR-5095 |
| DBT | hsa-miR-1295b-3p |
| DBT | hsa-miR-1178-3p |
| DBT | hsa-miR-1226-3p |
| DBT | hsa-miR-548h-5p |
| DBT | hsa-miR-762 |
| DBT | hsa-miR-1844 |
| DBT | hsa-miR-4652-3p |
| DBT | hsa-miR-4494 |
| DBT | hsa-miR-4680-5p |
| DBT | hsa-miR-2284 |
| DBT | hsa-miR-4419a |
| DBT | hsa-miR-374 |
| DBT | hsa-miR-5589-5p |
| DBT | hsa-miR-876-3p |
| DBT | hsa-miR-6741-3p |
| DBT | hsa-miR-7-3p |
| DBT | hsa-miR-511-5p |
| DBT | hsa-miR-5088-3p |
| DBT | hsa-miR-3926 |
| DBT | hsa-miR-4436b-5p |
| DBT | hsa-miR-207 |
| DBT | hsa-miR-454-3p |
| DBT | hsa-miR-27-3p |
| DBT | hsa-miR-423-3p |
| DBT | hsa-miR-3065-3p |
| DBT | hsa-miR-92 |
| DBT | hsa-miR-1842 |
| DBT | hsa-miR-514a-5p |
| DBT | hsa-miR-4768-3p |
| DBT | hsa-miR-1287 |
| DBT | hsa-miR-350 |
| DBT | hsa-miR-6806-3p |
| DBT | hsa-miR-151-3p |
| DBT | hsa-miR-5004-5p |
| DBT | hsa-miR-150-5p |
| DBT | hsa-miR-4671-3p |
| DBT | hsa-miR-200 |
| DBT | hsa-miR-3560 |
| DBT | hsa-miR-651 |
| DBT | hsa-miR-324 |
| DBT | hsa-miR-291 |
| DBT | hsa-miR-20-5p |
| DBT | hsa-miR-769-3p |
| DBT | hsa-miR-653-5p |
| DBT | hsa-miR-580-5p |
| DBT | hsa-miR-7208-3p |
| DBT | hsa-miR-452-5p |
| DBT | hsa-miR-2917 |
| DBT | hsa-miR-592-3p |
| DBT | hsa-miR-16-1-3p |
| DBT | hsa-miR-5591-3p |
| DBT | hsa-miR-205-5p |
| DBT | hsa-miR-509-3p |
| DBT | hsa-miR-6835-5p |
| DBT | hsa-miR-2117 |
| DBT | hsa-miR-539-5p |
| DBT | hsa-miR-7109-5p |
| DBT | hsa-miR-667-5p |
| DBT | hsa-miR-7193-5p |
| DBT | hsa-miR-6503-3p |
| DBT | hsa-miR-466f-3p |
| DBT | hsa-miR-6749-3p |
| DBT | hsa-miR-23-3p |
| DBT | hsa-miR-495-3p |
| DBT | hsa-miR-7349-3p |
| DBT | hsa-miR-455-5p |
| DBT | hsa-miR-6124 |
| DBT | hsa-miR-27-5p |
| DBT | hsa-miR-132-3p |
| DBT | hsa-miR-4522 |
| DBT | hsa-miR-7851-3p |
| DBT | hsa-miR-650 |
| DBT | hsa-miR-6934-5p |
| DBT | hsa-miR-2293 |
| DBT | hsa-miR-1234-3p |
| DBT | hsa-miR-221-3p |
| DBT | hsa-miR-550a-3-5p |
| DBT | hsa-miR-6833-3p |
| DBT | hsa-miR-2113 |
| DBT | hsa-miR-4697-5p |
| DBT | hsa-miR-2382-3p |
| DBT | hsa-miR-130a-5p |
| DBT | hsa-miR-34c-5p |
| DBT | hsa-miR-6120-5p |
| DBT | hsa-miR-6864-3p |
| DBT | hsa-miR-6742-3p |
| DBT | hsa-miR-105b |
| DBT | hsa-miR-491-3p |
| DBT | hsa-miR-142-5p |
| DBT | hsa-miR-365-5p |
| DBT | hsa-miR-3915 |
| DBT | hsa-miR-741-3p |
| DBT | hsa-miR-27a-5p |
| DBT | hsa-miR-7013-5p |
| DBT | hsa-miR-548ap-5p |
| DBT | hsa-miR-9-3p |
| DBT | hsa-miR-216b |
| DBT | hsa-miR-103-3p |
| DBT | hsa-miR-1271-5p |
| DBT | hsa-miR-518a-5p |
| DBT | hsa-miR-30-5p |
| DBT | hsa-miR-3134 |
| DBT | hsa-miR-3606-3p |
| DBT | hsa-miR-589-5p |
| DBT | hsa-miR-2380 |
| DBT | hsa-miR-590-5p |
| DBT | hsa-miR-8087 |
| DBT | hsa-miR-624-3p |
| DBT | hsa-miR-4428 |
| DBT | hsa-miR-4691-3p |
| DBT | hsa-miR-7335-3p |
| DBT | hsa-miR-4499 |
| DBT | hsa-miR-200bc |
| DBT | hsa-miR-3193 |
| DBT | hsa-miR-8839 |
| DBT | hsa-miR-3150b-5p |
| DBT | hsa-miR-4687-3p |
| DBT | hsa-miR-6783-5p |
| DBT | hsa-miR-4287 |
| DBT | hsa-miR-376ab-3p |
| DBT | hsa-miR-7441-5p |
| DBT | hsa-miR-7229-3p |
| DBT | hsa-miR-875-3p |
| DBT | hsa-miR-3074-2-3p |
| DBT | hsa-miR-214-3p |
| DBT | hsa-miR-1a-5p |
| DBT | hsa-miR-7210-3p |
| DBT | hsa-miR-465a-5p |
| DBT | hsa-miR-586 |
| DBT | hsa-miR-382-5p |
| DBT | hsa-miR-513a-3p |
| DBT | hsa-miR-2284aa |
| DBT | hsa-miR-592 |
| DBT | hsa-let-7c-1-3p |
| DBT | hsa-let-7ag-3p |
| DBT | hsa-miR-6800-3p |
| DBT | hsa-miR-889 |
| DBT | hsa-miR-323a-5p |
| DBT | hsa-let-7-3p |
| DBT | hsa-miR-566 |
| DBT | hsa-miR-561-3p |
| DBT | hsa-miR-4766-3p |
| DBT | hsa-miR-2432 |
| DBT | hsa-miR-548aa |
| DBT | hsa-miR-7387-3p |
| DBT | hsa-miR-1248 |
| DBT | hsa-miR-4771 |
| DBT | hsa-miR-8841 |
| DBT | hsa-miR-6735-5p |
| DBT | hsa-miR-3138 |
| DBT | hsa-miR-2054 |
| DBT | hsa-miR-4650 |
| DBT | hsa-miR-7054-3p |
| DBT | hsa-miR-3201 |
| DBT | hsa-miR-7044-3p |
| DBT | hsa-miR-196 |
| DBT | hsa-miR-8068 |
| DBT | hsa-miR-4432 |
| DBT | hsa-miR-141-3p |
| DBT | hsa-miR-3104-3p |
| DBT | hsa-miR-517-5p |
| DBT | hsa-miR-518 |
| DBT | hsa-miR-656-3p |
| DBT | hsa-miR-2387 |
| DBT | hsa-miR-6780b-5p |
| DBT | hsa-miR-376c-5p |
| DBT | hsa-miR-497-5p |
| DBT | hsa-miR-7310-5p |
| DBT | hsa-miR-6867-3p |
| DBT | hsa-miR-143-3p |
| DBT | hsa-miR-763 |
| DBT | hsa-miR-4536-5p |
| DBT | hsa-miR-660-5p |
| DBT | hsa-miR-1909-5p |
| DBT | hsa-miR-142b |
| DBT | hsa-miR-380-5p |
| DBT | hsa-miR-3094-3p |
| DBT | hsa-miR-7173-3p |
| DBT | hsa-miR-8094 |
| DBT | hsa-miR-1966-3p |
| DBT | hsa-miR-676-5p |
| DBT | hsa-miR-4454 |
| DBT | hsa-miR-3151-5p |
| DBT | hsa-miR-190a-5p |
| DBT | hsa-miR-2390 |
| DBT | hsa-miR-6780a-5p |
| DBT | hsa-miR-6515-5p |
| DBT | hsa-miR-3664-5p |
| DBT | hsa-miR-7015-3p |
| DBT | hsa-miR-548ap-3p |
| DBT | hsa-miR-6744-3p |
| DBT | hsa-miR-1323-5p |
| DBT | hsa-miR-4797-5p |
| DBT | hsa-miR-7204-5p |
| DBT | hsa-miR-1303 |
| DBT | hsa-miR-3074-1-3p |
| DBT | hsa-miR-1225-5p |
| DBT | hsa-miR-4651 |
| DBT | hsa-miR-2311 |
| DBT | hsa-miR-891-3p |
| DBT | hsa-miR-642-3p |
| DBT | hsa-miR-7389-3p |
| DBT | hsa-miR-4509 |
| DBT | hsa-miR-546 |
| DBT | hsa-miR-7386g-5p |
| DBT | hsa-miR-216b-3p |
| DBT | hsa-miR-4723-5p |
| DBT | hsa-miR-6728-3p |
| DBT | hsa-miR-1306-3p |
| DBT | hsa-miR-3617-3p |
| DBT | hsa-miR-2482 |
| DBT | hsa-miR-548s |
| DBT | hsa-miR-7162-3p |
| DBT | hsa-miR-7288-3p |
| DBT | hsa-miR-1229-5p |
| DBT | hsa-miR-99-3p |
| DBT | hsa-miR-7248-5p |
| DBT | hsa-miR-3199 |
| DBT | hsa-miR-9-5p |
| DBT | hsa-miR-7861 |
| DBT | hsa-miR-7307-3p |
| DBT | hsa-miR-5706 |
| DBT | hsa-miR-2114-3p |
| DBT | hsa-miR-4713-3p |
| DBT | hsa-miR-4504 |
| DBT | hsa-miR-3663-3p |
| DBT | hsa-miR-370 |
| DBT | hsa-miR-1305 |
| DBT | hsa-miR-6838-3p |
| DBT | hsa-miR-7277-3p |
| DBT | hsa-miR-1233-3p |
| DBT | hsa-miR-7207-5p |
| DBT | hsa-miR-7386g-3p |
| DBT | hsa-miR-18a-3p |
| DBT | hsa-miR-6992-3p |
| DBT | hsa-miR-199-5p |
| DBT | hsa-miR-4772-5p |
| DBT | hsa-miR-181-5p |
| DBT | hsa-miR-8084 |
| DBT | hsa-miR-623 |
| DBT | hsa-miR-499b-3p |
| DBT | hsa-miR-3909 |
| DBT | hsa-miR-4776-5p |
| DBT | hsa-miR-291b-3p |
| DBT | hsa-miR-432-5p |
| DBT | hsa-miR-4639-3p |
| DBT | hsa-miR-517-3p |
| DBT | hsa-miR-1243-5p |
| DBT | hsa-miR-365-3p |
| DBT | hsa-miR-2458 |
| DBT | hsa-miR-5683 |
| DBT | hsa-miR-337-3p |
| DBT | hsa-let-7f-2-3p |
| DBT | hsa-miR-3126-3p |
| DBT | hsa-miR-2439-5p |
| DBT | hsa-miR-210-5p |
| DBT | hsa-miR-8823 |
| DBT | hsa-miR-7648-3p |
| DBT | hsa-miR-1261 |
| DBT | hsa-miR-215 |
| DBT | hsa-miR-6868-3p |
| DBT | hsa-miR-3117-5p |
| DBT | hsa-miR-674-5p |
| DBT | hsa-miR-10b-3p |
| DBT | hsa-miR-1301 |
| DBT | hsa-miR-622 |
| DBT | hsa-miR-5623-3p |
| DBT | hsa-miR-3135b |
| DBT | hsa-miR-6531 |
| DBT | hsa-miR-5008-3p |
| DBT | hsa-miR-493-5p |
| DBT | hsa-miR-154-3p |
| DBT | hsa-miR-6894-5p |
| DBT | hsa-miR-7055-3p |
| DBT | hsa-miR-7352-3p |
| DBT | hsa-miR-2460 |
| DBT | hsa-miR-7180-3p |
| DBT | hsa-miR-616-5p |
| DBT | hsa-miR-224-3p |
| DBT | hsa-miR-1179 |
| DBT | hsa-miR-8843 |
| DBT | hsa-miR-6771-3p |
| DBT | hsa-miR-4703-3p |
| DBT | hsa-miR-4662-5p |
| DBT | hsa-miR-500-5p |
| DBT | hsa-miR-92-3p |
| DBT | hsa-miR-2488 |
| DBT | hsa-miR-375 |
| DBT | hsa-miR-1241 |
| DBT | hsa-miR-3106-5p |
| DBT | hsa-miR-16-2-3p |
| DBT | hsa-miR-548j-3p |
| DBT | hsa-miR-6924-3p |
| DBT | hsa-miR-122 |
| DBT | hsa-miR-6830-3p |
| DBT | hsa-miR-103b |
| DBT | hsa-miR-2446 |
| DBT | hsa-miR-1234-3p |
| DBT | hsa-miR-1237-3p |
| DBT | hsa-miR-3588 |
| DBT | hsa-miR-7110-3p |
| DBT | hsa-miR-6854-5p |
| DBT | hsa-miR-543 |
| DBT | hsa-miR-2357 |
| DBT | hsa-miR-1269a |
| DBT | hsa-miR-5687 |
| DBT | hsa-miR-6514-3p |
| DBT | hsa-miR-2285x |
| DBT | hsa-miR-323a-3p |
| DBT | hsa-miR-2331-5p |
| DBT | hsa-miR-15b-3p |
| DBT | hsa-miR-98-5p |
| DBT | hsa-miR-196-2-3p |
| DBT | hsa-miR-512 |
| DBT | hsa-miR-103 |
| DBT | hsa-miR-2329-5p |
| DBT | hsa-miR-181b-3p |
| DBT | hsa-miR-490-5p |
| DBT | hsa-miR-8844 |
| DBT | hsa-miR-299 |
| DBT | hsa-miR-129-5p |
| DBT | hsa-miR-344f-5p |
| DBT | hsa-miR-219a-5p |
| DBT | hsa-miR-4310 |
| DBT | hsa-miR-154-5p |
| DBT | hsa-miR-7172-3p |
| DBT | hsa-miR-937-5p |
| DBT | hsa-miR-3956 |
| DBT | hsa-miR-4297 |
| DBT | hsa-miR-301b-5p |
| DBT | hsa-miR-8895 |
| DBT | hsa-miR-7151-5p |
| DBT | hsa-miR-548p |
| DBT | hsa-miR-532-5p |
| DBT | hsa-miR-876 |
| DBT | hsa-miR-5187-5p |
| DBT | hsa-miR-607 |
| DBT | hsa-miR-466l-3p |
| DBT | hsa-miR-339-5p |
| DBT | hsa-miR-6505-3p |
| DBT | hsa-miR-331-5p |
| DBT | hsa-miR-1841 |
| DBT | hsa-miR-6130 |
| DBT | hsa-miR-8908f |
| DBT | hsa-miR-6737-3p |
| DBT | hsa-miR-146-5p |
| DBT | hsa-miR-890 |
| DBT | hsa-miR-4648 |
| DBT | hsa-miR-3689d |
| DBT | hsa-miR-2478 |
| DBT | hsa-miR-1911-3p |
| DBT | hsa-miR-148b-5p |
| DBT | hsa-miR-548ab |
| DBT | hsa-miR-2284y |
| DBT | hsa-miR-6752-3p |
| DBT | hsa-miR-223-5p |
| DBT | hsa-miR-548e-5p |
| DBT | hsa-miR-6316 |
| DBT | hsa-miR-744-3p |
| DBT | hsa-miR-7847-3p |
| DBT | hsa-miR-183-5p.2 |
| DBT | hsa-miR-7641 |
| DBT | hsa-miR-382-3p |
| DBT | hsa-miR-629-5p |
| DBT | hsa-miR-7222-5p |
| DBT | hsa-miR-4725-3p |
| DBT | hsa-miR-8848 |
| DBT | hsa-miR-1207-5p |
| DBT | hsa-miR-373-3p |
| DBT | hsa-miR-4769-5p |
| DBT | hsa-miR-548au-3p |
| DBT | hsa-miR-548d-5p |
| DBT | hsa-miR-3596 |
| DBT | hsa-miR-520f-5p |
| DBT | hsa-miR-2400 |
| DBT | hsa-miR-4676-5p |
| DBT | hsa-miR-1295a |
| DBT | hsa-miR-4745-5p |
| DBT | hsa-miR-2362 |
| DBT | hsa-miR-548az-5p |
| DBT | hsa-miR-655 |
| DBT | hsa-miR-655-5p |
| DBT | hsa-miR-1343-3p |
| DBT | hsa-miR-6881-3p |
| DBT | hsa-miR-5684 |
| DBT | hsa-miR-758-3p |
| DBT | hsa-miR-216 |
| DBT | hsa-miR-7157-3p |
| DBT | hsa-miR-383 |
| DBT | hsa-miR-1252-5p |
| DBT | hsa-miR-1913 |
| DBT | hsa-miR-532-5p |
| DBT | hsa-miR-7050-3p |
| DBT | hsa-miR-873b |
| DBT | hsa-miR-6895-3p |
| DBT | hsa-let-7-5p |
| DBT | hsa-miR-759 |
| DBT | hsa-miR-200c-3p |
| DBT | hsa-miR-4459 |
| DBT | hsa-miR-31-5p |
| DBT | hsa-miR-143-3p |
| DBT | hsa-miR-376a-1-5p |
| DBT | hsa-miR-3157-5p |
| DBT | hsa-miR-431-5p |
| DBT | hsa-miR-6757-3p |
| DBT | hsa-miR-5682 |
| DBT | hsa-miR-215-3p |
| DBT | hsa-miR-3121-5p |
| DBT | hsa-miR-378d |
| DBT | hsa-miR-2384 |
| DBT | hsa-miR-7705 |
| DBT | hsa-miR-7855-5p |
| DBT | hsa-miR-6715-3p |
| DBT | hsa-miR-7393-5p |
| DBT | hsa-miR-30ad-3p |
| DBT | hsa-miR-496.1 |
| DBT | hsa-miR-7111-3p |
| DBT | hsa-miR-708-5p |
| DBT | hsa-miR-6940-5p |
| DBT | hsa-miR-605-3p |
| DBT | hsa-miR-4482-5p |
| DBT | hsa-miR-5692bc |
| DBT | hsa-miR-410-3p |
| DBT | hsa-miR-892a |
| DBT | hsa-miR-8869 |
| DBT | hsa-miR-614 |
| DBT | hsa-miR-3667-5p |
| DBT | hsa-miR-3156-5p |
| DBT | hsa-miR-218-5p |
| DBT | hsa-miR-7191-3p |
| DBT | hsa-miR-33-3p |
| DBT | hsa-miR-8845 |
| DBT | hsa-miR-4778-5p |
| DBT | hsa-miR-488-3p |
| DBT | hsa-miR-653-3p |
| DBT | hsa-miR-664-3p |
| DBT | hsa-miR-340-3p |
| DBT | hsa-miR-1587 |
| DBT | hsa-miR-513 |
| DBT | hsa-miR-339-5p |
| DBT | hsa-miR-561-3p |
| DBT | hsa-miR-136-5p |
| DBT | hsa-miR-1b-5p |
| DBT | hsa-miR-6834-5p |
| DBT | hsa-miR-5707 |
| DBT | hsa-miR-742-3p |
| DBT | hsa-miR-335-5p |
| DBT | hsa-miR-24-5p |
| DBT | hsa-miR-190a-3p |
| DBT | hsa-miR-7188-3p |
| DBT | hsa-miR-8838 |
| DBT | hsa-miR-553 |
| DBT | hsa-miR-6879-5p |
| DBT | hsa-miR-6776-3p |
| DBT | hsa-miR-296-5p |
| DBT | hsa-miR-6860 |
| DBT | hsa-miR-2276-3p |
| DBT | hsa-miR-7674-5p |
| DBT | hsa-miR-203a-3p.1 |
| DBT | hsa-miR-634 |
| DBT | hsa-miR-424-3p |
| DBT | hsa-miR-2284q |
| DBT | hsa-miR-7702 |
| DBT | hsa-miR-150-5p |
| DBT | hsa-miR-650d |
| DBT | hsa-miR-4301 |
| DBT | hsa-miR-938 |
| DBT | hsa-miR-3714 |
| DBT | hsa-miR-6752-5p |
| DBT | hsa-miR-3202 |
| DBT | hsa-miR-596 |
| DBT | hsa-miR-875-5p |
| DBT | hsa-miR-1245a |
| DBT | hsa-miR-1343 |
| DBT | hsa-miR-124-3p.1 |
| DBT | hsa-miR-2284a |
| DBT | hsa-miR-30-3p |
| DBT | hsa-miR-3657 |
| DBT | hsa-miR-7006-3p |
| DBT | hsa-miR-702-5p |
| DBT | hsa-miR-383-3p |
| DBT | hsa-miR-8801 |
| DBT | hsa-miR-3072 |
| DBT | hsa-miR-2298 |
| DBT | hsa-miR-147a |
| DBT | hsa-miR-6715b-5p |
| DBT | hsa-miR-934-3p |
| DBT | hsa-miR-6861-3p |
| DBT | hsa-miR-6529-3p |
| DBT | hsa-miR-5590-5p |
| DBT | hsa-miR-6548-5p |
| DBT | hsa-miR-367-3p |
| DBT | hsa-miR-23-5p |
| DBT | hsa-miR-6890-5p |
| DBT | hsa-miR-2410 |
| DBT | hsa-miR-3667-3p |
| DBT | hsa-miR-8098 |
| DBT | hsa-miR-7211-5p |
| DBT | hsa-miR-3967 |
| DBT | hsa-miR-1-5p |
| DBT | hsa-miR-7180 |
| DBT | hsa-miR-576-5p |
| DBT | hsa-miR-3590-3p |
| DBT | hsa-miR-2398 |
| DBT | hsa-miR-297 |
| DBT | hsa-miR-6874-3p |
| DBT | hsa-miR-7386m-3p |
| DBT | hsa-miR-4733-3p |
| DBT | hsa-miR-548as-3p |
| DBT | hsa-miR-2447 |
| DBT | hsa-miR-181 |
| DBT | hsa-miR-2369 |
| DBT | hsa-miR-193-5p |
| DBT | hsa-miR-6754-3p |
| DBT | hsa-miR-2486-3p |
| DBT | hsa-miR-26a-3p |
| DBT | hsa-miR-3674 |
| DBT | hsa-miR-302-5p |
| DBT | hsa-miR-4511 |
| DBT | hsa-miR-376a-2-5p |
| DBT | hsa-miR-624-3p |
| DBT | hsa-miR-4723-3p |
| DBT | hsa-miR-2406 |
| DBT | hsa-miR-4433a-3p |
| DBT | hsa-miR-4271 |
| DBT | hsa-miR-1973 |
| DBT | hsa-miR-612 |
| DBT | hsa-miR-3082-5p |
| DBT | hsa-miR-371 |
| DBT | hsa-miR-595 |
| DBT | hsa-miR-2359 |
| DBT | hsa-miR-2361 |
| DBT | hsa-miR-499 |
| DBT | hsa-miR-7374a-3p |
| DBT | hsa-miR-129-2-3p |
| DBT | hsa-miR-4419b |
| DBT | hsa-miR-4685-3p |
| DBT | hsa-miR-4789-5p |
| DBT | hsa-miR-4748 |
| DBT | hsa-miR-887-5p |
| DBT | hsa-miR-604 |
| DBT | hsa-miR-7192-5p |
| DBT | hsa-miR-500-3p |
| DBT | hsa-miR-7049-3p |
| DBT | hsa-miR-455-3p.2 |
| DBT | hsa-miR-185-5p |
| DBT | hsa-miR-433-3p |
| DBT | hsa-miR-4670-3p |
| DBT | hsa-miR-3607-3p |
| DBT | hsa-miR-204-3p |
| DBT | hsa-miR-3183 |
| DBT | hsa-miR-8831 |
| DBT | hsa-miR-668-3p |
| DBT | hsa-miR-196-3p |
| DBT | hsa-miR-1185-3p |
| DBT | hsa-miR-6721-5p |
| DBT | hsa-miR-294 |
| DBT | hsa-miR-7172-5p |
| DBT | hsa-miR-606 |
| DBT | hsa-miR-3529-3p |
| DBT | hsa-miR-139-5p |
| DBT | hsa-miR-8898 |
| DBT | hsa-miR-6514-5p |
| DBT | hsa-miR-579 |
| DBT | hsa-miR-3145-5p |
| DBT | hsa-miR-6788-5p |
| DBT | hsa-miR-5571-5p |
| DBT | hsa-miR-4450 |
| DBT | hsa-miR-3143 |
| DBT | hsa-miR-879-3p |
| DBT | hsa-miR-3653-3p |
| DBT | hsa-miR-6728-5p |
| DBT | hsa-miR-2284k |
| DBT | hsa-miR-568 |
| DBT | hsa-miR-4721 |
| DBT | hsa-miR-200a-3p |
| DBT | hsa-miR-7241-3p |
| DBT | hsa-miR-2375 |
| DBT | hsa-miR-372 |
| DBT | hsa-miR-323-3p |
| DBT | hsa-miR-28-3p |
| DBT | hsa-miR-8877 |
| DBT | hsa-miR-292b-5p |
| DBT | hsa-miR-6846-5p |
| DBT | hsa-miR-6751-5p |
| DBT | hsa-miR-1267 |
| DBT | hsa-miR-377-5p |
| DBT | hsa-miR-7281-3p |
| DBT | hsa-miR-300-5p |
| DBT | hsa-miR-191-5p |
| DBT | hsa-miR-6855-3p |
| DBT | hsa-miR-8902 |
| DBT | hsa-miR-411-5p |
| DBT | hsa-miR-4720-3p |
| DBT | hsa-miR-5704 |
| DBT | hsa-miR-181b-2-3p |
| DBT | hsa-miR-33a-3p |
| DBT | hsa-miR-6134 |
| DBT | hsa-miR-485 |
| DBT | hsa-miR-6756-3p |
| DBT | hsa-miR-7173-5p |
| DBT | hsa-miR-432-5p |
| DBT | hsa-miR-409 |
| DBT | hsa-miR-153 |
| DBT | hsa-miR-7114-5p |
| DBT | hsa-miR-548at-3p |
| DBT | hsa-miR-3682-5p |
| DBT | hsa-miR-513a-5p |
| DBT | hsa-miR-3059-5p |
| DBT | hsa-miR-7163-3p |
| DBT | hsa-miR-6549-3p |
| DBT | hsa-miR-1283 |
| DBT | hsa-miR-3977 |
| DBT | hsa-miR-3192-3p |
| DBT | hsa-miR-3617-5p |
| DBT | hsa-miR-548b-3p |
| DBT | hsa-miR-1208 |
| DBT | hsa-miR-3170 |
| DBT | hsa-miR-4698 |
| DBT | hsa-miR-340-5p |
| DBT | hsa-miR-3102-3p.2-3p |
| DBT | hsa-miR-6827-3p |
| DBT | hsa-miR-8061 |
| DBT | hsa-miR-548az-3p |
| DBT | hsa-miR-2116-3p |
| DBT | hsa-miR-8069 |
| DBT | hsa-miR-10 |
| DBT | hsa-miR-2277-3p |
| DBT | hsa-miR-548ah-3p |
| DBT | hsa-miR-7094-1-5p |
| DBT | hsa-miR-4284 |
| DBT | hsa-miR-2442 |
| DBT | hsa-miR-379-5p |
| DBT | hsa-miR-124-5p |
| DBT | hsa-miR-3907 |
| DBT | hsa-miR-7675-3p |
| DBT | hsa-miR-369-3p |
| DBT | hsa-miR-3972 |
| DBT | hsa-miR-7072-5p |
| DBT | hsa-miR-6785-5p |
| DBT | hsa-miR-6832-3p |
| DBT | hsa-miR-335-3p |
| DBT | hsa-miR-4768-5p |
| DBT | hsa-miR-2373-5p |
| DBT | hsa-miR-140-3p.2 |
| DBT | hsa-miR-2360 |
| DBT | hsa-miR-1258 |
| DBT | hsa-miR-6855-5p |
| DBT | hsa-miR-224-5p |
| DBT | hsa-miR-6811-3p |
| DBT | hsa-miR-1897-3p |
| DBT | hsa-miR-193-3p |
| DBT | hsa-miR-7026-3p |
| DBT | hsa-miR-5579-5p |
| DBT | hsa-miR-3084-3p |
| DBT | hsa-miR-4482-3p |
| DBT | hsa-miR-6792-3p |
| DBT | hsa-miR-1271-3p |
| DBT | hsa-miR-377-3p |
| DBT | hsa-miR-1950 |
| DBT | hsa-miR-4804-3p |
| DBT | hsa-miR-96-3p |
| DBT | hsa-miR-373-5p |
| DBT | hsa-miR-4253 |
| DBT | hsa-miR-8797 |
| DBT | hsa-miR-1282 |
| DBT | hsa-miR-4666a-3p |
| DBT | hsa-miR-550 |
| DBT | hsa-miR-1224-5p |
| DBT | hsa-miR-544b |
| DBT | hsa-miR-613 |
| DBT | hsa-miR-1255b-2-3p |
| DBT | hsa-miR-6506-3p |
| DBT | hsa-miR-548bb-3p |
| DBT | hsa-miR-4715-3p |
| DBT | hsa-miR-4463 |
| DBT | hsa-miR-92a-5p |
| DBT | hsa-miR-33b-3p |
| DBT | hsa-miR-3146 |
| DBT | hsa-miR-6769b-5p |
| DBT | hsa-miR-2285u |
| DBT | hsa-miR-4999-3p |
| DBT | hsa-miR-495-3p |
| DBT | hsa-miR-548o-5p |
| DBT | hsa-miR-3163 |
| DBT | hsa-miR-599-5p |
| DBT | hsa-miR-30 |
| DBT | hsa-miR-1244 |
| DBT | hsa-miR-5697-3p |
| DBT | hsa-miR-4638-3p |
| DBT | hsa-miR-33 |
| DBT | hsa-miR-7000-3p |
| DBT | hsa-miR-1185-5p |
| DBT | hsa-miR-4766-5p |
| DBT | hsa-miR-127-5p |
| DBT | hsa-miR-365b-5p |
| DBT | hsa-miR-1180-5p |
| DBT | hsa-miR-338-3p |
| DBT | hsa-miR-548aj-3p |
| DBT | hsa-miR-191-3p |
| DBT | hsa-miR-31-3p |
| DBT | hsa-miR-2284b |
| DBT | hsa-miR-666-3p |
| DBT | hsa-miR-516b-5p |
| DBT | hsa-miR-26 |
| DBT | hsa-miR-514-3p |
| DBT | hsa-miR-552 |
| DBT | hsa-miR-6851-3p |
| DBT | hsa-miR-3658 |
| DBT | hsa-miR-294-5p |
| DBT | hsa-miR-7371a-3p |
| DBT | hsa-miR-487-5p |
| DBT | hsa-miR-939-5p |
| DBT | hsa-miR-921 |
| DBT | hsa-miR-5579-3p |
| DBT | hsa-miR-3935 |
| DBT | hsa-miR-4689 |
| DBT | hsa-miR-193a |
| DBT | hsa-miR-8095 |
| DBT | hsa-miR-488-5p |
| DBT | hsa-miR-1291 |
| DBT | hsa-miR-8842 |
| DBT | hsa-miR-2322-3p |
| DBT | hsa-miR-135a-1-3p |
| DBT | hsa-miR-4796-5p |
| DBT | hsa-miR-4793-3p |
| DBT | hsa-miR-4257 |
| DBT | hsa-miR-6508-3p |
| DBT | hsa-miR-4423 |
| DBT | hsa-miR-556-5p |
| DBT | hsa-miR-4328 |
| DBT | hsa-miR-6501-5p |
| DBT | hsa-miR-548i-3p |
| DBT | hsa-miR-4435 |
| DBT | hsa-miR-1245b-5p |
| DBT | hsa-miR-335-5p |
| DBT | hsa-miR-320a |
| DBT | hsa-miR-6830-5p |
| DBT | hsa-miR-663b |
| DBT | hsa-miR-2887 |
| DBT | hsa-miR-6797-3p |
| DBT | hsa-miR-511-3p |
| DBT | hsa-miR-6768-5p |
| DBT | hsa-miR-5008-5p |
| DBT | hsa-miR-548ak |
| DBT | hsa-miR-6848-3p |
| DBT | hsa-miR-6925-3p |
| DBT | hsa-miR-6505-5p |
| DBT | hsa-miR-4696 |
| DBT | hsa-miR-7190-5p |
| DBT | hsa-miR-7394b-5p |
| DBT | hsa-miR-450a-2-3p |
| DBT | hsa-miR-196b-3p |
| DBT | hsa-miR-4714-3p |
| DBT | hsa-miR-767-5p |
| DBT | hsa-miR-15c-3p |
| DBT | hsa-miR-6873-3p |
| DBT | hsa-miR-743ab-3p |
| DBT | hsa-miR-129b-3p |
| DBT | hsa-miR-3190-5p |
| DBT | hsa-miR-548ar-5p |
| DBT | hsa-miR-24-3p |
| DBT | hsa-miR-409-3p |
| DBT | hsa-miR-1251-3p |
| DBT | hsa-miR-575 |
| DBT | hsa-miR-6760-3p |
| DBT | hsa-miR-7270-5p |
| DBT | hsa-miR-6845-3p |
| DBT | hsa-miR-450b-3p |
| DBT | hsa-miR-6831-3p |
| DBT | hsa-miR-548ae-3p |
| DBT | hsa-miR-6641-5p |
| DBT | hsa-miR-1542-5p |
| DBT | hsa-miR-630 |
| DBT | hsa-miR-2374 |
| DBT | hsa-miR-7254-5p |
| DBT | hsa-miR-619 |
| DBT | hsa-miR-6853-5p |
| DBT | hsa-miR-2284c |
| DBT | hsa-miR-9500 |
| DBT | hsa-miR-4802-5p |
| DBT | hsa-miR-6794-3p |
| DBT | hsa-miR-344b-3p |
| DBT | hsa-miR-197-3p |
| DBT | hsa-miR-670-3p |
| DBT | hsa-miR-6503-5p |
| DBT | hsa-miR-7169-5p |
| DBT | hsa-miR-4799-5p |
| DBT | hsa-miR-2422 |
| DBT | hsa-miR-211-5p |
| DBT | hsa-miR-372-5p |
| DBT | hsa-miR-6537-5p |
| DBT | hsa-miR-1251-5p |
| DBT | hsa-miR-7168-5p |
| DBT | hsa-miR-6344 |
| DBT | hsa-miR-3920 |
| DBT | hsa-miR-7116-3p |
| DBT | hsa-miR-1839 |
| DBT | hsa-miR-1257 |
| DBT | hsa-miR-1273g-3p |
| DBT | hsa-miR-5196-3p |
| DBT | hsa-miR-4464 |
| DBT | hsa-miR-122-5p |
| DBT | hsa-miR-548a-3p |
| DBT | hsa-miR-202-5p |
| DBT | hsa-miR-4650-3p |
| DBT | hsa-miR-3129-5p |
| DBT | hsa-miR-32-3p |
| DBT | hsa-miR-2394 |
| DBT | hsa-miR-219-5p |
| DBT | hsa-miR-1285-3p |
| DBT | hsa-miR-2363 |
| DBT | hsa-miR-647 |
| DBT | hsa-miR-4739 |
| DBT | hsa-miR-20a-3p |
| DBT | hsa-miR-548ac |
| DBT | hsa-miR-182-5p |
| DBT | hsa-miR-3130-5p |
| DBT | hsa-miR-6780b-3p |
| DBT | hsa-miR-8850 |
| DBT | hsa-miR-7386n-3p |
| DBT | hsa-miR-487a-5p |
| DBT | hsa-miR-889-5p |
| DBT | hsa-miR-548b-3p |
| DBT | hsa-miR-3605-5p |
| DBT | hsa-miR-8829 |
| DBT | hsa-miR-4669 |
| DBT | hsa-miR-548g-3p |
| DBT | hsa-miR-1249-5p |
| DBT | hsa-miR-186-3p |
| DBT | hsa-miR-323b-3p |
| DBT | hsa-miR-8052 |
| DBT | hsa-miR-2053 |
| DBT | hsa-miR-491-5p |
| DBT | hsa-miR-149-5p |
| DBT | hsa-miR-6733-3p |
| DBT | hsa-miR-1230 |
| DBT | hsa-miR-616-3p |
| DBT | hsa-miR-3663-5p |
| DBT | hsa-miR-4665-5p |
| DBT | hsa-miR-6119-5p |
| DBT | hsa-miR-942 |
| DBT | hsa-miR-199-3p |
| DBT | hsa-miR-4427 |
| DBT | hsa-miR-4317 |
| DBT | hsa-miR-2285g |
| DBT | hsa-miR-2475 |
| DBT | hsa-miR-7199-3p |
| DBT | hsa-miR-1243 |
| DBT | hsa-miR-661 |
| DBT | hsa-miR-4764-3p |
| DBT | hsa-miR-651-5p |
| DBT | hsa-miR-4477b |
| DBT | hsa-miR-183-5p.1 |
| DBT | hsa-miR-345-5p |
| DBT | hsa-miR-138-3p |
| DBT | hsa-miR-7388c-3p |
| DBT | hsa-miR-486-3p |
| DBT | hsa-miR-141-3p |
| DBT | hsa-miR-599 |
| DBT | hsa-miR-3646 |
| DBT | hsa-miR-222-3p |
| DBT | hsa-miR-6071 |
| DBT | hsa-miR-593-3p |
| DBT | hsa-miR-652-3p |
| DBT | hsa-miR-2485 |
| DBT | hsa-miR-2483-3p |
| DBT | hsa-miR-5193 |
| DBT | hsa-miR-548aq-3p |
| DBT | hsa-miR-2285y |
| DBT | hsa-miR-2284n |
| DBT | hsa-miR-6850-3p |
| DBT | hsa-miR-8063 |
| DBT | hsa-miR-410 |
| DBT | hsa-miR-1264-3p |
| DBT | hsa-miR-329-5p |
| DBT | hsa-miR-8075 |
| DBT | hsa-miR-875 |
| DBT | hsa-miR-96-5p |
| DBT | hsa-miR-4533 |
| DBT | hsa-miR-548ar-3p |
| DBT | hsa-miR-6774-5p |
| DBT | hsa-miR-548ag |
| DBT | hsa-miR-449a |
| DBT | hsa-miR-2309 |
| DBT | hsa-miR-516a-5p |
| DBT | hsa-miR-4423-5p |
| DBT | hsa-miR-2287 |
| DBT | hsa-miR-379-5p |
| DBT | hsa-miR-7-5p |
| DBT | hsa-miR-4667 |
| DBT | hsa-miR-21-5p |
| DBT | hsa-miR-6775-3p |
| DBT | hsa-miR-425 |
| DBT | hsa-miR-4637 |
| DBT | hsa-miR-3127-3p |
| DBT | hsa-miR-2285ad |
| DBT | hsa-miR-7863 |
| DBT | hsa-miR-196a-3p |
| DBT | hsa-miR-8114 |
| DBT | hsa-miR-134 |
| DBT | hsa-miR-885-5p |
| DBT | hsa-miR-562 |
| DBT | hsa-miR-6525 |
| DBT | hsa-miR-5571-3p |
| DBT | hsa-miR-574-5p |
| DBT | hsa-miR-6690-5p |
| DBT | hsa-miR-659-3p |
| DBT | hsa-miR-7174-3p |
| DBT | hsa-miR-4324 |
| DBT | hsa-miR-8883 |
| DBT | hsa-miR-4740-3p |
| DBT | hsa-miR-6568-3p |
| DBT | hsa-miR-4763-5p |
| DBT | hsa-miR-376a-5p |
| DBT | hsa-miR-6847-5p |
| DBT | hsa-miR-4261 |
| DBT | hsa-miR-583 |
| DBT | hsa-miR-6126 |
| DBT | hsa-miR-7049-5p |
| DBT | hsa-miR-2115-3p |
| DBT | hsa-miR-1546-5p |
| DBT | hsa-miR-585-5p |
| DBT | hsa-miR-7215-3p |
| DBT | hsa-miR-1205 |
| DBT | hsa-miR-6529-5p |
| DBT | hsa-miR-4513 |
| DBT | hsa-miR-384 |
| DBT | hsa-miR-4418 |
| DBT | hsa-miR-548av-3p |
| DBT | hsa-miR-6544-5p |
| DBT | hsa-miR-203b-3p |
| DBT | hsa-miR-92b-5p |
| DBT | hsa-miR-549-3p |
| DBT | hsa-miR-3917 |
| DBT | hsa-miR-101-1-5p |
| DBT | hsa-miR-582-5p |
| DBT | hsa-miR-421 |
| DBT | hsa-miR-5583-5p |
| DBT | hsa-miR-8064 |
| DBT | hsa-miR-19b-5p |
| DBT | hsa-let-7a-2-3p |
| DBT | hsa-miR-2317 |
| DBT | hsa-miR-203a-5p |
| DBT | hsa-miR-2325b |
| DBT | hsa-miR-1182 |
| DBT | hsa-miR-587 |
| DBT | hsa-miR-2902 |
| DBT | hsa-miR-766-3p |
| DBT | hsa-miR-548h-3p |
| DBT | hsa-miR-2284d |
| DBT | hsa-miR-3922-3p |
| DBT | hsa-miR-551b-5p |
| DBT | hsa-miR-659-5p |
| DBT | hsa-miR-200ab-5p |
| DBT | hsa-miR-3672 |
| DBT | hsa-miR-7337-3p |
| DBT | hsa-miR-4443 |
| DBT | hsa-miR-1260a |
| DBT | hsa-miR-211-3p |
| DBT | hsa-miR-4800-5p |
| DBT | hsa-miR-4470 |
| DBT | hsa-miR-2308 |
| DBT | hsa-miR-548av-5p |
| DBT | hsa-miR-4520-2-3p |
| DBT | hsa-miR-5591-5p |
| DBT | hsa-miR-548cej-3p |
| DBT | hsa-miR-1544-3p |
| DBT | hsa-miR-527 |
| DBT | hsa-miR-6536 |
| DBT | hsa-miR-4642 |
| DBT | hsa-miR-362-5p |
| DBT | hsa-miR-4786-5p |
| DBT | hsa-miR-330-3p |
| DBT | hsa-miR-3924 |
| DBT | hsa-miR-6512-5p |
| DBT | hsa-miR-4527 |
| DBT | hsa-miR-7388a-3p |
| DBT | hsa-miR-514a-3p |
| DBT | hsa-miR-2285t |
| DBT | hsa-miR-4300 |
| DBT | hsa-miR-8821 |
| DBT | hsa-miR-7386j-3p |
| DBT | hsa-miR-4488 |
| DBT | hsa-miR-3064-3p |
| DBT | hsa-miR-1253 |
| DBT | hsa-miR-4694-3p |
| DBT | hsa-miR-2307 |
| DBT | hsa-miR-29a-3p |
| DBT | hsa-miR-2896 |
| DBT | hsa-miR-5003-3p |
| DBT | hsa-miR-1-3p |
| DBT | hsa-miR-25-3p |
| DBT | hsa-miR-126-5p |
| DBT | hsa-miR-3616-3p |
| DBT | hsa-miR-548z |
| DBT | hsa-miR-644a |
| DBT | hsa-miR-8820 |
| DBT | hsa-miR-5007-3p |
| DBT | hsa-miR-6754-5p |
| DBT | hsa-miR-493 |
| DBT | hsa-miR-3121-3p |
| DBT | hsa-miR-589-5p |
| DBT | hsa-miR-7515 |
| DBT | hsa-miR-4749-3p |
| DBT | hsa-miR-1262-3p |
| DBT | hsa-miR-4424 |
| DBT | hsa-miR-2285d |
| DBT | hsa-miR-106-5p |
| DBT | hsa-miR-512-3p |
| DBT | hsa-miR-498 |
| DBT | hsa-miR-372-3p |
| DBT | hsa-miR-3120-3p |
| DBT | hsa-miR-7398q-3p |
| DBT | hsa-miR-3975 |
| DBT | hsa-miR-883b-5p |
| DBT | hsa-miR-6809-3p |
| DBT | hsa-miR-8855 |
| DBT | hsa-miR-1304-5p |
| DBT | hsa-miR-3133 |
| DBT | hsa-miR-4473 |
| DBT | hsa-miR-2378 |
| DBT | hsa-miR-4678 |
| DBT | hsa-miR-569 |
| DBT | hsa-miR-145-5p |
| DBT | hsa-miR-1740-5p |
| DBT | hsa-miR-765 |
| DBT | hsa-miR-7035-5p |
| DBT | hsa-miR-584-5p |
| DBT | hsa-miR-2471-5p |
| DBT | hsa-miR-6826-5p |
| DBT | hsa-miR-6780a-3p |
| DBT | hsa-miR-135b-3p |
| DBT | hsa-miR-3911 |
| DBT | hsa-miR-616-3p |
| DBT | hsa-miR-8837 |
| DBT | hsa-miR-499b-3p |
| DBT | hsa-miR-3068-3p |
| DBT | hsa-miR-4475 |
| DBT | hsa-miR-517 |
| DBT | hsa-miR-6790-3p |
| DBT | hsa-miR-8082 |
| DBT | hsa-miR-8824 |
| DBT | hsa-miR-8800 |
| DBT | hsa-miR-365a-3p |
| DBT | hsa-miR-1231 |
| DBT | hsa-miR-582-5p |
| DBT | hsa-miR-4761-3p |
| DBT | hsa-miR-7398n-3p |
| DBT | hsa-miR-7158-5p |
| DBT | hsa-miR-6509-3p |
| DBT | hsa-miR-194-5p |
| DBT | hsa-miR-506-3p |
| DBT | hsa-miR-500a-5p |
| DBT | hsa-miR-3684 |
| DBT | hsa-miR-6923-5p |
| DBT | hsa-miR-7386k-3p |
| DBT | hsa-miR-567 |
| DBT | hsa-miR-214-3p |
| DBT | hsa-miR-22-3p |
| DBT | hsa-miR-4753-5p |
| DBT | hsa-miR-30e |
| DBT | hsa-miR-3679-3p |
| DBT | hsa-miR-1840 |
| DBT | hsa-miR-205-3p |
| DBT | hsa-miR-5572 |
| DBT | hsa-miR-6516-3p |
| DBT | hsa-miR-1296 |
| DBT | hsa-miR-1722-3p |
| DBT | hsa-miR-4680-3p |
| DBT | hsa-miR-124-3p.2 |
| DBT | hsa-miR-7001-3p |
| DBT | hsa-miR-6541 |
| DBT | hsa-miR-7300-3p |
| DBT | hsa-miR-8079 |
| DBT | hsa-miR-4778-3p |
| DBT | hsa-miR-2461-5p |
| DBT | hsa-miR-502a |
| DBT | hsa-miR-6515-3p |
| DBT | hsa-miR-4302 |
| DBT | hsa-miR-5089-5p |
| DBT | hsa-miR-4487 |
| DBT | hsa-miR-2294 |
| DBT | hsa-miR-4273 |
| DBT | hsa-miR-4708-3p |
| DBT | hsa-miR-195-5p |
| DBT | hsa-miR-302bd |
| DBT | hsa-miR-6803-5p |
| DBT | hsa-miR-7208-5p |
| DBT | hsa-miR-872-3p |
| DBT | hsa-miR-2284j |
| DBT | hsa-miR-3618-3p |
| DBT | hsa-miR-4664-5p |
| DBT | hsa-miR-3150a-3p |
| DBT | hsa-miR-1237-3p |
| DBT | hsa-miR-128-3p |
| DBT | hsa-miR-7320-5p |
| DBT | hsa-miR-429-5p |
| DBT | hsa-miR-1912-3p |
| DBT | hsa-miR-2376 |
| DBT | hsa-miR-377-3p |
| DBT | hsa-miR-101a-5p |
| DBT | hsa-miR-302a-5p |
| DBT | hsa-miR-451-5p |
| DBT | hsa-miR-328 |
| DBT | hsa-miR-2286 |
| DBT | hsa-miR-545-3p |
| DBT | hsa-miR-7386e-5p |
| DBT | hsa-miR-3925-5p |
| DBT | hsa-miR-2285z |
| DBT | hsa-miR-6726-5p |
| DBT | hsa-miR-363-5p |
| DBT | hsa-miR-7386i-3p |
| DBT | hsa-miR-4762-3p |
| DBT | hsa-miR-7241-5p |
| DBT | hsa-miR-202-3p |
| DBT | hsa-miR-3942-3p |
| DBT | hsa-miR-5197-3p |
| DBT | hsa-miR-7398b-3p |
| DBT | hsa-miR-3135a |
| DBT | hsa-miR-4794 |
| DBT | hsa-miR-3192-5p |
| DBT | hsa-miR-8070 |
| DBT | hsa-miR-329 |
| DBT | hsa-miR-7850-5p |
| DBT | hsa-miR-935 |
| DBT | hsa-miR-466 |
| DBT | hsa-miR-19 |
| DBT | hsa-miR-2285c |
| DBT | hsa-miR-7848-3p |
| DBT | hsa-miR-4764-5p |
| DBT | hsa-miR-539-5p |
| DBT | hsa-miR-7398a-3p |
| DBT | hsa-miR-6839-5p |
| DBT | hsa-miR-6131 |
| DBT | hsa-miR-548ad-5p |
| DBT | hsa-miR-4684-5p |
| DBT | hsa-miR-6735-3p |
| DBT | hsa-miR-3591-5p |
| DBT | hsa-miR-3078-5p |
| DBT | hsa-miR-2467-3p |
| DBT | hsa-miR-2278 |
| DBT | hsa-miR-4638-5p |
| DBT | hsa-miR-7703 |
| DBT | hsa-miR-7453-3p |
| DBT | hsa-miR-4780 |
| DBT | hsa-miR-520 |
| DBT | hsa-miR-1245a |
| DBT | hsa-miR-2409 |
| DBT | hsa-miR-2284ab |
| DBT | hsa-miR-191-5p |
| DBT | hsa-miR-6815-5p |
| DBT | hsa-miR-6376 |
| DBT | hsa-miR-1287-5p |
| DBT | hsa-miR-4520-3p |
| DBT | hsa-miR-561-5p |
| DBT | hsa-miR-6654-3p |
| DBT | hsa-miR-2284x |
| DBT | hsa-miR-7182-3p |
| DBT | hsa-miR-890-3p |
| DBT | hsa-miR-1245b-3p |
| DBT | hsa-miR-4288 |
| DBT | hsa-miR-2368-3p |
| DBT | hsa-miR-4260 |
| DBT | hsa-miR-6759-5p |
| DBT | hsa-miR-3612 |
| DBT | hsa-miR-7262a-5p |
| DBT | hsa-miR-7193-3p |
| DBT | hsa-miR-554 |
| DBT | hsa-miR-548w |
| DBT | hsa-miR-208-5p |
| DBT | hsa-miR-299-5p |
| DBT | hsa-miR-8867 |
| DBT | hsa-miR-4447 |
| DBT | hsa-miR-2444 |
| DBT | hsa-miR-3580-5p |
| DBT | hsa-miR-637 |
| DBT | hsa-miR-7155-5p |
| DBT | hsa-miR-8485 |
| DBT | hsa-miR-4280 |
| DBT | hsa-miR-8057 |
| DBT | hsa-miR-371-3p |
| DBT | hsa-miR-450a |
| DBT | hsa-miR-6884-3p |
| DBT | hsa-miR-4697-3p |
| DBT | hsa-miR-188-5p |
| DBT | hsa-miR-7175-3p |
| DBT | hsa-miR-671-5p |
| DBT | hsa-miR-4534 |
| DBT | hsa-miR-374c-3p |
| DBT | hsa-miR-4662a-3p |
| DBT | hsa-miR-6814-3p |
| DBT | hsa-miR-7371g-5p |
| DBT | hsa-miR-7167-3p |
| DBT | hsa-miR-4743-3p |
| DBT | hsa-miR-331-3p |
| DBT | hsa-miR-5094 |
| DBT | hsa-miR-760-3p |
| DBT | hsa-miR-552-5p |
| DBT | hsa-miR-342-3p |
| DBT | hsa-miR-411-5p.2 |
| DBT | hsa-miR-7678-3p |
| DBT | hsa-miR-5197-5p |
| DBT | hsa-miR-16-5p |
| DBT | hsa-miR-8805 |
| DBT | hsa-miR-1388-3p |
| DBT | hsa-miR-380 |
| DBT | hsa-miR-4481 |
| DBT | hsa-miR-7108-5p |
| DBT | hsa-miR-4430 |
| DBT | hsa-miR-3928-3p |
| DBT | hsa-miR-672-3p |
| DBT | hsa-miR-5009-3p |
| DBT | hsa-miR-7371j-5p |
| DBT | hsa-miR-494-5p |
| DBT | hsa-miR-8814 |
| DBT | hsa-miR-6740-5p |
| DBT | hsa-miR-210-3p |
| DBT | hsa-miR-2284-5p |
| DBT | hsa-miR-3194-5p |
| DBT | hsa-miR-2429 |
| DBT | hsa-miR-5001-3p |
| DBT | hsa-miR-519d-5p |
| DBT | hsa-miR-633 |
| DBT | hsa-miR-519e-3p |
| DBT | hsa-miR-4760-5p |
| DBT | hsa-miR-92a-2-5p |
| DBT | hsa-miR-3616-5p |
| DBT | hsa-miR-4524b-3p |
| DBT | hsa-miR-8791 |
| DBT | hsa-miR-4753-3p |
| DBT | hsa-miR-8076 |
| DBT | hsa-miR-1262-5p |
| DBT | hsa-miR-570-3p |
| DBT | hsa-miR-582-3p |
| DBT | hsa-miR-423-5p |
| DBT | hsa-miR-548au-5p |
| DBT | hsa-miR-134-3p |
| DBT | hsa-miR-708-3p |
| DBT | hsa-miR-4666a-5p |
| DBT | hsa-miR-939-5p |
| DBT | hsa-miR-6128 |
| DBT | hsa-miR-7156-3p |
| DBT | hsa-miR-302 |
| DBT | hsa-miR-7158-3p |
| DBT | hsa-miR-3140-3p |
| DBT | hsa-miR-1655-5p |
| DBT | hsa-miR-3127-5p |
| DBT | hsa-miR-5691 |
| DBT | hsa-miR-6858-5p |
| DBT | hsa-miR-345-5p |
| DBT | hsa-miR-140-3p |
| DBT | hsa-miR-4643 |
| DBT | hsa-miR-6828-5p |
| DBT | hsa-miR-7186-5p |
| DBT | hsa-miR-944 |
| DBT | hsa-miR-4786-3p |
| DBT | hsa-miR-7045-3p |
| DBT | hsa-miR-1236-3p |
| DBT | hsa-miR-4691-5p |
| DBT | hsa-miR-643 |
| DBT | hsa-miR-8861 |
| DBT | hsa-miR-761 |
| DBT | hsa-miR-2372 |
| DBT | hsa-miR-6837-3p |
| DBT | hsa-miR-766-3p |
| DBT | hsa-miR-3671 |
| DBT | hsa-miR-6857-3p |
| DBT | hsa-miR-4789-3p |
| DBT | hsa-miR-4695-5p |
| DBT | hsa-miR-802 |
| DBT | hsa-miR-1273e |
| DBT | hsa-miR-3533 |
| DBT | hsa-miR-599-3p |
| DBT | hsa-miR-3649 |
| DBT | hsa-miR-6133 |
| DBT | hsa-miR-1238-3p |
| DBT | hsa-miR-4653-3p |
| DBT | hsa-miR-192-5p |
| DBT | hsa-miR-6077 |
| DBT | hsa-miR-642b-5p |
| DBT | hsa-miR-3137 |
| DBT | hsa-miR-222-3p |
| DBT | hsa-miR-6812-5p |
| DBT | hsa-miR-516a-3p |
| DBT | hsa-miR-29c-5p |
| DBT | hsa-miR-3194-3p |
| DBT | hsa-miR-1912 |
| DBT | hsa-miR-941 |
| DBT | hsa-miR-6890-3p |
| DBT | hsa-miR-18-5p |
| DBT | hsa-miR-6872-3p |
| DBT | hsa-miR-660-3p |
| DBT | hsa-miR-4439 |
| DBT | hsa-miR-2343 |
| DBT | hsa-miR-219b-3p |
| DBT | hsa-miR-6761-5p |
| DBT | hsa-miR-4722-3p |
| DBT | hsa-miR-3921 |
| DBT | hsa-miR-7843-5p |
| DBT | hsa-miR-4659b-5p |
| DBT | hsa-miR-6827-5p |
| DBT | hsa-miR-6418-5p |
| DBT | hsa-miR-1304-5p |
| DBT | hsa-miR-4437 |
| DBT | hsa-miR-3124-3p |
| DBT | hsa-miR-301a-5p |
| DBT | hsa-miR-3574 |
| DBT | hsa-miR-520gh |
| DBT | hsa-miR-1225 |
| DBT | hsa-miR-5000-5p |
| DBT | hsa-miR-5196-5p |
| DBT | hsa-miR-2110 |
| DBT | hsa-miR-18-3p |
| DBT | hsa-miR-2454-3p |
| DBT | hsa-miR-641 |
| DBT | hsa-miR-1976 |
| DBT | hsa-miR-767-3p |
| DBT | hsa-miR-361-5p |
| DBT | hsa-miR-5006-5p |
| DBT | hsa-miR-4528 |
| DBT | hsa-miR-450a-1-3p |
| DBT | hsa-miR-4434 |
| DBT | hsa-miR-143-5p |
| DBT | hsa-miR-7185-3p |
| DBT | hsa-miR-6769a-5p |
| DBT | hsa-miR-669p-3p |
| DBT | hsa-miR-3136-5p |
| DBT | hsa-miR-5101 |
| DBT | hsa-miR-579-3p |
| DBT | hsa-miR-6819-5p |
| DBT | hsa-miR-873-3p |
| DBT | hsa-miR-20b-3p |
| DBT | hsa-miR-6815-3p |
| DBT | hsa-miR-204-5p |
| DBT | hsa-miR-7a-1-3p |
| DBT | hsa-miR-125b-2-3p |
| DBT | hsa-miR-350-3p |
| DBT | hsa-miR-2453 |
| DBT | hsa-miR-5580-5p |
| DBT | hsa-miR-2352 |
| DBT | hsa-miR-760 |
| DBT | hsa-miR-6852-5p |
| DBT | hsa-miR-34b-3p |
| DBT | hsa-miR-6862-5p |
| DBT | hsa-miR-146a-3p |
| DBT | hsa-miR-7347-3p |
| DBT | hsa-miR-6511-5p |
| DBT | hsa-miR-29b-1-5p |
| DBT | hsa-miR-361-3p |
| DBT | hsa-miR-4635 |
| DBT | hsa-miR-5692a |
| DBT | hsa-miR-411a |
| DBT | hsa-miR-1285-5p |
| DBT | hsa-miR-6526 |
| DBT | hsa-miR-3660 |
| DBT | hsa-miR-95-5p |
| DBT | hsa-miR-548v |
| DBT | hsa-miR-5010-3p |
| DBT | hsa-miR-494-3p |
| DBT | hsa-miR-3928-5p |
| DBT | hsa-miR-4679 |
| DBT | hsa-miR-2484 |
| DBT | hsa-miR-766-5p |
| DBT | hsa-miR-4659a-3p |
| DBT | hsa-miR-193b-5p |
| DBT | hsa-miR-5697-5p |
| DBT | hsa-miR-6843-3p |
| DBT | hsa-miR-451b |
| DBT | hsa-miR-376abd |
| DBT | hsa-miR-17-3p |
| DBT | hsa-miR-5585-3p |
| DBT | hsa-miR-648 |
| DBT | hsa-miR-3692-3p |
| DBT | hsa-miR-544a |
| DBT | hsa-miR-7398s-5p |
| DBT | hsa-miR-3087-3p |
| DBT | hsa-miR-5002-3p |
| DBT | hsa-miR-2284g |
| DBT | hsa-miR-2284u |
| DBT | hsa-miR-1737 |
| DBT | hsa-miR-548j-5p |
| DBT | hsa-miR-502b |
| DBT | hsa-miR-4323 |
| DBT | hsa-miR-6764-5p |
| DBT | hsa-miR-17 |
| DBT | hsa-miR-3074-5p |
| DBT | hsa-miR-6920-5p |
| DBT | hsa-miR-2957 |
| DBT | hsa-miR-101-3p.2 |
| DBT | hsa-miR-29 |
| DBT | hsa-miR-497-3p |
| DBT | hsa-miR-29a-3p |
| DBT | hsa-miR-3158-3p |
| DBT | hsa-miR-218-5p |
| DBT | hsa-miR-8817 |
| DBT | hsa-miR-3115 |
| DBT | hsa-miR-625-3p |
| DBT | hsa-miR-5690 |
| DBT | hsa-miR-576-5p |
| DBT | hsa-miR-499-5p |
| DBT | hsa-miR-200a-3p |
| DBT | hsa-miR-6809-5p |
| DBT | hsa-miR-515-3p |
| DBT | hsa-miR-4653-5p |
| DBT | hsa-miR-217 |
| DBT | hsa-miR-3162-5p |
| DBT | hsa-miR-7200-3p |
| DBT | hsa-miR-301 |
| DBT | hsa-miR-7008-3p |
| DBT | hsa-miR-6821-5p |
| DBT | hsa-miR-3934-5p |
| DBT | hsa-miR-1236-3p |
| DBT | hsa-miR-6748-5p |
| DBT | hsa-miR-219-3p |
| DBT | hsa-miR-8879 |
| DBT | hsa-miR-17-5p |
| DBT | hsa-miR-216-3p |
| DBT | hsa-miR-4666b |
| DBT | hsa-miR-5009-5p |
| DBT | hsa-miR-6337 |
| DBT | hsa-miR-383-5p.2 |
| DBT | hsa-miR-7150 |
| DBT | hsa-miR-1240 |
| DBT | hsa-miR-2290 |
| DBT | hsa-miR-6513-3p |
| DBT | hsa-miR-212-5p |
| DBT | hsa-miR-29a-5p |
| DBT | hsa-miR-499a-3p |
| DBT | hsa-miR-4421 |
| DBT | hsa-miR-8893 |
| DBT | hsa-miR-296-3p |
| DBT | hsa-miR-3691-3p |
| DBT | hsa-miR-6951-3p |
| DBT | hsa-miR-433 |
| DBT | hsa-miR-495-5p |
| DBT | hsa-miR-197-5p |
| DBT | hsa-miR-6812-3p |
| DBT | hsa-miR-7160-5p |
| DBT | hsa-miR-4659b-3p |
| DBT | hsa-miR-6856-3p |
| DBT | hsa-miR-7852-3p |
| DBT | hsa-miR-1970 |
| DBT | hsa-miR-7651-3p |
| DBT | hsa-miR-7392-3p |
| DBT | hsa-miR-877-3p |
| DBT | hsa-miR-451a |
| DBT | hsa-miR-4729 |
| DBT | hsa-miR-26-5p |
| DBT | hsa-miR-7189-3p |
| DBT | hsa-miR-378-5p |
| DBT | hsa-miR-7159-5p |
| DBT | hsa-miR-3473g |
| DBT | hsa-miR-520c-3p |
| DBT | hsa-miR-6762-3p |
| DBT | hsa-miR-4520-5p |
| DBT | hsa-miR-3150b-3p |
| DBT | hsa-miR-6875-3p |
| DBT | hsa-miR-8065 |
| DBT | hsa-miR-501-3p |
| DBT | hsa-miR-4649-3p |
| DBT | hsa-miR-1296-3p |
| DBT | hsa-miR-4772-3p |
| DBT | hsa-miR-515-5p |
| DBT | hsa-miR-8810 |
| DBT | hsa-miR-892b |
| DBT | hsa-miR-7168-3p |
| DBT | hsa-miR-3965 |
| DBT | hsa-miR-4733-5p |
| DBT | hsa-miR-1276 |
| DBT | hsa-miR-3654 |
| DBT | hsa-miR-7243-3p |
| DBT | hsa-miR-7082-3p |
| DBT | hsa-miR-6818-5p |
| DBT | hsa-miR-627-5p |
| DBT | hsa-miR-504-3p |
| DBT | hsa-miR-6129 |
| DBT | hsa-miR-6878-3p |
| DBT | hsa-miR-20 |
| DBT | hsa-miR-4795-3p |
| DBT | hsa-miR-2323 |
| DBT | hsa-miR-6965-3p |
| DBT | hsa-miR-1251 |
| DBT | hsa-miR-6880-5p |
| DBT | hsa-miR-5700 |
| DBT | hsa-miR-1972 |
| DBT | hsa-miR-8827 |
| DBT | hsa-miR-2319 |
| DBT | hsa-miR-2331-3p |
| DBT | hsa-miR-526b-5p |
| DBT | hsa-miR-7385e-3p |
| DBT | hsa-miR-8826 |
| DBT | hsa-miR-340-5p |
| DBT | hsa-miR-3688-3p |
| DBT | hsa-miR-1284 |
| DBT | hsa-miR-8083 |
| DBT | hsa-miR-2888 |
| DBT | hsa-miR-6833-5p |
| DBT | hsa-miR-548y |
| DBT | hsa-miR-20-3p |
| DBT | hsa-miR-4650-5p |
| DBT | hsa-miR-6792-5p |
| DBT | hsa-miR-8117 |
| DBT | hsa-miR-4731-5p |
| DBT | hsa-miR-3664-5p |
| DBT | hsa-miR-2395 |
| DBT | hsa-miR-125 |
| DBT | hsa-miR-5003-5p |
| DBT | hsa-miR-4539 |
| DBT | hsa-miR-149-5p |
| DBT | hsa-miR-155-5p |
| DBT | hsa-miR-2345 |
| DBT | hsa-miR-4803 |
| DBT | hsa-miR-2285af |
| DBT | hsa-miR-186-5p |
| DBT | hsa-miR-8081 |
| DBT | hsa-miR-677 |
| DBT | hsa-miR-196-5p |
| DBT | hsa-miR-598-3p |
| DBT | hsa-miR-3688-5p |
| DBT | hsa-miR-7157-5p |
| DBT | hsa-miR-212-3p |
| DBT | hsa-miR-3127-5p |
| DBT | hsa-miR-6888-5p |
| DBT | hsa-miR-7202-3p |
| DBT | hsa-miR-187-3p |
| DBT | hsa-miR-6500-3p |
| DBT | hsa-miR-487a |
| DBT | hsa-miR-449c-3p |
| DBT | hsa-miR-4483 |
| DBT | hsa-miR-367-3p |
| DBT | hsa-miR-4312 |
| DBT | hsa-miR-95 |
| DBT | hsa-miR-1584-5p |
| DBT | hsa-miR-4773 |
| DBT | hsa-miR-30c-3p |
| DBT | hsa-miR-5584-3p |
| DBT | hsa-miR-6734-3p |
| DBT | hsa-miR-6797-5p |
| DBT | hsa-miR-3064-5p |
| DBT | hsa-miR-4632-5p |
| DBT | hsa-miR-671-5p |
| DBT | hsa-miR-496-5p |
| DBT | hsa-miR-3123 |
| DBT | hsa-miR-7278-5p |
| DBT | hsa-miR-7186-3p |
| DBT | hsa-miR-8908-5p |
| DBT | hsa-miR-7324-3p |
| DBT | hsa-miR-2891 |
| DBT | hsa-miR-6753-3p |
| DBT | hsa-miR-664 |
| DBT | hsa-miR-142 |
| DBT | hsa-miR-7178-3p |
| DBT | hsa-miR-924 |
| DBT | hsa-miR-9-3-3p |
| DBT | hsa-miR-1837 |
| DBT | hsa-miR-429-3p |
| DBT | hsa-miR-651-3p |
| DBT | hsa-miR-684 |
| DBT | hsa-miR-3919 |
| DBT | hsa-miR-323 |
| DBT | hsa-miR-6760-5p |
| DBT | hsa-miR-200c-5p |
| DBT | hsa-miR-301-3p |
| DBT | hsa-miR-769-5p |
| DBT | hsa-miR-485-5p |
| DBT | hsa-miR-4677-5p |
| DBT | hsa-miR-34c-3p |
| DBT | hsa-miR-205-5p |
| DBT | hsa-miR-3978 |
| DBT | hsa-miR-8077 |
| DBT | hsa-miR-7153-3p |
| DBT | hsa-miR-548ai |
| DBT | hsa-miR-384-5p |
| DBT | hsa-miR-628-5p |
| DBT | hsa-miR-3578 |
| DBT | hsa-miR-216b-5p |
| DBT | hsa-miR-548aef |
| DBT | hsa-miR-548as-5p |
| DBT | hsa-miR-2450bd |
| DBT | hsa-miR-548f-3p |
| DBT | hsa-miR-465c-5p |
| DBT | hsa-miR-3611 |
| DBT | hsa-miR-1641 |
| DBT | hsa-miR-8812 |
| DBT | hsa-miR-892-3p |
| DBT | hsa-miR-7087-3p |
| DBT | hsa-miR-7251-3p |
| DBT | hsa-miR-4289 |
| DBT | hsa-miR-942-3p |
| DBT | hsa-miR-2909 |
| DBT | hsa-miR-3650 |
| DBT | hsa-miR-509-3-5p |
| DBT | hsa-miR-4738-3p |
| DBT | hsa-miR-6727-3p |
| DBT | hsa-miR-7385f-3p |
| DBT | hsa-miR-654-5p |
| DBT | hsa-miR-6793-3p |
| DBT | hsa-miR-1227-3p |
| DBT | hsa-miR-200b-5p |
| DBT | hsa-miR-1691 |
| DBT | hsa-miR-6777-3p |
| DBT | hsa-miR-6715a-3p |
| DBT | hsa-let-7a-3p |
| DBT | hsa-miR-548b-5p |
| DBT | hsa-miR-4490 |
| DBT | hsa-miR-1295a |
| DBT | hsa-miR-34a-3p |
| DBT | hsa-miR-1278 |
| DBT | hsa-miR-7194-5p |
| DBT | hsa-miR-548am-5p |
| DBT | hsa-miR-6384 |
| DBT | hsa-miR-4758-5p |
| DBT | hsa-miR-525-5p |
| DBT | hsa-miR-6879-3p |
| DBT | hsa-miR-519-3p |
| DBT | hsa-miR-767 |
| DBT | hsa-miR-3614-5p |
| DBT | hsa-miR-542-3p |
| DBT | hsa-miR-8833 |
| DBT | hsa-miR-7165-3p |
| DBT | hsa-miR-204-5p |
| DBT | hsa-miR-548l |
| DBT | hsa-miR-943 |
| DBT | hsa-miR-3173-3p |
| DBT | hsa-miR-6782-3p |
| DBT | hsa-miR-135-5p |
| DBT | hsa-miR-3610 |
| DBT | hsa-miR-302d-5p |
| DBT | hsa-miR-145-5p |
| DBT | hsa-miR-4441 |
| DBT | hsa-miR-4254 |
| DBT | hsa-miR-7201-3p |
| DBT | hsa-miR-30b-3p |
| DBT | hsa-miR-6759-3p |
| DBT | hsa-miR-6829-3p |
| DBT | hsa-miR-5121 |
| DBT | hsa-miR-320a |
| DBT | hsa-miR-2377 |
| DBT | hsa-miR-550a-3p |
| DBT | hsa-miR-6123 |
| DBT | hsa-miR-2385-5p |
| DBT | hsa-miR-506-3p |
| DBT | hsa-let-7c-3p |
| DBT | hsa-miR-2285j |
| DBT | hsa-miR-7360-3p |
| DBT | hsa-miR-6769b-3p |
| DBT | hsa-miR-7383-5p |
| DBT | hsa-miR-411-5p |
| DBT | hsa-miR-2426 |
| DBT | hsa-miR-3151-3p |
| DBT | hsa-miR-6892-5p |
| DBT | hsa-miR-4422 |
| DBT | hsa-miR-4711-3p |
| DBT | hsa-miR-31-5p |
| DBT | hsa-miR-6623-3p |
| DBT | hsa-miR-1200 |
| DBT | hsa-miR-876-5p |
| DBT | hsa-miR-675-3p |
| DBT | hsa-miR-4478 |
| DBT | hsa-miR-501-5p |
| DBT | hsa-miR-2408 |
| DBT | hsa-miR-4782-5p |
| DBT | hsa-miR-6793-5p |
| DBT | hsa-miR-4535 |
| DBT | hsa-miR-545-5p |
| DBT | hsa-miR-1288-5p |
| DBT | hsa-miR-3065-5p |
| DBT | hsa-miR-1814 |
| DBT | hsa-miR-8058 |
| DBT | hsa-miR-6986-3p |
| DBT | hsa-miR-6737-5p |
| DBT | hsa-miR-100-3p |
| DBT | hsa-miR-4705 |
| DBT | hsa-miR-6861-5p |
| DBT | hsa-miR-2477 |
| DBT | hsa-miR-4775 |
| DBT | hsa-miR-34 |
| DBT | hsa-miR-6858-3p |
| DBT | hsa-miR-6817-5p |
| DBT | hsa-miR-7673-3p |
| DBT | hsa-miR-27 |
| DBT | hsa-miR-4674 |
| DBT | hsa-miR-2401 |
| DBT | hsa-miR-654-3p |
| DBT | hsa-miR-6952-5p |
| DBT | hsa-miR-8905 |
| DBT | hsa-miR-4510 |
| DBT | hsa-miR-570-3p |
| DBT | hsa-miR-1938 |
| DBT | hsa-miR-2417 |
| DBT | hsa-miR-4452 |
| DBT | hsa-miR-4279 |
| DBT | hsa-miR-1252 |
| DBT | hsa-miR-32-5p |
| DBT | hsa-miR-2461-3p |
| DBT | hsa-miR-631 |
| DBT | hsa-miR-330-3p.2 |
| DBT | hsa-miR-6748-3p |
| DBT | hsa-miR-8060 |
| DBT | hsa-miR-2366 |
| DBT | hsa-miR-3122 |
| DBT | hsa-miR-676-3p |
| DBT | hsa-miR-183-5p |
| DBT | hsa-miR-5693 |
| DBT | hsa-miR-539-3p |
| DBT | hsa-miR-107 |
| DBT | hsa-miR-7107-5p |
| DBT | hsa-miR-7280-5p |
| DBT | hsa-miR-1982-5p |
| DBT | hsa-miR-548an |
| DBT | hsa-miR-1226-5p |
| DBT | hsa-miR-412-3p |
| DBT | hsa-miR-4524a-3p |
| DBT | hsa-miR-881-3p |
| DBT | hsa-miR-491-5p |
| DBT | hsa-miR-5680 |
| DBT | hsa-miR-494 |
| DBT | hsa-miR-532-3p |
| DBT | hsa-miR-4711-5p |
| DBT | hsa-miR-4713-5p |
| DBT | hsa-miR-2371 |
| DBT | hsa-miR-185-5p |
| DBT | hsa-miR-2328-5p |
| DBT | hsa-miR-4769-3p |
| DBT | hsa-miR-6922-3p |
| DBT | hsa-miR-325-3p |
| DBT | hsa-miR-6773-3p |
| DBT | hsa-miR-6779-3p |
| DBT | hsa-miR-1233-3p |
| DBT | hsa-miR-1955-3p |
| DBT | hsa-miR-4774-3p |
| DBT | hsa-miR-1540 |
| DBT | hsa-miR-4537 |
| DBT | hsa-miR-8071 |
| DBT | hsa-miR-210 |
| DBT | hsa-miR-6865-5p |
| DBT | hsa-miR-885 |
| DBT | hsa-miR-3661 |
| DBT | hsa-miR-4639-5p |
| DBT | hsa-miR-4719 |
| DBT | hsa-miR-93-5p |
| DBT | hsa-miR-3577-5p |
| DBT | hsa-miR-2393 |
| DBT | hsa-miR-378-3p |
| DBT | hsa-miR-936 |
| DBT | hsa-miR-7029-3p |
| DBT | hsa-miR-920 |
| DBT | hsa-miR-1270 |
| DBT | hsa-miR-450b |
| DBT | hsa-miR-758-3p |
| DBT | hsa-miR-6778-5p |
| DBT | hsa-miR-6507-5p |
| DBT | hsa-miR-2425-5p |
| DBT | hsa-miR-8807 |
| DBT | hsa-miR-7263-3p |
| DBT | hsa-miR-4314 |
| DBT | hsa-miR-5689 |
| DBT | hsa-miR-7156-5p |
| DBT | hsa-miR-7112-5p |
| DBT | hsa-miR-6873-5p |
| DBT | hsa-miR-7251-5p |
| DBT | hsa-miR-548ba |
| DBT | hsa-miR-105-5p |
| DBT | hsa-miR-2340 |
| DBT | hsa-miR-424-5p |
| DBT | hsa-miR-4695-3p |
| DBT | hsa-miR-3910 |
| DBT | hsa-miR-6979-5p |
| DBT | hsa-miR-656 |
| DBT | hsa-miR-8870 |
| DBT | hsa-miR-3929 |
| DBT | hsa-miR-3958 |
| DBT | hsa-miR-7854-3p |
| DBT | hsa-miR-6886-3p |
| DBT | hsa-miR-4750-3p |
| DBT | hsa-miR-298 |
| DBT | hsa-miR-3689-3p |
| DBT | hsa-miR-181-3p |
| DBT | hsa-miR-153-3p |
| DBT | hsa-miR-609 |
| DBT | hsa-miR-4714-5p |
| DBT | hsa-miR-346 |
| DBT | hsa-miR-412 |
| DBT | hsa-miR-2469 |
| DBT | hsa-miR-507 |
| DBT | hsa-miR-590-3p |
| DBT | hsa-miR-2284i |
| DBT | hsa-miR-502-3p |
| DBT | hsa-miR-6723-5p |
| DBT | hsa-miR-627 |
| DBT | hsa-miR-30e-3p |
| DBT | hsa-miR-2370-3p |
| DBT | hsa-miR-107-3p |
| DBT | hsa-miR-192-5p |
| DBT | hsa-miR-140-5p |
| DBT | hsa-miR-3613-3p |
| DBT | hsa-miR-381-3p |
| DBT | hsa-miR-4716-5p |
| DBT | hsa-miR-7666-3p |
| DBT | hsa-miR-891a-3p |
| DBT | hsa-miR-342-5p |
| DBT | hsa-miR-103-2-5p |
| DBT | hsa-miR-302-3p |
| DBT | hsa-miR-98-5p |
| DBT | hsa-miR-6952-3p |
| DBT | hsa-miR-422 |
| DBT | hsa-miR-338-5p |
| DBT | hsa-miR-8906 |
| DBT | hsa-miR-7257-3p |
| DBT | hsa-miR-380-3p |
| DBT | hsa-miR-2296 |
| DBT | hsa-miR-2300-5p |
| DBT | hsa-miR-516b-3p |
| DBT | hsa-miR-148 |
| DBT | hsa-miR-1903 |
| DBT | hsa-miR-5096 |
| DBT | hsa-miR-2320-5p |
| DBT | hsa-miR-2434 |
| DBT | hsa-miR-7362-3p |
| DBT | hsa-miR-2402 |
| DBT | hsa-miR-217-3p |
| DBT | hsa-miR-3675-3p |
| DBT | hsa-miR-6747-3p |
| DBT | hsa-miR-6718-5p |
| DBT | hsa-miR-4477a |
| DBT | hsa-miR-344h-3p |
| DBT | hsa-miR-3108-5p |
| DBT | hsa-miR-363-3p |
| DBT | hsa-miR-4668-5p |
| DBT | hsa-miR-515 |
| DBT | hsa-miR-6504-3p |
| DBT | hsa-miR-4792 |
| DBT | hsa-miR-29-3p |
| DBT | hsa-miR-4645-5p |
| DBT | hsa-miR-653 |
| DBT | hsa-miR-548d-3p |
| DBT | hsa-miR-6807-5p |
| DBT | hsa-miR-4728-5p |
| DBT | hsa-miR-6716-5p |
| DBT | hsa-miR-2437 |
| DBT | hsa-miR-8865 |
| DBT | hsa-miR-2285s |
| DBT | hsa-miR-6125 |
| DBT | hsa-miR-4646-5p |
| DBT | hsa-miR-4307 |
| DBT | hsa-miR-4252 |
| DBT | hsa-miR-1246 |
| DBT | hsa-miR-130 |
| DBT | hsa-miR-1643-3p |
| DBT | hsa-miR-6371 |
| DBT | hsa-miR-7386o-3p |
| DBT | hsa-miR-6802-5p |
| DBT | hsa-miR-148-5p |
| DBT | hsa-miR-6729-3p |
| DBT | hsa-miR-548i |
| DBT | hsa-miR-1283a |
| DBT | hsa-miR-7386a-3p |
| DBT | hsa-miR-655-3p |
| DBT | hsa-miR-496.2 |
| DBT | hsa-miR-3174 |
| DBT | hsa-miR-6751-3p |
| DBT | hsa-miR-151 |
| DBT | hsa-miR-7162-5p |
| DBT | hsa-miR-1252-3p |
| DBT | hsa-miR-7198-3p |
| DBT | hsa-miR-376c |
| DBT | hsa-miR-7025-3p |
| DBT | hsa-miR-154a |
| DBT | hsa-miR-337-3p |
| DBT | hsa-miR-6335 |
| DBT | hsa-miR-4451 |
| DBT | hsa-miR-1273d |
| DBT | hsa-miR-4444 |
| DBT | hsa-miR-7849-3p |
| DBT | hsa-miR-3914 |
| DBT | hsa-miR-676-3p |
| DBT | hsa-miR-128 |
| DBT | hsa-miR-429 |
| DBT | hsa-miR-7190-3p |
| DBT | hsa-miR-3158-5p |
| DBT | hsa-miR-4760-3p |
| DBT | hsa-miR-3151 |
| DBT | hsa-miR-484 |
| DBT | hsa-miR-206 |
| DBT | hsa-miR-4791 |
| DBT | hsa-miR-4682 |
| DBT | hsa-miR-3148 |
| DBT | hsa-miR-146 |
| DBT | hsa-miR-7171-5p |
| DBT | hsa-miR-2116-5p |
| DBT | hsa-miR-645 |
| DBT | hsa-miR-3159 |
| DBT | hsa-miR-8874 |
| DBT | hsa-miR-4438 |
| DBT | hsa-miR-6828-3p |
| DBT | hsa-miR-3167-3p |
| DBT | hsa-miR-4742-3p |
| DBT | hsa-miR-1237-5p |
| DBT | hsa-miR-6770-5p |
| DBT | hsa-miR-6986-5p |
| DBT | hsa-miR-4724-3p |
| DBT | hsa-miR-1914-5p |
| DBT | hsa-miR-329-3p |
| DBT | hsa-miR-526 |
| DBT | hsa-miR-4680 |
| DBT | hsa-miR-669bf-3p |
| DBT | hsa-miR-600 |
| DBT | hsa-miR-1238-5p |
| DBT | hsa-miR-593-3p |
| DBT | hsa-miR-376ab |
| DBT | hsa-miR-4325 |
| DBT | hsa-miR-3129-3p |
| DBT | hsa-miR-136-5p |
| DBT | hsa-miR-378j |
| DBT | hsa-miR-2312 |
| DBT | hsa-miR-3121-3p |
| DBT | hsa-miR-7107-3p |
| DBT | hsa-miR-548ae-5p |
| DBT | hsa-miR-640 |
| DBT | hsa-miR-3652 |
| DBT | hsa-miR-2335 |
| DBT | hsa-miR-632 |
| DBT | hsa-miR-22-5p |
| DBT | hsa-miR-4423-3p |
| DBT | hsa-miR-6866-5p |
| DBT | hsa-miR-578 |
| DBT | hsa-miR-497-5p |
| DBT | hsa-miR-489-3p |
| DBT | hsa-miR-6871-5p |
| DBT | hsa-miR-2355-3p |
| DBT | hsa-miR-2464-5p |
| DBT | hsa-miR-3140-5p |
| DBT | hsa-miR-224-5p |
| DBT | hsa-miR-6889-3p |
| DBT | hsa-miR-872 |
| DBT | hsa-miR-4755-3p |
| DBT | hsa-miR-326 |
| DBT | hsa-miR-514b-5p |
| DBT | hsa-miR-203 |
| DBT | hsa-miR-6832-5p |
| DBT | hsa-miR-466i-3p |
| DBT | hsa-miR-6883-5p |
| DBT | hsa-miR-3176 |
| DBT | hsa-miR-154-5p |
| DBT | hsa-miR-3916 |
| DBT | hsa-miR-6857-5p |
| DBT | hsa-miR-132-5p |
| DBT | hsa-miR-7292-5p |
| DBT | hsa-miR-411-3p |
| DBT | hsa-miR-5615-5p |
| DBT | hsa-miR-302a-3p |
| DBT | hsa-miR-2355-5p |
| DBT | hsa-miR-7177-3p |
| DBT | hsa-miR-6872-5p |
| DBT | hsa-miR-221-3p |
| DBT | hsa-miR-216a |
| DBT | hsa-miR-8908-3p |
| DBT | hsa-miR-200ac-5p |
| DBT | hsa-miR-608 |
| DBT | hsa-miR-452-5p |
| DBT | hsa-miR-376e |
| DBT | hsa-miR-548x-3p |
| DBT | hsa-miR-4448 |
| DBT | hsa-miR-300-3p |
| DBT | hsa-miR-665 |
| DBT | hsa-miR-5093 |
| DBT | hsa-miR-2480 |
| DBT | hsa-miR-518g-3p |
| DBT | hsa-miR-215-5p |
| DBT | hsa-miR-544a |
| DBT | hsa-miR-328-3p |
| DBT | hsa-miR-7205-3p |
| DBT | hsa-miR-362-5p |
| DBT | hsa-miR-132-3p |
| DBT | hsa-miR-34-5p |
| DBT | hsa-miR-520g-5p |
| DBT | hsa-miR-1306-5p |
| DBT | hsa-miR-4724-5p |
| DBT | hsa-miR-510-3p |
| DBT | hsa-miR-16-3p |
| DBT | hsa-miR-2412 |
| DBT | hsa-miR-7398u-3p |
| DBT | hsa-miR-556-5p |
| DBT | hsa-miR-693-3p |
| DBT | hsa-miR-4660 |
| DBT | hsa-miR-6074 |
| DBT | hsa-miR-6933-3p |
| DBT | hsa-miR-2333 |
| DBT | hsa-miR-299-3p |
| DBT | hsa-miR-500b-3p |
| DBT | hsa-miR-34b-3p |
| DBT | hsa-miR-124-3p |
| DBT | hsa-miR-6848-5p |
| DBT | hsa-miR-6740-3p |
| DBT | hsa-miR-6736-3p |
| DBT | hsa-miR-7334-3p |
| DBT | hsa-miR-3927-5p |
| DBT | hsa-miR-638 |
| DBT | hsa-miR-573 |
| DBT | hsa-miR-6631-5p |
| DBT | hsa-miR-511 |
| DBT | hsa-miR-323a-5p |
| DBT | hsa-miR-501-5p |
| DBT | hsa-miR-4266 |
| DBT | hsa-miR-2464-3p |
| DBT | hsa-miR-4761-5p |
| DBT | hsa-miR-19-3p |
| DBT | hsa-miR-5195-5p |
| DBT | hsa-miR-466n-3p |
| DBT | hsa-miR-4524a-5p |
| DBT | hsa-miR-4677-3p |
| DBT | hsa-miR-3934-3p |
| DBT | hsa-miR-4256 |
| DBT | hsa-miR-6758-3p |
| DBT | hsa-miR-7151-3p |
| DBT | hsa-miR-2455 |
| DBT | hsa-miR-3613a |
| DBT | hsa-miR-3973 |
| DBT | hsa-miR-6814-5p |
| DBT | hsa-miR-2115-5p |
| DBT | hsa-miR-3577-3p |
| DBT | hsa-miR-409a |
| DBT | hsa-miR-669b-5p |
| DBT | hsa-miR-2901 |
| DBT | hsa-miR-618 |
| DBT | hsa-miR-7b-3p |
| DBT | hsa-miR-6732-5p |
| DBT | hsa-miR-7088-5p |
| DBT | hsa-miR-1835 |
| DBT | hsa-miR-4693-3p |
| DBT | hsa-miR-2397-5p |
| DBT | hsa-miR-3675-5p |
| DBT | hsa-miR-212-3p |
| DBT | hsa-miR-1194 |
| DBT | hsa-miR-92b-3p |
| DBT | hsa-miR-673-5p |
| DBT | hsa-miR-7163-5p |
| DBT | hsa-miR-550b-2-5p |
| DBT | hsa-miR-18 |
| DBT | hsa-miR-2353 |
| DBT | hsa-miR-1-3p |
| DBT | hsa-miR-186-5p |
| DBT | hsa-miR-4282 |
| DBT | hsa-miR-548c-3p |
| DBT | hsa-miR-2381 |
| DBT | hsa-miR-6929-5p |
| DBT | hsa-miR-551-5p |
| DBT | hsa-miR-6842-5p |
| DBT | hsa-miR-455-3p |
| DBT | hsa-miR-448-3p |
| DBT | hsa-miR-4536-5p |
| DBT | hsa-miR-4277 |
| DBT | hsa-miR-6731-3p |
| DBT | hsa-miR-6481 |
| DBT | hsa-miR-106a-3p |
| DBT | hsa-miR-7203-5p |
| DBT | hsa-miR-4531 |
| DBT | hsa-miR-3685 |
| DBT | hsa-miR-1271-5p |
| DBT | hsa-miR-194-3p |
| DBT | hsa-miR-3189-3p |
| DBT | hsa-miR-194-5p |
| DBT | hsa-miR-4491 |
| DBT | hsa-miR-4797-3p |
| DBT | hsa-miR-584-5p |
| DBT | hsa-miR-6822-3p |
| DBT | hsa-miR-5696 |
| DBT | hsa-miR-548bd-3p |
| DBT | hsa-miR-3145-3p |
| DBT | hsa-miR-378 |
| DBT | hsa-let-7 |
| DBT | hsa-miR-519e-3p |
| DBT | hsa-miR-2459 |
| DBT | hsa-miR-3180-5p |
| DBT | hsa-miR-200a-5p |
| DBT | hsa-miR-8054 |
| DBT | hsa-miR-6851-5p |
| DBT | hsa-miR-1721 |
| DBT | hsa-miR-570-5p |
| DBT | hsa-miR-5695 |
| DBT | hsa-miR-361-5p |
| DBT | hsa-miR-374-5p |
| DBT | hsa-miR-4673 |
| DBT | hsa-miR-1224 |
| DBT | hsa-miR-5581-5p |
| DBT | hsa-miR-4318 |
| DBT | hsa-miR-939-3p |
| DBT | hsa-miR-142-3p.2 |
| DBT | hsa-miR-2893 |
| DBT | hsa-miR-195-3p |
| DBT | hsa-miR-138-2-3p |
| DBT | hsa-miR-1843 |
| DBT | hsa-miR-2415-3p |
| DBT | hsa-miR-7398s-3p |
| DBT | hsa-miR-663a |
| DBT | hsa-miR-4774-5p |
| DBT | hsa-miR-2325 |
| DBT | hsa-miR-455-5p |
| DBT | hsa-miR-6787-3p |
| DBT | hsa-miR-219-1-3p |
| DBT | hsa-miR-4316 |
| DBT | hsa-miR-3593-3p |
| DBT | hsa-miR-5190 |
| DBT | hsa-miR-548a-5p |
| DBT | hsa-miR-302c-5p |
| DBT | hsa-miR-559 |
| DBT | hsa-miR-7038-3p |
| DBT | hsa-miR-150-3p |
| DBT | hsa-miR-1224-3p |
| DBT | hsa-miR-3602 |
| DBT | hsa-miR-7-5p |
| DBT | hsa-miR-183-5p |
| DBT | hsa-miR-133a-5p |
| DBT | hsa-miR-4757-5p |
| DBT | hsa-miR-8806 |
| DBT | hsa-miR-6810-5p |
| DBT | hsa-miR-490-3p |
| DBT | hsa-miR-182-5p |
| DBT | hsa-miR-7166-3p |
| DBT | hsa-miR-8846 |
| DBT | hsa-miR-7845-5p |
| DBT | hsa-miR-7277-5p |
| DBT | hsa-miR-96-5p |
| DBT | hsa-miR-6844 |
| DBT | hsa-miR-1273h-5p |
| DBT | hsa-miR-8080 |
| DBT | hsa-miR-6513-5p |
| DBT | hsa-miR-3136-5p |
| DBT | hsa-miR-93-5p |
| DBT | hsa-miR-4727-5p |
| DBT | hsa-miR-548k |
| DBT | hsa-miR-6939-5p |
| DBT | hsa-miR-5615-3p |
| DBT | hsa-miR-448 |
| DBT | hsa-miR-489 |
| DBT | hsa-miR-214-5p |
| DBT | hsa-miR-942-5p |
| DBT | hsa-miR-6985-5p |
| DBT | hsa-miR-2284m |
| DBT | hsa-miR-4641 |
| DBT | hsa-miR-3120-5p |
| DBT | hsa-miR-3613-5p |
| DBT | hsa-miR-6069 |
| DBT | hsa-miR-152 |
| DBT | hsa-miR-3192 |
| DBT | hsa-miR-7214-5p |
| DBT | hsa-miR-516a |
| DBT | hsa-miR-7655-5p |
| DBT | hsa-miR-1804 |
| DBT | hsa-miR-508-5p |
| DBT | hsa-miR-1266-3p |
| DBT | hsa-miR-369-3p |
| DBT | hsa-miR-603 |
| DBT | hsa-miR-5698 |
| DBT | hsa-miR-222-5p |
| DBT | hsa-miR-8876 |
| DBT | hsa-miR-1279 |
| DBT | hsa-miR-7704 |
| DBT | hsa-miR-6954-3p |
| DBT | hsa-miR-4270 |
| DBT | hsa-miR-135 |
| DBT | hsa-miR-200bc-3p |
| DBT | hsa-miR-3144-5p |
| DBT | hsa-miR-577-3p |
| DBT | hsa-miR-7856-5p |
| DBT | hsa-miR-3088-3p |
| DBT | hsa-miR-2330-5p |
| DBT | hsa-miR-4455 |
| DBT | hsa-miR-4293 |
| DBT | hsa-miR-219a-1-3p |
| DBT | hsa-miR-3651 |
| DBT | hsa-miR-577 |
| DBT | hsa-miR-4446-5p |
| DBT | hsa-miR-1915-3p |
| DBT | hsa-miR-4747-5p |
| DBT | hsa-miR-518c-5p |
| DBT | hsa-miR-4267 |
| DBT | hsa-miR-33-5p |
| DBT | hsa-miR-7206-3p |
| DBT | hsa-miR-7166-5p |
| DBT | hsa-miR-548aw |
| DBT | hsa-miR-4468 |
| DBT | hsa-miR-1273g-5p |
| DBT | hsa-miR-4709-3p |
| DBT | hsa-miR-4474-3p |
| DBT | hsa-miR-1202 |
| DBT | hsa-miR-6079 |
| DBT | hsa-miR-3941 |
| DBT | hsa-miR-513b-3p |
| DBT | hsa-miR-1250-3p |
| DBT | hsa-miR-3913-5p |
| DBT | hsa-miR-6821-3p |
| DBT | hsa-miR-10-5p |
| DBT | hsa-miR-1264 |
| DBT | hsa-miR-580-3p |
| DBT | hsa-miR-619-5p |
| DBT | hsa-miR-2285 |
| DBT | hsa-miR-7009-3p |
| DBT | hsa-miR-1839-3p |
| DBT | hsa-miR-219-2-3p |
| DBT | hsa-miR-4802-3p |
| DBT | hsa-miR-1185-5p |
| DBT | hsa-miR-4460 |
| DBT | hsa-miR-2468 |
| DBT | hsa-miR-629-3p |
| DBT | hsa-miR-2861 |
| DBT | hsa-miR-516 |
| DBT | hsa-miR-7388b-5p |
| DBT | hsa-miR-140-3p.1 |
| DBT | hsa-miR-3126-5p |
| DBT | hsa-miR-106 |
| DBT | hsa-miR-3662 |
| DBT | hsa-miR-26-2-3p |
| DBT | hsa-miR-1243-3p |
| DBT | hsa-miR-203b-5p |
| DBT | hsa-miR-2347 |
| DBT | hsa-miR-520g-3p |
| DBT | hsa-miR-3103-3p |
| DBT | hsa-miR-6086 |
| DBT | hsa-miR-30a-3p |
| DBT | hsa-miR-550a-5p |
| DBT | hsa-miR-7207-3p |
| DBT | hsa-miR-124 |
| DBT | hsa-miR-376-3p |
| DBT | hsa-miR-7111-5p |
| DBT | hsa-miR-8089 |
| DBT | hsa-miR-1285-3p |
| DBT | hsa-miR-677-5p |
| DBT | hsa-miR-7152-5p |
| DBT | hsa-miR-153-5p |
| DBT | hsa-miR-674-3p |
| DBT | hsa-miR-6881-5p |
| DBT | hsa-miR-200-3p |
| DBT | hsa-miR-7060-3p |
| DBT | hsa-miR-378a-5p |
| DBT | hsa-miR-6720-3p |
| DBT | hsa-miR-552-3p |
| DBT | hsa-miR-5589-3p |
| DBT | hsa-miR-6869-5p |
| DBT | hsa-miR-3156-3p |
| DBT | hsa-miR-3116 |
| DBT | hsa-miR-3185 |
| DBT | hsa-miR-1273h-3p |
| DBT | hsa-miR-2310 |
| DBT | hsa-miR-3200-5p |
| DBT | hsa-miR-7201-5p |
| DBT | hsa-miR-7680-5p |
| DBT | hsa-miR-371a-5p |
| DBT | hsa-miR-493-3p |
| DBT | hsa-miR-378c |
| DBT | hsa-miR-1263 |
| DBT | hsa-miR-6882-5p |
| DBT | hsa-miR-365a-5p |
| DBT | hsa-miR-129b-5p |
| DBT | hsa-miR-4502 |
| DBT | hsa-miR-6747-5p |
| DBT | hsa-miR-4763-3p |
| DBT | hsa-miR-2439-3p |
| DBT | hsa-miR-3152-5p |
| DBT | hsa-miR-1304-3p |
| DBT | hsa-miR-183-3p |
| DBT | hsa-miR-219a-2-3p |
| DBT | hsa-miR-889-3p |
| DBT | hsa-miR-669 |
| DBT | hsa-miR-4251 |
| DBT | hsa-miR-2449 |
| DBT | hsa-miR-548n |
| DBT | hsa-miR-7175-5p |
| DBT | hsa-miR-25-3p |
| DBT | hsa-miR-4417 |
| DBT | hsa-miR-3161 |
| DBT | hsa-miR-24-3p |
| DBT | hsa-miR-548at-5p |
| DBT | hsa-miR-589-3p |
| DBT | hsa-miR-499b-5p |
| DLAT | hsa-miR-5615-5p |
| DLAT | hsa-miR-4489 |
| DLAT | hsa-miR-202-3p |
| DLAT | hsa-miR-6700-5p |
| DLAT | hsa-miR-7659-3p |
| DLAT | hsa-miR-3924 |
| DLAT | hsa-miR-3154 |
| DLAT | hsa-miR-194-5p |
| DLAT | hsa-miR-124-5p |
| DLAT | hsa-miR-6943-3p |
| DLAT | hsa-miR-6791-3p |
| DLAT | hsa-miR-654-3p |
| DLAT | hsa-miR-556-3p |
| DLAT | hsa-miR-2136 |
| DLAT | hsa-miR-7348-5p |
| DLAT | hsa-miR-2323 |
| DLAT | hsa-miR-181-3p |
| DLAT | hsa-miR-627-3p |
| DLAT | hsa-miR-628-5p |
| DLAT | hsa-miR-7039-3p |
| DLAT | hsa-miR-581 |
| DLAT | hsa-miR-3075 |
| DLAT | hsa-miR-409 |
| DLAT | hsa-miR-4456 |
| DLAT | hsa-miR-463-3p |
| DLAT | hsa-miR-1306-3p |
| DLAT | hsa-miR-7386m-3p |
| DLAT | hsa-miR-4753-5p |
| DLAT | hsa-miR-3689d |
| DLAT | hsa-miR-6555-5p |
| DLAT | hsa-miR-487-3p |
| DLAT | hsa-miR-6418-5p |
| DLAT | hsa-miR-5697-3p |
| DLAT | hsa-miR-4799-5p |
| DLAT | hsa-miR-7647-3p |
| DLAT | hsa-miR-383 |
| DLAT | hsa-miR-144-3p |
| DLAT | hsa-miR-214-3p |
| DLAT | hsa-miR-642 |
| DLAT | hsa-miR-2352 |
| DLAT | hsa-miR-376ab |
| DLAT | hsa-miR-300 |
| DLAT | hsa-miR-522-3p |
| DLAT | hsa-miR-374-5p |
| DLAT | hsa-miR-466g |
| DLAT | hsa-miR-692 |
| DLAT | hsa-miR-125b-1-3p |
| DLAT | hsa-miR-513c-5p |
| DLAT | hsa-miR-219-2-3p |
| DLAT | hsa-miR-6549-3p |
| DLAT | hsa-miR-466b-2-3p |
| DLAT | hsa-miR-183-3p |
| DLAT | hsa-miR-547-5p |
| DLAT | hsa-miR-1737 |
| DLAT | hsa-miR-342-5p |
| DLAT | hsa-miR-3596d |
| DLAT | hsa-miR-1226-3p |
| DLAT | hsa-miR-7374b-3p |
| DLAT | hsa-miR-888-5p |
| DLAT | hsa-miR-3553 |
| DLAT | hsa-miR-548bb-5p |
| DLAT | hsa-miR-4266 |
| DLAT | hsa-miR-7384-3p |
| DLAT | hsa-miR-24-3p |
| DLAT | hsa-miR-4455 |
| DLAT | hsa-miR-8117 |
| DLAT | hsa-miR-4278 |
| DLAT | hsa-miR-4642 |
| DLAT | hsa-miR-365a-3p |
| DLAT | hsa-miR-1285-5p |
| DLAT | hsa-miR-4760-3p |
| DLAT | hsa-miR-4714-3p |
| DLAT | hsa-miR-92b-5p |
| DLAT | hsa-miR-2285b |
| DLAT | hsa-miR-6792-5p |
| DLAT | hsa-miR-7217-3p |
| DLAT | hsa-miR-3198 |
| DLAT | hsa-miR-548c-5p |
| DLAT | hsa-miR-2314 |
| DLAT | hsa-let-7j-3p |
| DLAT | hsa-miR-7048-3p |
| DLAT | hsa-miR-511-5p |
| DLAT | hsa-miR-207 |
| DLAT | hsa-miR-4433b-3p |
| DLAT | hsa-miR-6753-5p |
| DLAT | hsa-miR-7678-3p |
| DLAT | hsa-miR-6964-5p |
| DLAT | hsa-miR-135a-1-3p |
| DLAT | hsa-miR-500 |
| DLAT | hsa-miR-6516-3p |
| DLAT | hsa-miR-1781-3p |
| DLAT | hsa-miR-1982-3p |
| DLAT | hsa-miR-8869 |
| DLAT | hsa-miR-1729-5p |
| DLAT | hsa-miR-24-3p |
| DLAT | hsa-miR-3942-3p |
| DLAT | hsa-miR-499b-3p |
| DLAT | hsa-miR-3182 |
| DLAT | hsa-miR-7047-5p |
| DLAT | hsa-miR-3653-3p |
| DLAT | hsa-miR-7070-5p |
| DLAT | hsa-miR-3616-5p |
| DLAT | hsa-miR-1911-3p |
| DLAT | hsa-miR-624-3p |
| DLAT | hsa-miR-3177-5p |
| DLAT | hsa-miR-4667-3p |
| DLAT | hsa-miR-1-3p |
| DLAT | hsa-miR-6926-3p |
| DLAT | hsa-miR-465a-5p |
| DLAT | hsa-miR-4701-3p |
| DLAT | hsa-miR-3126-3p |
| DLAT | hsa-miR-499b-3p |
| DLAT | hsa-miR-10a-3p |
| DLAT | hsa-miR-6923-5p |
| DLAT | hsa-miR-1206 |
| DLAT | hsa-miR-4309 |
| DLAT | hsa-miR-6704-5p |
| DLAT | hsa-miR-3084bc-5p |
| DLAT | hsa-miR-2438 |
| DLAT | hsa-miR-631 |
| DLAT | hsa-miR-6982-3p |
| DLAT | hsa-miR-2284z |
| DLAT | hsa-miR-676-3p |
| DLAT | hsa-miR-96-5p |
| DLAT | hsa-miR-2053 |
| DLAT | hsa-miR-1619 |
| DLAT | hsa-miR-146b-3p |
| DLAT | hsa-miR-7973 |
| DLAT | hsa-miR-5011-5p |
| DLAT | hsa-miR-495-5p |
| DLAT | hsa-miR-687 |
| DLAT | hsa-miR-4277 |
| DLAT | hsa-miR-544-3p |
| DLAT | hsa-miR-1912-3p |
| DLAT | hsa-miR-129-2-3p |
| DLAT | hsa-miR-1622 |
| DLAT | hsa-miR-4503 |
| DLAT | hsa-miR-8855 |
| DLAT | hsa-miR-1910-3p |
| DLAT | hsa-miR-7074-3p |
| DLAT | hsa-miR-505-3p |
| DLAT | hsa-miR-6640-5p |
| DLAT | hsa-miR-1804 |
| DLAT | hsa-miR-6124 |
| DLAT | hsa-miR-3102-5p |
| DLAT | hsa-miR-7194-3p |
| DLAT | hsa-miR-6085 |
| DLAT | hsa-miR-133 |
| DLAT | hsa-miR-6813-5p |
| DLAT | hsa-miR-4719 |
| DLAT | hsa-miR-7371e-3p |
| DLAT | hsa-miR-327 |
| DLAT | hsa-miR-6337 |
| DLAT | hsa-miR-1933-5p |
| DLAT | hsa-miR-6845-3p |
| DLAT | hsa-miR-6965-5p |
| DLAT | hsa-miR-498 |
| DLAT | hsa-miR-342-3p |
| DLAT | hsa-miR-6830-3p |
| DLAT | hsa-miR-2375 |
| DLAT | hsa-miR-8867 |
| DLAT | hsa-miR-3911 |
| DLAT | hsa-miR-199-3p |
| DLAT | hsa-miR-297bc-3p |
| DLAT | hsa-miR-6829-3p |
| DLAT | hsa-miR-1596-3p |
| DLAT | hsa-miR-27-3p |
| DLAT | hsa-miR-7213-3p |
| DLAT | hsa-miR-2432 |
| DLAT | hsa-miR-3569-3p |
| DLAT | hsa-miR-3192-3p |
| DLAT | hsa-miR-6997-5p |
| DLAT | hsa-miR-5697 |
| DLAT | hsa-miR-146-5p |
| DLAT | hsa-miR-6972-5p |
| DLAT | hsa-miR-4694-3p |
| DLAT | hsa-miR-4638-3p |
| DLAT | hsa-miR-454-3p |
| DLAT | hsa-miR-6953-3p |
| DLAT | hsa-miR-5696 |
| DLAT | hsa-miR-1261 |
| DLAT | hsa-miR-597-5p |
| DLAT | hsa-miR-6344 |
| DLAT | hsa-miR-6809-5p |
| DLAT | hsa-miR-770-5p |
| DLAT | hsa-miR-150-5p |
| DLAT | hsa-miR-212-3p |
| DLAT | hsa-miR-548g-3p |
| DLAT | hsa-miR-8106 |
| DLAT | hsa-miR-8895 |
| DLAT | hsa-miR-888-5p |
| DLAT | hsa-miR-7398l-3p |
| DLAT | hsa-miR-2284aa |
| DLAT | hsa-miR-8831 |
| DLAT | hsa-miR-6919-5p |
| DLAT | hsa-miR-133-3p |
| DLAT | hsa-miR-126-5p |
| DLAT | hsa-miR-7397-3p |
| DLAT | hsa-miR-4423 |
| DLAT | hsa-miR-7370-5p |
| DLAT | hsa-miR-6083 |
| DLAT | hsa-miR-3660 |
| DLAT | hsa-miR-7014-5p |
| DLAT | hsa-miR-7193-3p |
| DLAT | hsa-miR-375 |
| DLAT | hsa-miR-2285af |
| DLAT | hsa-miR-7175-3p |
| DLAT | hsa-miR-452-3p |
| DLAT | hsa-miR-8838 |
| DLAT | hsa-miR-7260-5p |
| DLAT | hsa-miR-6707-5p |
| DLAT | hsa-miR-32-3p |
| DLAT | hsa-miR-7314-5p |
| DLAT | hsa-miR-1277-5p |
| DLAT | hsa-miR-3922-5p |
| DLAT | hsa-miR-1297 |
| DLAT | hsa-miR-1262 |
| DLAT | hsa-miR-7385f-3p |
| DLAT | hsa-miR-2325 |
| DLAT | hsa-miR-6633-5p |
| DLAT | hsa-miR-3686 |
| DLAT | hsa-miR-497-3p |
| DLAT | hsa-miR-883-3p |
| DLAT | hsa-miR-511-3p |
| DLAT | hsa-miR-132-3p |
| DLAT | hsa-miR-329-5p |
| DLAT | hsa-miR-7050-5p |
| DLAT | hsa-miR-556-5p |
| DLAT | hsa-miR-3569-5p |
| DLAT | hsa-let-7-3p |
| DLAT | hsa-miR-7262a-5p |
| DLAT | hsa-miR-129-5p |
| DLAT | hsa-miR-548ad-5p |
| DLAT | hsa-miR-347 |
| DLAT | hsa-miR-7371g-5p |
| DLAT | hsa-miR-3125 |
| DLAT | hsa-miR-8070 |
| DLAT | hsa-miR-378-3p |
| DLAT | hsa-miR-3070-5p |
| DLAT | hsa-miR-130a-5p |
| DLAT | hsa-miR-153-5p |
| DLAT | hsa-miR-29a-5p |
| DLAT | hsa-miR-33a-3p |
| DLAT | hsa-miR-182-5p |
| DLAT | hsa-miR-298-5p |
| DLAT | hsa-miR-6733-3p |
| DLAT | hsa-miR-466l-3p |
| DLAT | hsa-miR-668-3p |
| DLAT | hsa-miR-4302 |
| DLAT | hsa-miR-548c-3p |
| DLAT | hsa-miR-1299 |
| DLAT | hsa-miR-6119-3p |
| DLAT | hsa-miR-3584-5p |
| DLAT | hsa-miR-3941 |
| DLAT | hsa-miR-548ae-5p |
| DLAT | hsa-miR-448-5p |
| DLAT | hsa-miR-644a |
| DLAT | hsa-miR-5003-5p |
| DLAT | hsa-miR-3614-5p |
| DLAT | hsa-miR-467f |
| DLAT | hsa-miR-27a-5p |
| DLAT | hsa-miR-1747-3p |
| DLAT | hsa-miR-34b-3p |
| DLAT | hsa-miR-1768 |
| DLAT | hsa-miR-1661 |
| DLAT | hsa-miR-7275-5p |
| DLAT | hsa-miR-96-3p |
| DLAT | hsa-miR-6834-3p |
| DLAT | hsa-miR-877-3p |
| DLAT | hsa-miR-891b |
| DLAT | hsa-miR-4797-5p |
| DLAT | hsa-miR-1712-3p |
| DLAT | hsa-miR-205-5p |
| DLAT | hsa-miR-2284-5p |
| DLAT | hsa-miR-7353-3p |
| DLAT | hsa-miR-7060-5p |
| DLAT | hsa-miR-7445-5p |
| DLAT | hsa-miR-7856-5p |
| DLAT | hsa-miR-548i |
| DLAT | hsa-miR-3568 |
| DLAT | hsa-miR-466f-3p |
| DLAT | hsa-miR-1651-3p |
| DLAT | hsa-miR-378c |
| DLAT | hsa-miR-6741-5p |
| DLAT | hsa-miR-200-3p |
| DLAT | hsa-miR-1207-3p |
| DLAT | hsa-miR-4778-5p |
| DLAT | hsa-miR-1434-5p |
| DLAT | hsa-miR-376c-5p |
| DLAT | hsa-miR-101c |
| DLAT | hsa-miR-8805 |
| DLAT | hsa-miR-513b-5p |
| DLAT | hsa-miR-6795-5p |
| DLAT | hsa-miR-1968-3p |
| DLAT | hsa-miR-188-5p |
| DLAT | hsa-miR-758-5p |
| DLAT | hsa-miR-1284 |
| DLAT | hsa-miR-539-5p |
| DLAT | hsa-miR-3594-5p |
| DLAT | hsa-miR-26-3p |
| DLAT | hsa-miR-502-5p |
| DLAT | hsa-miR-6982-5p |
| DLAT | hsa-miR-6902-5p |
| DLAT | hsa-miR-218a-1-3p |
| DLAT | hsa-miR-101-1-5p |
| DLAT | hsa-miR-7386h-5p |
| DLAT | hsa-miR-6543-3p |
| DLAT | hsa-miR-6917-3p |
| DLAT | hsa-miR-6731-5p |
| DLAT | hsa-miR-467g |
| DLAT | hsa-miR-1267 |
| DLAT | hsa-miR-570-3p |
| DLAT | hsa-miR-432-5p |
| DLAT | hsa-miR-599-5p |
| DLAT | hsa-let-7f-2-3p |
| DLAT | hsa-miR-548ba |
| DLAT | hsa-miR-200b-5p |
| DLAT | hsa-miR-548y |
| DLAT | hsa-miR-200ac-5p |
| DLAT | hsa-miR-340-5p |
| DLAT | hsa-miR-8080 |
| DLAT | hsa-miR-1747-5p |
| DLAT | hsa-miR-653-5p |
| DLAT | hsa-miR-1759-3p |
| DLAT | hsa-miR-7007-5p |
| DLAT | hsa-miR-204-5p |
| DLAT | hsa-miR-548av-5p |
| DLAT | hsa-miR-20-3p |
| DLAT | hsa-miR-2284l |
| DLAT | hsa-miR-7651-3p |
| DLAT | hsa-miR-2310 |
| DLAT | hsa-miR-935 |
| DLAT | hsa-miR-3068-5p |
| DLAT | hsa-miR-1207 |
| DLAT | hsa-miR-1-1-5p |
| DLAT | hsa-miR-589-3p |
| DLAT | hsa-miR-3938-5p |
| DLAT | hsa-miR-3572-3p |
| DLAT | hsa-miR-493-3p |
| DLAT | hsa-let-7abf-3p |
| DLAT | hsa-miR-7646-5p |
| DLAT | hsa-miR-7262b-5p |
| DLAT | hsa-miR-1-2-5p |
| DLAT | hsa-miR-3583-5p |
| DLAT | hsa-miR-219-3p |
| DLAT | hsa-miR-148-3p |
| DLAT | hsa-miR-7386g-3p |
| DLAT | hsa-miR-7386k-3p |
| DLAT | hsa-miR-7062-5p |
| DLAT | hsa-miR-3572 |
| DLAT | hsa-miR-193-5p |
| DLAT | hsa-miR-6590-3p |
| DLAT | hsa-miR-582-3p |
| DLAT | hsa-miR-5004-3p |
| DLAT | hsa-miR-17-3p |
| DLAT | hsa-miR-1324 |
| DLAT | hsa-miR-6514-5p |
| DLAT | hsa-miR-4528 |
| DLAT | hsa-miR-7388b-3p |
| DLAT | hsa-miR-7398m-3p |
| DLAT | hsa-miR-548n |
| DLAT | hsa-miR-2448-3p |
| DLAT | hsa-miR-6315 |
| DLAT | hsa-miR-489-3p |
| DLAT | hsa-miR-3121-3p |
| DLAT | hsa-miR-20-5p |
| DLAT | hsa-miR-5195-5p |
| DLAT | hsa-miR-1956 |
| DLAT | hsa-miR-3682-3p |
| DLAT | hsa-miR-7469-5p |
| DLAT | hsa-miR-548au-3p |
| DLAT | hsa-miR-1457 |
| DLAT | hsa-miR-373-3p |
| DLAT | hsa-miR-1197-5p |
| DLAT | hsa-miR-582-5p |
| DLAT | hsa-miR-4762-3p |
| DLAT | hsa-miR-3689f |
| DLAT | hsa-miR-6569-5p |
| DLAT | hsa-miR-2284g |
| DLAT | hsa-miR-2418 |
| DLAT | hsa-miR-513a-3p |
| DLAT | hsa-miR-4428 |
| DLAT | hsa-miR-1584-3p |
| DLAT | hsa-miR-1964-5p |
| DLAT | hsa-miR-329-3p |
| DLAT | hsa-miR-562 |
| DLAT | hsa-miR-3571 |
| DLAT | hsa-miR-365a-3p |
| DLAT | hsa-miR-514b-5p |
| DLAT | hsa-miR-15a-3p |
| DLAT | hsa-miR-7332-5p |
| DLAT | hsa-miR-8884 |
| DLAT | hsa-miR-362-3p |
| DLAT | hsa-miR-3607-5p |
| DLAT | hsa-miR-3103-5p |
| DLAT | hsa-miR-1264-3p |
| DLAT | hsa-miR-380 |
| DLAT | hsa-miR-361-5p |
| DLAT | hsa-miR-5694 |
| DLAT | hsa-miR-7305-5p |
| DLAT | hsa-miR-4282 |
| DLAT | hsa-miR-8874 |
| DLAT | hsa-miR-4263 |
| DLAT | hsa-miR-605-3p |
| DLAT | hsa-miR-4446-5p |
| DLAT | hsa-miR-329b |
| DLAT | hsa-miR-6780b-3p |
| DLAT | hsa-miR-3473be |
| DLAT | hsa-miR-92a-1-5p |
| DLAT | hsa-miR-7355-3p |
| DLAT | hsa-miR-323b-3p |
| DLAT | hsa-miR-7398u-3p |
| DLAT | hsa-miR-552-5p |
| DLAT | hsa-miR-6833-5p |
| DLAT | hsa-miR-6750-3p |
| DLAT | hsa-miR-668 |
| DLAT | hsa-miR-6612-5p |
| DLAT | hsa-miR-1564-5p |
| DLAT | hsa-miR-1711 |
| DLAT | hsa-miR-875-3p |
| DLAT | hsa-miR-380-5p |
| DLAT | hsa-miR-2340 |
| DLAT | hsa-miR-7398r-3p |
| DLAT | hsa-miR-465d-3p |
| DLAT | hsa-miR-7029-5p |
| DLAT | hsa-miR-7202-3p |
| DLAT | hsa-miR-6914-3p |
| DLAT | hsa-miR-7291-5p |
| DLAT | hsa-miR-7395-3p |
| DLAT | hsa-miR-7318-5p |
| DLAT | hsa-miR-4281 |
| DLAT | hsa-miR-429-5p |
| DLAT | hsa-miR-4716-3p |
| DLAT | hsa-miR-329 |
| DLAT | hsa-miR-106-5p |
| DLAT | hsa-miR-879-5p |
| DLAT | hsa-miR-6822-5p |
| DLAT | hsa-miR-199-5p |
| DLAT | hsa-miR-143-3p |
| DLAT | hsa-miR-30e |
| DLAT | hsa-miR-548w |
| DLAT | hsa-miR-7077-3p |
| DLAT | hsa-miR-6393 |
| DLAT | hsa-miR-7006-5p |
| DLAT | hsa-miR-548b-3p |
| DLAT | hsa-miR-548ai |
| DLAT | hsa-miR-6943-5p |
| DLAT | hsa-miR-676-5p |
| DLAT | hsa-miR-371 |
| DLAT | hsa-miR-6538 |
| DLAT | hsa-miR-2284y |
| DLAT | hsa-miR-130b-5p |
| DLAT | hsa-miR-7051-5p |
| DLAT | hsa-miR-6357 |
| DLAT | hsa-miR-669bf-3p |
| DLAT | hsa-miR-6513-5p |
| DLAT | hsa-miR-335-3p |
| DLAT | hsa-miR-4446-3p |
| DLAT | hsa-miR-3148 |
| DLAT | hsa-miR-7671-3p |
| DLAT | hsa-miR-4699-5p |
| DLAT | hsa-miR-6340 |
| DLAT | hsa-miR-371b-5p |
| DLAT | hsa-miR-96-5p |
| DLAT | hsa-miR-376abd |
| DLAT | hsa-miR-5099 |
| DLAT | hsa-miR-708-5p |
| DLAT | hsa-miR-6986-3p |
| DLAT | hsa-miR-6345 |
| DLAT | hsa-miR-216c-3p |
| DLAT | hsa-miR-465-5p |
| DLAT | hsa-miR-3920 |
| DLAT | hsa-miR-3586-3p |
| DLAT | hsa-miR-3661 |
| DLAT | hsa-miR-346 |
| DLAT | hsa-miR-4330 |
| DLAT | hsa-miR-4635 |
| DLAT | hsa-miR-7024-3p |
| DLAT | hsa-miR-7650-5p |
| DLAT | hsa-miR-5132-3p |
| DLAT | hsa-miR-3577 |
| DLAT | hsa-miR-761 |
| DLAT | hsa-miR-3060-5p |
| DLAT | hsa-miR-4729 |
| DLAT | hsa-miR-1769-5p |
| DLAT | hsa-miR-124 |
| DLAT | hsa-miR-362-5p |
| DLAT | hsa-miR-2303 |
| DLAT | hsa-miR-597 |
| DLAT | hsa-miR-7344-3p |
| DLAT | hsa-miR-3689b-5p |
| DLAT | hsa-miR-4797-3p |
| DLAT | hsa-miR-4299 |
| DLAT | hsa-miR-4443 |
| DLAT | hsa-miR-6322 |
| DLAT | hsa-miR-181b-1-3p |
| DLAT | hsa-miR-6544-5p |
| DLAT | hsa-miR-7398a-3p |
| DLAT | hsa-miR-223-5p |
| DLAT | hsa-miR-8904a |
| DLAT | hsa-miR-465-3p |
| DLAT | hsa-miR-302a-5p |
| DLAT | hsa-miR-26-5p |
| DLAT | hsa-miR-1695 |
| DLAT | hsa-miR-4490 |
| DLAT | hsa-miR-7357-5p |
| DLAT | hsa-miR-7437-5p |
| DLAT | hsa-miR-570-5p |
| DLAT | hsa-miR-6918-3p |
| DLAT | hsa-miR-548aq-5p |
| DLAT | hsa-miR-2285aa |
| DLAT | hsa-miR-1597-5p |
| DLAT | hsa-let-7a-2-3p |
| DLAT | hsa-miR-7298-3p |
| DLAT | hsa-miR-465c-5p |
| DLAT | hsa-miR-7386g-5p |
| DLAT | hsa-miR-1197 |
| DLAT | hsa-miR-7178-3p |
| DLAT | hsa-miR-6798-5p |
| DLAT | hsa-miR-6417 |
| DLAT | hsa-miR-194-5p |
| DLAT | hsa-miR-2284ac |
| DLAT | hsa-miR-6558-3p |
| DLAT | hsa-miR-7197-3p |
| DLAT | hsa-miR-770-3p |
| DLAT | hsa-miR-1626-5p |
| DLAT | hsa-miR-1954 |
| DLAT | hsa-miR-320-5p |
| DLAT | hsa-miR-6515-5p |
| DLAT | hsa-miR-8058 |
| DLAT | hsa-miR-7657-3p |
| DLAT | hsa-miR-6971-3p |
| DLAT | hsa-miR-5580-3p |
| DLAT | hsa-miR-656-3p |
| DLAT | hsa-miR-488-5p |
| DLAT | hsa-miR-4768-5p |
| DLAT | hsa-miR-345-5p |
| DLAT | hsa-miR-190a-5p |
| DLAT | hsa-miR-7118-5p |
| DLAT | hsa-miR-494-3p |
| DLAT | hsa-miR-18-3p |
| DLAT | hsa-miR-2284u |
| DLAT | hsa-miR-693-3p |
| DLAT | hsa-miR-6736-5p |
| DLAT | hsa-miR-488-3p |
| DLAT | hsa-miR-6641-5p |
| DLAT | hsa-miR-100-3p |
| DLAT | hsa-miR-7374c-3p |
| DLAT | hsa-miR-6983-3p |
| DLAT | hsa-miR-383-5p.1 |
| DLAT | hsa-miR-7324-5p |
| DLAT | hsa-miR-3097-5p |
| DLAT | hsa-miR-6868-3p |
| DLAT | hsa-miR-3916 |
| DLAT | hsa-miR-7244-5p |
| DLAT | hsa-miR-4422 |
| DLAT | hsa-miR-6728-5p |
| DLAT | hsa-miR-664 |
| DLAT | hsa-miR-872-3p |
| DLAT | hsa-miR-616-5p |
| DLAT | hsa-miR-23-3p |
| DLAT | hsa-miR-7215-5p |
| DLAT | hsa-miR-200a-3p |
| DLAT | hsa-miR-9769-5p |
| DLAT | hsa-miR-942-5p |
| DLAT | hsa-miR-2446 |
| DLAT | hsa-miR-5571-5p |
| DLAT | hsa-miR-195-3p |
| DLAT | hsa-miR-203-3p |
| DLAT | hsa-miR-6512-5p |
| DLAT | hsa-miR-374 |
| DLAT | hsa-miR-26 |
| DLAT | hsa-miR-3610 |
| DLAT | hsa-miR-7196-5p |
| DLAT | hsa-miR-7191-3p |
| DLAT | hsa-miR-128 |
| DLAT | hsa-miR-101-3p |
| DLAT | hsa-miR-6927-5p |
| DLAT | hsa-miR-181b-2-3p |
| DLAT | hsa-miR-553 |
| DLAT | hsa-miR-325 |
| DLAT | hsa-miR-1760 |
| DLAT | hsa-miR-1680-5p |
| DLAT | hsa-miR-7055-5p |
| DLAT | hsa-miR-655-3p |
| DLAT | hsa-miR-3589 |
| DLAT | hsa-miR-507b |
| DLAT | hsa-miR-132-3p |
| DLAT | hsa-miR-136-5p |
| DLAT | hsa-miR-6069 |
| DLAT | hsa-miR-545-5p |
| DLAT | hsa-miR-7195-5p |
| DLAT | hsa-miR-763 |
| DLAT | hsa-miR-520g-5p |
| DLAT | hsa-miR-8061 |
| DLAT | hsa-miR-335-5p |
| DLAT | hsa-miR-2489 |
| DLAT | hsa-miR-505-3p.2 |
| DLAT | hsa-miR-6874-3p |
| DLAT | hsa-miR-1225-5p |
| DLAT | hsa-miR-2052 |
| DLAT | hsa-miR-5695 |
| DLAT | hsa-miR-7094-1-5p |
| DLAT | hsa-miR-7398b-3p |
| DLAT | hsa-miR-3667-3p |
| DLAT | hsa-miR-4672 |
| DLAT | hsa-miR-129-3p |
| DLAT | hsa-miR-1626-3p |
| DLAT | hsa-miR-6805-5p |
| DLAT | hsa-miR-1652 |
| DLAT | hsa-miR-30 |
| DLAT | hsa-miR-129-5p |
| DLAT | hsa-miR-587 |
| DLAT | hsa-miR-7303-5p |
| DLAT | hsa-miR-628-3p |
| DLAT | hsa-miR-217 |
| DLAT | hsa-miR-380-3p |
| DLAT | hsa-miR-495-3p |
| DLAT | hsa-miR-6715-5p |
| DLAT | hsa-miR-5571-3p |
| DLAT | hsa-miR-376a-3p |
| DLAT | hsa-miR-7211-3p |
| DLAT | hsa-miR-7207-3p |
| DLAT | hsa-miR-1543 |
| DLAT | hsa-miR-7467-3p |
| DLAT | hsa-miR-7398s-3p |
| DLAT | hsa-miR-6632-5p |
| DLAT | hsa-miR-548as-5p |
| DLAT | hsa-miR-4717-5p |
| DLAT | hsa-miR-3606-3p |
| DLAT | hsa-miR-217-3p |
| DLAT | hsa-miR-1775-5p |
| DLAT | hsa-miR-296-3p |
| DLAT | hsa-miR-26a-3p |
| DLAT | hsa-miR-34 |
| DLAT | hsa-miR-95-5p |
| DLAT | hsa-miR-548ar-5p |
| DLAT | hsa-miR-10b-3p |
| DLAT | hsa-miR-3688-3p |
| DLAT | hsa-miR-1287-5p |
| DLAT | hsa-miR-490-5p |
| DLAT | hsa-miR-1199-5p |
| DLAT | hsa-miR-7153-3p |
| DLAT | hsa-miR-448 |
| DLAT | hsa-miR-3596c |
| DLAT | hsa-miR-7480-5p |
| DLAT | hsa-miR-3167-3p |
| DLAT | hsa-miR-7478-3p |
| DLAT | hsa-miR-3600 |
| DLAT | hsa-miR-2313-3p |
| DLAT | hsa-miR-361-3p |
| DLAT | hsa-miR-4423-3p |
| DLAT | hsa-miR-6128 |
| DLAT | hsa-miR-520-5p |
| DLAT | hsa-miR-4521 |
| DLAT | hsa-miR-1281 |
| DLAT | hsa-miR-1243-3p |
| DLAT | hsa-miR-625-5p |
| DLAT | hsa-miR-376e |
| DLAT | hsa-miR-519e-5p |
| DLAT | hsa-miR-22-5p |
| DLAT | hsa-miR-6902-3p |
| DLAT | hsa-miR-1555-5p |
| DLAT | hsa-miR-122 |
| DLAT | hsa-miR-3133 |
| DLAT | hsa-miR-2482 |
| DLAT | hsa-miR-6584-5p |
| DLAT | hsa-miR-5618-3p |
| DLAT | hsa-miR-1592 |
| DLAT | hsa-miR-548l |
| DLAT | hsa-miR-707 |
| DLAT | hsa-miR-8826 |
| DLAT | hsa-miR-483-3p.1 |
| DLAT | hsa-miR-6984-3p |
| DLAT | hsa-miR-8065 |
| DLAT | hsa-miR-7301-5p |
| DLAT | hsa-miR-6649-3p |
| DLAT | hsa-miR-98-3p |
| DLAT | hsa-miR-9-3-3p |
| DLAT | hsa-miR-145-5p |
| DLAT | hsa-miR-3539 |
| DLAT | hsa-miR-3120-3p |
| DLAT | hsa-miR-3065-5p |
| DLAT | hsa-miR-138-2-3p |
| DLAT | hsa-miR-203a-5p |
| DLAT | hsa-miR-656 |
| DLAT | hsa-miR-133a-3p.1 |
| DLAT | hsa-miR-7386e-5p |
| DLAT | hsa-miR-8908-3p |
| DLAT | hsa-miR-378 |
| DLAT | hsa-miR-511 |
| DLAT | hsa-miR-466n-3p |
| DLAT | hsa-miR-4264 |
| DLAT | hsa-miR-466bco-5p |
| DLAT | hsa-miR-128-3p |
| DLAT | hsa-miR-3551-3p |
| DLAT | hsa-miR-6808-5p |
| DLAT | hsa-miR-194-2-3p |
| DLAT | hsa-miR-4445-5p |
| DLAT | hsa-miR-8900 |
| DLAT | hsa-miR-6859-5p |
| DLAT | hsa-miR-767 |
| DLAT | hsa-miR-495-3p |
| DLAT | hsa-miR-7168-5p |
| DLAT | hsa-miR-130-3p |
| DLAT | hsa-miR-4452 |
| DLAT | hsa-miR-8069 |
| DLAT | hsa-miR-6511a-5p |
| DLAT | hsa-miR-883-5p |
| DLAT | hsa-miR-7207-5p |
| DLAT | hsa-miR-669hk-3p |
| DLAT | hsa-miR-576-5p |
| DLAT | hsa-miR-3066-5p |
| DLAT | hsa-miR-18-5p |
| DLAT | hsa-miR-147a |
| DLAT | hsa-miR-1224-3p |
| DLAT | hsa-miR-8100 |
| DLAT | hsa-miR-375-3p |
| DLAT | hsa-miR-30e-3p |
| DLAT | hsa-miR-182-5p |
| DLAT | hsa-miR-6873-3p |
| DLAT | hsa-miR-702-5p |
| DLAT | hsa-miR-202-5p |
| DLAT | hsa-miR-5009-5p |
| DLAT | hsa-miR-200a-3p |
| DLAT | hsa-miR-4499 |
| DLAT | hsa-miR-548t-5p |
| DLAT | hsa-miR-7013-3p |
| DLAT | hsa-miR-3475-3p |
| DLAT | hsa-miR-7705 |
| DLAT | hsa-miR-302-5p |
| DLAT | hsa-miR-670-3p |
| DLAT | hsa-miR-6899-5p |
| DLAT | hsa-miR-7851-3p |
| DLAT | hsa-miR-23a-5p |
| DLAT | hsa-miR-520g-3p |
| DLAT | hsa-miR-4802-5p |
| DLAT | hsa-miR-6828-5p |
| DLAT | hsa-miR-7470-3p |
| DLAT | hsa-miR-322-3p |
| DLAT | hsa-miR-331-3p |
| DLAT | hsa-miR-30a-3p |
| DLAT | hsa-miR-6930-5p |
| DLAT | hsa-miR-205-3p |
| DLAT | hsa-miR-1298-3p |
| DLAT | hsa-miR-1546-5p |
| DLAT | hsa-miR-7-3p |
| DLAT | hsa-miR-5103 |
| DLAT | hsa-miR-140-3p |
| DLAT | hsa-miR-4708-3p |
| DLAT | hsa-miR-466 |
| DLAT | hsa-miR-548ab |
| DLAT | hsa-miR-6732-5p |
| DLAT | hsa-miR-365b-3p |
| DLAT | hsa-miR-6999-5p |
| DLAT | hsa-miR-1575 |
| DLAT | hsa-miR-302-3p |
| DLAT | hsa-miR-3689e |
| DLAT | hsa-miR-1179 |
| DLAT | hsa-miR-513c-3p |
| DLAT | hsa-miR-373-5p |
| DLAT | hsa-miR-515-5p |
| DLAT | hsa-miR-6893-5p |
| DLAT | hsa-miR-603 |
| DLAT | hsa-miR-146a-3p |
| DLAT | hsa-miR-6595-5p |
| DLAT | hsa-miR-590-5p |
| DLAT | hsa-miR-9500 |
| DLAT | hsa-miR-1298 |
| DLAT | hsa-miR-3127-5p |
| DLAT | hsa-miR-1949 |
| DLAT | hsa-miR-34a-3p |
| DLAT | hsa-miR-2381 |
| DLAT | hsa-miR-21-5p |
| DLAT | hsa-miR-3543 |
| DLAT | hsa-miR-6823-5p |
| DLAT | hsa-miR-7067-3p |
| DLAT | hsa-miR-548ap-5p |
| DLAT | hsa-miR-7371j-5p |
| DLAT | hsa-miR-377-3p |
| DLAT | hsa-miR-8857 |
| DLAT | hsa-miR-4652-3p |
| DLAT | hsa-miR-421-3p |
| DLAT | hsa-miR-1842 |
| DLAT | hsa-miR-3093-3p |
| DLAT | hsa-miR-548j-5p |
| DLAT | hsa-miR-5187-3p |
| DLAT | hsa-miR-7032-3p |
| DLAT | hsa-miR-5089-5p |
| DLAT | hsa-miR-216b-3p |
| DLAT | hsa-miR-6330 |
| DLAT | hsa-miR-222-5p |
| DLAT | hsa-miR-874-5p |
| DLAT | hsa-miR-30c-3p |
| DLAT | hsa-miR-484 |
| DLAT | hsa-miR-1687-5p |
| DLAT | hsa-miR-669p-3p |
| DLAT | hsa-miR-1289 |
| DLAT | hsa-miR-4509 |
| DLAT | hsa-miR-8118 |
| DLAT | hsa-miR-191-3p |
| DLAT | hsa-miR-590-3p |
| DLAT | hsa-miR-584-3p |
| DLAT | hsa-miR-2298 |
| DLAT | hsa-miR-6912-3p |
| DLAT | hsa-miR-1814 |
| DLAT | hsa-miR-6542-5p |
| DLAT | hsa-miR-5617-5p |
| DLAT | hsa-miR-450b-3p |
| DLAT | hsa-miR-320a |
| DLAT | hsa-miR-702-3p |
| DLAT | hsa-miR-7239-5p |
| DLAT | hsa-miR-6808-3p |
| DLAT | hsa-miR-1468-3p |
| DLAT | hsa-miR-8801 |
| DLAT | hsa-miR-1464 |
| DLAT | hsa-miR-1894-5p |
| DLAT | hsa-miR-539-5p |
| DLAT | hsa-miR-6734-3p |
| DLAT | hsa-miR-183-5p.2 |
| DLAT | hsa-miR-1240 |
| DLAT | hsa-miR-892a |
| DLAT | hsa-miR-7349-3p |
| DLAT | hsa-miR-2367-3p |
| DLAT | hsa-miR-376-3p |
| DLAT | hsa-miR-300-5p |
| DLAT | hsa-miR-892-5p |
| DLAT | hsa-miR-8839 |
| DLAT | hsa-miR-344b-3p |
| DLAT | hsa-miR-664a-3p |
| DLAT | hsa-miR-215 |
| DLAT | hsa-miR-1688 |
| DLAT | hsa-miR-139-5p |
| DLAT | hsa-miR-7266-3p |
| DLAT | hsa-miR-644a |
| DLAT | hsa-miR-497-5p |
| DLAT | hsa-miR-5584-5p |
| DLAT | hsa-miR-190a-3p |
| DLAT | hsa-miR-4760-5p |
| DLAT | hsa-miR-579 |
| DLAT | hsa-miR-95-3p |
| DLAT | hsa-miR-5088-5p |
| DLAT | hsa-miR-7261-3p |
| DLAT | hsa-miR-1563 |
| DLAT | hsa-miR-2909 |
| DLAT | hsa-miR-3127-5p |
| DLAT | hsa-miR-432-5p |
| DLAT | hsa-miR-323 |
| DLAT | hsa-miR-7398-5p |
| DLAT | hsa-miR-7661-5p |
| DLAT | hsa-miR-421 |
| DLAT | hsa-miR-548ag |
| DLAT | hsa-miR-6715a-3p |
| DLAT | hsa-miR-6329 |
| DLAT | hsa-miR-6616-5p |
| DLAT | hsa-miR-483-3p |
| DLAT | hsa-miR-381-3p |
| DLAT | hsa-miR-125a-3p |
| DLAT | hsa-miR-8827 |
| DLAT | hsa-miR-548az-5p |
| DLAT | hsa-miR-7158-3p |
| DLAT | hsa-miR-7374a-3p |
| DLAT | hsa-miR-224-5p |
| DLAT | hsa-miR-192-5p |
| DLAT | hsa-miR-450a-1-3p |
| DLAT | hsa-miR-655 |
| DLAT | hsa-miR-2188-5p |
| DLAT | hsa-miR-539-3p |
| DLAT | hsa-miR-1191b-5p |
| DLAT | hsa-miR-6974-3p |
| DLAT | hsa-miR-548o-5p |
| DLAT | hsa-miR-1458 |
| DLAT | hsa-miR-1781-5p |
| DLAT | hsa-miR-6750-5p |
| DLAT | hsa-miR-6854-5p |
| DLAT | hsa-miR-7191-5p |
| DLAT | hsa-miR-30d-3p |
| DLAT | hsa-miR-579-3p |
| DLAT | hsa-miR-885-5p |
| DLAT | hsa-miR-7046-3p |
| DLAT | hsa-miR-1981-5p |
| DLAT | hsa-miR-1459 |
| DLAT | hsa-miR-1788-5p |
| DLAT | hsa-miR-206-5p |
| DLAT | hsa-miR-7274-3p |
| DLAT | hsa-miR-1603 |
| DLAT | hsa-miR-211-5p |
| DLAT | hsa-miR-6516-5p |
| DLAT | hsa-miR-5002-5p |
| DLAT | hsa-miR-6931-5p |
| DLAT | hsa-miR-6886-3p |
| DLAT | hsa-miR-2332 |
| DLAT | hsa-let-7f-1-3p |
| DLAT | hsa-miR-452-5p |
| DLAT | hsa-miR-7252-5p |
| DLAT | hsa-miR-548d-5p |
| DLAT | hsa-miR-599 |
| DLAT | hsa-miR-409-3p |
| DLAT | hsa-miR-6961-5p |
| DLAT | hsa-miR-410 |
| DLAT | hsa-miR-148-5p |
| DLAT | hsa-miR-1343-3p |
| DLAT | hsa-miR-1185-5p |
| DLAT | hsa-miR-6504-3p |
| DLAT | hsa-miR-4803 |
| DLAT | hsa-miR-376a-5p |
| DLAT | hsa-miR-5132-5p |
| DLAT | hsa-miR-561-5p |
| DLAT | hsa-miR-6397 |
| DLAT | hsa-miR-7328-3p |
| DLAT | hsa-miR-1271-5p |
| DLAT | hsa-miR-1904 |
| DLAT | hsa-miR-4520-3p |
| DLAT | hsa-miR-1721 |
| DLAT | hsa-miR-6833-3p |
| DLAT | hsa-miR-2320-3p |
| DLAT | hsa-miR-6887-5p |
| DLAT | hsa-miR-1596-5p |
| DLAT | hsa-miR-3524-3p |
| DLAT | hsa-miR-3173-3p |
| DLAT | hsa-miR-1249-5p |
| DLAT | hsa-miR-6905-3p |
| DLAT | hsa-miR-679 |
| DLAT | hsa-miR-7667-3p |
| DLAT | hsa-miR-573 |
| DLAT | hsa-miR-338-5p |
| DLAT | hsa-miR-3547-3p |
| DLAT | hsa-miR-4641 |
| DLAT | hsa-miR-610 |
| DLAT | hsa-miR-6891-5p |
| DLAT | hsa-miR-3123 |
| DLAT | hsa-miR-1296 |
| DLAT | hsa-miR-1250-3p |
| DLAT | hsa-miR-940 |
| DLAT | hsa-miR-6887-3p |
| DLAT | hsa-miR-7323-5p |
| DLAT | hsa-miR-199-1-5p |
| DLAT | hsa-miR-6801-5p |
| DLAT | hsa-miR-101a-5p |
| DLAT | hsa-miR-422 |
| DLAT | hsa-miR-2284a |
| DLAT | hsa-miR-6837-5p |
| DLAT | hsa-miR-708-3p |
| DLAT | hsa-miR-6348 |
| DLAT | hsa-miR-3075-3p |
| DLAT | hsa-miR-30-3p |
| DLAT | hsa-miR-6639-5p |
| DLAT | hsa-miR-873-5p |
| DLAT | hsa-miR-5692a |
| DLAT | hsa-miR-6931-3p |
| DLAT | hsa-miR-4294 |
| DLAT | hsa-miR-2284b |
| DLAT | hsa-miR-1256 |
| DLAT | hsa-miR-101-5p |
| DLAT | hsa-miR-29b-5p |
| DLAT | hsa-miR-487-5p |
| DLAT | hsa-miR-3161 |
| DLAT | hsa-miR-5684 |
| DLAT | hsa-miR-122-5p |
| DLAT | hsa-miR-5124a |
| DLAT | hsa-miR-3057-5p |
| DLAT | hsa-miR-409-5p |
| DLAT | hsa-miR-5094 |
| DLAT | hsa-miR-331-3p |
| DLAT | hsa-miR-377-3p |
| DLAT | hsa-miR-741-3p |
| DLAT | hsa-miR-16c-3p |
| DLAT | hsa-miR-7246-5p |
| DLAT | hsa-miR-8833 |
| DLAT | hsa-miR-8823 |
| DLAT | hsa-miR-6534 |
| DLAT | hsa-miR-548k |
| DLAT | hsa-miR-3094-3p |
| DLAT | hsa-miR-483-3p |
| DLAT | hsa-miR-6500-3p |
| DLAT | hsa-miR-519d-5p |
| DLAT | hsa-miR-2322-5p |
| DLAT | hsa-miR-6505-5p |
| DLAT | hsa-miR-7287-5p |
| DLAT | hsa-miR-223-3p |
| DLAT | hsa-miR-615-3p |
| DLAT | hsa-miR-548ak |
| DLAT | hsa-miR-7656-3p |
| DLAT | hsa-miR-1743 |
| DLAT | hsa-miR-6776-3p |
| DLAT | hsa-miR-4272 |
| DLAT | hsa-miR-9-3p |
| DLAT | hsa-miR-4774-5p |
| DLAT | hsa-miR-4780 |
| DLAT | hsa-miR-664-3p |
| DLAT | hsa-miR-544a |
| DLAT | hsa-miR-455-5p |
| DLAT | hsa-miR-130-5p |
| DLAT | hsa-miR-3160-5p |
| DLAT | hsa-miR-6853-3p |
| DLAT | hsa-miR-667-3p |
| DLAT | hsa-miR-892-3p |
| DLAT | hsa-miR-29a-2-5p |
| DLAT | hsa-miR-6794-5p |
| DLAT | hsa-miR-5680 |
| DLAT | hsa-miR-6654-3p |
| DLAT | hsa-miR-3190-3p |
| DLAT | hsa-miR-2376 |
| DLAT | hsa-miR-1542-5p |
| DLAT | hsa-miR-7159-5p |
| DLAT | hsa-miR-299 |
| DLAT | hsa-miR-7227-3p |
| DLAT | hsa-miR-6359 |
| DLAT | hsa-miR-3689a-5p |
| DLAT | hsa-miR-7364-3p |
| DLAT | hsa-miR-7174-5p |
| DLAT | hsa-miR-3122 |
| DLAT | hsa-miR-216a |
| DLAT | hsa-miR-3162-5p |
| DLAT | hsa-miR-4709-3p |
| DLAT | hsa-miR-632 |
| DLAT | hsa-miR-181-5p |
| DLAT | hsa-miR-609 |
| DLAT | hsa-miR-124c-5p |
| DLAT | hsa-miR-6961-3p |
| DLAT | hsa-miR-2363 |
| DLAT | hsa-miR-8898 |
| DLAT | hsa-miR-384 |
| DLAT | hsa-miR-381-3p |
| DLAT | hsa-miR-548h-5p |
| DLAT | hsa-miR-350 |
| DLAT | hsa-miR-140-3p.2 |
| DLAT | hsa-miR-545-3p |
| DLAT | hsa-miR-2054 |
| DLAT | hsa-miR-330-3p.2 |
| DLAT | hsa-miR-520c-3p |
| DLAT | hsa-miR-6074 |
| DLAT | hsa-miR-378d |
| DLAT | hsa-miR-299-5p |
| DLAT | hsa-miR-466b-4-3p |
| DLAT | hsa-miR-7021-5p |
| DLAT | hsa-miR-15b-3p |
| DLAT | hsa-miR-3192 |
| DLAT | hsa-miR-7026-5p |
| DLAT | hsa-miR-1552-5p |
| DLAT | hsa-miR-504-3p |
| DLAT | hsa-miR-520gh |
| DLAT | hsa-miR-6969-5p |
| DLAT | hsa-miR-548c-3p |
| DLAT | hsa-miR-3973 |
| DLAT | hsa-miR-125-3p |
| DLAT | hsa-miR-8076 |
| DLAT | hsa-miR-1545c-3p |
| DLAT | hsa-miR-217-5p |
| DLAT | hsa-miR-8873a |
| DLAT | hsa-miR-1837 |
| DLAT | hsa-miR-28-3p |
| DLAT | hsa-miR-568 |
| DLAT | hsa-miR-301-3p |
| DLAT | hsa-miR-3106-5p |
| DLAT | hsa-miR-633 |
| DLAT | hsa-miR-2320-5p |
| DLAT | hsa-miR-1546-3p |
| DLAT | hsa-miR-6878-5p |
| DLAT | hsa-miR-1540-5p |
| DLAT | hsa-miR-2459 |
| DLAT | hsa-miR-145-5p |
| DLAT | hsa-miR-3558-3p |
| DLAT | hsa-miR-3685 |
| DLAT | hsa-miR-203-3p.1 |
| DLAT | hsa-miR-376a-2-5p |
| DLAT | hsa-miR-654-5p |
| DLAT | hsa-miR-6772-5p |
| DLAT | hsa-miR-487b |
| DLAT | hsa-miR-141-3p |
| DLAT | hsa-miR-7686-5p |
| DLAT | hsa-miR-7655-3p |
| DLAT | hsa-miR-6926-5p |
| DLAT | hsa-miR-142-5p |
| DLAT | hsa-miR-152-3p |
| DLAT | hsa-miR-4643 |
| DLAT | hsa-miR-543 |
| DLAT | hsa-miR-7664-3p |
| DLAT | hsa-miR-2681-5p |
| DLAT | hsa-miR-467-3p |
| DLAT | hsa-miR-4477a |
| DLAT | hsa-miR-4753-3p |
| DLAT | hsa-miR-6608-3p |
| DLAT | hsa-miR-1178-5p |
| DLAT | hsa-miR-6932-3p |
| DLAT | hsa-miR-8085 |
| DLAT | hsa-miR-7393-5p |
| DLAT | hsa-miR-2901 |
| DLAT | hsa-miR-376a-1-5p |
| DLAT | hsa-miR-513 |
| DLAT | hsa-miR-548an |
| DLAT | hsa-miR-7214-3p |
| DLAT | hsa-miR-1632-5p |
| DLAT | hsa-miR-8854 |
| DLAT | hsa-miR-1710 |
| DLAT | hsa-miR-2285z |
| DLAT | hsa-miR-34b-5p |
| DLAT | hsa-miR-3561-3p |
| DLAT | hsa-miR-4685-5p |
| DLAT | hsa-miR-29-5p |
| DLAT | hsa-miR-767-5p |
| DLAT | hsa-miR-365-5p |
| DLAT | hsa-miR-697 |
| DLAT | hsa-miR-7286-3p |
| DLAT | hsa-miR-7850-5p |
| DLAT | hsa-miR-7254-3p |
| DLAT | hsa-miR-3913-5p |
| DLAT | hsa-miR-15c-3p |
| DLAT | hsa-miR-17-5p |
| DLAT | hsa-miR-618 |
| DLAT | hsa-miR-4446 |
| DLAT | hsa-miR-4304 |
| DLAT | hsa-miR-186-3p |
| DLAT | hsa-miR-6947-3p |
| DLAT | hsa-miR-7248-5p |
| DLAT | hsa-miR-2294 |
| DLAT | hsa-miR-669e-3p |
| DLAT | hsa-miR-7234-3p |
| DLAT | hsa-miR-3533 |
| DLAT | hsa-miR-8824 |
| DLAT | hsa-miR-3079-5p |
| DLAT | hsa-miR-383-3p |
| DLAT | hsa-miR-6620-5p |
| DLAT | hsa-miR-8063 |
| DLAT | hsa-miR-7078-3p |
| DLAT | hsa-miR-6565-5p |
| DLAT | hsa-miR-7385e-3p |
| DLAT | hsa-miR-1615 |
| DLAT | hsa-miR-7g-3p |
| DLAT | hsa-miR-30c-2-3p |
| DLAT | hsa-miR-4764-3p |
| DLAT | hsa-miR-1551-3p |
| DLAT | hsa-miR-1630 |
| DLAT | hsa-miR-191-5p |
| DLAT | hsa-miR-186-5p |
| DLAT | hsa-miR-6690-5p |
| DLAT | hsa-miR-6120-5p |
| DLAT | hsa-miR-4724-5p |
| DLAT | hsa-miR-6677-3p |
| DLAT | hsa-miR-342-3p |
| DLAT | hsa-miR-1627-5p |
| DLAT | hsa-miR-5192 |
| DLAT | hsa-miR-8485 |
| DLAT | hsa-miR-4728-3p |
| DLAT | hsa-miR-883ab-3p |
| DLAT | hsa-miR-1582 |
| DLAT | hsa-miR-7054-5p |
| DLAT | hsa-miR-468-5p |
| DLAT | hsa-miR-885 |
| DLAT | hsa-miR-3158-3p |
| DLAT | hsa-miR-887-5p |
| DLAT | hsa-miR-7325-3p |
| DLAT | hsa-miR-2406 |
| DLAT | hsa-miR-1786 |
| DLAT | hsa-miR-7274-5p |
| DLAT | hsa-miR-607 |
| DLAT | hsa-miR-6851-5p |
| DLAT | hsa-miR-6548-5p |
| DLAT | hsa-miR-623 |
| DLAT | hsa-miR-1305 |
| DLAT | hsa-miR-769-3p |
| DLAT | hsa-miR-1452 |
| DLAT | hsa-miR-502-5p |
| DLAT | hsa-miR-200c-5p |
| DLAT | hsa-miR-3658 |
| DLAT | hsa-miR-106-3p |
| DLAT | hsa-miR-760-5p |
| DLAT | hsa-miR-532-3p |
| DLAT | hsa-miR-449-3p |
| DLAT | hsa-miR-219-5p |
| DLAT | hsa-miR-6788-5p |
| DLAT | hsa-miR-28-5p |
| DLAT | hsa-miR-211-5p |
| DLAT | hsa-miR-7192-3p |
| DLAT | hsa-miR-7090-5p |
| DLAT | hsa-miR-559 |
| DLAT | hsa-miR-548b-3p |
| DLAT | hsa-miR-7081-3p |
| DLAT | hsa-miR-5092 |
| DLAT | hsa-miR-466bcp-3p |
| DLAT | hsa-miR-881-3p |
| DLAT | hsa-miR-4744 |
| DLAT | hsa-miR-338-3p |
| DLAT | hsa-miR-29b-1-5p |
| DLAT | hsa-miR-6507-5p |
| DLAT | hsa-miR-224-5p |
| DLAT | hsa-miR-6946-5p |
| DLAT | hsa-miR-6950-3p |
| DLAT | hsa-miR-7350-5p |
| DLAT | hsa-miR-3135b |
| DLAT | hsa-miR-7656-5p |
| DLAT | hsa-miR-191-5p |
| DLAT | hsa-miR-709 |
| DLAT | hsa-miR-1948-5p |
| DLAT | hsa-miR-6321 |
| DLAT | hsa-miR-423-5p |
| DLAT | hsa-miR-3173-3p |
| DLAT | hsa-miR-6316 |
| DLAT | hsa-miR-1722-3p |
| DLAT | hsa-miR-193 |
| DLAT | hsa-miR-513a |
| DLAT | hsa-miR-664b-5p |
| DLAT | hsa-miR-551b-5p |
| DLAT | hsa-miR-7687-3p |
| DLAT | hsa-miR-7201-3p |
| DLAT | hsa-miR-2285 |
| DLAT | hsa-let-7g-3p |
| DLAT | hsa-miR-8797 |
| DLAT | hsa-miR-3192-5p |
| DLAT | hsa-miR-1191a |
| DLAT | hsa-miR-7235-5p |
| DLAT | hsa-miR-548au-5p |
| DLAT | hsa-miR-3679-3p |
| DLAT | hsa-miR-872-5p |
| DLAT | hsa-miR-7978 |
| DLAT | hsa-miR-6809-3p |
| DLAT | hsa-miR-141-3p |
| DLAT | hsa-miR-6131 |
| DLAT | hsa-miR-5007-3p |
| DLAT | hsa-miR-4524b-3p |
| DLAT | hsa-miR-7975 |
| DLAT | hsa-miR-219a-2-3p |
| DLAT | hsa-miR-3692-3p |
| DLAT | hsa-miR-548a-5p |
| DLAT | hsa-miR-548am-5p |
| DLAT | hsa-miR-1544b-5p |
| DLAT | hsa-miR-7232-3p |
| DLAT | hsa-miR-382-5p |
| DLAT | hsa-miR-653 |
| DLAT | hsa-miR-8107 |
| DLAT | hsa-miR-5585-5p |
| DLAT | hsa-miR-548q |
| DLAT | hsa-miR-7237-3p |
| DLAT | hsa-miR-6963-5p |
| DLAT | hsa-miR-548ay-5p |
| DLAT | hsa-miR-30-5p |
| DLAT | hsa-miR-4666a-5p |
| DLAT | hsa-miR-7342-5p |
| DLAT | hsa-miR-7018-5p |
| DLAT | hsa-miR-1726 |
| DLAT | hsa-miR-542-3p |
| DLAT | hsa-miR-7275-3p |
| DLAT | hsa-miR-2284x |
| DLAT | hsa-miR-1296-5p |
| DLAT | hsa-miR-4789-3p |
| DLAT | hsa-miR-147a |
| DLAT | hsa-miR-7334-3p |
| DLAT | hsa-miR-3121-3p |
| DLAT | hsa-miR-369-3p |
| DLAT | hsa-miR-1434-3p |
| DLAT | hsa-miR-148b-5p |
| DLAT | hsa-miR-16-2-3p |
| DLAT | hsa-miR-2409 |
| DLAT | hsa-miR-6715b-3p |
| DLAT | hsa-miR-450b |
| DLAT | hsa-miR-8883 |
| DLAT | hsa-miR-7173-3p |
| DLAT | hsa-miR-466c-3p |
| DLAT | hsa-miR-26b-3p |
| DLAT | hsa-miR-21-3p |
| DLAT | hsa-miR-5121 |
| DLAT | hsa-miR-140-3p.1 |
| DLAT | hsa-miR-144-3p |
| DLAT | hsa-miR-548b-5p |
| DLAT | hsa-miR-7044-5p |
| DLAT | hsa-miR-873-3p |
| DLAT | hsa-miR-875 |
| DLAT | hsa-miR-6374 |
| DLAT | hsa-miR-877-5p |
| DLAT | hsa-miR-7011-3p |
| DLAT | hsa-miR-669h-5p |
| DLAT | hsa-miR-466ade-3p |
| DLAT | hsa-miR-7371b-3p |
| DLAT | hsa-miR-451a |
| DLAT | hsa-miR-290b-5p |
| DLAT | hsa-miR-134-3p |
| DLAT | hsa-miR-450-5p |
| DLAT | hsa-miR-16-1-3p |
| DLAT | hsa-miR-335-5p |
| DLAT | hsa-miR-7386i-5p |
| DLAT | hsa-miR-301b-5p |
| DLAT | hsa-miR-4775 |
| DLAT | hsa-miR-7371f-5p |
| DLAT | hsa-miR-7342-3p |
| DLAT | hsa-miR-6558-5p |
| DLAT | hsa-miR-3145-5p |
| DLAT | hsa-miR-1756b |
| DLAT | hsa-miR-150-5p |
| DLAT | hsa-miR-16-3p |
| DLAT | hsa-miR-7380-5p |
| DLAT | hsa-miR-197-3p |
| DLAT | hsa-miR-1960 |
| DLAT | hsa-miR-8054 |
| DLAT | hsa-miR-1981-3p |
| DLAT | hsa-miR-2468 |
| DLAT | hsa-miR-873b |
| DLAT | hsa-miR-548 |
| DLAT | hsa-miR-1843b-5p |
| DLAT | hsa-miR-6976-3p |
| DLAT | hsa-miR-2371 |
| DLAT | hsa-miR-7359-5p |
| DLAT | hsa-miR-138-3p |
| DLAT | hsa-miR-522-3p |
| DLAT | hsa-miR-651 |
| DLAT | hsa-miR-8888 |
| DLAT | hsa-miR-802 |
| DLAT | hsa-miR-181a-3p |
| DLAT | hsa-miR-2421 |
| DLAT | hsa-miR-126a-5p |
| DLAT | hsa-miR-3086-5p |
| DLAT | hsa-miR-2387 |
| DLAT | hsa-miR-642b-5p |
| DLAT | hsa-miR-210-5p |
| DLAT | hsa-miR-494 |
| DLAT | hsa-miR-4670-3p |
| DLAT | hsa-miR-411-3p |
| DLAT | hsa-miR-1663-3p |
| DLAT | hsa-miR-4698 |
| DLAT | hsa-miR-1248 |
| DLAT | hsa-miR-4761-5p |
| DLAT | hsa-miR-2957 |
| DLAT | hsa-miR-1942 |
| DLAT | hsa-miR-628-3p |
| DLAT | hsa-miR-2329-5p |
| DLAT | hsa-miR-6531 |
| DLAT | hsa-miR-374a-3p |
| DLAT | hsa-miR-7297-3p |
| DLAT | hsa-miR-369-3p |
| DLAT | hsa-miR-460-5p |
| DLAT | hsa-miR-365-3p |
| DLAT | hsa-miR-679-5p |
| DLAT | hsa-miR-376b-3p |
| DLAT | hsa-miR-2311 |
| DLAT | hsa-miR-3167-5p |
| DLAT | hsa-miR-519e-3p |
| DLAT | hsa-miR-383-5p.2 |
| DLAT | hsa-miR-499 |
| DLAT | hsa-miR-6327 |
| DLAT | hsa-miR-1271-5p |
| DLAT | hsa-miR-7056-3p |
| DLAT | hsa-miR-1279 |
| DLAT | hsa-miR-466b-3p |
| DLAT | hsa-miR-449c-5p |
| DLD | hsa-miR-130-3p |
| DLD | hsa-miR-5196-5p |
| DLD | hsa-miR-3665 |
| DLD | hsa-miR-652-5p |
| DLD | hsa-miR-6827-5p |
| DLD | hsa-miR-2285b |
| DLD | hsa-miR-3682-5p |
| DLD | hsa-miR-4477b |
| DLD | hsa-miR-6516-3p |
| DLD | hsa-miR-2399-5p |
| DLD | hsa-miR-2480 |
| DLD | hsa-miR-1839-5p |
| DLD | hsa-miR-600 |
| DLD | hsa-miR-6797-5p |
| DLD | hsa-miR-1232 |
| DLD | hsa-miR-7387-3p |
| DLD | hsa-miR-4796-5p |
| DLD | hsa-miR-1910-3p |
| DLD | hsa-miR-2466-3p |
| DLD | hsa-miR-7385f-3p |
| DLD | hsa-miR-2382-5p |
| DLD | hsa-miR-506-3p |
| DLD | hsa-miR-6996-3p |
| DLD | hsa-miR-125 |
| DLD | hsa-miR-5011-3p |
| DLD | hsa-miR-2285x |
| DLD | hsa-miR-7665-5p |
| DLD | hsa-miR-146-5p |
| DLD | hsa-miR-619-5p |
| DLD | hsa-miR-4740-5p |
| DLD | hsa-miR-365a-3p |
| DLD | hsa-miR-2483-3p |
| DLD | hsa-miR-3667-3p |
| DLD | hsa-miR-3590-3p |
| DLD | hsa-miR-7371d-5p |
| DLD | hsa-miR-4714-3p |
| DLD | hsa-miR-759 |
| DLD | hsa-miR-2310 |
| DLD | hsa-miR-6835-5p |
| DLD | hsa-miR-4668-3p |
| DLD | hsa-miR-4507 |
| DLD | hsa-miR-6743-5p |
| DLD | hsa-miR-2861 |
| DLD | hsa-miR-4763-5p |
| DLD | hsa-miR-3065-5p |
| DLD | hsa-miR-6929-5p |
| DLD | hsa-miR-1-5p |
| DLD | hsa-miR-2411-3p |
| DLD | hsa-miR-6132 |
| DLD | hsa-miR-577-3p |
| DLD | hsa-miR-4460 |
| DLD | hsa-miR-880-5p |
| DLD | hsa-miR-3938 |
| DLD | hsa-miR-196a-2-3p |
| DLD | hsa-miR-4473 |
| DLD | hsa-miR-7847-3p |
| DLD | hsa-miR-202-3p |
| DLD | hsa-miR-687 |
| DLD | hsa-miR-5693 |
| DLD | hsa-miR-4709-3p |
| DLD | hsa-miR-3612 |
| DLD | hsa-miR-4307 |
| DLD | hsa-miR-3680-3p |
| DLD | hsa-miR-107 |
| DLD | hsa-miR-497-3p |
| DLD | hsa-miR-4287 |
| DLD | hsa-miR-27 |
| DLD | hsa-miR-1976 |
| DLD | hsa-miR-1388-3p |
| DLD | hsa-miR-5619-3p |
| DLD | hsa-miR-7-5p |
| DLD | hsa-miR-101a-5p |
| DLD | hsa-miR-6513-5p |
| DLD | hsa-miR-5098 |
| DLD | hsa-miR-4323 |
| DLD | hsa-miR-2424 |
| DLD | hsa-miR-2442 |
| DLD | hsa-miR-6216 |
| DLD | hsa-miR-4719 |
| DLD | hsa-miR-3192-3p |
| DLD | hsa-miR-23-3p |
| DLD | hsa-miR-2489 |
| DLD | hsa-miR-627-3p |
| DLD | hsa-miR-7386m-3p |
| DLD | hsa-miR-194-5p |
| DLD | hsa-miR-8871 |
| DLD | hsa-miR-4794 |
| DLD | hsa-miR-543-3p |
| DLD | hsa-miR-7284-3p |
| DLD | hsa-miR-3686 |
| DLD | hsa-miR-139-5p |
| DLD | hsa-miR-6730-3p |
| DLD | hsa-miR-6836-5p |
| DLD | hsa-miR-3616-5p |
| DLD | hsa-miR-6832-5p |
| DLD | hsa-miR-6514-5p |
| DLD | hsa-miR-508-3p |
| DLD | hsa-miR-190-3p |
| DLD | hsa-miR-7370-3p |
| DLD | hsa-miR-570-3p |
| DLD | hsa-miR-2284z |
| DLD | hsa-miR-6834-5p |
| DLD | hsa-miR-412 |
| DLD | hsa-miR-2284d |
| DLD | hsa-miR-1238-3p |
| DLD | hsa-miR-6079 |
| DLD | hsa-miR-7386-5p |
| DLD | hsa-miR-548y |
| DLD | hsa-miR-3664-3p |
| DLD | hsa-miR-3124-3p |
| DLD | hsa-miR-378 |
| DLD | hsa-miR-3672 |
| DLD | hsa-miR-24-3p |
| DLD | hsa-miR-187-5p |
| DLD | hsa-miR-7257-5p |
| DLD | hsa-miR-5118 |
| DLD | hsa-miR-2297 |
| DLD | hsa-miR-3924 |
| DLD | hsa-miR-6529 |
| DLD | hsa-miR-7151-3p |
| DLD | hsa-miR-2352 |
| DLD | hsa-miR-29-3p |
| DLD | hsa-miR-149-3p |
| DLD | hsa-miR-548aj-5p |
| DLD | hsa-miR-181c-3p |
| DLD | hsa-miR-409 |
| DLD | hsa-miR-6840-3p |
| DLD | hsa-miR-7244-3p |
| DLD | hsa-miR-548ad-5p |
| DLD | hsa-miR-1972 |
| DLD | hsa-miR-655-5p |
| DLD | hsa-miR-31-3p |
| DLD | hsa-miR-216b-3p |
| DLD | hsa-miR-8103 |
| DLD | hsa-miR-6122-5p |
| DLD | hsa-miR-194-3p |
| DLD | hsa-miR-548bb-5p |
| DLD | hsa-miR-218-5p |
| DLD | hsa-miR-212-3p |
| DLD | hsa-miR-1297 |
| DLD | hsa-miR-6764-5p |
| DLD | hsa-miR-3613b |
| DLD | hsa-miR-6919-5p |
| DLD | hsa-miR-296-5p |
| DLD | hsa-miR-7340-5p |
| DLD | hsa-miR-1229-3p |
| DLD | hsa-miR-7450-3p |
| DLD | hsa-miR-7232-5p |
| DLD | hsa-miR-452-3p |
| DLD | hsa-miR-24-3p |
| DLD | hsa-miR-1591-5p |
| DLD | hsa-miR-3619-5p |
| DLD | hsa-miR-144-3p |
| DLD | hsa-miR-6903-5p |
| DLD | hsa-miR-3068-5p |
| DLD | hsa-miR-499a-3p |
| DLD | hsa-miR-146 |
| DLD | hsa-miR-19a-5p |
| DLD | hsa-miR-631 |
| DLD | hsa-miR-4688 |
| DLD | hsa-miR-548ak |
| DLD | hsa-miR-138-2-3p |
| DLD | hsa-let-7-3p |
| DLD | hsa-miR-6756-5p |
| DLD | hsa-miR-8805 |
| DLD | hsa-miR-650 |
| DLD | hsa-miR-214-3p |
| DLD | hsa-miR-2284aa |
| DLD | hsa-miR-4638-3p |
| DLD | hsa-miR-1-3p |
| DLD | hsa-miR-7271-5p |
| DLD | hsa-miR-7006-3p |
| DLD | hsa-miR-7664-3p |
| DLD | hsa-miR-7365-3p |
| DLD | hsa-miR-6909-5p |
| DLD | hsa-miR-207 |
| DLD | hsa-miR-487b-5p |
| DLD | hsa-miR-5589-3p |
| DLD | hsa-miR-7641 |
| DLD | hsa-miR-320a |
| DLD | hsa-miR-548ah-3p |
| DLD | hsa-miR-343 |
| DLD | hsa-miR-7110-5p |
| DLD | hsa-miR-4259 |
| DLD | hsa-miR-1197 |
| DLD | hsa-miR-2379 |
| DLD | hsa-miR-584-5p |
| DLD | hsa-miR-512-3p |
| DLD | hsa-miR-4433a-3p |
| DLD | hsa-miR-378a-5p |
| DLD | hsa-miR-376c-5p |
| DLD | hsa-miR-4652-3p |
| DLD | hsa-miR-5583-5p |
| DLD | hsa-miR-3653-5p |
| DLD | hsa-miR-450b-3p |
| DLD | hsa-miR-5586-3p |
| DLD | hsa-miR-1546-3p |
| DLD | hsa-miR-5000-3p |
| DLD | hsa-miR-1207 |
| DLD | hsa-miR-4726-3p |
| DLD | hsa-miR-4752 |
| DLD | hsa-miR-3140-3p |
| DLD | hsa-miR-2291 |
| DLD | hsa-miR-876 |
| DLD | hsa-miR-96-3p |
| DLD | hsa-miR-323a-3p |
| DLD | hsa-miR-1983 |
| DLD | hsa-miR-194-2-3p |
| DLD | hsa-miR-7366-5p |
| DLD | hsa-miR-2277-3p |
| DLD | hsa-miR-2325 |
| DLD | hsa-miR-3187-3p |
| DLD | hsa-miR-6731-3p |
| DLD | hsa-miR-7114-5p |
| DLD | hsa-miR-18a-3p |
| DLD | hsa-miR-671-5p |
| DLD | hsa-miR-507 |
| DLD | hsa-miR-676-3p |
| DLD | hsa-miR-556-5p |
| DLD | hsa-miR-548c-5p |
| DLD | hsa-miR-545-3p |
| DLD | hsa-miR-1264-3p |
| DLD | hsa-miR-4738-3p |
| DLD | hsa-miR-3569-5p |
| DLD | hsa-miR-5001-3p |
| DLD | hsa-miR-93-3p |
| DLD | hsa-miR-500-3p |
| DLD | hsa-miR-2365 |
| DLD | hsa-miR-5132-3p |
| DLD | hsa-miR-8860 |
| DLD | hsa-miR-7039-3p |
| DLD | hsa-miR-2285j |
| DLD | hsa-miR-5194 |
| DLD | hsa-miR-6891-5p |
| DLD | hsa-miR-490-3p |
| DLD | hsa-miR-425-5p |
| DLD | hsa-miR-340-5p |
| DLD | hsa-miR-204-5p |
| DLD | hsa-miR-6742-3p |
| DLD | hsa-miR-938 |
| DLD | hsa-miR-302b-5p |
| DLD | hsa-miR-380-3p |
| DLD | hsa-miR-4703-3p |
| DLD | hsa-miR-301-5p |
| DLD | hsa-miR-9b-3p |
| DLD | hsa-miR-2330-3p |
| DLD | hsa-miR-4275 |
| DLD | hsa-miR-7020-3p |
| DLD | hsa-miR-383 |
| DLD | hsa-miR-381-3p |
| DLD | hsa-miR-5587-5p |
| DLD | hsa-miR-4511 |
| DLD | hsa-miR-378b |
| DLD | hsa-miR-692 |
| DLD | hsa-miR-455-5p |
| DLD | hsa-miR-6516 |
| DLD | hsa-miR-4694-3p |
| DLD | hsa-miR-625-3p |
| DLD | hsa-miR-4760-3p |
| DLD | hsa-miR-499b-3p |
| DLD | hsa-miR-7262a-5p |
| DLD | hsa-miR-330-3p |
| DLD | hsa-miR-677-5p |
| DLD | hsa-miR-548ah-5p |
| DLD | hsa-miR-513c-3p |
| DLD | hsa-miR-208-5p |
| DLD | hsa-miR-2376 |
| DLD | hsa-miR-1-3p |
| DLD | hsa-miR-6798-5p |
| DLD | hsa-miR-380 |
| DLD | hsa-miR-7383-5p |
| DLD | hsa-miR-132-3p |
| DLD | hsa-miR-761 |
| DLD | hsa-miR-204-3p |
| DLD | hsa-miR-5681a |
| DLD | hsa-miR-5011-5p |
| DLD | hsa-miR-382-5p |
| DLD | hsa-miR-6343 |
| DLD | hsa-miR-3431 |
| DLD | hsa-miR-5004-5p |
| DLD | hsa-miR-3572-5p |
| DLD | hsa-miR-3136-5p |
| DLD | hsa-miR-6741-5p |
| DLD | hsa-miR-4732-3p |
| DLD | hsa-miR-8843 |
| DLD | hsa-miR-1306-3p |
| DLD | hsa-miR-6749-3p |
| DLD | hsa-miR-8841 |
| DLD | hsa-miR-6502-5p |
| DLD | hsa-miR-6780a-5p |
| DLD | hsa-miR-371 |
| DLD | hsa-miR-7302-5p |
| DLD | hsa-miR-520-3p |
| DLD | hsa-miR-6931-3p |
| DLD | hsa-miR-1255 |
| DLD | hsa-miR-5697-3p |
| DLD | hsa-miR-4774-3p |
| DLD | hsa-miR-511-5p |
| DLD | hsa-miR-548o-5p |
| DLD | hsa-miR-4282 |
| DLD | hsa-miR-608 |
| DLD | hsa-miR-5095 |
| DLD | hsa-miR-485-5p |
| DLD | hsa-miR-8868 |
| DLD | hsa-miR-506-3p |
| DLD | hsa-miR-2284-5p |
| DLD | hsa-miR-135a-2-3p |
| DLD | hsa-miR-664 |
| DLD | hsa-miR-583 |
| DLD | hsa-miR-1283a |
| DLD | hsa-miR-212-3p |
| DLD | hsa-miR-338-5p |
| DLD | hsa-miR-150-5p |
| DLD | hsa-miR-7977 |
| DLD | hsa-miR-4528 |
| DLD | hsa-miR-4667-3p |
| DLD | hsa-miR-548al |
| DLD | hsa-miR-2446 |
| DLD | hsa-miR-2355-3p |
| DLD | hsa-miR-1954 |
| DLD | hsa-miR-30 |
| DLD | hsa-miR-449a |
| DLD | hsa-miR-429 |
| DLD | hsa-miR-3964 |
| DLD | hsa-miR-6990-5p |
| DLD | hsa-miR-520 |
| DLD | hsa-miR-499b-3p |
| DLD | hsa-miR-323-3p |
| DLD | hsa-miR-7221-5p |
| DLD | hsa-miR-24-5p |
| DLD | hsa-miR-3166 |
| DLD | hsa-miR-3102-3p.2-3p |
| DLD | hsa-miR-7398r-3p |
| DLD | hsa-miR-2284v |
| DLD | hsa-miR-5101 |
| DLD | hsa-miR-7168-5p |
| DLD | hsa-miR-7170-5p |
| DLD | hsa-miR-204-5p |
| DLD | hsa-miR-149-5p |
| DLD | hsa-miR-7296-5p |
| DLD | hsa-miR-7186-3p |
| DLD | hsa-miR-1465 |
| DLD | hsa-miR-15a-3p |
| DLD | hsa-miR-5695 |
| DLD | hsa-miR-584-5p |
| DLD | hsa-miR-3170 |
| DLD | hsa-miR-7214-5p |
| DLD | hsa-miR-6728-5p |
| DLD | hsa-miR-219a-1-3p |
| DLD | hsa-miR-30e-3p |
| DLD | hsa-miR-548ap-3p |
| DLD | hsa-miR-6792-3p |
| DLD | hsa-miR-30e |
| DLD | hsa-miR-2318 |
| DLD | hsa-miR-505-3p.1 |
| DLD | hsa-miR-1909-3p |
| DLD | hsa-miR-3617-5p |
| DLD | hsa-miR-8819 |
| DLD | hsa-miR-6937-3p |
| DLD | hsa-miR-511 |
| DLD | hsa-miR-669n |
| DLD | hsa-miR-8908d |
| DLD | hsa-miR-1915-3p |
| DLD | hsa-miR-513b-5p |
| DLD | hsa-miR-6727-3p |
| DLD | hsa-miR-493-5p |
| DLD | hsa-miR-1247-3p |
| DLD | hsa-miR-6825-5p |
| DLD | hsa-miR-6946-3p |
| DLD | hsa-miR-322-3p |
| DLD | hsa-miR-8904a |
| DLD | hsa-miR-412-3p |
| DLD | hsa-miR-196 |
| DLD | hsa-miR-7204-3p |
| DLD | hsa-miR-455-3p |
| DLD | hsa-miR-4742 |
| DLD | hsa-miR-3116 |
| DLD | hsa-miR-8890 |
| DLD | hsa-miR-7065-5p |
| DLD | hsa-miR-936 |
| DLD | hsa-miR-6841-3p |
| DLD | hsa-miR-7108-3p |
| DLD | hsa-miR-889 |
| DLD | hsa-miR-211-3p |
| DLD | hsa-miR-140-5p |
| DLD | hsa-miR-7318-5p |
| DLD | hsa-miR-5582-5p |
| DLD | hsa-miR-200bc-3p |
| DLD | hsa-miR-3675-5p |
| DLD | hsa-miR-4682 |
| DLD | hsa-miR-8800 |
| DLD | hsa-miR-30c-5p |
| DLD | hsa-miR-5096 |
| DLD | hsa-miR-505-3p |
| DLD | hsa-miR-677 |
| DLD | hsa-miR-3935 |
| DLD | hsa-miR-7182-3p |
| DLD | hsa-miR-4446-5p |
| DLD | hsa-miR-294 |
| DLD | hsa-miR-873-5p.1 |
| DLD | hsa-miR-372-3p |
| DLD | hsa-miR-196-5p |
| DLD | hsa-miR-6377 |
| DLD | hsa-miR-6829-3p |
| DLD | hsa-miR-769-3p |
| DLD | hsa-miR-665 |
| DLD | hsa-miR-30a-5p |
| DLD | hsa-miR-345-5p |
| DLD | hsa-miR-7172-5p |
| DLD | hsa-miR-19-5p |
| DLD | hsa-miR-935 |
| DLD | hsa-miR-3671 |
| DLD | hsa-miR-3617-3p |
| DLD | hsa-miR-539-5p |
| DLD | hsa-miR-3133 |
| DLD | hsa-miR-1277-5p |
| DLD | hsa-miR-487-5p |
| DLD | hsa-miR-374-5p |
| DLD | hsa-miR-7357-5p |
| DLD | hsa-miR-1273h-3p |
| DLD | hsa-miR-2284c |
| DLD | hsa-miR-6734-5p |
| DLD | hsa-miR-487a |
| DLD | hsa-miR-106-5p |
| DLD | hsa-miR-192-5p |
| DLD | hsa-miR-3529-3p |
| DLD | hsa-miR-4691-5p |
| DLD | hsa-miR-6969-3p |
| DLD | hsa-miR-524-5p |
| DLD | hsa-miR-573 |
| DLD | hsa-miR-1275 |
| DLD | hsa-miR-6731-5p |
| DLD | hsa-miR-16-5p |
| DLD | hsa-miR-590-5p |
| DLD | hsa-miR-7153-3p |
| DLD | hsa-miR-6737-3p |
| DLD | hsa-miR-8113 |
| DLD | hsa-miR-6083 |
| DLD | hsa-miR-499-5p |
| DLD | hsa-miR-135a-1-3p |
| DLD | hsa-miR-33 |
| DLD | hsa-miR-4727-5p |
| DLD | hsa-miR-7022-3p |
| DLD | hsa-miR-548bb-3p |
| DLD | hsa-miR-465a-5p |
| DLD | hsa-miR-628-3p |
| DLD | hsa-miR-2284y |
| DLD | hsa-miR-7061-5p |
| DLD | hsa-miR-7848-3p |
| DLD | hsa-miR-2369 |
| DLD | hsa-miR-3105-3p |
| DLD | hsa-miR-2284k |
| DLD | hsa-miR-7070-5p |
| DLD | hsa-miR-183-5p.2 |
| DLD | hsa-miR-3138 |
| DLD | hsa-miR-7160-5p |
| DLD | hsa-miR-328-5p |
| DLD | hsa-miR-6500-5p |
| DLD | hsa-miR-4268 |
| DLD | hsa-miR-2465 |
| DLD | hsa-miR-466aep-5p |
| DLD | hsa-miR-2317 |
| DLD | hsa-miR-1233-5p |
| DLD | hsa-miR-4643 |
| DLD | hsa-miR-6511-5p |
| DLD | hsa-miR-7329-5p |
| DLD | hsa-miR-4671-3p |
| DLD | hsa-miR-7210-3p |
| DLD | hsa-miR-2486-3p |
| DLD | hsa-miR-8847 |
| DLD | hsa-miR-502-5p |
| DLD | hsa-miR-505-3p.2 |
| DLD | hsa-miR-1545c-3p |
| DLD | hsa-miR-1343-5p |
| DLD | hsa-miR-208-3p |
| DLD | hsa-miR-2284j |
| DLD | hsa-miR-4672 |
| DLD | hsa-miR-6720-5p |
| DLD | hsa-miR-183-3p |
| DLD | hsa-miR-153-5p |
| DLD | hsa-miR-633 |
| DLD | hsa-miR-6754-3p |
| DLD | hsa-miR-421 |
| DLD | hsa-miR-2431-3p |
| DLD | hsa-miR-485-3p |
| DLD | hsa-miR-3179 |
| DLD | hsa-miR-4802-5p |
| DLD | hsa-miR-1252-5p |
| DLD | hsa-miR-4715-5p |
| DLD | hsa-miR-7158-3p |
| DLD | hsa-miR-6759-3p |
| DLD | hsa-miR-3140-3p |
| DLD | hsa-miR-23a-3p |
| DLD | hsa-miR-3151-3p |
| DLD | hsa-miR-4651 |
| DLD | hsa-miR-548e-3p |
| DLD | hsa-miR-1298-3p |
| DLD | hsa-miR-1271-5p |
| DLD | hsa-miR-766-3p |
| DLD | hsa-miR-465-5p |
| DLD | hsa-miR-7341-3p |
| DLD | hsa-miR-547-5p |
| DLD | hsa-miR-514-3p |
| DLD | hsa-miR-763 |
| DLD | hsa-miR-3120-3p |
| DLD | hsa-miR-9-5p |
| DLD | hsa-miR-548aj-3p |
| DLD | hsa-miR-7073-5p |
| DLD | hsa-miR-548ar-5p |
| DLD | hsa-miR-547-3p |
| DLD | hsa-miR-619 |
| DLD | hsa-miR-3572 |
| DLD | hsa-miR-4668-5p |
| DLD | hsa-miR-4725-3p |
| DLD | hsa-miR-7386k-3p |
| DLD | hsa-miR-1287-5p |
| DLD | hsa-miR-8062 |
| DLD | hsa-miR-4661-5p |
| DLD | hsa-miR-6722-3p |
| DLD | hsa-miR-1982-3p |
| DLD | hsa-miR-674-5p |
| DLD | hsa-miR-1207-3p |
| DLD | hsa-miR-2284ab |
| DLD | hsa-miR-2293 |
| DLD | hsa-miR-3169 |
| DLD | hsa-miR-605-3p |
| DLD | hsa-miR-3938-3p |
| DLD | hsa-miR-6768-3p |
| DLD | hsa-miR-7398u-3p |
| DLD | hsa-miR-129-5p |
| DLD | hsa-miR-2363 |
| DLD | hsa-miR-4657 |
| DLD | hsa-miR-1284 |
| DLD | hsa-miR-2285q |
| DLD | hsa-miR-6331 |
| DLD | hsa-miR-494-3p |
| DLD | hsa-miR-6855-3p |
| DLD | hsa-miR-33-5p |
| DLD | hsa-miR-655-3p |
| DLD | hsa-miR-522-3p |
| DLD | hsa-miR-707 |
| DLD | hsa-miR-1548-3p |
| DLD | hsa-miR-376abd |
| DLD | hsa-miR-2366 |
| DLD | hsa-miR-576-5p |
| DLD | hsa-miR-6513-3p |
| DLD | hsa-miR-3970 |
| DLD | hsa-miR-1179 |
| DLD | hsa-miR-548l |
| DLD | hsa-miR-1827 |
| DLD | hsa-miR-3544-3p |
| DLD | hsa-miR-5006-5p |
| DLD | hsa-miR-377-3p |
| DLD | hsa-let-7f-2-3p |
| DLD | hsa-miR-1296 |
| DLD | hsa-miR-146a-3p |
| DLD | hsa-miR-541-5p |
| DLD | hsa-miR-2397-3p |
| DLD | hsa-miR-298 |
| DLD | hsa-miR-486-5p |
| DLD | hsa-miR-555 |
| DLD | hsa-miR-4289 |
| DLD | hsa-miR-7046-5p |
| DLD | hsa-miR-1903 |
| DLD | hsa-miR-3689d |
| DLD | hsa-miR-1838 |
| DLD | hsa-miR-1306-3p |
| DLD | hsa-miR-1343 |
| DLD | hsa-miR-8838 |
| DLD | hsa-miR-1894-5p |
| DLD | hsa-miR-6927-5p |
| DLD | hsa-miR-7196-3p |
| DLD | hsa-miR-8872 |
| DLD | hsa-miR-4633-3p |
| DLD | hsa-miR-1943-5p |
| DLD | hsa-miR-7344-3p |
| DLD | hsa-miR-6791-3p |
| DLD | hsa-miR-890-5p |
| DLD | hsa-miR-149-5p |
| DLD | hsa-miR-7180 |
| DLD | hsa-miR-6851-5p |
| DLD | hsa-miR-3978 |
| DLD | hsa-miR-6520 |
| DLD | hsa-miR-1256 |
| DLD | hsa-miR-7086-5p |
| DLD | hsa-miR-3674 |
| DLD | hsa-miR-8905 |
| DLD | hsa-miR-1597-5p |
| DLD | hsa-miR-3573-5p |
| DLD | hsa-miR-5579-5p |
| DLD | hsa-miR-190a-3p |
| DLD | hsa-miR-497-5p |
| DLD | hsa-miR-1225-5p |
| DLD | hsa-miR-8075 |
| DLD | hsa-miR-1278 |
| DLD | hsa-miR-432-5p |
| DLD | hsa-miR-1237-3p |
| DLD | hsa-miR-548n |
| DLD | hsa-miR-6388 |
| DLD | hsa-miR-5683 |
| DLD | hsa-miR-548an |
| DLD | hsa-miR-4655-5p |
| DLD | hsa-miR-5582-3p |
| DLD | hsa-miR-6715a-3p |
| DLD | hsa-miR-3201 |
| DLD | hsa-miR-2397-5p |
| DLD | hsa-miR-891 |
| DLD | hsa-miR-142b |
| DLD | hsa-miR-495-3p |
| DLD | hsa-miR-1272 |
| DLD | hsa-miR-7180-3p |
| DLD | hsa-miR-181b-1-3p |
| DLD | hsa-miR-1781-3p |
| DLD | hsa-miR-5125-5p |
| DLD | hsa-miR-3667-5p |
| DLD | hsa-miR-298-3p |
| DLD | hsa-miR-2284n |
| DLD | hsa-miR-3130-3p |
| DLD | hsa-miR-6771-3p |
| DLD | hsa-miR-200c-3p |
| DLD | hsa-miR-5186 |
| DLD | hsa-miR-105-5p |
| DLD | hsa-miR-6526 |
| DLD | hsa-miR-4755-5p |
| DLD | hsa-miR-297 |
| DLD | hsa-miR-6766-5p |
| DLD | hsa-miR-2285d |
| DLD | hsa-miR-548x-3p |
| DLD | hsa-miR-503-3p |
| DLD | hsa-miR-1323 |
| DLD | hsa-miR-4704-5p |
| DLD | hsa-miR-92a-2-5p |
| DLD | hsa-miR-1677-3p |
| DLD | hsa-miR-8807 |
| DLD | hsa-miR-7473-5p |
| DLD | hsa-miR-548az-3p |
| DLD | hsa-miR-129-5p |
| DLD | hsa-miR-6769a-3p |
| DLD | hsa-miR-891b |
| DLD | hsa-miR-544b |
| DLD | hsa-miR-877-3p |
| DLD | hsa-miR-487a-5p |
| DLD | hsa-miR-199 |
| DLD | hsa-miR-1930-5p |
| DLD | hsa-miR-2443 |
| DLD | hsa-miR-7386d-3p |
| DLD | hsa-miR-1283 |
| DLD | hsa-miR-4747-5p |
| DLD | hsa-miR-4999-5p |
| DLD | hsa-miR-183-5p |
| DLD | hsa-miR-668-3p |
| DLD | hsa-miR-877-5p |
| DLD | hsa-miR-501-3p |
| DLD | hsa-miR-5110 |
| DLD | hsa-miR-4717-3p |
| DLD | hsa-miR-656-3p |
| DLD | hsa-miR-7260-3p |
| DLD | hsa-miR-561-5p |
| DLD | hsa-miR-2380 |
| DLD | hsa-miR-155-5p |
| DLD | hsa-miR-7055-5p |
| DLD | hsa-miR-8826 |
| DLD | hsa-miR-203 |
| DLD | hsa-miR-362-3p |
| DLD | hsa-miR-7685-5p |
| DLD | hsa-miR-2285c |
| DLD | hsa-miR-561-3p |
| DLD | hsa-miR-2957 |
| DLD | hsa-miR-1721 |
| DLD | hsa-miR-345-3p |
| DLD | hsa-miR-7281-3p |
| DLD | hsa-miR-1185-3p |
| DLD | hsa-miR-216a |
| DLD | hsa-miR-7227-3p |
| DLD | hsa-miR-4796-3p |
| DLD | hsa-miR-591 |
| DLD | hsa-miR-6715b-3p |
| DLD | hsa-miR-200 |
| DLD | hsa-miR-8824 |
| DLD | hsa-miR-3063-5p |
| DLD | hsa-miR-148-3p |
| DLD | hsa-miR-2284s |
| DLD | hsa-miR-5698 |
| DLD | hsa-miR-2420 |
| DLD | hsa-miR-23c |
| DLD | hsa-miR-6751-5p |
| DLD | hsa-miR-653-5p |
| DLD | hsa-miR-185-5p |
| DLD | hsa-miR-7163-5p |
| DLD | hsa-miR-8084 |
| DLD | hsa-miR-2464-5p |
| DLD | hsa-miR-548bd-3p |
| DLD | hsa-miR-6837-5p |
| DLD | hsa-miR-141-5p |
| DLD | hsa-miR-183-5p.1 |
| DLD | hsa-miR-3471 |
| DLD | hsa-miR-1304-3p |
| DLD | hsa-miR-103 |
| DLD | hsa-miR-219c-3p |
| DLD | hsa-miR-6739-5p |
| DLD | hsa-miR-548c-3p |
| DLD | hsa-miR-1927 |
| DLD | hsa-miR-190b-3p |
| DLD | hsa-miR-532-5p |
| DLD | hsa-miR-329-3p |
| DLD | hsa-miR-3606-3p |
| DLD | hsa-miR-3173-3p |
| DLD | hsa-miR-6871-5p |
| DLD | hsa-miR-7199-5p |
| DLD | hsa-miR-3977 |
| DLD | hsa-miR-6910-3p |
| DLD | hsa-miR-6787-3p |
| DLD | hsa-miR-514a-3p |
| DLD | hsa-miR-670 |
| DLD | hsa-miR-7378-3p |
| DLD | hsa-miR-3163 |
| DLD | hsa-miR-505-3p |
| DLD | hsa-miR-6981-3p |
| DLD | hsa-miR-2370-5p |
| DLD | hsa-miR-551b-5p |
| DLD | hsa-miR-4755-3p |
| DLD | hsa-miR-215-5p |
| DLD | hsa-miR-26-1-3p |
| DLD | hsa-miR-489-3p |
| DLD | hsa-miR-483-3p |
| DLD | hsa-miR-3914 |
| DLD | hsa-miR-33a-3p |
| DLD | hsa-miR-8114 |
| DLD | hsa-miR-6848-3p |
| DLD | hsa-miR-1246 |
| DLD | hsa-miR-6852-5p |
| DLD | hsa-miR-3944-5p |
| DLD | hsa-miR-4804-3p |
| DLD | hsa-miR-4759 |
| DLD | hsa-miR-4780 |
| DLD | hsa-miR-26-3-3p |
| DLD | hsa-miR-371a-5p |
| DLD | hsa-miR-587 |
| DLD | hsa-miR-6371 |
| DLD | hsa-miR-4646-3p |
| DLD | hsa-miR-4728-5p |
| DLD | hsa-miR-7262b-5p |
| DLD | hsa-miR-8816 |
| DLD | hsa-miR-6926-5p |
| DLD | hsa-miR-1323-5p |
| DLD | hsa-miR-7188-5p |
| DLD | hsa-miR-3547-5p |
| DLD | hsa-miR-2681-5p |
| DLD | hsa-miR-26a-3p |
| DLD | hsa-miR-3146 |
| DLD | hsa-miR-582-3p |
| DLD | hsa-miR-3190-5p |
| DLD | hsa-miR-302d-5p |
| DLD | hsa-miR-7195-5p |
| DLD | hsa-miR-29b-1-5p |
| DLD | hsa-miR-98-5p |
| DLD | hsa-miR-6822-5p |
| DLD | hsa-miR-7174-5p |
| DLD | hsa-miR-2358 |
| DLD | hsa-miR-4797-5p |
| DLD | hsa-miR-6736-3p |
| DLD | hsa-miR-544a |
| DLD | hsa-miR-6915-3p |
| DLD | hsa-miR-34a-3p |
| DLD | hsa-miR-4324 |
| DLD | hsa-miR-548ar-3p |
| DLD | hsa-miR-192-3p |
| DLD | hsa-miR-5192 |
| DLD | hsa-miR-449-5p |
| DLD | hsa-miR-890-3p |
| DLD | hsa-miR-7349-3p |
| DLD | hsa-miR-6780a-3p |
| DLD | hsa-miR-330-5p |
| DLD | hsa-miR-2284u |
| DLD | hsa-miR-4705 |
| DLD | hsa-miR-5583-3p |
| DLD | hsa-miR-147-5p |
| DLD | hsa-miR-1188-5p |
| DLD | hsa-miR-7852-3p |
| DLD | hsa-miR-6505-3p |
| DLD | hsa-miR-1258-3p |
| DLD | hsa-miR-8893 |
| DLD | hsa-miR-548ac |
| DLD | hsa-miR-7373b-3p |
| DLD | hsa-miR-6869-5p |
| DLD | hsa-miR-299-5p |
| DLD | hsa-miR-6868-3p |
| DLD | hsa-miR-222-5p |
| DLD | hsa-miR-6069 |
| DLD | hsa-miR-8803 |
| DLD | hsa-miR-374c-5p |
| DLD | hsa-miR-548g-5p |
| DLD | hsa-miR-329 |
| DLD | hsa-miR-466 |
| DLD | hsa-miR-7208-5p |
| DLD | hsa-miR-1298-5p |
| DLD | hsa-miR-143-3p |
| DLD | hsa-miR-7202-3p |
| DLD | hsa-miR-372 |
| DLD | hsa-miR-2284ac |
| DLD | hsa-miR-656-5p |
| DLD | hsa-miR-411a |
| DLD | hsa-miR-489 |
| DLD | hsa-miR-200a-3p |
| DLD | hsa-miR-1964-3p |
| DLD | hsa-miR-544-5p |
| DLD | hsa-miR-3149 |
| DLD | hsa-miR-4742-3p |
| DLD | hsa-miR-5087 |
| DLD | hsa-miR-548a-3p |
| DLD | hsa-miR-6866-5p |
| DLD | hsa-miR-4744 |
| DLD | hsa-miR-3082-3p |
| DLD | hsa-miR-23 |
| DLD | hsa-miR-1915-5p |
| DLD | hsa-miR-518a-5p |
| DLD | hsa-miR-3551-3p |
| DLD | hsa-miR-552-5p |
| DLD | hsa-miR-6803-5p |
| DLD | hsa-miR-7386i-3p |
| DLD | hsa-miR-4524a-5p |
| DLD | hsa-miR-181-3p |
| DLD | hsa-miR-137 |
| DLD | hsa-miR-6874-5p |
| DLD | hsa-miR-4424 |
| DLD | hsa-miR-939-3p |
| DLD | hsa-miR-183-5p |
| DLD | hsa-miR-3154 |
| DLD | hsa-miR-548as-5p |
| DLD | hsa-miR-3097-5p |
| DLD | hsa-miR-499b-5p |
| DLD | hsa-miR-10a-3p |
| DLD | hsa-miR-6323 |
| DLD | hsa-miR-4696 |
| DLD | hsa-miR-891a-3p |
| DLD | hsa-miR-124-3p.1 |
| DLD | hsa-miR-6502-3p |
| DLD | hsa-miR-742-5p |
| DLD | hsa-miR-6975-3p |
| DLD | hsa-miR-588 |
| DLD | hsa-miR-1301 |
| DLD | hsa-miR-6503-3p |
| DLD | hsa-miR-548t-5p |
| DLD | hsa-miR-582-5p |
| DLD | hsa-miR-6824-3p |
| DLD | hsa-miR-362-5p |
| DLD | hsa-miR-3121-3p |
| DLD | hsa-miR-6887-3p |
| DLD | hsa-miR-6826-5p |
| DLD | hsa-miR-454 |
| DLD | hsa-miR-376a-2-5p |
| DLD | hsa-miR-520g-3p |
| DLD | hsa-miR-127-5p |
| DLD | hsa-miR-17-5p |
| DLD | hsa-miR-26 |
| DLD | hsa-miR-4711-5p |
| DLD | hsa-miR-7195-3p |
| DLD | hsa-miR-411-5p.1 |
| DLD | hsa-miR-448-5p |
| DLD | hsa-miR-455-3p.1 |
| DLD | hsa-miR-188-5p |
| DLD | hsa-miR-223-5p |
| DLD | hsa-miR-1285-5p |
| DLD | hsa-miR-7065-3p |
| DLD | hsa-miR-1191b-5p |
| DLD | hsa-miR-6849-3p |
| DLD | hsa-miR-545-5p |
| DLD | hsa-miR-34c-5p |
| DLD | hsa-miR-875 |
| DLD | hsa-miR-590-3p |
| DLD | hsa-miR-5616-3p |
| DLD | hsa-miR-34 |
| DLD | hsa-miR-6905-3p |
| DLD | hsa-miR-597-3p |
| DLD | hsa-miR-3908 |
| DLD | hsa-miR-3976 |
| DLD | hsa-miR-221-3p |
| DLD | hsa-miR-1966-3p |
| DLD | hsa-miR-7398l-3p |
| DLD | hsa-miR-4513 |
| DLD | hsa-miR-8862 |
| DLD | hsa-miR-199-5p |
| DLD | hsa-miR-2444 |
| DLD | hsa-miR-330-3p |
| DLD | hsa-miR-8867 |
| DLD | hsa-miR-1667-3p |
| DLD | hsa-miR-2285z |
| DLD | hsa-miR-132-3p |
| DLD | hsa-let-7f-1-3p |
| DLD | hsa-miR-2285r |
| DLD | hsa-miR-181-5p |
| DLD | hsa-miR-508-3p |
| DLD | hsa-miR-3077-3p |
| DLD | hsa-miR-4420 |
| DLD | hsa-miR-668 |
| DLD | hsa-miR-625-5p |
| DLD | hsa-miR-3591-3p |
| DLD | hsa-miR-7377-3p |
| DLD | hsa-miR-214-3p |
| DLD | hsa-miR-548j-5p |
| DLD | hsa-miR-4261 |
| DLD | hsa-miR-879-3p |
| DLD | hsa-miR-26-3p |
| DLD | hsa-miR-3607-3p |
| DLD | hsa-miR-331-5p |
| DLD | hsa-miR-2296 |
| DLD | hsa-miR-6750-5p |
| DLD | hsa-miR-411-3p |
| DLD | hsa-miR-424-5p |
| DLD | hsa-miR-7349-5p |
| DLD | hsa-miR-1200 |
| DLD | hsa-miR-2284b |
| DLD | hsa-miR-449c-3p |
| DLD | hsa-miR-4426 |
| DLD | hsa-miR-3912-5p |
| DLD | hsa-miR-4722-3p |
| DLD | hsa-miR-202-5p |
| DLD | hsa-miR-3688-5p |
| DLD | hsa-miR-4641 |
| DLD | hsa-miR-6864-3p |
| DLD | hsa-miR-197-3p |
| DLD | hsa-miR-3104-5p |
| DLD | hsa-miR-942-5p |
| DLD | hsa-miR-373-5p |
| DLD | hsa-miR-219-5p |
| DLD | hsa-miR-2354 |
| DLD | hsa-miR-203-3p |
| DLD | hsa-miR-1910-5p |
| DLD | hsa-miR-4647 |
| DLD | hsa-miR-466bcp-3p |
| DLD | hsa-miR-539-5p |
| DLD | hsa-miR-875-5p |
| DLD | hsa-miR-4302 |
| DLD | hsa-miR-548ae-5p |
| DLD | hsa-miR-6890-5p |
| DLD | hsa-miR-6747-3p |
| DLD | hsa-miR-1273d |
| DLD | hsa-miR-8875 |
| DLD | hsa-miR-181b-2-3p |
| DLD | hsa-miR-371b-5p |
| DLD | hsa-miR-4769-5p |
| DLD | hsa-miR-674-3p |
| DLD | hsa-miR-3714 |
| DLD | hsa-miR-548v |
| DLD | hsa-miR-140-3p |
| DLD | hsa-miR-486-5p |
| DLD | hsa-miR-6506-5p |
| DLD | hsa-miR-16-1-3p |
| DLD | hsa-miR-34b-3p |
| DLD | hsa-miR-8840 |
| DLD | hsa-miR-3583-5p |
| DLD | hsa-miR-10b-5p |
| DLD | hsa-miR-377-5p |
| DLD | hsa-miR-7246-3p |
| DLD | hsa-miR-212-5p |
| DLD | hsa-miR-557 |
| DLD | hsa-miR-302f |
| DLD | hsa-miR-147a |
| DLD | hsa-miR-889-3p |
| DLD | hsa-miR-1262-3p |
| DLD | hsa-miR-4707-3p |
| DLD | hsa-miR-6524 |
| DLD | hsa-miR-6744-5p |
| DLD | hsa-miR-7256-5p |
| DLD | hsa-miR-4795-5p |
| DLD | hsa-miR-135-3p |
| DLD | hsa-miR-873b |
| DLD | hsa-miR-5193 |
| DLD | hsa-miR-1208 |
| DLD | hsa-miR-8083 |
| DLD | hsa-miR-1197-3p |
| DLD | hsa-miR-1803 |
| DLD | hsa-miR-5691 |
| DLD | hsa-miR-2322-5p |
| DLD | hsa-miR-488-5p |
| DLD | hsa-miR-548aef |
| DLD | hsa-miR-331-3p |
| DLD | hsa-miR-7207-3p |
| DLD | hsa-miR-8825 |
| DLD | hsa-miR-222-3p |
| DLD | hsa-miR-1273h-5p |
| DLD | hsa-miR-1322 |
| DLD | hsa-miR-7217-3p |
| DLD | hsa-miR-513a-3p |
| DLD | hsa-miR-513c-5p |
| DLD | hsa-miR-4727-3p |
| DLD | hsa-miR-5703 |
| DLD | hsa-miR-7226-3p |
| DLD | hsa-miR-378d |
| DLD | hsa-miR-15 |
| DLD | hsa-miR-144-5p |
| DLD | hsa-miR-1248 |
| DLD | hsa-miR-548c-3p |
| DLD | hsa-miR-8851 |
| DLD | hsa-miR-195-5p |
| DLD | hsa-miR-7025-3p |
| DLD | hsa-miR-465c-5p |
| DLD | hsa-miR-7156-5p |
| DLD | hsa-let-7a-3p |
| DLD | hsa-miR-506-5p |
| DLD | hsa-miR-571 |
| DLD | hsa-miR-4439 |
| DLD | hsa-miR-8831 |
| DLD | hsa-miR-344-3p |
| DLD | hsa-miR-563 |
| DLD | hsa-miR-4700-3p |
| DLD | hsa-miR-141-3p |
| DLD | hsa-miR-6778-3p |
| DLD | hsa-miR-6500-3p |
| DLD | hsa-miR-181b-3p |
| DLD | hsa-miR-606 |
| DLD | hsa-miR-6959-5p |
| DLD | hsa-miR-96-5p |
| DLD | hsa-miR-4709-5p |
| DLD | hsa-miR-1814b |
| DLD | hsa-miR-499b-5p |
| DLD | hsa-miR-190a-5p |
| DLD | hsa-miR-1228-3p |
| DLD | hsa-miR-1243-3p |
| DLD | hsa-miR-216-3p |
| DLD | hsa-miR-3943 |
| DLD | hsa-miR-4715-3p |
| DLD | hsa-miR-4666a-3p |
| DLD | hsa-miR-4800-5p |
| DLD | hsa-miR-7686-5p |
| DLD | hsa-let-7abf-3p |
| DLD | hsa-miR-215 |
| DLD | hsa-miR-2325b |
| DLD | hsa-miR-4425 |
| DLD | hsa-miR-548a-5p |
| DLD | hsa-miR-6238 |
| DLD | hsa-miR-7273-3p |
| DLD | hsa-miR-302 |
| DLD | hsa-miR-470-5p |
| DLD | hsa-miR-7385c-3p |
| DLD | hsa-miR-383-3p |
| DLD | hsa-miR-876-5p |
| DLD | hsa-miR-222-3p |
| DLD | hsa-miR-562 |
| DLD | hsa-miR-139-3p |
| DLD | hsa-miR-509-3p |
| DLD | hsa-miR-561-3p |
| DLD | hsa-miR-4723-5p |
| DLD | hsa-miR-2464-3p |
| DLD | hsa-miR-7332-3p |
| DLD | hsa-miR-548cej-3p |
| DLD | hsa-miR-3922-3p |
| DLD | hsa-miR-3123 |
| DLD | hsa-miR-3577-3p |
| DLD | hsa-miR-300 |
| DLD | hsa-miR-1468-3p |
| DLD | hsa-miR-34-5p |
| DLD | hsa-miR-2284w |
| DLD | hsa-miR-6801-5p |
| DLD | hsa-miR-676-5p |
| DLD | hsa-miR-2386 |
| DLD | hsa-miR-6536 |
| DLD | hsa-miR-8077 |
| DLD | hsa-miR-454-5p |
| DLD | hsa-miR-3941 |
| DLD | hsa-miR-2284l |
| DLD | hsa-miR-3544-5p |
| DLD | hsa-miR-376-3p |
| DLD | hsa-miR-7347-3p |
| DLD | hsa-miR-193b-3p |
| DLD | hsa-miR-186-3p |
| DLD | hsa-miR-4731-5p |
| DLD | hsa-miR-1254 |
| DLD | hsa-miR-2305 |
| DLD | hsa-miR-628-5p |
| DLD | hsa-miR-7056-5p |
| DLD | hsa-miR-200c-5p |
| DLD | hsa-miR-548ab |
| DLD | hsa-miR-7385e-3p |
| DLD | hsa-miR-7168-3p |
| DLD | hsa-let-7a-2-3p |
| DLD | hsa-miR-335-5p |
| DLD | hsa-miR-8855 |
| DLD | hsa-miR-4484 |
| DLD | hsa-miR-2117 |
| DLD | hsa-miR-432-5p |
| DLD | hsa-miR-7157-3p |
| DLD | hsa-miR-4330 |
| DLD | hsa-miR-7680-5p |
| DLD | hsa-miR-301-3p |
| DLD | hsa-miR-7178-3p |
| DLD | hsa-miR-219-1-3p |
| DLD | hsa-miR-7154-5p |
| DLD | hsa-miR-2303 |
| DLD | hsa-miR-6886-5p |
| DLD | hsa-miR-20-5p |
| DLD | hsa-miR-4516 |
| DLD | hsa-miR-4286 |
| DLD | hsa-miR-124 |
| DLD | hsa-miR-7855-5p |
| DLD | hsa-miR-5687 |
| DLD | hsa-miR-6833-5p |
| DLD | hsa-miR-891-3p |
| DLD | hsa-miR-345-5p |
| DLD | hsa-miR-873-5p |
| DLD | hsa-miR-7166-5p |
| DLD | hsa-miR-548h-5p |
| DLD | hsa-miR-224-5p |
| DLD | hsa-miR-642-3p |
| DLD | hsa-miR-8081 |
| DLD | hsa-miR-6119-3p |
| DLD | hsa-miR-4639-3p |
| DLD | hsa-miR-502b |
| DLD | hsa-miR-8837 |
| DLD | hsa-miR-491-5p |
| DLD | hsa-miR-4775 |
| DLD | hsa-miR-6882-3p |
| DLD | hsa-miR-374a-3p |
| DLD | hsa-miR-2390 |
| DLD | hsa-miR-4475 |
| DLD | hsa-miR-10b-3p |
| DLD | hsa-miR-7388c-5p |
| DLD | hsa-miR-548i |
| DLD | hsa-miR-7315-3p |
| DLD | hsa-miR-6765-5p |
| DLD | hsa-miR-3136-5p |
| DLD | hsa-miR-3664-5p |
| DLD | hsa-miR-4271 |
| DLD | hsa-miR-6812-3p |
| DLD | hsa-miR-4802-3p |
| DLD | hsa-miR-4472 |
| DLD | hsa-miR-425 |
| DLD | hsa-miR-3167-3p |
| DLD | hsa-miR-548aq-3p |
| DLD | hsa-miR-7113-5p |
| DLD | hsa-miR-4778-5p |
| DLD | hsa-miR-6577-3p |
| DLD | hsa-miR-511-3p |
| DLD | hsa-miR-6779-5p |
| DLD | hsa-miR-136-3p |
| DLD | hsa-miR-1185-5p |
| DLD | hsa-miR-6883-3p |
| DLD | hsa-miR-30a-3p |
| DLD | hsa-miR-409-3p |
| DLD | hsa-miR-373-3p |
| DLD | hsa-miR-1936 |
| DLD | hsa-miR-122 |
| DLD | hsa-miR-4803 |
| DLD | hsa-miR-944 |
| DLD | hsa-miR-301 |
| DLD | hsa-miR-7154-3p |
| DLD | hsa-miR-6077 |
| DLD | hsa-miR-34b-3p |
| DLD | hsa-miR-3668 |
| DLD | hsa-miR-7652-5p |
| DLD | hsa-miR-7225-5p |
| DLD | hsa-miR-8808 |
| DLD | hsa-miR-449a-3p |
| DLD | hsa-miR-496b |
| DLD | hsa-miR-7175-5p |
| DLD | hsa-miR-4515 |
| DLD | hsa-miR-548aq-5p |
| DLD | hsa-miR-570-3p |
| DLD | hsa-miR-7845-5p |
| DLD | hsa-miR-299 |
| DLD | hsa-miR-4716 |
| DLD | hsa-miR-93-5p |
| DLD | hsa-miR-4432 |
| DLD | hsa-miR-513 |
| DLD | hsa-miR-520f-3p |
| DLD | hsa-miR-145-5p |
| DLD | hsa-miR-670-5p |
| DLD | hsa-miR-450b-5p |
| DLD | hsa-miR-1198-5p |
| DLD | hsa-miR-34c-3p |
| DLD | hsa-miR-7189-3p |
| DLD | hsa-miR-466i-3p |
| DLD | hsa-miR-7g-3p |
| DLD | hsa-miR-6481 |
| DLD | hsa-miR-4680-3p |
| DLD | hsa-miR-424-5p |
| DLD | hsa-miR-7682-3p |
| DLD | hsa-miR-4446-3p |
| DLD | hsa-miR-617 |
| DLD | hsa-miR-616-3p |
| DLD | hsa-miR-133 |
| DLD | hsa-miR-8820 |
| DLD | hsa-miR-1226-5p |
| DLD | hsa-miR-3940-5p |
| DLD | hsa-miR-4445-5p |
| DLD | hsa-miR-6340 |
| DLD | hsa-miR-411-5p.2 |
| DLD | hsa-miR-18-3p |
| DLD | hsa-miR-6338 |
| DLD | hsa-miR-27a-5p |
| DLD | hsa-miR-7077-5p |
| DLD | hsa-miR-660-3p |
| DLD | hsa-miR-653-3p |
| DLD | hsa-miR-1224-3p |
| DLD | hsa-miR-5584-5p |
| DLD | hsa-miR-520gh |
| DLD | hsa-miR-4756-3p |
| DLD | hsa-miR-6976-5p |
| DLD | hsa-miR-1941-3p |
| DLD | hsa-miR-6764-3p |
| DLD | hsa-miR-3120-3p |
| DLD | hsa-miR-379-5p |
| DLD | hsa-miR-7302-3p |
| DLD | hsa-miR-3570 |
| DLD | hsa-miR-2399-3p |
| DLD | hsa-miR-449b-3p |
| DLD | hsa-miR-16-3p |
| DLD | hsa-miR-6420 |
| DLD | hsa-miR-3127-5p |
| DLD | hsa-miR-519 |
| DLD | hsa-miR-6799-5p |
| DLD | hsa-miR-519d-5p |
| DLD | hsa-miR-7156-3p |
| DLD | hsa-miR-1269a |
| DLD | hsa-miR-1273f |
| DLD | hsa-miR-545-3p |
| DLD | hsa-miR-664a-5p |
| DLD | hsa-miR-1843a-5p |
| DLD | hsa-miR-713 |
| DLD | hsa-miR-4524a-3p |
| DLD | hsa-miR-7272-3p |
| DLD | hsa-miR-3614-5p |
| DLD | hsa-miR-3121-5p |
| DLD | hsa-miR-6504-3p |
| DLD | hsa-miR-769 |
| DLD | hsa-miR-5003-5p |
| DLD | hsa-miR-7313-3p |
| DLD | hsa-miR-3622b-5p |
| DLD | hsa-miR-324 |
| DLD | hsa-miR-8056 |
| DLD | hsa-miR-6843-3p |
| DLD | hsa-miR-468-5p |
| DLD | hsa-miR-15-5p |
| DLD | hsa-miR-8804 |
| DLD | hsa-miR-743ab-3p |
| DLD | hsa-miR-4724-3p |
| DLD | hsa-miR-672-3p |
| DLD | hsa-miR-4695-3p |
| DLD | hsa-miR-470-3p |
| DLD | hsa-miR-3190-3p |
| DLD | hsa-miR-7043-5p |
| DLD | hsa-miR-1960 |
| DLD | hsa-miR-575 |
| DLD | hsa-miR-6516-5p |
| DLD | hsa-miR-186-5p |
| DLD | hsa-miR-5690 |
| DLD | hsa-miR-653 |
| DLD | hsa-miR-7386g-3p |
| DLD | hsa-miR-8813 |
| DLD | hsa-miR-297-5p |
| DLD | hsa-miR-496 |
| DLD | hsa-miR-7974 |
| DLD | hsa-miR-2426 |
| DLD | hsa-miR-669b-5p |
| DLD | hsa-miR-4480 |
| DLD | hsa-miR-516-3p |
| DLD | hsa-miR-548x-5p |
| DLD | hsa-miR-323b-3p |
| DLD | hsa-miR-873-5p |
| DLD | hsa-miR-7060-3p |
| DLD | hsa-miR-4272 |
| DLD | hsa-miR-140-3p.2 |
| DLD | hsa-miR-3129-3p |
| DLD | hsa-miR-192-5p |
| DLD | hsa-miR-4691-3p |
| DLD | hsa-miR-30b-5p |
| DLD | hsa-miR-6730-5p |
| DLD | hsa-miR-676-3p |
| DLD | hsa-miR-4650-5p |
| DLD | hsa-miR-7081-5p |
| DLD | hsa-miR-8107 |
| DLD | hsa-miR-6983-5p |
| DLD | hsa-miR-3475-5p |
| DLD | hsa-miR-3058-3p |
| DLD | hsa-miR-5000-5p |
| DLD | hsa-miR-3578 |
| DLD | hsa-miR-139-5p |
| DLD | hsa-miR-3144-3p |
| DLD | hsa-miR-411-5p |
| DLD | hsa-miR-8066 |
| DLD | hsa-miR-107-3p |
| DLD | hsa-miR-7159-5p |
| DLD | hsa-miR-30e-5p |
| DLD | hsa-miR-4764-3p |
| DLD | hsa-miR-2461-5p |
| DLD | hsa-miR-224-5p |
| DLD | hsa-miR-4781-3p |
| DLD | hsa-miR-8901 |
| DLD | hsa-miR-510-3p |
| DLD | hsa-miR-452-5p |
| DLD | hsa-miR-7321-5p |
| DLD | hsa-miR-3609 |
| DLD | hsa-miR-188-5p |
| DLD | hsa-miR-548av-5p |
| DLD | hsa-miR-20a-3p |
| DLD | hsa-miR-105b |
| DLD | hsa-miR-134-5p |
| DLD | hsa-miR-548ay-3p |
| DLD | hsa-miR-701-3p |
| DLD | hsa-miR-218-5p |
| DLD | hsa-miR-5197-5p |
| DLD | hsa-miR-7386j-3p |
| DLD | hsa-miR-384 |
| DLD | hsa-miR-302bd |
| DLD | hsa-miR-106 |
| DLD | hsa-miR-335-3p |
| DLD | hsa-miR-96-5p |
| DLD | hsa-miR-3153 |
| DLD | hsa-miR-7660-3p |
| DLD | hsa-miR-5089-5p |
| DLD | hsa-miR-1306-5p |
| DLD | hsa-miR-567 |
| DLD | hsa-miR-367-3p |
| DLD | hsa-miR-527 |
| DLD | hsa-miR-8791 |
| DLD | hsa-let-7 |
| DLD | hsa-miR-4742-5p |
| DLD | hsa-miR-383-5p.1 |
| DLD | hsa-miR-665-3p |
| DLD | hsa-miR-208a-3p |
| DLD | hsa-miR-16-5p |
| DLD | hsa-miR-584-3p |
| DLD | hsa-miR-5680 |
| DLD | hsa-miR-133c |
| DLD | hsa-miR-3928-5p |
| DLD | hsa-miR-3078-3p |
| DLD | hsa-miR-515-5p |
| DLD | hsa-miR-216b |
| DLD | hsa-miR-6807-5p |
| DLD | hsa-miR-5089-3p |
| DLD | hsa-miR-2372 |
| DLD | hsa-miR-3087-5p |
| DLD | hsa-miR-548e-5p |
| DLD | hsa-miR-1255b-5p |
| DLD | hsa-miR-5195-5p |
| DLD | hsa-miR-6996-5p |
| DLD | hsa-miR-4457 |
| DLD | hsa-miR-7032-5p |
| DLD | hsa-miR-6750-3p |
| DLD | hsa-miR-6755-3p |
| DLD | hsa-miR-6828-5p |
| DLD | hsa-miR-125a-3p |
| DLD | hsa-miR-4801 |
| DLD | hsa-miR-30--3p |
| DLD | hsa-miR-7681-5p |
| DLD | hsa-miR-8119 |
| DLD | hsa-miR-1253 |
| DLD | hsa-miR-650abc |
| DLD | hsa-miR-337-3p |
| DLD | hsa-miR-7111-5p |
| DLD | hsa-miR-7021-3p |
| DLD | hsa-miR-7-3p |
| DLD | hsa-miR-8883 |
| DLD | hsa-miR-185-5p |
| DLD | hsa-miR-6976-3p |
| DLD | hsa-miR-3646 |
| DLD | hsa-miR-7192-5p |
| DLD | hsa-miR-2285 |
| DLD | hsa-miR-29b-5p |
| DLD | hsa-miR-611 |
| DLD | hsa-miR-30c-3p |
| DLD | hsa-miR-6752-5p |
| DLD | hsa-miR-1662 |
| DLD | hsa-miR-201-5p |
| DLD | hsa-miR-2333 |
| DLD | hsa-miR-5701 |
| DLD | hsa-miR-1263 |
| DLD | hsa-miR-4716-3p |
| DLD | hsa-miR-514b-5p |
| DLD | hsa-miR-20 |
| DLD | hsa-miR-4695-5p |
| DLD | hsa-miR-6785-5p |
| DLD | hsa-miR-559 |
| DLD | hsa-let-7-5p |
| DLD | hsa-miR-460-5p |
| DLD | hsa-miR-1252-3p |
| DLD | hsa-miR-20-3p |
| DLD | hsa-miR-2285af |
| DLD | hsa-miR-548z |
| DLD | hsa-miR-7317-3p |
| DLD | hsa-miR-410 |
| DLD | hsa-miR-2315 |
| DLD | hsa-miR-4666b |
| DLD | hsa-miR-548ap-5p |
| DLD | hsa-miR-6165 |
| DLD | hsa-miR-550a-3p |
| DLD | hsa-miR-380-5p |
| DLD | hsa-miR-3613-5p |
| DLD | hsa-miR-8844 |
| DLD | hsa-miR-423-5p |
| DLD | hsa-miR-3136-3p |
| DLD | hsa-miR-548f-3p |
| DLD | hsa-miR-2402 |
| DLD | hsa-miR-8060 |
| DLD | hsa-miR-3113-3p |
| DLD | hsa-miR-30d-5p |
| DLD | hsa-miR-1582 |
| DLD | hsa-miR-217-3p |
| DLD | hsa-miR-433-3p |
| DLD | hsa-miR-219-3p |
| DLD | hsa-miR-154c |
| DLD | hsa-miR-6795-3p |
| DLD | hsa-miR-3973 |
| DLD | hsa-miR-741-3p |
| DLD | hsa-miR-675-3p |
| DLD | hsa-miR-374-3p |
| DLD | hsa-miR-548at-3p |
| DLD | hsa-miR-490-5p |
| DLD | hsa-miR-3076-3p |
| DLD | hsa-miR-28-3p |
| DLD | hsa-miR-700-5p |
| DLD | hsa-miR-7164-5p |
| DLD | hsa-miR-194-5p |
| DLD | hsa-miR-656 |
| DLD | hsa-miR-3690 |
| DLD | hsa-miR-519-3p |
| DLD | hsa-miR-3099-5p |
| DLD | hsa-miR-8821 |
| DLD | hsa-miR-2449 |
| DLD | hsa-miR-6405 |
| DLD | hsa-miR-6408 |
| DLD | hsa-miR-8485 |
| DLD | hsa-miR-670-3p |
| DLD | hsa-miR-2467-3p |
| DLD | hsa-miR-5571-3p |
| DLD | hsa-miR-217 |
| DLD | hsa-miR-580-3p |
| DLD | hsa-miR-589-3p |
| DLD | hsa-miR-1966-5p |
| DLD | hsa-miR-939-5p |
| DLD | hsa-miR-105-5p |
| DLD | hsa-miR-543 |
| DLD | hsa-miR-6733-5p |
| DLD | hsa-miR-2440 |
| DLD | hsa-miR-1301-5p |
| DLD | hsa-miR-7192-3p |
| DLD | hsa-miR-3200-5p |
| DLD | hsa-miR-122-5p |
| DLD | hsa-miR-3658 |
| DLD | hsa-miR-7078-3p |
| DLD | hsa-miR-1839 |
| DLD | hsa-miR-6631-5p |
| DLD | hsa-miR-8888 |
| DLD | hsa-miR-3689-3p |
| DLD | hsa-miR-373-3p |
| DLD | hsa-miR-361-5p |
| DLD | hsa-miR-2483-5p |
| DLD | hsa-miR-741-5p |
| DLD | hsa-miR-338-3p |
| DLD | hsa-miR-603 |
| DLD | hsa-miR-6508-5p |
| DLD | hsa-miR-1287-3p |
| DLD | hsa-miR-5128 |
| DLD | hsa-miR-662 |
| DLD | hsa-miR-471-3p |
| DLD | hsa-miR-7675-3p |
| DLD | hsa-miR-2361 |
| DLD | hsa-miR-7106-5p |
| DLD | hsa-miR-101-3p |
| DLD | hsa-miR-4277 |
| DLD | hsa-miR-6830-5p |
| DLD | hsa-miR-4685-5p |
| DLD | hsa-miR-495-3p |
| DLD | hsa-miR-802-5p |
| DLD | hsa-miR-134-3p |
| DLD | hsa-miR-376a-3p |
| DLD | hsa-miR-7161-5p |
| DLD | hsa-miR-450a-1-3p |
| DLD | hsa-miR-548ao-3p |
| DLD | hsa-miR-607 |
| DLD | hsa-miR-2422 |
| DLD | hsa-miR-4311 |
| DLD | hsa-miR-548am-5p |
| DLD | hsa-miR-3157-5p |
| DLD | hsa-miR-221-3p |
| DLD | hsa-miR-4653-3p |
| DLD | hsa-miR-8879 |
| DLD | hsa-miR-3678-3p |
| DLD | hsa-miR-2284m |
| DLD | hsa-miR-605-5p |
| DLD | hsa-miR-376ab |
| DLD | hsa-miR-23-5p |
| DLD | hsa-miR-7198-3p |
| DLD | hsa-miR-6794-5p |
| DLD | hsa-miR-4773 |
| DLD | hsa-miR-548w |
| DLD | hsa-miR-6363 |
| DLD | hsa-miR-599-3p |
| DLD | hsa-miR-2285aa |
| DLD | hsa-miR-450b |
| DLD | hsa-miR-589-5p |
| DLD | hsa-miR-7172-3p |
| DLD | hsa-miR-361-3p |
| DLD | hsa-miR-3117-3p |
| DLD | hsa-miR-4694-5p |
| DLD | hsa-miR-1298 |
| DLD | hsa-miR-704 |
| DLD | hsa-miR-548d-5p |
| DLD | hsa-miR-6985-5p |
| DLD | hsa-miR-377-3p |
| DLD | hsa-miR-4291 |
| DLD | hsa-miR-1281 |
| DLD | hsa-miR-1244 |
| DLD | hsa-miR-466l-3p |
| DLD | hsa-miR-382-3p |
| DLD | hsa-miR-9-3p |
| DLD | hsa-miR-2393 |
| DLD | hsa-miR-2054 |
| DLD | hsa-miR-3134 |
| DLD | hsa-miR-6529a |
| DLD | hsa-miR-514a-5p |
| DLD | hsa-miR-519e-5p |
| DLD | hsa-miR-8850 |
| DLD | hsa-miR-15b-3p |
| DLD | hsa-miR-1180-5p |
| DLD | hsa-miR-340-5p |
| DLD | hsa-miR-4447 |
| DLD | hsa-miR-8054 |
| DLD | hsa-miR-6119-5p |
| DLD | hsa-miR-3164 |
| DLD | hsa-miR-6826-3p |
| DLD | hsa-miR-5197-3p |
| DLD | hsa-miR-1249-5p |
| DLD | hsa-miR-4529-5p |
| DLD | hsa-miR-3088-3p |
| DLD | hsa-miR-7234-5p |
| DLD | hsa-miR-2285g |
| DLD | hsa-miR-6359 |
| DLD | hsa-miR-616-5p |
| DLD | hsa-miR-151-3p |
| DLD | hsa-miR-4707-5p |
| DLD | hsa-miR-568 |
| DLD | hsa-miR-6401 |
| DLD | hsa-miR-1290 |
| DLD | hsa-miR-4731-3p |
| DLD | hsa-miR-361-5p |
| DLD | hsa-miR-7059-3p |
| DLD | hsa-miR-5620-3p |
| DLD | hsa-miR-6873-5p |
| DLD | hsa-miR-3074-3p |
| DLD | hsa-miR-8887 |
| DLD | hsa-miR-1240 |
| DLD | hsa-miR-553 |
| DLD | hsa-miR-30ad-3p |
| DLD | hsa-miR-4743-3p |
| DLD | hsa-miR-514a-3p |
| DLD | hsa-miR-7175-3p |
| DLD | hsa-miR-6515-3p |
| DLD | hsa-miR-7-5p |
| DLD | hsa-miR-4273 |
| DLD | hsa-miR-641 |
| DLD | hsa-miR-6974-3p |
| DLD | hsa-miR-4530 |
| DLD | hsa-miR-4448 |
| DLD | hsa-miR-502-5p |
| DLD | hsa-miR-3589 |
| DLD | hsa-miR-644a |
| DLD | hsa-miR-126-3p |
| DLD | hsa-miR-4433b-3p |
| DLD | hsa-miR-1261 |
| DLD | hsa-miR-344h-3p |
| DLD | hsa-miR-6511a-5p |
| DLD | hsa-miR-2341 |
| DLD | hsa-miR-3069-3p |
| DLD | hsa-miR-4771 |
| DLD | hsa-miR-677-3p |
| DLD | hsa-miR-6846-3p |
| DLD | hsa-miR-3109-3p |
| DLD | hsa-miR-548am-3p |
| DLD | hsa-miR-6788-5p |
| DLD | hsa-miR-142-3p.2 |
| DLD | hsa-miR-7681-3p |
| DLD | hsa-let-7c-1-3p |
| DLD | hsa-miR-486-3p |
| DLD | hsa-miR-651-3p |
| DLD | hsa-miR-7398m-3p |
| DLD | hsa-miR-3147 |
| DLD | hsa-miR-654-5p |
| DLD | hsa-miR-708-3p |
| DLD | hsa-miR-3613-3p |
| DLD | hsa-miR-1296-3p |
| DLD | hsa-miR-218-1-3p |
| DLD | hsa-miR-6987-3p |
| DLD | hsa-miR-548u |
| DLD | hsa-miR-324-3p |
| DLD | hsa-miR-384-5p |
| DLD | hsa-miR-6715-3p |
| DLD | hsa-miR-323b-5p |
| DLD | hsa-miR-5589-5p |
| DLD | hsa-miR-920 |
| DLD | hsa-miR-1288-3p |
| DLD | hsa-miR-7155-3p |
| DLD | hsa-miR-6379 |
| DLD | hsa-miR-7333-5p |
| DLD | hsa-miR-3942-3p |
| DLD | hsa-miR-6973b-5p |
| DLD | hsa-miR-7323-3p |
| DLD | hsa-miR-6913-3p |
| DLD | hsa-miR-6757-3p |
| DLD | hsa-miR-651 |
| DLD | hsa-miR-136-5p |
| DLD | hsa-miR-4662b |
| DLD | hsa-miR-302c-3p |
| DLD | hsa-miR-522-3p |
| DLD | hsa-miR-302c-3p.2 |
| DLD | hsa-miR-488-3p |
| DLD | hsa-miR-890 |
| DLD | hsa-miR-4720-3p |
| DLD | hsa-miR-7034-3p |
| DLD | hsa-miR-6086 |
| DLD | hsa-miR-4654 |
| DLD | hsa-miR-155-5p |
| DLD | hsa-miR-4789-3p |
| DLD | hsa-miR-6883-5p |
| DLD | hsa-miR-6806-3p |
| DLD | hsa-miR-4663 |
| DLD | hsa-miR-4685-3p |
| DLD | hsa-miR-4724-5p |
| DLD | hsa-miR-7385b-3p |
| DLD | hsa-miR-7111-3p |
| DLD | hsa-miR-7158-5p |
| DLD | hsa-miR-3065-3p |
| DLD | hsa-miR-2115-3p |
| DLD | hsa-miR-495-5p |
| DLD | hsa-miR-697 |
| DLD | hsa-miR-6881-3p |
| DLD | hsa-miR-216-5p |
| DLD | hsa-miR-618 |
| DLD | hsa-miR-5692a |
| DLD | hsa-miR-6870-5p |
| DLD | hsa-miR-6639-5p |
| DLD | hsa-miR-1266-3p |
| DLD | hsa-miR-875-3p |
| DLD | hsa-miR-4445-3p |
| DLD | hsa-miR-499 |
| DLD | hsa-miR-4791 |
| DLD | hsa-miR-2285e |
| DLD | hsa-miR-5187-3p |
| DLD | hsa-miR-548t-3p |
| DLD | hsa-miR-6368 |
| DLD | hsa-miR-3919 |
| DLD | hsa-miR-4251 |
| DLD | hsa-miR-6932-3p |
| DLD | hsa-miR-429-3p |
| DLD | hsa-miR-8094 |
| DLD | hsa-miR-3559-5p |
| DLD | hsa-miR-4793-3p |
| DLD | hsa-miR-3965 |
| DLD | hsa-miR-1814 |
| DLD | hsa-miR-1202 |
| DLD | hsa-miR-8087 |
| DLD | hsa-miR-7200-3p |
| DLD | hsa-miR-548k |
| DLD | hsa-miR-764 |
| DLD | hsa-miR-302-3p |
| DLD | hsa-miR-410-3p |
| DLD | hsa-miR-27b-5p |
| DLD | hsa-miR-186-5p |
| DLD | hsa-miR-6765-3p |
| DLD | hsa-miR-5008-3p |
| DLD | hsa-miR-211-5p |
| DLD | hsa-miR-27-3p |
| DLD | hsa-miR-577-5p |
| DLD | hsa-miR-7374a-3p |
| DLD | hsa-miR-2284a |
| DLD | hsa-miR-6505-5p |
| DLD | hsa-miR-6878-5p |
| DLD | hsa-miR-551-5p |
| DLD | hsa-miR-141-3p |
| DLD | hsa-miR-4517 |
| DLD | hsa-miR-7180-5p |
| DLD | hsa-miR-411-5p |
| DLD | hsa-miR-1271-5p |
| DLD | hsa-miR-372-5p |
| DLD | hsa-miR-4428 |
| DLD | hsa-miR-942-3p |
| DLD | hsa-miR-6783-5p |
| DLD | hsa-miR-2439-3p |
| DLD | hsa-miR-4665-5p |
| DLD | hsa-miR-8067 |
| DLD | hsa-miR-7676-5p |
| DLD | hsa-miR-302a-5p |
| DLD | hsa-miR-6951-5p |
| DLD | hsa-miR-548d-3p |
| DLD | hsa-miR-4470 |
| DLD | hsa-miR-3176 |
| DLD | hsa-miR-124-5p |
| DLD | hsa-miR-6842-5p |
| DLD | hsa-miR-6897-5p |
| DLD | hsa-miR-548 |
| DLD | hsa-miR-99-3p |
| DLD | hsa-miR-548f-5p |
| DLD | hsa-miR-6404 |
| DLD | hsa-miR-1199-5p |
| DLD | hsa-miR-7398t-3p |
| DLD | hsa-miR-6780b-5p |
| DLD | hsa-miR-488-3p |
| DLD | hsa-miR-7222-3p |
| DLD | hsa-miR-452-5p |
| DLD | hsa-miR-3117-3p |
| DLD | hsa-miR-548p |
| DLD | hsa-miR-206 |
| DLD | hsa-miR-2345 |
| DLD | hsa-miR-193b-5p |
| DLD | hsa-miR-29-5p |
| DLD | hsa-miR-2319 |
| DLD | hsa-miR-421-3p |
| DLD | hsa-miR-7011-5p |
| DLD | hsa-miR-200a-3p |
| DLD | hsa-miR-548as-3p |
| DLD | hsa-miR-23b-3p |
| DLD | hsa-miR-7237-3p |
| DLD | hsa-miR-512 |
| DLD | hsa-miR-195-5p |
| DLD | hsa-miR-151a-5p |
| DLD | hsa-miR-519e-3p |
| DLD | hsa-miR-671-5p |
| DLD | hsa-miR-892b |
| DLD | hsa-miR-2183 |
| DLD | hsa-miR-4501 |
| DLD | hsa-miR-548b-5p |
| DLD | hsa-miR-4257 |
| DLD | hsa-miR-29a-1-5p |
| DLD | hsa-miR-4733-5p |
| DLD | hsa-miR-142-5p |
| DLD | hsa-miR-7687-3p |
| DLD | hsa-miR-2355-5p |
| DLD | hsa-miR-6770-5p |
| DLD | hsa-miR-374c-3p |
| DLD | hsa-miR-1a-5p |
| DLD | hsa-miR-877-5p |
| DLD | hsa-miR-5006-3p |
| DLD | hsa-miR-98-3p |
| DLD | hsa-miR-532-3p |
| DLD | hsa-miR-1239 |
| DLD | hsa-miR-150-5p |
| DLD | hsa-miR-101b-5p |
| DLD | hsa-miR-1250-3p |
| DLD | hsa-miR-337-3p |
| DLD | hsa-miR-6836-3p |
| DLD | hsa-miR-6896-3p |
| DLD | hsa-miR-7064-3p |
| DLD | hsa-miR-592-5p |
| DLD | hsa-miR-666-3p |
| DLD | hsa-miR-7286-3p |
| DLD | hsa-miR-145-5p |
| DLD | hsa-miR-8070 |
| DLD | hsa-let-7b-3p |
| DLD | hsa-miR-338-3p |
| DLD | hsa-miR-6123 |
| DLD | hsa-miR-16-2-3p |
| DLD | hsa-miR-4789-5p |
| DLD | hsa-miR-4714-5p |
| DLD | hsa-miR-1304-5p |
| DLD | hsa-miR-6529-5p |
| DLD | hsa-miR-374b-3p |
| DLD | hsa-miR-152-3p |
| DLD | hsa-miR-3100-5p |
| DLD | hsa-miR-137-3p |
| DLD | hsa-miR-3145-3p |
| DLD | hsa-miR-32-3p |
| DLD | hsa-miR-6693-3p |
| DLD | hsa-miR-296-3p |
| DLD | hsa-miR-454-3p |
| DLD | hsa-miR-6525 |
| DLD | hsa-miR-138-3p |
| DLD | hsa-miR-4735-5p |
| DLD | hsa-miR-1255a |
| DLD | hsa-miR-8085 |
| DLD | hsa-miR-205-3p |
| DLD | hsa-miR-4539 |
| DLD | hsa-miR-6758-3p |
| DLD | hsa-miR-548j-3p |
| DLD | hsa-miR-6884-3p |
| DLD | hsa-miR-1276 |
| DLD | hsa-miR-7191-5p |
| DLD | hsa-miR-6772-3p |
| DLD | hsa-miR-7854-3p |
| DLD | hsa-miR-26b-3p |
| DLD | hsa-miR-4427 |
| DLD | hsa-miR-7119-3p |
| DLD | hsa-miR-6360 |
| DLD | hsa-miR-922 |
| DLD | hsa-miR-4676-5p |
| DLD | hsa-miR-7198-5p |
| DLD | hsa-miR-501-5p |
| DLD | hsa-miR-6512-5p |
| DLD | hsa-miR-1193-5p |
| DLD | hsa-miR-548aa |
| DLD | hsa-miR-6776-3p |
| DLD | hsa-miR-7053-3p |
| DLD | hsa-miR-6859-3p |
| DLD | hsa-miR-1678 |
| DLD | hsa-miR-4531 |
| DLD | hsa-miR-211-5p |
| DLD | hsa-miR-4434 |
| DLD | hsa-miR-2416 |
| DLD | hsa-miR-6892-5p |
| DLD | hsa-miR-433 |
| DLD | hsa-miR-29a-3p |
| DLD | hsa-miR-125-5p |
| DLD | hsa-miR-369-3p |
| DLD | hsa-miR-518-5p |
| DLD | hsa-miR-1178-5p |
| DLD | hsa-miR-7201-3p |
| DLD | hsa-miR-3121-3p |
| DLD | hsa-miR-6837-3p |
| DLD | hsa-miR-8904b |
| DLD | hsa-miR-2284g |
| DLD | hsa-miR-3156-5p |
| DLD | hsa-miR-130 |
| DLD | hsa-miR-1229-5p |
| DLD | hsa-miR-4509 |
| DLD | hsa-miR-6917-3p |
| DLD | hsa-miR-6861-5p |
| DLD | hsa-miR-873-3p |
| DLD | hsa-miR-370-3p |
| DLD | hsa-miR-496.1 |
| DLD | hsa-miR-2278 |
| DLD | hsa-miR-4459 |
| DLD | hsa-miR-3942-5p |
| DLD | hsa-miR-5196-3p |
| DLD | hsa-miR-328 |
| DLD | hsa-miR-1343-3p |
| DLD | hsa-miR-494 |
| DLD | hsa-miR-6372 |
| DLD | hsa-miR-1265 |
| DLD | hsa-miR-205-5p |
| DLD | hsa-miR-216 |
| DLD | hsa-miR-3925-5p |
| DLD | hsa-miR-497-5p |
| DLD | hsa-miR-196a-3p |
| DLD | hsa-miR-6857-3p |
| DLD | hsa-miR-766-3p |
| DLD | hsa-miR-6499-3p |
| DLD | hsa-miR-6778-5p |
| DLD | hsa-miR-1237-3p |
| DLD | hsa-miR-330-3p.2 |
| DLD | hsa-miR-6760-3p |
| DLD | hsa-miR-6997-3p |
| DLD | hsa-miR-30-5p |
| DLD | hsa-miR-30b-3p |
| DLD | hsa-miR-4677-3p |
| DLD | hsa-miR-381-3p |
| DLD | hsa-miR-135b-3p |
| DLD | hsa-miR-7012-5p |
| DLD | hsa-miR-585-5p |
| DLD | hsa-let-7g-3p |
| DLD | hsa-miR-655 |
| DLD | hsa-miR-2421 |
| DLD | hsa-miR-126-5p |
| DLD | hsa-miR-33-3p |
| DLD | hsa-miR-1537-5p |
| DLD | hsa-miR-342-3p |
| DLD | hsa-miR-466b-3p |
| DLD | hsa-miR-370 |
| DLD | hsa-miR-892-3p |
| DLD | hsa-miR-3558-3p |
| DLD | hsa-miR-3159 |
| DLD | hsa-miR-6794-3p |
| DLD | hsa-miR-574-5p |
| DLD | hsa-miR-144-3p |
| DLD | hsa-miR-577 |
| DLD | hsa-miR-2406 |
| DLD | hsa-miR-26-5p |
| DLD | hsa-miR-5010-5p |
| DLD | hsa-miR-320e |
| DLD | hsa-miR-6501-3p |
| DLD | hsa-miR-548ay-5p |
| DLD | hsa-miR-548h-3p |
| DLD | hsa-miR-181 |
| DLD | hsa-miR-3911 |
| DLD | hsa-miR-3662 |
| DLD | hsa-miR-30-3p |
| DLD | hsa-miR-7057-5p |
| DLD | hsa-miR-548ae-3p |
| DLD | hsa-miR-335-5p |
| DLD | hsa-miR-451b |
| DLD | hsa-miR-1243 |
| DLD | hsa-miR-7975 |
| DLD | hsa-miR-6970-3p |
| DLD | hsa-miR-7350-5p |
| DLD | hsa-miR-4461 |
| DLD | hsa-miR-548aw |
| DLD | hsa-miR-6738-3p |
| DLD | hsa-miR-3173-3p |
| DLD | hsa-miR-7194-5p |
| DLD | hsa-miR-5003-3p |
| DLD | hsa-miR-548az-5p |
| DLD | hsa-miR-98-5p |
| DLD | hsa-miR-580 |
| DLD | hsa-miR-7386c-5p |
| DLD | hsa-miR-549-3p |
| DLD | hsa-miR-136-5p |
| DLD | hsa-miR-544a |
| DLD | hsa-miR-592 |
| DLD | hsa-miR-8886 |
| DLD | hsa-miR-190-5p |
| DLD | hsa-miR-3972 |
| DLD | hsa-miR-7200-5p |
| DLD | hsa-miR-1434-3p |
| DLD | hsa-miR-9500 |
| DLD | hsa-miR-365-3p |
| DLD | hsa-miR-3127-5p |
| DLD | hsa-miR-21-3p |
| DLD | hsa-miR-1303 |
| DLD | hsa-miR-7179-3p |
| DLD | hsa-miR-103-3p |
| DLD | hsa-miR-290b-5p |
| DLD | hsa-miR-1913 |
| DLD | hsa-miR-669c-3p |
| DLD | hsa-miR-379-3p |
| DLD | hsa-miR-6832-3p |
| DLD | hsa-miR-5088-3p |
| DLD | hsa-miR-3062-5p |
| DLD | hsa-miR-3135b |
| DLD | hsa-miR-7398-3p |
| DLD | hsa-miR-599 |
| DLD | hsa-miR-8055 |
| DLD | hsa-miR-6134 |
| DLD | hsa-miR-7035-5p |
| DLD | hsa-miR-6972-3p |
| DLD | hsa-miR-2360 |
| DLD | hsa-miR-5624-5p |
| DLD | hsa-miR-125b-2-3p |
| DLD | hsa-miR-3167-5p |
| DLD | hsa-miR-4495 |
| DLD | hsa-miR-7392-3p |
| DLD | hsa-miR-4284 |
| DLD | hsa-miR-548au-5p |
| DLD | hsa-miR-636 |
| DLD | hsa-miR-2311 |
| DLD | hsa-miR-593-3p |
| DLD | hsa-miR-758-5p |
| DLD | hsa-miR-8806 |
| DLD | hsa-miR-93-5p |
| DLD | hsa-miR-2367-3p |
| DLD | hsa-miR-6855-5p |
| DLST | hsa-miR-3090-3p |
| DLST | hsa-miR-892b |
| DLST | hsa-miR-452-5p |
| DLST | hsa-miR-322-5p |
| DLST | hsa-miR-4635 |
| DLST | hsa-miR-516a-3p |
| DLST | hsa-miR-4668-5p |
| DLST | hsa-miR-6856-5p |
| DLST | hsa-miR-879-5p |
| DLST | hsa-miR-185-5p |
| DLST | hsa-miR-2347 |
| DLST | hsa-miR-140-5p |
| DLST | hsa-miR-2443 |
| DLST | hsa-miR-5582-5p |
| DLST | hsa-miR-342-3p |
| DLST | hsa-miR-15b-5p |
| DLST | hsa-miR-549a |
| DLST | hsa-miR-8796 |
| DLST | hsa-miR-4483 |
| DLST | hsa-miR-4650-3p |
| DLST | hsa-miR-133-5p |
| DLST | hsa-miR-29e |
| DLST | hsa-miR-375 |
| DLST | hsa-miR-221-3p |
| DLST | hsa-miR-1962 |
| DLST | hsa-miR-1238-3p |
| DLST | hsa-miR-7024-3p |
| DLST | hsa-miR-6778-3p |
| DLST | hsa-let-7e-5p |
| DLST | hsa-miR-206-5p |
| DLST | hsa-miR-1193 |
| DLST | hsa-miR-545-3p |
| DLST | hsa-miR-1249-3p |
| DLST | hsa-miR-29b-5p |
| DLST | hsa-miR-4667-3p |
| DLST | hsa-miR-1839 |
| DLST | hsa-miR-2459 |
| DLST | hsa-miR-3200-5p |
| DLST | hsa-miR-3619-5p |
| DLST | hsa-miR-327 |
| DLST | hsa-miR-2889 |
| DLST | hsa-miR-7843-5p |
| DLST | hsa-miR-7216-3p |
| DLST | hsa-miR-6792-3p |
| DLST | hsa-miR-6841-3p |
| DLST | hsa-miR-6737-3p |
| DLST | hsa-miR-150-5p |
| DLST | hsa-miR-7001-3p |
| DLST | hsa-miR-7299-5p |
| DLST | hsa-miR-7080-5p |
| DLST | hsa-miR-5681a |
| DLST | hsa-miR-6516-5p |
| DLST | hsa-miR-6845-3p |
| DLST | hsa-miR-195-5p |
| DLST | hsa-miR-362-3p |
| DLST | hsa-miR-4287 |
| DLST | hsa-miR-16-5p |
| DLST | hsa-miR-3193 |
| DLST | hsa-miR-376c-3p |
| DLST | hsa-miR-6072 |
| DLST | hsa-miR-607 |
| DLST | hsa-miR-874-5p |
| DLST | hsa-miR-7249-3p |
| DLST | hsa-miR-760-5p |
| DLST | hsa-miR-1248 |
| DLST | hsa-miR-6822-5p |
| DLST | hsa-miR-548ar-3p |
| DLST | hsa-miR-7008-3p |
| DLST | hsa-miR-3470b |
| DLST | hsa-miR-6992-3p |
| DLST | hsa-miR-7066-5p |
| DLST | hsa-miR-103-3p |
| DLST | hsa-miR-330-3p |
| DLST | hsa-miR-934-3p |
| DLST | hsa-miR-6334 |
| DLST | hsa-miR-4674 |
| DLST | hsa-miR-6974-5p |
| DLST | hsa-miR-1188-3p |
| DLST | hsa-miR-6769b-3p |
| DLST | hsa-miR-6129 |
| DLST | hsa-miR-6817-3p |
| DLST | hsa-miR-224-3p |
| DLST | hsa-miR-3682-3p |
| DLST | hsa-miR-4731-3p |
| DLST | hsa-miR-518d-5p |
| DLST | hsa-miR-4524a-5p |
| DLST | hsa-miR-1943-5p |
| DLST | hsa-miR-1301-5p |
| DLST | hsa-miR-383-5p |
| DLST | hsa-miR-7385-5p |
| DLST | hsa-miR-1842 |
| DLST | hsa-miR-887-5p |
| DLST | hsa-miR-7047-3p |
| DLST | hsa-miR-873-3p |
| DLST | hsa-miR-1273g-3p |
| DLST | hsa-miR-661 |
| DLST | hsa-miR-2890 |
| DLST | hsa-miR-5197-3p |
| DLST | hsa-miR-3958 |
| DLST | hsa-miR-5046 |
| DLST | hsa-miR-500b-3p |
| DLST | hsa-miR-8812 |
| DLST | hsa-miR-3678-3p |
| DLST | hsa-miR-497-5p |
| DLST | hsa-miR-3689-3p |
| DLST | hsa-miR-6949-3p |
| DLST | hsa-miR-2426 |
| DLST | hsa-miR-361-3p |
| DLST | hsa-miR-133cd-5p |
| DLST | hsa-miR-4797-5p |
| DLST | hsa-miR-6734-5p |
| DLST | hsa-miR-7183-3p |
| DLST | hsa-miR-143-3p |
| DLST | hsa-miR-98-5p |
| DLST | hsa-miR-15a-3p |
| DLST | hsa-miR-3094-3p |
| DLST | hsa-miR-6131 |
| DLST | hsa-miR-133a-5p |
| DLST | hsa-miR-7250-3p |
| DLST | hsa-miR-874 |
| DLST | hsa-miR-4269 |
| DLST | hsa-miR-294 |
| DLST | hsa-miR-764 |
| DLST | hsa-miR-7343-3p |
| DLST | hsa-miR-185-5p |
| DLST | hsa-miR-4445-5p |
| DLST | hsa-miR-429 |
| DLST | hsa-miR-7159-3p |
| DLST | hsa-miR-323b-5p |
| DLST | hsa-miR-7678-5p |
| DLST | hsa-miR-455-5p |
| DLST | hsa-miR-627-3p |
| DLST | hsa-miR-2337 |
| DLST | hsa-miR-7297-5p |
| DLST | hsa-miR-1295b-5p |
| DLST | hsa-miR-6134 |
| DLST | hsa-miR-2355-5p |
| DLST | hsa-miR-331-3p |
| DLST | hsa-miR-5107-3p |
| DLST | hsa-miR-455-3p |
| DLST | hsa-miR-324 |
| DLST | hsa-miR-6870-3p |
| DLST | hsa-miR-2477 |
| DLST | hsa-miR-5104 |
| DLST | hsa-miR-6891-3p |
| DLST | hsa-miR-1224-3p |
| DLST | hsa-miR-873a-5p.1 |
| DLST | hsa-miR-2373-3p |
| DLST | hsa-miR-550 |
| DLST | hsa-miR-506-3p |
| DLST | hsa-miR-7281-3p |
| DLST | hsa-miR-212-3p |
| DLST | hsa-miR-8800 |
| DLST | hsa-miR-219-5p |
| DLST | hsa-miR-4763-3p |
| DLST | hsa-miR-3690 |
| DLST | hsa-miR-7354-3p |
| DLST | hsa-miR-7163-3p |
| DLST | hsa-miR-590-3p |
| DLST | hsa-miR-200bc-3p |
| DLST | hsa-miR-466bco-5p |
| DLST | hsa-miR-7191-3p |
| DLST | hsa-miR-320-3p |
| DLST | hsa-miR-6882-5p |
| DLST | hsa-miR-7383-3p |
| DLST | hsa-miR-632 |
| DLST | hsa-miR-452-3p |
| DLST | hsa-miR-411-5p.2 |
| DLST | hsa-miR-130b-5p |
| DLST | hsa-miR-6859-5p |
| DLST | hsa-miR-6418-5p |
| DLST | hsa-miR-2368-5p |
| DLST | hsa-miR-1915-5p |
| DLST | hsa-miR-4434 |
| DLST | hsa-miR-580-3p |
| DLST | hsa-miR-1191b-5p |
| DLST | hsa-miR-6904-3p |
| DLST | hsa-miR-548e-3p |
| DLST | hsa-miR-7220-3p |
| DLST | hsa-miR-1468-5p |
| DLST | hsa-miR-1273h-3p |
| DLST | hsa-miR-7110-3p |
| DLST | hsa-miR-7306-5p |
| DLST | hsa-miR-136-5p |
| DLST | hsa-miR-18-5p |
| DLST | hsa-miR-7035-5p |
| DLST | hsa-miR-6790-3p |
| DLST | hsa-miR-6873-3p |
| DLST | hsa-miR-519-5p |
| DLST | hsa-miR-1941-3p |
| DLST | hsa-miR-4279 |
| DLST | hsa-miR-143-3p |
| DLST | hsa-miR-139-3p |
| DLST | hsa-miR-6089 |
| DLST | hsa-miR-3922-3p |
| DLST | hsa-miR-7226-5p |
| DLST | hsa-miR-760 |
| DLST | hsa-miR-362-5p |
| DLST | hsa-miR-148-3p |
| DLST | hsa-miR-873b |
| DLST | hsa-miR-2431-3p |
| DLST | hsa-miR-2420 |
| DLST | hsa-miR-6751-3p |
| DLST | hsa-miR-132-3p |
| DLST | hsa-miR-128-3p |
| DLST | hsa-miR-2467-3p |
| DLST | hsa-miR-7289-5p |
| DLST | hsa-miR-6873-5p |
| DLST | hsa-miR-877-5p |
| DLST | hsa-miR-6971-3p |
| DLST | hsa-miR-3105-3p |
| DLST | hsa-miR-1226-5p |
| DLST | hsa-miR-7285-3p |
| DLST | hsa-miR-7263-5p |
| DLST | hsa-miR-526 |
| DLST | hsa-miR-190b-3p |
| DLST | hsa-miR-2360 |
| DLST | hsa-miR-222-3p |
| DLST | hsa-miR-7683-5p |
| DLST | hsa-miR-6775-3p |
| DLST | hsa-miR-1253 |
| DLST | hsa-miR-4281 |
| DLST | hsa-miR-8888 |
| DLST | hsa-miR-4538 |
| DLST | hsa-miR-4728-5p |
| DLST | hsa-miR-2285d |
| DLST | hsa-miR-6865-3p |
| DLST | hsa-miR-5695 |
| DLST | hsa-miR-6969-5p |
| DLST | hsa-miR-7193-5p |
| DLST | hsa-miR-584-3p |
| DLST | hsa-miR-743b-3p |
| DLST | hsa-miR-5112 |
| DLST | hsa-miR-1298-5p |
| DLST | hsa-miR-7062-5p |
| DLST | hsa-miR-467b-5p |
| DLST | hsa-miR-16-5p |
| DLST | hsa-miR-2313-3p |
| DLST | hsa-miR-6333 |
| DLST | hsa-miR-186-5p |
| DLST | hsa-miR-574-5p |
| DLST | hsa-miR-6785-5p |
| DLST | hsa-miR-6794-3p |
| DLST | hsa-miR-1343-3p |
| DLST | hsa-miR-146b-3p |
| DLST | hsa-miR-409-3p |
| DLST | hsa-miR-6896-5p |
| DLST | hsa-miR-6762-3p |
| DLST | hsa-miR-7375-5p |
| DLST | hsa-miR-1226-3p |
| DLST | hsa-miR-767 |
| DLST | hsa-miR-1839-5p |
| DLST | hsa-miR-5619-3p |
| DLST | hsa-miR-5685 |
| DLST | hsa-miR-182-3p |
| DLST | hsa-miR-455-3p.1 |
| DLST | hsa-miR-708-5p |
| DLST | hsa-miR-7652-5p |
| DLST | hsa-miR-190-5p |
| DLST | hsa-miR-6355 |
| DLST | hsa-miR-550-5p |
| DLST | hsa-miR-1256 |
| DLST | hsa-miR-292-3p |
| DLST | hsa-miR-6077 |
| DLST | hsa-miR-6856-3p |
| DLST | hsa-miR-7021-3p |
| DLST | hsa-miR-22-3p |
| DLST | hsa-miR-329-3p |
| DLST | hsa-miR-7314-3p |
| DLST | hsa-miR-874-3p |
| DLST | hsa-miR-7015-3p |
| DLST | hsa-miR-3679-3p |
| DLST | hsa-miR-562 |
| DLST | hsa-miR-6731-5p |
| DLST | hsa-miR-8550 |
| DLST | hsa-miR-3081-5p |
| DLST | hsa-miR-27b-5p |
| DLST | hsa-miR-873a-5p.2 |
| DLST | hsa-miR-700-5p |
| DLST | hsa-miR-6890-3p |
| DLST | hsa-miR-471-5p |
| DLST | hsa-miR-3921 |
| DLST | hsa-miR-666-3p |
| DLST | hsa-miR-4709-3p |
| DLST | hsa-miR-133-3p |
| DLST | hsa-miR-6945-5p |
| DLST | hsa-miR-937-5p |
| DLST | hsa-miR-450a |
| DLST | hsa-miR-1343 |
| DLST | hsa-miR-4517 |
| DLST | hsa-miR-6847-5p |
| DLST | hsa-miR-7221-5p |
| DLST | hsa-miR-29b-2-5p |
| DLST | hsa-miR-7337-5p |
| DLST | hsa-miR-2411-5p |
| DLST | hsa-miR-218-5p |
| DLST | hsa-miR-380-5p |
| DLST | hsa-miR-141-5p |
| DLST | hsa-miR-24-3p |
| DLST | hsa-miR-24-3p |
| DLST | hsa-miR-9768-5p |
| DLST | hsa-miR-4639-3p |
| DLST | hsa-let-7 |
| DLST | hsa-miR-1249-3p |
| DLST | hsa-miR-6511-5p |
| DLST | hsa-miR-7279-3p |
| DLST | hsa-miR-6130 |
| DLST | hsa-miR-7388c-5p |
| DLST | hsa-miR-4637 |
| DLST | hsa-miR-5584-5p |
| DLST | hsa-miR-2489 |
| DLST | hsa-miR-8075 |
| DLST | hsa-miR-106-3p |
| DLST | hsa-miR-197-3p |
| DLST | hsa-miR-7398t-3p |
| DLST | hsa-miR-8073 |
| DLST | hsa-miR-2448-3p |
| DLST | hsa-miR-3547-3p |
| DLST | hsa-miR-3475-3p |
| DLST | hsa-miR-651-3p |
| DLST | hsa-miR-3577 |
| DLST | hsa-miR-5002-3p |
| DLST | hsa-miR-8819 |
| DLST | hsa-miR-342-3p |
| DLST | hsa-miR-377-3p |
| DLST | hsa-miR-376c |
| DLST | hsa-miR-145-3p |
| DLST | hsa-miR-134-5p |
| DLST | hsa-miR-205-5p |
| DLST | hsa-miR-582-5p |
| DLST | hsa-miR-33 |
| DLST | hsa-miR-6768-5p |
| DLST | hsa-miR-3616-5p |
| DLST | hsa-miR-4433a-5p |
| DLST | hsa-miR-323a-5p |
| DLST | hsa-miR-1972 |
| DLST | hsa-miR-767-3p |
| DLST | hsa-miR-3176 |
| DLST | hsa-miR-892-3p |
| DLST | hsa-miR-3116 |
| DLST | hsa-miR-495-3p |
| DLST | hsa-miR-380-3p |
| DLST | hsa-miR-6912-5p |
| DLST | hsa-miR-7208-3p |
| DLST | hsa-miR-6076 |
| DLST | hsa-miR-6868-3p |
| DLST | hsa-miR-214-3p |
| DLST | hsa-miR-3583-5p |
| DLST | hsa-miR-7192-5p |
| DLST | hsa-miR-582-3p |
| DLST | hsa-miR-4691-5p |
| DLST | hsa-miR-1273e |
| DLST | hsa-miR-6811-3p |
| DLST | hsa-miR-514b-5p |
| DLST | hsa-miR-6876-5p |
| DLST | hsa-miR-1255-5p |
| DLST | hsa-miR-9-5p |
| DLST | hsa-miR-7084-5p |
| DLST | hsa-miR-29b-1-5p |
| DLST | hsa-miR-1298 |
| DLST | hsa-miR-5196-3p |
| DLST | hsa-miR-512-3p |
| DLST | hsa-miR-5693 |
| DLST | hsa-miR-3185 |
| DLST | hsa-miR-7197-3p |
| DLST | hsa-miR-548an |
| DLST | hsa-miR-6883-3p |
| DLST | hsa-miR-21-3p |
| DLST | hsa-miR-6990-5p |
| DLST | hsa-miR-516-3p |
| DLST | hsa-miR-369-5p |
| DLST | hsa-miR-4721 |
| DLST | hsa-miR-3963 |
| DLST | hsa-miR-7398u-3p |
| DLST | hsa-miR-769 |
| DLST | hsa-miR-23b-5p |
| DLST | hsa-miR-3474 |
| DLST | hsa-miR-134 |
| DLST | hsa-miR-16-2-3p |
| DLST | hsa-miR-3561-5p |
| DLST | hsa-miR-7381-5p |
| DLST | hsa-miR-2285c |
| DLST | hsa-miR-1184 |
| DLST | hsa-miR-1304-5p |
| DLST | hsa-miR-2898 |
| DLST | hsa-miR-548ae-3p |
| DLST | hsa-miR-548f-3p |
| DLST | hsa-let-7g-5p |
| DLST | hsa-miR-5114 |
| DLST | hsa-miR-3971 |
| DLST | hsa-miR-383-3p |
| DLST | hsa-miR-294-5p |
| DLST | hsa-miR-7205-5p |
| DLST | hsa-miR-4659b-5p |
| DLST | hsa-miR-6788-5p |
| DLST | hsa-miR-677-3p |
| DLST | hsa-miR-669p-3p |
| DLST | hsa-miR-599-5p |
| DLST | hsa-miR-2415-3p |
| DLST | hsa-miR-1960 |
| DLST | hsa-miR-8901 |
| DLST | hsa-let-7a-5p |
| DLST | hsa-miR-8795 |
| DLST | hsa-miR-1304-5p |
| DLST | hsa-miR-6879-5p |
| DLST | hsa-miR-7656-3p |
| DLST | hsa-miR-6990-3p |
| DLST | hsa-miR-7343-5p |
| DLST | hsa-miR-4797-3p |
| DLST | hsa-miR-1306-3p |
| DLST | hsa-miR-7374b-3p |
| DLST | hsa-miR-24-5p |
| DLST | hsa-miR-8863 |
| DLST | hsa-miR-7204-3p |
| DLST | hsa-miR-30a-3p |
| DLST | hsa-miR-3590-3p |
| DLST | hsa-miR-5194 |
| DLST | hsa-miR-423-3p |
| DLST | hsa-miR-1897-3p |
| DLST | hsa-miR-3076-5p |
| DLST | hsa-miR-30b-3p |
| DLST | hsa-miR-324-5p |
| DLST | hsa-miR-6991-5p |
| DLST | hsa-miR-2287 |
| DLST | hsa-miR-7325-5p |
| DLST | hsa-miR-3927-3p |
| DLST | hsa-miR-7386b-3p |
| DLST | hsa-miR-2453 |
| DLST | hsa-miR-7047-5p |
| DLST | hsa-miR-124-5p |
| DLST | hsa-miR-6874-3p |
| DLST | hsa-miR-18a-3p |
| DLST | hsa-miR-579 |
| DLST | hsa-miR-6977-5p |
| DLST | hsa-miR-3158-5p |
| DLST | hsa-miR-2441 |
| DLST | hsa-miR-582-5p |
| DLST | hsa-miR-711 |
| DLST | hsa-miR-30e-3p |
| DLST | hsa-miR-760-3p |
| DLST | hsa-miR-1260b |
| DLST | hsa-miR-5193 |
| DLST | hsa-miR-191-5p |
| DLST | hsa-miR-7203-3p |
| DLST | hsa-miR-7688-3p |
| DLST | hsa-miR-624-3p |
| DLST | hsa-miR-490-3p |
| DLST | hsa-miR-873-5p.1 |
| DLST | hsa-miR-7367-5p |
| DLST | hsa-miR-201-3p |
| DLST | hsa-miR-545-3p |
| DLST | hsa-miR-127-5p |
| DLST | hsa-miR-23a-5p |
| DLST | hsa-miR-6801-3p |
| DLST | hsa-miR-423-3p |
| DLST | hsa-miR-4323 |
| DLST | hsa-miR-1913 |
| DLST | hsa-miR-7287-5p |
| DLST | hsa-miR-7366-3p |
| DLST | hsa-miR-650abc |
| DLST | hsa-miR-384-3p |
| DLST | hsa-miR-344g-3p |
| DLST | hsa-miR-7179-3p |
| DLST | hsa-miR-138-3p |
| DLST | hsa-miR-6379 |
| DLST | hsa-miR-1273h-5p |
| DLST | hsa-miR-3190-5p |
| DLST | hsa-miR-30-3p |
| DLST | hsa-miR-2325b |
| DLST | hsa-miR-7195-5p |
| DLST | hsa-miR-548az-3p |
| DLST | hsa-miR-4775 |
| DLST | hsa-miR-296-5p |
| DLST | hsa-miR-210-5p |
| DLST | hsa-miR-8868 |
| DLST | hsa-miR-6992-5p |
| DLST | hsa-miR-7371a-3p |
| DLST | hsa-miR-129-5p |
| DLST | hsa-miR-680 |
| DLST | hsa-miR-15-5p |
| DLST | hsa-miR-3569-3p |
| DLST | hsa-miR-7200-5p |
| DLST | hsa-miR-7371e-3p |
| DLST | hsa-miR-7348-3p |
| DLST | hsa-miR-5702 |
| DLST | hsa-miR-5192 |
| DLST | hsa-miR-6823-5p |
| DLST | hsa-miR-508-3p |
| DLST | hsa-miR-548aq-3p |
| DLST | hsa-miR-1835 |
| DLST | hsa-miR-4291 |
| DLST | hsa-miR-196a-3p |
| DLST | hsa-miR-4726-3p |
| DLST | hsa-miR-1544b-5p |
| DLST | hsa-miR-2364 |
| DLST | hsa-miR-6758-5p |
| DLST | hsa-miR-4290 |
| DLST | hsa-miR-3591-3p |
| DLST | hsa-miR-7386i-5p |
| DLST | hsa-miR-1228-3p |
| DLST | hsa-miR-1254 |
| DLST | hsa-miR-1914-5p |
| DLST | hsa-miR-8850 |
| DLST | hsa-miR-7347-5p |
| DLST | hsa-miR-1271-3p |
| DLST | hsa-miR-540-3p |
| DLST | hsa-miR-7247-5p |
| DLST | hsa-miR-580 |
| DLST | hsa-miR-411-5p |
| DLST | hsa-miR-217-3p |
| DLST | hsa-miR-292b-5p |
| DLST | hsa-miR-2387 |
| DLST | hsa-miR-4534 |
| DLST | hsa-miR-548av-3p |
| DLST | hsa-miR-3059-3p |
| DLST | hsa-miR-3079-3p |
| DLST | hsa-miR-6721-5p |
| DLST | hsa-miR-3560 |
| DLST | hsa-miR-7150 |
| DLST | hsa-miR-329 |
| DLST | hsa-miR-524-5p |
| DLST | hsa-miR-30e |
| DLST | hsa-miR-30c-2-3p |
| DLST | hsa-miR-7216-5p |
| DLST | hsa-miR-6878-5p |
| DLST | hsa-miR-7089-3p |
| DLST | hsa-miR-15a-5p |
| DLST | hsa-miR-6898-3p |
| DLST | hsa-miR-6124 |
| DLST | hsa-miR-424-5p |
| DLST | hsa-miR-6734-3p |
| DLST | hsa-let-7d-5p |
| DLST | hsa-miR-7353-5p |
| DLST | hsa-miR-126-3p |
| DLST | hsa-miR-6797-3p |
| DLST | hsa-miR-449a |
| DLST | hsa-miR-7335-3p |
| DLST | hsa-miR-2285t |
| DLST | hsa-miR-490-3p |
| DLST | hsa-miR-2370-3p |
| DLST | hsa-miR-3714 |
| DLST | hsa-miR-6973b-3p |
| DLST | hsa-miR-3941 |
| DLST | hsa-miR-3912-3p |
| DLST | hsa-miR-669d-5p |
| DLST | hsa-miR-4510 |
| DLST | hsa-miR-149-5p |
| DLST | hsa-miR-660-3p |
| DLST | hsa-miR-7064-5p |
| DLST | hsa-miR-455-5p |
| DLST | hsa-miR-3098-5p |
| DLST | hsa-miR-7246-3p |
| DLST | hsa-miR-885 |
| DLST | hsa-miR-302c-3p |
| DLST | hsa-miR-5623-3p |
| DLST | hsa-miR-503-5p |
| DLST | hsa-miR-28-5p |
| DLST | hsa-miR-7002-5p |
| DLST | hsa-miR-2370-5p |
| DLST | hsa-miR-5700 |
| DLST | hsa-miR-548aj-3p |
| DLST | hsa-miR-6955-3p |
| DLST | hsa-miR-4672 |
| DLST | hsa-miR-4524b-3p |
| DLST | hsa-miR-1199-5p |
| DLST | hsa-miR-6879-3p |
| DLST | hsa-miR-8846 |
| DLST | hsa-miR-876 |
| DLST | hsa-miR-6348 |
| DLST | hsa-miR-7344-5p |
| DLST | hsa-miR-339-5p |
| DLST | hsa-miR-411-5p.1 |
| DLST | hsa-miR-7043-3p |
| DLST | hsa-miR-7195-3p |
| DLST | hsa-miR-669e-5p |
| DLST | hsa-miR-3127-3p |
| DLST | hsa-miR-1252-5p |
| DLST | hsa-miR-7217-3p |
| DLST | hsa-miR-488-5p |
| DLST | hsa-miR-188-5p |
| DLST | hsa-miR-2447 |
| DLST | hsa-miR-412-3p |
| DLST | hsa-miR-593-3p |
| DLST | hsa-miR-224-5p |
| DLST | hsa-miR-1260a |
| DLST | hsa-miR-8822 |
| DLST | hsa-miR-6997-5p |
| DLST | hsa-miR-6940-3p |
| DLST | hsa-miR-195-5p |
| DLST | hsa-miR-6908-3p |
| DLST | hsa-miR-504-3p |
| DLST | hsa-miR-3936 |
| DLST | hsa-miR-483-3p.1 |
| DLST | hsa-miR-667-3p |
| DLST | hsa-miR-875 |
| DLST | hsa-miR-523-3p |
| DLST | hsa-miR-4669 |
| DLST | hsa-miR-891b |
| DLST | hsa-miR-148b-5p |
| DLST | hsa-miR-18 |
| DLST | hsa-miR-6894-3p |
| DLST | hsa-miR-432-5p |
| DLST | hsa-miR-6886-3p |
| DLST | hsa-miR-7042-3p |
| DLST | hsa-miR-7324-3p |
| DLST | hsa-miR-8834b |
| DLST | hsa-miR-9500 |
| DLST | hsa-miR-107 |
| DLST | hsa-miR-8082 |
| DLST | hsa-miR-4521 |
| DLST | hsa-miR-7010-5p |
| DLST | hsa-miR-221-5p |
| DLST | hsa-miR-4690-3p |
| DLST | hsa-miR-4441 |
| DLST | hsa-miR-6740-3p |
| DLST | hsa-miR-193b-5p |
| DLST | hsa-miR-6779-5p |
| DLST | hsa-miR-6868-5p |
| DLST | hsa-miR-7659-5p |
| DLST | hsa-miR-881-5p |
| DLST | hsa-miR-873-5p.2 |
| DLST | hsa-miR-1-3p |
| DLST | hsa-miR-1237-3p |
| DLST | hsa-miR-2285e |
| DLST | hsa-miR-544a |
| DLST | hsa-miR-2284q |
| DLST | hsa-miR-4301 |
| DLST | hsa-miR-4668-3p |
| DLST | hsa-miR-339-5p |
| DLST | hsa-miR-138-5p |
| DLST | hsa-miR-18-3p |
| DLST | hsa-miR-16-1-3p |
| DLST | hsa-miR-6809-3p |
| DLST | hsa-miR-593-3p |
| DLST | hsa-miR-326 |
| DLST | hsa-miR-6324 |
| DLST | hsa-miR-1296 |
| DLST | hsa-miR-4270 |
| DLST | hsa-miR-7027-5p |
| DLST | hsa-miR-124-3p.2 |
| DLST | hsa-miR-466 |
| DLST | hsa-miR-99-3p |
| DLST | hsa-miR-7176-3p |
| DLST | hsa-miR-548b-3p |
| DLST | hsa-miR-377-3p |
| DLST | hsa-miR-133a-3p.2 |
| DLST | hsa-miR-2307 |
| DLST | hsa-miR-2306 |
| DLST | hsa-miR-8849 |
| DLST | hsa-miR-1668 |
| DLST | hsa-miR-1224 |
| DLST | hsa-miR-922 |
| DLST | hsa-miR-5008-3p |
| DLST | hsa-miR-8805 |
| DLST | hsa-miR-769-5p |
| DLST | hsa-miR-1540 |
| DLST | hsa-miR-6810-3p |
| DLST | hsa-miR-6808-5p |
| DLST | hsa-miR-2450bd |
| DLST | hsa-miR-6753-5p |
| DLST | hsa-miR-493-3p |
| DLST | hsa-miR-6354 |
| DLST | hsa-miR-6715b-5p |
| DLST | hsa-miR-502-5p |
| DLST | hsa-miR-7262b-5p |
| DLST | hsa-miR-6522 |
| DLST | hsa-miR-3150b-3p |
| DLST | hsa-miR-1584-3p |
| DLST | hsa-miR-155-3p |
| DLST | hsa-miR-6988-3p |
| DLST | hsa-miR-507b |
| DLST | hsa-miR-1282 |
| DLST | hsa-miR-7351-5p |
| DLST | hsa-miR-7215-5p |
| DLST | hsa-miR-5008-5p |
| DLST | hsa-miR-3097-5p |
| DLST | hsa-miR-3184-3p |
| DLST | hsa-miR-6127 |
| DLST | hsa-miR-208-3p |
| DLST | hsa-miR-2373-5p |
| DLST | hsa-miR-1249-5p |
| DLST | hsa-miR-710 |
| DLST | hsa-miR-3590-5p |
| DLST | hsa-miR-4516 |
| DLST | hsa-miR-7245-5p |
| DLST | hsa-miR-7279-5p |
| DLST | hsa-miR-7020-5p |
| DLST | hsa-miR-3155a |
| DLST | hsa-miR-4419a |
| DLST | hsa-miR-2115-5p |
| DLST | hsa-miR-3914 |
| DLST | hsa-miR-6887-3p |
| DLST | hsa-miR-130a-5p |
| DLST | hsa-miR-30ad-3p |
| DLST | hsa-miR-6715-5p |
| DLST | hsa-miR-6996-5p |
| DLST | hsa-miR-8827 |
| DLST | hsa-miR-206 |
| DLST | hsa-miR-8792 |
| DLST | hsa-miR-520c-3p |
| DLST | hsa-miR-7162-5p |
| DLST | hsa-miR-4511 |
| DLST | hsa-miR-2331-5p |
| DLST | hsa-miR-1291 |
| DLST | hsa-miR-495-3p |
| DLST | hsa-miR-6735-3p |
| DLST | hsa-miR-6908-5p |
| DLST | hsa-miR-8063 |
| DLST | hsa-miR-328-3p |
| DLST | hsa-miR-681 |
| DLST | hsa-miR-30d-3p |
| DLST | hsa-miR-199-3p |
| DLST | hsa-miR-15 |
| DLST | hsa-miR-193 |
| DLST | hsa-miR-672-3p |
| DLST | hsa-miR-7068-5p |
| DLST | hsa-miR-6798-5p |
| DLST | hsa-miR-2285aa |
| DLST | hsa-miR-302d-5p |
| DLST | hsa-miR-19-3p |
| DLST | hsa-miR-3103-5p |
| DLST | hsa-miR-7016-3p |
| DLST | hsa-miR-5627-5p |
| DLST | hsa-miR-3541 |
| DLST | hsa-miR-1912 |
| DLST | hsa-miR-1227-3p |
| DLST | hsa-miR-7252-5p |
| DLST | hsa-miR-770-5p |
| DLST | hsa-miR-4288 |
| DLST | hsa-miR-6502-5p |
| DLST | hsa-miR-7286-3p |
| DLST | hsa-miR-4685-3p |
| DLST | hsa-miR-7224-3p |
| DLST | hsa-miR-196-5p |
| DLST | hsa-miR-4768-5p |
| DLST | hsa-miR-376a-5p |
| DLST | hsa-miR-1283 |
| DLST | hsa-miR-3144-5p |
| DLST | hsa-miR-376c-5p |
| DLST | hsa-miR-2449 |
| DLST | hsa-miR-7371b-3p |
| DLST | hsa-miR-511-5p |
| DLST | hsa-miR-5187-3p |
| DLST | hsa-miR-122 |
| DLST | hsa-miR-7663-5p |
| DLST | hsa-miR-187-5p |
| DLST | hsa-miR-214-3p |
| DLST | hsa-miR-6833-3p |
| DLST | hsa-miR-137-3p |
| DLST | hsa-miR-6947-3p |
| DLST | hsa-miR-2894 |
| DLST | hsa-miR-6735-5p |
| DLST | hsa-miR-1251-5p |
| DLST | hsa-miR-484 |
| DLST | hsa-miR-30c-3p |
| DLST | hsa-miR-210-3p |
| DLST | hsa-miR-302 |
| DLST | hsa-miR-193-3p |
| DLST | hsa-miR-1194 |
| DLST | hsa-miR-5617-5p |
| DLST | hsa-miR-1837 |
| DLST | hsa-miR-6769b-5p |
| DLST | hsa-miR-222-3p |
| DLST | hsa-miR-7109-3p |
| DLST | hsa-miR-548j-3p |
| DLST | hsa-miR-662 |
| DLST | hsa-miR-7007-5p |
| DLST | hsa-miR-135a-1-3p |
| DLST | hsa-miR-4680-3p |
| DLST | hsa-miR-2396 |
| DLST | hsa-miR-1838 |
| DLST | hsa-miR-7351-3p |
| DLST | hsa-miR-548i-3p |
| DLST | hsa-miR-8891 |
| DLST | hsa-miR-384 |
| DLST | hsa-miR-146 |
| DLST | hsa-miR-128 |
| DLST | hsa-miR-7361-5p |
| DLST | hsa-miR-6791-3p |
| DLST | hsa-miR-891 |
| DLST | hsa-miR-759 |
| DLST | hsa-miR-7688-5p |
| DLST | hsa-miR-3152-5p |
| DLST | hsa-miR-105-5p |
| DLST | hsa-miR-7683-3p |
| DLST | hsa-miR-4303 |
| DLST | hsa-miR-7-5p |
| DLST | hsa-miR-34b-3p |
| DLST | hsa-miR-4774-3p |
| DLST | hsa-miR-5186 |
| DLST | hsa-miR-135b-3p |
| DLST | hsa-miR-344-5p |
| DLST | hsa-miR-1255 |
| DLST | hsa-miR-7094b-2-5p |
| DLST | hsa-miR-3692-5p |
| DLST | hsa-miR-6893-5p |
| DLST | hsa-miR-502-5p |
| DLST | hsa-miR-130-3p |
| DLST | hsa-miR-1231-3p |
| DLST | hsa-miR-409-5p |
| DLST | hsa-miR-7243-3p |
| DLST | hsa-miR-548x-3p |
| DLST | hsa-miR-7178-3p |
| DLST | hsa-miR-4712-3p |
| DLST | hsa-miR-7661-5p |
| DLST | hsa-miR-885-5p |
| DLST | hsa-miR-5001-3p |
| DLST | hsa-miR-7385a-3p |
| DLST | hsa-miR-3125 |
| DLST | hsa-miR-301-3p |
| DLST | hsa-miR-7398q-3p |
| DLST | hsa-miR-489-3p |
| DLST | hsa-miR-4277 |
| DLST | hsa-miR-6760-5p |
| DLST | hsa-miR-1911-3p |
| DLST | hsa-miR-524-3p |
| DLST | hsa-miR-7106-5p |
| DLST | hsa-miR-465-3p |
| DLST | hsa-miR-3566 |
| DLST | hsa-miR-690 |
| DLST | hsa-miR-7171-5p |
| DLST | hsa-miR-622 |
| DLST | hsa-miR-1293 |
| DLST | hsa-miR-7264-3p |
| DLST | hsa-miR-548p |
| DLST | hsa-miR-7359-5p |
| DLST | hsa-miR-129-2-3p |
| DLST | hsa-miR-223-5p |
| DLST | hsa-miR-17 |
| DLST | hsa-miR-508-3p |
| DLST | hsa-miR-3657 |
| DLST | hsa-miR-4518 |
| DLST | hsa-miR-4533 |
| DLST | hsa-miR-4254 |
| DLST | hsa-miR-6754-5p |
| DLST | hsa-miR-6765-5p |
| DLST | hsa-miR-222-5p |
| DLST | hsa-miR-7322-5p |
| DLST | hsa-miR-2427 |
| DLST | hsa-miR-152-5p |
| DLST | hsa-miR-4711-3p |
| DLST | hsa-miR-485-5p |
| DLST | hsa-miR-5615-3p |
| DLST | hsa-miR-589-5p |
| DLST | hsa-miR-485 |
| DLST | hsa-miR-450b |
| DLST | hsa-miR-7681-3p |
| DLST | hsa-miR-876-3p |
| DLST | hsa-miR-1470 |
| DLST | hsa-miR-6514-3p |
| DLST | hsa-miR-3057-3p |
| DLST | hsa-miR-2381 |
| DLST | hsa-miR-6764-5p |
| DLST | hsa-miR-6961-3p |
| DLST | hsa-miR-6972-5p |
| DLST | hsa-miR-7398e-3p |
| DLST | hsa-miR-141-3p |
| DLST | hsa-miR-8903 |
| DLST | hsa-miR-6829-3p |
| DLST | hsa-miR-3156-3p |
| DLST | hsa-miR-302bd |
| DLST | hsa-miR-7258-5p |
| DLST | hsa-miR-4717-3p |
| DLST | hsa-miR-7652-3p |
| DLST | hsa-miR-7680-3p |
| DLST | hsa-miR-8844 |
| DLST | hsa-miR-6851-3p |
| DLST | hsa-miR-133b |
| DLST | hsa-miR-5125 |
| DLST | hsa-miR-4650-5p |
| DLST | hsa-miR-125 |
| DLST | hsa-miR-365-3p |
| DLST | hsa-miR-3612 |
| DLST | hsa-miR-4653-5p |
| DLST | hsa-miR-8066 |
| DLST | hsa-miR-137 |
| DLST | hsa-miR-7082-3p |
| DLST | hsa-miR-105-5p |
| DLST | hsa-miR-1981-5p |
| DLST | hsa-miR-7200-3p |
| DLST | hsa-miR-470-5p |
| DLST | hsa-miR-763 |
| DLST | hsa-miR-4474-5p |
| DLST | hsa-miR-7157-3p |
| DLST | hsa-miR-133a-3p.1 |
| DLST | hsa-miR-532-5p |
| DLST | hsa-miR-5089-3p |
| DLST | hsa-miR-7386e-3p |
| DLST | hsa-miR-4427 |
| DLST | hsa-miR-145-5p |
| DLST | hsa-miR-135-5p |
| DLST | hsa-miR-8801 |
| DLST | hsa-miR-7277-5p |
| DLST | hsa-miR-6519 |
| DLST | hsa-miR-548a-3p |
| DLST | hsa-miR-98-5p |
| DLST | hsa-miR-144-3p |
| DLST | hsa-miR-6821-3p |
| DLST | hsa-miR-1266-5p |
| DLST | hsa-miR-322-3p |
| DLST | hsa-miR-5691 |
| DLST | hsa-miR-7386i-3p |
| DLST | hsa-miR-34-5p |
| DLST | hsa-miR-3606-5p |
| DLST | hsa-miR-10 |
| DLST | hsa-miR-600 |
| DLST | hsa-miR-6340 |
| DLST | hsa-miR-133 |
| DLST | hsa-miR-7978 |
| DLST | hsa-miR-7065-3p |
| DLST | hsa-miR-505-5p |
| DLST | hsa-miR-1261 |
| DLST | hsa-miR-2896 |
| DLST | hsa-miR-7350-3p |
| DLST | hsa-miR-939-3p |
| DLST | hsa-miR-7037-5p |
| DLST | hsa-miR-497-3p |
| DLST | hsa-miR-346 |
| DLST | hsa-miR-1237-3p |
| DLST | hsa-miR-7329-5p |
| DLST | hsa-miR-624-3p |
| DLST | hsa-miR-7086-5p |
| DLST | hsa-miR-133-3p.1 |
| DLST | hsa-miR-7-5p |
| DLST | hsa-miR-3160-3p |
| DLST | hsa-miR-7287-3p |
| DLST | hsa-miR-2303 |
| DLST | hsa-miR-7319-5p |
| DLST | hsa-miR-1-3p |
| DLST | hsa-miR-135-3p |
| DLST | hsa-miR-1301 |
| DLST | hsa-miR-761 |
| DLST | hsa-miR-6773-3p |
| DLST | hsa-miR-519 |
| DLST | hsa-miR-197-3p |
| DLST | hsa-miR-338-3p |
| DLST | hsa-miR-4524a-3p |
| DLST | hsa-miR-328 |
| DLST | hsa-miR-4523 |
| DLST | hsa-miR-548ah-3p |
| DLST | hsa-miR-20-3p |
| DLST | hsa-miR-1207-5p |
| DLST | hsa-miR-1247-3p |
| DLST | hsa-miR-1322 |
| DLST | hsa-miR-7374-5p |
| DLST | hsa-miR-379-3p |
| DLST | hsa-miR-6828-3p |
| DLST | hsa-miR-3927-3p |
| DLST | hsa-miR-7177-5p |
| DLST | hsa-miR-140-3p.1 |
| DLST | hsa-miR-7228-3p |
| DLST | hsa-miR-3551-5p |
| DLST | hsa-miR-7365-3p |
| DLST | hsa-miR-8085 |
| DLST | hsa-miR-2422 |
| DLST | hsa-miR-5616-3p |
| DLST | hsa-miR-146-5p |
| DLST | hsa-miR-467eh-5p |
| DLST | hsa-miR-5680 |
| DLST | hsa-miR-8885 |
| DLST | hsa-miR-3120-3p |
| DLST | hsa-miR-4463 |
| DLST | hsa-miR-3544 |
| DLST | hsa-miR-2379 |
| DLST | hsa-miR-7011-3p |
| DLST | hsa-miR-338-3p |
| DLST | hsa-miR-124 |
| DLST | hsa-miR-378g |
| DLST | hsa-miR-7292-3p |
| DLST | hsa-miR-499-5p |
| DLST | hsa-miR-5123 |
| DLST | hsa-let-7-5p |
| DLST | hsa-miR-1547-5p |
| DLST | hsa-miR-4480 |
| DLST | hsa-miR-7307-3p |
| DLST | hsa-miR-9a-3p |
| DLST | hsa-miR-6831-5p |
| DLST | hsa-miR-2334 |
| DLST | hsa-miR-2285z |
| DLST | hsa-miR-33-5p |
| DLST | hsa-miR-218-3p |
| DLST | hsa-miR-196a-2-3p |
| DLST | hsa-miR-5088-3p |
| DLST | hsa-miR-3065-3p |
| DLST | hsa-miR-589-3p |
| DLST | hsa-miR-7355-5p |
| DLST | hsa-miR-507 |
| DLST | hsa-miR-149-3p |
| DLST | hsa-miR-7327-5p |
| DLST | hsa-miR-8057 |
| DLST | hsa-miR-542-5p |
| DLST | hsa-miR-1252 |
| DLST | hsa-miR-7381-3p |
| DLST | hsa-miR-8120 |
| DLST | hsa-miR-424-5p |
| DLST | hsa-miR-7362-3p |
| DLST | hsa-miR-4761-5p |
| DLST | hsa-miR-573 |
| DLST | hsa-miR-4686 |
| DLST | hsa-miR-7290-5p |
| DLST | hsa-miR-2285g |
| DLST | hsa-miR-532-3p |
| DLST | hsa-miR-5096 |
| DLST | hsa-miR-3164 |
| DLST | hsa-miR-924 |
| DLST | hsa-miR-2285s |
| DLST | hsa-miR-5585-3p |
| DLST | hsa-miR-548b-3p |
| DLST | hsa-let-7c-5p |
| DLST | hsa-miR-7015-5p |
| DLST | hsa-miR-3685 |
| DLST | hsa-miR-3074-5p |
| DLST | hsa-miR-516b-3p |
| DLST | hsa-miR-875-5p |
| DLST | hsa-miR-22-3p |
| DLST | hsa-miR-940 |
| DLST | hsa-miR-938 |
| DLST | hsa-miR-6965-5p |
| DLST | hsa-miR-6780a-5p |
| DLST | hsa-miR-7371j-3p |
| DLST | hsa-miR-6892-3p |
| DLST | hsa-miR-3649 |
| DLST | hsa-miR-516a |
| DLST | hsa-miR-669m-5p |
| DLST | hsa-miR-802-3p |
| DLST | hsa-miR-9-3p |
| DLST | hsa-miR-6939-3p |
| DLST | hsa-miR-330-5p |
| DLST | hsa-miR-295-5p |
| DLST | hsa-miR-4436b-3p |
| DLST | hsa-miR-550b-2-5p |
| DLST | hsa-miR-7373-5p |
| DLST | hsa-miR-7292-5p |
| DLST | hsa-miR-4778-3p |
| DLST | hsa-miR-1843 |
| DLST | hsa-miR-5703 |
| DLST | hsa-miR-1271-5p |
| DLST | hsa-miR-1260a |
| DLST | hsa-miR-4632-5p |
| DLST | hsa-miR-7322-3p |
| DLST | hsa-miR-429-3p |
| DLST | hsa-miR-520g-5p |
| DLST | hsa-miR-3916 |
| DLST | hsa-miR-4720-3p |
| DLST | hsa-miR-692 |
| DLST | hsa-let-7i-5p |
| DLST | hsa-miR-7371-3p |
| DLST | hsa-miR-497-5p |
| DLST | hsa-miR-208-5p |
| DLST | hsa-miR-193-5p |
| DLST | hsa-miR-5092 |
| DLST | hsa-miR-4659a-5p |
| DLST | hsa-miR-6817-5p |
| DLST | hsa-miR-186-5p |
| DLST | hsa-miR-4733-3p |
| DLST | hsa-miR-4453 |
| DLST | hsa-miR-1272 |
| DLST | hsa-miR-466-5p |
| DLST | hsa-miR-1468-3p |
| DLST | hsa-miR-552-3p |
| DLST | hsa-miR-326-3p |
| DLST | hsa-miR-96-5p |
| DLST | hsa-miR-324-3p |
| DLST | hsa-miR-7668-3p |
| DLST | hsa-miR-7032-5p |
| DLST | hsa-miR-3134 |
| DLST | hsa-miR-4437 |
| DLST | hsa-miR-1915-3p |
| DLST | hsa-miR-3593-3p |
| DLST | hsa-miR-7282-5p |
| DLST | hsa-miR-7235-5p |
| DLST | hsa-miR-214-5p |
| DLST | hsa-miR-6836-3p |
| DLST | hsa-miR-328-5p |
| DLST | hsa-miR-7283-3p |
| DLST | hsa-miR-875-3p |
| DLST | hsa-miR-4684-5p |
| DLST | hsa-miR-518-5p |
| DLST | hsa-miR-411-3p |
| DLST | hsa-miR-431-5p |
| DLST | hsa-miR-34b-3p |
| DLST | hsa-miR-216c-3p |
| DLST | hsa-miR-2359 |
| DLST | hsa-miR-548v |
| DLST | hsa-miR-5000-3p |
| DLST | hsa-miR-634 |
| DLST | hsa-miR-3680-3p |
| DLST | hsa-miR-434-5p |
| DLST | hsa-miR-4328 |
| DLST | hsa-miR-2309 |
| DLST | hsa-miR-361-5p |
| DLST | hsa-miR-7041-5p |
| DLST | hsa-miR-298 |
| DLST | hsa-miR-552 |
| DLST | hsa-miR-7669-3p |
| DLST | hsa-miR-330-3p |
| DLST | hsa-miR-549a |
| DLST | hsa-miR-4662a-3p |
| DLST | hsa-miR-7272-3p |
| DLST | hsa-miR-3194-3p |
| DLST | hsa-miR-2308 |
| DLST | hsa-miR-7368-5p |
| DLST | hsa-miR-7019-3p |
| DLST | hsa-miR-2389 |
| DLST | hsa-miR-4801 |
| DLST | hsa-miR-3595 |
| DLST | hsa-miR-124-3p |
| DLST | hsa-miR-371 |
| DLST | hsa-miR-873-5p |
| DLST | hsa-miR-5108 |
| DLST | hsa-miR-6861-3p |
| DLST | hsa-miR-7004-5p |
| DLST | hsa-miR-411-5p |
| DLST | hsa-miR-29-5p |
| DLST | hsa-miR-7185-3p |
| DLST | hsa-miR-140-5p |
| DLST | hsa-miR-7660-5p |
| DLST | hsa-miR-6133 |
| DLST | hsa-miR-7063-3p |
| DLST | hsa-miR-7312-3p |
| DLST | hsa-miR-423-5p |
| DLST | hsa-miR-152-3p |
| DLST | hsa-miR-1843a-5p |
| DLST | hsa-miR-6799-5p |
| DLST | hsa-miR-598-3p |
| DLST | hsa-miR-376e |
| DLST | hsa-miR-335-3p |
| DLST | hsa-miR-1545c-5p |
| DLST | hsa-miR-2351 |
| DLST | hsa-miR-7386d-5p |
| DLST | hsa-miR-2376 |
| DLST | hsa-miR-330-3p.1 |
| DLST | hsa-miR-4476 |
| DLST | hsa-miR-17-3p |
| DLST | hsa-miR-6963-3p |
| DLST | hsa-miR-3978 |
| DLST | hsa-miR-449-5p |
| DLST | hsa-miR-150-5p |
| DLST | hsa-miR-148-5p |
| DLST | hsa-miR-4769-3p |
| DLST | hsa-miR-1266 |
| DLST | hsa-miR-6883-5p |
| DLST | hsa-miR-548e-5p |
| DLST | hsa-miR-654-5p |
| DLST | hsa-miR-636 |
| DLST | hsa-miR-3975 |
| DLST | hsa-let-7b-5p |
| DLST | hsa-miR-504 |
| DLST | hsa-miR-7382-5p |
| DLST | hsa-miR-3967 |
| DLST | hsa-miR-7081-3p |
| DLST | hsa-miR-4736 |
| DLST | hsa-miR-6728-3p |
| DLST | hsa-miR-215-3p |
| DLST | hsa-miR-302-5p |
| DLST | hsa-let-7f-5p |
| DLST | hsa-miR-6818-5p |
| DLST | hsa-miR-5133 |
| DLST | hsa-miR-650 |
| DLST | hsa-miR-296-3p |
| DLST | hsa-miR-6965-3p |
| DLST | hsa-miR-6749-3p |
| DLST | hsa-miR-153-5p |
| DLST | hsa-miR-1227-3p |
| DLST | hsa-miR-1643a-5p |
| DLST | hsa-miR-1205 |
| DLST | hsa-miR-6756-3p |
| DLST | hsa-miR-6384 |
| DLST | hsa-miR-188-3p |
| DLST | hsa-miR-331-3p |
| DLST | hsa-miR-2322-5p |
| DLST | hsa-miR-409a |
| DLST | hsa-miR-3616-3p |
| DLST | hsa-miR-6919-5p |
| DLST | hsa-miR-6750-5p |
| DLST | hsa-miR-7198-3p |
| DLST | hsa-miR-138-5p |
| DLST | hsa-miR-7045-3p |
| DLST | hsa-miR-6388 |
| DLST | hsa-miR-16-3p |
| DLST | hsa-miR-3600 |
| DLST | hsa-miR-7026-3p |
| DLST | hsa-miR-6830-5p |
| DLST | hsa-miR-28-5p |
| DLST | hsa-miR-148a-5p |
| DLST | hsa-miR-3573-3p |
| DLST | hsa-miR-548aef |
| DLST | hsa-miR-873-5p |
| DLST | hsa-miR-425-5p |
| DLST | hsa-miR-1226-3p |
| DLST | hsa-miR-125b-2-3p |
| DLST | hsa-miR-7005-3p |
| DLST | hsa-miR-3667-3p |
| DLST | hsa-miR-7370-5p |
| DLST | hsa-miR-221-3p |
| DLST | hsa-miR-580-5p |
| DLST | hsa-miR-6937-3p |
| DLST | hsa-miR-877-5p |
| DLST | hsa-miR-548am-3p |
| DLST | hsa-miR-218-5p |
| DLST | hsa-miR-302b-5p |
| DLST | hsa-miR-3922-5p |
| DLST | hsa-miR-7218-3p |
| DLST | hsa-miR-2439-5p |
| DLST | hsa-miR-7386h-3p |
| DLST | hsa-miR-6957-5p |
| DLST | hsa-miR-7017-5p |
| DLST | hsa-miR-7339-3p |
| DLST | hsa-miR-6926-3p |
| FDX1 | hsa-miR-8063 |
| FDX1 | hsa-miR-890-5p |
| FDX1 | hsa-miR-6540-3p |
| FDX1 | hsa-miR-7683-3p |
| FDX1 | hsa-miR-641 |
| FDX1 | hsa-miR-640 |
| FDX1 | hsa-miR-4728-5p |
| FDX1 | hsa-miR-4289 |
| FDX1 | hsa-miR-661 |
| FDX1 | hsa-miR-345-5p |
| FDX1 | hsa-miR-4276 |
| FDX1 | hsa-miR-150-5p |
| FDX1 | hsa-miR-3065-5p |
| FDX1 | hsa-miR-494 |
| FDX1 | hsa-miR-636 |
| FDX1 | hsa-let-7abf-3p |
| FDX1 | hsa-miR-9-5p |
| FDX1 | hsa-miR-7169-3p |
| FDX1 | hsa-miR-5683 |
| FDX1 | hsa-miR-6723-5p |
| FDX1 | hsa-miR-548ar-5p |
| FDX1 | hsa-miR-202-5p |
| FDX1 | hsa-miR-4455 |
| FDX1 | hsa-miR-342-3p |
| FDX1 | hsa-miR-7371d-5p |
| FDX1 | hsa-miR-295-5p |
| FDX1 | hsa-miR-4669 |
| FDX1 | hsa-miR-889-5p |
| FDX1 | hsa-miR-412-3p |
| FDX1 | hsa-miR-6499-3p |
| FDX1 | hsa-miR-490-3p |
| FDX1 | hsa-miR-3109-5p |
| FDX1 | hsa-miR-9-3p |
| FDX1 | hsa-miR-6769b-5p |
| FDX1 | hsa-miR-5620-3p |
| FDX1 | hsa-miR-132-3p |
| FDX1 | hsa-miR-3558-5p |
| FDX1 | hsa-miR-548g-5p |
| FDX1 | hsa-miR-294-5p |
| FDX1 | hsa-miR-2285q |
| FDX1 | hsa-miR-6481 |
| FDX1 | hsa-miR-3147 |
| FDX1 | hsa-miR-2284q |
| FDX1 | hsa-miR-6509-5p |
| FDX1 | hsa-miR-2284ab |
| FDX1 | hsa-miR-335-3p |
| FDX1 | hsa-miR-6789-3p |
| FDX1 | hsa-miR-1254 |
| FDX1 | hsa-miR-3548 |
| FDX1 | hsa-miR-3473f |
| FDX1 | hsa-miR-550a-5p |
| FDX1 | hsa-miR-3120-3p |
| FDX1 | hsa-miR-7678-3p |
| FDX1 | hsa-miR-887-5p |
| FDX1 | hsa-miR-6081 |
| FDX1 | hsa-miR-4419b |
| FDX1 | hsa-miR-7293-5p |
| FDX1 | hsa-miR-4768-3p |
| FDX1 | hsa-miR-7371b-5p |
| FDX1 | hsa-miR-4762-3p |
| FDX1 | hsa-miR-1714 |
| FDX1 | hsa-miR-3612 |
| FDX1 | hsa-miR-32-3p |
| FDX1 | hsa-miR-2284d |
| FDX1 | hsa-miR-4297 |
| FDX1 | hsa-miR-548at-3p |
| FDX1 | hsa-miR-5195-5p |
| FDX1 | hsa-miR-3941 |
| FDX1 | hsa-miR-7229-3p |
| FDX1 | hsa-miR-580 |
| FDX1 | hsa-miR-892b |
| FDX1 | hsa-miR-1241 |
| FDX1 | hsa-miR-8838 |
| FDX1 | hsa-miR-4318 |
| FDX1 | hsa-miR-3087-3p |
| FDX1 | hsa-miR-2467-3p |
| FDX1 | hsa-miR-7034-3p |
| FDX1 | hsa-miR-625-3p |
| FDX1 | hsa-miR-7212-3p |
| FDX1 | hsa-miR-518-5p |
| FDX1 | hsa-miR-874-3p |
| FDX1 | hsa-miR-4422 |
| FDX1 | hsa-miR-6788-5p |
| FDX1 | hsa-miR-3121-3p |
| FDX1 | hsa-miR-221-5p |
| FDX1 | hsa-miR-193-3p |
| FDX1 | hsa-miR-4780 |
| FDX1 | hsa-miR-6120-3p |
| FDX1 | hsa-miR-343 |
| FDX1 | hsa-miR-4303 |
| FDX1 | hsa-miR-4537 |
| FDX1 | hsa-miR-7215-3p |
| FDX1 | hsa-miR-585-5p |
| FDX1 | hsa-miR-7649-5p |
| FDX1 | hsa-miR-7377-3p |
| FDX1 | hsa-miR-374b-3p |
| FDX1 | hsa-miR-7373d-3p |
| FDX1 | hsa-miR-2307 |
| FDX1 | hsa-miR-577 |
| FDX1 | hsa-miR-6808-5p |
| FDX1 | hsa-miR-181-5p |
| FDX1 | hsa-miR-450a-2-3p |
| FDX1 | hsa-miR-7089-3p |
| FDX1 | hsa-miR-500b-5p |
| FDX1 | hsa-miR-4799-3p |
| FDX1 | hsa-miR-4714-5p |
| FDX1 | hsa-miR-510-3p |
| FDX1 | hsa-miR-200b-5p |
| FDX1 | hsa-miR-543-5p |
| FDX1 | hsa-miR-3166 |
| FDX1 | hsa-miR-5681a |
| FDX1 | hsa-miR-7198-3p |
| FDX1 | hsa-miR-3664-5p |
| FDX1 | hsa-miR-7977 |
| FDX1 | hsa-miR-584-5p |
| FDX1 | hsa-miR-155-5p |
| FDX1 | hsa-miR-520-3p |
| FDX1 | hsa-miR-6840-5p |
| FDX1 | hsa-miR-4733-3p |
| FDX1 | hsa-miR-1237-3p |
| FDX1 | hsa-miR-7688-5p |
| FDX1 | hsa-miR-665 |
| FDX1 | hsa-miR-488-3p |
| FDX1 | hsa-miR-1815 |
| FDX1 | hsa-miR-3129-5p |
| FDX1 | hsa-miR-432-3p |
| FDX1 | hsa-miR-548an |
| FDX1 | hsa-miR-7077-3p |
| FDX1 | hsa-miR-5000-5p |
| FDX1 | hsa-miR-6752-3p |
| FDX1 | hsa-miR-322-5p |
| FDX1 | hsa-miR-6973a-3p |
| FDX1 | hsa-miR-15a-3p |
| FDX1 | hsa-miR-6894-3p |
| FDX1 | hsa-miR-513a |
| FDX1 | hsa-miR-3928-3p |
| FDX1 | hsa-miR-6984-5p |
| FDX1 | hsa-miR-4763-5p |
| FDX1 | hsa-miR-5101 |
| FDX1 | hsa-miR-2117 |
| FDX1 | hsa-miR-4698 |
| FDX1 | hsa-miR-550-5p |
| FDX1 | hsa-miR-7273-5p |
| FDX1 | hsa-miR-7386l-5p |
| FDX1 | hsa-miR-8092 |
| FDX1 | hsa-miR-2399-5p |
| FDX1 | hsa-miR-135 |
| FDX1 | hsa-miR-5584-3p |
| FDX1 | hsa-miR-361-3p |
| FDX1 | hsa-miR-491-3p |
| FDX1 | hsa-miR-5581-5p |
| FDX1 | hsa-miR-7010-5p |
| FDX1 | hsa-miR-8867 |
| FDX1 | hsa-miR-7116-5p |
| FDX1 | hsa-miR-539-5p |
| FDX1 | hsa-miR-138-5p |
| FDX1 | hsa-miR-4540 |
| FDX1 | hsa-miR-7259-5p |
| FDX1 | hsa-miR-548ab |
| FDX1 | hsa-miR-3646 |
| FDX1 | hsa-miR-4323 |
| FDX1 | hsa-miR-4437 |
| FDX1 | hsa-miR-23b-5p |
| FDX1 | hsa-miR-454-5p |
| FDX1 | hsa-miR-3658 |
| FDX1 | hsa-miR-432-5p |
| FDX1 | hsa-miR-4327 |
| FDX1 | hsa-miR-336-5p |
| FDX1 | hsa-miR-889-3p |
| FDX1 | hsa-miR-505-5p |
| FDX1 | hsa-miR-2377 |
| FDX1 | hsa-miR-548ad-5p |
| FDX1 | hsa-miR-1299 |
| FDX1 | hsa-miR-2455 |
| FDX1 | hsa-miR-136-5p |
| FDX1 | hsa-miR-6134 |
| FDX1 | hsa-miR-7160-5p |
| FDX1 | hsa-miR-6359 |
| FDX1 | hsa-miR-2054 |
| FDX1 | hsa-miR-7201-5p |
| FDX1 | hsa-miR-548i |
| FDX1 | hsa-miR-335-5p |
| FDX1 | hsa-miR-4661-3p |
| FDX1 | hsa-miR-8071 |
| FDX1 | hsa-miR-216b |
| FDX1 | hsa-miR-2446 |
| FDX1 | hsa-miR-2285aa |
| FDX1 | hsa-miR-7172-3p |
| FDX1 | hsa-miR-105-5p |
| FDX1 | hsa-miR-3163 |
| FDX1 | hsa-let-7-3p |
| FDX1 | hsa-miR-6354 |
| FDX1 | hsa-miR-384-3p |
| FDX1 | hsa-miR-7180-5p |
| FDX1 | hsa-miR-7301-5p |
| FDX1 | hsa-miR-548c-3p |
| FDX1 | hsa-miR-4287 |
| FDX1 | hsa-miR-7393-5p |
| FDX1 | hsa-miR-4273 |
| FDX1 | hsa-miR-3182 |
| FDX1 | hsa-miR-101b-3p.2 |
| FDX1 | hsa-miR-365a-3p |
| FDX1 | hsa-miR-7686-5p |
| FDX1 | hsa-miR-4635 |
| FDX1 | hsa-miR-299-3p |
| FDX1 | hsa-miR-6772-5p |
| FDX1 | hsa-miR-4715-3p |
| FDX1 | hsa-miR-5709-3p |
| FDX1 | hsa-miR-485-5p |
| FDX1 | hsa-miR-623 |
| FDX1 | hsa-miR-380-3p |
| FDX1 | hsa-miR-432-5p |
| FDX1 | hsa-miR-7257-3p |
| FDX1 | hsa-miR-3199 |
| FDX1 | hsa-miR-1287-3p |
| FDX1 | hsa-miR-8075 |
| FDX1 | hsa-miR-609 |
| FDX1 | hsa-miR-502-5p |
| FDX1 | hsa-miR-890 |
| FDX1 | hsa-miR-1291 |
| FDX1 | hsa-miR-487a-3p |
| FDX1 | hsa-miR-7020-5p |
| FDX1 | hsa-miR-8074 |
| FDX1 | hsa-miR-6841-3p |
| FDX1 | hsa-miR-4259 |
| FDX1 | hsa-miR-214-3p |
| FDX1 | hsa-miR-15b-3p |
| FDX1 | hsa-miR-500-3p |
| FDX1 | hsa-miR-3938 |
| FDX1 | hsa-miR-455-5p |
| FDX1 | hsa-miR-222-3p |
| FDX1 | hsa-miR-7023-3p |
| FDX1 | hsa-miR-603 |
| FDX1 | hsa-miR-5187-3p |
| FDX1 | hsa-miR-590-3p |
| FDX1 | hsa-miR-4535 |
| FDX1 | hsa-miR-4649-3p |
| FDX1 | hsa-miR-616-5p |
| FDX1 | hsa-miR-550a-3p |
| FDX1 | hsa-miR-708-3p |
| FDX1 | hsa-miR-98-3p |
| FDX1 | hsa-miR-548a-5p |
| FDX1 | hsa-miR-3653-3p |
| FDX1 | hsa-miR-7641 |
| FDX1 | hsa-miR-570-5p |
| FDX1 | hsa-miR-4720-3p |
| FDX1 | hsa-miR-7191-5p |
| FDX1 | hsa-miR-2333 |
| FDX1 | hsa-miR-7445-5p |
| FDX1 | hsa-miR-3974 |
| FDX1 | hsa-miR-7319-5p |
| FDX1 | hsa-miR-741-5p |
| FDX1 | hsa-miR-3130-3p |
| FDX1 | hsa-miR-135a-1-3p |
| FDX1 | hsa-miR-7043-3p |
| FDX1 | hsa-miR-891a-5p |
| FDX1 | hsa-miR-4725-5p |
| FDX1 | hsa-miR-497-3p |
| FDX1 | hsa-miR-8067 |
| FDX1 | hsa-miR-6914-3p |
| FDX1 | hsa-miR-1290 |
| FDX1 | hsa-miR-16-1-3p |
| FDX1 | hsa-miR-711 |
| FDX1 | hsa-miR-4279 |
| FDX1 | hsa-miR-23-3p |
| FDX1 | hsa-miR-3154 |
| FDX1 | hsa-miR-634 |
| FDX1 | hsa-miR-3938-3p |
| FDX1 | hsa-miR-6314 |
| FDX1 | hsa-miR-6715b-3p |
| FDX1 | hsa-miR-8791 |
| FDX1 | hsa-miR-612 |
| FDX1 | hsa-miR-5189-5p |
| FDX1 | hsa-miR-7159-3p |
| FDX1 | hsa-miR-27b-3p |
| FDX1 | hsa-miR-101-3p.1 |
| FDX1 | hsa-miR-4729 |
| FDX1 | hsa-miR-1255-3p |
| FDX1 | hsa-miR-6762-5p |
| FDX1 | hsa-miR-892a |
| FDX1 | hsa-miR-4283 |
| FDX1 | hsa-miR-7068-3p |
| FDX1 | hsa-miR-155-5p |
| FDX1 | hsa-miR-3620-3p |
| FDX1 | hsa-miR-5119 |
| FDX1 | hsa-miR-6680-3p |
| FDX1 | hsa-miR-590-5p |
| FDX1 | hsa-miR-1545c-3p |
| FDX1 | hsa-miR-1246 |
| FDX1 | hsa-miR-1933-5p |
| FDX1 | hsa-miR-6855-3p |
| FDX1 | hsa-miR-96-5p |
| FDX1 | hsa-miR-2285af |
| FDX1 | hsa-miR-137-3p |
| FDX1 | hsa-miR-5697-5p |
| FDX1 | hsa-miR-922 |
| FDX1 | hsa-miR-676-5p |
| FDX1 | hsa-miR-3678-5p |
| FDX1 | hsa-miR-934 |
| FDX1 | hsa-miR-6748-3p |
| FDX1 | hsa-miR-2469 |
| FDX1 | hsa-miR-448-5p |
| FDX1 | hsa-miR-4703-3p |
| FDX1 | hsa-miR-606 |
| FDX1 | hsa-miR-6516-5p |
| FDX1 | hsa-miR-4755-3p |
| FDX1 | hsa-miR-2373-5p |
| FDX1 | hsa-miR-7041-3p |
| FDX1 | hsa-miR-6528 |
| FDX1 | hsa-miR-6737-5p |
| FDX1 | hsa-miR-6508-3p |
| FDX1 | hsa-miR-134-3p |
| FDX1 | hsa-miR-6122-5p |
| FDX1 | hsa-miR-6893-5p |
| FDX1 | hsa-miR-2052 |
| FDX1 | hsa-miR-449c-5p |
| FDX1 | hsa-miR-6809-3p |
| FDX1 | hsa-miR-195-3p |
| FDX1 | hsa-miR-1179 |
| FDX1 | hsa-miR-290 |
| FDX1 | hsa-miR-29b-2-5p |
| FDX1 | hsa-miR-4756-3p |
| FDX1 | hsa-miR-6766-5p |
| FDX1 | hsa-miR-1915-5p |
| FDX1 | hsa-miR-3596d |
| FDX1 | hsa-miR-545-3p |
| FDX1 | hsa-miR-17-3p |
| FDX1 | hsa-miR-872-3p |
| FDX1 | hsa-miR-548ba |
| FDX1 | hsa-miR-7194-3p |
| FDX1 | hsa-miR-155-3p |
| FDX1 | hsa-miR-499 |
| FDX1 | hsa-miR-290b-5p |
| FDX1 | hsa-miR-3942-3p |
| FDX1 | hsa-miR-7296-5p |
| FDX1 | hsa-miR-490-3p |
| FDX1 | hsa-miR-374-5p |
| FDX1 | hsa-miR-4478 |
| FDX1 | hsa-miR-592 |
| FDX1 | hsa-miR-548ai |
| FDX1 | hsa-miR-210 |
| FDX1 | hsa-miR-8789 |
| FDX1 | hsa-miR-7189-3p |
| FDX1 | hsa-miR-137 |
| FDX1 | hsa-miR-142-5p |
| FDX1 | hsa-miR-216 |
| FDX1 | hsa-miR-4752 |
| FDX1 | hsa-miR-3130-5p |
| FDX1 | hsa-miR-107 |
| FDX1 | hsa-miR-216b-5p |
| FDX1 | hsa-miR-2363 |
| FDX1 | hsa-miR-1546-5p |
| FDX1 | hsa-miR-6732-3p |
| FDX1 | hsa-miR-8884 |
| FDX1 | hsa-miR-20b-3p |
| FDX1 | hsa-miR-3084-5p |
| FDX1 | hsa-miR-203-3p |
| FDX1 | hsa-miR-6792-5p |
| FDX1 | hsa-miR-526 |
| FDX1 | hsa-miR-7188-3p |
| FDX1 | hsa-miR-297bc-3p |
| FDX1 | hsa-miR-133a-5p |
| FDX1 | hsa-miR-1842 |
| FDX1 | hsa-miR-8081 |
| FDX1 | hsa-miR-216-3p |
| FDX1 | hsa-miR-3116 |
| FDX1 | hsa-miR-3176 |
| FDX1 | hsa-miR-146a-3p |
| FDX1 | hsa-miR-223-5p |
| FDX1 | hsa-miR-6330 |
| FDX1 | hsa-miR-599-5p |
| FDX1 | hsa-miR-942-5p |
| FDX1 | hsa-miR-3190-5p |
| FDX1 | hsa-miR-4761-5p |
| FDX1 | hsa-miR-2439-3p |
| FDX1 | hsa-miR-3192-3p |
| FDX1 | hsa-miR-4690-3p |
| FDX1 | hsa-miR-487-5p |
| FDX1 | hsa-miR-1911-3p |
| FDX1 | hsa-miR-1236-5p |
| FDX1 | hsa-miR-144-3p |
| FDX1 | hsa-miR-507 |
| FDX1 | hsa-miR-331-5p |
| FDX1 | hsa-miR-2458 |
| FDX1 | hsa-miR-7047-3p |
| FDX1 | hsa-miR-4760-3p |
| FDX1 | hsa-miR-27 |
| FDX1 | hsa-miR-345-3p |
| FDX1 | hsa-miR-6851-3p |
| FDX1 | hsa-miR-5088-3p |
| FDX1 | hsa-miR-2284c |
| FDX1 | hsa-miR-4778-5p |
| FDX1 | hsa-miR-664 |
| FDX1 | hsa-miR-2483-3p |
| FDX1 | hsa-miR-5687 |
| FDX1 | hsa-let-7f-2-3p |
| FDX1 | hsa-miR-294 |
| FDX1 | hsa-miR-874 |
| FDX1 | hsa-miR-3972 |
| FDX1 | hsa-miR-7681-3p |
| FDX1 | hsa-miR-3613-3p |
| FDX1 | hsa-miR-1434-3p |
| FDX1 | hsa-miR-2303 |
| FDX1 | hsa-miR-1226-3p |
| FDX1 | hsa-miR-2285h |
| FDX1 | hsa-miR-7231-3p |
| FDX1 | hsa-miR-1256 |
| FDX1 | hsa-miR-338-3p |
| FDX1 | hsa-miR-7025-3p |
| FDX1 | hsa-miR-1185-3p |
| FDX1 | hsa-miR-1948-5p |
| FDX1 | hsa-miR-7199-5p |
| FDX1 | hsa-miR-519e-3p |
| FDX1 | hsa-miR-942 |
| FDX1 | hsa-miR-577-5p |
| FDX1 | hsa-miR-7048-3p |
| FDX1 | hsa-miR-6930-3p |
| FDX1 | hsa-miR-7398m-3p |
| FDX1 | hsa-miR-4639-3p |
| FDX1 | hsa-miR-7314-5p |
| FDX1 | hsa-miR-467-3p |
| FDX1 | hsa-miR-3529-3p |
| FDX1 | hsa-miR-6844 |
| FDX1 | hsa-miR-6913-3p |
| FDX1 | hsa-miR-4803 |
| FDX1 | hsa-miR-548ay-5p |
| FDX1 | hsa-miR-758-3p |
| FDX1 | hsa-miR-194-3p |
| FDX1 | hsa-miR-7213-5p |
| FDX1 | hsa-miR-6881-3p |
| FDX1 | hsa-miR-4460 |
| FDX1 | hsa-miR-5585-5p |
| FDX1 | hsa-miR-2284n |
| FDX1 | hsa-miR-6833-5p |
| FDX1 | hsa-miR-130a-5p |
| FDX1 | hsa-miR-4645-5p |
| FDX1 | hsa-miR-1277-5p |
| FDX1 | hsa-miR-7012-3p |
| FDX1 | hsa-miR-6862-3p |
| FDX1 | hsa-miR-6377 |
| FDX1 | hsa-miR-5189-3p |
| FDX1 | hsa-miR-7111-3p |
| FDX1 | hsa-miR-499b-5p |
| FDX1 | hsa-miR-499a-3p |
| FDX1 | hsa-miR-592-5p |
| FDX1 | hsa-miR-3064-3p |
| FDX1 | hsa-miR-409a |
| FDX1 | hsa-miR-2362 |
| FDX1 | hsa-miR-329-5p |
| FDX1 | hsa-miR-1827 |
| FDX1 | hsa-miR-2284v |
| FDX1 | hsa-miR-4753-5p |
| FDX1 | hsa-miR-548w |
| FDX1 | hsa-miR-7106-5p |
| FDX1 | hsa-miR-6821-5p |
| FDX1 | hsa-miR-28-5p |
| FDX1 | hsa-miR-4311 |
| FDX1 | hsa-miR-6319 |
| FDX1 | hsa-miR-221-3p |
| FDX1 | hsa-miR-632 |
| FDX1 | hsa-miR-103-5p |
| FDX1 | hsa-miR-551b-5p |
| FDX1 | hsa-miR-5192 |
| FDX1 | hsa-miR-369-3p |
| FDX1 | hsa-miR-653-5p |
| FDX1 | hsa-miR-4482-3p |
| FDX1 | hsa-miR-3170 |
| FDX1 | hsa-miR-18a-3p |
| FDX1 | hsa-miR-510 |
| FDX1 | hsa-miR-5087 |
| FDX1 | hsa-miR-376b-3p |
| FDX1 | hsa-miR-4261 |
| FDX1 | hsa-miR-7210-3p |
| FDX1 | hsa-miR-7351-5p |
| FDX1 | hsa-miR-9-5p |
| FDX1 | hsa-miR-6793-3p |
| FDX1 | hsa-miR-29a-3p |
| FDX1 | hsa-miR-6887-3p |
| FDX1 | hsa-miR-548ah-3p |
| FDX1 | hsa-miR-8095 |
| FDX1 | hsa-miR-95-5p |
| FDX1 | hsa-miR-371b-5p |
| FDX1 | hsa-miR-888-5p |
| FDX1 | hsa-miR-145-5p |
| FDX1 | hsa-miR-300 |
| FDX1 | hsa-miR-517 |
| FDX1 | hsa-miR-684 |
| FDX1 | hsa-miR-6799-5p |
| FDX1 | hsa-miR-654-5p |
| FDX1 | hsa-miR-3165 |
| FDX1 | hsa-miR-513a-3p |
| FDX1 | hsa-miR-4668-3p |
| FDX1 | hsa-miR-8869 |
| FDX1 | hsa-miR-7650-3p |
| FDX1 | hsa-miR-8842 |
| FDX1 | hsa-miR-501-5p |
| FDX1 | hsa-miR-6811-3p |
| FDX1 | hsa-miR-7007-3p |
| FDX1 | hsa-miR-324-3p |
| FDX1 | hsa-miR-409 |
| FDX1 | hsa-miR-542-3p |
| FDX1 | hsa-miR-1575 |
| FDX1 | hsa-miR-7178-3p |
| FDX1 | hsa-miR-4533 |
| FDX1 | hsa-miR-379-5p |
| FDX1 | hsa-miR-216b-3p |
| FDX1 | hsa-miR-6742-3p |
| FDX1 | hsa-miR-505-3p |
| FDX1 | hsa-miR-3672 |
| FDX1 | hsa-miR-937-5p |
| FDX1 | hsa-miR-290a-5p |
| FDX1 | hsa-miR-4260 |
| FDX1 | hsa-miR-27b-5p |
| FDX1 | hsa-miR-548aj-5p |
| FDX1 | hsa-miR-576-5p |
| FDX1 | hsa-miR-199-5p |
| FDX1 | hsa-miR-544-5p |
| FDX1 | hsa-miR-4679 |
| FDX1 | hsa-miR-6860 |
| FDX1 | hsa-miR-761 |
| FDX1 | hsa-miR-2368-3p |
| FDX1 | hsa-miR-6898-5p |
| FDX1 | hsa-miR-451b |
| FDX1 | hsa-miR-1910-3p |
| FDX1 | hsa-miR-543-3p |
| FDX1 | hsa-miR-1248 |
| FDX1 | hsa-miR-1306-5p |
| FDX1 | hsa-miR-3473g |
| FDX1 | hsa-miR-6885-3p |
| FDX1 | hsa-miR-135-5p |
| FDX1 | hsa-miR-1271-3p |
| FDX1 | hsa-miR-449a |
| FDX1 | hsa-miR-4253 |
| FDX1 | hsa-miR-573 |
| FDX1 | hsa-miR-2351 |
| FDX1 | hsa-miR-549-5p |
| FDX1 | hsa-miR-7216-3p |
| FDX1 | hsa-miR-597-3p |
| FDX1 | hsa-miR-6924-3p |
| FDX1 | hsa-miR-29a-5p |
| FDX1 | hsa-miR-3177-5p |
| FDX1 | hsa-miR-7153-3p |
| FDX1 | hsa-miR-190a-3p |
| FDX1 | hsa-miR-181b-1-3p |
| FDX1 | hsa-miR-483-3p.2 |
| FDX1 | hsa-miR-7027-5p |
| FDX1 | hsa-miR-200bc |
| FDX1 | hsa-miR-5125 |
| FDX1 | hsa-miR-873-3p |
| FDX1 | hsa-miR-4666a-3p |
| FDX1 | hsa-miR-7013-3p |
| FDX1 | hsa-miR-153-3p |
| FDX1 | hsa-miR-203a-5p |
| FDX1 | hsa-miR-16-3p |
| FDX1 | hsa-miR-4735-5p |
| FDX1 | hsa-miR-2393 |
| FDX1 | hsa-miR-7154-5p |
| FDX1 | hsa-miR-1233-3p |
| FDX1 | hsa-miR-7302-3p |
| FDX1 | hsa-miR-8821 |
| FDX1 | hsa-miR-557 |
| FDX1 | hsa-miR-7203-5p |
| FDX1 | hsa-miR-3122 |
| FDX1 | hsa-miR-6735-3p |
| FDX1 | hsa-miR-133 |
| FDX1 | hsa-miR-2285n |
| FDX1 | hsa-miR-501-3p |
| FDX1 | hsa-miR-3192-5p |
| FDX1 | hsa-miR-298 |
| FDX1 | hsa-miR-7387-5p |
| FDX1 | hsa-miR-3605-5p |
| FDX1 | hsa-miR-3691-3p |
| FDX1 | hsa-miR-297 |
| FDX1 | hsa-miR-204-5p |
| FDX1 | hsa-miR-362-3p |
| FDX1 | hsa-miR-376c |
| FDX1 | hsa-miR-7578 |
| FDX1 | hsa-miR-300-5p |
| FDX1 | hsa-miR-129-5p |
| FDX1 | hsa-miR-1262-3p |
| FDX1 | hsa-miR-4640-5p |
| FDX1 | hsa-miR-1284 |
| FDX1 | hsa-miR-1305 |
| FDX1 | hsa-miR-292a-5p |
| FDX1 | hsa-miR-6795-3p |
| FDX1 | hsa-miR-2284z |
| FDX1 | hsa-miR-548c-5p |
| FDX1 | hsa-miR-3189-5p |
| FDX1 | hsa-miR-579-3p |
| FDX1 | hsa-miR-1251-3p |
| FDX1 | hsa-miR-148a-5p |
| FDX1 | hsa-miR-382-3p |
| FDX1 | hsa-miR-8807 |
| FDX1 | hsa-miR-6888-5p |
| FDX1 | hsa-miR-539-3p |
| FDX1 | hsa-miR-518c-5p |
| FDX1 | hsa-miR-126-5p |
| FDX1 | hsa-miR-200c-5p |
| FDX1 | hsa-miR-197-3p |
| FDX1 | hsa-miR-7162-3p |
| FDX1 | hsa-miR-7383-5p |
| FDX1 | hsa-miR-541-5p |
| FDX1 | hsa-miR-372-5p |
| FDX1 | hsa-miR-7263-3p |
| FDX1 | hsa-miR-4672 |
| FDX1 | hsa-miR-411-5p |
| FDX1 | hsa-miR-141-3p |
| FDX1 | hsa-miR-7252-3p |
| FDX1 | hsa-miR-3692-3p |
| FDX1 | hsa-miR-3119 |
| FDX1 | hsa-miR-548ae-3p |
| FDX1 | hsa-miR-431-5p |
| FDX1 | hsa-miR-548o-5p |
| FDX1 | hsa-miR-7171-3p |
| FDX1 | hsa-miR-132-5p |
| FDX1 | hsa-miR-153 |
| FDX1 | hsa-miR-346 |
| FDX1 | hsa-miR-7157-5p |
| FDX1 | hsa-miR-1202 |
| FDX1 | hsa-miR-410-3p |
| FDX1 | hsa-miR-6807-5p |
| FDX1 | hsa-miR-325 |
| FDX1 | hsa-miR-6529-3p |
| FDX1 | hsa-miR-7218-3p |
| FDX1 | hsa-miR-766-3p |
| FDX1 | hsa-miR-3551-3p |
| FDX1 | hsa-miR-4790-3p |
| FDX1 | hsa-miR-4524a-5p |
| FDX1 | hsa-miR-559 |
| FDX1 | hsa-miR-31-3p |
| FDX1 | hsa-miR-563 |
| FDX1 | hsa-miR-4518 |
| FDX1 | hsa-miR-467f |
| FDX1 | hsa-miR-548aq-3p |
| FDX1 | hsa-miR-548s |
| FDX1 | hsa-miR-93-5p |
| FDX1 | hsa-miR-489-3p |
| FDX1 | hsa-miR-29a-1-5p |
| FDX1 | hsa-miR-561-3p |
| FDX1 | hsa-miR-7195-3p |
| FDX1 | hsa-miR-2334 |
| FDX1 | hsa-miR-3970 |
| FDX1 | hsa-miR-4662-5p |
| FDX1 | hsa-miR-6720-5p |
| FDX1 | hsa-miR-883-3p |
| FDX1 | hsa-miR-3470b |
| FDX1 | hsa-miR-105-5p |
| FDX1 | hsa-miR-18 |
| FDX1 | hsa-miR-2352 |
| FDX1 | hsa-miR-1224-3p |
| FDX1 | hsa-miR-3617-3p |
| FDX1 | hsa-miR-409-3p |
| FDX1 | hsa-miR-1243-5p |
| FDX1 | hsa-miR-218-5p |
| FDX1 | hsa-miR-125a-3p |
| FDX1 | hsa-miR-4795-5p |
| FDX1 | hsa-miR-151-3p |
| FDX1 | hsa-miR-4316 |
| FDX1 | hsa-miR-6768-5p |
| FDX1 | hsa-miR-583 |
| FDX1 | hsa-miR-204-3p |
| FDX1 | hsa-miR-7673-5p |
| FDX1 | hsa-miR-3978 |
| FDX1 | hsa-miR-5682 |
| FDX1 | hsa-miR-579 |
| FDX1 | hsa-miR-1252-3p |
| FDX1 | hsa-miR-6715a-3p |
| FDX1 | hsa-miR-4418 |
| FDX1 | hsa-miR-4731-3p |
| FDX1 | hsa-miR-6508-5p |
| FDX1 | hsa-miR-5692bc |
| FDX1 | hsa-miR-668-3p |
| FDX1 | hsa-miR-4277 |
| FDX1 | hsa-miR-2284-5p |
| FDX1 | hsa-miR-7239-5p |
| FDX1 | hsa-miR-27a-3p |
| FDX1 | hsa-miR-34 |
| FDX1 | hsa-miR-378-5p |
| FDX1 | hsa-miR-2284m |
| FDX1 | hsa-miR-6322 |
| FDX1 | hsa-miR-4452 |
| FDX1 | hsa-miR-149-3p |
| FDX1 | hsa-miR-6859-3p |
| FDX1 | hsa-miR-659-5p |
| FDX1 | hsa-miR-568 |
| FDX1 | hsa-miR-7371f-5p |
| FDX1 | hsa-miR-125-5p |
| FDX1 | hsa-miR-429 |
| FDX1 | hsa-miR-6872-5p |
| FDX1 | hsa-miR-3942-5p |
| FDX1 | hsa-miR-7156-3p |
| FDX1 | hsa-miR-471-3p |
| FDX1 | hsa-miR-6773-3p |
| FDX1 | hsa-miR-466g |
| FDX1 | hsa-miR-369-3p |
| FDX1 | hsa-miR-455-3p |
| FDX1 | hsa-miR-124-5p |
| FDX1 | hsa-miR-7208-5p |
| FDX1 | hsa-miR-6315 |
| FDX1 | hsa-miR-140-3p.1 |
| FDX1 | hsa-miR-655 |
| FDX1 | hsa-miR-34b-5p |
| FDX1 | hsa-miR-758-5p |
| FDX1 | hsa-miR-466f-3p |
| FDX1 | hsa-miR-4307 |
| FDX1 | hsa-miR-4786-3p |
| FDX1 | hsa-miR-7300-5p |
| FDX1 | hsa-miR-3617-5p |
| FDX1 | hsa-miR-101a-3p.2 |
| FDX1 | hsa-miR-877-3p |
| FDX1 | hsa-miR-548ap-5p |
| FDX1 | hsa-miR-223-3p |
| FDX1 | hsa-miR-6845-5p |
| FDX1 | hsa-miR-7665-5p |
| FDX1 | hsa-miR-513b-3p |
| FDX1 | hsa-miR-3912-5p |
| FDX1 | hsa-miR-509-5p |
| FDX1 | hsa-miR-1296 |
| FDX1 | hsa-miR-7289-5p |
| FDX1 | hsa-miR-587 |
| FDX1 | hsa-miR-106a-3p |
| FDX1 | hsa-miR-871-5p |
| FDX1 | hsa-miR-3670 |
| FDX1 | hsa-miR-6969-5p |
| FDX1 | hsa-miR-936 |
| FDX1 | hsa-miR-3145-3p |
| FDX1 | hsa-miR-671-5p |
| FDX1 | hsa-miR-3958 |
| FDX1 | hsa-miR-4469 |
| FDX1 | hsa-miR-713 |
| FDX1 | hsa-miR-4680-5p |
| FDX1 | hsa-miR-1197-3p |
| FDX1 | hsa-miR-888-3p |
| FDX1 | hsa-miR-7333-3p |
| FDX1 | hsa-miR-548aa |
| FDX1 | hsa-miR-6852-5p |
| FDX1 | hsa-miR-141-3p |
| FDX1 | hsa-miR-1283a |
| FDX1 | hsa-miR-19b-5p |
| FDX1 | hsa-miR-548i-3p |
| FDX1 | hsa-miR-7974 |
| FDX1 | hsa-miR-575 |
| FDX1 | hsa-miR-2392 |
| FDX1 | hsa-miR-524-5p |
| FDX1 | hsa-miR-1343 |
| FDX1 | hsa-miR-4765 |
| FDX1 | hsa-miR-7211-3p |
| FDX1 | hsa-miR-6417 |
| FDX1 | hsa-miR-6984-3p |
| FDX1 | hsa-miR-630 |
| FDX1 | hsa-miR-1226-3p |
| FDX1 | hsa-miR-548k |
| FDX1 | hsa-miR-7059-3p |
| FDX1 | hsa-miR-618 |
| FDX1 | hsa-miR-3682-3p |
| FDX1 | hsa-miR-4781-3p |
| FDX1 | hsa-miR-4528 |
| FDX1 | hsa-miR-371a-5p |
| FDX1 | hsa-miR-8908-3p |
| FDX1 | hsa-miR-888-5p |
| FDX1 | hsa-miR-96-5p |
| FDX1 | hsa-miR-7-5p |
| FDX1 | hsa-miR-7394a-5p |
| FDX1 | hsa-miR-186-5p |
| FDX1 | hsa-miR-5696 |
| FDX1 | hsa-miR-802-5p |
| FDX1 | hsa-miR-4643 |
| FDX1 | hsa-miR-211-5p |
| FDX1 | hsa-miR-93-3p |
| FDX1 | hsa-miR-129-2-3p |
| FDX1 | hsa-miR-553 |
| FDX1 | hsa-miR-100-3p |
| FDX1 | hsa-miR-425-5p |
| FDX1 | hsa-miR-6864-3p |
| FDX1 | hsa-miR-4661-5p |
| FDX1 | hsa-miR-4427 |
| FDX1 | hsa-miR-875-3p |
| FDX1 | hsa-miR-7340-5p |
| FDX1 | hsa-miR-5579-3p |
| FDX1 | hsa-miR-1260a |
| FDX1 | hsa-miR-8052 |
| FDX1 | hsa-miR-6874-3p |
| FDX1 | hsa-miR-692 |
| FDX1 | hsa-miR-3121-3p |
| FDX1 | hsa-miR-6784-3p |
| FDX1 | hsa-miR-651 |
| FDX1 | hsa-miR-6785-5p |
| FDX1 | hsa-miR-710 |
| FDX1 | hsa-miR-666-3p |
| FDX1 | hsa-miR-1643a-5p |
| FDX1 | hsa-miR-3571 |
| FDX1 | hsa-miR-4691-5p |
| FDX1 | hsa-miR-6883-5p |
| FDX1 | hsa-miR-940 |
| FDX1 | hsa-miR-3657 |
| FDX1 | hsa-miR-499-5p |
| FDX1 | hsa-miR-4693-5p |
| FDX1 | hsa-miR-590-5p |
| FDX1 | hsa-miR-6958-3p |
| FDX1 | hsa-miR-488-3p |
| FDX1 | hsa-miR-3123 |
| FDX1 | hsa-miR-4290 |
| FDX1 | hsa-miR-548b-5p |
| FDX1 | hsa-miR-7065-5p |
| FDX1 | hsa-miR-693-5p |
| FDX1 | hsa-miR-4263 |
| FDX1 | hsa-miR-4799-5p |
| FDX1 | hsa-miR-381-3p |
| FDX1 | hsa-miR-302b-5p |
| FDX1 | hsa-miR-6909-3p |
| FDX1 | hsa-miR-7192-3p |
| FDX1 | hsa-miR-548am-5p |
| FDX1 | hsa-miR-145-5p |
| FDX1 | hsa-miR-5123 |
| FDX1 | hsa-miR-101b-3p.1 |
| FDX1 | hsa-miR-2887 |
| FDX1 | hsa-miR-6880-5p |
| FDX1 | hsa-miR-1295b-3p |
| FDX1 | hsa-miR-6976-5p |
| FDX1 | hsa-miR-1643b-5p |
| FDX1 | hsa-miR-361-5p |
| FDX1 | hsa-miR-21-5p |
| FDX1 | hsa-miR-2285j |
| FDX1 | hsa-miR-6855-5p |
| FDX1 | hsa-miR-7398l-3p |
| FDX1 | hsa-miR-384 |
| FDX1 | hsa-miR-5689 |
| FDX1 | hsa-miR-3064-5p |
| FDX1 | hsa-miR-411-3p |
| FDX1 | hsa-miR-3085 |
| FDX1 | hsa-miR-4680-3p |
| FDX1 | hsa-miR-7-3p |
| FDX1 | hsa-miR-6802-3p |
| FDX1 | hsa-miR-3533 |
| FDX1 | hsa-miR-4483 |
| FDX1 | hsa-miR-5583-3p |
| FDX1 | hsa-miR-7270-5p |
| FDX1 | hsa-miR-7207-3p |
| FDX1 | hsa-miR-2278 |
| FDX1 | hsa-miR-4717-3p |
| FDX1 | hsa-miR-3544-5p |
| FDX1 | hsa-miR-10b-3p |
| FDX1 | hsa-miR-3945 |
| FDX1 | hsa-miR-4699-5p |
| FDX1 | hsa-miR-6875-3p |
| FDX1 | hsa-miR-509-3-5p |
| FDX1 | hsa-miR-3101-3p |
| FDX1 | hsa-miR-376c-3p |
| FDX1 | hsa-miR-628-3p |
| FDX1 | hsa-miR-5096 |
| FDX1 | hsa-miR-293-5p |
| FDX1 | hsa-miR-188-5p |
| FDX1 | hsa-miR-6862-5p |
| FDX1 | hsa-miR-3653-5p |
| FDX1 | hsa-miR-455-5p |
| FDX1 | hsa-miR-30a-3p |
| FDX1 | hsa-miR-7181-5p |
| FDX1 | hsa-miR-6812-5p |
| FDX1 | hsa-miR-5581-3p |
| FDX1 | hsa-miR-876-3p |
| FDX1 | hsa-miR-143-5p |
| FDX1 | hsa-miR-6071 |
| FDX1 | hsa-miR-185-3p |
| FDX1 | hsa-miR-513c-3p |
| FDX1 | hsa-miR-660-3p |
| FDX1 | hsa-miR-3616-5p |
| FDX1 | hsa-miR-6513-5p |
| FDX1 | hsa-miR-4524b-3p |
| FDX1 | hsa-miR-148b-5p |
| FDX1 | hsa-miR-3180-5p |
| FDX1 | hsa-miR-199-3p |
| FDX1 | hsa-miR-3680-3p |
| FDX1 | hsa-miR-7119-3p |
| FDX1 | hsa-miR-141-5p |
| FDX1 | hsa-miR-1-5p |
| FDX1 | hsa-miR-548ae-5p |
| FDX1 | hsa-miR-5691 |
| FDX1 | hsa-miR-10a-3p |
| FDX1 | hsa-miR-149-5p |
| FDX1 | hsa-miR-15-5p |
| FDX1 | hsa-miR-875 |
| FDX1 | hsa-miR-1273h-5p |
| FDX1 | hsa-miR-493-3p |
| FDX1 | hsa-miR-6849-3p |
| FDX1 | hsa-miR-548at-5p |
| FDX1 | hsa-miR-4310 |
| FDX1 | hsa-miR-2285z |
| FDX1 | hsa-miR-204-5p |
| FDX1 | hsa-miR-743-5p |
| FDX1 | hsa-miR-7680-5p |
| FDX1 | hsa-miR-3085-3p |
| FDX1 | hsa-miR-6792-3p |
| FDX1 | hsa-miR-188-5p |
| FDX1 | hsa-miR-4789-3p |
| FDX1 | hsa-miR-6780b-3p |
| FDX1 | hsa-miR-190-5p |
| FDX1 | hsa-miR-381-3p |
| FDX1 | hsa-miR-1323-3p |
| FDX1 | hsa-miR-548t-3p |
| FDX1 | hsa-miR-7664-3p |
| FDX1 | hsa-miR-2284i |
| FDX1 | hsa-miR-344b-3p |
| FDX1 | hsa-miR-548j-5p |
| FDX1 | hsa-miR-344-3p |
| FDX1 | hsa-miR-5585-3p |
| FDX1 | hsa-miR-4796-5p |
| FDX1 | hsa-miR-203-5p |
| FDX1 | hsa-miR-8863 |
| FDX1 | hsa-miR-3470a |
| FDX1 | hsa-miR-6131 |
| FDX1 | hsa-miR-365b-5p |
| FDX1 | hsa-miR-449b-3p |
| FDX1 | hsa-miR-511-5p |
| FDX1 | hsa-miR-7297-5p |
| FDX1 | hsa-miR-6910-3p |
| FDX1 | hsa-miR-515-5p |
| FDX1 | hsa-miR-5695 |
| FDX1 | hsa-miR-329-3p |
| FDX1 | hsa-miR-4417 |
| FDX1 | hsa-miR-4670-3p |
| FDX1 | hsa-miR-4795-3p |
| FDX1 | hsa-miR-548d-5p |
| FDX1 | hsa-miR-512-3p |
| FDX1 | hsa-miR-5692a |
| FDX1 | hsa-miR-201-5p |
| FDX1 | hsa-miR-6768-3p |
| FDX1 | hsa-miR-1185-5p |
| FDX1 | hsa-miR-122-5p |
| FDX1 | hsa-miR-4328 |
| FDX1 | hsa-miR-450a-1-3p |
| FDX1 | hsa-miR-208a-3p |
| FDX1 | hsa-miR-7221-3p |
| FDX1 | hsa-miR-6959-5p |
| FDX1 | hsa-miR-6775-3p |
| FDX1 | hsa-miR-1912-3p |
| FDX1 | hsa-miR-10-5p |
| FDX1 | hsa-miR-674-5p |
| FDX1 | hsa-miR-694 |
| FDX1 | hsa-miR-326-3p |
| FDX1 | hsa-miR-659-3p |
| FDX1 | hsa-miR-330-3p |
| FDX1 | hsa-miR-6344 |
| FDX1 | hsa-miR-3104-5p |
| FDX1 | hsa-miR-3071-3p |
| FDX1 | hsa-miR-7322-3p |
| FDX1 | hsa-miR-16-5p |
| FDX1 | hsa-miR-889 |
| FDX1 | hsa-miR-2285 |
| FDX1 | hsa-miR-373-5p |
| FDX1 | hsa-miR-548am-3p |
| FDX1 | hsa-miR-1298 |
| FDX1 | hsa-miR-2372 |
| FDX1 | hsa-miR-1258-3p |
| FDX1 | hsa-miR-574-5p |
| FDX1 | hsa-miR-147a |
| FDX1 | hsa-miR-4740-5p |
| FDX1 | hsa-miR-548c-3p |
| FDX1 | hsa-miR-2376 |
| FDX1 | hsa-miR-873a-5p.2 |
| FDX1 | hsa-miR-580-5p |
| FDX1 | hsa-miR-686 |
| FDX1 | hsa-miR-3140-3p |
| FDX1 | hsa-miR-449c-3p |
| FDX1 | hsa-miR-6740-3p |
| FDX1 | hsa-miR-7174-5p |
| FDX1 | hsa-miR-935 |
| FDX1 | hsa-miR-1184 |
| FDX1 | hsa-miR-708-5p |
| FDX1 | hsa-miR-2284k |
| FDX1 | hsa-miR-582-3p |
| FDX1 | hsa-miR-455-3p.2 |
| FDX1 | hsa-miR-147-5p |
| FDX1 | hsa-miR-872-5p |
| FDX1 | hsa-miR-2329-3p |
| FDX1 | hsa-miR-18b-3p |
| FDX1 | hsa-miR-1230 |
| FDX1 | hsa-miR-6750-3p |
| FDX1 | hsa-miR-5694 |
| FDX1 | hsa-miR-4536-5p |
| FDX1 | hsa-miR-30-5p |
| FDX1 | hsa-miR-548aq-5p |
| FDX1 | hsa-miR-4650 |
| FDX1 | hsa-miR-7658-5p |
| FDX1 | hsa-miR-548ay-3p |
| FDX1 | hsa-miR-627 |
| FDX1 | hsa-miR-215-3p |
| FDX1 | hsa-miR-7026-3p |
| FDX1 | hsa-miR-5190 |
| FDX1 | hsa-miR-6870-3p |
| FDX1 | hsa-miR-3074-2-3p |
| FDX1 | hsa-miR-222-3p |
| FDX1 | hsa-miR-22-5p |
| FDX1 | hsa-miR-130b-5p |
| FDX1 | hsa-miR-466b-2-3p |
| FDX1 | hsa-miR-3110-5p |
| FDX1 | hsa-miR-18-3p |
| FDX1 | hsa-miR-5589-3p |
| FDX1 | hsa-miR-7347-5p |
| FDX1 | hsa-miR-4282 |
| FDX1 | hsa-miR-181b-2-3p |
| FDX1 | hsa-miR-7166-3p |
| FDX1 | hsa-miR-514a-5p |
| FDX1 | hsa-miR-4797-5p |
| FDX1 | hsa-miR-103 |
| FDX1 | hsa-miR-196 |
| FDX1 | hsa-miR-633 |
| FDX1 | hsa-miR-939-3p |
| FDX1 | hsa-miR-3152-3p |
| FDX1 | hsa-miR-4495 |
| FDX1 | hsa-miR-7330-3p |
| FDX1 | hsa-miR-6769a-5p |
| FDX1 | hsa-miR-7398q-3p |
| FDX1 | hsa-miR-5112 |
| FDX1 | hsa-miR-4999-5p |
| FDX1 | hsa-miR-5684 |
| FDX1 | hsa-miR-6886-3p |
| FDX1 | hsa-miR-466b-4-3p |
| FDX1 | hsa-miR-741-3p |
| FDX1 | hsa-miR-4727-5p |
| FDX1 | hsa-miR-101-5p |
| FDX1 | hsa-miR-1178-3p |
| FDX1 | hsa-miR-468-3p |
| FDX1 | hsa-miR-664a-3p |
| FDX1 | hsa-let-7a-3p |
| FDX1 | hsa-miR-566 |
| FDX1 | hsa-miR-2320-5p |
| FDX1 | hsa-let-7f-1-3p |
| FDX1 | hsa-miR-3907 |
| FDX1 | hsa-miR-7349-3p |
| FDX1 | hsa-miR-376a-5p |
| FDX1 | hsa-miR-4503 |
| FDX1 | hsa-miR-148-5p |
| FDX1 | hsa-miR-212-3p |
| FDX1 | hsa-miR-3529-5p |
| FDX1 | hsa-miR-489-5p |
| FDX1 | hsa-miR-206-5p |
| FDX1 | hsa-miR-743a-3p |
| FDX1 | hsa-miR-30 |
| FDX1 | hsa-miR-3572-3p |
| FDX1 | hsa-miR-3915 |
| FDX1 | hsa-miR-652-3p |
| FDX1 | hsa-miR-5003-3p |
| FDX1 | hsa-miR-4524-5p |
| FDX1 | hsa-miR-548au-5p |
| FDX1 | hsa-miR-6502-3p |
| FDX1 | hsa-miR-2902 |
| FDX1 | hsa-miR-7396-5p |
| FDX1 | hsa-miR-365-5p |
| FDX1 | hsa-miR-670-3p |
| FDX1 | hsa-miR-519 |
| FDX1 | hsa-miR-2366 |
| FDX1 | hsa-miR-1208 |
| FDX1 | hsa-miR-2304 |
| FDX1 | hsa-miR-548 |
| FDX1 | hsa-miR-7a-1-3p |
| FDX1 | hsa-miR-1620 |
| FDX1 | hsa-miR-4477a |
| FDX1 | hsa-miR-1972 |
| FDX1 | hsa-miR-3557-3p |
| FDX1 | hsa-miR-3059-5p |
| FDX1 | hsa-miR-429-3p |
| FDX1 | hsa-miR-3074-3p |
| FDX1 | hsa-miR-1836 |
| FDX1 | hsa-miR-6756-5p |
| FDX1 | hsa-miR-7228-3p |
| FDX1 | hsa-miR-582-5p |
| FDX1 | hsa-miR-6324 |
| FDX1 | hsa-miR-140-5p |
| FDX1 | hsa-miR-3606-3p |
| FDX1 | hsa-miR-494-3p |
| FDX1 | hsa-miR-33-3p |
| FDX1 | hsa-miR-4284 |
| FDX1 | hsa-miR-5197-5p |
| FDX1 | hsa-miR-3934-3p |
| FDX1 | hsa-miR-7854-3p |
| FDX1 | hsa-miR-6950-3p |
| FDX1 | hsa-miR-548x-5p |
| FDX1 | hsa-miR-370 |
| FDX1 | hsa-miR-3058-5p |
| FDX1 | hsa-miR-7294-3p |
| FDX1 | hsa-miR-4727-3p |
| FDX1 | hsa-miR-7054-3p |
| FDX1 | hsa-miR-512-5p |
| FDX1 | hsa-miR-152-5p |
| FDX1 | hsa-miR-214-5p |
| FDX1 | hsa-miR-1323 |
| FDX1 | hsa-miR-3689-3p |
| FDX1 | hsa-miR-2365 |
| FDX1 | hsa-miR-4776-5p |
| FDX1 | hsa-miR-6861-3p |
| FDX1 | hsa-miR-7386g-3p |
| FDX1 | hsa-miR-30c-3p |
| FDX1 | hsa-miR-2285x |
| FDX1 | hsa-miR-629-3p |
| FDX1 | hsa-miR-3577-5p |
| FDX1 | hsa-miR-4673 |
| FDX1 | hsa-miR-378-3p |
| FDX1 | hsa-miR-1982-3p |
| FDX1 | hsa-miR-8073 |
| FDX1 | hsa-miR-691 |
| FDX1 | hsa-miR-519e-5p |
| FDX1 | hsa-miR-584-3p |
| FDX1 | hsa-miR-6083 |
| FDX1 | hsa-miR-4677-5p |
| FDX1 | hsa-miR-548f-5p |
| FDX1 | hsa-miR-29-5p |
| FDX1 | hsa-miR-764 |
| FDX1 | hsa-miR-548ag |
| FDX1 | hsa-miR-465-5p |
| FDX1 | hsa-miR-8485 |
| FDX1 | hsa-miR-4676-5p |
| FDX1 | hsa-miR-483-3p |
| FDX1 | hsa-miR-6523b |
| FDX1 | hsa-miR-217 |
| FDX1 | hsa-miR-2404 |
| FDX1 | hsa-miR-450b |
| FDX1 | hsa-miR-370-3p |
| FDX1 | hsa-miR-760-3p |
| FDX1 | hsa-miR-7164-5p |
| FDX1 | hsa-miR-6828-3p |
| FDX1 | hsa-miR-23a-5p |
| FDX1 | hsa-miR-548m |
| FDX1 | hsa-miR-5697-3p |
| FDX1 | hsa-miR-567 |
| FDX1 | hsa-miR-668 |
| FDX1 | hsa-miR-802 |
| FDX1 | hsa-miR-125 |
| FDX1 | hsa-miR-4539 |
| FDX1 | hsa-miR-20-3p |
| FDX1 | hsa-miR-5000-3p |
| FDX1 | hsa-miR-607 |
| FDX1 | hsa-miR-7398r-3p |
| FDX1 | hsa-miR-101-3p |
| FDX1 | hsa-miR-1197 |
| FDX1 | hsa-miR-3187-5p |
| FDX1 | hsa-miR-6777-3p |
| FDX1 | hsa-miR-9-3-3p |
| FDX1 | hsa-miR-4524a-3p |
| FDX1 | hsa-miR-6814-3p |
| FDX1 | hsa-miR-7201-3p |
| FDX1 | hsa-miR-551-5p |
| FDX1 | hsa-miR-489 |
| FDX1 | hsa-miR-652-3p |
| FDX1 | hsa-miR-622 |
| FDX1 | hsa-miR-3580-5p |
| FDX1 | hsa-miR-182-5p |
| FDX1 | hsa-miR-873-5p.1 |
| FDX1 | hsa-miR-7847-3p |
| FDX1 | hsa-miR-7684-3p |
| FDX1 | hsa-miR-101-2-5p |
| FDX1 | hsa-miR-1912 |
| FDX1 | hsa-miR-2368-5p |
| FDX1 | hsa-miR-216a |
| FDX1 | hsa-miR-101-1-5p |
| FDX1 | hsa-miR-4425 |
| FDX1 | hsa-miR-582-5p |
| FDX1 | hsa-miR-769 |
| FDX1 | hsa-miR-3157-3p |
| FDX1 | hsa-miR-5588-5p |
| FDX1 | hsa-miR-412 |
| FDX1 | hsa-miR-466ade-3p |
| FDX1 | hsa-miR-627-5p |
| FDX1 | hsa-miR-1236-3p |
| FDX1 | hsa-miR-6769b-3p |
| FDX1 | hsa-miR-452-3p |
| FDX1 | hsa-miR-186-5p |
| FDX1 | hsa-miR-4639-5p |
| FDX1 | hsa-miR-1263 |
| FDX1 | hsa-miR-7360-3p |
| FDX1 | hsa-miR-3121-5p |
| FDX1 | hsa-miR-106 |
| FDX1 | hsa-miR-467g |
| FDX1 | hsa-miR-2482 |
| FDX1 | hsa-miR-3913-5p |
| FDX1 | hsa-miR-7008-3p |
| FDX1 | hsa-miR-344f-3p |
| FDX1 | hsa-miR-126b-5p |
| FDX1 | hsa-miR-1929-3p |
| FDX1 | hsa-miR-4428 |
| FDX1 | hsa-miR-154-3p |
| FDX1 | hsa-miR-4773 |
| FDX1 | hsa-miR-30-3p |
| FDX1 | hsa-miR-200-3p |
| FDX1 | hsa-miR-7049-5p |
| FDX1 | hsa-miR-30d-3p |
| FDX1 | hsa-miR-3077-3p |
| FDX1 | hsa-miR-3934-5p |
| FDX1 | hsa-miR-144-5p |
| FDX1 | hsa-miR-880-3p |
| FDX1 | hsa-miR-6748-5p |
| FDX1 | hsa-miR-7194-5p |
| FDX1 | hsa-miR-6507-5p |
| FDX1 | hsa-miR-339-5p |
| FDX1 | hsa-miR-548ak |
| FDX1 | hsa-miR-6728-3p |
| FDX1 | hsa-miR-198 |
| FDX1 | hsa-miR-597 |
| FDX1 | hsa-miR-223-3p |
| FDX1 | hsa-miR-570-3p |
| FDX1 | hsa-miR-4793-3p |
| FDX1 | hsa-miR-421 |
| FDX1 | hsa-miR-881-3p |
| FDX1 | hsa-miR-7200-3p |
| FDX1 | hsa-miR-4652-3p |
| FDX1 | hsa-miR-3600 |
| FDX1 | hsa-miR-7398u-3p |
| FDX1 | hsa-miR-3120-5p |
| FDX1 | hsa-miR-6718-5p |
| FDX1 | hsa-miR-1285-5p |
| FDX1 | hsa-miR-3929 |
| FDX1 | hsa-miR-4482-5p |
| FDX1 | hsa-miR-29a-3p |
| FDX1 | hsa-miR-3074-1-3p |
| FDX1 | hsa-miR-495-3p |
| FDX1 | hsa-miR-4766-5p |
| FDX1 | hsa-miR-383-5p |
| FDX1 | hsa-miR-6332 |
| FDX1 | hsa-miR-596 |
| FDX1 | hsa-miR-1322 |
| FDX1 | hsa-miR-410 |
| FDX1 | hsa-miR-1245b-3p |
| FDX1 | hsa-miR-669e-3p |
| FDX1 | hsa-miR-1238-3p |
| FDX1 | hsa-miR-2335 |
| FDX1 | hsa-miR-6906-3p |
| FDX1 | hsa-miR-4489 |
| FDX1 | hsa-miR-133cd-5p |
| FDX1 | hsa-miR-8837 |
| FDX1 | hsa-miR-5006-3p |
| FDX1 | hsa-miR-214-3p |
| FDX1 | hsa-miR-9768-3p |
| FDX1 | hsa-miR-3671 |
| FDX1 | hsa-miR-5591-3p |
| FDX1 | hsa-miR-200a-3p |
| FDX1 | hsa-miR-325-3p |
| FDX1 | hsa-miR-490-5p |
| FDX1 | hsa-miR-883ab-3p |
| FDX1 | hsa-miR-6902-3p |
| FDX1 | hsa-miR-5186 |
| FDX1 | hsa-miR-498 |
| FDX1 | hsa-miR-8805 |
| FDX1 | hsa-miR-944 |
| FDX1 | hsa-miR-548bb-5p |
| FDX1 | hsa-let-7b-3p |
| FDX1 | hsa-miR-1276 |
| FDX1 | hsa-miR-505-3p.2 |
| FDX1 | hsa-miR-27-3p |
| FDX1 | hsa-miR-548ax |
| FDX1 | hsa-miR-742-5p |
| FDX1 | hsa-miR-34b-3p |
| FDX1 | hsa-miR-519d-5p |
| FDX1 | hsa-miR-6902-5p |
| FDX1 | hsa-miR-6854-5p |
| FDX1 | hsa-miR-4685-3p |
| FDX1 | hsa-miR-6879-3p |
| FDX1 | hsa-miR-619-3p |
| FDX1 | hsa-miR-548av-5p |
| FDX1 | hsa-miR-375 |
| FDX1 | hsa-miR-6507-3p |
| FDX1 | hsa-miR-7655-3p |
| FDX1 | hsa-miR-448 |
| FDX1 | hsa-miR-7385b-3p |
| FDX1 | hsa-miR-6826-5p |
| FDX1 | hsa-miR-520f-5p |
| FDX1 | hsa-miR-504 |
| FDX1 | hsa-miR-7321-5p |
| FDX1 | hsa-miR-151 |
| FDX1 | hsa-miR-151a-5p |
| FDX1 | hsa-miR-3925-3p |
| FDX1 | hsa-miR-376bc-5p |
| FDX1 | hsa-miR-4718 |
| FDX1 | hsa-miR-7856-5p |
| FDX1 | hsa-miR-7262a-5p |
| FDX1 | hsa-miR-3066-3p |
| FDX1 | hsa-miR-4715-5p |
| FDX1 | hsa-miR-505-3p.1 |
| FDX1 | hsa-miR-6858-3p |
| FDX1 | hsa-miR-497-5p |
| FDX1 | hsa-miR-7397-5p |
| FDX1 | hsa-miR-183-3p |
| FDX1 | hsa-miR-7114-5p |
| FDX1 | hsa-miR-642-3p |
| FDX1 | hsa-miR-1540-5p |
| FDX1 | hsa-miR-525-5p |
| FDX1 | hsa-miR-7226-3p |
| FDX1 | hsa-miR-1294 |
| FDX1 | hsa-miR-763 |
| FDX1 | hsa-miR-181b-3p |
| FDX1 | hsa-miR-532-3p |
| FDX1 | hsa-miR-6758-3p |
| FDX1 | hsa-miR-7177-5p |
| FDX1 | hsa-miR-6806-3p |
| FDX1 | hsa-miR-1841 |
| FDX1 | hsa-miR-6881-5p |
| FDX1 | hsa-miR-4775 |
| FDX1 | hsa-miR-6730-3p |
| FDX1 | hsa-miR-127-5p |
| FDX1 | hsa-miR-4708-3p |
| FDX1 | hsa-miR-2328-5p |
| FDX1 | hsa-miR-431-5p |
| FDX1 | hsa-miR-3620-5p |
| FDX1 | hsa-miR-336-3p |
| FDX1 | hsa-miR-2421 |
| FDX1 | hsa-miR-7052-3p |
| FDX1 | hsa-miR-1434-5p |
| FDX1 | hsa-miR-6529-5p |
| FDX1 | hsa-miR-7279-3p |
| FDX1 | hsa-miR-380-5p |
| FDX1 | hsa-miR-182-3p |
| FDX1 | hsa-miR-363-3p |
| FDX1 | hsa-miR-487-3p |
| FDX1 | hsa-miR-8086 |
| FDX1 | hsa-miR-1266-5p |
| FDX1 | hsa-miR-548l |
| FDX1 | hsa-miR-145-3p |
| FDX1 | hsa-miR-19-3p |
| FDX1 | hsa-miR-743b-3p |
| FDX1 | hsa-miR-5586-3p |
| FDX1 | hsa-miR-8901 |
| FDX1 | hsa-miR-451a |
| FDX1 | hsa-miR-3150b-5p |
| FDX1 | hsa-miR-548y |
| FDX1 | hsa-miR-128 |
| FDX1 | hsa-miR-5690 |
| FDX1 | hsa-miR-30e-3p |
| FDX1 | hsa-miR-4686 |
| FDX1 | hsa-miR-20 |
| FDX1 | hsa-miR-2681-5p |
| FDX1 | hsa-miR-1323-5p |
| FDX1 | hsa-miR-7669-3p |
| FDX1 | hsa-miR-499b-5p |
| FDX1 | hsa-miR-1293 |
| FDX1 | hsa-miR-5710 |
| FDX1 | hsa-miR-1295b-5p |
| FDX1 | hsa-miR-2311 |
| FDX1 | hsa-miR-892-5p |
| FDX1 | hsa-miR-107-3p |
| FDX1 | hsa-miR-4789-5p |
| FDX1 | hsa-miR-4484 |
| FDX1 | hsa-miR-1712-3p |
| FDX1 | hsa-miR-4646-3p |
| FDX1 | hsa-miR-6943-3p |
| FDX1 | hsa-miR-4645-3p |
| FDX1 | hsa-miR-292-5p |
| FDX1 | hsa-miR-7018-3p |
| FDX1 | hsa-miR-584-5p |
| FDX1 | hsa-miR-548i-5p |
| FDX1 | hsa-miR-3686 |
| FDX1 | hsa-miR-1626-5p |
| FDX1 | hsa-miR-205-5p |
| FDX1 | hsa-miR-492 |
| FDX1 | hsa-miR-466i-3p |
| FDX1 | hsa-miR-759 |
| FDX1 | hsa-miR-340-5p |
| FDX1 | hsa-miR-7352-3p |
| FDX1 | hsa-miR-208-3p |
| FDX1 | hsa-miR-2313-3p |
| FDX1 | hsa-miR-380 |
| FDX1 | hsa-miR-4474-5p |
| FDX1 | hsa-miR-3685 |
| FDX1 | hsa-miR-3106-3p |
| FDX1 | hsa-miR-758-3p |
| FDX1 | hsa-miR-1244 |
| FDX1 | hsa-miR-369-5p |
| FDX1 | hsa-miR-466 |
| FDX1 | hsa-miR-24-3p |
| FDX1 | hsa-miR-873-5p |
| FDX1 | hsa-miR-7161-3p |
| FDX1 | hsa-miR-211-5p |
| FDX1 | hsa-miR-330-3p.2 |
| FDX1 | hsa-miR-5132-3p |
| FDX1 | hsa-miR-4695-3p |
| FDX1 | hsa-miR-338-3p |
| FDX1 | hsa-miR-6794-3p |
| FDX1 | hsa-miR-330-5p |
| FDX1 | hsa-miR-3622b-5p |
| FDX1 | hsa-miR-222-5p |
| FDX1 | hsa-miR-3090-5p |
| FDX1 | hsa-miR-570-3p |
| FDX1 | hsa-miR-1537-5p |
| FDX1 | hsa-miR-4716-5p |
| FDX1 | hsa-miR-495-5p |
| FDX1 | hsa-miR-323 |
| FDX1 | hsa-miR-103-3p |
| FDX1 | hsa-miR-23 |
| FDX1 | hsa-miR-218-2-3p |
| FDX1 | hsa-miR-200a-3p |
| FDX1 | hsa-miR-3084bc-5p |
| FDX1 | hsa-miR-4473 |
| FDX1 | hsa-miR-6739-3p |
| FDX1 | hsa-miR-3148 |
| FDX1 | hsa-miR-500b-3p |
| FDX1 | hsa-miR-2475 |
| FDX1 | hsa-miR-1237-3p |
| FDX1 | hsa-miR-1190 |
| FDX1 | hsa-miR-7010-3p |
| FDX1 | hsa-miR-16-2-3p |
| FDX1 | hsa-miR-1193-5p |
| FDX1 | hsa-miR-6765-3p |
| FDX1 | hsa-miR-7193-5p |
| FDX1 | hsa-miR-207 |
| FDX1 | hsa-miR-3667-5p |
| FDX1 | hsa-miR-1301-3p |
| FDX1 | hsa-miR-8810 |
| FDX1 | hsa-miR-8877 |
| FDX1 | hsa-miR-24-3p |
| FDX1 | hsa-miR-6771-3p |
| FDX1 | hsa-miR-29-3p |
| FDX1 | hsa-miR-96-3p |
| FDX1 | hsa-miR-1304-3p |
| FDX1 | hsa-miR-526b-5p |
| FDX1 | hsa-miR-520 |
| FDX1 | hsa-miR-548ap-3p |
| FDX1 | hsa-miR-34a-3p |
| FDX1 | hsa-miR-7158-3p |
| FDX1 | hsa-miR-628-3p |
| FDX1 | hsa-miR-6738-3p |
| FDX1 | hsa-miR-323a-5p |
| FDX1 | hsa-miR-2284aa |
| FDX1 | hsa-miR-292b-5p |
| FDX1 | hsa-miR-4633-5p |
| FDX1 | hsa-miR-6780a-3p |
| FDX1 | hsa-miR-6911-3p |
| FDX1 | hsa-miR-548as-3p |
| FDX1 | hsa-miR-938 |
| FDX1 | hsa-miR-2489 |
| FDX1 | hsa-miR-4802-3p |
| FDX1 | hsa-miR-4517 |
| FDX1 | hsa-miR-182-5p |
| FDX1 | hsa-miR-6847-3p |
| FDX1 | hsa-miR-653 |
| FDX1 | hsa-miR-495-3p |
| FDX1 | hsa-miR-193b-5p |
| FDX1 | hsa-miR-4502 |
| FDX1 | hsa-miR-3664-3p |
| FDX1 | hsa-miR-599 |
| FDX1 | hsa-miR-107-5p |
| FDX1 | hsa-miR-7168-5p |
| FDX1 | hsa-miR-525-5p |
| FDX1 | hsa-miR-4671-3p |
| FDX1 | hsa-miR-548p |
| FDX1 | hsa-miR-5572 |
| FDX1 | hsa-miR-128-3p |
| FDX1 | hsa-miR-302a-5p |
| FDX1 | hsa-miR-320a |
| FDX1 | hsa-miR-7385c-3p |
| FDX1 | hsa-miR-5685 |
| FDX1 | hsa-miR-4267 |
| FDX1 | hsa-miR-153-5p |
| FDX1 | hsa-miR-6128 |
| FDX1 | hsa-miR-302 |
| FDX1 | hsa-miR-26b-3p |
| FDX1 | hsa-miR-7015-5p |
| FDX1 | hsa-miR-21-3p |
| FDX1 | hsa-miR-1185-5p |
| FDX1 | hsa-miR-3922-3p |
| FDX1 | hsa-miR-562 |
| FDX1 | hsa-miR-365a-5p |
| FDX1 | hsa-miR-7392-3p |
| FDX1 | hsa-miR-3150a-5p |
| FDX1 | hsa-miR-532-5p |
| FDX1 | hsa-miR-4801 |
| FDX1 | hsa-miR-6529b |
| FDX1 | hsa-miR-4513 |
| FDX1 | hsa-miR-379-3p |
| FDX1 | hsa-miR-3919 |
| FDX1 | hsa-miR-548as-5p |
| FDX1 | hsa-miR-21-5p |
| FDX1 | hsa-miR-2385-3p |
| FDX1 | hsa-miR-8847 |
| FDX1 | hsa-miR-766-3p |
| FDX1 | hsa-miR-7657-5p |
| FDX1 | hsa-miR-3575 |
| FDX1 | hsa-miR-3060-5p |
| FDX1 | hsa-miR-154-5p |
| FDX1 | hsa-miR-597-5p |
| FDX1 | hsa-miR-1251-5p |
| FDX1 | hsa-miR-2300-3p |
| FDX1 | hsa-miR-3622a-5p |
| FDX1 | hsa-miR-6779-5p |
| FDX1 | hsa-miR-374 |
| FDX1 | hsa-miR-4717-5p |
| FDX1 | hsa-miR-6965-3p |
| FDX1 | hsa-miR-328-3p |
| FDX1 | hsa-miR-1266 |
| FDX1 | hsa-miR-450b-5p |
| FDX1 | hsa-miR-6837-3p |
| FDX1 | hsa-miR-4480 |
| FDX1 | hsa-miR-571 |
| FDX1 | hsa-miR-5007-3p |
| FDX1 | hsa-miR-548x-3p |
| FDX1 | hsa-miR-879-3p |
| FDX1 | hsa-miR-328 |
| FDX1 | hsa-miR-2053 |
| FDX1 | hsa-miR-1468-3p |
| FDX1 | hsa-miR-6531 |
| FDX1 | hsa-miR-34bc-3p |
| FDX1 | hsa-miR-6780a-5p |
| FDX1 | hsa-miR-7387-3p |
| FDX1 | hsa-miR-6074 |
| FDX1 | hsa-miR-550a-3-5p |
| FDX1 | hsa-miR-6511a-5p |
| FDX1 | hsa-miR-34b-3p |
| FDX1 | hsa-miR-4753-3p |
| FDX1 | hsa-miR-4675 |
| FDX1 | hsa-let-7-5p |
| FDX1 | hsa-miR-190b-3p |
| FDX1 | hsa-miR-6865-3p |
| FDX1 | hsa-miR-8060 |
| FDX1 | hsa-miR-302c-3p.2 |
| FDX1 | hsa-miR-203b-5p |
| FDX1 | hsa-miR-133b-5p |
| FDX1 | hsa-miR-669p-3p |
| FDX1 | hsa-miR-188-3p |
| FDX1 | hsa-miR-7171-5p |
| FDX1 | hsa-miR-6793-5p |
| FDX1 | hsa-miR-6908-3p |
| FDX1 | hsa-miR-5580-3p |
| FDX1 | hsa-miR-6834-3p |
| FDX1 | hsa-miR-2285b |
| FDX1 | hsa-miR-365-3p |
| FDX1 | hsa-miR-673-5p |
| FDX1 | hsa-miR-376-3p |
| FDX1 | hsa-miR-219-3p |
| FDX1 | hsa-miR-4677-3p |
| FDX1 | hsa-miR-26c |
| FDX1 | hsa-miR-138-5p |
| FDX1 | hsa-miR-26-5p |
| FDX1 | hsa-miR-6754-3p |
| FDX1 | hsa-miR-2461-3p |
| FDX1 | hsa-miR-1814a |
| FDX1 | hsa-miR-3431 |
| FDX1 | hsa-miR-4653-3p |
| FDX1 | hsa-miR-3185 |
| FDX1 | hsa-miR-4720-5p |
| FDX1 | hsa-miR-4732-3p |
| FDX1 | hsa-miR-129b-5p |
| FDX1 | hsa-miR-144-3p |
| FDX1 | hsa-miR-549-3p |
| FDX1 | hsa-miR-627-3p |
| FDX1 | hsa-miR-6819-5p |
| FDX1 | hsa-miR-6124 |
| FDX1 | hsa-miR-6514-3p |
| FDX1 | hsa-miR-3065-3p |
| FDX1 | hsa-miR-655-3p |
| FDX1 | hsa-miR-4305 |
| FDX1 | hsa-miR-3618 |
| FDX1 | hsa-miR-1303 |
| FDX1 | hsa-miR-186-3p |
| FDX1 | hsa-miR-769-5p |
| FDX1 | hsa-miR-26-2-3p |
| FDX1 | hsa-miR-3618-5p |
| FDX1 | hsa-miR-2370-3p |
| FDX1 | hsa-miR-1343-3p |
| FDX1 | hsa-miR-548ao-3p |
| FDX1 | hsa-miR-4738-3p |
| FDX1 | hsa-miR-3926 |
| FDX1 | hsa-miR-548ao-5p |
| FDX1 | hsa-miR-4468 |
| FDX1 | hsa-miR-134 |
| FDX1 | hsa-miR-4741 |
| FDX1 | hsa-miR-5006-5p |
| FDX1 | hsa-miR-3662 |
| FDX1 | hsa-miR-1298-5p |
| FDX1 | hsa-miR-421-5p |
| FDX1 | hsa-miR-362-5p |
| FDX1 | hsa-miR-22-3p |
| FDX1 | hsa-miR-5702 |
| FDX1 | hsa-miR-3928-5p |
| FDX1 | hsa-miR-19-5p |
| FDX1 | hsa-miR-2285u |
| FDX1 | hsa-miR-767-3p |
| FDX1 | hsa-miR-881-5p |
| FDX1 | hsa-miR-383-3p |
| FDX1 | hsa-miR-1250-3p |
| FDX1 | hsa-miR-7213-3p |
| FDX1 | hsa-miR-136-5p |
| FDX1 | hsa-miR-2355-3p |
| FDX1 | hsa-miR-433-3p |
| FDX1 | hsa-miR-1247-5p |
| FDX1 | hsa-miR-6896-3p |
| FDX1 | hsa-miR-203 |
| FDX1 | hsa-miR-300-3p |
| FDX1 | hsa-miR-30b-3p |
| FDX1 | hsa-miR-5100 |
| FDX1 | hsa-miR-7156-5p |
| FDX1 | hsa-miR-139-5p |
| FDX1 | hsa-miR-3936 |
| FDX1 | hsa-miR-1973 |
| FDX1 | hsa-miR-544a |
| FDX1 | hsa-miR-323-3p |
| FDX1 | hsa-miR-508-5p |
| FDX1 | hsa-miR-363-5p |
| FDX1 | hsa-miR-7673-3p |
| FDX1 | hsa-miR-3074-5p |
| FDX1 | hsa-miR-195-5p |
| FDX1 | hsa-miR-3607-3p |
| FDX1 | hsa-miR-1285-3p |
| FDX1 | hsa-miR-4268 |
| FDX1 | hsa-miR-548j-3p |
| FDX1 | hsa-miR-4674 |
| FDX1 | hsa-miR-672-3p |
| FDX1 | hsa-miR-140-3p |
| FDX1 | hsa-miR-8818 |
| FDX1 | hsa-miR-3604 |
| FDX1 | hsa-miR-3977 |
| FDX1 | hsa-miR-548aj-3p |
| FDX1 | hsa-miR-8054 |
| FDX1 | hsa-miR-1662 |
| FDX1 | hsa-miR-624-5p |
| FDX1 | hsa-miR-28-3p |
| FDX1 | hsa-miR-3568 |
| FDX1 | hsa-miR-203b-3p |
| FDX1 | hsa-miR-491-5p |
| FDX1 | hsa-miR-340-5p |
| FDX1 | hsa-miR-6759-5p |
| FDX1 | hsa-miR-466c-3p |
| FDX1 | hsa-miR-6826-3p |
| FDX1 | hsa-miR-2301 |
| FDX1 | hsa-miR-670-5p |
| FDX1 | hsa-miR-1839 |
| FDX1 | hsa-miR-7094-1-5p |
| FDX1 | hsa-miR-5693 |
| FDX1 | hsa-miR-466b-3p |
| FDX1 | hsa-miR-471-5p |
| FDX1 | hsa-miR-7851-3p |
| FDX1 | hsa-miR-4252 |
| FDX1 | hsa-miR-7188-5p |
| FDX1 | hsa-miR-2284j |
| FDX1 | hsa-miR-2317 |
| FDX1 | hsa-miR-468-5p |
| FDX1 | hsa-miR-7670-5p |
| FDX1 | hsa-miR-1283 |
| FDX1 | hsa-miR-6730-5p |
| FDX1 | hsa-miR-29e |
| FDX1 | hsa-miR-4755-5p |
| FDX1 | hsa-miR-351-3p |
| FDX1 | hsa-miR-338-5p |
| FDX1 | hsa-miR-5001-3p |
| FDX1 | hsa-miR-578 |
| FDX1 | hsa-miR-3202 |
| FDX1 | hsa-miR-7202-5p |
| FDX1 | hsa-miR-513a-5p |
| FDX1 | hsa-miR-22-3p |
| FDX1 | hsa-miR-378a-5p |
| FDX1 | hsa-miR-7210-5p |
| FDX1 | hsa-miR-548h-5p |
| FDX1 | hsa-miR-6745 |
| GCSH | hsa-miR-224-5p |
| GCSH | hsa-miR-224-5p |
| GCSH | hsa-miR-7232-5p |
| GCSH | hsa-miR-4422 |
| GCSH | hsa-miR-8807 |
| GCSH | hsa-miR-374 |
| GCSH | hsa-miR-93-5p |
| GCSH | hsa-miR-1544-3p |
| GCSH | hsa-miR-7236-5p |
| GCSH | hsa-miR-124-5p |
| GCSH | hsa-miR-218-1-3p |
| GCSH | hsa-miR-130 |
| GCSH | hsa-miR-330-3p.2 |
| GCSH | hsa-miR-375 |
| GCSH | hsa-miR-106 |
| GCSH | hsa-miR-589-3p |
| GCSH | hsa-miR-19 |
| GCSH | hsa-miR-3675-3p |
| GCSH | hsa-miR-138-2-3p |
| GCSH | hsa-miR-7323-3p |
| GCSH | hsa-miR-301 |
| GCSH | hsa-miR-9-3p |
| GCSH | hsa-miR-624-5p |
| GCSH | hsa-miR-32-3p |
| GCSH | hsa-miR-6991-5p |
| GCSH | hsa-miR-7307-5p |
| GCSH | hsa-miR-454 |
| GCSH | hsa-miR-7296-5p |
| GCSH | hsa-miR-683 |
| GCSH | hsa-miR-20 |
| GCSH | hsa-miR-496b |
| GLS | hsa-miR-92-3p |
| GLS | hsa-miR-1185-5p |
| GLS | hsa-miR-5089-5p |
| GLS | hsa-miR-203 |
| GLS | hsa-miR-548x-5p |
| GLS | hsa-miR-4699-3p |
| GLS | hsa-miR-203-3p.1 |
| GLS | hsa-miR-570-3p |
| GLS | hsa-miR-3168 |
| GLS | hsa-miR-889 |
| GLS | hsa-miR-4721 |
| GLS | hsa-miR-891 |
| GLS | hsa-miR-3189-3p |
| GLS | hsa-miR-7864 |
| GLS | hsa-miR-144-3p |
| GLS | hsa-miR-3691-5p |
| GLS | hsa-miR-384-5p |
| GLS | hsa-miR-290 |
| GLS | hsa-miR-130-5p |
| GLS | hsa-miR-7274-3p |
| GLS | hsa-miR-2310 |
| GLS | hsa-miR-205-3p |
| GLS | hsa-miR-2904 |
| GLS | hsa-miR-452-3p |
| GLS | hsa-miR-758-3p |
| GLS | hsa-miR-6940-3p |
| GLS | hsa-let-7e-3p |
| GLS | hsa-miR-1257 |
| GLS | hsa-miR-548aj-5p |
| GLS | hsa-miR-7398u-3p |
| GLS | hsa-miR-6418-3p |
| GLS | hsa-miR-346-5p |
| GLS | hsa-miR-513c-5p |
| GLS | hsa-miR-222-3p |
| GLS | hsa-miR-653-5p |
| GLS | hsa-miR-208-3p |
| GLS | hsa-miR-4773 |
| GLS | hsa-miR-548d-3p |
| GLS | hsa-miR-7394a-5p |
| GLS | hsa-miR-7231-5p |
| GLS | hsa-miR-1941-3p |
| GLS | hsa-miR-208a-3p |
| GLS | hsa-miR-27 |
| GLS | hsa-miR-3938-5p |
| GLS | hsa-miR-466n-3p |
| GLS | hsa-miR-2285x |
| GLS | hsa-miR-2285aa |
| GLS | hsa-miR-675-3p |
| GLS | hsa-miR-483-3p |
| GLS | hsa-miR-491-3p |
| GLS | hsa-miR-17-5p |
| GLS | hsa-miR-769 |
| GLS | hsa-miR-7337-3p |
| GLS | hsa-miR-6388 |
| GLS | hsa-miR-514b-5p |
| GLS | hsa-miR-30b-3p |
| GLS | hsa-miR-9-5p |
| GLS | hsa-miR-532-5p |
| GLS | hsa-miR-20a-3p |
| GLS | hsa-miR-1964-5p |
| GLS | hsa-miR-196-1-3p |
| GLS | hsa-miR-363-3p |
| GLS | hsa-miR-4731-3p |
| GLS | hsa-miR-2285q |
| GLS | hsa-miR-4698 |
| GLS | hsa-miR-367-3p |
| GLS | hsa-miR-93-5p |
| GLS | hsa-miR-142-3p.2 |
| GLS | hsa-miR-8067 |
| GLS | hsa-miR-511-3p |
| GLS | hsa-miR-129-5p |
| GLS | hsa-miR-128-3p |
| GLS | hsa-miR-28-3p |
| GLS | hsa-miR-628-3p |
| GLS | hsa-miR-350 |
| GLS | hsa-miR-98-3p |
| GLS | hsa-miR-6908-3p |
| GLS | hsa-miR-142 |
| GLS | hsa-miR-7386j-3p |
| GLS | hsa-miR-1468-5p |
| GLS | hsa-miR-6071 |
| GLS | hsa-miR-7223-5p |
| GLS | hsa-miR-7286-3p |
| GLS | hsa-miR-1958 |
| GLS | hsa-miR-221-5p |
| GLS | hsa-miR-144-3p |
| GLS | hsa-miR-4662a-3p |
| GLS | hsa-miR-6984-3p |
| GLS | hsa-miR-376b-3p |
| GLS | hsa-miR-548ap-5p |
| GLS | hsa-miR-513a |
| GLS | hsa-miR-3682-5p |
| GLS | hsa-miR-573 |
| GLS | hsa-miR-8118 |
| GLS | hsa-miR-3065-3p |
| GLS | hsa-miR-4684-3p |
| GLS | hsa-miR-7703 |
| GLS | hsa-miR-3559-5p |
| GLS | hsa-miR-425 |
| GLS | hsa-miR-32-5p |
| GLS | hsa-miR-16-1-3p |
| GLS | hsa-miR-2399-5p |
| GLS | hsa-miR-21-3p |
| GLS | hsa-miR-6957-5p |
| GLS | hsa-miR-103 |
| GLS | hsa-miR-152-3p |
| GLS | hsa-miR-7666-3p |
| GLS | hsa-miR-632 |
| GLS | hsa-miR-1252-3p |
| GLS | hsa-miR-7349-3p |
| GLS | hsa-miR-466l-3p |
| GLS | hsa-miR-16-2-3p |
| GLS | hsa-miR-3058-3p |
| GLS | hsa-miR-371 |
| GLS | hsa-miR-10a-3p |
| GLS | hsa-miR-6853-3p |
| GLS | hsa-miR-2393 |
| GLS | hsa-miR-6675-5p |
| GLS | hsa-miR-7211-5p |
| GLS | hsa-miR-34a-3p |
| GLS | hsa-miR-6945-3p |
| GLS | hsa-miR-4471 |
| GLS | hsa-miR-2471-5p |
| GLS | hsa-miR-4491 |
| GLS | hsa-miR-2371 |
| GLS | hsa-miR-292a-5p |
| GLS | hsa-miR-499b-3p |
| GLS | hsa-miR-3956 |
| GLS | hsa-miR-4753-3p |
| GLS | hsa-miR-105-5p |
| GLS | hsa-miR-3576 |
| GLS | hsa-miR-203a-3p.1 |
| GLS | hsa-miR-885-5p |
| GLS | hsa-miR-642-3p |
| GLS | hsa-miR-875-5p |
| GLS | hsa-miR-704 |
| GLS | hsa-miR-7388b-3p |
| GLS | hsa-miR-6587-3p |
| GLS | hsa-miR-200c-3p |
| GLS | hsa-miR-212-3p |
| GLS | hsa-miR-4635 |
| GLS | hsa-miR-641 |
| GLS | hsa-miR-7283-3p |
| GLS | hsa-miR-329b |
| GLS | hsa-miR-548v |
| GLS | hsa-miR-145-5p |
| GLS | hsa-miR-330-3p.1 |
| GLS | hsa-miR-522-3p |
| GLS | hsa-miR-499b-5p |
| GLS | hsa-miR-411-5p.2 |
| GLS | hsa-miR-7054-5p |
| GLS | hsa-miR-7281-3p |
| GLS | hsa-miR-4744 |
| GLS | hsa-miR-7386g-5p |
| GLS | hsa-miR-3611 |
| GLS | hsa-miR-3059-5p |
| GLS | hsa-miR-7653-5p |
| GLS | hsa-miR-580 |
| GLS | hsa-miR-543-3p |
| GLS | hsa-miR-548bb-5p |
| GLS | hsa-miR-3652 |
| GLS | hsa-miR-4777-3p |
| GLS | hsa-miR-580-3p |
| GLS | hsa-miR-95-5p |
| GLS | hsa-miR-548ae-5p |
| GLS | hsa-miR-802 |
| GLS | hsa-miR-742-3p |
| GLS | hsa-miR-23 |
| GLS | hsa-miR-7386d-5p |
| GLS | hsa-miR-3145-3p |
| GLS | hsa-miR-548as-5p |
| GLS | hsa-miR-548aj-3p |
| GLS | hsa-miR-669j |
| GLS | hsa-miR-3566 |
| GLS | hsa-miR-6959-5p |
| GLS | hsa-miR-19a-5p |
| GLS | hsa-miR-6669-5p |
| GLS | hsa-miR-33-3p |
| GLS | hsa-miR-7357-5p |
| GLS | hsa-miR-7261-5p |
| GLS | hsa-miR-3583-3p |
| GLS | hsa-miR-2446 |
| GLS | hsa-miR-7262b-5p |
| GLS | hsa-miR-208a-5p |
| GLS | hsa-miR-302a-5p |
| GLS | hsa-miR-3572 |
| GLS | hsa-miR-200ac-5p |
| GLS | hsa-miR-518-3p |
| GLS | hsa-miR-5619-3p |
| GLS | hsa-miR-692 |
| GLS | hsa-miR-7077-5p |
| GLS | hsa-miR-693-3p |
| GLS | hsa-miR-6819-5p |
| GLS | hsa-miR-582-5p |
| GLS | hsa-miR-7-3p |
| GLS | hsa-miR-190a-5p |
| GLS | hsa-miR-8874 |
| GLS | hsa-miR-7680-5p |
| GLS | hsa-miR-3618-5p |
| GLS | hsa-miR-148b-5p |
| GLS | hsa-miR-34b-3p |
| GLS | hsa-miR-143-5p |
| GLS | hsa-miR-2464-5p |
| GLS | hsa-miR-4789-5p |
| GLS | hsa-miR-7386l-3p |
| GLS | hsa-miR-449c-3p |
| GLS | hsa-miR-544a |
| GLS | hsa-miR-584-3p |
| GLS | hsa-miR-4752 |
| GLS | hsa-miR-8864 |
| GLS | hsa-miR-669b-5p |
| GLS | hsa-miR-381-3p |
| GLS | hsa-miR-664-5p |
| GLS | hsa-miR-421-3p |
| GLS | hsa-miR-2300-3p |
| GLS | hsa-miR-872-3p |
| GLS | hsa-miR-549a |
| GLS | hsa-miR-7705 |
| GLS | hsa-miR-3660 |
| GLS | hsa-miR-200bc |
| GLS | hsa-miR-466bco-5p |
| GLS | hsa-miR-2284g |
| GLS | hsa-miR-5684 |
| GLS | hsa-miR-605-5p |
| GLS | hsa-miR-7200-5p |
| GLS | hsa-miR-613 |
| GLS | hsa-miR-3138 |
| GLS | hsa-miR-383-5p.2 |
| GLS | hsa-miR-551-5p |
| GLS | hsa-miR-4659b-5p |
| GLS | hsa-miR-374 |
| GLS | hsa-miR-1323 |
| GLS | hsa-miR-325-3p |
| GLS | hsa-miR-7385e-3p |
| GLS | hsa-miR-7159-3p |
| GLS | hsa-miR-29-5p |
| GLS | hsa-miR-6560-3p |
| GLS | hsa-miR-7053-3p |
| GLS | hsa-miR-140-3p.2 |
| GLS | hsa-miR-7386h-5p |
| GLS | hsa-miR-130a-5p |
| GLS | hsa-miR-2285n |
| GLS | hsa-miR-548bb-3p |
| GLS | hsa-miR-7285-3p |
| GLS | hsa-miR-495-3p |
| GLS | hsa-miR-3066-3p |
| GLS | hsa-miR-7321-5p |
| GLS | hsa-miR-4733-5p |
| GLS | hsa-miR-7386d-3p |
| GLS | hsa-miR-374b-5p |
| GLS | hsa-miR-491-5p |
| GLS | hsa-miR-2285af |
| GLS | hsa-miR-8084 |
| GLS | hsa-miR-4771 |
| GLS | hsa-miR-5572 |
| GLS | hsa-miR-3551-3p |
| GLS | hsa-miR-4528 |
| GLS | hsa-miR-6912-3p |
| GLS | hsa-miR-7371-5p |
| GLS | hsa-miR-7185-3p |
| GLS | hsa-let-7-5p |
| GLS | hsa-miR-217 |
| GLS | hsa-miR-29b-1-5p |
| GLS | hsa-miR-26-3p |
| GLS | hsa-miR-335-3p |
| GLS | hsa-miR-1953 |
| GLS | hsa-miR-3201 |
| GLS | hsa-miR-8059 |
| GLS | hsa-miR-1305 |
| GLS | hsa-miR-3064-3p |
| GLS | hsa-miR-7010-5p |
| GLS | hsa-miR-605 |
| GLS | hsa-miR-421 |
| GLS | hsa-miR-374b-3p |
| GLS | hsa-miR-6765-5p |
| GLS | hsa-miR-100-3p |
| GLS | hsa-miR-599 |
| GLS | hsa-miR-6733-5p |
| GLS | hsa-miR-142-5p |
| GLS | hsa-miR-4705 |
| GLS | hsa-miR-539-3p |
| GLS | hsa-miR-1237-3p |
| GLS | hsa-miR-3129-3p |
| GLS | hsa-miR-1948-5p |
| GLS | hsa-miR-1551-3p |
| GLS | hsa-miR-7009-3p |
| GLS | hsa-miR-4652-3p |
| GLS | hsa-miR-452-5p |
| GLS | hsa-miR-378-3p |
| GLS | hsa-miR-7386f-3p |
| GLS | hsa-miR-380-3p |
| GLS | hsa-miR-6953-5p |
| GLS | hsa-miR-1252-5p |
| GLS | hsa-miR-2330-3p |
| GLS | hsa-miR-499b-5p |
| GLS | hsa-miR-568 |
| GLS | hsa-miR-467g |
| GLS | hsa-miR-367-3p |
| GLS | hsa-miR-7270-3p |
| GLS | hsa-miR-2387 |
| GLS | hsa-miR-588 |
| GLS | hsa-miR-6124 |
| GLS | hsa-miR-7341-3p |
| GLS | hsa-miR-548e-5p |
| GLS | hsa-miR-567 |
| GLS | hsa-miR-3604 |
| GLS | hsa-miR-548aef |
| GLS | hsa-miR-579 |
| GLS | hsa-miR-3185 |
| GLS | hsa-miR-7266-5p |
| GLS | hsa-miR-875 |
| GLS | hsa-miR-7385f-3p |
| GLS | hsa-miR-1252 |
| GLS | hsa-miR-135 |
| GLS | hsa-miR-3187-3p |
| GLS | hsa-miR-4722-3p |
| GLS | hsa-miR-124-3p.1 |
| GLS | hsa-miR-32-3p |
| GLS | hsa-miR-8908f |
| GLS | hsa-miR-7465-5p |
| GLS | hsa-miR-1246 |
| GLS | hsa-miR-548f-5p |
| GLS | hsa-miR-672-3p |
| GLS | hsa-miR-1903 |
| GLS | hsa-miR-655-3p |
| GLS | hsa-miR-764-5p |
| GLS | hsa-miR-4715-3p |
| GLS | hsa-miR-7060-3p |
| GLS | hsa-miR-4526 |
| GLS | hsa-miR-2284ac |
| GLS | hsa-miR-944 |
| GLS | hsa-miR-1547-5p |
| GLS | hsa-miR-664 |
| GLS | hsa-miR-7116-3p |
| GLS | hsa-miR-7297-5p |
| GLS | hsa-miR-2377 |
| GLS | hsa-miR-155-5p |
| GLS | hsa-miR-7026-5p |
| GLS | hsa-miR-224-5p |
| GLS | hsa-miR-320-3p |
| GLS | hsa-miR-376ab |
| GLS | hsa-miR-7387-3p |
| GLS | hsa-miR-302 |
| GLS | hsa-miR-506-3p |
| GLS | hsa-miR-7384-5p |
| GLS | hsa-miR-2284aa |
| GLS | hsa-miR-2284l |
| GLS | hsa-miR-7201-5p |
| GLS | hsa-miR-467f |
| GLS | hsa-miR-6855-5p |
| GLS | hsa-miR-4271 |
| GLS | hsa-miR-361-3p |
| GLS | hsa-miR-5011-5p |
| GLS | hsa-miR-1306-5p |
| GLS | hsa-miR-7249-3p |
| GLS | hsa-miR-1591-5p |
| GLS | hsa-miR-513a-3p |
| GLS | hsa-miR-2423 |
| GLS | hsa-miR-3596c |
| GLS | hsa-miR-1248 |
| GLS | hsa-miR-690 |
| GLS | hsa-miR-155-3p |
| GLS | hsa-miR-2307 |
| GLS | hsa-miR-20-5p |
| GLS | hsa-miR-890-3p |
| GLS | hsa-miR-3060-5p |
| GLS | hsa-miR-7013-5p |
| GLS | hsa-miR-4775 |
| GLS | hsa-miR-6933-3p |
| GLS | hsa-miR-6322 |
| GLS | hsa-miR-323b-3p |
| GLS | hsa-miR-5701 |
| GLS | hsa-miR-7246-5p |
| GLS | hsa-miR-1814a |
| GLS | hsa-miR-4727-3p |
| GLS | hsa-miR-2352 |
| GLS | hsa-miR-7-5p |
| GLS | hsa-miR-2284k |
| GLS | hsa-miR-338-3p |
| GLS | hsa-miR-204-5p |
| GLS | hsa-miR-22-3p |
| GLS | hsa-miR-206 |
| GLS | hsa-miR-380 |
| GLS | hsa-miR-2285ad |
| GLS | hsa-miR-33 |
| GLS | hsa-miR-7253-5p |
| GLS | hsa-miR-130b-5p |
| GLS | hsa-miR-6935-3p |
| GLS | hsa-miR-379-3p |
| GLS | hsa-miR-6737-5p |
| GLS | hsa-miR-93-5p |
| GLS | hsa-miR-7083-3p |
| GLS | hsa-miR-2114-5p |
| GLS | hsa-miR-126-5p |
| GLS | hsa-miR-598-3p |
| GLS | hsa-miR-200-3p |
| GLS | hsa-miR-3097-3p |
| GLS | hsa-miR-8810 |
| GLS | hsa-miR-703 |
| GLS | hsa-miR-2284u |
| GLS | hsa-miR-714 |
| GLS | hsa-miR-7653-3p |
| GLS | hsa-miR-186-5p |
| GLS | hsa-miR-6937-3p |
| GLS | hsa-miR-6371 |
| GLS | hsa-miR-548ah-3p |
| GLS | hsa-miR-548at-5p |
| GLS | hsa-miR-7681-5p |
| GLS | hsa-miR-294 |
| GLS | hsa-miR-4657 |
| GLS | hsa-miR-3585-5p |
| GLS | hsa-miR-6901-3p |
| GLS | hsa-miR-7035-3p |
| GLS | hsa-miR-3613-3p |
| GLS | hsa-miR-466b-2-3p |
| GLS | hsa-miR-577-5p |
| GLS | hsa-miR-6776-3p |
| GLS | hsa-miR-607 |
| GLS | hsa-miR-7296-5p |
| GLS | hsa-miR-6365 |
| GLS | hsa-miR-1284 |
| GLS | hsa-miR-455-5p |
| GLS | hsa-miR-369-3p |
| GLS | hsa-miR-7202-3p |
| GLS | hsa-miR-8831 |
| GLS | hsa-miR-7313-3p |
| GLS | hsa-miR-7347-5p |
| GLS | hsa-miR-548at-3p |
| GLS | hsa-miR-200c-3p |
| GLS | hsa-miR-6775-3p |
| GLS | hsa-miR-7656-3p |
| GLS | hsa-miR-6999-5p |
| GLS | hsa-miR-3161 |
| GLS | hsa-miR-520-5p |
| GLS | hsa-miR-8817 |
| GLS | hsa-miR-452-5p |
| GLS | hsa-miR-186-3p |
| GLS | hsa-miR-6809-3p |
| GLS | hsa-miR-6509-5p |
| GLS | hsa-miR-6345 |
| GLS | hsa-miR-1-3p |
| GLS | hsa-miR-7330-5p |
| GLS | hsa-miR-5193 |
| GLS | hsa-miR-6348 |
| GLS | hsa-miR-6739-5p |
| GLS | hsa-miR-2897 |
| GLS | hsa-miR-6411 |
| GLS | hsa-miR-20-3p |
| GLS | hsa-miR-361-5p |
| GLS | hsa-miR-4776-3p |
| GLS | hsa-miR-154-3p |
| GLS | hsa-miR-374c-5p |
| GLS | hsa-miR-466c-3p |
| GLS | hsa-miR-4729 |
| GLS | hsa-miR-5692bc |
| GLS | hsa-let-7-3p |
| GLS | hsa-miR-7856-5p |
| GLS | hsa-miR-653 |
| GLS | hsa-miR-2284n |
| GLS | hsa-miR-7849-3p |
| GLS | hsa-miR-548k |
| GLS | hsa-miR-4530 |
| GLS | hsa-miR-409 |
| GLS | hsa-miR-548az-3p |
| GLS | hsa-miR-4256 |
| GLS | hsa-miR-5696 |
| GLS | hsa-miR-9-3p |
| GLS | hsa-miR-3654 |
| GLS | hsa-miR-467b-5p |
| GLS | hsa-miR-3919 |
| GLS | hsa-miR-142-3p |
| GLS | hsa-miR-181 |
| GLS | hsa-miR-140-3p |
| GLS | hsa-miR-7005-5p |
| GLS | hsa-miR-7267-3p |
| GLS | hsa-miR-3675-5p |
| GLS | hsa-miR-4708-5p |
| GLS | hsa-miR-7183-3p |
| GLS | hsa-miR-5687 |
| GLS | hsa-miR-497-5p |
| GLS | hsa-miR-2343 |
| GLS | hsa-miR-743b-3p |
| GLS | hsa-miR-455-3p |
| GLS | hsa-miR-19-5p |
| GLS | hsa-miR-4422 |
| GLS | hsa-miR-6802-5p |
| GLS | hsa-miR-339-5p |
| GLS | hsa-miR-6975-5p |
| GLS | hsa-miR-8054 |
| GLS | hsa-miR-4799-5p |
| GLS | hsa-miR-3613b |
| GLS | hsa-miR-518a-5p |
| GLS | hsa-let-7abf-3p |
| GLS | hsa-miR-7312-3p |
| GLS | hsa-miR-7199-5p |
| GLS | hsa-miR-3099-5p |
| GLS | hsa-miR-383-3p |
| GLS | hsa-miR-6981-3p |
| GLS | hsa-miR-7187-3p |
| GLS | hsa-miR-8869 |
| GLS | hsa-miR-219a-5p |
| GLS | hsa-miR-7298-5p |
| GLS | hsa-miR-212-5p |
| GLS | hsa-miR-467-3p |
| GLS | hsa-miR-3144-3p |
| GLS | hsa-miR-218a-2-3p |
| GLS | hsa-miR-549a |
| GLS | hsa-miR-125b-2-3p |
| GLS | hsa-miR-103-2-5p |
| GLS | hsa-miR-466bcp-3p |
| GLS | hsa-miR-6319 |
| GLS | hsa-miR-4325 |
| GLS | hsa-miR-101-1-5p |
| GLS | hsa-miR-2399-3p |
| GLS | hsa-miR-432-5p |
| GLS | hsa-miR-6509-3p |
| GLS | hsa-miR-667-5p |
| GLS | hsa-miR-32-5p |
| GLS | hsa-miR-5190 |
| GLS | hsa-miR-743ab-3p |
| GLS | hsa-miR-6216 |
| GLS | hsa-miR-5101 |
| GLS | hsa-miR-2285y |
| GLS | hsa-miR-29b-5p |
| GLS | hsa-miR-7116-5p |
| GLS | hsa-miR-5590-5p |
| GLS | hsa-miR-126-3p |
| GLS | hsa-miR-1933-3p |
| GLS | hsa-miR-376b-5p |
| GLS | hsa-miR-6847-3p |
| GLS | hsa-miR-589-3p |
| GLS | hsa-miR-1911-5p |
| GLS | hsa-miR-2284z |
| GLS | hsa-miR-5617-5p |
| GLS | hsa-miR-6875-3p |
| GLS | hsa-miR-196c-3p |
| GLS | hsa-miR-7359-3p |
| GLS | hsa-miR-30-3p |
| GLS | hsa-miR-6607-5p |
| GLS | hsa-miR-191-3p |
| GLS | hsa-miR-8063 |
| GLS | hsa-miR-1285-5p |
| GLS | hsa-miR-124 |
| GLS | hsa-miR-200a-5p |
| GLS | hsa-miR-4750-3p |
| GLS | hsa-miR-345-5p |
| GLS | hsa-miR-501-5p |
| GLS | hsa-miR-141-5p |
| GLS | hsa-miR-200ab-5p |
| GLS | hsa-miR-651-5p |
| GLS | hsa-miR-2358 |
| GLS | hsa-miR-2284x |
| GLS | hsa-miR-3573-3p |
| GLS | hsa-miR-520-3p |
| GLS | hsa-miR-4505 |
| GLS | hsa-miR-3084-3p |
| GLS | hsa-miR-1-3p |
| GLS | hsa-miR-4272 |
| GLS | hsa-miR-466aep-5p |
| GLS | hsa-miR-8895 |
| GLS | hsa-miR-8108 |
| GLS | hsa-miR-3924 |
| GLS | hsa-miR-6325 |
| GLS | hsa-miR-1304-5p |
| GLS | hsa-miR-26-5p |
| GLS | hsa-miR-31-5p |
| GLS | hsa-miR-3618-3p |
| GLS | hsa-miR-7676-5p |
| GLS | hsa-miR-7684-3p |
| GLS | hsa-miR-6384 |
| GLS | hsa-miR-6847-5p |
| GLS | hsa-miR-595 |
| GLS | hsa-miR-599-3p |
| GLS | hsa-miR-18-3p |
| GLS | hsa-miR-548e-3p |
| GLS | hsa-miR-742-5p |
| GLS | hsa-miR-1264-3p |
| GLS | hsa-miR-190-3p |
| GLS | hsa-miR-7379-5p |
| GLS | hsa-miR-2285j |
| GLS | hsa-miR-548c-5p |
| GLS | hsa-miR-181-5p |
| GLS | hsa-miR-1236-3p |
| GLS | hsa-miR-1208 |
| GLS | hsa-miR-587 |
| GLS | hsa-miR-7119-3p |
| GLS | hsa-miR-6758-3p |
| GLS | hsa-miR-219-3p |
| GLS | hsa-miR-8843 |
| GLS | hsa-miR-7295-3p |
| GLS | hsa-miR-6507-5p |
| GLS | hsa-miR-342-3p |
| GLS | hsa-miR-545-5p |
| GLS | hsa-miR-3128 |
| GLS | hsa-miR-6989-3p |
| GLS | hsa-miR-4430 |
| GLS | hsa-miR-669bf-3p |
| GLS | hsa-miR-8797 |
| GLS | hsa-miR-7657-5p |
| GLS | hsa-miR-7092-3p |
| GLS | hsa-miR-1843a-5p |
| GLS | hsa-miR-4307 |
| GLS | hsa-miR-5618-5p |
| GLS | hsa-miR-486-5p |
| GLS | hsa-miR-7332-3p |
| GLS | hsa-miR-486-5p |
| GLS | hsa-miR-3558-5p |
| GLS | hsa-miR-2417 |
| GLS | hsa-miR-380-5p |
| GLS | hsa-miR-326 |
| GLS | hsa-miR-23-3p |
| GLS | hsa-miR-330-3p |
| GLS | hsa-miR-3143 |
| GLS | hsa-miR-381-3p |
| GLS | hsa-miR-128 |
| GLS | hsa-miR-7463-3p |
| GLS | hsa-miR-7175-3p |
| GLS | hsa-miR-669o-5p |
| GLS | hsa-miR-429-3p |
| GLS | hsa-miR-548h-5p |
| GLS | hsa-miR-7299-5p |
| GLS | hsa-miR-628-3p |
| GLS | hsa-miR-7043-5p |
| GLS | hsa-miR-223-5p |
| GLS | hsa-miR-629-3p |
| GLS | hsa-miR-449-3p |
| GLS | hsa-miR-669i |
| GLS | hsa-miR-2985 |
| GLS | hsa-miR-3590-3p |
| GLS | hsa-miR-6520 |
| GLS | hsa-miR-522-3p |
| GLS | hsa-miR-194-5p |
| GLS | hsa-miR-4803 |
| GLS | hsa-miR-710 |
| GLS | hsa-miR-338-3p |
| GLS | hsa-miR-466 |
| GLS | hsa-miR-3167-5p |
| GLS | hsa-miR-557 |
| GLS | hsa-miR-7386e-5p |
| GLS | hsa-miR-323 |
| GLS | hsa-miR-7462-3p |
| GLS | hsa-miR-145a-3p |
| GLS | hsa-miR-5709-3p |
| GLS | hsa-miR-1273g-3p |
| GLS | hsa-miR-511 |
| GLS | hsa-miR-1185-3p |
| GLS | hsa-miR-3074-2-3p |
| GLS | hsa-miR-101-3p.1 |
| GLS | hsa-miR-4668-3p |
| GLS | hsa-miR-2477 |
| GLS | hsa-miR-518 |
| GLS | hsa-miR-449-5p |
| GLS | hsa-miR-4694-5p |
| GLS | hsa-miR-7344-5p |
| GLS | hsa-miR-3548 |
| GLS | hsa-miR-669e-3p |
| GLS | hsa-miR-940 |
| GLS | hsa-miR-1253 |
| GLS | hsa-miR-496 |
| GLS | hsa-miR-30d-3p |
| GLS | hsa-miR-7287-3p |
| GLS | hsa-miR-7352-3p |
| GLS | hsa-miR-2285b |
| GLS | hsa-miR-185-3p |
| GLS | hsa-miR-5582-3p |
| GLS | hsa-miR-302c-3p |
| GLS | hsa-miR-7225-3p |
| GLS | hsa-miR-4482-3p |
| GLS | hsa-miR-1597-3p |
| GLS | hsa-miR-3094-3p |
| GLS | hsa-miR-3473be |
| GLS | hsa-let-7a-3p |
| GLS | hsa-miR-4461 |
| GLS | hsa-miR-196-3p |
| GLS | hsa-miR-373-3p |
| GLS | hsa-miR-216c-5p |
| GLS | hsa-miR-3158-5p |
| GLS | hsa-miR-767-3p |
| GLS | hsa-miR-548am-5p |
| GLS | hsa-miR-7473-5p |
| GLS | hsa-miR-6512-5p |
| GLS | hsa-miR-7031-5p |
| GLS | hsa-miR-135-5p |
| GLS | hsa-miR-17-3p |
| GLS | hsa-miR-1225-5p |
| GLS | hsa-miR-4742-3p |
| GLS | hsa-miR-4791 |
| GLS | hsa-miR-935 |
| GLS | hsa-miR-4762-3p |
| GLS | hsa-miR-132-3p |
| GLS | hsa-miR-6760-3p |
| GLS | hsa-miR-1251-3p |
| GLS | hsa-miR-7683-3p |
| GLS | hsa-miR-7159-5p |
| GLS | hsa-miR-150-5p |
| GLS | hsa-miR-7857 |
| GLS | hsa-miR-122-5p |
| GLS | hsa-miR-3589 |
| GLS | hsa-miR-1279 |
| GLS | hsa-miR-454-5p |
| GLS | hsa-miR-3578 |
| GLS | hsa-miR-7671-5p |
| GLS | hsa-miR-8076 |
| GLS | hsa-miR-3560 |
| GLS | hsa-miR-16-3p |
| GLS | hsa-miR-3614-5p |
| GLS | hsa-miR-8806 |
| GLS | hsa-miR-6500-3p |
| GLS | hsa-miR-2322-5p |
| GLS | hsa-miR-7053-5p |
| GLS | hsa-miR-297bc-3p |
| GLS | hsa-miR-222-5p |
| GLS | hsa-miR-2284m |
| GLS | hsa-miR-6727-3p |
| GLS | hsa-miR-2957 |
| GLS | hsa-miR-22-5p |
| GLS | hsa-miR-4801 |
| GLS | hsa-miR-1273f |
| GLS | hsa-miR-6832-3p |
| GLS | hsa-let-7f-1-3p |
| GLS | hsa-miR-331-5p |
| GLS | hsa-miR-31-3p |
| GLS | hsa-miR-203-3p |
| GLS | hsa-miR-1323-3p |
| GLS | hsa-miR-1306-3p |
| GLS | hsa-miR-216a |
| GLS | hsa-miR-2902 |
| GLS | hsa-miR-3153 |
| GLS | hsa-miR-548y |
| GLS | hsa-miR-647 |
| GLS | hsa-miR-3087-5p |
| GLS | hsa-miR-1954 |
| GLS | hsa-miR-627-3p |
| GLS | hsa-miR-3119 |
| GLS | hsa-miR-7398q-3p |
| GLS | hsa-miR-7302-3p |
| GLS | hsa-miR-6704-5p |
| GLS | hsa-miR-2284a |
| GLS | hsa-miR-2365 |
| GLS | hsa-miR-2284 |
| GLS | hsa-miR-7234-5p |
| GLS | hsa-miR-6834-3p |
| GLS | hsa-miR-1249-5p |
| GLS | hsa-miR-377-3p |
| GLS | hsa-miR-3553 |
| GLS | hsa-miR-510-5p |
| GLS | hsa-miR-6077 |
| GLS | hsa-miR-7340-3p |
| GLS | hsa-miR-541-5p |
| GLS | hsa-miR-7262a-5p |
| GLS | hsa-miR-570-3p |
| GLS | hsa-miR-2319 |
| GLS | hsa-miR-1283a |
| GLS | hsa-miR-2284-5p |
| GLS | hsa-miR-4743-3p |
| GLS | hsa-miR-1842 |
| GLS | hsa-miR-7398-3p |
| GLS | hsa-miR-5197-5p |
| GLS | hsa-miR-691 |
| GLS | hsa-miR-4252 |
| GLS | hsa-miR-586 |
| GLS | hsa-miR-7054-3p |
| GLS | hsa-miR-6393 |
| GLS | hsa-miR-1226-5p |
| GLS | hsa-miR-5623-3p |
| GLS | hsa-miR-1198-5p |
| GLS | hsa-miR-4470 |
| GLS | hsa-miR-497-3p |
| GLS | hsa-miR-7371g-5p |
| GLS | hsa-miR-2372 |
| GLS | hsa-miR-1731-3p |
| GLS | hsa-miR-8056 |
| GLS | hsa-miR-4433b-3p |
| GLS | hsa-miR-527 |
| GLS | hsa-miR-548ap-3p |
| GLS | hsa-miR-466ade-3p |
| GLS | hsa-miR-301b-5p |
| GLS | hsa-miR-2367-3p |
| GLS | hsa-miR-3556a |
| GLS | hsa-miR-2467-5p |
| GLS | hsa-miR-4694-3p |
| GLS | hsa-let-7c-1-3p |
| GLS | hsa-miR-6403 |
| GLS | hsa-miR-183-5p |
| GLS | hsa-miR-7660-3p |
| GLS | hsa-miR-3714 |
| GLS | hsa-miR-222-3p |
| GLS | hsa-miR-7204-3p |
| GLS | hsa-miR-4709-3p |
| GLS | hsa-miR-484 |
| GLS | hsa-miR-6516-3p |
| GLS | hsa-miR-488-3p |
| GLS | hsa-miR-519-3p |
| GLS | hsa-miR-548g-5p |
| GLS | hsa-miR-8908c |
| GLS | hsa-miR-340-5p |
| GLS | hsa-miR-33-5p |
| GLS | hsa-miR-8838 |
| GLS | hsa-miR-4659a-5p |
| GLS | hsa-miR-372-3p |
| GLS | hsa-miR-7000-3p |
| GLS | hsa-miR-6922-3p |
| GLS | hsa-miR-377-3p |
| GLS | hsa-miR-3973 |
| GLS | hsa-miR-505-3p |
| GLS | hsa-miR-362-5p |
| GLS | hsa-miR-193-3p |
| GLS | hsa-miR-3595 |
| GLS | hsa-miR-7161-5p |
| GLS | hsa-miR-6824-5p |
| GLS | hsa-miR-4724-5p |
| GLS | hsa-miR-6899-5p |
| GLS | hsa-miR-495-3p |
| GLS | hsa-miR-292-5p |
| GLS | hsa-miR-2285u |
| GLS | hsa-miR-184-5p |
| GLS | hsa-miR-7651-3p |
| GLS | hsa-miR-409-5p |
| GLS | hsa-miR-449a-3p |
| GLS | hsa-miR-2285h |
| GLS | hsa-miR-224-5p |
| GLS | hsa-miR-4446-5p |
| GLS | hsa-miR-1191b-5p |
| GLS | hsa-miR-215-3p |
| GLS | hsa-miR-6515-3p |
| GLS | hsa-miR-5098 |
| GLS | hsa-miR-190b-3p |
| GLS | hsa-miR-6927-3p |
| GLS | hsa-miR-5625-3p |
| GLS | hsa-miR-7276-3p |
| GLS | hsa-miR-6970-5p |
| GLS | hsa-miR-1291 |
| GLS | hsa-miR-7236-5p |
| GLS | hsa-miR-467eh-5p |
| GLS | hsa-miR-129-3p |
| GLS | hsa-miR-30 |
| GLS | hsa-miR-548au-5p |
| GLS | hsa-miR-200a-3p |
| GLS | hsa-miR-3140-3p |
| GLS | hsa-miR-4670-3p |
| GLS | hsa-miR-146b-3p |
| GLS | hsa-miR-495-5p |
| GLS | hsa-miR-4276 |
| GLS | hsa-miR-192-3p |
| GLS | hsa-miR-3475-5p |
| GLS | hsa-miR-124-3p |
| GLS | hsa-miR-23a-5p |
| GLS | hsa-miR-2284y |
| GLS | hsa-miR-2970-5p |
| GLS | hsa-miR-7g-3p |
| GLS | hsa-miR-3105-5p |
| GLS | hsa-miR-2053 |
| GLS | hsa-miR-3568 |
| GLS | hsa-miR-219-1-3p |
| GLS | hsa-miR-1544-3p |
| GLS | hsa-miR-7022-5p |
| GLS | hsa-miR-6568-3p |
| GLS | hsa-miR-518-5p |
| GLS | hsa-miR-6961-3p |
| GLS | hsa-miR-19b-5p |
| GLS | hsa-miR-5583-5p |
| GLS | hsa-miR-4477a |
| GLS | hsa-miR-188-5p |
| GLS | hsa-miR-3570 |
| GLS | hsa-miR-6327 |
| GLS | hsa-miR-7352-5p |
| GLS | hsa-miR-7398b-3p |
| GLS | hsa-miR-7354-3p |
| GLS | hsa-miR-195-3p |
| GLS | hsa-miR-3083-5p |
| GLS | hsa-miR-190a-3p |
| GLS | hsa-miR-548ay-5p |
| GLS | hsa-miR-1298 |
| GLS | hsa-miR-376c-3p |
| GLS | hsa-miR-26 |
| GLS | hsa-miR-548n |
| GLS | hsa-miR-1547-3p |
| GLS | hsa-miR-6828-3p |
| GLS | hsa-miR-548i |
| GLS | hsa-miR-466b-4-3p |
| GLS | hsa-miR-145-5p |
| GLS | hsa-miR-3152-5p |
| GLS | hsa-miR-548am-3p |
| GLS | hsa-miR-141-3p |
| GLS | hsa-miR-4724-3p |
| GLS | hsa-miR-6969-3p |
| GLS | hsa-miR-1827 |
| GLS | hsa-miR-2891 |
| GLS | hsa-let-7d-3p |
| GLS | hsa-miR-3969 |
| GLS | hsa-miR-3170 |
| GLS | hsa-miR-25-3p |
| GLS | hsa-miR-3092-5p |
| GLS | hsa-miR-548o-5p |
| GLS | hsa-miR-7398a-3p |
| GLS | hsa-miR-569 |
| GLS | hsa-miR-3074-1-3p |
| GLS | hsa-miR-7440-3p |
| GLS | hsa-miR-7168-5p |
| GLS | hsa-miR-551b-5p |
| GLS | hsa-miR-200 |
| GLS | hsa-miR-7032-3p |
| GLS | hsa-miR-2483-3p |
| GLS | hsa-miR-27-3p |
| GLS | hsa-miR-6868-3p |
| GLS | hsa-miR-7395-5p |
| GLS | hsa-miR-302bd |
| GLS | hsa-miR-6715-3p |
| GLS | hsa-miR-7073-3p |
| GLS | hsa-miR-1468-3p |
| GLS | hsa-miR-7324-3p |
| GLS | hsa-miR-1695 |
| GLS | hsa-miR-361-5p |
| GLS | hsa-miR-889-5p |
| GLS | hsa-miR-7075-3p |
| GLS | hsa-miR-4682 |
| GLS | hsa-miR-338-5p |
| GLS | hsa-miR-337-3p |
| GLS | hsa-miR-301a-5p |
| GLS | hsa-miR-7039-3p |
| GLS | hsa-miR-1243 |
| GLS | hsa-miR-548x-3p |
| GLS | hsa-miR-5003-5p |
| GLS | hsa-miR-326-5p |
| GLS | hsa-miR-7383-5p |
| GLS | hsa-miR-4714-3p |
| GLS | hsa-miR-7386l-5p |
| GLS | hsa-miR-1933-5p |
| GLS | hsa-miR-3564 |
| GLS | hsa-miR-6122-5p |
| GLS | hsa-miR-6641-5p |
| GLS | hsa-let-7ag-3p |
| GLS | hsa-miR-548aq-3p |
| GLS | hsa-miR-548z |
| GLS | hsa-miR-29a-5p |
| GLS | hsa-miR-6913-3p |
| GLS | hsa-miR-1236-3p |
| GLS | hsa-miR-300 |
| GLS | hsa-miR-494 |
| GLS | hsa-miR-216c-3p |
| GLS | hsa-miR-16-5p |
| GLS | hsa-miR-302-5p |
| GLS | hsa-miR-1297 |
| GLS | hsa-let-7f-2-3p |
| GLS | hsa-miR-3680-5p |
| GLS | hsa-miR-7259-5p |
| GLS | hsa-miR-8850 |
| GLS | hsa-miR-106-3p |
| GLS | hsa-miR-295-5p |
| GLS | hsa-miR-7666-5p |
| GLS | hsa-miR-548j-5p |
| GLS | hsa-miR-5579-3p |
| GLS | hsa-miR-7679-5p |
| GLS | hsa-miR-150-5p |
| GLS | hsa-miR-429 |
| GLS | hsa-miR-2470 |
| GLS | hsa-miR-2378 |
| GLS | hsa-miR-6719-3p |
| GLS | hsa-miR-2284v |
| GLS | hsa-miR-6800-5p |
| GLS | hsa-miR-424-5p |
| GLS | hsa-miR-33a-3p |
| GLS | hsa-miR-517 |
| GLS | hsa-miR-25-3p |
| GLS | hsa-let-7b-3p |
| GLS | hsa-miR-221-3p |
| GLS | hsa-miR-3074-5p |
| GLS | hsa-miR-4279 |
| GLS | hsa-miR-465a-5p |
| GLS | hsa-miR-217-3p |
| GLS | hsa-miR-409-3p |
| GLS | hsa-miR-519 |
| GLS | hsa-miR-376c |
| GLS | hsa-miR-7382-3p |
| GLS | hsa-miR-6338 |
| GLS | hsa-miR-4301 |
| GLS | hsa-miR-651 |
| GLS | hsa-miR-6874-3p |
| GLS | hsa-miR-3923 |
| GLS | hsa-miR-5585-3p |
| GLS | hsa-miR-124-5p |
| GLS | hsa-miR-677-5p |
| GLS | hsa-miR-3084bc-5p |
| GLS | hsa-miR-450b |
| GLS | hsa-miR-1805-3p |
| GLS | hsa-miR-1277-5p |
| GLS | hsa-miR-543-5p |
| GLS | hsa-miR-6527 |
| GLS | hsa-miR-1199-5p |
| GLS | hsa-miR-559 |
| GLS | hsa-miR-7301-5p |
| GLS | hsa-miR-21-5p |
| GLS | hsa-miR-1747-3p |
| GLS | hsa-miR-4639-5p |
| GLS | hsa-miR-6983-3p |
| GLS | hsa-miR-6844 |
| GLS | hsa-miR-548q |
| GLS | hsa-miR-7332-5p |
| GLS | hsa-miR-7371b-5p |
| GLS | hsa-miR-669c-5p |
| GLS | hsa-miR-7327-5p |
| GLS | hsa-miR-7178-3p |
| GLS | hsa-miR-3149 |
| GLS | hsa-miR-344eh-5p |
| GLS | hsa-miR-8061 |
| GLS | hsa-miR-181a-2-3p |
| GLS | hsa-miR-195-5p |
| GLS | hsa-miR-2284j |
| GLS | hsa-miR-194-5p |
| GLS | hsa-miR-20b-3p |
| GLS | hsa-miR-10b-3p |
| GLS | hsa-miR-6628-3p |
| GLS | hsa-miR-4435 |
| GLS | hsa-miR-892-3p |
| GLS | hsa-miR-8804 |
| GLS | hsa-miR-6806-5p |
| GLS | hsa-miR-1804 |
| GLS | hsa-miR-7-5p |
| GLS | hsa-miR-1304-3p |
| GLS | hsa-miR-7326-5p |
| GLS | hsa-miR-8881 |
| GLS | hsa-miR-486-3p |
| GLS | hsa-miR-3148 |
| GLS | hsa-miR-561-3p |
| GLS | hsa-miR-664a-3p |
| GLS | hsa-miR-3557-3p |
| GLS | hsa-miR-6783-5p |
| GLS | hsa-miR-507 |
| GLS | hsa-miR-7273-5p |
| GLS | hsa-miR-143-3p |
| GLS | hsa-miR-4753-5p |
| GLS | hsa-miR-7647-5p |
| GLS | hsa-miR-5583-3p |
| GLS | hsa-miR-19-3p |
| GLS | hsa-miR-6890-5p |
| GLS | hsa-miR-7341-5p |
| GLS | hsa-miR-500b-5p |
| GLS | hsa-miR-292b-5p |
| GLS | hsa-miR-548s |
| GLS | hsa-miR-4286 |
| GLS | hsa-miR-6593-3p |
| GLS | hsa-miR-700-5p |
| GLS | hsa-miR-1460 |
| GLS | hsa-miR-669hk-3p |
| GLS | hsa-miR-30-5p |
| GLS | hsa-miR-3658 |
| GLS | hsa-miR-4760-5p |
| GLS | hsa-miR-202-5p |
| GLS | hsa-miR-503-3p |
| GLS | hsa-miR-322-3p |
| GLS | hsa-miR-548ay-3p |
| GLS | hsa-miR-7578 |
| GLS | hsa-miR-4659a-3p |
| GLS | hsa-miR-6932-3p |
| GLS | hsa-miR-410 |
| GLS | hsa-miR-3556b |
| GLS | hsa-miR-7243-5p |
| GLS | hsa-miR-6730-3p |
| GLS | hsa-miR-2480 |
| GLS | hsa-miR-7118-5p |
| GLS | hsa-miR-130-3p |
| GLS | hsa-miR-548u |
| GLS | hsa-miR-6910-3p |
| GLS | hsa-miR-3121-3p |
| GLS | hsa-miR-34b-3p |
| GLS | hsa-miR-7211-3p |
| GLS | hsa-miR-6481 |
| GLS | hsa-miR-365-3p |
| GLS | hsa-miR-3616-5p |
| GLS | hsa-miR-2439-3p |
| GLS | hsa-miR-1956-3p |
| GLS | hsa-miR-513c-3p |
| GLS | hsa-miR-7859 |
| GLS | hsa-miR-7164-5p |
| GLS | hsa-miR-2284ab |
| GLS | hsa-miR-106b-5p |
| GLS | hsa-miR-7371f-5p |
| GLS | hsa-miR-6950-3p |
| GLS | hsa-miR-548b-5p |
| GLS | hsa-miR-548ac |
| GLS | hsa-miR-34 |
| GLS | hsa-miR-3910 |
| GLS | hsa-miR-129-2-3p |
| GLS | hsa-miR-7243-3p |
| GLS | hsa-miR-4659b-3p |
| GLS | hsa-miR-200bc-3p |
| GLS | hsa-miR-8094 |
| GLS | hsa-miR-1a-5p |
| GLS | hsa-miR-6986-3p |
| GLS | hsa-miR-2110 |
| GLS | hsa-miR-146-5p |
| GLS | hsa-miR-7276-5p |
| GLS | hsa-miR-6834-5p |
| GLS | hsa-miR-302-3p |
| GLS | hsa-miR-2284-3p |
| GLS | hsa-miR-599-5p |
| GLS | hsa-miR-92-5p |
| GLS | hsa-miR-579-3p |
| GLS | hsa-miR-548ad-5p |
| GLS | hsa-miR-466c-5p |
| GLS | hsa-miR-7206-3p |
| GLS | hsa-miR-146 |
| GLS | hsa-miR-2888 |
| GLS | hsa-miR-548f-3p |
| GLS | hsa-miR-1841 |
| GLS | hsa-miR-548aw |
| GLS | hsa-miR-3163 |
| GLS | hsa-miR-1250-3p |
| GLS | hsa-miR-223-3p |
| GLS | hsa-miR-4282 |
| GLS | hsa-miR-1287 |
| GLS | hsa-miR-6997-5p |
| GLS | hsa-miR-107 |
| GLS | hsa-miR-520f-5p |
| GLS | hsa-miR-6507-3p |
| GLS | hsa-miR-561-3p |
| GLS | hsa-miR-4303 |
| GLS | hsa-miR-410-3p |
| GLS | hsa-miR-383-5p |
| GLS | hsa-miR-2465 |
| GLS | hsa-miR-548av-5p |
| GLS | hsa-miR-511-5p |
| GLS | hsa-miR-369-3p |
| GLS | hsa-miR-6611-5p |
| GLS | hsa-miR-4778-3p |
| GLS | hsa-miR-135a-3p |
| GLS | hsa-miR-3596a |
| GLS | hsa-miR-7307-5p |
| GLS | hsa-miR-758-3p |
| GLS | hsa-miR-8074 |
| GLS | hsa-miR-668-3p |
| GLS | hsa-miR-2284d |
| GLS | hsa-miR-7264-3p |
| GLS | hsa-miR-3596d |
| GLS | hsa-miR-411-3p |
| GLS | hsa-miR-544a |
| GLS | hsa-miR-3084-5p |
| GLS | hsa-miR-5692a |
| GLS | hsa-miR-7006-3p |
| GLS | hsa-miR-4735-5p |
| GLS | hsa-miR-6548-5p |
| GLS | hsa-miR-15b-3p |
| GLS | hsa-miR-7312-5p |
| GLS | hsa-miR-3662 |
| GLS | hsa-miR-589-5p |
| GLS | hsa-miR-7675-3p |
| GLS | hsa-miR-466g |
| GLS | hsa-miR-3688-3p |
| GLS | hsa-miR-3606-3p |
| GLS | hsa-miR-4719 |
| GLS | hsa-miR-600 |
| GLS | hsa-miR-7291-5p |
| GLS | hsa-miR-3551-5p |
| GLS | hsa-miR-1-5p |
| GLS | hsa-miR-548as-3p |
| GLS | hsa-miR-1343-5p |
| GLS | hsa-miR-1224 |
| GLS | hsa-miR-7293-5p |
| GLS | hsa-miR-7027-5p |
| GLS | hsa-miR-5694 |
| GLS | hsa-miR-2421 |
| GLS | hsa-miR-1245b-5p |
| GLS | hsa-miR-330-3p |
| GLS | hsa-miR-2462 |
| GLS | hsa-miR-1454 |
| GLS | hsa-miR-6583-5p |
| GLS | hsa-miR-1262-5p |
| GLS | hsa-miR-106 |
| GLS | hsa-miR-1296-3p |
| GLS | hsa-miR-1968-5p |
| GLS | hsa-miR-203-3p.2 |
| GLS | hsa-miR-2428 |
| GLS | hsa-miR-891b |
| GLS | hsa-miR-10b-5p |
| GLS | hsa-miR-221-3p |
| GLS | hsa-miR-7373d-3p |
| GLS | hsa-miR-193b-5p |
| GLS | hsa-miR-6416-3p |
| GLS | hsa-miR-460-5p |
| GLS | hsa-miR-7235-3p |
| GLS | hsa-miR-3942-3p |
| GLS | hsa-miR-7515 |
| GLS | hsa-miR-6581-5p |
| GLS | hsa-miR-577 |
| GLS | hsa-miR-7269-5p |
| GLS | hsa-miR-4712-3p |
| GLS | hsa-miR-6343 |
| GLS | hsa-miR-1434-3p |
| GLS | hsa-miR-92 |
| GLS | hsa-miR-547-5p |
| GLS | hsa-miR-3473g |
| GLS | hsa-miR-3617-5p |
| GLS | hsa-miR-346 |
| GLS | hsa-miR-4666a-3p |
| GLS | hsa-miR-3154 |
| GLS | hsa-miR-679 |
| GLS | hsa-miR-589-5p |
| GLS | hsa-miR-1298-5p |
| GLS | hsa-miR-2285 |
| GLS | hsa-miR-2893 |
| GLS | hsa-miR-6331 |
| GLS | hsa-let-7a-2-3p |
| GLS | hsa-miR-4539 |
| GLS | hsa-miR-548j-3p |
| GLS | hsa-miR-7386b-3p |
| GLS | hsa-miR-2052 |
| GLS | hsa-miR-6414 |
| GLS | hsa-miR-3077-3p |
| GLS | hsa-miR-1684-5p |
| GLS | hsa-miR-5089-3p |
| GLS | hsa-miR-3572-5p |
| GLS | hsa-miR-512-5p |
| GLS | hsa-miR-1912-3p |
| GLS | hsa-miR-466i-3p |
| GLS | hsa-miR-6851-3p |
| GLS | hsa-miR-372-5p |
| GLS | hsa-miR-325-5p |
| GLS | hsa-miR-6812-5p |
| GLS | hsa-miR-1282 |
| GLS | hsa-miR-382-3p |
| GLS | hsa-miR-132-3p |
| GLS | hsa-miR-5787 |
| GLS | hsa-miR-483-3p.2 |
| GLS | hsa-miR-3133 |
| GLS | hsa-miR-2311 |
| GLS | hsa-miR-105b |
| GLS | hsa-miR-499 |
| GLS | hsa-miR-5007-3p |
| GLS | hsa-miR-7161-3p |
| GLS | hsa-miR-7371j-3p |
| GLS | hsa-miR-300-5p |
| GLS | hsa-miR-2284c |
| GLS | hsa-miR-6524 |
| GLS | hsa-miR-2117 |
| GLS | hsa-miR-548c-3p |
| GLS | hsa-miR-7314-5p |
| GLS | hsa-miR-325 |
| GLS | hsa-miR-577-3p |
| GLS | hsa-miR-766-3p |
| GLS | hsa-miR-465c-5p |
| GLS | hsa-miR-29a-3p |
| GLS | hsa-miR-2329-5p |
| GLS | hsa-miR-205-5p |
| GLS | hsa-miR-2405 |
| GLS | hsa-miR-137 |
| GLS | hsa-miR-20 |
| GLS | hsa-miR-7670-5p |
| GLS | hsa-miR-500a-5p |
| GLS | hsa-miR-7650-3p |
| GLS | hsa-miR-30e-3p |
| GLS | hsa-miR-548aq-5p |
| GLS | hsa-miR-344b-3p |
| GLS | hsa-miR-330-3p.2 |
| GLS | hsa-miR-411-5p |
| GLS | hsa-miR-6545-3p |
| GLS | hsa-miR-5125-5p |
| GLS | hsa-miR-2895 |
| GLS | hsa-miR-548ar-3p |
| GLS | hsa-miR-7231-3p |
| GLS | hsa-miR-350-3p |
| GLS | hsa-miR-549-3p |
| GLS | hsa-miR-590-5p |
| GLS | hsa-miR-290b-5p |
| GLS | hsa-miR-875-3p |
| GLS | hsa-miR-938 |
| GLS | hsa-miR-363-3p |
| GLS | hsa-miR-7246-3p |
| GLS | hsa-miR-6954-5p |
| GLS | hsa-miR-328-3p |
| GLS | hsa-miR-548 |
| GLS | hsa-miR-6373 |
| GLS | hsa-miR-669 |
| GLS | hsa-miR-323a-3p |
| GLS | hsa-miR-450a-2-3p |
| GLS | hsa-miR-148-3p |
| GLS | hsa-miR-7393-5p |
| GLS | hsa-miR-21-5p |
| GLS | hsa-miR-1301 |
| GLS | hsa-miR-7a-1-3p |
| GLS | hsa-miR-548p |
| GLS | hsa-miR-655 |
| GLS | hsa-miR-7386n-3p |
| GLS | hsa-miR-3200-5p |
| GLS | hsa-miR-498 |
| GLS | hsa-miR-153-5p |
| GLS | hsa-miR-34-5p |
| GLS | hsa-miR-196a-3p |
| GLS | hsa-miR-580-5p |
| GLS | hsa-miR-15 |
| GLS | hsa-miR-203a-5p |
| GLS | hsa-miR-374-5p |
| GLS | hsa-miR-7678-5p |
| GLS | hsa-miR-101-3p |
| GLS | hsa-miR-548bd-3p |
| GLS | hsa-miR-670-3p |
| GLS | hsa-miR-2367-5p |
| GLS | hsa-miR-2322-3p |
| GLS | hsa-miR-293-5p |
| GLS | hsa-miR-7194-5p |
| GLS | hsa-miR-4760-3p |
| GLS | hsa-miR-694 |
| GLS | hsa-miR-3081-5p |
| GLS | hsa-miR-6951-3p |
| GLS | hsa-miR-2458 |
| GLS | hsa-miR-7386i-5p |
| GLS | hsa-miR-590-3p |
| GLS | hsa-miR-1190 |
| GLS | hsa-miR-6842-3p |
| GLS | hsa-miR-545-3p |
| GLS | hsa-miR-362-5p |
| GLS | hsa-miR-548ae-3p |
| GLS | hsa-miR-374-3p |
| GLS | hsa-miR-877-3p |
| GLS | hsa-miR-219a-2-3p |
| GLS | hsa-miR-548ak |
| GLS | hsa-miR-96-3p |
| GLS | hsa-miR-548m |
| GLS | hsa-miR-760-3p |
| GLS | hsa-miR-4668-5p |
| GLS | hsa-miR-651-3p |
| GLS | hsa-miR-6702-5p |
| GLS | hsa-miR-7333-5p |
| GLS | hsa-miR-3146 |
| GLS | hsa-miR-548i-3p |
| GLS | hsa-miR-203-5p |
| GLS | hsa-miR-548c-3p |
| GLS | hsa-miR-4714-5p |
| GLS | hsa-miR-664-3p |
| GLS | hsa-miR-336-3p |
| GLS | hsa-miR-8867 |
| GLS | hsa-miR-212-3p |
| GLS | hsa-miR-299-3p |
| GLS | hsa-miR-187-5p |
| GLS | hsa-miR-758-5p |
| GLS | hsa-miR-548l |
| GLS | hsa-miR-6885-3p |
| GLS | hsa-miR-1273h-3p |
| GLS | hsa-miR-2294 |
| GLS | hsa-miR-7386g-3p |
| GLS | hsa-miR-126a-5p |
| GLS | hsa-miR-8905 |
| GLS | hsa-miR-6734-5p |
| GLS | hsa-miR-6417 |
| GLS | hsa-miR-320a |
| GLS | hsa-miR-3473f |
| GLS | hsa-miR-548a-3p |
| GLS | hsa-miR-633 |
| GLS | hsa-miR-9-5p |
| GLS | hsa-miR-7664-5p |
| GLS | hsa-miR-545-3p |
| GLS | hsa-miR-3078-5p |
| GLS | hsa-miR-3470b |
| GLS | hsa-miR-5619-5p |
| GLS | hsa-miR-3653-3p |
| GLS | hsa-miR-3616-3p |
| GLS | hsa-miR-323-3p |
| GLS | hsa-miR-2381 |
| GLS | hsa-miR-656-5p |
| GLS | hsa-miR-106-5p |
| GLS | hsa-miR-489-3p |
| GLS | hsa-miR-517-5p |
| GLS | hsa-miR-1273e |
| GLS | hsa-miR-493-5p |
| GLS | hsa-miR-181b-1-3p |
| GLS | hsa-miR-1966-3p |
| GLS | hsa-miR-3593-3p |
| GLS | hsa-miR-186-5p |
| GLS | hsa-miR-6871-3p |
| GLS | hsa-miR-2284q |
| GLS | hsa-miR-450a |
| GLS | hsa-miR-23b-5p |
| GLS | hsa-miR-5584-3p |
| GLS | hsa-miR-3112-3p |
| GLS | hsa-miR-155-5p |
| GLS | hsa-miR-5624-5p |
| GLS | hsa-miR-101a-5p |
| GLS | hsa-miR-686 |
| GLS | hsa-miR-7186-3p |
| GLS | hsa-miR-4725-3p |
| GLS | hsa-miR-7655-3p |
| GLS | hsa-miR-4774-3p |
| GLS | hsa-miR-6508-5p |
| GLS | hsa-miR-340-5p |
| GLS | hsa-miR-6531 |
| GLS | hsa-miR-656 |
| GLS | hsa-miR-421-5p |
| GLS | hsa-miR-4511 |
| GLS | hsa-miR-1261 |
| GLS | hsa-miR-3646 |
| GLS | hsa-miR-7388a-3p |
| GLS | hsa-miR-2284b |
| GLS | hsa-miR-130c-5p |
| GLS | hsa-miR-3095-5p |
| GLS | hsa-miR-485-3p |
| GLS | hsa-miR-499a-3p |
| GLS | hsa-miR-3068-3p |
| GLS | hsa-miR-4766-5p |
| GLS | hsa-miR-876 |
| GLS | hsa-miR-6964-3p |
| GLS | hsa-miR-1258 |
| GLS | hsa-miR-465-5p |
| GLS | hsa-miR-302c-5p |
| GLS | hsa-miR-4517 |
| GLS | hsa-miR-151-3p |
| GLS | hsa-miR-429-5p |
| GLS | hsa-miR-30c-2-3p |
| GLS | hsa-miR-1545b-5p |
| GLS | hsa-miR-3167-3p |
| GLS | hsa-miR-6374 |
| GLS | hsa-miR-494-3p |
| GLS | hsa-miR-6770-5p |
| GLS | hsa-miR-16c-3p |
| GLS | hsa-miR-548a-5p |
| GLS | hsa-miR-505-3p.2 |
| GLS | hsa-miR-4495 |
| GLS | hsa-miR-7207-5p |
| GLS | hsa-miR-3964 |
| GLS | hsa-miR-576-5p |
| GLS | hsa-miR-208-5p |
| GLS | hsa-miR-7309-3p |
| GLS | hsa-miR-1745 |
| GLS | hsa-miR-6344 |
| GLS | hsa-miR-548aa |
| GLS | hsa-miR-487a |
| GLS | hsa-miR-7049-5p |
| GLS | hsa-miR-135ab-3p |
| GLS | hsa-miR-328 |
| GLS | hsa-miR-499b-3p |
| GLS | hsa-miR-2325 |
| GLS | hsa-miR-500 |
| GLS | hsa-miR-7390-5p |
| GLS | hsa-miR-524-5p |
| GLS | hsa-miR-467a-5p |
| GLS | hsa-miR-7199-3p |
| GLS | hsa-miR-411a |
| GLS | hsa-miR-3097-5p |
| GLS | hsa-miR-4260 |
| GLS | hsa-miR-543 |
| GLS | hsa-miR-302b-5p |
| GLS | hsa-miR-1543 |
| GLS | hsa-miR-4512 |
| GLS | hsa-miR-5697-3p |
| GLS | hsa-miR-6320 |
| GLS | hsa-miR-376c-5p |
| GLS | hsa-miR-105-5p |
| GLS | hsa-miR-4263 |
| GLS | hsa-miR-506-3p |
| GLS | hsa-miR-4480 |
| GLS | hsa-miR-2429 |
| GLS | hsa-miR-124-3p.2 |
| GLS | hsa-miR-4704-5p |
| GLS | hsa-miR-412 |
| GLS | hsa-miR-6903-5p |
| GLS | hsa-miR-101-3p |
| GLS | hsa-miR-2489 |
| GLS | hsa-miR-759 |
| GLS | hsa-miR-7374a-3p |
| GLS | hsa-miR-5003-3p |
| GLS | hsa-miR-129b-5p |
| GLS | hsa-miR-143-3p |
| GLS | hsa-miR-7297-3p |
| GLS | hsa-miR-1814 |
| GLS | hsa-miR-889-3p |
| GLS | hsa-miR-510-3p |
| GLS | hsa-miR-5585-5p |
| GLS | hsa-miR-548t-3p |
| GLS | hsa-miR-10b-5p |
| GLS | hsa-miR-5697 |
| GLS | hsa-miR-7258-5p |
| GLS | hsa-miR-302d-5p |
| GLS | hsa-miR-290a-5p |
| GLS | hsa-miR-1283 |
| GLS | hsa-miR-1237-3p |
| GLS | hsa-miR-8802 |
| GLS | hsa-miR-375 |
| GLS | hsa-miR-30a-3p |
| GLS | hsa-miR-2404 |
| GLS | hsa-miR-1643-3p |
| GLS | hsa-miR-1812-5p |
| GLS | hsa-miR-8808 |
| GLS | hsa-miR-7292-3p |
| GLS | hsa-miR-7358-5p |
| GLS | hsa-miR-6357 |
| GLS | hsa-miR-3121-3p |
| GLS | hsa-miR-7057-3p |
| GLS | hsa-miR-6237 |
| GLS | hsa-miR-301-3p |
| GLS | hsa-let-7c-3p |
| GLS | hsa-miR-2188-3p |
| GLS | hsa-miR-6780b-5p |
| GLS | hsa-miR-129-5p |
| GLS | hsa-miR-3166 |
| GLS | hsa-miR-7398t-3p |
| GLS | hsa-miR-1322 |
| GLS | hsa-miR-548ar-5p |
| GLS | hsa-miR-8818 |
| GLS | hsa-miR-539-5p |
| GLS | hsa-miR-548h-3p |
| GLS | hsa-miR-660-3p |
| GLS | hsa-miR-488-3p |
| GLS | hsa-miR-34c-3p |
| GLS | hsa-miR-3671 |
| GLS | hsa-miR-3913-3p |
| GLS | hsa-miR-553 |
| GLS | hsa-miR-2285z |
| GLS | hsa-miR-874-5p |
| GLS | hsa-miR-1179 |
| GLS | hsa-miR-766-3p |
| GLS | hsa-miR-3070-2-3p |
| GLS | hsa-miR-548ab |
| GLS | hsa-miR-448-3p |
| GLS | hsa-miR-3065-5p |
| GLS | hsa-miR-3070-3p |
| GLS | hsa-miR-30c-1-3p |
| GLS | hsa-miR-6128 |
| GLS | hsa-miR-7215-5p |
| GLS | hsa-miR-872 |
| GLS | hsa-miR-8106 |
| GLS | hsa-miR-297 |
| GLS | hsa-miR-6738-3p |
| GLS | hsa-miR-548d-5p |
| GLS | hsa-miR-294-5p |
| GLS | hsa-miR-3675-3p |
| GLS | hsa-miR-6749-3p |
| GLS | hsa-miR-292a-3p |
| GLS | hsa-miR-706 |
| GLS | hsa-miR-197-3p |
| GLS | hsa-miR-450a-1-3p |
| GLS | hsa-miR-548w |
| GLS | hsa-miR-2382-5p |
| GLS | hsa-miR-878-5p |
| GLS | hsa-miR-590-5p |
| LIAS | hsa-miR-5009-3p |
| LIAS | hsa-miR-500b-3p |
| LIAS | hsa-miR-4722-3p |
| LIAS | hsa-miR-4650 |
| LIAS | hsa-miR-4653-3p |
| LIAS | hsa-miR-513c-3p |
| LIAS | hsa-miR-520 |
| LIAS | hsa-miR-4652-3p |
| LIAS | hsa-miR-3609 |
| LIAS | hsa-miR-6887-3p |
| LIAS | hsa-miR-876-5p |
| LIAS | hsa-miR-411-5p |
| LIAS | hsa-miR-548c-3p |
| LIAS | hsa-miR-183-3p |
| LIAS | hsa-miR-7856-5p |
| LIAS | hsa-miR-7201-5p |
| LIAS | hsa-miR-4503 |
| LIAS | hsa-miR-512 |
| LIAS | hsa-miR-590-3p |
| LIAS | hsa-miR-9-5p |
| LIAS | hsa-miR-1183 |
| LIAS | hsa-miR-4731-5p |
| LIAS | hsa-miR-742-5p |
| LIAS | hsa-miR-212-5p |
| LIAS | hsa-miR-532-5p |
| LIAS | hsa-miR-518-3p |
| LIAS | hsa-miR-1915-3p |
| LIAS | hsa-miR-6926-3p |
| LIAS | hsa-miR-125 |
| LIAS | hsa-miR-513a-3p |
| LIAS | hsa-miR-3681-5p |
| LIAS | hsa-miR-8904a |
| LIAS | hsa-miR-3653-5p |
| LIAS | hsa-miR-6584-5p |
| LIAS | hsa-miR-8869 |
| LIAS | hsa-miR-6831-3p |
| LIAS | hsa-miR-2285af |
| LIAS | hsa-miR-3663-5p |
| LIAS | hsa-miR-6504-3p |
| LIAS | hsa-miR-1224 |
| LIAS | hsa-miR-24-3p |
| LIAS | hsa-miR-31-3p |
| LIAS | hsa-miR-6868-5p |
| LIAS | hsa-miR-4284 |
| LIAS | hsa-miR-6778-3p |
| LIAS | hsa-miR-667-3p |
| LIAS | hsa-miR-6509-3p |
| LIAS | hsa-miR-675-3p |
| LIAS | hsa-miR-6878-3p |
| LIAS | hsa-miR-153-5p |
| LIAS | hsa-miR-622 |
| LIAS | hsa-miR-5010-3p |
| LIAS | hsa-miR-2371 |
| LIAS | hsa-miR-31-5p |
| LIAS | hsa-miR-1297 |
| LIAS | hsa-miR-4460 |
| LIAS | hsa-miR-8057 |
| LIAS | hsa-miR-6747-3p |
| LIAS | hsa-miR-548s |
| LIAS | hsa-miR-489 |
| LIAS | hsa-miR-501-3p |
| LIAS | hsa-miR-2411-5p |
| LIAS | hsa-miR-6134 |
| LIAS | hsa-miR-3135b |
| LIAS | hsa-miR-20b-3p |
| LIAS | hsa-miR-4430 |
| LIAS | hsa-miR-1240 |
| LIAS | hsa-miR-4704-5p |
| LIAS | hsa-miR-4679 |
| LIAS | hsa-miR-6937-3p |
| LIAS | hsa-miR-3569 |
| LIAS | hsa-miR-509-5p |
| LIAS | hsa-miR-8082 |
| LIAS | hsa-miR-7165-3p |
| LIAS | hsa-miR-8103 |
| LIAS | hsa-miR-455-5p |
| LIAS | hsa-miR-4781-3p |
| LIAS | hsa-miR-6879-3p |
| LIAS | hsa-miR-484 |
| LIAS | hsa-miR-4743-3p |
| LIAS | hsa-miR-4720-5p |
| LIAS | hsa-miR-6840-3p |
| LIAS | hsa-miR-502-5p |
| LIAS | hsa-miR-372-3p |
| LIAS | hsa-miR-6797-5p |
| LIAS | hsa-miR-139-3p |
| LIAS | hsa-miR-583 |
| LIAS | hsa-miR-7472-5p |
| LIAS | hsa-miR-129-5p |
| LIAS | hsa-miR-2284-5p |
| LIAS | hsa-let-7a-2-3p |
| LIAS | hsa-miR-24-3p |
| LIAS | hsa-miR-3606-3p |
| LIAS | hsa-miR-6791-3p |
| LIAS | hsa-miR-92a-5p |
| LIAS | hsa-miR-6877-3p |
| LIAS | hsa-miR-4794 |
| LIAS | hsa-miR-6790-3p |
| LIAS | hsa-miR-19-5p |
| LIAS | hsa-miR-6792-5p |
| LIAS | hsa-miR-378a-5p |
| LIAS | hsa-miR-4477a |
| LIAS | hsa-miR-17-5p |
| LIAS | hsa-miR-455-3p.1 |
| LIAS | hsa-miR-6987-5p |
| LIAS | hsa-miR-6513-3p |
| LIAS | hsa-miR-4726-3p |
| LIAS | hsa-miR-1243-3p |
| LIAS | hsa-miR-2424 |
| LIAS | hsa-miR-6078 |
| LIAS | hsa-miR-6131 |
| LIAS | hsa-miR-342-3p |
| LIAS | hsa-miR-20 |
| LIAS | hsa-miR-6821-3p |
| LIAS | hsa-miR-181b-1-3p |
| LIAS | hsa-miR-6764-5p |
| LIAS | hsa-miR-5089-5p |
| LIAS | hsa-miR-6318 |
| LIAS | hsa-miR-4633-5p |
| LIAS | hsa-miR-4524b-3p |
| LIAS | hsa-miR-197-3p |
| LIAS | hsa-miR-543-3p |
| LIAS | hsa-let-7c-3p |
| LIAS | hsa-miR-599 |
| LIAS | hsa-miR-21-5p |
| LIAS | hsa-miR-6885-3p |
| LIAS | hsa-miR-17-3p |
| LIAS | hsa-miR-302-3p |
| LIAS | hsa-miR-548e-5p |
| LIAS | hsa-miR-520-3p |
| LIAS | hsa-miR-8867 |
| LIAS | hsa-miR-3151-5p |
| LIAS | hsa-miR-3116 |
| LIAS | hsa-miR-7053-3p |
| LIAS | hsa-miR-8062 |
| LIAS | hsa-miR-381-3p |
| LIAS | hsa-miR-4279 |
| LIAS | hsa-miR-7239-5p |
| LIAS | hsa-miR-582-3p |
| LIAS | hsa-miR-125a-3p |
| LIAS | hsa-miR-204-3p |
| LIAS | hsa-miR-499b-3p |
| LIAS | hsa-miR-143-3p |
| LIAS | hsa-miR-146a-3p |
| LIAS | hsa-miR-3557-3p |
| LIAS | hsa-miR-593-5p |
| LIAS | hsa-miR-183-5p |
| LIAS | hsa-miR-136-3p |
| LIAS | hsa-miR-26 |
| LIAS | hsa-miR-582-5p |
| LIAS | hsa-miR-302a-3p |
| LIAS | hsa-miR-1911-3p |
| LIAS | hsa-miR-4740-3p |
| LIAS | hsa-miR-5588-5p |
| LIAS | hsa-miR-499b-3p |
| LIAS | hsa-miR-302c-3p |
| LIAS | hsa-miR-619-5p |
| LIAS | hsa-miR-3658 |
| LIAS | hsa-miR-4534 |
| LIAS | hsa-miR-6128 |
| LIAS | hsa-miR-519 |
| LIAS | hsa-miR-5096 |
| LIAS | hsa-miR-2398 |
| LIAS | hsa-miR-296-3p |
| LIAS | hsa-miR-1302 |
| LIAS | hsa-miR-4727-3p |
| LIAS | hsa-miR-519-3p |
| LIAS | hsa-miR-223-5p |
| LIAS | hsa-miR-20-5p |
| LIAS | hsa-let-7g-3p |
| LIAS | hsa-miR-502a |
| LIAS | hsa-miR-3128 |
| LIAS | hsa-miR-509-3-5p |
| LIAS | hsa-miR-1193-5p |
| LIAS | hsa-miR-6529-5p |
| LIAS | hsa-miR-588 |
| LIAS | hsa-miR-3591-5p |
| LIAS | hsa-miR-451-3p |
| LIAS | hsa-miR-3652 |
| LIAS | hsa-miR-3913-5p |
| LIAS | hsa-miR-6515-5p |
| LIAS | hsa-miR-674-5p |
| LIAS | hsa-miR-8854 |
| LIAS | hsa-miR-126-5p |
| LIAS | hsa-miR-4999-3p |
| LIAS | hsa-miR-7188-3p |
| LIAS | hsa-miR-548ah-5p |
| LIAS | hsa-miR-6890-3p |
| LIAS | hsa-miR-6843-3p |
| LIAS | hsa-miR-483-3p.2 |
| LIAS | hsa-miR-465-5p |
| LIAS | hsa-miR-6707-5p |
| LIAS | hsa-miR-3646 |
| LIAS | hsa-miR-302 |
| LIAS | hsa-miR-1234-3p |
| LIAS | hsa-miR-2113 |
| LIAS | hsa-miR-590-5p |
| LIAS | hsa-miR-373-3p |
| LIAS | hsa-miR-675-5p |
| LIAS | hsa-miR-6727-3p |
| LIAS | hsa-miR-6744-3p |
| LIAS | hsa-miR-8868 |
| LIAS | hsa-miR-3120-3p |
| LIAS | hsa-miR-6506-5p |
| LIAS | hsa-miR-373-3p |
| LIAS | hsa-miR-3187-3p |
| LIAS | hsa-miR-15a-3p |
| LIAS | hsa-miR-4757-5p |
| LIAS | hsa-miR-181a-2-3p |
| LIAS | hsa-miR-4772-3p |
| LIAS | hsa-miR-489-3p |
| LIAS | hsa-miR-3122 |
| LIAS | hsa-miR-5691 |
| LIAS | hsa-miR-4645-5p |
| LIAS | hsa-miR-499 |
| LIAS | hsa-miR-1304-3p |
| LIAS | hsa-miR-6640-5p |
| LIAS | hsa-miR-4716-5p |
| LIAS | hsa-miR-4796-3p |
| LIAS | hsa-miR-455-5p |
| LIAS | hsa-miR-764 |
| LIAS | hsa-miR-4673 |
| LIAS | hsa-miR-1616 |
| LIAS | hsa-miR-4708-5p |
| LIAS | hsa-miR-4298 |
| LIAS | hsa-miR-448 |
| LIAS | hsa-miR-4755-3p |
| LIAS | hsa-miR-512-3p |
| LIAS | hsa-miR-499a-3p |
| LIAS | hsa-miR-193-5p |
| LIAS | hsa-miR-4693-5p |
| LIAS | hsa-miR-372 |
| LIAS | hsa-miR-7263-5p |
| LIAS | hsa-miR-3655 |
| LIAS | hsa-miR-378-5p |
| LIAS | hsa-miR-661 |
| LIAS | hsa-miR-640 |
| LIAS | hsa-miR-3934-5p |
| LIAS | hsa-miR-1254 |
| LIAS | hsa-miR-6829-3p |
| LIAS | hsa-miR-197-3p |
| LIAS | hsa-miR-7166-3p |
| LIAS | hsa-miR-31-5p |
| LIAS | hsa-miR-5095 |
| LIAS | hsa-miR-486-3p |
| LIAS | hsa-miR-4438 |
| LIAS | hsa-miR-6902-5p |
| LIAS | hsa-miR-4775 |
| LIAS | hsa-miR-26-5p |
| LIAS | hsa-miR-143-3p |
| LIAS | hsa-miR-511-5p |
| LIAS | hsa-let-7abf-3p |
| LIAS | hsa-miR-1976 |
| LIAS | hsa-miR-6819-3p |
| LIAS | hsa-miR-7294-5p |
| LIAS | hsa-miR-664a-5p |
| LIAS | hsa-miR-106 |
| LIAS | hsa-miR-4418 |
| LIAS | hsa-miR-4435 |
| LIAS | hsa-miR-2404 |
| LIAS | hsa-miR-7193-5p |
| LIAS | hsa-miR-5697-3p |
| LIAS | hsa-miR-1249-5p |
| LIAS | hsa-miR-7207-5p |
| LIAS | hsa-miR-452-5p |
| LIAS | hsa-miR-4735-5p |
| LIAS | hsa-miR-3576 |
| LIAS | hsa-miR-106-5p |
| LIAS | hsa-miR-671-5p |
| LIAS | hsa-miR-4269 |
| LIAS | hsa-miR-6807-5p |
| LIAS | hsa-miR-500-3p |
| LIAS | hsa-miR-548c-3p |
| LIAS | hsa-miR-2285 |
| LIAS | hsa-miR-122 |
| LIAS | hsa-miR-34bc-3p |
| LIAS | hsa-miR-892-3p |
| LIAS | hsa-miR-92a-2-5p |
| LIAS | hsa-miR-4799-3p |
| LIAS | hsa-miR-4301 |
| LIAS | hsa-miR-6848-3p |
| LIAS | hsa-miR-4524a-5p |
| LIAS | hsa-miR-493-5p |
| LIAS | hsa-miR-4524a-3p |
| LIAS | hsa-miR-1273g-3p |
| LIAS | hsa-miR-455-3p.2 |
| LIAS | hsa-miR-4677-3p |
| LIAS | hsa-miR-507 |
| LIAS | hsa-miR-93-5p |
| LIAS | hsa-miR-510 |
| LIAS | hsa-miR-4282 |
| LIAS | hsa-miR-1273f |
| LIAS | hsa-miR-433 |
| LIAS | hsa-miR-582-5p |
| LIAS | hsa-miR-3616-3p |
| LIAS | hsa-miR-3613b |
| LIAS | hsa-miR-93-5p |
| LIAS | hsa-miR-1626-3p |
| LIAS | hsa-miR-7151-3p |
| LIAS | hsa-miR-3958 |
| LIAS | hsa-miR-5589-5p |
| LIAS | hsa-miR-6715b-5p |
| LIAS | hsa-miR-4635 |
| LIAS | hsa-miR-6514-5p |
| LIPT1 | hsa-miR-301a-5p |
| LIPT1 | hsa-miR-653-5p |
| LIPT1 | hsa-miR-576-5p |
| LIPT1 | hsa-miR-4735-5p |
| LIPT1 | hsa-miR-7116-3p |
| LIPT1 | hsa-miR-410 |
| LIPT1 | hsa-miR-653 |
| LIPT1 | hsa-miR-181b-1-3p |
| LIPT1 | hsa-miR-1804 |
| LIPT1 | hsa-miR-4666a-3p |
| LIPT1 | hsa-miR-4742-3p |
| LIPT1 | hsa-miR-4524a-3p |
| LIPT1 | hsa-miR-489-3p |
| LIPT1 | hsa-miR-4738-3p |
| LIPT1 | hsa-miR-496.2 |
| LIPT1 | hsa-miR-489 |
| LIPT1 | hsa-miR-19-3p |
| LIPT1 | hsa-miR-510-3p |
| LIPT1 | hsa-miR-548g-3p |
| LIPT1 | hsa-miR-19 |
| LIPT1 | hsa-miR-514a-3p |
| LIPT1 | hsa-miR-4263 |
| LIPT1 | hsa-miR-30-5p |
| LIPT1 | hsa-miR-514a-3p |
| LIPT1 | hsa-miR-4712-3p |
| LIPT1 | hsa-miR-7056-3p |
| LIPT1 | hsa-miR-7859 |
| LIPT1 | hsa-miR-129-5p |
| LIPT1 | hsa-miR-4524a-5p |
| LIPT1 | hsa-miR-551-5p |
| LIPT1 | hsa-miR-6660-3p |
| LIPT1 | hsa-miR-1651-3p |
| LIPT1 | hsa-miR-674-5p |
| LIPT1 | hsa-miR-2285 |
| LIPT1 | hsa-miR-4477a |
| LIPT1 | hsa-miR-190-3p |
| LIPT1 | hsa-miR-129-5p |
| LIPT1 | hsa-miR-223-5p |
| LIPT1 | hsa-miR-340-5p |
| LIPT1 | hsa-miR-514-3p |
| LIPT1 | hsa-miR-1982-3p |
| LIPT1 | hsa-miR-6951-3p |
| LIPT1 | hsa-miR-2404 |
| MTF1 | hsa-miR-4476 |
| MTF1 | hsa-miR-5696 |
| MTF1 | hsa-miR-1232 |
| MTF1 | hsa-miR-139-5p |
| MTF1 | hsa-miR-7296-3p |
| MTF1 | hsa-miR-7681-5p |
| MTF1 | hsa-miR-7392-5p |
| MTF1 | hsa-miR-4638-3p |
| MTF1 | hsa-miR-295-5p |
| MTF1 | hsa-miR-3913-5p |
| MTF1 | hsa-miR-6698-3p |
| MTF1 | hsa-miR-185-5p |
| MTF1 | hsa-miR-7159-5p |
| MTF1 | hsa-miR-2330-5p |
| MTF1 | hsa-miR-153-5p |
| MTF1 | hsa-miR-5197-3p |
| MTF1 | hsa-miR-345-5p |
| MTF1 | hsa-miR-3556a |
| MTF1 | hsa-miR-3095-3p |
| MTF1 | hsa-miR-1586 |
| MTF1 | hsa-miR-363-3p |
| MTF1 | hsa-miR-6735-5p |
| MTF1 | hsa-miR-2284c |
| MTF1 | hsa-miR-6680-3p |
| MTF1 | hsa-miR-7195-3p |
| MTF1 | hsa-miR-4793-3p |
| MTF1 | hsa-miR-32-5p |
| MTF1 | hsa-miR-2305 |
| MTF1 | hsa-miR-672-5p |
| MTF1 | hsa-miR-196a-3p |
| MTF1 | hsa-miR-548ad-5p |
| MTF1 | hsa-miR-2457 |
| MTF1 | hsa-miR-3475-3p |
| MTF1 | hsa-miR-1273h-5p |
| MTF1 | hsa-miR-1705 |
| MTF1 | hsa-miR-650 |
| MTF1 | hsa-miR-7328-5p |
| MTF1 | hsa-miR-7280-5p |
| MTF1 | hsa-miR-212-3p |
| MTF1 | hsa-miR-6943-5p |
| MTF1 | hsa-miR-7150 |
| MTF1 | hsa-miR-1970 |
| MTF1 | hsa-miR-130c-5p |
| MTF1 | hsa-miR-5571-5p |
| MTF1 | hsa-miR-500-3p |
| MTF1 | hsa-miR-6319 |
| MTF1 | hsa-miR-365a-3p |
| MTF1 | hsa-miR-20a-3p |
| MTF1 | hsa-miR-1197 |
| MTF1 | hsa-miR-548ao-5p |
| MTF1 | hsa-miR-7058-5p |
| MTF1 | hsa-miR-2346 |
| MTF1 | hsa-let-7ag-3p |
| MTF1 | hsa-miR-651 |
| MTF1 | hsa-miR-7022-3p |
| MTF1 | hsa-miR-7352-5p |
| MTF1 | hsa-miR-3058-5p |
| MTF1 | hsa-miR-136-5p |
| MTF1 | hsa-miR-7678-5p |
| MTF1 | hsa-miR-4273 |
| MTF1 | hsa-miR-374b-3p |
| MTF1 | hsa-miR-7356-3p |
| MTF1 | hsa-miR-6955-3p |
| MTF1 | hsa-miR-7179-3p |
| MTF1 | hsa-miR-34-5p |
| MTF1 | hsa-miR-3680-5p |
| MTF1 | hsa-miR-455-3p.1 |
| MTF1 | hsa-miR-669ao-3p |
| MTF1 | hsa-miR-30-5p |
| MTF1 | hsa-miR-1240 |
| MTF1 | hsa-miR-4443 |
| MTF1 | hsa-miR-6679-5p |
| MTF1 | hsa-miR-7276-3p |
| MTF1 | hsa-miR-331-3p |
| MTF1 | hsa-miR-669i |
| MTF1 | hsa-miR-8867 |
| MTF1 | hsa-miR-141-3p |
| MTF1 | hsa-miR-466l-5p |
| MTF1 | hsa-miR-7398t-3p |
| MTF1 | hsa-miR-219b-3p |
| MTF1 | hsa-miR-6129 |
| MTF1 | hsa-miR-6515-3p |
| MTF1 | hsa-miR-520d-3p |
| MTF1 | hsa-miR-548y |
| MTF1 | hsa-miR-6775-3p |
| MTF1 | hsa-miR-548ae-5p |
| MTF1 | hsa-miR-7194-3p |
| MTF1 | hsa-miR-7156-3p |
| MTF1 | hsa-miR-1248 |
| MTF1 | hsa-miR-7067-5p |
| MTF1 | hsa-miR-4676-5p |
| MTF1 | hsa-miR-22-3p |
| MTF1 | hsa-miR-3916 |
| MTF1 | hsa-miR-124-3p |
| MTF1 | hsa-miR-2285j |
| MTF1 | hsa-miR-7012-5p |
| MTF1 | hsa-miR-648 |
| MTF1 | hsa-miR-204-5p |
| MTF1 | hsa-miR-3606-3p |
| MTF1 | hsa-miR-7386o-3p |
| MTF1 | hsa-miR-873-3p |
| MTF1 | hsa-miR-551b-5p |
| MTF1 | hsa-miR-7578 |
| MTF1 | hsa-miR-4251 |
| MTF1 | hsa-miR-344-3p |
| MTF1 | hsa-miR-362-5p |
| MTF1 | hsa-miR-31-5p |
| MTF1 | hsa-miR-381-5p |
| MTF1 | hsa-miR-7371-5p |
| MTF1 | hsa-miR-7216-3p |
| MTF1 | hsa-miR-4742 |
| MTF1 | hsa-miR-6819-3p |
| MTF1 | hsa-miR-7068-3p |
| MTF1 | hsa-miR-7192-3p |
| MTF1 | hsa-miR-8103 |
| MTF1 | hsa-miR-29a-3p |
| MTF1 | hsa-miR-568 |
| MTF1 | hsa-miR-1806 |
| MTF1 | hsa-miR-302-3p |
| MTF1 | hsa-miR-6942-3p |
| MTF1 | hsa-miR-7a-2-3p |
| MTF1 | hsa-miR-7478-5p |
| MTF1 | hsa-miR-7162-3p |
| MTF1 | hsa-miR-7353-3p |
| MTF1 | hsa-miR-7659-5p |
| MTF1 | hsa-miR-7285-5p |
| MTF1 | hsa-miR-3934-3p |
| MTF1 | hsa-miR-6750-5p |
| MTF1 | hsa-miR-2386 |
| MTF1 | hsa-miR-2411-5p |
| MTF1 | hsa-miR-383-5p.2 |
| MTF1 | hsa-miR-8847 |
| MTF1 | hsa-miR-583 |
| MTF1 | hsa-miR-1291 |
| MTF1 | hsa-miR-146b-3p |
| MTF1 | hsa-miR-6799-5p |
| MTF1 | hsa-miR-548al |
| MTF1 | hsa-miR-6124 |
| MTF1 | hsa-miR-7476-5p |
| MTF1 | hsa-miR-34 |
| MTF1 | hsa-miR-7308-3p |
| MTF1 | hsa-miR-7024-3p |
| MTF1 | hsa-miR-605-3p |
| MTF1 | hsa-miR-7361-5p |
| MTF1 | hsa-miR-135 |
| MTF1 | hsa-miR-1397-5p |
| MTF1 | hsa-miR-4318 |
| MTF1 | hsa-miR-4480 |
| MTF1 | hsa-miR-577-5p |
| MTF1 | hsa-miR-2113 |
| MTF1 | hsa-miR-548t-5p |
| MTF1 | hsa-miR-2284m |
| MTF1 | hsa-miR-5615-3p |
| MTF1 | hsa-miR-4732-5p |
| MTF1 | hsa-miR-3064-3p |
| MTF1 | hsa-miR-6768-5p |
| MTF1 | hsa-miR-1185-3p |
| MTF1 | hsa-miR-5582-3p |
| MTF1 | hsa-miR-5010-5p |
| MTF1 | hsa-miR-2285af |
| MTF1 | hsa-miR-330-3p |
| MTF1 | hsa-miR-374-5p |
| MTF1 | hsa-miR-7286-3p |
| MTF1 | hsa-miR-711 |
| MTF1 | hsa-miR-8842 |
| MTF1 | hsa-miR-628-5p |
| MTF1 | hsa-miR-30e-3p |
| MTF1 | hsa-miR-7074-5p |
| MTF1 | hsa-miR-543 |
| MTF1 | hsa-miR-6121-5p |
| MTF1 | hsa-miR-4298 |
| MTF1 | hsa-miR-2298 |
| MTF1 | hsa-miR-513b-3p |
| MTF1 | hsa-miR-7186-5p |
| MTF1 | hsa-miR-196c-3p |
| MTF1 | hsa-miR-7166-3p |
| MTF1 | hsa-miR-6715-3p |
| MTF1 | hsa-miR-883-5p |
| MTF1 | hsa-miR-181a-5p |
| MTF1 | hsa-miR-3127-5p |
| MTF1 | hsa-miR-519e-3p |
| MTF1 | hsa-miR-544a |
| MTF1 | hsa-miR-101b-3p.2 |
| MTF1 | hsa-miR-7062-5p |
| MTF1 | hsa-miR-7302-5p |
| MTF1 | hsa-miR-1678 |
| MTF1 | hsa-miR-7306-5p |
| MTF1 | hsa-miR-3544-5p |
| MTF1 | hsa-miR-877-5p |
| MTF1 | hsa-miR-1691 |
| MTF1 | hsa-miR-6993-5p |
| MTF1 | hsa-miR-669d-5p |
| MTF1 | hsa-miR-6917-3p |
| MTF1 | hsa-miR-6846-5p |
| MTF1 | hsa-miR-7463-3p |
| MTF1 | hsa-miR-541-3p |
| MTF1 | hsa-miR-519 |
| MTF1 | hsa-miR-153 |
| MTF1 | hsa-miR-7395-5p |
| MTF1 | hsa-miR-7204-3p |
| MTF1 | hsa-miR-1614-3p |
| MTF1 | hsa-miR-5096 |
| MTF1 | hsa-miR-135a-3p |
| MTF1 | hsa-miR-527 |
| MTF1 | hsa-miR-344-5p |
| MTF1 | hsa-miR-1651-5p |
| MTF1 | hsa-miR-203b-3p |
| MTF1 | hsa-miR-6984-5p |
| MTF1 | hsa-miR-8850 |
| MTF1 | hsa-miR-291 |
| MTF1 | hsa-miR-29-3p |
| MTF1 | hsa-miR-3972 |
| MTF1 | hsa-miR-9-5p |
| MTF1 | hsa-miR-3977 |
| MTF1 | hsa-miR-1324 |
| MTF1 | hsa-miR-151-3p |
| MTF1 | hsa-miR-548a-3p |
| MTF1 | hsa-miR-3159 |
| MTF1 | hsa-miR-148b-3p |
| MTF1 | hsa-miR-211-3p |
| MTF1 | hsa-miR-2285t |
| MTF1 | hsa-miR-3109-3p |
| MTF1 | hsa-miR-8086 |
| MTF1 | hsa-miR-7315-5p |
| MTF1 | hsa-miR-4426 |
| MTF1 | hsa-miR-524-5p |
| MTF1 | hsa-miR-2373-5p |
| MTF1 | hsa-miR-6759-5p |
| MTF1 | hsa-miR-154b |
| MTF1 | hsa-miR-137 |
| MTF1 | hsa-miR-4677-3p |
| MTF1 | hsa-miR-4437 |
| MTF1 | hsa-miR-6896-3p |
| MTF1 | hsa-miR-204-5p |
| MTF1 | hsa-miR-3162-5p |
| MTF1 | hsa-miR-7515 |
| MTF1 | hsa-miR-598-5p |
| MTF1 | hsa-miR-6965-3p |
| MTF1 | hsa-miR-7229-3p |
| MTF1 | hsa-miR-940 |
| MTF1 | hsa-miR-1655-3p |
| MTF1 | hsa-miR-128-3p |
| MTF1 | hsa-miR-3112-5p |
| MTF1 | hsa-miR-7109-5p |
| MTF1 | hsa-miR-7865 |
| MTF1 | hsa-miR-1188-5p |
| MTF1 | hsa-miR-4659b-3p |
| MTF1 | hsa-miR-642a-5p |
| MTF1 | hsa-miR-8838 |
| MTF1 | hsa-miR-7064-5p |
| MTF1 | hsa-miR-6123 |
| MTF1 | hsa-miR-2366 |
| MTF1 | hsa-miR-9a-3p |
| MTF1 | hsa-miR-6975-3p |
| MTF1 | hsa-miR-3926 |
| MTF1 | hsa-miR-105b |
| MTF1 | hsa-miR-7051-3p |
| MTF1 | hsa-miR-767-5p |
| MTF1 | hsa-miR-24-3p |
| MTF1 | hsa-miR-7371e-5p |
| MTF1 | hsa-miR-3622b-5p |
| MTF1 | hsa-miR-2450a |
| MTF1 | hsa-miR-3094-5p |
| MTF1 | hsa-miR-181c-5p |
| MTF1 | hsa-miR-2054 |
| MTF1 | hsa-miR-24-3p |
| MTF1 | hsa-miR-6744-3p |
| MTF1 | hsa-miR-7398i-3p |
| MTF1 | hsa-miR-520a-3p |
| MTF1 | hsa-miR-2293 |
| MTF1 | hsa-miR-212-3p |
| MTF1 | hsa-miR-8119 |
| MTF1 | hsa-miR-2465 |
| MTF1 | hsa-miR-589-5p |
| MTF1 | hsa-miR-4725-5p |
| MTF1 | hsa-miR-4766-3p |
| MTF1 | hsa-miR-7653-5p |
| MTF1 | hsa-miR-6418-5p |
| MTF1 | hsa-let-7f-2-3p |
| MTF1 | hsa-miR-1275 |
| MTF1 | hsa-miR-23a-5p |
| MTF1 | hsa-miR-3470b |
| MTF1 | hsa-miR-6972-5p |
| MTF1 | hsa-miR-586 |
| MTF1 | hsa-miR-6501-5p |
| MTF1 | hsa-miR-3188 |
| MTF1 | hsa-miR-4700-3p |
| MTF1 | hsa-miR-1626-3p |
| MTF1 | hsa-miR-6812-5p |
| MTF1 | hsa-miR-7198-3p |
| MTF1 | hsa-miR-148-5p |
| MTF1 | hsa-miR-3978 |
| MTF1 | hsa-miR-522-3p |
| MTF1 | hsa-miR-7378-5p |
| MTF1 | hsa-miR-6810-5p |
| MTF1 | hsa-miR-548aq-5p |
| MTF1 | hsa-miR-3596d |
| MTF1 | hsa-miR-18 |
| MTF1 | hsa-miR-7398j-3p |
| MTF1 | hsa-miR-3591-5p |
| MTF1 | hsa-miR-7083-3p |
| MTF1 | hsa-miR-3546 |
| MTF1 | hsa-miR-4786-5p |
| MTF1 | hsa-miR-5588-5p |
| MTF1 | hsa-miR-6929-5p |
| MTF1 | hsa-miR-662 |
| MTF1 | hsa-miR-502-3p |
| MTF1 | hsa-miR-570-3p |
| MTF1 | hsa-miR-3145-3p |
| MTF1 | hsa-miR-1354 |
| MTF1 | hsa-miR-298-3p |
| MTF1 | hsa-miR-4999-3p |
| MTF1 | hsa-miR-500b-3p |
| MTF1 | hsa-miR-4737 |
| MTF1 | hsa-miR-4646-5p |
| MTF1 | hsa-miR-881-3p |
| MTF1 | hsa-miR-8905 |
| MTF1 | hsa-miR-6715b-3p |
| MTF1 | hsa-miR-4507 |
| MTF1 | hsa-miR-146-5p |
| MTF1 | hsa-miR-6975-5p |
| MTF1 | hsa-miR-876-3p |
| MTF1 | hsa-miR-4419a |
| MTF1 | hsa-miR-6613-3p |
| MTF1 | hsa-miR-369-3p |
| MTF1 | hsa-miR-3134 |
| MTF1 | hsa-miR-1812-5p |
| MTF1 | hsa-miR-7321-3p |
| MTF1 | hsa-miR-7289-3p |
| MTF1 | hsa-miR-101a-3p.2 |
| MTF1 | hsa-miR-7028-3p |
| MTF1 | hsa-miR-1467-3p |
| MTF1 | hsa-miR-693-5p |
| MTF1 | hsa-miR-1962 |
| MTF1 | hsa-miR-8074 |
| MTF1 | hsa-miR-8076 |
| MTF1 | hsa-miR-146a-3p |
| MTF1 | hsa-miR-2329-3p |
| MTF1 | hsa-miR-16-3p |
| MTF1 | hsa-miR-513c-5p |
| MTF1 | hsa-miR-7372-3p |
| MTF1 | hsa-miR-6970-3p |
| MTF1 | hsa-miR-6523b |
| MTF1 | hsa-miR-6851-5p |
| MTF1 | hsa-miR-3059-3p |
| MTF1 | hsa-miR-1273g-5p |
| MTF1 | hsa-miR-3063-5p |
| MTF1 | hsa-miR-6331 |
| MTF1 | hsa-miR-8094 |
| MTF1 | hsa-miR-3921 |
| MTF1 | hsa-miR-3961 |
| MTF1 | hsa-miR-599 |
| MTF1 | hsa-miR-6870-5p |
| MTF1 | hsa-miR-6607-5p |
| MTF1 | hsa-miR-7170-3p |
| MTF1 | hsa-miR-4433b-5p |
| MTF1 | hsa-miR-6949-3p |
| MTF1 | hsa-miR-2370-3p |
| MTF1 | hsa-let-7a-3p |
| MTF1 | hsa-miR-103-5p |
| MTF1 | hsa-miR-2345 |
| MTF1 | hsa-miR-1257 |
| MTF1 | hsa-miR-7374a-3p |
| MTF1 | hsa-miR-6794-3p |
| MTF1 | hsa-miR-3474 |
| MTF1 | hsa-miR-3908 |
| MTF1 | hsa-miR-504-5p.1 |
| MTF1 | hsa-miR-7398b-3p |
| MTF1 | hsa-miR-7665-5p |
| MTF1 | hsa-miR-7156-5p |
| MTF1 | hsa-miR-8808 |
| MTF1 | hsa-miR-3124-3p |
| MTF1 | hsa-miR-193-3p |
| MTF1 | hsa-miR-125b-2-3p |
| MTF1 | hsa-miR-890-3p |
| MTF1 | hsa-miR-3970 |
| MTF1 | hsa-miR-8873a |
| MTF1 | hsa-miR-2315 |
| MTF1 | hsa-miR-367-3p |
| MTF1 | hsa-miR-466dk-5p |
| MTF1 | hsa-miR-145-5p |
| MTF1 | hsa-miR-206 |
| MTF1 | hsa-miR-6869-5p |
| MTF1 | hsa-miR-2368-3p |
| MTF1 | hsa-miR-5587-5p |
| MTF1 | hsa-miR-3929 |
| MTF1 | hsa-miR-6827-5p |
| MTF1 | hsa-miR-421-5p |
| MTF1 | hsa-miR-6569-5p |
| MTF1 | hsa-miR-575 |
| MTF1 | hsa-miR-7363-5p |
| MTF1 | hsa-miR-7168-5p |
| MTF1 | hsa-miR-6731-3p |
| MTF1 | hsa-miR-183-5p.2 |
| MTF1 | hsa-miR-7480-5p |
| MTF1 | hsa-miR-1182 |
| MTF1 | hsa-miR-373-3p |
| MTF1 | hsa-miR-3103-5p |
| MTF1 | hsa-miR-370 |
| MTF1 | hsa-miR-4723-3p |
| MTF1 | hsa-miR-2306 |
| MTF1 | hsa-miR-3646 |
| MTF1 | hsa-miR-669j |
| MTF1 | hsa-miR-5006-5p |
| MTF1 | hsa-miR-7172-3p |
| MTF1 | hsa-miR-188-5p |
| MTF1 | hsa-miR-4505 |
| MTF1 | hsa-miR-1191b-3p |
| MTF1 | hsa-miR-26b-3p |
| MTF1 | hsa-miR-5691 |
| MTF1 | hsa-miR-2442 |
| MTF1 | hsa-miR-7028-5p |
| MTF1 | hsa-miR-423-3p |
| MTF1 | hsa-miR-3557-3p |
| MTF1 | hsa-miR-188-5p |
| MTF1 | hsa-miR-6336 |
| MTF1 | hsa-miR-1243-3p |
| MTF1 | hsa-miR-5623-3p |
| MTF1 | hsa-miR-376c-5p |
| MTF1 | hsa-miR-3975 |
| MTF1 | hsa-miR-2488 |
| MTF1 | hsa-miR-1608 |
| MTF1 | hsa-miR-7483-5p |
| MTF1 | hsa-miR-7079-5p |
| MTF1 | hsa-miR-6561-5p |
| MTF1 | hsa-miR-378 |
| MTF1 | hsa-miR-4256 |
| MTF1 | hsa-miR-200ab-5p |
| MTF1 | hsa-miR-181b-1-3p |
| MTF1 | hsa-miR-6133 |
| MTF1 | hsa-miR-1811 |
| MTF1 | hsa-miR-7199-5p |
| MTF1 | hsa-miR-874-5p |
| MTF1 | hsa-miR-7327-5p |
| MTF1 | hsa-miR-302d-3p |
| MTF1 | hsa-miR-8063 |
| MTF1 | hsa-miR-93-5p |
| MTF1 | hsa-miR-548h-5p |
| MTF1 | hsa-miR-1237-3p |
| MTF1 | hsa-miR-130ac-5p |
| MTF1 | hsa-miR-875-5p |
| MTF1 | hsa-miR-301-3p |
| MTF1 | hsa-miR-548c-3p |
| MTF1 | hsa-miR-6965-5p |
| MTF1 | hsa-miR-4516 |
| MTF1 | hsa-miR-1798-5p |
| MTF1 | hsa-miR-2331-5p |
| MTF1 | hsa-miR-2468 |
| MTF1 | hsa-miR-7069-5p |
| MTF1 | hsa-miR-140-3p.2 |
| MTF1 | hsa-miR-6773-3p |
| MTF1 | hsa-miR-450a-2-3p |
| MTF1 | hsa-miR-4802-3p |
| MTF1 | hsa-miR-7061-5p |
| MTF1 | hsa-miR-181b-3p |
| MTF1 | hsa-miR-181d-5p |
| MTF1 | hsa-miR-3577-3p |
| MTF1 | hsa-miR-185-3p |
| MTF1 | hsa-miR-764-3p |
| MTF1 | hsa-miR-3138 |
| MTF1 | hsa-miR-6842-3p |
| MTF1 | hsa-miR-7460-3p |
| MTF1 | hsa-miR-7035-5p |
| MTF1 | hsa-miR-142-3p.2 |
| MTF1 | hsa-miR-6807-5p |
| MTF1 | hsa-miR-4763-3p |
| MTF1 | hsa-miR-548c-3p |
| MTF1 | hsa-miR-6605-5p |
| MTF1 | hsa-miR-604 |
| MTF1 | hsa-miR-7269-5p |
| MTF1 | hsa-miR-3594-3p |
| MTF1 | hsa-miR-7283-5p |
| MTF1 | hsa-miR-7169-5p |
| MTF1 | hsa-miR-3664-5p |
| MTF1 | hsa-miR-548k |
| MTF1 | hsa-miR-520e |
| MTF1 | hsa-miR-1839 |
| MTF1 | hsa-miR-299-3p |
| MTF1 | hsa-miR-3154 |
| MTF1 | hsa-miR-553 |
| MTF1 | hsa-miR-382-5p |
| MTF1 | hsa-miR-4709-3p |
| MTF1 | hsa-miR-3087-3p |
| MTF1 | hsa-miR-465-5p |
| MTF1 | hsa-miR-7213-3p |
| MTF1 | hsa-miR-6927-3p |
| MTF1 | hsa-miR-145-5p |
| MTF1 | hsa-miR-2117 |
| MTF1 | hsa-miR-7b-3p |
| MTF1 | hsa-miR-1179 |
| MTF1 | hsa-miR-3125 |
| MTF1 | hsa-miR-1305 |
| MTF1 | hsa-miR-494 |
| MTF1 | hsa-miR-510-3p |
| MTF1 | hsa-miR-3173-3p |
| MTF1 | hsa-miR-2446 |
| MTF1 | hsa-miR-6481 |
| MTF1 | hsa-miR-7238-3p |
| MTF1 | hsa-miR-7314-5p |
| MTF1 | hsa-miR-7651-5p |
| MTF1 | hsa-miR-3064 |
| MTF1 | hsa-miR-1247-5p |
| MTF1 | hsa-miR-1178-5p |
| MTF1 | hsa-miR-669g |
| MTF1 | hsa-miR-7371a-5p |
| MTF1 | hsa-miR-6718-5p |
| MTF1 | hsa-miR-3583-3p |
| MTF1 | hsa-miR-3128 |
| MTF1 | hsa-miR-579-3p |
| MTF1 | hsa-miR-7670-5p |
| MTF1 | hsa-miR-695 |
| MTF1 | hsa-miR-6845-3p |
| MTF1 | hsa-miR-7187-3p |
| MTF1 | hsa-miR-6990-5p |
| MTF1 | hsa-miR-4778-3p |
| MTF1 | hsa-miR-302b-5p |
| MTF1 | hsa-miR-4639-3p |
| MTF1 | hsa-miR-7335-3p |
| MTF1 | hsa-miR-6631-5p |
| MTF1 | hsa-miR-202-3p |
| MTF1 | hsa-miR-6970-5p |
| MTF1 | hsa-miR-1237-3p |
| MTF1 | hsa-miR-4282 |
| MTF1 | hsa-miR-3176 |
| MTF1 | hsa-miR-4529-5p |
| MTF1 | hsa-miR-505-5p |
| MTF1 | hsa-miR-185-5p |
| MTF1 | hsa-miR-2404 |
| MTF1 | hsa-miR-455-5p |
| MTF1 | hsa-miR-8881 |
| MTF1 | hsa-miR-4735-5p |
| MTF1 | hsa-miR-769-3p |
| MTF1 | hsa-miR-486-5p |
| MTF1 | hsa-miR-362-5p |
| MTF1 | hsa-miR-6700-5p |
| MTF1 | hsa-miR-548au-3p |
| MTF1 | hsa-miR-1190 |
| MTF1 | hsa-miR-520-5p |
| MTF1 | hsa-miR-2377 |
| MTF1 | hsa-miR-2470 |
| MTF1 | hsa-miR-599-5p |
| MTF1 | hsa-miR-1271-5p |
| MTF1 | hsa-miR-4708-3p |
| MTF1 | hsa-miR-5134-5p |
| MTF1 | hsa-miR-4720-3p |
| MTF1 | hsa-miR-6512-5p |
| MTF1 | hsa-miR-7309-5p |
| MTF1 | hsa-miR-376a-2-5p |
| MTF1 | hsa-miR-7269-3p |
| MTF1 | hsa-miR-7206-5p |
| MTF1 | hsa-miR-642 |
| MTF1 | hsa-miR-548i-3p |
| MTF1 | hsa-miR-4775 |
| MTF1 | hsa-miR-934-3p |
| MTF1 | hsa-miR-448-5p |
| MTF1 | hsa-miR-4310 |
| MTF1 | hsa-miR-5131 |
| MTF1 | hsa-miR-6883-3p |
| MTF1 | hsa-miR-1236-5p |
| MTF1 | hsa-miR-1255b-2-3p |
| MTF1 | hsa-miR-7332-3p |
| MTF1 | hsa-miR-7090-3p |
| MTF1 | hsa-miR-1712-3p |
| MTF1 | hsa-miR-6784-3p |
| MTF1 | hsa-miR-6754-3p |
| MTF1 | hsa-miR-8793 |
| MTF1 | hsa-miR-3190-3p |
| MTF1 | hsa-miR-8485 |
| MTF1 | hsa-let-7abf-3p |
| MTF1 | hsa-miR-3120-3p |
| MTF1 | hsa-miR-6508-5p |
| MTF1 | hsa-miR-2053 |
| MTF1 | hsa-miR-6922-3p |
| MTF1 | hsa-miR-3620-3p |
| MTF1 | hsa-miR-6922-5p |
| MTF1 | hsa-miR-1964-3p |
| MTF1 | hsa-miR-6398 |
| MTF1 | hsa-miR-4683 |
| MTF1 | hsa-miR-7371b-3p |
| MTF1 | hsa-miR-1651-3p |
| MTF1 | hsa-miR-140-3p.1 |
| MTF1 | hsa-miR-3925-5p |
| MTF1 | hsa-miR-5089-5p |
| MTF1 | hsa-miR-501-5p |
| MTF1 | hsa-miR-6904-5p |
| MTF1 | hsa-miR-4280 |
| MTF1 | hsa-miR-126-3p |
| MTF1 | hsa-miR-5692a |
| MTF1 | hsa-miR-1667-3p |
| MTF1 | hsa-miR-623 |
| MTF1 | hsa-miR-3076-5p |
| MTF1 | hsa-miR-7057-3p |
| MTF1 | hsa-miR-487-5p |
| MTF1 | hsa-miR-1643a-5p |
| MTF1 | hsa-miR-350 |
| MTF1 | hsa-miR-429-3p |
| MTF1 | hsa-miR-4696 |
| MTF1 | hsa-miR-3549 |
| MTF1 | hsa-miR-1639 |
| MTF1 | hsa-miR-2352 |
| MTF1 | hsa-miR-1911-3p |
| MTF1 | hsa-miR-8888 |
| MTF1 | hsa-miR-3575 |
| MTF1 | hsa-miR-512-5p |
| MTF1 | hsa-miR-876 |
| MTF1 | hsa-miR-7160-3p |
| MTF1 | hsa-miR-548e-5p |
| MTF1 | hsa-miR-7164-5p |
| MTF1 | hsa-miR-6984-3p |
| MTF1 | hsa-miR-6793-5p |
| MTF1 | hsa-miR-5101 |
| MTF1 | hsa-miR-6847-3p |
| MTF1 | hsa-miR-192-5p |
| MTF1 | hsa-miR-3146 |
| MTF1 | hsa-miR-7385-5p |
| MTF1 | hsa-miR-6669-3p |
| MTF1 | hsa-miR-29a-5p |
| MTF1 | hsa-miR-301 |
| MTF1 | hsa-miR-1457 |
| MTF1 | hsa-miR-410-5p |
| MTF1 | hsa-miR-150-5p |
| MTF1 | hsa-miR-2367-5p |
| MTF1 | hsa-miR-6791-5p |
| MTF1 | hsa-miR-4719 |
| MTF1 | hsa-miR-7234-5p |
| MTF1 | hsa-miR-3473g |
| MTF1 | hsa-miR-669n |
| MTF1 | hsa-miR-192-3p |
| MTF1 | hsa-miR-8068 |
| MTF1 | hsa-miR-3530-3p |
| MTF1 | hsa-miR-520f-3p |
| MTF1 | hsa-miR-2464-3p |
| MTF1 | hsa-miR-6751-5p |
| MTF1 | hsa-miR-7246-5p |
| MTF1 | hsa-miR-6388 |
| MTF1 | hsa-miR-330-5p |
| MTF1 | hsa-miR-7352-3p |
| MTF1 | hsa-miR-1249-3p |
| MTF1 | hsa-miR-6894-5p |
| MTF1 | hsa-miR-2897 |
| MTF1 | hsa-miR-548b-3p |
| MTF1 | hsa-miR-1626-5p |
| MTF1 | hsa-miR-7373-5p |
| MTF1 | hsa-miR-2284k |
| MTF1 | hsa-miR-7111-5p |
| MTF1 | hsa-miR-548ag |
| MTF1 | hsa-miR-138-1-3p |
| MTF1 | hsa-miR-484 |
| MTF1 | hsa-miR-545-5p |
| MTF1 | hsa-miR-5618-5p |
| MTF1 | hsa-miR-7042-5p |
| MTF1 | hsa-miR-7006-3p |
| MTF1 | hsa-miR-1468-3p |
| MTF1 | hsa-miR-2367-3p |
| MTF1 | hsa-miR-7397-3p |
| MTF1 | hsa-miR-7252-3p |
| MTF1 | hsa-miR-548aw |
| MTF1 | hsa-miR-7367-5p |
| MTF1 | hsa-miR-1968-3p |
| MTF1 | hsa-miR-149-3p |
| MTF1 | hsa-miR-373-5p |
| MTF1 | hsa-miR-7004-3p |
| MTF1 | hsa-miR-4761-3p |
| MTF1 | hsa-miR-140-5p |
| MTF1 | hsa-miR-766-3p |
| MTF1 | hsa-miR-3692-5p |
| MTF1 | hsa-miR-7359-3p |
| MTF1 | hsa-miR-2110 |
| MTF1 | hsa-miR-4270 |
| MTF1 | hsa-miR-299-5p |
| MTF1 | hsa-miR-3547-5p |
| MTF1 | hsa-miR-1976 |
| MTF1 | hsa-miR-181a-2-3p |
| MTF1 | hsa-miR-2284v |
| MTF1 | hsa-miR-7057-5p |
| MTF1 | hsa-miR-653 |
| MTF1 | hsa-miR-676-3p |
| MTF1 | hsa-miR-8054 |
| MTF1 | hsa-miR-5007-3p |
| MTF1 | hsa-miR-371b-5p |
| MTF1 | hsa-miR-193b-5p |
| MTF1 | hsa-miR-6517 |
| MTF1 | hsa-miR-549a |
| MTF1 | hsa-miR-323b-5p |
| MTF1 | hsa-miR-548ay-5p |
| MTF1 | hsa-miR-544-3p |
| MTF1 | hsa-miR-664a-5p |
| MTF1 | hsa-miR-1301 |
| MTF1 | hsa-miR-93-3p |
| MTF1 | hsa-miR-2381 |
| MTF1 | hsa-miR-6529-5p |
| MTF1 | hsa-miR-2898 |
| MTF1 | hsa-miR-1677-5p |
| MTF1 | hsa-miR-1239 |
| MTF1 | hsa-miR-7341-3p |
| MTF1 | hsa-miR-7383-5p |
| MTF1 | hsa-miR-670 |
| MTF1 | hsa-miR-6877-3p |
| MTF1 | hsa-miR-5626-5p |
| MTF1 | hsa-miR-7054-5p |
| MTF1 | hsa-miR-6778-3p |
| MTF1 | hsa-miR-7350-5p |
| MTF1 | hsa-miR-7859 |
| MTF1 | hsa-miR-501-5p |
| MTF1 | hsa-miR-3127-5p |
| MTF1 | hsa-miR-211-5p |
| MTF1 | hsa-miR-7109-3p |
| MTF1 | hsa-miR-9500 |
| MTF1 | hsa-miR-713 |
| MTF1 | hsa-miR-329-5p |
| MTF1 | hsa-miR-1343-3p |
| MTF1 | hsa-miR-2284z |
| MTF1 | hsa-miR-7026-5p |
| MTF1 | hsa-miR-6829-3p |
| MTF1 | hsa-let-7afk-3p |
| MTF1 | hsa-miR-6334 |
| MTF1 | hsa-miR-4755-3p |
| MTF1 | hsa-miR-8885 |
| MTF1 | hsa-miR-3919 |
| MTF1 | hsa-miR-516a |
| MTF1 | hsa-miR-224-3p |
| MTF1 | hsa-miR-3153 |
| MTF1 | hsa-miR-542-5p |
| MTF1 | hsa-miR-7053-3p |
| MTF1 | hsa-miR-677 |
| MTF1 | hsa-miR-221-5p |
| MTF1 | hsa-miR-7235-5p |
| MTF1 | hsa-miR-218-5p |
| MTF1 | hsa-miR-30c-3p |
| MTF1 | hsa-miR-7119-3p |
| MTF1 | hsa-miR-19-5p |
| MTF1 | hsa-miR-548aef |
| MTF1 | hsa-miR-7461-5p |
| MTF1 | hsa-miR-338-5p |
| MTF1 | hsa-miR-7201-3p |
| MTF1 | hsa-miR-6499-3p |
| MTF1 | hsa-miR-191-5p |
| MTF1 | hsa-miR-6081 |
| MTF1 | hsa-miR-17-5p |
| MTF1 | hsa-miR-4423-5p |
| MTF1 | hsa-miR-7657-3p |
| MTF1 | hsa-miR-1662 |
| MTF1 | hsa-miR-3090-5p |
| MTF1 | hsa-miR-3084-5p |
| MTF1 | hsa-miR-217-3p |
| MTF1 | hsa-miR-7162-5p |
| MTF1 | hsa-miR-8077 |
| MTF1 | hsa-miR-6946-5p |
| MTF1 | hsa-miR-3132 |
| MTF1 | hsa-miR-7110-3p |
| MTF1 | hsa-miR-6855-5p |
| MTF1 | hsa-miR-135a-1-3p |
| MTF1 | hsa-miR-580 |
| MTF1 | hsa-miR-1659 |
| MTF1 | hsa-miR-2334 |
| MTF1 | hsa-let-7-5p |
| MTF1 | hsa-miR-6871-3p |
| MTF1 | hsa-miR-412-3p |
| MTF1 | hsa-miR-6931-5p |
| MTF1 | hsa-miR-4645-5p |
| MTF1 | hsa-miR-7239-5p |
| MTF1 | hsa-let-7f-1-3p |
| MTF1 | hsa-miR-1253 |
| MTF1 | hsa-miR-6715b-5p |
| MTF1 | hsa-miR-6945-3p |
| MTF1 | hsa-miR-2368-5p |
| MTF1 | hsa-miR-6685-3p |
| MTF1 | hsa-miR-130b-5p |
| MTF1 | hsa-miR-34b-5p |
| MTF1 | hsa-miR-514a-3p |
| MTF1 | hsa-miR-2347 |
[truncated: 208,761 more chars]
