# Supplementary material for: Cuproptosis‐related miRNAs signature and immune infiltration characteristics in colorectal cancer
Source: Cancer Med. 2023 Jun 19;12(15):16661–78. doi: 10.1002/cam4.6270 (PMC10469834; doi:10.1002/cam4.6270)
Supplement: Supplementary file 3 — Table S2 [file CAM4-12-16661-s006.docx]

TABLE S2. The 72 differentially expressed miRNAs between CRC tissues and normal tissues.

| ID | logFC | logCPM | PValue | FDR |
| --- | --- | --- | --- | --- |
| hsa-miR-592 | 5.548649 | 13.85074 | 1.43E-20 | 4.25E-18 |
| hsa-miR-577 | 3.535695 | 14.11237 | 1.48E-20 | 4.25E-18 |
| hsa-miR-889 | 4.104854 | 13.78413 | 1.34E-16 | 2.57E-14 |
| hsa-miR-450b | 5.228565 | 13.61612 | 3.74E-16 | 5.38E-14 |
| hsa-miR-504 | -2.13446 | 12.56464 | 7.17E-16 | 8.26E-14 |
| hsa-miR-454 | 4.239095 | 13.61469 | 7.59E-15 | 7.29E-13 |
| hsa-miR-1224 | -2.04386 | 12.62011 | 1.06E-14 | 8.73E-13 |
| hsa-miR-542 | 1.959115 | 14.31334 | 1.46E-13 | 1.05E-11 |
| hsa-miR-885 | -2.72518 | 12.12789 | 1.79E-13 | 1.14E-11 |
| hsa-miR-653 | 4.938379 | 13.416 | 4.45E-13 | 2.56E-11 |
| hsa-miR-376c | 4.033656 | 13.47183 | 1.85E-12 | 9.69E-11 |
| hsa-miR-142 | 1.294353 | 14.76267 | 8.37E-12 | 4.02E-10 |
| hsa-miR-217 | 2.159357 | 13.95053 | 2.59E-11 | 1.15E-09 |
| hsa-miR-552 | 1.625679 | 14.295 | 3.87E-11 | 1.59E-09 |
| hsa-miR-421 | 3.121783 | 13.42149 | 1.20E-10 | 4.61E-09 |
| hsa-miR-651 | 3.359029 | 13.27031 | 9.26E-10 | 3.33E-08 |
| hsa-miR-1245a | 4.277208 | 13.00951 | 5.30E-09 | 1.80E-07 |
| hsa-miR-598 | 1.940579 | 13.72417 | 1.94E-08 | 6.21E-07 |
| hsa-miR-549a | 4.161846 | 12.94608 | 2.24E-08 | 6.80E-07 |
| hsa-miR-215 | 1.090654 | 14.55693 | 2.70E-08 | 7.78E-07 |
| hsa-miR-496 | 3.005906 | 13.18382 | 1.02E-07 | 2.81E-06 |
| hsa-miR-627 | 2.70466 | 13.1565 | 1.92E-07 | 4.66E-06 |
| hsa-miR-95 | 1.682242 | 13.75904 | 1.94E-07 | 4.66E-06 |
| hsa-miR-655 | 3.41942 | 13.08378 | 2.24E-07 | 5.15E-06 |
| hsa-miR-4791 | 3.953901 | 12.83753 | 2.33E-07 | 5.17E-06 |
| hsa-miR-7705 | 3.946434 | 12.83376 | 4.01E-07 | 8.56E-06 |
| hsa-miR-5683 | 3.877168 | 12.79985 | 1.20E-06 | 2.46E-05 |
| hsa-miR-346 | -2.14142 | 12.10035 | 1.56E-06 | 3.11E-05 |
| hsa-miR-605 | -1.39811 | 12.59948 | 2.45E-06 | 4.55E-05 |
| hsa-miR-3662 | 3.772911 | 12.74912 | 2.55E-06 | 4.59E-05 |
| hsa-miR-643 | 3.75866 | 12.74241 | 2.82E-06 | 4.93E-05 |
| hsa-miR-7702 | -1.31701 | 12.74095 | 3.19E-06 | 5.25E-05 |
| hsa-miR-380 | 3.771222 | 12.74832 | 3.35E-06 | 5.35E-05 |
| hsa-miR-494 | 2.741729 | 13.02873 | 5.65E-06 | 8.80E-05 |
| hsa-miR-4634 | -2.30528 | 11.9286 | 1.07E-05 | 0.000159 |
| hsa-miR-559 | 3.630899 | 12.68373 | 1.08E-05 | 0.000159 |
| hsa-miR-1343 | -1.35659 | 12.59253 | 1.30E-05 | 0.000188 |
| hsa-miR-656 | 3.59016 | 12.66561 | 1.51E-05 | 0.000213 |
| hsa-miR-1260b | -2.08806 | 12.02405 | 1.74E-05 | 0.000238 |
| hsa-miR-580 | 3.558687 | 12.65181 | 2.13E-05 | 0.000285 |
| hsa-miR-216a | 2.668722 | 12.87373 | 2.61E-05 | 0.000342 |
| hsa-miR-412 | 1.605403 | 13.36964 | 2.85E-05 | 0.000365 |
| hsa-miR-767 | -1.12137 | 12.80947 | 6.50E-05 | 0.000814 |
| hsa-miR-493 | 1.14567 | 13.75977 | 6.70E-05 | 0.000821 |
| hsa-miR-6516 | 3.38761 | 12.57971 | 7.85E-05 | 0.000942 |
| hsa-miR-7974 | 2.289945 | 12.7915 | 0.000148 | 0.001708 |
| hsa-miR-1255a | 3.270069 | 12.53302 | 0.00019 | 0.002143 |
| hsa-miR-206 | 3.246367 | 12.52399 | 0.000216 | 0.002397 |
| hsa-miR-5706 | 3.223078 | 12.51499 | 0.000276 | 0.002995 |
| hsa-miR-6125 | -1.29411 | 12.45497 | 0.000317 | 0.003315 |
| hsa-miR-466 | 3.122572 | 12.47776 | 0.000529 | 0.005252 |
| hsa-miR-3154 | -1.53398 | 12.1311 | 0.000839 | 0.008052 |
| hsa-miR-3684 | 3.01786 | 12.44045 | 0.001 | 0.009294 |
| hsa-miR-3941 | 2.360658 | 12.5644 | 0.001182 | 0.010807 |
| hsa-miR-3654 | 2.948055 | 12.41665 | 0.00136 | 0.012243 |
| hsa-miR-4437 | -1.65421 | 11.93185 | 0.001422 | 0.012605 |
| hsa-miR-5696 | 2.960181 | 12.42069 | 0.001503 | 0.012922 |
| hsa-miR-5684 | 2.941181 | 12.4143 | 0.001529 | 0.01295 |
| hsa-miR-665 | 2.930401 | 12.4107 | 0.001751 | 0.014617 |
| hsa-miR-3132 | -1.6129 | 11.93472 | 0.002404 | 0.019232 |
| hsa-miR-4651 | -1.75783 | 12.01432 | 0.00255 | 0.020124 |
| hsa-miR-1265 | -1.74648 | 11.93695 | 0.003016 | 0.023474 |
| hsa-miR-581 | 2.078429 | 12.59947 | 0.003275 | 0.025149 |
| hsa-miR-641 | 2.782866 | 12.36318 | 0.003757 | 0.028142 |
| hsa-miR-548v | 1.455718 | 12.82168 | 0.003762 | 0.028142 |
| hsa-miR-1284 | 2.780586 | 12.36247 | 0.003887 | 0.028703 |
| hsa-miR-3656 | -1.68065 | 11.95808 | 0.003953 | 0.028823 |
| hsa-miR-3138 | -1.30144 | 12.17664 | 0.004545 | 0.032724 |
| hsa-miR-1295a | -1.05194 | 12.35564 | 0.00623 | 0.043761 |
| hsa-miR-5193 | -1.33272 | 12.1085 | 0.006405 | 0.044113 |
| hsa-miR-573 | 2.074693 | 12.45674 | 0.006433 | 0.044113 |
| hsa-miR-3174 | 2.652313 | 12.32383 | 0.007281 | 0.048207 |
