# Supplementary material for: Cuproptosis‐related miRNAs signature and immune infiltration characteristics in colorectal cancer
Source: Cancer Med. 2023 Jun 19;12(15):16661–78. doi: 10.1002/cam4.6270 (PMC10469834; doi:10.1002/cam4.6270)
Supplement: Supplementary file 4 — Table S3 [file CAM4-12-16661-s003.docx]

TABLE S3 The results of multivariate Cox regression.

| id | coef |
| --- | --- |
| hsa-miR-653 | 0.668792 |
| hsa-miR-552 | -0.87966 |
| hsa-miR-216a | 0.58155 |
| hsa-miR-3684 | 0.482573 |
| hsa-miR-4437 | 2.128164 |
| hsa-miR-641 | 0.388606 |
