# Supplementary material for: Cuproptosis‐related miRNAs signature and immune infiltration characteristics in colorectal cancer
Source: Cancer Med. 2023 Jun 19;12(15):16661–78. doi: 10.1002/cam4.6270 (PMC10469834; doi:10.1002/cam4.6270)
Supplement: Supplementary file 5 — Table S4 [file CAM4-12-16661-s007.docx]

TABLE S4 The risk score and risk group of all the samples.

| id | riskScore | risk |
| --- | --- | --- |
| TCGA-CM-5863 | 0.709197 | low |
| TCGA-A6-6142 | 1.840316 | high |
| TCGA-AZ-5403 | 2.109404 | high |
| TCGA-AA-3662 | 3.222367 | high |
| TCGA-A6-5656 | 0.4741 | low |
| TCGA-CM-6167 | 0.974638 | low |
| TCGA-CA-5256 | 0.623011 | low |
| TCGA-AA-A02H | 1.259084 | high |
| TCGA-AU-3779 | 1.76211 | high |
| TCGA-AZ-6600 | 3.415259 | high |
| TCGA-AA-3877 | 1.08924 | high |
| TCGA-D5-6922 | 1.699523 | high |
| TCGA-AA-3495 | 1.265135 | high |
| TCGA-AA-3519 | 0.23945 | low |
| TCGA-DM-A28C | 0.675994 | low |
| TCGA-G4-6293 | 0.518166 | low |
| TCGA-A6-5665 | 0.5836 | low |
| TCGA-CA-6718 | 1.351473 | high |
| TCGA-A6-5659 | 1.885213 | high |
| TCGA-NH-A50U | 5.972589 | high |
| TCGA-AA-A01F | 0.255434 | low |
| TCGA-AY-4070 | 1.303257 | high |
| TCGA-AZ-6608 | 1.615887 | high |
| TCGA-CK-5916 | 1.167639 | high |
| TCGA-AA-3664 | 0.359187 | low |
| TCGA-AA-3554 | 1.302152 | high |
| TCGA-AY-A8YK | 1.212711 | high |
| TCGA-AA-3494 | 0.808314 | low |
| TCGA-AA-3864 | 0.440886 | low |
| TCGA-AA-3555 | 0.395347 | low |
| TCGA-AA-3713 | 0.264011 | low |
| TCGA-AZ-6599 | 24.50453 | high |
| TCGA-A6-5664 | 0.178623 | low |
| TCGA-AA-3531 | 0.198387 | low |
| TCGA-F4-6704 | 2.667528 | high |
| TCGA-AZ-6605 | 0.637496 | low |
| TCGA-AA-A00L | 0.417757 | low |
| TCGA-NH-A8F8 | 2.255999 | high |
| TCGA-5M-AATE | 1.692328 | high |
| TCGA-AA-3977 | 0.487696 | low |
| TCGA-DM-A28M | 0.64571 | low |
| TCGA-A6-2683 | 11.34377 | high |
| TCGA-DM-A1D0 | 2.281065 | high |
| TCGA-DM-A1D4 | 0.602938 | low |
| TCGA-DM-A28K | 0.903512 | low |
| TCGA-AA-3522 | 1.069709 | high |
| TCGA-DM-A28F | 2.157614 | high |
| TCGA-DM-A285 | 1.099909 | high |
| TCGA-A6-2674 | 0.394498 | low |
| TCGA-A6-4105 | 2.177097 | high |
| TCGA-AA-3930 | 1.03407 | high |
| TCGA-AA-3842 | 0.460425 | low |
| TCGA-CM-6161 | 0.947537 | low |
| TCGA-F4-6809 | 1.366003 | high |
| TCGA-CA-6717 | 3.943972 | high |
| TCGA-DM-A28G | 2.5641 | high |
| TCGA-A6-3809 | 0.601263 | low |
| TCGA-A6-5667 | 3.262479 | high |
| TCGA-CA-5254 | 0.338831 | low |
| TCGA-A6-6654 | 0.931974 | low |
| TCGA-G4-6317 | 2.156462 | high |
| TCGA-G4-6295 | 0.689662 | low |
| TCGA-A6-6652 | 1.733295 | high |
| TCGA-AA-3866 | 0.925899 | low |
| TCGA-F4-6459 | 4.107302 | high |
| TCGA-AA-3542 | 0.855093 | low |
| TCGA-DM-A1HB | 0.247382 | low |
| TCGA-A6-5666 | 0.945345 | low |
| TCGA-AA-3956 | 0.781314 | low |
| TCGA-A6-2672 | 0.890075 | low |
| TCGA-F4-6856 | 0.505179 | low |
| TCGA-CM-4748 | 1.478711 | high |
| TCGA-A6-2681 | 0.567321 | low |
| TCGA-AY-A69D | 1.152583 | high |
| TCGA-AA-3544 | 0.383274 | low |
| TCGA-AZ-4315 | 0.663928 | low |
| TCGA-AA-3489 | 2.142834 | high |
| TCGA-AA-3710 | 0.419598 | low |
| TCGA-D5-6926 | 0.318083 | low |
| TCGA-AA-3553 | 1.991615 | high |
| TCGA-AA-A01V | 0.412504 | low |
| TCGA-AA-3520 | 0.358275 | low |
| TCGA-DM-A28H | 0.721164 | low |
| TCGA-A6-5657 | 1.796696 | high |
| TCGA-D5-6930 | 1.76146 | high |
| TCGA-AA-3860 | 0.347732 | low |
| TCGA-AA-3526 | 0.657329 | low |
| TCGA-AA-A01S | 0.743324 | low |
| TCGA-CM-5860 | 0.698257 | low |
| TCGA-AA-3530 | 0.505466 | low |
| TCGA-D5-6536 | 0.781771 | low |
| TCGA-D5-6538 | 2.853338 | high |
| TCGA-A6-5660 | 2.063403 | high |
| TCGA-A6-2677 | 1.58508 | high |
| TCGA-AZ-4313 | 0.226173 | low |
| TCGA-AA-3854 | 0.796616 | low |
| TCGA-AA-3655 | 0.682855 | low |
| TCGA-A6-A567 | 1.548706 | high |
| TCGA-CM-6171 | 0.478996 | low |
| TCGA-G4-6294 | 2.423968 | high |
| TCGA-AA-3952 | 1.219539 | high |
| TCGA-DM-A288 | 1.441978 | high |
| TCGA-AY-5543 | 0.736313 | low |
| TCGA-AA-A00D | 0.260737 | low |
| TCGA-F4-6806 | 0.762735 | low |
| TCGA-AY-A54L | 1.454572 | high |
| TCGA-CM-6165 | 1.704252 | high |
| TCGA-AA-A01P | 2.869811 | high |
| TCGA-A6-2675 | 1.558677 | high |
| TCGA-CM-5344 | 0.591248 | low |
| TCGA-AA-3973 | 1.641078 | high |
| TCGA-AA-A01X | 0.840237 | low |
| TCGA-CM-5349 | 0.511978 | low |
| TCGA-A6-6648 | 0.576316 | low |
| TCGA-AA-3867 | 1.398259 | high |
| TCGA-F4-6808 | 1.837453 | high |
| TCGA-CM-6677 | 2.513482 | high |
| TCGA-AA-3561 | 0.349628 | low |
| TCGA-G4-6628 | 2.887021 | high |
| TCGA-AU-6004 | 1.169684 | high |
| TCGA-D5-6533 | 0.500748 | low |
| TCGA-CM-6172 | 0.617591 | low |
| TCGA-AA-3666 | 3.072253 | high |
| TCGA-CM-6678 | 0.691233 | low |
| TCGA-D5-6928 | 2.761005 | high |
| TCGA-AA-3939 | 0.391629 | low |
| TCGA-CM-6164 | 0.836214 | low |
| TCGA-CK-5914 | 0.248227 | low |
| TCGA-AA-A024 | 0.90751 | low |
| TCGA-NH-A50V | 1.314395 | high |
| TCGA-AA-A02F | 1.503093 | high |
| TCGA-AA-3855 | 1.4675 | high |
| TCGA-DM-A28E | 0.59521 | low |
| TCGA-DM-A280 | 2.886 | high |
| TCGA-D5-6535 | 1.114011 | high |
| TCGA-D5-6932 | 1.627452 | high |
| TCGA-DM-A1DA | 1.327459 | high |
| TCGA-AA-3532 | 0.836373 | low |
| TCGA-AA-A01T | 0.253691 | low |
| TCGA-G4-6321 | 1.243337 | high |
| TCGA-G4-6310 | 0.516862 | low |
| TCGA-A6-A565 | 1.161958 | high |
| TCGA-AA-3875 | 0.429812 | low |
| TCGA-CK-4951 | 2.108202 | high |
| TCGA-A6-3808 | 2.666666 | high |
| TCGA-CM-6170 | 1.472708 | high |
| TCGA-A6-6780 | 1.992687 | high |
| TCGA-AA-3833 | 1.335129 | high |
| TCGA-F4-6460 | 2.289591 | high |
| TCGA-A6-6653 | 0.374963 | low |
| TCGA-A6-A56B | 1.8811 | high |
| TCGA-AA-3811 | 7.132883 | high |
| TCGA-A6-2679 | 0.258396 | low |
| TCGA-AA-3966 | 3.722256 | high |
| TCGA-CK-4950 | 0.911279 | low |
| TCGA-AA-3688 | 1.002275 | high |
| TCGA-4T-AA8H | 3.315988 | high |
| TCGA-AY-6197 | 0.312498 | low |
| TCGA-AA-3548 | 0.609291 | low |
| TCGA-AA-A010 | 0.240643 | low |
| TCGA-AA-3982 | 0.765749 | low |
| TCGA-G4-6315 | 1.228171 | high |
| TCGA-AA-3812 | 0.699224 | low |
| TCGA-AA-3509 | 0.292517 | low |
| TCGA-4N-A93T | 1.528132 | high |
| TCGA-G4-6311 | 1.681163 | high |
| TCGA-G4-6303 | 2.498317 | high |
| TCGA-A6-6651 | 4.183568 | high |
| TCGA-G4-6306 | 4.471489 | high |
| TCGA-AA-3549 | 0.384781 | low |
| TCGA-QG-A5Z2 | 0.92527 | low |
| TCGA-AA-3856 | 1.248622 | high |
| TCGA-NH-A6GC | 0.855607 | low |
| TCGA-AA-3543 | 0.579395 | low |
| TCGA-DM-A1HA | 1.648477 | high |
| TCGA-AA-3821 | 0.635049 | low |
| TCGA-AA-3518 | 0.648711 | low |
| TCGA-G4-6586 | 2.64802 | high |
| TCGA-AD-6548 | 1.433012 | high |
| TCGA-A6-2676 | 1.010898 | high |
| TCGA-AA-3514 | 1.060776 | high |
| TCGA-AA-3818 | 0.93249 | low |
| TCGA-AA-3858 | 0.206493 | low |
| TCGA-AD-6895 | 3.576854 | high |
| TCGA-AD-6889 | 0.872279 | low |
| TCGA-AA-3560 | 0.320746 | low |
| TCGA-AA-3680 | 1.515904 | high |
| TCGA-NH-A6GB | 0.463057 | low |
| TCGA-G4-6323 | 0.675229 | low |
| TCGA-A6-6138 | 1.242509 | high |
| TCGA-QG-A5YV | 0.626084 | low |
| TCGA-AZ-6607 | 2.130483 | high |
| TCGA-AA-A02R | 2.100334 | high |
| TCGA-AZ-4614 | 0.267552 | low |
| TCGA-AZ-6601 | 1.27289 | high |
| TCGA-G4-6627 | 1.269795 | high |
| TCGA-AA-3870 | 0.315727 | low |
| TCGA-AZ-6606 | 1.080544 | high |
| TCGA-CM-6166 | 1.16149 | high |
| TCGA-AA-A00U | 0.405423 | low |
| TCGA-AA-3552 | 1.074313 | high |
| TCGA-G4-6320 | 0.325849 | low |
| TCGA-A6-3807 | 0.645987 | low |
| TCGA-D5-5540 | 0.323384 | low |
| TCGA-WS-AB45 | 1.536613 | high |
| TCGA-AA-3673 | 0.942632 | low |
| TCGA-CA-6719 | 0.781343 | low |
| TCGA-T9-A92H | 0.872741 | low |
| TCGA-AG-3731 | 2.130049 | high |
| TCGA-F5-6861 | 0.539445 | low |
| TCGA-AG-3894 | 1.666178 | high |
| TCGA-AG-3885 | 0.698199 | low |
| TCGA-EI-6508 | 3.130734 | high |
| TCGA-EI-6509 | 1.486879 | high |
| TCGA-AG-4022 | 1.758816 | high |
| TCGA-F5-6863 | 2.27468 | high |
| TCGA-CI-6623 | 1.107073 | high |
| TCGA-CI-6620 | 4.423898 | high |
| TCGA-CI-6619 | 3.794596 | high |
| TCGA-EI-6917 | 2.99154 | high |
| TCGA-AG-3898 | 0.263354 | low |
| TCGA-AG-3728 | 0.540121 | low |
| TCGA-DY-A1DG | 2.402937 | high |
| TCGA-AG-A011 | 0.328304 | low |
| TCGA-AG-A00C | 0.763575 | low |
| TCGA-EI-6507 | 0.614316 | low |
| TCGA-AG-3878 | 0.256134 | low |
| TCGA-AG-3887 | 2.560376 | high |
| TCGA-AG-3575 | 3.81656 | high |
| TCGA-F5-6814 | 0.461696 | low |
| TCGA-EI-6884 | 8.002492 | high |
| TCGA-EF-5830 | 1.216447 | high |
| TCGA-AG-3612 | 0.786471 | low |
| TCGA-AG-3574 | 0.825029 | low |
| TCGA-EI-6512 | 0.490767 | low |
| TCGA-AF-4110 | 1.702129 | high |
| TCGA-AG-A02G | 0.36837 | low |
| TCGA-BM-6198 | 1.425585 | high |
| TCGA-AF-2692 | 0.46311 | low |
| TCGA-AG-3892 | 0.884799 | low |
| TCGA-EI-6881 | 0.194092 | low |
| TCGA-AA-3679 | 1.930106 | high |
| TCGA-AD-6901 | 2.343886 | high |
| TCGA-AZ-6598 | 0.581533 | low |
| TCGA-F4-6570 | 0.958385 | low |
| TCGA-AA-3667 | 1.211495 | high |
| TCGA-AA-3989 | 2.395214 | high |
| TCGA-D5-6530 | 1.253289 | high |
| TCGA-G4-6299 | 2.939303 | high |
| TCGA-AA-A00W | 0.289912 | low |
| TCGA-CM-6679 | 1.125268 | high |
| TCGA-D5-6529 | 0.995387 | high |
| TCGA-AY-A71X | 0.661203 | low |
| TCGA-A6-6650 | 2.454931 | high |
| TCGA-AA-3949 | 3.679445 | high |
| TCGA-A6-5662 | 0.457109 | low |
| TCGA-AA-A02K | 0.837484 | low |
| TCGA-AA-3844 | 0.548352 | low |
| TCGA-AA-A00J | 1.929348 | high |
| TCGA-AA-3861 | 0.467412 | low |
| TCGA-AA-3697 | 0.978572 | high |
| TCGA-CK-6748 | 0.670982 | low |
| TCGA-CK-5913 | 0.337402 | low |
| TCGA-AA-3980 | 3.073399 | high |
| TCGA-AA-A00Q | 0.52967 | low |
| TCGA-AD-5900 | 0.928619 | low |
| TCGA-D5-5539 | 1.405394 | high |
| TCGA-F4-6807 | 1.882548 | high |
| TCGA-A6-6137 | 2.673791 | high |
| TCGA-AA-3846 | 0.424521 | low |
| TCGA-CM-4752 | 0.707582 | low |
| TCGA-A6-2678 | 1.393369 | high |
| TCGA-AA-3663 | 0.203781 | low |
| TCGA-D5-6531 | 0.082271 | low |
| TCGA-AA-3947 | 1.156644 | high |
| TCGA-A6-6781 | 0.852213 | low |
| TCGA-D5-6923 | 0.755745 | low |
| TCGA-D5-6898 | 3.207353 | high |
| TCGA-F4-6463 | 1.054341 | high |
| TCGA-AA-A00O | 1.908327 | high |
| TCGA-DM-A0XF | 2.325355 | high |
| TCGA-AA-3506 | 0.48409 | low |
| TCGA-AA-3675 | 0.395128 | low |
| TCGA-AA-3534 | 0.902003 | low |
| TCGA-AD-6890 | 1.021466 | high |
| TCGA-AA-A01Z | 0.307853 | low |
| TCGA-D5-6924 | 1.028813 | high |
| TCGA-D5-5538 | 0.537175 | low |
| TCGA-5M-AAT6 | 11.75445 | high |
| TCGA-D5-6929 | 0.498281 | low |
| TCGA-CM-6676 | 3.228356 | high |
| TCGA-DM-A28A | 1.866002 | high |
| TCGA-AA-3972 | 0.625087 | low |
| TCGA-F4-6461 | 0.9908 | high |
| TCGA-A6-2684 | 1.731509 | high |
| TCGA-CA-6715 | 0.046976 | low |
| TCGA-A6-6141 | 3.533465 | high |
| TCGA-F4-6855 | 1.044066 | high |
| TCGA-DM-A282 | 0.689608 | low |
| TCGA-A6-A5ZU | 2.335683 | high |
| TCGA-AD-6963 | 1.425885 | high |
| TCGA-AA-A01R | 0.650299 | low |
| TCGA-AA-3696 | 4.370147 | high |
| TCGA-CM-6162 | 0.855219 | low |
| TCGA-NH-A50T | 0.487937 | low |
| TCGA-A6-4107 | 0.681473 | low |
| TCGA-A6-2671 | 1.994162 | high |
| TCGA-NH-A6GA | 1.24981 | high |
| TCGA-AA-A03J | 1.937943 | high |
| TCGA-NH-A8F7 | 1.132078 | high |
| TCGA-CM-4751 | 0.79122 | low |
| TCGA-CM-6168 | 0.70938 | low |
| TCGA-AD-6899 | 0.540356 | low |
| TCGA-CM-5862 | 1.267196 | high |
| TCGA-RU-A8FL | 2.878388 | high |
| TCGA-AA-A02Y | 0.512063 | low |
| TCGA-AA-3538 | 1.833125 | high |
| TCGA-AA-3986 | 2.941814 | high |
| TCGA-CM-5341 | 2.203956 | high |
| TCGA-AA-3950 | 0.95516 | low |
| TCGA-A6-3810 | 0.944456 | low |
| TCGA-CK-6747 | 2.220216 | high |
| TCGA-CM-5861 | 0.752377 | low |
| TCGA-AA-3685 | 0.624996 | low |
| TCGA-DM-A0X9 | 0.756442 | low |
| TCGA-AY-6386 | 0.899311 | low |
| TCGA-G4-6314 | 1.037652 | high |
| TCGA-G4-6309 | 0.24627 | low |
| TCGA-CK-6751 | 0.743967 | low |
| TCGA-3L-AA1B | 1.777915 | high |
| TCGA-AA-3511 | 2.335458 | high |
| TCGA-CA-5796 | 2.025504 | high |
| TCGA-D5-6927 | 1.274059 | high |
| TCGA-AA-A02E | 2.249112 | high |
| TCGA-AA-3510 | 0.652367 | low |
| TCGA-AA-3692 | 1.863957 | high |
| TCGA-CM-5864 | 0.82582 | low |
| TCGA-A6-6140 | 0.770374 | low |
| TCGA-AA-3869 | 0.346287 | low |
| TCGA-G4-6588 | 0.349558 | low |
| TCGA-AA-3837 | 1.137417 | high |
| TCGA-AA-3970 | 0.798808 | low |
| TCGA-AA-3660 | 0.231225 | low |
| TCGA-CM-4746 | 0.131543 | low |
| TCGA-AA-3831 | 0.591989 | low |
| TCGA-A6-2686 | 1.382744 | high |
| TCGA-CK-5912 | 0.214179 | low |
| TCGA-5M-AAT4 | 1.939958 | high |
| TCGA-CM-4743 | 1.082893 | high |
| TCGA-AA-3862 | 1.710259 | high |
| TCGA-AA-3496 | 1.913596 | high |
| TCGA-G4-6625 | 1.740702 | high |
| TCGA-CM-4747 | 0.834582 | low |
| TCGA-AA-3979 | 3.835183 | high |
| TCGA-D5-6931 | 1.361229 | high |
| TCGA-AZ-6603 | 1.390751 | high |
| TCGA-AA-3517 | 2.870109 | high |
| TCGA-DM-A0XD | 0.500609 | low |
| TCGA-AA-3975 | 1.392521 | high |
| TCGA-AA-3681 | 0.626918 | low |
| TCGA-AA-A01G | 1.592998 | high |
| TCGA-SS-A7HO | 0.943422 | low |
| TCGA-F4-6569 | 5.486487 | high |
| TCGA-G4-6304 | 2.094391 | high |
| TCGA-DM-A1D8 | 5.360027 | high |
| TCGA-CA-6716 | 1.976257 | high |
| TCGA-AA-A02J | 2.043785 | high |
| TCGA-AA-3941 | 0.567327 | low |
| TCGA-AD-6965 | 3.086762 | high |
| TCGA-AA-3524 | 0.8079 | low |
| TCGA-A6-2680 | 0.333049 | low |
| TCGA-CM-6674 | 2.170266 | high |
| TCGA-A6-5661 | 0.996856 | high |
| TCGA-D5-5541 | 1.707514 | high |
| TCGA-F4-6703 | 2.062561 | high |
| TCGA-AD-6888 | 0.655081 | low |
| TCGA-DM-A1D9 | 1.323401 | high |
| TCGA-A6-2682 | 3.888082 | high |
| TCGA-D5-6539 | 0.261999 | low |
| TCGA-D5-6534 | 1.706195 | high |
| TCGA-DM-A1DB | 0.414787 | low |
| TCGA-AZ-4323 | 8.180161 | high |
| TCGA-G4-6297 | 1.415965 | high |
| TCGA-AA-3994 | 2.482601 | high |
| TCGA-D5-6541 | 2.648577 | high |
| TCGA-A6-6649 | 0.801701 | low |
| TCGA-AD-6964 | 6.418004 | high |
| TCGA-D5-6532 | 1.698255 | high |
| TCGA-CM-5868 | 0.830853 | low |
| TCGA-A6-A566 | 1.211021 | high |
| TCGA-AA-3715 | 1.293137 | high |
| TCGA-AZ-4308 | 0.277719 | low |
| TCGA-AA-3815 | 0.397368 | low |
| TCGA-DM-A1D7 | 1.287052 | high |
| TCGA-QL-A97D | 1.09471 | high |
| TCGA-CM-4744 | 0.490171 | low |
| TCGA-CK-4952 | 0.057279 | low |
| TCGA-CK-4948 | 0.884909 | low |
| TCGA-CA-5255 | 0.165249 | low |
| TCGA-AA-3678 | 1.550025 | high |
| TCGA-AA-3848 | 1.102972 | high |
| TCGA-A6-2685 | 0.98428 | high |
| TCGA-CM-6169 | 1.007248 | high |
| TCGA-AA-3502 | 1.146032 | high |
| TCGA-AZ-4616 | 1.067914 | high |
| TCGA-F4-6805 | 2.686956 | high |
| TCGA-CM-5348 | 1.557277 | high |
| TCGA-G4-6322 | 0.149333 | low |
| TCGA-D5-5537 | 1.003312 | high |
| TCGA-QG-A5YX | 2.429856 | high |
| TCGA-AA-3955 | 0.188818 | low |
| TCGA-D5-7000 | 1.411668 | high |
| TCGA-AA-3562 | 0.44397 | low |
| TCGA-AZ-5407 | 1.762785 | high |
| TCGA-AZ-4615 | 0.302667 | low |
| TCGA-DM-A1D6 | 2.815336 | high |
| TCGA-AA-3516 | 0.541134 | low |
| TCGA-G4-6307 | 0.561293 | low |
| TCGA-AA-3851 | 3.499797 | high |
| TCGA-CM-6675 | 0.767594 | low |
| TCGA-AA-A029 | 0.458827 | low |
| TCGA-AA-3556 | 1.668967 | high |
| TCGA-D5-6540 | 1.091594 | high |
| TCGA-D5-6920 | 0.342157 | low |
| TCGA-CK-4947 | 0.354025 | low |
| TCGA-D5-6537 | 0.478706 | low |
| TCGA-AA-3968 | 1.643923 | high |
| TCGA-CM-6680 | 5.284898 | high |
| TCGA-AD-A5EK | 2.250313 | high |
| TCGA-QG-A5YW | 0.862111 | low |
| TCGA-AA-3819 | 0.579934 | low |
| TCGA-CM-6163 | 0.684334 | low |
| TCGA-A6-6782 | 0.640229 | low |
| TCGA-CA-5797 | 0.580233 | low |
| TCGA-AA-3971 | 0.914753 | low |
| TCGA-EI-6885 | 1.354394 | high |
| TCGA-AG-3883 | 0.35556 | low |
| TCGA-DY-A1DE | 1.776221 | high |
| TCGA-AG-A026 | 1.228669 | high |
| TCGA-EF-5831 | 0.353473 | low |
| TCGA-AG-3726 | 0.209843 | low |
| TCGA-AG-A008 | 0.97407 | low |
| TCGA-F5-6571 | 3.239034 | high |
| TCGA-AG-A02X | 0.685248 | low |
| TCGA-DC-6158 | 2.031301 | high |
| TCGA-AG-A02N | 0.852085 | low |
| TCGA-EI-6511 | 5.157844 | high |
| TCGA-CI-6622 | 1.852704 | high |
| TCGA-AG-A015 | 1.614419 | high |
| TCGA-AH-6549 | 2.008611 | high |
| TCGA-EI-6882 | 0.502321 | low |
| TCGA-AG-3890 | 0.430405 | low |
| TCGA-F5-6864 | 2.687332 | high |
| TCGA-DY-A1H8 | 1.770593 | high |
| TCGA-AG-A016 | 0.746648 | low |
| TCGA-EI-6513 | 0.38557 | low |
| TCGA-AG-A020 | 0.428807 | low |
| TCGA-AG-3587 | 1.258335 | high |
| TCGA-AG-3608 | 0.653874 | low |
| TCGA-EI-7002 | 1.166298 | high |
| TCGA-AG-A025 | 0.819659 | low |
| TCGA-EI-6506 | 1.5767 | high |
| TCGA-AG-A036 | 1.352796 | high |
| TCGA-G5-6233 | 0.363219 | low |
| TCGA-DC-5869 | 2.13348 | high |
| TCGA-AG-3896 | 0.257605 | low |
| TCGA-AG-4021 | 1.647959 | high |
| TCGA-EI-6510 | 1.435416 | high |
| TCGA-AG-A002 | 0.684835 | low |
| TCGA-AF-3911 | 1.205408 | high |
| TCGA-EI-6883 | 0.702087 | low |
| TCGA-CI-6624 | 1.694066 | high |
| TCGA-AG-4005 | 0.186848 | low |
| TCGA-AG-3584 | 1.293451 | high |
| TCGA-AF-2690 | 2.701791 | high |
| TCGA-CL-4957 | 3.097909 | high |
| TCGA-CI-6621 | 2.192678 | high |
| TCGA-EI-6514 | 0.807614 | low |
| TCGA-DT-5265 | 0.171415 | low |
| TCGA-AG-A01N | 0.629225 | low |
