# Supplementary material for: Cuproptosis‐related miRNAs signature and immune infiltration characteristics in colorectal cancer
Source: Cancer Med. 2023 Jun 19;12(15):16661–78. doi: 10.1002/cam4.6270 (PMC10469834; doi:10.1002/cam4.6270)
Supplement: Supplementary file 6 — Table S5 [file CAM4-12-16661-s001.docx]

TABLE S5 The ESTIMATE score of all the samples.

| Sample | StromalScore | ImmuneScore | ESTIMATEScore |
| --- | --- | --- | --- |
| TCGA-AA-3679-01A | 8910.81 | 11136.19 | 2.00E+04 |
| TCGA-AD-6901-01A | 11485.14 | 12402.42 | 2.39E+04 |
| TCGA-CM-5863-01A | 10581.94 | 11519.68 | 2.21E+04 |
| TCGA-AA-3672-01A | 9323.789 | 13206.45 | 2.25E+04 |
| TCGA-A6-6142-01A | 11431.16 | 11548.84 | 2.30E+04 |
| TCGA-AZ-6598-01A | 8653.553 | 12049.47 | 2.07E+04 |
| TCGA-AZ-5403-01A | 10414.4 | 11007.23 | 2.14E+04 |
| TCGA-F4-6570-01A | 11427.71 | 13822.51 | 2.53E+04 |
| TCGA-AA-3662-01A | 9618.074 | 12253.33 | 2.19E+04 |
| TCGA-A6-5656-01B | 10767.82 | 9701.659 | 2.05E+04 |
| TCGA-AA-3667-01A | 8341.212 | 11420.86 | 1.98E+04 |
| TCGA-CM-6167-01A | 12801.69 | 12707.11 | 2.55E+04 |
| TCGA-CA-5256-01A | 8421.576 | 11179.22 | 1.96E+04 |
| TCGA-AA-A02H-01A | 8143.588 | 9949.014 | 1.81E+04 |
| TCGA-AA-3989-01A | 10034.44 | 12406.01 | 2.24E+04 |
| TCGA-D5-6530-01A | 9109.885 | 12815.02 | 2.19E+04 |
| TCGA-G4-6299-01A | 10256.77 | 12526.9 | 2.28E+04 |
| TCGA-AU-3779-01A | 10043.36 | 12545.89 | 2.26E+04 |
| TCGA-AA-A00W-01A | 5441.182 | 10031.37 | 1.55E+04 |
| TCGA-AZ-6600-01A | 10675.82 | 12065.68 | 2.27E+04 |
| TCGA-CM-6679-01A | 10811.26 | 11787.06 | 2.26E+04 |
| TCGA-AA-3877-01A | 10485.49 | 12963.47 | 2.34E+04 |
| TCGA-D5-6529-01A | 11334.5 | 13444.84 | 2.48E+04 |
| TCGA-G4-6302-01A | 12466.76 | 12475.04 | 2.49E+04 |
| TCGA-AY-A71X-01A | 4335.82 | 8150.339 | 1.25E+04 |
| TCGA-A6-6650-01A | 6818.42 | 9264.518 | 1.61E+04 |
| TCGA-NH-A5IV-01A | 9393.952 | 12458.3 | 2.19E+04 |
| TCGA-AA-3949-01A | 11075.73 | 14373.88 | 2.54E+04 |
| TCGA-D5-6922-01A | 10791.89 | 11208 | 2.20E+04 |
| TCGA-AA-3495-01A | 8194.024 | 11463.96 | 1.97E+04 |
| TCGA-AA-3519-01A | 7683.19 | 10601.42 | 1.83E+04 |
| TCGA-DM-A28C-01A | 6663.736 | 8609.918 | 1.53E+04 |
| TCGA-G4-6293-01A | 8836.912 | 12497.95 | 2.13E+04 |
| TCGA-A6-5665-01A | 7320.982 | 11232.62 | 1.86E+04 |
| TCGA-CK-5915-01A | 7326.039 | 8771.772 | 1.61E+04 |
| TCGA-A6-5662-01A | 8861.713 | 9669.951 | 1.85E+04 |
| TCGA-AA-A02K-01A | 6692.824 | 9433.514 | 1.61E+04 |
| TCGA-AA-3844-01A | 7995.175 | 11274.25 | 1.93E+04 |
| TCGA-AA-A00J-01A | 9265.699 | 11021.2 | 2.03E+04 |
| TCGA-CA-6718-01A | 10188.77 | 13548.56 | 2.37E+04 |
| TCGA-AA-3861-01A | 7065.195 | 11955.21 | 1.90E+04 |
| TCGA-AA-3697-01A | 8618.792 | 11389.28 | 2.00E+04 |
| TCGA-A6-5659-01A | 8916.267 | 9729.375 | 1.86E+04 |
| TCGA-CK-6748-01A | 11341.4 | 11695.67 | 2.30E+04 |
| TCGA-NH-A50U-01A | 8317.849 | 9659.359 | 1.80E+04 |
| TCGA-CK-5913-01A | 9613.197 | 12039.28 | 21652.48 |
| TCGA-AA-A01F-01A | 5740.864 | 9413.093 | 15153.96 |
| TCGA-AY-4070-01A | 8585.472 | 11174.4 | 19759.87 |
| TCGA-AZ-6608-01A | 4863.473 | 8708.154 | 13571.63 |
| TCGA-CK-5916-01A | 10612.63 | 13429.45 | 24042.08 |
| TCGA-AA-3980-01A | 9385.095 | 12891.36 | 22276.45 |
| TCGA-AA-A00Q-01A | 8340.32 | 10650.72 | 18991.04 |
| TCGA-AD-5900-01A | 10257.15 | 12543.51 | 22800.66 |
| TCGA-AA-3664-01A | 7388.119 | 10736.86 | 18124.98 |
| TCGA-D5-5539-01A | 10547.16 | 11881.49 | 22428.65 |
| TCGA-AA-3554-01A | 10907.88 | 13524.58 | 24432.46 |
| TCGA-F4-6807-01A | 12309.04 | 13289.57 | 25598.61 |
| TCGA-AY-A8YK-01A | 7573.058 | 10091.51 | 17664.57 |
| TCGA-A6-6137-01A | 8554.451 | 11970 | 20524.45 |
| TCGA-AA-3494-01A | 8091.714 | 10858.98 | 18950.7 |
| TCGA-AA-3712-01A | 9779.529 | 10952.68 | 20732.21 |
| TCGA-AA-3864-01A | 9483.779 | 11123.67 | 20607.45 |
| TCGA-AA-3846-01A | 8697.345 | 11726.31 | 20423.65 |
| TCGA-CM-4752-01A | 9660.939 | 12222.96 | 21883.9 |
| TCGA-AA-3555-01A | 9726.084 | 11318.76 | 21044.84 |
| TCGA-A6-2678-01A | 9505.702 | 11700.99 | 21206.69 |
| TCGA-AA-3713-01A | 9084.611 | 12642.99 | 21727.6 |
| TCGA-AA-3663-01A | 8470.148 | 11385.03 | 19855.17 |
| TCGA-AZ-6599-01A | 5219.979 | 9034.634 | 14254.61 |
| TCGA-D5-6531-01A | 10282.97 | 12062.55 | 22345.52 |
| TCGA-A6-5664-01A | 11550.96 | 12872.08 | 24423.04 |
| TCGA-AA-3947-01A | 9793.741 | 11627.52 | 21421.26 |
| TCGA-A6-6781-01A | 12754.51 | 13696.02 | 26450.53 |
| TCGA-AA-3531-01A | 7373.7 | 9858.162 | 17231.86 |
| TCGA-AA-3529-01A | 8111.79 | 10434.38 | 18546.17 |
| TCGA-F4-6704-01A | 12206.07 | 12147.52 | 24353.58 |
| TCGA-D5-6923-01A | 10630.02 | 11045.15 | 21675.16 |
| TCGA-AD-A5EJ-01A | 8229.959 | 10554.72 | 18784.68 |
| TCGA-D5-6898-01A | 10617.58 | 11935.48 | 22553.06 |
| TCGA-5M-AATA-01A | 8844.557 | 11055.25 | 19899.8 |
| TCGA-F4-6463-01A | 10568.07 | 11124.15 | 21692.22 |
| TCGA-AZ-6605-01A | 12129.87 | 13061.95 | 25191.82 |
| TCGA-AA-A00L-01A | 7043.862 | 9379.079 | 16422.94 |
| TCGA-NH-A8F8-01A | 9735.413 | 10883.77 | 20619.18 |
| TCGA-5M-AATE-01A | 7651.159 | 9804.705 | 17455.86 |
| TCGA-AA-A00O-01A | 10195.89 | 11027.84 | 21223.73 |
| TCGA-AA-3977-01A | 9646.603 | 12224.3 | 21870.9 |
| TCGA-DM-A0XF-01A | 8640.283 | 10598.23 | 19238.52 |
| TCGA-AA-3506-01A | 9395.544 | 12139.12 | 21534.66 |
| TCGA-DM-A28M-01A | 5641.454 | 9119.809 | 14761.26 |
| TCGA-AA-3675-01A | 8710.648 | 10821.12 | 19531.76 |
| TCGA-AA-3534-01A | 8918.552 | 10450.43 | 19368.98 |
| TCGA-A6-5665-01B | 8308.823 | 10350.76 | 18659.58 |
| TCGA-AD-6890-01A | 8787.291 | 10730.66 | 19517.95 |
| TCGA-A6-2683-01A | 6747.697 | 9341.358 | 16089.06 |
| TCGA-AA-A01Z-01A | 6783.234 | 8877.191 | 15660.43 |
| TCGA-DM-A1D0-01A | 5557.65 | 7664.867 | 13222.52 |
| TCGA-D5-6924-01A | 11407.91 | 12662.06 | 24069.97 |
| TCGA-DM-A1D4-01A | 5620.207 | 9811.192 | 15431.4 |
| TCGA-DM-A28K-01A | 6593.185 | 9494.475 | 16087.66 |
| TCGA-AA-3522-01A | 7961.237 | 11357.09 | 19318.32 |
| TCGA-5M-AAT5-01A | 6458.449 | 9051.28 | 15509.73 |
| TCGA-DM-A28F-01A | 6830.447 | 9700.593 | 16531.04 |
| TCGA-AA-A022-01A | 9081.261 | 12727.73 | 21808.99 |
| TCGA-D5-5538-01A | 11094.03 | 12959.52 | 24053.55 |
| TCGA-5M-AAT6-01A | 11185.66 | 13390.89 | 24576.55 |
| TCGA-DM-A285-01A | 8611.786 | 8562.889 | 17174.67 |
| TCGA-A6-2674-01A | 11910.59 | 13765.69 | 25676.28 |
| TCGA-D5-6929-01A | 10347.08 | 11587.8 | 21934.88 |
| TCGA-A6-4105-01A | 10490.06 | 12869.22 | 23359.28 |
| TCGA-CM-6676-01A | 9259.238 | 9754.983 | 19014.22 |
| TCGA-AA-3930-01A | 9330.019 | 12239.79 | 21569.81 |
| TCGA-AA-3842-01A | 10281.62 | 11396.76 | 21678.38 |
| TCGA-DM-A28A-01A | 8737.459 | 9675.723 | 18413.18 |
| TCGA-AA-3972-01A | 8720.162 | 10320.53 | 19040.69 |
| TCGA-CM-6161-01A | 9658.702 | 12002.73 | 21661.43 |
| TCGA-F4-6461-01A | 10498.22 | 11842.76 | 22340.98 |
| TCGA-F4-6809-01A | 10847.44 | 12346.86 | 23194.3 |
| TCGA-G4-6626-01A | 6490.624 | 9245.203 | 15735.83 |
| TCGA-A6-2684-01A | 10859.81 | 12315.46 | 23175.28 |
| TCGA-CA-6717-01A | 11927.99 | 13345.33 | 25273.32 |
| TCGA-DM-A28G-01A | 7326.028 | 9826.094 | 17152.12 |
| TCGA-A6-3809-01A | 9658.437 | 13258.84 | 22917.28 |
| TCGA-A6-5667-01A | 9605.174 | 10392.93 | 19998.11 |
| TCGA-CA-5254-01A | 9319.426 | 10288.72 | 19608.15 |
| TCGA-A6-6654-01A | 12814.73 | 14311.24 | 27125.97 |
| TCGA-CA-6715-01A | 7141.616 | 8833.122 | 15974.74 |
| TCGA-A6-6141-01A | 9033.331 | 12189.02 | 21222.35 |
| TCGA-G4-6317-02A | 7114.937 | 8421.295 | 15536.23 |
| TCGA-G4-6317-01A | 5572.2 | 7824.68 | 13396.88 |
| TCGA-G4-6295-01A | 8830.606 | 12503.66 | 21334.27 |
| TCGA-F4-6855-01A | 11884.39 | 11374.41 | 23258.79 |
| TCGA-AM-5821-01A | 9556.58 | 12884.91 | 22441.49 |
| TCGA-DM-A282-01A | 7905.823 | 8880.779 | 16786.6 |
| TCGA-AM-5820-01A | 9347.855 | 10188.16 | 19536.02 |
| TCGA-A6-A5ZU-01A | 10505.32 | 12117.5 | 22622.81 |
| TCGA-AD-6963-01A | 8028.647 | 11963.65 | 19992.3 |
| TCGA-F4-6854-01A | 10011.53 | 11284.39 | 21295.92 |
| TCGA-A6-6652-01A | 6994.578 | 9117.529 | 16112.11 |
| TCGA-AA-A01R-01A | 8151.198 | 13068.24 | 21219.44 |
| TCGA-AA-3866-01A | 11267.74 | 13635.79 | 24903.53 |
| TCGA-AA-3696-01A | 8611.638 | 9589.732 | 18201.37 |
| TCGA-F4-6459-01A | 11335.08 | 11229.77 | 22564.85 |
| TCGA-CM-6162-01A | 13237.95 | 14007.96 | 27245.91 |
| TCGA-AA-3542-01A | 6833.069 | 9660.508 | 16493.58 |
| TCGA-NH-A50T-01A | 6619.763 | 8527.899 | 15147.66 |
| TCGA-A6-4107-01A | 9745.248 | 11725.3 | 21470.55 |
| TCGA-DM-A1HB-01A | 7482.14 | 9650.208 | 17132.35 |
| TCGA-A6-2671-01A | 10401.57 | 11358.79 | 21760.36 |
| TCGA-A6-5666-01A | 7182.726 | 9502.775 | 16685.5 |
| TCGA-AA-3956-01A | 9504.287 | 11426.95 | 20931.24 |
| TCGA-NH-A6GA-01A | 8014.79 | 10339.87 | 18354.66 |
| TCGA-A6-2672-01B | 8201.965 | 11862.57 | 20064.53 |
| TCGA-AA-A03J-01A | 8693.388 | 11720.22 | 20413.6 |
| TCGA-NH-A8F7-06A | 5187.88 | 7227.65 | 12415.53 |
| TCGA-F4-6856-01A | 8677.04 | 11027.78 | 19704.82 |
| TCGA-CM-4748-01A | 7958.989 | 10215.1 | 18174.09 |
| TCGA-A6-2681-01A | 10781.54 | 11989.07 | 22770.61 |
| TCGA-CM-4751-01A | 9766.148 | 12410.19 | 22176.34 |
| TCGA-AY-6196-01A | 13925.14 | 15550.52 | 29475.66 |
| TCGA-AY-A69D-01A | 7813.618 | 10159.15 | 17972.77 |
| TCGA-AA-3984-01A | 9760.815 | 11901.41 | 21662.22 |
| TCGA-CM-6168-01A | 12289.55 | 13024.16 | 25313.72 |
| TCGA-AD-6899-01A | 11391.94 | 12929.59 | 24321.54 |
| TCGA-CM-5862-01A | 8832.007 | 9141.532 | 17973.54 |
| TCGA-AA-3544-01A | 10625.73 | 13388.44 | 24014.17 |
| TCGA-AZ-4315-01A | 8469.265 | 11188.2 | 19657.46 |
| TCGA-RU-A8FL-01A | 4122.137 | 8076.965 | 12199.1 |
| TCGA-AA-3489-01A | 13004.55 | 14328.25 | 27332.8 |
| TCGA-AA-3710-01A | 10150.84 | 14384.54 | 24535.39 |
| TCGA-D5-6926-01A | 11082.05 | 11875.78 | 22957.82 |
| TCGA-AY-4071-01A | 9735.392 | 13010.45 | 22745.84 |
| TCGA-AA-3553-01A | 9926.756 | 12392.62 | 22319.38 |
| TCGA-AA-A02Y-01A | 5319.202 | 10959.01 | 16278.21 |
| TCGA-AA-3538-01A | 9606.533 | 10861.94 | 20468.47 |
| TCGA-AA-A01V-01A | 5651.513 | 10810.77 | 16462.28 |
| TCGA-AA-3986-01A | 9275.778 | 13498.43 | 22774.2 |
| TCGA-CM-5341-01A | 11372.5 | 13110.63 | 24483.13 |
| TCGA-AA-3520-01A | 9974.856 | 11577.5 | 21552.36 |
| TCGA-DM-A28H-01A | 6882.317 | 8775.643 | 15657.96 |
| TCGA-A6-5657-01A | 10572.93 | 12067.22 | 22640.15 |
| TCGA-D5-6930-01A | 10655.58 | 12696.2 | 23351.78 |
| TCGA-AA-3860-01A | 10129.19 | 12392.15 | 22521.34 |
| TCGA-AA-3950-01A | 11248.55 | 13941 | 25189.55 |
| TCGA-A6-3810-01A | 10824.3 | 12059.06 | 22883.36 |
| TCGA-AA-3526-01A | 9379.107 | 12156.09 | 21535.19 |
| TCGA-AA-A01S-01A | 5313.898 | 8180.376 | 13494.27 |
| TCGA-CK-6747-01A | 9123.274 | 11140.25 | 20263.52 |
| TCGA-CM-5860-01A | 11382.28 | 11839.31 | 23221.6 |
| TCGA-AA-3530-01A | 7652.361 | 11372.22 | 19024.58 |
| TCGA-D5-6536-01A | 9326.703 | 11207.28 | 20533.98 |
| TCGA-D5-6538-01A | 6919.726 | 8030.103 | 14949.83 |
| TCGA-A6-5660-01A | 9633.85 | 10852.47 | 20486.32 |
| TCGA-A6-2677-01A | 7142.033 | 9365.747 | 16507.78 |
| TCGA-CM-5861-01A | 7822.808 | 10155.22 | 17978.03 |
| TCGA-AZ-4313-01A | 7455.226 | 9873.07 | 17328.3 |
| TCGA-AA-3685-01A | 9740.894 | 12796.4 | 22537.29 |
| TCGA-AA-3854-01A | 8124.862 | 10709.13 | 18833.99 |
| TCGA-AA-3655-01A | 9443.348 | 11144.52 | 20587.87 |
| TCGA-DM-A0X9-01A | 7909.132 | 10842.7 | 18751.84 |
| TCGA-A6-A567-01A | 8288.177 | 9417.725 | 17705.9 |
| TCGA-AY-6386-01A | 8236.677 | 11568.26 | 19804.94 |
| TCGA-G4-6314-01A | 11021.01 | 11141.5 | 22162.51 |
| TCGA-G4-6309-01A | 7517.008 | 10544.75 | 18061.76 |
| TCGA-CK-6751-01A | 9978.042 | 11164.82 | 21142.86 |
| TCGA-CM-6171-01A | 8563.588 | 11551.67 | 20115.26 |
| TCGA-G4-6294-01A | 7842.501 | 10365.18 | 18207.68 |
| TCGA-3L-AA1B-01A | 9020.137 | 10948.27 | 19968.41 |
| TCGA-AA-3511-01A | 10287.32 | 10916.62 | 21203.94 |
| TCGA-CA-5796-01A | 7799.802 | 11414.11 | 19213.91 |
| TCGA-D5-6927-01A | 10562.21 | 13057.75 | 23619.97 |
| TCGA-AA-A02E-01A | 7390.636 | 10251.36 | 17642 |
| TCGA-AA-3952-01A | 9533.139 | 10836.76 | 20369.9 |
| TCGA-DM-A288-01A | 6219.604 | 8490.718 | 14710.32 |
| TCGA-AA-3510-01A | 8675.723 | 12143.19 | 20818.92 |
| TCGA-AA-3692-01A | 9270.039 | 12127.95 | 21397.99 |
| TCGA-CM-5864-01A | 7633.112 | 10478.62 | 18111.73 |
| TCGA-A6-6140-01A | 7356.369 | 10778.85 | 18135.22 |
| TCGA-AA-3869-01A | 9956.416 | 12680.13 | 22636.55 |
| TCGA-AA-3527-01A | 9742.609 | 11778.05 | 21520.66 |
| TCGA-NH-A8F7-01A | 6471.316 | 8306.847 | 14778.16 |
| TCGA-G4-6588-01A | 8894.497 | 11429.52 | 20324.02 |
| TCGA-AY-5543-01A | 7546.719 | 11329.41 | 18876.13 |
| TCGA-AA-3837-01A | 9266.062 | 10766.32 | 20032.38 |
| TCGA-AA-3492-01A | 9179.065 | 11667.8 | 20846.86 |
| TCGA-AA-A00D-01A | 10054.03 | 13632.33 | 23686.36 |
| TCGA-F4-6806-01A | 9100.445 | 11136.12 | 20236.56 |
| TCGA-AA-3970-01A | 8525.552 | 12028.47 | 20554.02 |
| TCGA-AA-3660-01A | 8952.809 | 10832.55 | 19785.36 |
| TCGA-CM-4746-01A | 6422.607 | 9871.757 | 16294.36 |
| TCGA-AY-A54L-01A | 4599.207 | 8294.529 | 12893.74 |
| TCGA-CM-6165-01A | 10923.7 | 11807.05 | 22730.75 |
| TCGA-AA-A01P-01A | 10903.07 | 13974.02 | 24877.09 |
| TCGA-A6-2675-01A | 11554.98 | 12555.16 | 24110.15 |
| TCGA-CM-5344-01A | 10046.08 | 10235.08 | 20281.16 |
| TCGA-AA-3973-01A | 8482.349 | 9914.4 | 18396.75 |
| TCGA-AA-3831-01A | 9000.659 | 11859.8 | 20860.46 |
| TCGA-A6-2686-01A | 10773.98 | 14041.27 | 24815.25 |
| TCGA-CK-5912-01A | 8848.134 | 10214.11 | 19062.24 |
| TCGA-AA-A01X-01A | 7541.906 | 9841.083 | 17382.99 |
| TCGA-CM-5349-01A | 10368.79 | 11931.25 | 22300.04 |
| TCGA-5M-AAT4-01A | 6919.703 | 8900.675 | 15820.38 |
| TCGA-A6-6648-01A | 7530.31 | 10120.56 | 17650.87 |
| TCGA-CM-4743-01A | 8273.452 | 11721.25 | 19994.7 |
| TCGA-AA-3867-01A | 10788.36 | 11356.01 | 22144.36 |
| TCGA-AA-3862-01A | 9140.053 | 12419.86 | 21559.92 |
| TCGA-F4-6808-01A | 7123.253 | 9403.229 | 16526.48 |
| TCGA-CM-6677-01A | 10020.16 | 11462.53 | 21482.69 |
| TCGA-AA-3561-01A | 7900.154 | 10491.6 | 18391.75 |
| TCGA-G4-6628-01A | 10369.75 | 13997.64 | 24367.39 |
| TCGA-A6-6650-01B | 6421.099 | 8400.325 | 14821.43 |
| TCGA-AU-6004-01A | 10260.12 | 12843.39 | 23103.51 |
| TCGA-D5-6533-01A | 9118.37 | 11023.84 | 20142.21 |
| TCGA-AA-3496-01A | 10974.27 | 12904.62 | 23878.89 |
| TCGA-G4-6625-01A | 10729.81 | 13730.27 | 24460.08 |
| TCGA-CM-6172-01A | 9453.872 | 10709.4 | 20163.27 |
| TCGA-AA-3666-01A | 8898.036 | 11935.71 | 20833.75 |
| TCGA-CM-6678-01A | 8219.808 | 9512.897 | 17732.71 |
| TCGA-AA-3852-01A | 10020.3 | 12026.98 | 22047.28 |
| TCGA-CM-4747-01A | 8923.995 | 10533.41 | 19457.41 |
| TCGA-AA-3979-01A | 7160.848 | 9696.425 | 16857.27 |
| TCGA-D5-6928-01A | 12533.67 | 16117.53 | 28651.19 |
| TCGA-D5-6931-01A | 9885.974 | 12203.84 | 22089.81 |
| TCGA-AA-A02O-01A | 7212.974 | 10479.42 | 17692.39 |
| TCGA-AA-3939-01A | 9162.349 | 11828.08 | 20990.43 |
| TCGA-AZ-6603-01A | 10313.08 | 11833.12 | 22146.2 |
| TCGA-AA-3517-01A | 8647.594 | 11193.03 | 19840.62 |
| TCGA-CM-6164-01A | 8952.258 | 11050.56 | 20002.82 |
| TCGA-CK-5914-01A | 8848.487 | 10566.33 | 19414.81 |
| TCGA-CK-6746-01A | 8103.395 | 12895.18 | 20998.58 |
| TCGA-AA-A03F-01A | 6735.706 | 10856.46 | 17592.16 |
| TCGA-AA-3693-01A | 8087.491 | 10345.46 | 18432.95 |
| TCGA-AA-A024-01A | 7211.325 | 9999.687 | 17211.01 |
| TCGA-DM-A0XD-01A | 8023.996 | 9649.084 | 17673.08 |
| TCGA-NH-A50V-01A | 10026.26 | 11668.37 | 21694.63 |
| TCGA-AA-A02F-01A | 7817.023 | 8640.72 | 16457.74 |
| TCGA-AA-3975-01A | 9173.686 | 11474.07 | 20647.76 |
| TCGA-AA-3681-01A | 8825.944 | 12360.23 | 21186.17 |
| TCGA-AA-A01G-01A | 6983.797 | 9601.531 | 16585.33 |
| TCGA-SS-A7HO-01A | 6370.72 | 8690.861 | 15061.58 |
| TCGA-F4-6569-01A | 12853.52 | 12821.72 | 25675.25 |
| TCGA-AA-3855-01A | 8365.546 | 11764.93 | 20130.48 |
| TCGA-G4-6304-01A | 6115.673 | 10507.8 | 16623.47 |
| TCGA-DM-A28E-01A | 5536.21 | 8698.028 | 14234.24 |
| TCGA-DM-A280-01A | 8327.463 | 10233.06 | 18560.52 |
| TCGA-D5-6535-01A | 9347.46 | 11968.7 | 21316.16 |
| TCGA-DM-A1D8-01A | 6763.816 | 8903.119 | 15666.94 |
| TCGA-D5-6932-01A | 9767.332 | 11303.13 | 21070.47 |
| TCGA-CA-6716-01A | 8904.314 | 9173.794 | 18078.11 |
| TCGA-AA-A02J-01A | 5984.384 | 8319.098 | 14303.48 |
| TCGA-DM-A1DA-01A | 6768.315 | 9515.766 | 16284.08 |
| TCGA-AA-3941-01A | 7559.873 | 10162.78 | 17722.66 |
| TCGA-AD-6965-01A | 8452.157 | 10139.48 | 18591.63 |
| TCGA-AA-3524-01A | 8826.002 | 11075.99 | 19901.99 |
| TCGA-AA-3532-01A | 10527.34 | 13151.41 | 23678.76 |
| TCGA-AA-A01T-01A | 6174.074 | 9344.588 | 15518.66 |
| TCGA-A6-2680-01A | 9079.283 | 11338.06 | 20417.35 |
| TCGA-G4-6321-01A | 7462.391 | 12156.93 | 19619.32 |
| TCGA-AA-3845-01A | 9448.948 | 12521.16 | 21970.11 |
| TCGA-CM-6674-01A | 10153.21 | 12000.21 | 22153.43 |
| TCGA-G4-6310-01A | 8578.821 | 8230.876 | 16809.7 |
| TCGA-A6-A565-01A | 10600.89 | 13990.7 | 24591.59 |
| TCGA-AA-3875-01A | 9455.286 | 12363.81 | 21819.1 |
| TCGA-CK-4951-01A | 10802.89 | 13085.34 | 23888.23 |
| TCGA-A6-5661-01A | 8501.732 | 11103.53 | 19605.26 |
| TCGA-A6-3808-01A | 11577.94 | 12983.2 | 24561.14 |
| TCGA-D5-5541-01A | 10376.23 | 11979.42 | 22355.65 |
| TCGA-F4-6703-01A | 13934.03 | 15160.85 | 29094.88 |
| TCGA-AD-6888-01A | 5173.698 | 9155.629 | 14329.33 |
| TCGA-DM-A1D9-01A | 7370.219 | 9229.752 | 16599.97 |
| TCGA-CM-6170-01A | 10066.94 | 11589.12 | 21656.06 |
| TCGA-A6-2682-01A | 10054.38 | 11716.99 | 21771.38 |
| TCGA-D5-6539-01A | 8624.921 | 11605.85 | 20230.77 |
| TCGA-A6-6780-01A | 8646.35 | 13632.15 | 22278.5 |
| TCGA-D5-6534-01A | 13507.88 | 14848.36 | 28356.24 |
| TCGA-AA-3833-01A | 10316.88 | 12574.24 | 22891.12 |
| TCGA-F4-6460-01A | 10269.82 | 11311.5 | 21581.31 |
| TCGA-DM-A1DB-01A | 5674.561 | 10125.81 | 15800.37 |
| TCGA-A6-6653-01A | 9178.854 | 11574.83 | 20753.68 |
| TCGA-G4-6298-01A | 8320.194 | 8445.258 | 16765.45 |
| TCGA-A6-A56B-01A | 8998.212 | 9492.104 | 18490.32 |
| TCGA-A6-2672-01A | 10215.97 | 13938.52 | 24154.49 |
| TCGA-AZ-4323-01A | 10735.83 | 14306.37 | 25042.21 |
| TCGA-G4-6297-01A | 11199.76 | 12113.33 | 23313.09 |
| TCGA-AA-3811-01A | 9076.38 | 11758.13 | 20834.51 |
| TCGA-AA-3994-01A | 9221.171 | 11214.58 | 20435.75 |
| TCGA-D5-6541-01A | 11460.3 | 12865.05 | 24325.35 |
| TCGA-A6-2679-01A | 8413.22 | 11870.9 | 20284.12 |
| TCGA-A6-6649-01A | 10161.9 | 12042.4 | 22204.29 |
| TCGA-AD-6964-01A | 12118.5 | 14218.36 | 26336.86 |
| TCGA-D5-6532-01A | 7446.603 | 9472.326 | 16918.93 |
| TCGA-CM-5868-01A | 9300.562 | 10182.8 | 19483.36 |
| TCGA-A6-A566-01A | 13506.21 | 13960.35 | 27466.55 |
| TCGA-A6-5656-01A | 6486.728 | 8606.21 | 15092.94 |
| TCGA-A6-6781-01B | 13233.94 | 13459.41 | 26693.35 |
| TCGA-AA-3966-01A | 11069.54 | 14120.94 | 25190.48 |
| TCGA-CK-4950-01A | 9349.093 | 12540.32 | 21889.42 |
| TCGA-AA-3688-01A | 8615.153 | 11113.99 | 19729.15 |
| TCGA-AA-3715-01A | 11232.93 | 13699.77 | 24932.7 |
| TCGA-AZ-4308-01A | 9545.342 | 10966.49 | 20511.84 |
| TCGA-4T-AA8H-01A | 5331.335 | 8820.426 | 14151.76 |
| TCGA-AY-6197-01A | 7389.418 | 11173.28 | 18562.69 |
| TCGA-AA-3548-01A | 8995.008 | 11733.83 | 20728.83 |
| TCGA-AA-A010-01A | 8057.404 | 10945.36 | 19002.77 |
| TCGA-AA-3982-01A | 10029.93 | 12754.34 | 22784.27 |
| TCGA-G4-6315-01A | 6524.83 | 9300.34 | 15825.17 |
| TCGA-AA-3812-01A | 10192.17 | 11911.78 | 22103.95 |
| TCGA-AA-3509-01A | 9117.947 | 11515.9 | 20633.85 |
| TCGA-4N-A93T-01A | 5370.89 | 9066.964 | 14437.85 |
| TCGA-AA-3815-01A | 9357.065 | 13565 | 22922.07 |
| TCGA-G4-6311-01A | 10216.57 | 11691.29 | 21907.87 |
| TCGA-DM-A1D7-01A | 7863.933 | 10150.05 | 18013.98 |
| TCGA-G4-6303-01A | 9970.909 | 10854.64 | 20825.55 |
| TCGA-A6-6651-01A | 12985.83 | 13746.71 | 26732.54 |
| TCGA-QL-A97D-01A | 7613.298 | 11824.86 | 19438.16 |
| TCGA-G4-6306-01A | 5676.155 | 10252.59 | 15928.75 |
| TCGA-CM-4744-01A | 7494.62 | 11720.61 | 19215.23 |
| TCGA-CK-4952-01A | 8086.601 | 10783.82 | 18870.42 |
| TCGA-AA-3872-01A | 11650.46 | 12309.43 | 23959.89 |
| TCGA-CK-4948-01B | 10047.25 | 11351.32 | 21398.57 |
| TCGA-CA-5255-01A | 6283.224 | 9419.316 | 15702.54 |
| TCGA-AA-3549-01A | 8447.638 | 11309.55 | 19757.18 |
| TCGA-AA-3678-01A | 8449.326 | 11985.05 | 20434.38 |
| TCGA-QG-A5Z2-01A | 6177.936 | 12063.26 | 18241.19 |
| TCGA-AA-3848-01A | 7584.646 | 10513.1 | 18097.74 |
| TCGA-A6-2685-01A | 11751.17 | 13084.57 | 24835.73 |
| TCGA-AA-3856-01A | 8993.122 | 12811.29 | 21804.41 |
| TCGA-CM-6169-01A | 12416.86 | 13689.91 | 26106.77 |
| TCGA-NH-A6GC-01A | 9009.305 | 9738.676 | 18747.98 |
| TCGA-AA-3502-01A | 6316.997 | 11174.53 | 17491.53 |
| TCGA-AZ-4616-01A | 8693.94 | 11351.01 | 20044.95 |
| TCGA-AA-3543-01A | 9551.29 | 13320.79 | 22872.08 |
| TCGA-DM-A1HA-01A | 4527.592 | 8813.094 | 13340.69 |
| TCGA-F4-6805-01A | 11860.65 | 12685.74 | 24546.39 |
| TCGA-CM-5348-01A | 11827.36 | 12588.69 | 24416.05 |
| TCGA-G4-6322-01A | 8094.515 | 10633.37 | 18727.88 |
| TCGA-AA-3821-01A | 9614.536 | 11548.93 | 21163.47 |
| TCGA-D5-5537-01A | 9114.076 | 10086.08 | 19200.16 |
| TCGA-AA-3518-01A | 8178.991 | 12277.68 | 20456.67 |
| TCGA-QG-A5YX-01A | 6233.108 | 9556.376 | 15789.48 |
| TCGA-G4-6586-01A | 6621.557 | 11774.48 | 18396.03 |
| TCGA-AA-3955-01A | 7949.812 | 10433.26 | 18383.07 |
| TCGA-AD-6548-01A | 10255.26 | 12560.21 | 22815.47 |
| TCGA-A6-2676-01A | 9760.937 | 13436.61 | 23197.55 |
| TCGA-D5-7000-01A | 9893.509 | 11788.77 | 21682.28 |
| TCGA-AA-3562-01A | 9306.539 | 11417.65 | 20724.19 |
| TCGA-AA-3514-01A | 10010.79 | 10944.46 | 20955.25 |
| TCGA-AZ-5407-01A | 7272.159 | 11490.84 | 18763 |
| TCGA-AZ-4615-01A | 10395.02 | 13219.43 | 23614.45 |
| TCGA-A6-5661-01B | 9876.403 | 12037.54 | 21913.94 |
| TCGA-AA-3818-01A | 8534.064 | 10850.24 | 19384.31 |
| TCGA-DM-A1D6-01A | 5715.719 | 7575.664 | 13291.38 |
| TCGA-AA-3516-01A | 8763.876 | 12110.94 | 20874.81 |
| TCGA-G4-6307-01A | 6070.184 | 8872.682 | 14942.87 |
| TCGA-AA-3851-01A | 9302.719 | 12171.98 | 21474.7 |
| TCGA-AA-3858-01A | 9460.731 | 11257.8 | 20718.53 |
| TCGA-CM-6675-01A | 7931.914 | 9920.023 | 17851.94 |
| TCGA-AA-A029-01A | 6923.284 | 9705.062 | 16628.35 |
| TCGA-AA-3525-01A | 7780.761 | 11405.04 | 19185.8 |
| TCGA-AA-3521-01A | 9045.282 | 10840.31 | 19885.59 |
| TCGA-AA-3556-01A | 8993.673 | 11542.09 | 20535.76 |
| TCGA-AD-6895-01A | 9799.697 | 12271.13 | 22070.83 |
| TCGA-D5-6540-01A | 8546.467 | 11533.85 | 20080.32 |
| TCGA-D5-6920-01A | 8814.337 | 12092.16 | 20906.49 |
| TCGA-AD-6889-01A | 7750.146 | 10506.07 | 18256.22 |
| TCGA-AA-3560-01A | 9249.149 | 11696.12 | 20945.26 |
| TCGA-CK-4947-01B | 10329.82 | 12562.83 | 22892.65 |
| TCGA-AA-3680-01A | 8425.513 | 11638.3 | 20063.81 |
| TCGA-D5-6537-01A | 7019.006 | 10416.18 | 17435.19 |
| TCGA-NH-A6GB-01A | 8122.558 | 11448.81 | 19571.37 |
| TCGA-G4-6323-01A | 6587.729 | 12207.39 | 18795.12 |
| TCGA-AA-3968-01A | 10128.74 | 11747.65 | 21876.39 |
| TCGA-A6-6138-01A | 11117.45 | 13591.14 | 24708.59 |
| TCGA-QG-A5YV-01A | 7158.694 | 10101.92 | 17260.61 |
| TCGA-AA-3850-01A | 9621.316 | 12603.8 | 22225.12 |
| TCGA-AZ-6607-01A | 12353.99 | 12693.66 | 25047.66 |
| TCGA-AA-A02R-01A | 10148.11 | 13680.93 | 23829.03 |
| TCGA-AZ-4614-01A | 5962.893 | 9717.92 | 15680.81 |
| TCGA-CM-6680-01A | 10343.48 | 12211.08 | 22554.56 |
| TCGA-AZ-6601-01A | 10654.73 | 13339.83 | 23994.56 |
| TCGA-G4-6627-01A | 11029.01 | 12895.08 | 23924.09 |
| TCGA-AD-A5EK-01A | 7885.407 | 9257.231 | 17142.64 |
| TCGA-AA-3870-01A | 10886.4 | 12570.47 | 23456.86 |
| TCGA-QG-A5YW-01A | 8761.604 | 11717.93 | 20479.53 |
| TCGA-AA-3819-01A | 8156.672 | 10643.71 | 18800.38 |
| TCGA-AZ-6606-01A | 6770.097 | 9958.545 | 16728.64 |
| TCGA-AA-3814-01A | 10846.07 | 12844.06 | 23690.13 |
| TCGA-CM-6166-01A | 8471.405 | 8861.955 | 17333.36 |
| TCGA-AA-A00U-01A | 7826.865 | 10334.68 | 18161.55 |
| TCGA-AA-3552-01A | 9006.627 | 12624.26 | 21630.89 |
| TCGA-CM-6163-01A | 10440.58 | 12371.92 | 22812.5 |
| TCGA-G4-6320-01A | 6537.144 | 10071.3 | 16608.45 |
| TCGA-A6-3807-01A | 9676.853 | 12007.73 | 21684.58 |
| TCGA-D5-5540-01A | 7950.507 | 10291.97 | 18242.47 |
| TCGA-WS-AB45-01A | 14186.4 | 14899.4 | 29085.8 |
| TCGA-AA-3673-01A | 9097.858 | 11092.78 | 20190.64 |
| TCGA-A6-6782-01A | 11391.42 | 12525.61 | 23917.03 |
| TCGA-CA-6719-01A | 10988.17 | 11894.22 | 22882.39 |
| TCGA-CA-5797-01A | 9978.74 | 11027.19 | 21005.93 |
| TCGA-A6-6780-01B | 10049.62 | 12949.91 | 22999.53 |
| TCGA-AA-3971-01A | 8991.44 | 12512.37 | 21503.81 |
| TCGA-AA-3684-01A | 11101.83 | 12906.03 | 24007.86 |
| TCGA-T9-A92H-01A | 6751.104 | 9460.643 | 16211.75 |
| TCGA-AG-3731-01A | 12055.51 | 13318.82 | 25374.33 |
| TCGA-F5-6861-01A | 8594.981 | 10283.96 | 18878.94 |
| TCGA-AG-3894-01A | 8501.254 | 10672.41 | 19173.66 |
| TCGA-EI-6885-01A | 10597.77 | 12020.82 | 22618.58 |
| TCGA-AG-3885-01A | 8239.811 | 11539.36 | 19779.17 |
| TCGA-EI-6508-01A | 8583.027 | 10090.11 | 18673.14 |
| TCGA-EI-6509-01A | 8690.826 | 9410.327 | 18101.15 |
| TCGA-AG-3883-01A | 10893.99 | 12411.3 | 23305.29 |
| TCGA-DY-A1DE-01A | 9003.583 | 11015.52 | 20019.1 |
| TCGA-AG-4022-01A | 10722.46 | 12329.62 | 23052.08 |
| TCGA-AG-A026-01A | 8126.714 | 9074.139 | 17200.85 |
| TCGA-EF-5831-01A | 9991.81 | 10919.02 | 20910.83 |
| TCGA-AG-3726-01A | 8612.869 | 10764.56 | 19377.42 |
| TCGA-F5-6863-01A | 8246.134 | 8913.985 | 17160.12 |
| TCGA-AG-A008-01A | 5246.55 | 9532.313 | 14778.86 |
| TCGA-F5-6571-01A | 11300.09 | 12928.12 | 24228.22 |
| TCGA-AG-A01Y-01A | 9015.548 | 11385 | 20400.54 |
| TCGA-AG-A02X-01A | 6204.83 | 9357.868 | 15562.7 |
| TCGA-DC-6158-01A | 12012.15 | 12665.05 | 24677.2 |
| TCGA-AG-A02N-01A | 6540.683 | 11032.44 | 17573.12 |
| TCGA-F5-6810-01A | 10602.05 | 10513.33 | 21115.38 |
| TCGA-CI-6623-01B | 8689.45 | 11041.05 | 19730.5 |
| TCGA-CI-6620-01A | 9502.917 | 10741.87 | 20244.79 |
| TCGA-CI-6619-01B | 9188.536 | 11701.89 | 20890.43 |
| TCGA-EI-6917-01A | 11297.17 | 13561.99 | 24859.16 |
| TCGA-AG-3898-01A | 9612.269 | 11873.38 | 21485.65 |
| TCGA-EI-6511-01A | 10919.01 | 13679.43 | 24598.44 |
| TCGA-AG-3728-01A | 10404.44 | 12882.1 | 23286.53 |
| TCGA-CI-6622-01A | 7904.093 | 9843.892 | 17747.99 |
| TCGA-DY-A1DG-01A | 5261.067 | 8728.845 | 13989.91 |
| TCGA-AG-A011-01A | 8532.03 | 10463.72 | 18995.75 |
| TCGA-AG-A015-01A | 6937.511 | 9527.902 | 16465.41 |
| TCGA-AG-A00C-01A | 5075.531 | 9737.28 | 14812.81 |
| TCGA-AH-6549-01A | 9957.063 | 10680.09 | 20637.16 |
| TCGA-EI-6882-01A | 8596.251 | 11423.71 | 20019.96 |
| TCGA-AG-3890-01A | 9099.412 | 12254.93 | 21354.35 |
| TCGA-F5-6864-01A | 11302.16 | 11662.26 | 22964.42 |
| TCGA-DY-A1H8-01A | 4996.05 | 8856.523 | 13852.57 |
| TCGA-AG-A016-01A | 6779.991 | 8464.543 | 15244.54 |
| TCGA-EI-6507-01A | 10298.12 | 13002.69 | 23300.82 |
| TCGA-EI-6513-01A | 8820.707 | 10569.73 | 19390.43 |
| TCGA-AG-A020-01A | 5833.102 | 9199.214 | 15032.32 |
| TCGA-AG-3587-01A | 8986.533 | 11034.3 | 20020.84 |
| TCGA-AG-3608-01A | 8961.355 | 12244.9 | 21206.25 |
| TCGA-EI-7002-01A | 9897.741 | 10824.8 | 20722.54 |
| TCGA-AG-3878-01A | 10809.94 | 12867.47 | 23677.42 |
| TCGA-AG-3887-01A | 8652.936 | 11425.9 | 20078.84 |
| TCGA-AG-3575-01A | 10576.69 | 12203.94 | 22780.63 |
| TCGA-F5-6814-01A | 9480.51 | 11721.26 | 21201.77 |
| TCGA-EI-6884-01A | 10353.3 | 12344.93 | 22698.23 |
| TCGA-AG-A025-01A | 8459.681 | 10269.69 | 18729.37 |
| TCGA-EI-6506-01A | 8881.103 | 12021.74 | 20902.84 |
| TCGA-EF-5830-01A | 7948.761 | 10378.49 | 18327.25 |
| TCGA-AG-A036-01A | 8540.756 | 10480.08 | 19020.84 |
| TCGA-AG-3612-01A | 10101.47 | 11685.04 | 21786.51 |
| TCGA-G5-6233-01A | 8631.223 | 10682.43 | 19313.65 |
| TCGA-AG-3574-01A | 8780.292 | 10897.22 | 19677.51 |
| TCGA-DC-5869-01A | 9617.125 | 10581.99 | 20199.12 |
| TCGA-CL-5918-01A | 6919.306 | 10331.12 | 17250.43 |
| TCGA-AG-3896-01A | 9474.171 | 11292.08 | 20766.25 |
| TCGA-AG-4021-01A | 10355.24 | 10681.28 | 21036.51 |
| TCGA-AG-A01L-01A | 7299.306 | 9876.36 | 17175.67 |
| TCGA-EI-6512-01A | 8762.599 | 11162.34 | 19924.94 |
| TCGA-EI-6510-01A | 6146.066 | 10538.86 | 16684.93 |
| TCGA-AG-A002-01A | 6705.567 | 8562.369 | 15267.94 |
| TCGA-AF-4110-01A | 10733.38 | 12894.82 | 23628.21 |
| TCGA-AG-A02G-01A | 6626.852 | 10084.47 | 16711.33 |
| TCGA-AF-3911-01A | 9784.161 | 11070.97 | 20855.14 |
| TCGA-EI-6883-01A | 8335.717 | 11048.64 | 19384.36 |
| TCGA-BM-6198-01A | 10266.87 | 12766.85 | 23033.72 |
| TCGA-AF-2692-01A | 8936.341 | 11048.55 | 19984.89 |
| TCGA-CI-6624-01C | 10703.65 | 12277.21 | 22980.86 |
| TCGA-AG-A01W-01A | 7440.735 | 10718.87 | 18159.61 |
| TCGA-AG-4005-01A | 9571.542 | 10674.52 | 20246.06 |
| TCGA-AG-3584-01A | 9507.687 | 11505.83 | 21013.52 |
| TCGA-AF-2690-01A | 13567.94 | 14556.52 | 28124.46 |
| TCGA-CL-4957-01A | 8569.847 | 9916.3 | 18486.15 |
| TCGA-CI-6621-01A | 10384.6 | 12323.25 | 22707.85 |
| TCGA-EI-6514-01A | 9943.696 | 10713.95 | 20657.64 |
| TCGA-AG-3892-01A | 8501.413 | 12943.78 | 21445.19 |
| TCGA-DT-5265-01A | 10940.11 | 11564.2 | 22504.32 |
| TCGA-EI-6881-01A | 7745.452 | 10880.3 | 18625.75 |
| TCGA-AG-A01N-01A | 7198.874 | 8866.136 | 16065.01 |
