# Supplementary material for: Cuproptosis‐related miRNAs signature and immune infiltration characteristics in colorectal cancer
Source: Cancer Med. 2023 Jun 19;12(15):16661–78. doi: 10.1002/cam4.6270 (PMC10469834; doi:10.1002/cam4.6270)
Supplement: Supplementary file 7 — Table S6 [file CAM4-12-16661-s002.docx]

TABLE S6 The CMS subtype of all the samples.

| ID | type |
| --- | --- |
| TCGA-AD-6901 | CMS4 |
| TCGA-A6-6142 | CMS4 |
| TCGA-AZ-5403 | CMS4 |
| TCGA-CM-6167 | CMS4 |
| TCGA-AU-3779 | CMS4 |
| TCGA-AZ-6600 | CMS4 |
| TCGA-CM-6679 | CMS4 |
| TCGA-D5-6529 | CMS4 |
| TCGA-G4-6302 | CMS4 |
| TCGA-D5-6922 | CMS4 |
| TCGA-A6-5662 | CMS4 |
| TCGA-CK-6748 | CMS4 |
| TCGA-AY-4070 | CMS4 |
| TCGA-F4-6807 | CMS4 |
| TCGA-AA-3712 | CMS4 |
| TCGA-A6-5664 | CMS4 |
| TCGA-A6-6781 | CMS4 |
| TCGA-F4-6704 | CMS4 |
| TCGA-D5-6923 | CMS4 |
| TCGA-D5-6898 | CMS4 |
| TCGA-5M-AATA | CMS4 |
| TCGA-F4-6463 | CMS4 |
| TCGA-AZ-6605 | CMS4 |
| TCGA-NH-A8F8 | CMS4 |
| TCGA-AA-A00O | CMS4 |
| TCGA-D5-6924 | CMS4 |
| TCGA-D5-5538 | CMS4 |
| TCGA-5M-AAT6 | CMS4 |
| TCGA-DM-A285 | CMS4 |
| TCGA-A6-2674 | CMS4 |
| TCGA-D5-6929 | CMS4 |
| TCGA-A6-4105 | CMS4 |
| TCGA-CM-6676 | CMS4 |
| TCGA-AA-3842 | CMS4 |
| TCGA-DM-A28A | CMS4 |
| TCGA-F4-6461 | CMS4 |
| TCGA-F4-6809 | CMS4 |
| TCGA-A6-2684 | CMS4 |
| TCGA-CA-6717 | CMS4 |
| TCGA-A6-5667 | CMS4 |
| TCGA-A6-6654 | CMS4 |
| TCGA-F4-6855 | CMS4 |
| TCGA-AM-5820 | CMS4 |
| TCGA-A6-A5ZU | CMS4 |
| TCGA-F4-6854 | CMS4 |
| TCGA-AA-3866 | CMS4 |
| TCGA-F4-6459 | CMS4 |
| TCGA-CM-6162 | CMS4 |
| TCGA-A6-2671 | CMS4 |
| TCGA-A6-2681 | CMS4 |
| TCGA-AY-6196 | CMS4 |
| TCGA-AA-3984 | CMS4 |
| TCGA-CM-6168 | CMS4 |
| TCGA-AD-6899 | CMS4 |
| TCGA-AA-3489 | CMS4 |
| TCGA-D5-6926 | CMS4 |
| TCGA-AA-3553 | CMS4 |
| TCGA-CM-5341 | CMS4 |
| TCGA-AA-3520 | CMS4 |
| TCGA-A6-5657 | CMS4 |
| TCGA-AA-3860 | CMS4 |
| TCGA-AA-3950 | CMS4 |
| TCGA-A6-3810 | CMS4 |
| TCGA-CM-5860 | CMS4 |
| TCGA-D5-6536 | CMS4 |
| TCGA-A6-A567 | CMS4 |
| TCGA-G4-6314 | CMS4 |
| TCGA-AA-3511 | CMS4 |
| TCGA-AA-3952 | CMS4 |
| TCGA-AA-3527 | CMS4 |
| TCGA-CM-6165 | CMS4 |
| TCGA-A6-2675 | CMS4 |
| TCGA-CM-5344 | CMS4 |
| TCGA-AA-3973 | CMS4 |
| TCGA-CM-5349 | CMS4 |
| TCGA-AA-3867 | CMS4 |
| TCGA-CM-6677 | CMS4 |
| TCGA-AA-3496 | CMS4 |
| TCGA-G4-6625 | CMS4 |
| TCGA-CM-4747 | CMS4 |
| TCGA-AZ-6603 | CMS4 |
| TCGA-NH-A50V | CMS4 |
| TCGA-F4-6569 | CMS4 |
| TCGA-D5-6932 | CMS4 |
| TCGA-AA-3532 | CMS4 |
| TCGA-G4-6310 | CMS4 |
| TCGA-A6-A565 | CMS4 |
| TCGA-A6-3808 | CMS4 |
| TCGA-D5-5541 | CMS4 |
| TCGA-F4-6703 | CMS4 |
| TCGA-CM-6170 | CMS4 |
| TCGA-A6-2682 | CMS4 |
| TCGA-D5-6534 | CMS4 |
| TCGA-F4-6460 | CMS4 |
| TCGA-G4-6298 | CMS4 |
| TCGA-A6-A56B | CMS4 |
| TCGA-AZ-4323 | CMS4 |
| TCGA-G4-6297 | CMS4 |
| TCGA-D5-6541 | CMS4 |
| TCGA-A6-6649 | CMS4 |
| TCGA-AD-6964 | CMS4 |
| TCGA-CM-5868 | CMS4 |
| TCGA-A6-A566 | CMS4 |
| TCGA-AZ-4308 | CMS4 |
| TCGA-AA-3812 | CMS4 |
| TCGA-G4-6311 | CMS4 |
| TCGA-G4-6303 | CMS4 |
| TCGA-A6-6651 | CMS4 |
| TCGA-AA-3872 | CMS4 |
| TCGA-CK-4948 | CMS4 |
| TCGA-A6-2685 | CMS4 |
| TCGA-CM-6169 | CMS4 |
| TCGA-F4-6805 | CMS4 |
| TCGA-CM-5348 | CMS4 |
| TCGA-AD-6548 | CMS4 |
| TCGA-AA-3514 | CMS4 |
| TCGA-CK-4947 | CMS4 |
| TCGA-AA-3968 | CMS4 |
| TCGA-A6-6138 | CMS4 |
| TCGA-AZ-6607 | CMS4 |
| TCGA-G4-6627 | CMS4 |
| TCGA-AA-3870 | CMS4 |
| TCGA-AA-3814 | CMS4 |
| TCGA-CM-6163 | CMS4 |
| TCGA-A6-3807 | CMS4 |
| TCGA-WS-AB45 | CMS4 |
| TCGA-A6-6782 | CMS4 |
| TCGA-CA-6719 | CMS4 |
| TCGA-CA-5797 | CMS4 |
| TCGA-AA-3684 | CMS4 |
| TCGA-AG-3731 | CMS4 |
| TCGA-EI-6885 | CMS4 |
| TCGA-EI-6509 | CMS4 |
| TCGA-AG-3883 | CMS4 |
| TCGA-AG-4022 | CMS4 |
| TCGA-AG-A026 | CMS4 |
| TCGA-EF-5831 | CMS4 |
| TCGA-F5-6863 | CMS4 |
| TCGA-F5-6571 | CMS4 |
| TCGA-AG-A01Y | CMS4 |
| TCGA-DC-6158 | CMS4 |
| TCGA-F5-6810 | CMS4 |
| TCGA-CI-6620 | CMS4 |
| TCGA-CI-6619 | CMS4 |
| TCGA-EI-6917 | CMS4 |
| TCGA-AG-3898 | CMS4 |
| TCGA-EI-6511 | CMS4 |
| TCGA-AG-3728 | CMS4 |
| TCGA-AH-6549 | CMS4 |
| TCGA-F5-6864 | CMS4 |
| TCGA-AG-3878 | CMS4 |
| TCGA-AG-3575 | CMS4 |
| TCGA-EI-6884 | CMS4 |
| TCGA-AG-3612 | CMS4 |
| TCGA-AG-4021 | CMS4 |
| TCGA-AF-4110 | CMS4 |
| TCGA-BM-6198 | CMS4 |
| TCGA-CI-6624 | CMS4 |
| TCGA-AG-4005 | CMS4 |
| TCGA-AF-2690 | CMS4 |
| TCGA-CI-6621 | CMS4 |
| TCGA-EI-6514 | CMS4 |
| TCGA-DT-5265 | CMS4 |
| TCGA-CM-5863 | CMS3 |
| TCGA-AY-A71X | CMS3 |
| TCGA-NH-A5IV | CMS3 |
| TCGA-AA-3861 | CMS3 |
| TCGA-D5-5539 | CMS3 |
| TCGA-AA-3864 | CMS3 |
| TCGA-AA-3555 | CMS3 |
| TCGA-A6-2678 | CMS3 |
| TCGA-AA-3663 | CMS3 |
| TCGA-AZ-6599 | CMS3 |
| TCGA-AA-3977 | CMS3 |
| TCGA-DM-A28M | CMS3 |
| TCGA-AA-3675 | CMS3 |
| TCGA-DM-A28K | CMS3 |
| TCGA-AA-3522 | CMS3 |
| TCGA-DM-A28G | CMS3 |
| TCGA-A6-6141 | CMS3 |
| TCGA-A6-4107 | CMS3 |
| TCGA-F4-6856 | CMS3 |
| TCGA-AY-A69D | CMS3 |
| TCGA-AA-A02Y | CMS3 |
| TCGA-AA-A01V | CMS3 |
| TCGA-CK-6747 | CMS3 |
| TCGA-AZ-4313 | CMS3 |
| TCGA-AA-3854 | CMS3 |
| TCGA-AA-3655 | CMS3 |
| TCGA-AY-6386 | CMS3 |
| TCGA-CK-6751 | CMS3 |
| TCGA-CA-5796 | CMS3 |
| TCGA-AA-3692 | CMS3 |
| TCGA-AA-3837 | CMS3 |
| TCGA-F4-6806 | CMS3 |
| TCGA-CM-4746 | CMS3 |
| TCGA-CM-6172 | CMS3 |
| TCGA-CM-6678 | CMS3 |
| TCGA-AA-3852 | CMS3 |
| TCGA-AA-A02O | CMS3 |
| TCGA-AA-3939 | CMS3 |
| TCGA-AA-A03F | CMS3 |
| TCGA-AA-A024 | CMS3 |
| TCGA-D5-6535 | CMS3 |
| TCGA-G4-6321 | CMS3 |
| TCGA-D5-6539 | CMS3 |
| TCGA-AA-3994 | CMS3 |
| TCGA-CK-4950 | CMS3 |
| TCGA-4T-AA8H | CMS3 |
| TCGA-AA-3548 | CMS3 |
| TCGA-CK-4952 | CMS3 |
| TCGA-QG-A5Z2 | CMS3 |
| TCGA-NH-A6GC | CMS3 |
| TCGA-AA-3502 | CMS3 |
| TCGA-G4-6322 | CMS3 |
| TCGA-QG-A5YX | CMS3 |
| TCGA-AZ-5407 | CMS3 |
| TCGA-AA-3818 | CMS3 |
| TCGA-DM-A1D6 | CMS3 |
| TCGA-AA-3851 | CMS3 |
| TCGA-AA-A029 | CMS3 |
| TCGA-AA-3556 | CMS3 |
| TCGA-D5-6920 | CMS3 |
| TCGA-AA-3560 | CMS3 |
| TCGA-AA-3680 | CMS3 |
| TCGA-G4-6323 | CMS3 |
| TCGA-AA-3850 | CMS3 |
| TCGA-CM-6680 | CMS3 |
| TCGA-QG-A5YW | CMS3 |
| TCGA-AA-A00U | CMS3 |
| TCGA-G4-6320 | CMS3 |
| TCGA-EI-6508 | CMS3 |
| TCGA-AG-A008 | CMS3 |
| TCGA-AG-A02X | CMS3 |
| TCGA-AG-A02N | CMS3 |
| TCGA-AG-A015 | CMS3 |
| TCGA-EI-6882 | CMS3 |
| TCGA-AG-A020 | CMS3 |
| TCGA-AG-3887 | CMS3 |
| TCGA-EI-6506 | CMS3 |
| TCGA-EI-6510 | CMS3 |
| TCGA-AA-3679 | CMS2 |
| TCGA-AA-3662 | CMS2 |
| TCGA-AA-3667 | CMS2 |
| TCGA-CA-5256 | CMS2 |
| TCGA-AA-A02H | CMS2 |
| TCGA-AA-3989 | CMS2 |
| TCGA-AA-A00W | CMS2 |
| TCGA-AA-3495 | CMS2 |
| TCGA-AA-3519 | CMS2 |
| TCGA-G4-6293 | CMS2 |
| TCGA-CK-5915 | CMS2 |
| TCGA-AA-3697 | CMS2 |
| TCGA-AZ-6608 | CMS2 |
| TCGA-AA-A00Q | CMS2 |
| TCGA-AY-A8YK | CMS2 |
| TCGA-A6-6137 | CMS2 |
| TCGA-AA-3494 | CMS2 |
| TCGA-AA-3846 | CMS2 |
| TCGA-CM-4752 | CMS2 |
| TCGA-AA-3531 | CMS2 |
| TCGA-AA-3529 | CMS2 |
| TCGA-AA-A00L | CMS2 |
| TCGA-5M-AATE | CMS2 |
| TCGA-DM-A0XF | CMS2 |
| TCGA-AA-3506 | CMS2 |
| TCGA-AA-3534 | CMS2 |
| TCGA-AD-6890 | CMS2 |
| TCGA-A6-2683 | CMS2 |
| TCGA-DM-A1D0 | CMS2 |
| TCGA-5M-AAT5 | CMS2 |
| TCGA-DM-A28F | CMS2 |
| TCGA-AA-3972 | CMS2 |
| TCGA-CM-6161 | CMS2 |
| TCGA-CA-6715 | CMS2 |
| TCGA-G4-6317 | CMS2 |
| TCGA-DM-A282 | CMS2 |
| TCGA-AD-6963 | CMS2 |
| TCGA-A6-6652 | CMS2 |
| TCGA-AA-3696 | CMS2 |
| TCGA-AA-3542 | CMS2 |
| TCGA-NH-A50T | CMS2 |
| TCGA-A6-5666 | CMS2 |
| TCGA-AA-3956 | CMS2 |
| TCGA-AA-A03J | CMS2 |
| TCGA-CM-5862 | CMS2 |
| TCGA-AA-3544 | CMS2 |
| TCGA-AY-4071 | CMS2 |
| TCGA-AA-3538 | CMS2 |
| TCGA-AA-3986 | CMS2 |
| TCGA-AA-3526 | CMS2 |
| TCGA-AA-A01S | CMS2 |
| TCGA-AA-3530 | CMS2 |
| TCGA-D5-6538 | CMS2 |
| TCGA-A6-5660 | CMS2 |
| TCGA-A6-2677 | CMS2 |
| TCGA-AA-3685 | CMS2 |
| TCGA-DM-A0X9 | CMS2 |
| TCGA-G4-6294 | CMS2 |
| TCGA-3L-AA1B | CMS2 |
| TCGA-CM-5864 | CMS2 |
| TCGA-A6-6140 | CMS2 |
| TCGA-AA-3869 | CMS2 |
| TCGA-AA-3970 | CMS2 |
| TCGA-AA-3660 | CMS2 |
| TCGA-AA-3831 | CMS2 |
| TCGA-AA-A01X | CMS2 |
| TCGA-A6-6648 | CMS2 |
| TCGA-F4-6808 | CMS2 |
| TCGA-D5-6533 | CMS2 |
| TCGA-AA-3517 | CMS2 |
| TCGA-CM-6164 | CMS2 |
| TCGA-CK-5914 | CMS2 |
| TCGA-AA-3693 | CMS2 |
| TCGA-AA-A02F | CMS2 |
| TCGA-AA-3975 | CMS2 |
| TCGA-AA-A01G | CMS2 |
| TCGA-AA-3855 | CMS2 |
| TCGA-CA-6716 | CMS2 |
| TCGA-AA-A02J | CMS2 |
| TCGA-AD-6965 | CMS2 |
| TCGA-AA-3524 | CMS2 |
| TCGA-AA-A01T | CMS2 |
| TCGA-A6-2680 | CMS2 |
| TCGA-AA-3875 | CMS2 |
| TCGA-AD-6888 | CMS2 |
| TCGA-DM-A1DB | CMS2 |
| TCGA-A6-2679 | CMS2 |
| TCGA-D5-6532 | CMS2 |
| TCGA-AA-3688 | CMS2 |
| TCGA-G4-6315 | CMS2 |
| TCGA-AA-3509 | CMS2 |
| TCGA-G4-6306 | CMS2 |
| TCGA-AA-3549 | CMS2 |
| TCGA-AA-3678 | CMS2 |
| TCGA-AA-3848 | CMS2 |
| TCGA-AA-3856 | CMS2 |
| TCGA-D5-5537 | CMS2 |
| TCGA-AA-3955 | CMS2 |
| TCGA-AA-3562 | CMS2 |
| TCGA-G4-6307 | CMS2 |
| TCGA-AA-3858 | CMS2 |
| TCGA-AA-3521 | CMS2 |
| TCGA-D5-6537 | CMS2 |
| TCGA-QG-A5YV | CMS2 |
| TCGA-AA-3819 | CMS2 |
| TCGA-CM-6166 | CMS2 |
| TCGA-AA-3552 | CMS2 |
| TCGA-AA-3673 | CMS2 |
| TCGA-AA-3971 | CMS2 |
| TCGA-T9-A92H | CMS2 |
| TCGA-F5-6861 | CMS2 |
| TCGA-AG-3894 | CMS2 |
| TCGA-AG-3726 | CMS2 |
| TCGA-CI-6623 | CMS2 |
| TCGA-CI-6622 | CMS2 |
| TCGA-AG-A011 | CMS2 |
| TCGA-AG-3890 | CMS2 |
| TCGA-AG-A016 | CMS2 |
| TCGA-EI-6513 | CMS2 |
| TCGA-AG-3587 | CMS2 |
| TCGA-AG-3608 | CMS2 |
| TCGA-EI-7002 | CMS2 |
| TCGA-AG-A025 | CMS2 |
| TCGA-EF-5830 | CMS2 |
| TCGA-AG-A036 | CMS2 |
| TCGA-AG-3574 | CMS2 |
| TCGA-DC-5869 | CMS2 |
| TCGA-CL-5918 | CMS2 |
| TCGA-AG-3896 | CMS2 |
| TCGA-AG-A01L | CMS2 |
| TCGA-AF-3911 | CMS2 |
| TCGA-EI-6883 | CMS2 |
| TCGA-AF-2692 | CMS2 |
| TCGA-AG-3584 | CMS2 |
| TCGA-CL-4957 | CMS2 |
| TCGA-EI-6881 | CMS2 |
| TCGA-AG-A01N | CMS2 |
| TCGA-AA-3672 | CMS1 |
| TCGA-AZ-6598 | CMS1 |
| TCGA-F4-6570 | CMS1 |
| TCGA-D5-6530 | CMS1 |
| TCGA-G4-6299 | CMS1 |
| TCGA-AA-3877 | CMS1 |
| TCGA-AA-3949 | CMS1 |
| TCGA-A6-5665 | CMS1 |
| TCGA-AA-A00J | CMS1 |
| TCGA-CA-6718 | CMS1 |
| TCGA-CK-5913 | CMS1 |
| TCGA-CK-5916 | CMS1 |
| TCGA-AD-5900 | CMS1 |
| TCGA-AA-3664 | CMS1 |
| TCGA-AA-3554 | CMS1 |
| TCGA-AA-3713 | CMS1 |
| TCGA-D5-6531 | CMS1 |
| TCGA-AD-A5EJ | CMS1 |
| TCGA-AA-A022 | CMS1 |
| TCGA-AA-3930 | CMS1 |
| TCGA-A6-3809 | CMS1 |
| TCGA-AM-5821 | CMS1 |
| TCGA-AA-A01R | CMS1 |
| TCGA-DM-A1HB | CMS1 |
| TCGA-NH-A6GA | CMS1 |
| TCGA-CM-4751 | CMS1 |
| TCGA-AA-3710 | CMS1 |
| TCGA-D5-6930 | CMS1 |
| TCGA-CM-5861 | CMS1 |
| TCGA-G4-6309 | CMS1 |
| TCGA-CM-6171 | CMS1 |
| TCGA-D5-6927 | CMS1 |
| TCGA-G4-6588 | CMS1 |
| TCGA-AA-3492 | CMS1 |
| TCGA-AA-A00D | CMS1 |
| TCGA-AA-A01P | CMS1 |
| TCGA-A6-2686 | CMS1 |
| TCGA-CM-4743 | CMS1 |
| TCGA-G4-6628 | CMS1 |
| TCGA-AU-6004 | CMS1 |
| TCGA-D5-6928 | CMS1 |
| TCGA-D5-6931 | CMS1 |
| TCGA-CK-6746 | CMS1 |
| TCGA-DM-A0XD | CMS1 |
| TCGA-AA-3681 | CMS1 |
| TCGA-DM-A280 | CMS1 |
| TCGA-AA-3941 | CMS1 |
| TCGA-AA-3845 | CMS1 |
| TCGA-CM-6674 | CMS1 |
| TCGA-CK-4951 | CMS1 |
| TCGA-A6-5661 | CMS1 |
| TCGA-AA-3833 | CMS1 |
| TCGA-A6-6653 | CMS1 |
| TCGA-AA-3811 | CMS1 |
| TCGA-AA-3966 | CMS1 |
| TCGA-AA-3715 | CMS1 |
| TCGA-AY-6197 | CMS1 |
| TCGA-AA-A010 | CMS1 |
| TCGA-AA-3815 | CMS1 |
| TCGA-CM-4744 | CMS1 |
| TCGA-CA-5255 | CMS1 |
| TCGA-AZ-4616 | CMS1 |
| TCGA-AA-3543 | CMS1 |
| TCGA-AA-3821 | CMS1 |
| TCGA-AA-3518 | CMS1 |
| TCGA-G4-6586 | CMS1 |
| TCGA-A6-2676 | CMS1 |
| TCGA-D5-7000 | CMS1 |
| TCGA-AZ-4615 | CMS1 |
| TCGA-AA-3516 | CMS1 |
| TCGA-CM-6675 | CMS1 |
| TCGA-AA-3525 | CMS1 |
| TCGA-AD-6895 | CMS1 |
| TCGA-D5-6540 | CMS1 |
| TCGA-AD-6889 | CMS1 |
| TCGA-AA-A02R | CMS1 |
| TCGA-AZ-4614 | CMS1 |
| TCGA-AZ-6601 | CMS1 |
| TCGA-AZ-6606 | CMS1 |
| TCGA-EI-6507 | CMS1 |
| TCGA-F5-6814 | CMS1 |
| TCGA-G5-6233 | CMS1 |
| TCGA-EI-6512 | CMS1 |
| TCGA-AG-3892 | CMS1 |
