# Supplementary material for: Cuproptosis‐related miRNAs signature and immune infiltration characteristics in colorectal cancer
Source: Cancer Med. 2023 Jun 19;12(15):16661–78. doi: 10.1002/cam4.6270 (PMC10469834; doi:10.1002/cam4.6270)
Supplement: Supplementary file 8 — Table S7 [file CAM4-12-16661-s005.docx]

TABLE S7 The IPS of all the samples.

| Sample | ips_ctla4_neg_pd1_neg | ips_ctla4_neg_pd1_pos | ips_ctla4_pos_pd1_neg | ips_ctla4_pos_pd1_pos |
| --- | --- | --- | --- | --- |
| TCGA-QG-A5YX | 10 | 8 | 1.00E+01 | 8.00E+00 |
| TCGA-G4-6306 | 10 | 9 | 1.00E+01 | 9.00E+00 |
| TCGA-AA-A02E | 10 | 8 | 9.00E+00 | 8.00E+00 |
| TCGA-AA-A00L | 10 | 8 | 1.00E+01 | 8.00E+00 |
| TCGA-G4-6626 | 10 | 8 | 9.00E+00 | 8.00E+00 |
| TCGA-AZ-4308 | 10 | 8 | 9.00E+00 | 8.00E+00 |
| TCGA-DM-A28K | 10 | 8 | 1.00E+01 | 8.00E+00 |
| TCGA-AA-3531 | 10 | 9 | 1.00E+01 | 8.00E+00 |
| TCGA-G4-6294 | 10 | 8 | 9.00E+00 | 7.00E+00 |
| TCGA-AD-6963 | 10 | 9 | 1.00E+01 | 9.00E+00 |
| TCGA-A6-5661 | 10 | 8 | 9.00E+00 | 8.00E+00 |
| TCGA-AA-3530 | 10 | 8 | 9.00E+00 | 8.00E+00 |
| TCGA-AA-3851 | 10 | 8 | 9.00E+00 | 8.00E+00 |
| TCGA-A6-2679 | 10 | 10 | 1.00E+01 | 1.00E+01 |
| TCGA-AA-3837 | 10 | 8 | 9.00E+00 | 7.00E+00 |
| TCGA-AA-3971 | 10 | 8 | 9.00E+00 | 8.00E+00 |
| TCGA-AZ-4313 | 10 | 9 | 1.00E+01 | 8.00E+00 |
| TCGA-AA-3552 | 10 | 9 | 1.00E+01 | 8.00E+00 |
| TCGA-AA-3527 | 10 | 10 | 1.00E+01 | 9.00E+00 |
| TCGA-D5-6930 | 10 | 9 | 1.00E+01 | 9.00E+00 |
| TCGA-AA-A03F | 10 | 8 | 1.00E+01 | 8.00E+00 |
| TCGA-AA-3688 | 10 | 8 | 9.00E+00 | 8.00E+00 |
| TCGA-DM-A28F | 10 | 8 | 9.00E+00 | 7.00E+00 |
| TCGA-AY-4071 | 10 | 9 | 1.00E+01 | 9.00E+00 |
| TCGA-AA-3862 | 10 | 9 | 1.00E+01 | 9.00E+00 |
| TCGA-NH-A6GC | 10 | 8 | 9.00E+00 | 7.00E+00 |
| TCGA-AA-3519 | 10 | 8 | 9.00E+00 | 8.00E+00 |
| TCGA-AA-3502 | 10 | 8 | 9.00E+00 | 7.00E+00 |
| TCGA-AY-5543 | 10 | 8 | 9.00E+00 | 8.00E+00 |
| TCGA-DM-A1D4 | 10 | 8 | 9.00E+00 | 7.00E+00 |
| TCGA-QG-A5Z2 | 10 | 9 | 1.00E+01 | 9.00E+00 |
| TCGA-AA-A01S | 10 | 8 | 9.00E+00 | 7.00E+00 |
| TCGA-DM-A28E | 10 | 8 | 9.00E+00 | 7.00E+00 |
| TCGA-4N-A93T | 10 | 8 | 9.00E+00 | 7.00E+00 |
| TCGA-DM-A1D8 | 10 | 9 | 1.00E+01 | 8 |
| TCGA-AA-A01I | 10 | 8 | 1.00E+01 | 8 |
| TCGA-A6-5656 | 10 | 8 | 9.00E+00 | 7 |
| TCGA-AA-3848 | 10 | 8 | 9.00E+00 | 8 |
| TCGA-AA-A029 | 10 | 8 | 9.00E+00 | 8 |
| TCGA-AA-3856 | 10 | 9 | 9.00E+00 | 8 |
| TCGA-G4-6323 | 10 | 8 | 9.00E+00 | 8 |
| TCGA-CM-5861 | 10 | 9 | 1.00E+01 | 8 |
| TCGA-AA-A01R | 10 | 10 | 1.00E+01 | 10 |
| TCGA-D5-6532 | 10 | 8 | 9.00E+00 | 7 |
| TCGA-AA-3518 | 10 | 9 | 9.00E+00 | 8 |
| TCGA-CK-5912 | 10 | 8 | 9 | 7 |
| TCGA-AA-3516 | 10 | 10 | 10 | 10 |
| TCGA-AA-3846 | 10 | 9 | 10 | 9 |
| TCGA-G4-6320 | 10 | 8 | 9 | 7 |
| TCGA-DM-A1DA | 10 | 8 | 9 | 7 |
| TCGA-AA-3672 | 10 | 10 | 10 | 10 |
| TCGA-DM-A28M | 10 | 8 | 9 | 7 |
| TCGA-AY-6197 | 10 | 8 | 9 | 8 |
| TCGA-AA-A01T | 10 | 9 | 10 | 9 |
| TCGA-AA-A00W | 10 | 8 | 9 | 7 |
| TCGA-AA-A01G | 10 | 8 | 9 | 7 |
| TCGA-AZ-4681 | 10 | 8 | 9 | 7 |
| TCGA-A6-2683 | 10 | 8 | 9 | 8 |
| TCGA-AZ-6599 | 10 | 8 | 9 | 7 |
| TCGA-CM-6675 | 10 | 8 | 9 | 7 |
| TCGA-CK-6747 | 10 | 8 | 9 | 8 |
| TCGA-DM-A0X9 | 10 | 8 | 9 | 8 |
| TCGA-AA-A022 | 10 | 10 | 10 | 10 |
| TCGA-CK-6751 | 10 | 8 | 9 | 8 |
| TCGA-CM-4752 | 10 | 9 | 10 | 9 |
| TCGA-AD-6895 | 10 | 9 | 9 | 9 |
| TCGA-AA-3844 | 10 | 8 | 9 | 8 |
| TCGA-T9-A92H | 10 | 9 | 10 | 8 |
| TCGA-A6-3807 | 10 | 9 | 9 | 8 |
| TCGA-CA-5255 | 10 | 8 | 9 | 8 |
| TCGA-DM-A1HA | 10 | 9 | 10 | 8 |
| TCGA-CK-5914 | 10 | 9 | 10 | 8 |
| TCGA-AA-3861 | 10 | 8 | 9 | 8 |
| TCGA-RU-A8FL | 10 | 8 | 9 | 7 |
| TCGA-D5-6929 | 10 | 8 | 9 | 8 |
| TCGA-F4-6856 | 10 | 8 | 9 | 8 |
| TCGA-CA-5796 | 10 | 8 | 9 | 8 |
| TCGA-A6-6653 | 10 | 9 | 10 | 8 |
| TCGA-G4-6321 | 10 | 9 | 10 | 9 |
| TCGA-AA-3855 | 10 | 8 | 9 | 8 |
| TCGA-DM-A0XD | 10 | 8 | 9 | 7 |
| TCGA-DM-A1D6 | 10 | 8 | 9 | 7 |
| TCGA-AA-3679 | 10 | 8 | 9 | 7 |
| TCGA-AA-A004 | 10 | 9 | 10 | 9 |
| TCGA-D5-5537 | 10 | 8 | 9 | 7 |
| TCGA-CM-4750 | 10 | 9 | 10 | 8 |
| TCGA-F4-6806 | 10 | 9 | 10 | 8 |
| TCGA-AZ-6606 | 10 | 8 | 9 | 8 |
| TCGA-CM-4743 | 10 | 10 | 10 | 10 |
| TCGA-NH-A6GB | 10 | 8 | 9 | 8 |
| TCGA-QL-A97D | 10 | 9 | 10 | 9 |
| TCGA-A6-4107 | 10 | 8 | 9 | 7 |
| TCGA-AA-A01Z | 10 | 8 | 9 | 7 |
| TCGA-D5-6535 | 10 | 9 | 10 | 9 |
| TCGA-CM-4751 | 10 | 9 | 9 | 9 |
| TCGA-AZ-4682 | 10 | 8 | 9 | 7 |
| TCGA-AA-3525 | 10 | 9 | 10 | 8 |
| TCGA-AA-3684 | 10 | 9 | 9 | 8 |
| TCGA-AA-3522 | 10 | 8 | 9 | 8 |
| TCGA-CM-4744 | 10 | 10 | 10 | 9 |
| TCGA-AA-A03J | 10 | 9 | 10 | 9 |
| TCGA-AZ-4616 | 10 | 9 | 9 | 9 |
| TCGA-G4-6322 | 10 | 8 | 9 | 8 |
| TCGA-CM-4748 | 10 | 8 | 9 | 7 |
| TCGA-AA-A02Y | 10 | 9 | 9 | 8 |
| TCGA-A6-2680 | 10 | 8 | 9 | 8 |
| TCGA-AG-A023 | 10 | 8 | 10 | 8 |
| TCGA-EI-6883 | 10 | 8 | 9 | 8 |
| TCGA-AG-A020 | 10 | 8 | 9 | 7 |
| TCGA-DC-5337 | 10 | 8 | 9 | 8 |
| TCGA-DC-6682 | 10 | 8 | 9 | 8 |
| TCGA-AG-3599 | 10 | 9 | 10 | 8 |
| TCGA-EI-6510 | 10 | 8 | 9 | 8 |
| TCGA-AG-A01L | 10 | 8 | 9 | 7 |
| TCGA-AG-3728 | 10 | 9 | 10 | 9 |
| TCGA-AG-3882 | 10 | 9 | 10 | 9 |
| TCGA-AG-A02G | 10 | 8 | 9 | 8 |
| TCGA-AG-A032 | 10 | 8 | 10 | 8 |
| TCGA-AG-3602 | 10 | 9 | 10 | 8 |
| TCGA-AG-A036 | 10 | 8 | 10 | 8 |
| TCGA-G5-6235 | 10 | 8 | 9 | 8 |
| TCGA-AG-3608 | 10 | 9 | 10 | 8 |
| TCGA-AG-3598 | 10 | 9 | 9 | 8 |
| TCGA-AG-3611 | 10 | 9 | 10 | 8 |
| TCGA-AG-3909 | 10 | 8 | 9 | 7 |
| TCGA-DY-A1DG | 10 | 9 | 10 | 8 |
| TCGA-AG-3887 | 10 | 8 | 9 | 8 |
| TCGA-AG-4007 | 10 | 8 | 9 | 8 |
| TCGA-AF-2691 | 10 | 8 | 9 | 8 |
| TCGA-AH-6544 | 10 | 9 | 10 | 8 |
| TCGA-AG-3890 | 10 | 8 | 10 | 8 |
| TCGA-CI-6622 | 10 | 8 | 9 | 7 |
| TCGA-AG-A01J | 10 | 8 | 9 | 7 |
| TCGA-DC-4749 | 10 | 8 | 9 | 7 |
| TCGA-AG-3591 | 10 | 8 | 9 | 8 |
| TCGA-CL-4957 | 10 | 8 | 9 | 7 |
| TCGA-F5-6812 | 10 | 8 | 9 | 8 |
| TCGA-AG-3885 | 10 | 9 | 9 | 8 |
| TCGA-AG-A02X | 10 | 8 | 9 | 7 |
| TCGA-AG-A00C | 10 | 9 | 10 | 8 |
| TCGA-A6-6137 | 9 | 8 | 9 | 8 |
| TCGA-AU-6004 | 9 | 8 | 9 | 8 |
| TCGA-AZ-4614 | 9 | 7 | 9 | 7 |
| TCGA-AA-3693 | 9 | 7 | 8 | 6 |
| TCGA-AA-3510 | 9 | 8 | 9 | 8 |
| TCGA-AY-6386 | 9 | 8 | 9 | 7 |
| TCGA-5M-AAT5 | 9 | 7 | 9 | 7 |
| TCGA-CK-5913 | 9 | 8 | 9 | 8 |
| TCGA-AA-3980 | 9 | 8 | 9 | 8 |
| TCGA-A6-6649 | 9 | 7 | 8 | 7 |
| TCGA-AA-3869 | 9 | 8 | 9 | 8 |
| TCGA-CA-5797 | 9 | 7 | 8 | 7 |
| TCGA-A6-6782 | 9 | 7 | 8 | 7 |
| TCGA-AA-3675 | 9 | 7 | 8 | 7 |
| TCGA-AA-3858 | 9 | 8 | 9 | 7 |
| TCGA-AA-3812 | 9 | 8 | 9 | 7 |
| TCGA-A6-A5ZU | 9 | 8 | 8 | 7 |
| TCGA-NH-A50T | 9 | 7 | 9 | 7 |
| TCGA-F4-6808 | 9 | 7 | 9 | 7 |
| TCGA-CM-5868 | 9 | 7 | 9 | 7 |
| TCGA-A6-6138 | 9 | 9 | 9 | 9 |
| TCGA-AA-A00A | 9 | 8 | 9 | 8 |
| TCGA-NH-A8F8 | 9 | 7 | 8 | 7 |
| TCGA-AA-A02K | 9 | 7 | 8 | 6 |
| TCGA-AA-3872 | 9 | 7 | 8 | 7 |
| TCGA-AA-3562 | 9 | 7 | 9 | 7 |
| TCGA-AA-A01F | 9 | 8 | 9 | 7 |
| TCGA-AA-3850 | 9 | 8 | 9 | 8 |
| TCGA-A6-6140 | 9 | 8 | 9 | 7 |
| TCGA-G4-6299 | 9 | 8 | 9 | 8 |
| TCGA-AA-3821 | 9 | 8 | 9 | 8 |
| TCGA-AA-A00E | 9 | 8 | 9 | 8 |
| TCGA-A6-5666 | 9 | 7 | 8 | 6 |
| TCGA-CA-5254 | 9 | 8 | 9 | 7 |
| TCGA-G4-6293 | 9 | 8 | 9 | 8 |
| TCGA-CM-6172 | 9 | 8 | 9 | 7 |
| TCGA-AA-3506 | 9 | 7 | 8 | 7 |
| TCGA-F4-6461 | 9 | 8 | 9 | 8 |
| TCGA-F4-6460 | 9 | 7 | 8 | 7 |
| TCGA-AA-3712 | 9 | 8 | 9 | 7 |
| TCGA-G4-6628 | 9 | 9 | 9 | 9 |
| TCGA-AA-3560 | 9 | 8 | 9 | 7 |
| TCGA-CM-5341 | 9 | 7 | 8 | 7 |
| TCGA-AA-3548 | 9 | 8 | 9 | 7 |
| TCGA-AA-3561 | 9 | 7 | 9 | 7 |
| TCGA-AA-3655 | 9 | 7 | 8 | 7 |
| TCGA-AA-3866 | 9 | 8 | 8 | 8 |
| TCGA-AA-3524 | 9 | 8 | 9 | 8 |
| TCGA-AA-3877 | 9 | 8 | 9 | 8 |
| TCGA-AA-3986 | 9 | 8 | 9 | 8 |
| TCGA-AA-A00D | 9 | 9 | 9 | 9 |
| TCGA-AA-A00U | 9 | 7 | 8 | 7 |
| TCGA-AA-A01P | 9 | 9 | 9 | 9 |
| TCGA-AD-6888 | 9 | 7 | 8 | 7 |
| TCGA-DM-A288 | 9 | 7 | 8 | 6 |
| TCGA-G4-6310 | 9 | 7 | 8 | 6 |
| TCGA-AA-3976 | 9 | 7 | 8 | 7 |
| TCGA-AZ-6605 | 9 | 8 | 8 | 7 |
| TCGA-A6-A567 | 9 | 7 | 8 | 7 |
| TCGA-AA-3495 | 9 | 7 | 8 | 7 |
| TCGA-AA-3973 | 9 | 8 | 9 | 7 |
| TCGA-G4-6588 | 9 | 7 | 8 | 7 |
| TCGA-F4-6807 | 9 | 8 | 9 | 8 |
| TCGA-AA-3972 | 9 | 7 | 8 | 7 |
| TCGA-AA-3511 | 9 | 7 | 9 | 7 |
| TCGA-CA-5256 | 9 | 8 | 9 | 7 |
| TCGA-AA-3526 | 9 | 8 | 9 | 8 |
| TCGA-AD-6889 | 9 | 8 | 9 | 7 |
| TCGA-A6-5662 | 9 | 8 | 8 | 7 |
| TCGA-AA-A01V | 9 | 9 | 10 | 9 |
| TCGA-AA-A00K | 9 | 7 | 9 | 7 |
| TCGA-CK-6746 | 9 | 9 | 9 | 9 |
| TCGA-AA-A01K | 9 | 8 | 9 | 8 |
| TCGA-G4-6627 | 9 | 8 | 9 | 7 |
| TCGA-AA-3819 | 9 | 7 | 8 | 6 |
| TCGA-A6-6652 | 9 | 7 | 9 | 7 |
| TCGA-CM-5860 | 9 | 8 | 9 | 8 |
| TCGA-CM-6677 | 9 | 7 | 8 | 7 |
| TCGA-A6-5659 | 9 | 7 | 8 | 6 |
| TCGA-AA-3975 | 9 | 7 | 9 | 7 |
| TCGA-AA-3532 | 9 | 9 | 9 | 9 |
| TCGA-DM-A28A | 9 | 7 | 8 | 7 |
| TCGA-AA-3681 | 9 | 8 | 8 | 7 |
| TCGA-AA-3860 | 9 | 8 | 9 | 8 |
| TCGA-AY-A69D | 9 | 7 | 8 | 7 |
| TCGA-AZ-4323 | 9 | 8 | 9 | 8 |
| TCGA-F4-6704 | 9 | 8 | 8 | 7 |
| TCGA-AY-A8YK | 9 | 7 | 9 | 7 |
| TCGA-AZ-6608 | 9 | 7 | 9 | 7 |
| TCGA-AD-6548 | 9 | 8 | 8 | 7 |
| TCGA-AA-3952 | 9 | 7 | 8 | 6 |
| TCGA-AA-3811 | 9 | 8 | 9 | 8 |
| TCGA-AY-A54L | 9 | 7 | 8 | 6 |
| TCGA-A6-2677 | 9 | 7 | 8 | 6 |
| TCGA-AA-3814 | 9 | 7 | 8 | 7 |
| TCGA-AA-3680 | 9 | 7 | 9 | 7 |
| TCGA-CK-4950 | 9 | 8 | 9 | 8 |
| TCGA-AA-3662 | 9 | 8 | 8 | 7 |
| TCGA-AA-3710 | 9 | 9 | 8 | 8 |
| TCGA-AA-3517 | 9 | 7 | 9 | 7 |
| TCGA-A6-5657 | 9 | 8 | 9 | 8 |
| TCGA-D5-6920 | 9 | 8 | 9 | 7 |
| TCGA-AA-3685 | 9 | 8 | 9 | 8 |
| TCGA-CM-6164 | 9 | 8 | 9 | 7 |
| TCGA-AY-4070 | 9 | 7 | 9 | 7 |
| TCGA-A6-A565 | 9 | 8 | 9 | 8 |
| TCGA-AA-3697 | 9 | 8 | 9 | 8 |
| TCGA-AA-3514 | 9 | 7 | 9 | 7 |
| TCGA-DM-A28C | 9 | 7 | 8 | 6 |
| TCGA-A6-6650 | 9 | 7 | 9 | 7 |
| TCGA-CA-6718 | 9 | 9 | 9 | 9 |
| TCGA-AA-3660 | 9 | 7 | 9 | 7 |
| TCGA-D5-6533 | 9 | 7 | 8 | 7 |
| TCGA-AA-A02O | 9 | 7 | 9 | 7 |
| TCGA-AA-3852 | 9 | 8 | 9 | 7 |
| TCGA-DM-A1D7 | 9 | 7 | 9 | 7 |
| TCGA-AU-3779 | 9 | 8 | 9 | 8 |
| TCGA-G4-6302 | 9 | 7 | 8 | 7 |
| TCGA-G4-6295 | 9 | 8 | 9 | 8 |
| TCGA-CM-6680 | 9 | 8 | 8 | 7 |
| TCGA-AA-3556 | 9 | 8 | 9 | 8 |
| TCGA-AA-3818 | 9 | 7 | 8 | 7 |
| TCGA-AA-3970 | 9 | 8 | 9 | 8 |
| TCGA-AA-A02W | 9 | 7 | 8 | 6 |
| TCGA-AA-A02J | 9 | 7 | 8 | 6 |
| TCGA-CK-4947 | 9 | 8 | 9 | 8 |
| TCGA-AA-3994 | 9 | 8 | 9 | 8 |
| TCGA-AA-A02R | 9 | 9 | 9 | 8 |
| TCGA-CM-6679 | 9 | 7 | 8 | 7 |
| TCGA-CA-6715 | 9 | 7 | 8 | 6 |
| TCGA-AA-3854 | 9 | 7 | 8 | 7 |
| TCGA-AA-3956 | 9 | 8 | 9 | 7 |
| TCGA-A6-2681 | 9 | 7 | 8 | 7 |
| TCGA-AA-3841 | 9 | 8 | 9 | 8 |
| TCGA-A6-2676 | 9 | 9 | 9 | 9 |
| TCGA-A6-3808 | 9 | 8 | 9 | 8 |
| TCGA-AA-3842 | 9 | 8 | 9 | 7 |
| TCGA-CM-6163 | 9 | 7 | 8 | 7 |
| TCGA-AA-A01C | 9 | 8 | 9 | 7 |
| TCGA-AA-3939 | 9 | 7 | 9 | 7 |
| TCGA-AA-3930 | 9 | 8 | 9 | 7 |
| TCGA-NH-A6GA | 9 | 7 | 9 | 7 |
| TCGA-AA-3941 | 9 | 7 | 9 | 7 |
| TCGA-AA-3875 | 9 | 7 | 8 | 7 |
| TCGA-CM-6674 | 9 | 8 | 9 | 7 |
| TCGA-G4-6304 | 9 | 8 | 9 | 7 |
| TCGA-AZ-6603 | 9 | 7 | 8 | 7 |
| TCGA-A6-6141 | 9 | 8 | 8 | 7 |
| TCGA-AA-3982 | 9 | 8 | 9 | 7 |
| TCGA-CM-5864 | 9 | 8 | 9 | 7 |
| TCGA-AA-3955 | 9 | 7 | 8 | 7 |
| TCGA-D5-6931 | 9 | 8 | 8 | 7 |
| TCGA-G4-6625 | 9 | 9 | 9 | 9 |
| TCGA-AA-3520 | 9 | 8 | 9 | 7 |
| TCGA-AA-A00F | 9 | 7 | 8 | 7 |
| TCGA-G4-6297 | 9 | 8 | 9 | 8 |
| TCGA-AA-3673 | 9 | 7 | 8 | 7 |
| TCGA-AA-A00Q | 9 | 7 | 8 | 7 |
| TCGA-A6-6648 | 9 | 8 | 9 | 7 |
| TCGA-DM-A280 | 9 | 7 | 8 | 7 |
| TCGA-AA-A02H | 9 | 7 | 9 | 7 |
| TCGA-F4-6809 | 9 | 8 | 9 | 7 |
| TCGA-AA-3544 | 9 | 8 | 9 | 8 |
| TCGA-AD-6890 | 9 | 8 | 9 | 8 |
| TCGA-AD-A5EK | 9 | 7 | 8 | 6 |
| TCGA-CM-5344 | 9 | 7 | 8 | 6 |
| TCGA-G4-6317 | 9 | 7 | 8 | 7 |
| TCGA-4T-AA8H | 9 | 7 | 9 | 7 |
| TCGA-AD-6899 | 9 | 8 | 8 | 7 |
| TCGA-AZ-5407 | 9 | 7 | 9 | 7 |
| TCGA-A6-3810 | 9 | 8 | 9 | 7 |
| TCGA-DM-A0XF | 9 | 7 | 8 | 7 |
| TCGA-AA-3553 | 9 | 8 | 9 | 8 |
| TCGA-NH-A50U | 9 | 7 | 8 | 7 |
| TCGA-F4-6569 | 9 | 7 | 8 | 7 |
| TCGA-QG-A5YV | 9 | 7 | 8 | 7 |
| TCGA-AD-6965 | 9 | 8 | 9 | 7 |
| TCGA-CM-6167 | 9 | 7 | 8 | 7 |
| TCGA-QG-A5YW | 9 | 8 | 9 | 7 |
| TCGA-AA-3845 | 9 | 9 | 8 | 9 |
| TCGA-AA-3715 | 9 | 9 | 9 | 9 |
| TCGA-AZ-6600 | 9 | 8 | 9 | 7 |
| TCGA-AA-3979 | 9 | 8 | 9 | 7 |
| TCGA-AA-A00Z | 9 | 8 | 9 | 7 |
| TCGA-AY-A71X | 9 | 7 | 9 | 7 |
| TCGA-AA-A024 | 9 | 8 | 9 | 8 |
| TCGA-AA-3989 | 9 | 8 | 8 | 7 |
| TCGA-AA-3667 | 9 | 8 | 9 | 7 |
| TCGA-D5-6539 | 9 | 8 | 9 | 7 |
| TCGA-G4-6315 | 9 | 7 | 8 | 6 |
| TCGA-AA-3968 | 9 | 8 | 8 | 7 |
| TCGA-AZ-4684 | 9 | 7 | 8 | 7 |
| TCGA-AA-3870 | 9 | 8 | 8 | 8 |
| TCGA-DM-A1D9 | 9 | 7 | 8 | 6 |
| TCGA-AZ-4615 | 9 | 9 | 9 | 8 |
| TCGA-CM-6678 | 9 | 7 | 8 | 6 |
| TCGA-DM-A1DB | 9 | 8 | 9 | 7 |
| TCGA-G4-6309 | 9 | 8 | 9 | 7 |
| TCGA-D5-6531 | 9 | 8 | 8 | 8 |
| TCGA-D5-6540 | 9 | 8 | 9 | 7 |
| TCGA-CM-5863 | 9 | 7 | 8 | 6 |
| TCGA-AA-3549 | 9 | 8 | 8 | 7 |
| TCGA-AA-3666 | 9 | 8 | 9 | 8 |
| TCGA-5M-AATE | 9 | 7 | 9 | 7 |
| TCGA-DM-A282 | 9 | 7 | 8 | 6 |
| TCGA-5M-AATA | 9 | 7 | 8 | 7 |
| TCGA-DC-6154 | 9 | 7 | 8 | 6 |
| TCGA-CI-6624 | 9 | 7 | 8 | 7 |
| TCGA-EI-6881 | 9 | 8 | 9 | 8 |
| TCGA-AG-A011 | 9 | 7 | 8 | 7 |
| TCGA-DC-6681 | 9 | 8 | 9 | 7 |
| TCGA-EI-6514 | 9 | 8 | 9 | 7 |
| TCGA-AG-3593 | 9 | 7 | 8 | 7 |
| TCGA-AH-6903 | 9 | 8 | 9 | 7 |
| TCGA-AG-A01N | 9 | 6 | 8 | 6 |
| TCGA-DY-A1DC | 9 | 7 | 9 | 7 |
| TCGA-AG-3592 | 9 | 8 | 9 | 7 |
| TCGA-AG-3726 | 9 | 7 | 8 | 7 |
| TCGA-EI-6512 | 9 | 7 | 8 | 7 |
| TCGA-DC-6160 | 9 | 8 | 9 | 7 |
| TCGA-AF-2693 | 9 | 8 | 9 | 8 |
| TCGA-AG-3581 | 9 | 7 | 8 | 7 |
| TCGA-AG-A026 | 9 | 7 | 8 | 6 |
| TCGA-AG-4015 | 9 | 8 | 9 | 7 |
| TCGA-AG-4022 | 9 | 7 | 9 | 7 |
| TCGA-AG-A015 | 9 | 7 | 8 | 7 |
| TCGA-G5-6641 | 9 | 7 | 9 | 7 |
| TCGA-F5-6571 | 9 | 8 | 8 | 8 |
| TCGA-AG-3612 | 9 | 7 | 8 | 7 |
| TCGA-DC-6683 | 9 | 7 | 8 | 6 |
| TCGA-AG-4005 | 9 | 7 | 8 | 7 |
| TCGA-AF-3911 | 9 | 8 | 9 | 7 |
| TCGA-EF-5830 | 9 | 7 | 8 | 7 |
| TCGA-AG-3605 | 9 | 7 | 8 | 7 |
| TCGA-AG-3732 | 9 | 8 | 9 | 8 |
| TCGA-EI-6513 | 9 | 7 | 8 | 7 |
| TCGA-AG-3586 | 9 | 8 | 9 | 8 |
| TCGA-F5-6813 | 9 | 8 | 9 | 8 |
| TCGA-AG-3896 | 9 | 8 | 9 | 7 |
| TCGA-CI-6623 | 9 | 7 | 8 | 7 |
| TCGA-AH-6547 | 9 | 8 | 8 | 7 |
| TCGA-AG-A016 | 9 | 7 | 9 | 7 |
| TCGA-AG-3582 | 9 | 8 | 8 | 7 |
| TCGA-CL-5918 | 9 | 7 | 9 | 7 |
| TCGA-AG-3892 | 9 | 9 | 9 | 9 |
| TCGA-AG-3725 | 9 | 8 | 9 | 8 |
| TCGA-AG-3878 | 9 | 8 | 9 | 8 |
| TCGA-AG-4008 | 9 | 7 | 8 | 7 |
| TCGA-AG-A01W | 9 | 8 | 9 | 7 |
| TCGA-F5-6861 | 9 | 7 | 8 | 6 |
| TCGA-EF-5831 | 9 | 7 | 8 | 7 |
| TCGA-AG-A02N | 9 | 8 | 9 | 7 |
| TCGA-DY-A1DD | 9 | 7 | 8 | 6 |
| TCGA-AG-3609 | 9 | 8 | 9 | 8 |
| TCGA-AG-A014 | 9 | 7 | 8 | 7 |
| TCGA-DY-A1DE | 9 | 8 | 9 | 8 |
| TCGA-AG-3580 | 9 | 8 | 9 | 7 |
| TCGA-AG-3898 | 9 | 8 | 9 | 7 |
| TCGA-AG-3583 | 9 | 8 | 9 | 7 |
| TCGA-AF-6136 | 9 | 8 | 9 | 7 |
| TCGA-AG-3575 | 9 | 9 | 9 | 8 |
| TCGA-AF-4110 | 9 | 8 | 9 | 8 |
| TCGA-AF-6672 | 9 | 8 | 9 | 7 |
| TCGA-DC-6157 | 9 | 7 | 8 | 7 |
| TCGA-DC-4745 | 9 | 7 | 9 | 7 |
| TCGA-AG-3881 | 9 | 8 | 9 | 8 |
| TCGA-AG-3883 | 9 | 8 | 9 | 8 |
| TCGA-AG-A025 | 9 | 8 | 9 | 7 |
| TCGA-AG-3893 | 9 | 8 | 9 | 7 |
| TCGA-AG-A00Y | 9 | 8 | 9 | 7 |
| TCGA-AG-A00H | 9 | 7 | 8 | 6 |
| TCGA-EI-6882 | 9 | 8 | 8 | 7 |
| TCGA-EI-6506 | 9 | 8 | 9 | 8 |
| TCGA-AG-3902 | 9 | 9 | 9 | 9 |
| TCGA-AF-A56L | 9 | 7 | 8 | 7 |
| TCGA-AG-3587 | 9 | 8 | 9 | 7 |
| TCGA-AG-3594 | 9 | 9 | 9 | 8 |
| TCGA-DY-A1DF | 9 | 7 | 8 | 6 |
| TCGA-F5-6465 | 9 | 8 | 8 | 8 |
| TCGA-CI-6620 | 9 | 7 | 8 | 7 |
| TCGA-EI-6884 | 9 | 8 | 9 | 7 |
| TCGA-F5-6811 | 9 | 8 | 9 | 7 |
| TCGA-AG-3727 | 9 | 8 | 8 | 7 |
| TCGA-DC-5869 | 9 | 7 | 8 | 7 |
| TCGA-BM-6198 | 9 | 8 | 9 | 8 |
| TCGA-AG-A008 | 9 | 8 | 9 | 7 |
| TCGA-G5-6233 | 9 | 8 | 9 | 7 |
| TCGA-AA-3977 | 8 | 7 | 8 | 7 |
| TCGA-CM-6161 | 8 | 7 | 8 | 6 |
| TCGA-CK-5916 | 8 | 7 | 7 | 7 |
| TCGA-D5-6541 | 8 | 7 | 8 | 7 |
| TCGA-A6-2678 | 8 | 7 | 8 | 6 |
| TCGA-CM-6676 | 8 | 6 | 7 | 5 |
| TCGA-A6-2684 | 8 | 7 | 8 | 7 |
| TCGA-AA-A01Q | 8 | 7 | 8 | 7 |
| TCGA-A6-5665 | 8 | 7 | 8 | 6 |
| TCGA-AA-3833 | 8 | 7 | 8 | 7 |
| TCGA-D5-5538 | 8 | 7 | 8 | 7 |
| TCGA-A6-5660 | 8 | 6 | 8 | 6 |
| TCGA-A6-6651 | 8 | 7 | 7 | 6 |
| TCGA-DM-A1D0 | 8 | 6 | 7 | 5 |
| TCGA-F4-6855 | 8 | 6 | 7 | 6 |
| TCGA-AD-5900 | 8 | 7 | 8 | 7 |
| TCGA-5M-AAT4 | 8 | 7 | 8 | 6 |
| TCGA-D5-6932 | 8 | 7 | 8 | 7 |
| TCGA-CM-5349 | 8 | 6 | 7 | 6 |
| TCGA-F4-6854 | 8 | 7 | 8 | 6 |
| TCGA-G4-6303 | 8 | 6 | 7 | 6 |
| TCGA-AM-5820 | 8 | 6 | 7 | 6 |
| TCGA-A6-5667 | 8 | 6 | 8 | 6 |
| TCGA-AY-6196 | 8 | 8 | 8 | 8 |
| TCGA-CK-4951 | 8 | 7 | 8 | 7 |
| TCGA-AA-A01X | 8 | 6 | 7 | 5 |
| TCGA-AA-A00O | 8 | 7 | 8 | 6 |
| TCGA-D5-5540 | 8 | 6 | 7 | 6 |
| TCGA-A6-2675 | 8 | 7 | 8 | 7 |
| TCGA-AA-3713 | 8 | 7 | 8 | 7 |
| TCGA-CM-6168 | 8 | 7 | 8 | 7 |
| TCGA-CM-6166 | 8 | 6 | 7 | 5 |
| TCGA-AZ-5403 | 8 | 6 | 7 | 6 |
| TCGA-AA-3492 | 8 | 7 | 8 | 7 |
| TCGA-A6-3809 | 8 | 8 | 8 | 8 |
| TCGA-AA-3543 | 8 | 8 | 8 | 7 |
| TCGA-D5-5539 | 8 | 7 | 8 | 6 |
| TCGA-D5-6922 | 8 | 7 | 8 | 7 |
| TCGA-D5-5541 | 8 | 7 | 7 | 6 |
| TCGA-A6-6780 | 8 | 8 | 8 | 8 |
| TCGA-CA-6716 | 8 | 6 | 7 | 5 |
| TCGA-A6-4105 | 8 | 7 | 8 | 7 |
| TCGA-AZ-6598 | 8 | 7 | 7 | 6 |
| TCGA-3L-AA1B | 8 | 7 | 8 | 6 |
| TCGA-DM-A285 | 8 | 6 | 8 | 5 |
| TCGA-AA-3696 | 8 | 6 | 7 | 5 |
| TCGA-G4-6314 | 8 | 6 | 8 | 6 |
| TCGA-AA-3538 | 8 | 6 | 7 | 5 |
| TCGA-AA-3678 | 8 | 7 | 8 | 7 |
| TCGA-CK-5915 | 8 | 6 | 7 | 5 |
| TCGA-D5-6529 | 8 | 8 | 8 | 7 |
| TCGA-QG-A5Z1 | 8 | 6 | 7 | 5 |
| TCGA-CA-6719 | 8 | 7 | 8 | 7 |
| TCGA-CM-4746 | 8 | 7 | 8 | 6 |
| TCGA-AA-3984 | 8 | 7 | 8 | 7 |
| TCGA-CM-6165 | 8 | 7 | 8 | 6 |
| TCGA-AA-A02F | 8 | 6 | 7 | 5 |
| TCGA-AZ-4315 | 8 | 7 | 7 | 6 |
| TCGA-AA-3534 | 8 | 6 | 7 | 5 |
| TCGA-A6-5664 | 8 | 7 | 8 | 7 |
| TCGA-D5-6530 | 8 | 7 | 7 | 7 |
| TCGA-AA-3488 | 8 | 7 | 8 | 6 |
| TCGA-A6-A56B | 8 | 6 | 7 | 5 |
| TCGA-G4-6586 | 8 | 7 | 8 | 7 |
| TCGA-A6-2682 | 8 | 6 | 7 | 6 |
| TCGA-G4-6311 | 8 | 7 | 8 | 6 |
| TCGA-AA-3509 | 8 | 7 | 8 | 6 |
| TCGA-CM-6169 | 8 | 7 | 8 | 7 |
| TCGA-A6-2671 | 8 | 7 | 8 | 7 |
| TCGA-G4-6307 | 8 | 6 | 8 | 6 |
| TCGA-CM-4747 | 8 | 7 | 8 | 6 |
| TCGA-A6-2674 | 8 | 8 | 8 | 7 |
| TCGA-CK-4948 | 8 | 6 | 8 | 6 |
| TCGA-AD-6964 | 8 | 8 | 8 | 8 |
| TCGA-AM-5821 | 8 | 9 | 8 | 8 |
| TCGA-AA-3496 | 8 | 8 | 8 | 7 |
| TCGA-NH-A8F7 | 8 | 6 | 8 | 6 |
| TCGA-AA-3494 | 8 | 7 | 8 | 6 |
| TCGA-D5-6536 | 8 | 6 | 8 | 6 |
| TCGA-A6-6781 | 8 | 7 | 7 | 7 |
| TCGA-A6-2672 | 8 | 8 | 8 | 8 |
| TCGA-AD-6901 | 8 | 7 | 8 | 7 |
| TCGA-CM-5348 | 8 | 6 | 7 | 6 |
| TCGA-CM-6170 | 8 | 7 | 8 | 7 |
| TCGA-AA-3542 | 8 | 6 | 8 | 6 |
| TCGA-AA-3521 | 8 | 7 | 8 | 6 |
| TCGA-D5-6534 | 8 | 8 | 8 | 8 |
| TCGA-D5-6537 | 8 | 7 | 8 | 6 |
| TCGA-CM-6162 | 8 | 8 | 8 | 7 |
| TCGA-AA-3489 | 8 | 8 | 8 | 7 |
| TCGA-A6-6142 | 8 | 6 | 7 | 6 |
| TCGA-AA-3554 | 8 | 8 | 8 | 7 |
| TCGA-NH-A50V | 8 | 7 | 8 | 7 |
| TCGA-AA-3864 | 8 | 7 | 8 | 6 |
| TCGA-F4-6570 | 8 | 7 | 7 | 7 |
| TCGA-D5-6928 | 8 | 9 | 8 | 9 |
| TCGA-CM-6171 | 8 | 6 | 7 | 6 |
| TCGA-AA-A017 | 8 | 6 | 8 | 6 |
| TCGA-AA-3692 | 8 | 7 | 8 | 6 |
| TCGA-AA-3831 | 8 | 6 | 7 | 6 |
| TCGA-D5-7000 | 8 | 7 | 8 | 7 |
| TCGA-AA-A00R | 8 | 8 | 8 | 8 |
| TCGA-F4-6459 | 8 | 6 | 7 | 6 |
| TCGA-AA-3664 | 8 | 7 | 8 | 7 |
| TCGA-D5-6924 | 8 | 7 | 8 | 7 |
| TCGA-D5-6538 | 8 | 6 | 7 | 5 |
| TCGA-AA-A00N | 8 | 7 | 8 | 7 |
| TCGA-AA-3529 | 8 | 6 | 8 | 6 |
| TCGA-AA-3867 | 8 | 7 | 8 | 6 |
| TCGA-AA-3949 | 8 | 9 | 8 | 8 |
| TCGA-F4-6463 | 8 | 6 | 8 | 6 |
| TCGA-CM-5862 | 8 | 7 | 8 | 6 |
| TCGA-A6-2685 | 8 | 7 | 8 | 7 |
| TCGA-D5-6923 | 8 | 6 | 7 | 6 |
| TCGA-5M-AAT6 | 8 | 8 | 8 | 8 |
| TCGA-F4-6805 | 8 | 7 | 8 | 7 |
| TCGA-DM-A28G | 8 | 7 | 8 | 6 |
| TCGA-F5-6864 | 8 | 7 | 8 | 6 |
| TCGA-AF-2690 | 8 | 7 | 8 | 7 |
| TCGA-CI-6619 | 8 | 7 | 8 | 7 |
| TCGA-DT-5265 | 8 | 6 | 8 | 6 |
| TCGA-AH-6549 | 8 | 7 | 8 | 6 |
| TCGA-DC-6156 | 8 | 7 | 7 | 6 |
| TCGA-F5-6810 | 8 | 6 | 7 | 6 |
| TCGA-EI-7002 | 8 | 7 | 8 | 6 |
| TCGA-G5-6572 | 8 | 6 | 7 | 6 |
| TCGA-DC-6158 | 8 | 7 | 8 | 7 |
| TCGA-AF-3913 | 8 | 6 | 7 | 6 |
| TCGA-AG-3578 | 8 | 6 | 7 | 6 |
| TCGA-AG-3894 | 8 | 7 | 8 | 6 |
| TCGA-CI-6621 | 8 | 7 | 8 | 7 |
| TCGA-AG-3600 | 8 | 7 | 8 | 7 |
| TCGA-AH-6897 | 8 | 7 | 8 | 6 |
| TCGA-EI-6917 | 8 | 7 | 8 | 7 |
| TCGA-AG-A01Y | 8 | 7 | 8 | 7 |
| TCGA-F5-6702 | 8 | 7 | 7 | 6 |
| TCGA-AG-4001 | 8 | 7 | 8 | 6 |
| TCGA-EI-6508 | 8 | 6 | 8 | 6 |
| TCGA-AH-6644 | 8 | 6 | 8 | 6 |
| TCGA-AF-2687 | 8 | 7 | 8 | 7 |
| TCGA-AF-2692 | 8 | 7 | 8 | 6 |
| TCGA-AG-3731 | 8 | 7 | 8 | 7 |
| TCGA-AG-3742 | 8 | 6 | 8 | 6 |
| TCGA-F5-6863 | 8 | 6 | 7 | 5 |
| TCGA-CL-5917 | 8 | 6 | 7 | 5 |
| TCGA-F5-6464 | 8 | 7 | 7 | 7 |
| TCGA-AF-A56N | 8 | 7 | 8 | 6 |
| TCGA-AF-5654 | 8 | 6 | 7 | 6 |
| TCGA-AG-3999 | 8 | 6 | 7 | 6 |
| TCGA-EI-6509 | 8 | 6 | 8 | 6 |
| TCGA-EI-6511 | 8 | 8 | 8 | 7 |
| TCGA-DC-6155 | 8 | 7 | 8 | 6 |
| TCGA-AG-3601 | 8 | 6 | 8 | 6 |
| TCGA-DY-A0XA | 8 | 7 | 8 | 6 |
| TCGA-AH-6643 | 8 | 7 | 9 | 7 |
| TCGA-AF-A56K | 8 | 6 | 8 | 6 |
| TCGA-AG-3584 | 8 | 7 | 8 | 6 |
| TCGA-EI-7004 | 8 | 7 | 8 | 7 |
| TCGA-AF-6655 | 8 | 7 | 8 | 7 |
| TCGA-AA-3555 | 7 | 6 | 7 | 5 |
| TCGA-D5-6926 | 7 | 7 | 7 | 6 |
| TCGA-A6-2686 | 7 | 7 | 7 | 7 |
| TCGA-CA-6717 | 7 | 6 | 7 | 6 |
| TCGA-D5-6898 | 7 | 6 | 7 | 5 |
| TCGA-AA-A010 | 7 | 6 | 8 | 6 |
| TCGA-F4-6703 | 7 | 7 | 7 | 7 |
| TCGA-G4-6298 | 7 | 5 | 6 | 5 |
| TCGA-AA-3663 | 7 | 6 | 7 | 6 |
| TCGA-A6-A566 | 7 | 6 | 7 | 6 |
| TCGA-AZ-6601 | 7 | 7 | 7 | 7 |
| TCGA-CK-6748 | 7 | 6 | 7 | 5 |
| TCGA-DM-A1HB | 7 | 6 | 7 | 5 |
| TCGA-A6-6654 | 7 | 7 | 7 | 7 |
| TCGA-CK-4952 | 7 | 6 | 7 | 5 |
| TCGA-AA-A00J | 7 | 6 | 7 | 5 |
| TCGA-AZ-6607 | 7 | 7 | 7 | 7 |
| TCGA-AA-A01D | 7 | 5 | 7 | 5 |
| TCGA-AA-3966 | 7 | 6 | 7 | 6 |
| TCGA-DM-A28H | 7 | 6 | 7 | 5 |
| TCGA-AA-3815 | 7 | 7 | 7 | 7 |
| TCGA-NH-A5IV | 7 | 6 | 6 | 5 |
| TCGA-AA-3950 | 7 | 7 | 7 | 7 |
| TCGA-AG-3574 | 7 | 6 | 7 | 5 |
| TCGA-EI-6885 | 7 | 6 | 6 | 6 |
| TCGA-F5-6814 | 7 | 7 | 7 | 7 |
| TCGA-AG-3901 | 7 | 6 | 7 | 6 |
| TCGA-DY-A1H8 | 7 | 6 | 7 | 5 |
| TCGA-AG-4021 | 7 | 5 | 6 | 5 |
| TCGA-AG-A002 | 7 | 5 | 7 | 5 |
| TCGA-AF-3400 | 7 | 7 | 7 | 7 |
| TCGA-D5-6927 | 6 | 6 | 6 | 6 |
| TCGA-WS-AB45 | 6 | 6 | 6 | 6 |
| TCGA-SS-A7HO | 6 | 5 | 6 | 5 |
| TCGA-AD-A5EJ | 6 | 5 | 6 | 5 |
| TCGA-AA-3947 | 6 | 5 | 6 | 5 |
| TCGA-EI-6507 | 6 | 6 | 6 | 6 |
